# RNAi Effector Diversity in Nematodes

**DOI:** 10.1371/journal.pntd.0001176

**Published:** 2011-06-07

**Authors:** Johnathan J. Dalzell, Paul McVeigh, Neil D. Warnock, Makedonka Mitreva, David McK. Bird, Pierre Abad, Colin C. Fleming, Tim A. Day, Angela Mousley, Nikki J. Marks, Aaron G. Maule

**Affiliations:** 1 Molecular Biosciences-Parasitology, School of Biological Sciences, Queen's University Belfast, Belfast, United Kingdom; 2 The Genome Center, Washington University School of Medicine, St. Louis, Missouri, United States of America; 3 Department of Plant Pathology, North Carolina State University, Raleigh, North Carolina, United States of America; 4 INRA, Unité Interactions Plantes-Microorganismes et Santé Végétale, Antibes, France; 5 Agri-Food and Biosciences Institute, Belfast, United Kingdom; 6 Department of Biomedical Sciences, Iowa State University, Ames, Iowa, United States of America; Biomedical Research Institute, United States of America

## Abstract

While RNA interference (RNAi) has been deployed to facilitate gene function studies in diverse helminths, parasitic nematodes appear variably susceptible. To test if this is due to inter-species differences in RNAi effector complements, we performed a primary sequence similarity survey for orthologs of 77 *Caenorhabditis elegans* RNAi pathway proteins in 13 nematode species for which genomic or transcriptomic datasets were available, with all outputs subjected to domain-structure verification. Our dataset spanned transcriptomes of *Ancylostoma caninum* and *Oesophagostomum dentatum*, and genomes of *Trichinella spiralis, Ascaris suum*, *Brugia malayi*, *Haemonchus contortus*, *Meloidogyne hapla*, *Meloidogyne incognita* and *Pristionchus pacificus*, as well as the *Caenorhabditis* species *C. brenneri*, *C. briggsae*, *C. japonica* and *C. remanei*, and revealed that: (i) Most of the *C. elegans* proteins responsible for uptake and spread of exogenously applied double stranded (ds)RNA are absent from parasitic species, including RNAi-competent plant-nematodes; (ii) The Argonautes (AGOs) responsible for gene expression regulation in *C. elegans* are broadly conserved, unlike those recruited during the induction of RNAi by exogenous dsRNA; (iii) Secondary Argonautes (SAGOs) are poorly conserved, and the nuclear AGO NRDE-3 was not identified in any parasite; (iv) All five *Caenorhabditis* spp. possess an expanded RNAi effector repertoire relative to the parasitic nematodes, consistent with the propensity for gene loss in nematode parasites; (v) In spite of the quantitative differences in RNAi effector complements across nematode species, all displayed qualitatively similar coverage of functional protein groups. In summary, we could not identify RNAi effector deficiencies that associate with reduced susceptibility in parasitic nematodes. Indeed, similarities in the RNAi effector complements of RNAi refractory and competent nematode parasites support the broad applicability of this research genetic tool in nematodes.

## Author Summary

Many organisms regulate gene expression through an RNA interference (RNAi) pathway, first characterized in the nematode *Caenorhabditis elegans*. This pathway can be triggered experimentally using double-stranded (ds)RNA to selected gene targets, thereby allowing researchers to ‘silence’ individual genes and so investigate their function. It is hoped that this technology will facilitate gene silencing in important parasitic nematodes that impose a considerable health and economic burden on mankind. Unfortunately, differences in RNAi susceptibility have been observed between species. Here we investigated the possibility that differences in the complement of effector proteins involved in the RNAi pathway are responsible for these differences in susceptibility. Our data revealed that most facets of the RNAi pathway are well represented across parasitic nematodes, although there were fewer pathway proteins in other nematodes compared to *C. elegans*. In contrast, the proteins responsible for uptake and spread of dsRNA are not well represented in parasitic nematodes. However, the importance of these differences is undermined by our observation that the protein complements in all the parasites were qualitatively similar, regardless of RNAi-susceptibility. Clearly, differences in the RNAi pathway of parasitic nematodes do not explain the variations in susceptibility to experimental RNAi.

## Introduction

RNA interference (RNAi) is a reverse genetics technique which permits the ablation of mRNA by introduction of complementary double-stranded RNA (dsRNA), through cellular mechanisms common to most eukaryotes (for review, see [1]) and provides a functional genomics platform in a range of organisms, including those intractable to traditional genetic manipulations. One such group of organisms are the parasitic nematodes for which there have been recent expansions in transcriptomic and genomic datasets [2]–[5].

Several groups have attempted to apply the RNAi protocols pioneered in *Caenorhabditis elegans* to parasitic nematodes. Significant progress has been made in plant-parasitic nematodes (PPNs) in which RNAi is an established experimental technique [6]–[8], and may have utility for parasite control in plants genetically engineered to express PPN-transcript-specific dsRNA [9], [10]. In contrast, RNAi experiments in animal- and human-parasitic nematodes have had variable levels of success (for reviews, see [11]–[13]). Of note are experiments reporting inefficient or inconsistent transcript knockdown, highlighted by successful silencing of only 3 of 8 *Ostertagia ostertagi* genes [14] and 2 of 11 *Haemonchus contortus* genes [15]. In *H. contortus*, one feature of successful RNAi appears to be the location of target gene expression, since genes predicted to be expressed in environmentally-exposed tissues are more readily silenced [16]. RNAi difficulties have also been seen in *Heligmosomoides polygyrus* [17] and the non-parasitic species *Pristionchus pacificus* and *Oscheius* sp1 CEW1 [18]–[20]. Notably, inter-species differences are apparent even within the genus *Caenorhabditis*, where *C. briggsae* (unlike *C. elegans*) is unable to take up dsRNA from the environment, due to a SID-2 which displays aberrant RNAi functionality [21].

Hypotheses to explain RNAi difficulties in parasitic nematodes have been reported, and include: (i) the lack of appropriate *in vitro* culture systems for parasitic nematodes [15]; (ii) inappropriate methods of dsRNA delivery, i.e. delivered externally, where microinjection directly into the worm is more effective in *C. elegans* [13]; (iii) differences in RNAi effector protein functionality [13], [15]; and (iv) differences in the complement of RNAi effectors between nematodes [12], [13], [15], [17]. The latter hypothesis has been confirmed for the apicomplexan *Plasmodium* spp. (the causative agents of malaria), which are refractory to RNAi due to deficiencies in key pathway components [22]–[24]. Here, we test this hypothesis in nematodes by investigating the complement of RNAi pathway proteins in selected nematode datasets. Using 77 *C. elegans* RNAi pathway proteins as query sequences, we performed BLAST trawls of nematode-derived genomic and transcriptomic resources. Our searches focused on high-quality sequence datasets, including the draft genomes of *Trichinella spiralis* (Clade I/clade 2; here and throughout, we utilize clade delineations of both Blaxter *et al.* (denoted clades I–V [25]) and Holterman *et al.* (denoted clades 1–12 [26]), *Ascaris suum* (Clade III/clade 8), *Brugia malayi* (Clade III/clade 8), *Meloidogyne incognita* (Clade IV/clade 12), *Meloidogyne hapla* (Clade IV/clade 12), *Caenorhabditis brenneri*, *Caenorhabditis briggsae*, *Caenorhabditis japonica*, *Caenorhabditis remanei* (Clade V/clade 9), *Haemonchus contortus* (Clade V/clade 9), and *Pristionchus pacificus* (Clade V/clade 9) as well as the transcriptomes of *Oesophagostomum dentatum* (Clade V/clade 9) and *Ancylostoma caninum* (Clade V/clade 9). We find that the RNAi effector complements of these species, whilst quantitatively different are qualitatively similar with regard to the presence of functional groupings, yielding no major inter-species differences except that all were notably less diverse than in *Caenorhabditis* spp. These data suggest that variable susceptibilities to RNAi amongst parasitic nematodes cannot be adequately explained by differences in RNAi effector complement between such species.

## Materials and Methods

### Reciprocal BLAST Methodology

**Figure 1:**
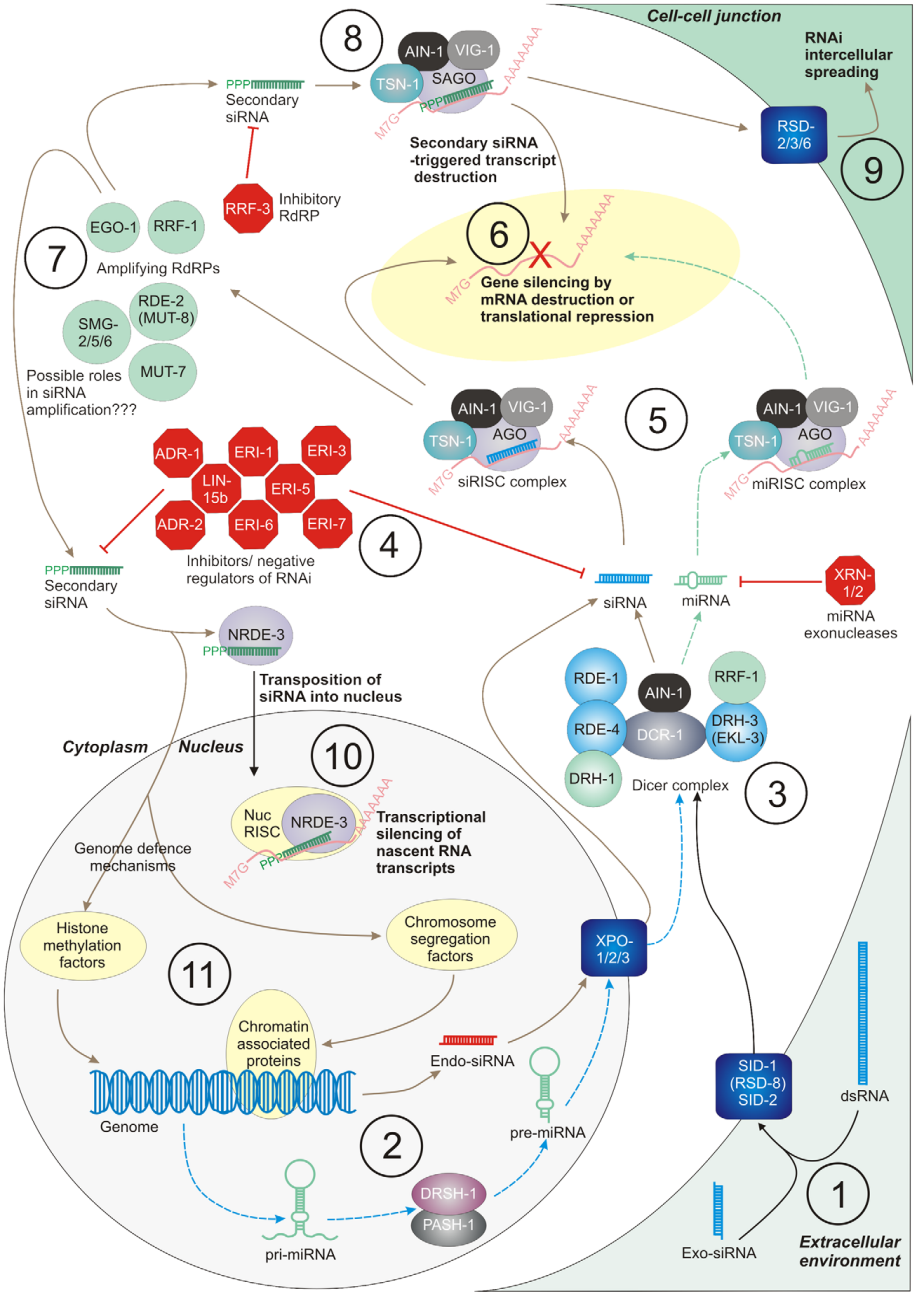
Core components of the *Caenorhabditis elegans* RNA interference (RNAi) pathway. (1) Exogenously applied double-stranded RNA (dsRNA) and small interfering RNA (exo-siRNA) are thought to enter cells via SID (Systemic RNA Interference Defective) proteins SID-1/RSD-8 and SID-2. (2) Endogenous RNAi-based pathways begin in the nucleus; micro-interfering RNA (miRNA) synthesis begins with transcription of hairpin-looped primary miRNA (pri-miRNA) transcripts from intergenic, intronic or antisense regions. pri-miRNAs are processed by the DRSH-1/PASH-1 complex to pre-miRNA, which are exported from the nucleus by exportin proteins XPO-1, -2 and -3. Endogenous siRNAs (endo-siRNAs) are also produced from genomic regions, and exported by XPO-1, 2, and -3. (3) Both pre-miRNAs and exogenously applied dsRNA molecules are bound and cleaved by the dicer complex, which consists of the RNAse III-like nuclease DCR-1, the dsRNA-binding proteins RDE-1 and -4, the helicases DRH-1 and DRH-3/EKL-3, the RNA-dependent RNA-polymerase (RdRP) RRF-1, and the uncharacterized protein, AIN-1. Dicer cleaves dsRNA to produce siRNA molecules, and pre-miRNA to mature miRNA, both of which are substrates for the RNA-induced silencing complex (RISC). (4) Both siRNAs and miRNAs are the focus of a battery of inhibitors, which allow down-regulation of the RNAi response. (5) The RISC complex incorporates a single strand of miRNA or siRNA (termed the guide strand), and binds a complementary mRNA strand, eliciting gene silencing by either mRNA destruction or translational repression (6). The central catalytic component of RISC is an argonaute (AGO) protein, allied with the nuclease TSN-1, the RNA-binding protein VIG-1, and AIN-1. (7) The RNAi response may be amplified by the action of the RdRPs RRF-1 and -2, SMG-5, RDE-2/MUT-8 and MUT-7, which produce a population of single-stranded RNAs bearing N-terminal tri-phosphates from a target mRNA template. (8) These secondary siRNAs interact with Secondary-siRNA-specific AGOs (SAGO-1 and -2), terminating in down-regulation of target transcript. Secondary siRNAs can also spread between cells through RSD-2, -3 and -6, resulting in intercellular spread of the RNAi effect (9), and can be imported into the nucleus by NRDE-3, which elicits transcriptional silencing of nascent RNA transcripts as part of nuclear RISC (nucRISC) (10). siRNAs may also control aspects of nuclear RNAi, including histone methylation, chromatin formation and chromosome segregation (11). Dashed lines indicate miRNA-based pathways, solid lines indicate siRNA-based pathways.

Seventy-seven *C. elegans* proteins known to be involved in core aspects of RNAi were identified from literature (Figure 1). These proteins were separated into five core functional groups; namely, small RNA biosynthesis, dsRNA uptake and spreading, AGOs and RISC, RNAi inhibitors, and nuclear effectors. Protein sequences were retrieved from WormBase (www.wormbase.org; release WS206) and used as search strings in a series of primary translated nucleotide (tBLASTn) and protein BLASTs (BLASTp) [27] against genome and transcriptome databases described below. All primary BLAST hits returning with a bitscore ≥40 and an expect value ≤0.01 were manually translated to amino acid sequence in six reading frames (www.expasy.ch/tools/dna.html), and analysed for identity and domain structure by BLASTp (through NCBI's Conserved Domain Database service) and InterProScan (www.ebi.ac.uk/Tools/InterProScan). The appropriate reading frame in each case (usually that with the largest uninterrupted open reading frame [ORF], however this was determined empirically on a case by case basis) was then subjected to reciprocal tBLASTn and BLASTp against the *C. elegans* non-redundant nucleotide and protein databases on the NCBI BLAST server (http://www.ncbi.nlm.nih.gov/BLAST), using default settings. The identity of the top-scoring reciprocal BLAST hit was accepted as identity of the relevant primary hit, as long as that identity was also supported by domain structure analysis (see Datasets S1, S2, S3, S4, S5). In the case of *H. contortus*, primary tBLASTn searches were performed and the separate high scoring return sequences were concatenated into a single sequence (to facilitate reciprocation) and used as reciprocal tBLASTn and BLASTp searches against *C. elegans*, as before.

### Databases

The *M. incognita* (http://www.inra.fr/meloidogyne_incognita/genomic_resources) and *B. malayi* (http://blast.jcvi.org/er-blast/index.cgi?project=bma1) genomes were searched using BLASTp to predicted protein sets, in addition to tBLASTn against available contig assembly, unplaced reads and associated ESTs [3], [5]. The *M. hapla* genome was searched using BLASTp against public release 4 (HapPep4: www.hapla.org) of the hand annotated and experimentally-validated *M. hapla* protein set [28], in addition to tBLASTn against the 10× contig assembly [4]. The *H. contortus* genome was searched using tBLASTn against the supercontig 26/08/09 database (http://www.sanger.ac.uk/cgi-bin/blast/submitblast/h_contortus). *A. suum*, *A. caninum*, *T. spiralis* and *O. dentatum* primary BLASTp and tBLASTn searches were performed using the datasets generated at Washington University, St Louis (available at www.nematode.net, [29]), as above; reciprocal BLAST searches against *C. elegans* datasets were then performed as before. Using the core eukaryotic genes as a reference [30], we estimated that 93% of the *A. caninum* [31]; and 87% of the *O. dentatum* transcriptome is identified, making these two dataset comparable to the full proteomes predicted from the genomes of the other species included in this study. *C. brenneri*, *C. briggsae*, *C. japonica* and *C. remanei* datasets were accessed through WormBase. Searches were also performed against publically-available nematode expressed sequence tags (ESTs) available through GenBank (www.ncbi.nlm.nih.gov), using methods as described above.

## Results and Discussion

In the absence of tractable methods for transgenesis or genetic manipulation, RNAi represents one of the few molecular genetics tools that can currently be applied to parasitic nematodes. However, reports documenting difficulties in the application of RNAi to some nematodes [11]–[13], [16], [17] suggest diminished potential for RNAi-based gene function and target validation studies in some species. We have employed primary sequence similarity-based methodology to identify putative orthologs of *C. elegans* RNAi pathway (Figure 1) proteins in a dataset of 13 nematode genomes/transcriptomes, as a means of investigating the inter-species conservation of RNAi effectors which might inform the wider utility of RNAi in parasitic nematodes. We selected these datasets in the first instance as those represented by predicted protein sets, which were most consistent with our primary protein similarity-based search methodology. Latterly, we extended our searches to include the publically-available genome construct of *H. contortus* (which at the time of searching lacked adequate gene predictions), due to the central importance of this species to the question of RNAi's applicability to animal-parasitic nematodes. While we recognise that we have omitted several other publically-available nematode genome datasets from our analyses, we considered that they did not meet our criteria for inclusion, as described above. Whilst several authors refer to the apparent presence/absence of a few RNAi effector proteins in single nematode species, one small scale study examined the occurrence of 18 such proteins across *H. contortus*, *B. malayi* and the flatworm parasite *Schistosoma mansoni* [17]. The putative orthologs that we identified are summarised in Tables 1–5, with corresponding protein sequences described in Datasets S1, S2, S3, S4, S5. While we addressed publically-available nematode ESTs in our searches, these contributed little to our analyses due to the fragmentary nature of their coverage of RNAi effector proteins (see Table S1).

**Table 1:** Small RNA biosynthetic proteins.

		*C. elegans* orthologs								
Clade	Species	*drh-3*	*drsh-1*	*xpo-1*	*xpo-2*	*dcr-1*	*drh-1*	*pash-1*	*rde-4*	*xpo-3*
I/2	*Trichinella spiralis*	X	X	X	X	X	X			
III/8	*Ascaris suum*	X	X	X	X	X	X			X
III/8	*Brugia malayi*	X	X	X	X	X	X	X	X	X
IV/12	*Meloidogyne hapla*	X	X	X	X		X	X		
IV/12	*Meloidogyne incognita*	X	X	X	X	X	X	X		
V/9	*Ancylostoma caninum*	X	X	X	X	X	X		X	
V/9	*Caenorhabditis brenneri*	X	X	X	X	X	X	X	X	X
V/9	*Caenorhabditis briggsae*	X	X	X	X	X	X	X	X	X
V/9	*Caenorhabditis japonica*	X	X	X	X	X	X	X	X	X
V/9	*Caenorhabditis remanei*	X	X	X	X	X	X	X	X	X
V/9	*Haemonchus contortus*	X	X	X	X	X	X	X		
V/9	*Oesophagostomum dentatum*	X	X	X	X	X	X			
V/9	*Pristionchus pacificus*	X	X	X	X	X		X		

Species represented solely by expressed sequence tag (EST) datasets are not included, refer to Table S1. ‘X’ indicates presence of ortholog. Note that *drh-2* is not included due to its sole presence as a pseudogene in *Caenorhabditis elegans*. See Dataset S1 for corresponding protein sequences.

**Table 2 pntd-0001176-t002:** dsRNA uptake and spreading, and siRNA amplification effectors.

		*C. elegans* orthologs										
		Amplification Proteins							Spreading Proteins			
Clade	Species	*smg-2*	*smg-6*	*ego-1*	*rrf-3*	*rrf-1*	*smg-5*	*rsd-2*	*rsd-3*	*sid-1*	*rsd-6*	*sid-2*
I/2	*Trichinella spiralis*	X	X		X				X			
III/8	*Ascaris suum*	X	X	X	X	X			X			
III/8	*Brugia malayi*	X	X	X	X				X			
IV/12	*Meloidogyne hapla*	X	X	X					X			
IV/12	*Meloidogyne incognita*	X	X	X					X			
V/9	*Ancylostoma caninum*	X	X		X				X			
V/9	*Caenorhabditis brenneri*	X	X	X	X	X	X		X	X	X	X
V/9	*Caenorhabditis briggsae*	X	X	X	X	X	X	X	X	X	X	X
V/9	*Caenorhabditis japonica*	X	X	X	X		X	X	X	X	X	X
V/9	*Caenorhabditis remanei*	X		X	X	X	X	X	X	X	X	X
V/9	*Haemonchus contortus*	X	X	X	X				X	X		
V/9	*Oesophagostomum dentatum*	X	X	X					X	X		
V/9	*Pristionchus pacificus*	X	X	X	X				X		X	

Species represented solely by expressed sequence tag (EST) datasets are not included, refer to Table S1. ‘X’ indicates presence of ortholog. Note that *rrf-2* is not shown, as it may represent a pseudogene found only in *Caenorhabditis elegans*. See Dataset S2 for corresponding protein sequences.

**Table 3 pntd-0001176-t003:** Argonautes (AGOs) and RNA-induced Silencing Complex (RISC) components.

		*C. elegans* orthologs																													
		Argonautes																										RISC Proteins			
Clade	Species	*alg-1*	R06C7.1	C04F12.1	F58G1.1	*alg-4*	*rde-1*	C16C10.3	*ppw-1*	*csr-1*	*ppw-2*	*sago-1*	T22B3.2	T22H9.3	*alg-2*	*ergo-1*	*prg-1*	F55A12.1	T23D8.7	nrde-3	*sago-2*	T23B3.2	Y49F6A.1	ZK1248.7	*prg-2*	C06A1.4^a^	C14B1.7^a^	*tsn-1*	*ain-1* a	*vig-1*	*ain-2* a
I/2	*Trichinella spiralis*	X				X									X	X												X			
III/8	*Ascaris suum*	X	X	X	X	X	X	X	X	X		X	X		X			X						X				X	X	X	
III/8	*Brugia malayi*	X	X	X	X																							X	X	X	X
IV/12	*Meloidogyne hapla*	X	X	X	X										X													X	X		
IV/12	*Meloidogyne incognita*	X	X	X	X	X								X	X													X	X		
V/9	*Ancylostoma caninum*	X	X	X	X	X	X	X		X	X	X		X			X	X						X				X	X	X	
V/9	*Caenorhabditis brenneri*	X	X	X	X		X	X	X	X		X	X			X	X		X	X	X	X	X					X	X	X	X
V/9	*Caenorhabditis briggsae*	X	X	X	X	X	X	X	X	X	X	X				X	X		X	X	X		X					X	X	X	X
V/9	*Caenorhabditis japonica*	X	X				X	X	X	X	X	X	X	X		X	X		X	X		X						X	X	X	
V/9	*Caenorhabditis remanei*	X	X	X	X		X	X	X	X	X	X	X	X		X			X	X	X	X	X					X	X	X	X
V/9	*Haemonchuscontortus*	X	X	X	X	X	X	X	X		X		X		X			X	X			X		X	X	X	X		X		
V/9	*Oesophagostomum dentatum*	X	X	X	X	X					X			X				X			X			X	X			X	X	X	
V/9	*Pristionchus pacificus*	X	X	X		X	X	X	X				X	X			X	X					X					X		X	

a Pseudogene in *C. elegans*.

Species represented solely by expressed sequence tag (EST) datasets are not included, refer to Table S1. ‘X’ indicates presence of ortholog. *Caenorhabditis elegans*-specific argonautes are not shown (M03D4.7; ZK218.8). See Dataset S3 for corresponding protein sequences.

**Table 4 pntd-0001176-t004:** RNAi inhibitors.

		*C. elegans* orthologs								
Clade	Species	*eri-1*	*xrn-2*	*adr-2*	*xrn-1*	*adr-1*	*lin-15b*	*eri-5*	*eri-6/7*	*eri-3*
I/2	*Trichinella spiralis*	X	X		X					
III/8	*Ascaris suum*	X	X	X	X	X				
III/8	*Brugia malayi*	X	X		X	X				
IV/12	*Meloidogyne hapla*	X	X	X						
IV/12	*Meloidogyne incognita*	X	X							
V/9	*Caenorhabditis brenneri*	X	X	X	X	X	X	X	X	
V/9	*Caenorhabditis briggsae*	X	X	X	X	X	X	X	X	X
V/9	*Caenorhabditis remanei*	X	X	X	X	X	X	X	X	X
V/9	*Caenorhabditis japonica*	X	X	X	X	X	X	X	X	
V/9	*Ancylostoma caninum*	X	X	X		X				
V/9	*Haemonchus contortus*	X	X	X	X	X				
V/9	*Oesophagostomum dentatum*	X	X	X	X	X				
V/9	*Pristionchus pacificus*	X	X	X	X					

Species represented solely by expressed sequence tag (EST) datasets are not included, refer to Table S1. ‘X’ indicates presence of ortholog. See Dataset S4 for corresponding protein sequences.

**Table 5:** Nuclear RNAi effectors.

		*C. elegans* orthologs														
Clade	Species	*mut-7*	*cid-1*	*ekl-1*	*gfl-1*	*mes-2*	*ekl-4*	*mes-6*	*rha-1*	*ekl-6*	*zfp-1*	*mut-2*	*ekl-5*	*mes-3*	*mut-16*	*rde-2*
I/2	*Trichinella spiralis*	X			X	X			X							
III/8	*Ascaris suum*	X	X		X	X	X	X	X		X					
III/8	*Brugia malayi*	X	X	X	X	X	X	X	X	X	X					
IV/12	*Meloidogyne hapla*	X	X	X			X	X	X		X					
IV/12	*Meloidogyne incognita*		X	X	X	X	X		X							
V/9	*Ancylostoma caninum*	X	X	X	X	X		X		X						
V/9	*Caenorhabditis brenneri*	X	X	X	X	X	X	X	X	X	X	X	X	X	X	X
V/9	*Caenorhabditis briggsae*	X	X	X	X	X	X	X	X	X	X	X	X	X	X	X
V/9	*Caenorhabditis japonica*	X	X	X	X	X	X	X	X	X	X	X	X	X	X	X
V/9	*Caenorhabditis remanei*	X	X	X	X	X	X	X	X	X	X	X	X	X	X	X
V/9	*Haemonchus contortus*	X	X	X	X	X	X	X	X	X	X	X				
V/9	*Oesophagostomum dentatum*	X	X	X	X	X		X								
V/9	*Pristionchus pacificus*	X		X			X			X	X					

Species represented solely by expressed sequence tag (EST) datasets are not included, refer to Table S1 for these data. ‘X’ indicates presence of ortholog. See Dataset S5 for corresponding protein sequences.

### *C. elegans* displays an expanded repertoire of RNAi effectors relative to other nematodes

Perhaps the most striking observation is that each of the parasite species considered here possessed only a fraction of our original search set of 77 *C. elegans* RNAi proteins (Table 6), with all displaying a greatly contracted suite of RNAi effector proteins; of the original 77 *C. elegans* search strings, *H. contortus* returned 46, *A. suum* 44, *A. caninum* 40, *O. dentatum* 38, *P. pacificus* 36, *B. malayi* 35, *M. hapla* 28, *M. incognita* 27, and *T. spiralis* 22. This reduction in diversity (which could suggest either that: (i) orthologs of the *C. elegans* proteins are absent from the species in question; (ii) they have diverged to a degree that is unrecognisable on a primary sequence level, or (iii) our datasets possess significant areas of inadequate coverage such that additional RNAi effector genes await discovery in these species) was observed across all of the functional groupings in our dataset, but was most pronounced within the proteins responsible for uptake/spread of dsRNA. In contrast, the other Caenorhabditid species possessed an RNAi effector complement much closer to that of *C. elegans*; *C. briggsae* 65, *C. remanei 65*, *C. brenneri* 63, and *C. japonica* 60 (Table 6). However, both parasitic and free-living species returned only a subset of putative AGO orthologs relative to *C. elegans*. AGO analysis presented a significant challenge within our sequence similarity searches, due in part to significant areas of sequence similarity between functionally disparate *C. elegans* proteins. In many cases our BLAST analysis presented a clustering of multiple distinct AGOs around an individual *C. elegans* ortholog. Additionally, in some examples we could identify putative AGO orthologs which reciprocated to non-cleavage competent *C. elegans* proteins, but which encoded catalytic residues consistent with cleavage-competency themselves [32]. Clearly, using gross sequence similarity as an identification tool for AGOs underestimates functional diversity (data not shown), and as a result, we considered that an in depth analysis of AGO family diversity was beyond the scope of this study. This did not represent an issue for the analysis of other RNAi pathway protein families.

**Table 6:** Nematode RNAi effector protein complements.

Clade	Species	Number of RNAi effector proteins
V/9	*Caenorhabditis elegans*	77
V/9	*Caenorhabditis briggsae*	65
V/9	*Caenorhabditis remanei*	65
V/9	*Caenorhabditis brenneri*	63
V/9	*Caenorhabditis japonica*	60
V/9	*Haemonchus contortus*	46
III/8	*Ascaris suum*	44
V/9	*Ancylostoma caninum*	40
V/9	*Oesophagostomum dentatum*	38
V/9	*Pristionchus pacificus*	36
III/8	*Brugia malayi*	35
IV/12	*Meloidogyne hapla*	28
IV/12	*Meloidogyne incognita*	27
I/2	*Trichinella spiralis*	22

Total number of RNAi effector proteins identified for each species.

### Small RNA biosynthesis

Small RNA-based genetic regulatory pathways are ubiquitous in eukaryotes, and represent a set of proteins with conserved function and structure in evolutionarily distant organisms. As such, our analysis of proteins that perform nuclear biosynthesis, nuclear export and cytoplasmic processing of small RNAs such as miRNAs (Figure 1; for recent review, see [1]) should provide a positive control measure for both our approach, and sequence data quality. These core proteins were well conserved within our dataset (Table 1; Dataset S1) - transcripts encoding many of the proteins required for siRNA and miRNA processing, including RNase III enzymes (drosha, DRSH-1; pasha, PASH-1; dicer, DCR-1), RNA helicases (dicer-related helicases DRH-1 and -3), and exportins (XPO-1 and -3) are highly conserved across the genomic and transcriptomic datasets considered here, although orthologs of the dsRNA-binding protein and dicer-complex cofactor, RDE-4, were notably absent from all of the parasites except *B. malayi* and *A. caninum*.

### dsRNA uptake and spreading

Our dataset recognizes five *C. elegans* genes putatively responsible for dsRNA uptake and spread, identified from mutant screens for defects in systemic RNAi (the RNAi spreading defective mutants *rsd-2*, *-3* and *-6*, and the systemic RNAi defective mutants *sid-1* and *-2*). Much interest has centered on SIDs as core determinants of dsRNA uptake/spreading mechanisms. These transmembrane proteins were first described in *C. elegans* as mediators of systemic and environmental RNAi due to their role in transmembrane transport of dsRNA [21], [33]. Putative SID orthologs have since been described in disparate organisms including mammalian cells [34], trematode flatworms [35], crustaceans [36] and insects [37], [38] (although *Drosophila melanogaster* does not possess known SID orthologs, heterologous expression of *C. elegans* SID-1 sensitizes *Drosophila* cells to RNAi by soaking [39]). Similarly, expression of SID-1 in *C. elegans* neurons reverses the neuronal intractability of this species [40]. The role of SID-2 in environmental RNAi has been demonstrated by functional expression of *C. elegans* SID-2 in *C. briggsae*, a transformation which confers susceptibility to environmental RNAi in this species [21]. Given the importance of SID-1 and -2 to functional RNAi in *C. elegans*, it is surprising that these proteins are so poorly conserved in other nematodes, where putative SID-1 orthologs were identified in *H. contortus* and *O. dentatum* only (Table 2) and *sid-2* was not identified outside the *Caenorhabditis* genus. Similarly poor conservation was observed with RSD-2 (not identified) and RSD-6 (seen only in *P. pacificus*). RSD-3 is the sole perfectly conserved spreading protein in our dataset, occurring in all 13 species (see Table 2; Dataset S2). Evidence from *C. elegans* implicates RSD-3 in intercellular spread since *rsd-3* null mutants are able to take up dsRNA from the gut lumen, but are unable to distribute this dsRNA into the germline [41]. Despite lacking identifiable orthologs of SID-1, and -2, as well as RSD-2 and -6, plant-parasitic *Meloidogyne* and *Globodera* spp. display systemic RNAi following soaking in dsRNA/siRNA [7], [8], [42], [43], suggesting that alternative uptake proteins (e.g. *fed* mutants; see [44]), or mechanisms are involved, perhaps similar to the receptor-mediated endocytotic dsRNA uptake process seen in insect gut cells [45]. Intriguingly, our own unpublished data demonstrate a phenomenon of well conserved miRNA target transcript up-regulation in response to dsRNA/siRNA soaking of *M. incognita*, *G. pallida* and *A. suum*, possibly in response to a ubiquitous saturation of RNAi pathway effectors shared between exogenous (dsRNA/siRNA) and endogenous (miRNA) small RNA pathways, which could indicate that uptake is not limiting for these nematodes ([46]; unpublished observations). Additionally, we cannot discount the possibility that poorly-characterised morphological differences, such as cuticle permeability, better enable dsRNA uptake or propagation in PPNs relative to other parasite species.

### Secondary siRNA amplification

In *C. elegans*, plants [47], and *Neurospora* [48], the RNAi effect is greatly amplified by the action of RNA-dependent RNA polymerases (RdRPs), which produce a population of secondary siRNAs from the target mRNA template [41], [49]–[52]. Further examples of RdRP-catalyzed amplification mechanisms have recently been reported in *Paramecium tetraurelia*, where multiple RdRPs appear to exist [53], and in *Drosophila*, where a non-canonical RdRP has been identified [54]. The most well-conserved RdRP in our dataset is EGO-1 (*Enhancer of Glp-One* [*glp-1*]), which appears in seven species (Table 2). RRF-3 (RNA-dependent RNA polymerase family member 3), which coordinates complex and ill-understood interactions between RNAi inhibition and amplification of the secondary siRNA response is reasonably well conserved, with RRF-1 less so. EGO-1 is an RdRP with core functions in transcription of “WAGO” (worm-specific AGO [55])-interacting 22G-RNAs responsible for silencing events involved in genome surveillance [56], [57] and with additional roles in germline development [58], heterochromatin assembly [59], [60], holocentric chromosome segregation [61], and P-granule function [62]. In light of these core roles, the inter-species conservation of EGO-1 is unsurprising. RRF-3, which is also reasonably well-conserved, was traditionally referred to as an inhibitory RdRP [63], although through recent work has been implicated in the production of secondary 26G-RNAs which seed a two-step process of secondary amplification against endogenous targets (endo-siRNAs) [57], [64], [65]. It is also believed that nonsense-mediated decay (NMD) proteins SMG-2 (Suppressor with Morphological effects on Genitalia 2), -5 and -6 may play a role in the induction and maintenance of secondary amplification [66], a hypothesis supported by analysis of *smg* null mutants which are defective for RNAi initiation [67]. SMG-2 and -6 are perfectly conserved across the genomes and transcriptomes considered here, while SMG-5 is not well conserved (see Table 2; Dataset S2). Conservation of EGO-1 suggests that all of the nematode species examined here are capable of some degree of secondary RNAi amplification, consistent with previous observations of the potency of RNAi in PPNs, where soaking in as little as 0.1 µg/ml dsRNA was capable of eliciting significant and consistent knockdown of transcripts in *Globodera pallida* and *M. incognita* second stage juveniles (J2s) [8].

### AGOs and RISC

*C. elegans* possesses at least 27 distinct AGOs (including pseudogenes C06A1.4 and C14B1.7) [32], which constitute the central effectors of the RNA-induced silencing complex (RISC), conferring both function and specificity to RISC. All of the nematodes in our dataset possessed multiple distinct AGOs (Table 3). A subset of well-conserved AGOs (defined according to closest *C. elegans* BLAST match) included the miRNA-interacting AGO, ALG-1 (Argonaute [Plant]-Like Gene), as well as several endo-siRNA-interacting AGOs including the 26G-RNA-interacting ALG-4 [68], and the 22G-RNA-interacting WAGOs, R06C7.1 and F58G1.1 [55]. Some members of the PIWI-clade of AGOs, such as PRG-1 (Piwi-Related Gene 1), PRG-2, ERGO-1 (Endogenous Rnai deficient arGOnaute 1) and the AGO/PIWI-clade secondary AGOs SAGO-1 and SAGO-2, are not well conserved. Surprisingly, RDE-1, which is believed to be the main AGO involved in silencing events triggered by exogenous dsRNA in *C. elegans*, was only identified in the animal parasitic nematodes *A. suum*, *H. contortus* and *A. caninum*. Thus the AGOs known in *C. elegans* to be responsible for endogenous regulation of gene expression are well conserved, while the AGOs responsible for executing RNAi triggered by exogenous dsRNA are not. However, as previously stated, our identification strategy does not account for the possibility that other uncharacterized AGOs exist in each nematode species, performing roles comparable to those AGOs which we could not identify. A further four *C. elegans* AGOs (M03D4.7; T23D8.7; ZK218.8, NRDE-3) did not appear to be present within our parasite dataset. The AGO NRDE-3, is responsible for nuclear translocation of RNAi triggers in *C.* elegans, and is involved in processes which lead to heritability of gene silencing events. As NRDE-3 is completely absent from the parasite datasets considered here, this may indicate that silencing events cannot be passed between generations of parasitic nematodes. Our data suggest that most nematodes have smaller AGO complements than *C. elegans*, although the impact this has on functional diversity is unknown. The contracted complement of AGOs identified in the parasite species relative to C. elegans is consistent with their propensity for gene loss [69]. This could indicate redundancy in the function of individual AGOs within *C. elegans*, or conversely a reduced functionality within the parasites considered here. Interestingly, ERGO-1 is involved in the function of endogenous siRNA populations within *C. elegans* [57], [65] but is poorly conserved perhaps indicating a differential small RNA population dynamic between species. Again, the poor conservation of such proteins in RNAi-competent plant-parasitic species would seem to suggest that such deficiencies need not undermine RNAi functionality.

In addition to the catalytic AGO protein, RISCs also comprise several protein co-factors, including multiple dsRNA-binding proteins and exonucleases which are thought to pass from elements of the biosynthetic machinery (Figure 1), although these co-factors are in fact quite poorly characterized, even in *C. elegans*. Our analysis reveals that TSN-1 (Tudor Staphylococcal Nuclease 1), which is a common component of RISC in *C. elegans*, *Drosophila* and mammalian cells [70], is well conserved across the species considered here (Table 3; Dataset S3). The ALG interacting protein AIN-1, responsible for targeting miRNA-bound ALGs to P-bodies [71], [72], is also reasonably well-conserved, being present in seven species. VIG-1, the *C. elegans* ortholog of *Drosophila* VASA intronic gene which regulates transition between larval and adult cellular fates though interaction with the *let-7* miRNA [73], was identified in five of our eight species.

### RNAi inhibitors

Proteins with RNAi-inhibiting function were first characterized in *C. elegans*, leading to the identification of RNAi-hypersensitive null mutant strains of RRF-3 [63] and ERI-1 [74]. Only two RNAi inhibitor orthologs, the DEDDh-like 3′-5′ siRNA exonuclease ERI-1 and the miRNA 5′-3′ exonuclease XRN-2 (XRN RiboNuclease related 2), are fully conserved across our genomic and transcriptomic datasets (Table 4; Dataset S4). Sporadically-conserved inhibitors included the adenosine deaminases ADR-1 and -2 [75], and LIN-15b, while orthologs of ERI-3, -5 and -6/7 [76] were not identified outside *Caenorhabditis* spp.

### Nuclear effectors

The RNAi pathway affects a number of poorly understood nuclear silencing mechanisms. We found that an uncharacterized nuclear effector, EKL-1 (Enhancer of KSR-1 Lethality 1 [KSR-1 is a Ras-ERK signaling scaffold protein] [77]) was the most highly conserved between species (Table 5; Dataset S5). Other chromatin-associated proteins, helicases and methylation factors are conserved to varying degrees, however MES-3 (Maternal Effect Sterile 3), RDE-2 (RNAi Defective 2), EKL-5 and MUT-16 were only found in *Caenorhabditis* spp.

### Conclusions

In spite of the contrasting experimental evidence from published studies, our data indicate that diverse nematode species possess the machinery required to facilitate an RNAi response. Our inability to culture many animal parasitic nematodes under *in vitro* conditions may represent one of the main reasons why RNAi is difficult to perform in these species. Certainly, where RNAi has been most successful in nematodes it has been in species/life-stages amenable to laboratory culture, e.g. free living species such as *C. elegans* or free-living stages of parasites such as PPN J2 larvae, and more recently *in vivo* in mosquito-stage *Brugia* [78], although some readily-cultured species seem refractory to RNAi [12]. Additionally, given that small non-coding RNAs are heavily involved in various cellular stress responses [79], it may be that adverse culture conditions lead to their increased expression, resulting in saturation of available RISC proteins, which would interfere with the organism's ability to direct an RNAi response to an exogenous trigger. If such saturation events varied between cells and/or tissues, then this could account for differing knockdown susceptibilities between some genes. Further, we have little information on differences in RNAi effector protein expression level or localization between species and/or life-stages, which might account for the observed variability. Other possible explanations for RNAi disparities include factors for which we have limited information, such as uncharacterized morphological differences between species (e.g. permeability of the cuticle to nucleic acids), or allelic diversity in discrete worm populations which may affect RNAi susceptibility in a similar fashion to drug susceptibility/resistance. In summary, our data do not support inter-species disparities in RNAi effector protein complements as an explanation for differences in RNAi competencies. Whilst the Caenorhabditid spp. encode significantly more RNAi pathway effectors than the other nematodes considered here, qualitative similarities in functional groupings across species with variable RNAi susceptibilities validate our conclusion.

## Supporting Information

Dataset S1 **Nematode proteins associated with biosynthesis and nuclear export of small RNA; domains and sequence data.** (*, putative stop codon)
(DOC)

Dataset S2 **Nematode proteins putatively responsible for secondary amplification, uptake and intercellular spread of siRNA; domains and sequence data.** (*, putative stop codon)
(DOC)

Dataset S3 **Components of nematode RNA-induced silencing complex (RISC); domains and sequence data.** (*, putative stop codon)
(DOC)

Dataset S4 **Nematode RNA interference (RNAi) inhibitor proteins; domains and sequence data.** (*, putative stop codon)
(DOC)

Dataset S5 **Nematode chromatin modifiers, histone methylation factors, and other nuclear effectors; domains and sequence data.** (*, putative stop codon)
(DOC)

Table S1 **Distribution of RNAi pathway components in nematode expressed-sequence tag, genome and transcriptome databases.** Select tab at page bottom to view RISC-associated proteins, or all other proteins. Key to colours described below. Species were assigned to Clades according to nematode molecular phylogeny as described by [25], [26].
(XLS)
