# Supplementary material for: RNAi Effector Diversity in Nematodes
Source: PLoS Negl Trop Dis. 2011 Jun 7;5(6):e1176. doi: 10.1371/journal.pntd.0001176 (PMC3110158; doi:10.1371/journal.pntd.0001176)
Supplement: Dataset S1 — Nematode proteins associated with biosynthesis and nuclear export of small RNA; domains and sequence data. (*, putative stop codon) (DOC) [file pntd.0001176.s001.doc]

**Dataset S1**

**Domain analysis**

The microprocessor RNase Drosha (DRSH-1) is characterised by dual catalytic RNase III domains and a carboxy-terminal dsRNA-binding domain. The cytosolic RNase Dicer (DCR-1) contains an amino-terminal DEXD-box helicase, followed by a PAZ domain, dual catalytic RNase III domains and a carboxy-terminal dsRNA-binding domain. The Drosha cofactor Pasha contains a tryptophan-rich (WW) domain, and a dsRNA-binding domain. Dicer-related helicase 3 (Drh-3) contains a DEAH/D-box helicase and a carboxy-terminal RIG-I-like regulatory domain. All three exportin (XPO) proteins are characterised by an amino-terminal importin-beta like/XPO superfamily domain and a carboxy-terminal armadillo-like XPO helicase domain. Finally, RDE-4 is a dsRNA-binding protein characterised by dual dsRNA-binding domains. In all cases, identified nematode orthologs were required to match the domain topologies outlined above. Minimally, in cases of short sequences, we required at least one matched domain in the top BLAST return, for that protein to be considered a putative ortholog.

**Shared protein domains**

Dicer possesses a PAZ domain which is also common to the Argonaute (AGO) protein family, however none of the putative dicer orthologs were identified here on the basis of PAZ domain similarity only. Dicer and drosha share dual catalytic RNase domains, and thus, delineation of a putative drosha ortholog from a potential truncated transcriptomic dicer sequence relied solely on BLAST identification. Many of the above proteins contain dsRNA-binding domains, however no putative protein ortholog was identified on the basis of one dsRNA-binding domain only, except *B. malayi* RDE-4, in which we could only identify a single conserved dsRNA-binding domain.

**DRH-3**

***Ancylostoma caninum* DRH-3**

MSSLAEQERYKPIVEALENELKEQFKKDANSRAIVFVTRRSTAVELMNYLNTEKVLGRHDLVGFVTSTSKKSAEYGQTKEEQQKALDDFNRGKRKVIIATSVVEEGLDVSTCNLIIKYNSSSNAVQRVQRRGRARARDSRSVLIVLSENVAQTEYQAIMAEKIMNKCLKRIQEQGEKALEKRVKEVMERQAKERRIVVEHRMKAREALMDKLFTIRCKIDSRDVCESTYVRTINGSSYVCVDPGIWRRHKIETKAISTKMKFVDEVTQILAEVRCECGETLGNVMKYAGTYLPTLNIHNLMFYERIEGKETAGEAISGWAKANERFWIPEANEQELREMLRSLAAEDKENKILLDVMCDKVIAVQEKLLEKERKKKEETMRIKTDWE

***Ascaris suum* DRH-3**

MKLVRVALGFGAGSGAIELRPYQEELVEAACRGVNTIICAPTGSGKTIVATYIIRNHLQERKMLGRPQGYVILI

***Brugia malayi* DRH-3**

MASDSGFRLGHLRLYKAELLGSVDNIEKLQQFRQFFGDSRSFTRLSELFKDDSNKNVALNYFWHQICFNREAMDRILSMATEGNKALLDSIHESMSAKSLKYYHKMFYEDAKKMDKIIMTIEPLPLCAGLKDASYGSIKNVVQEMMRSKKYEEAACYLMRNLPKVKSLENXSSDWYFDFLNACLIDSANRSIPEFIDPDYKIQLGDEKLERLDDAESIELRPYQQELVETACRGMNTIICAPTGSGKTVVAAHIILEHFRAMKAADKPSRVAMLVPTIPLVEQQCIMLNRYLRKTFWVDGMSGSEPVDENGRAPNVLASHVTVFTPQIFINLLRNIRRDDLLMRMLHRFDGPKPQIVGLTASLPLGAGRASVDAALDIKRAHRPEYDSFSQSLEICMRKIELAIKPELGRIAENKILNFKVEETVFPAHRNSTRYESFVGGLKNRLIELPDDKLKHQLIKMLEHLGYYYRALCLSDLLPNWYAYSYLCENMTKEIVQDAGDRTTIQNQLAKLFEKFVKSDELNFKENDSGEEKEILKMLHTILLEQYVSDPASRTIIFVTTRKLAQYLSHHLNAVKIVDRTSRAVGFITSSNRSSALNGQTTEEQRTVIENFNQGTLKVLVATSVAEEGLDISACNLIIKYNNTGSERSLIQRRGRARAKHSKSILLALDGSIEEKELENIQKEHLMRRCLEHIQTKSENQMKHLVEAKIKQMKALQEANLVRESEKKRSLEGKCYDLKCRLCGSFICKSSSMRIACDNHYVCCDPTIWERIDARVHNAKSLAIATLVGKLHCKGTDESDCSEVLGTIVKLYGAFLPTIAAKAIMIDDKEGLSGRPQYEKKWDSITTDKFCVEPITEFDLKVMLNSLHXYSREQHLQFEAEAGLAVKRALTEMKKEKRQFVIEE

***Caenorhabditis brenneri* DRH-3**

MHEPSCYEEEDISRQSLPFGSPYFPAYFRLLKWKILDVCVSTMKFKDIGDFALFEDCFPPGKLEEVVKKITGEAEPFTQDPEFKKLKETEVDPKFKRHAETYLKLRHDHKTKVERRRFAEYFLNKILFNEQGLRIADKLIWQHSRQVYGTAHWKDLPKDWMTTDTYKKKFYDEEELERDPYGMQRLDRVAGSARGMLIMKHLESDHLVRKEYDVLKKEVSDPENLCQTELVRNLLDEGPLWELKMEEGEEIIRKADKWNVHKTLVIFFYITGFRAKSEKEQEEWFYCLMDAMKNDPTNRAALHFLNKNYAEELDERQKERNRRRLQEFKGLKVDEKGVGNTIVTNIRPQKNETAYNPDADETNLVLRVYQEELVQPALEGKNCVIVGPTGCGKTEVAIYAALNHLRERQAAGQTGRVAMLVPKIPLVTQQKERFLQYCRGSFTVNGFFGSEKSDSGEGRKDDVIESHIVVMTPQILINMLQSVRVNERLYVADFSMMIFDEVHKATGNHPYVIINQIVQEWEHDKPQIIGLTASLNVSSTAQKDLSQMLFSIHTMLALLNAPYLSTIKLQASIDELNKHVSKPDDSVEVCQPTATALRNYIETYLGTAHIKLCHELESLSKLRHNCFPSNSYRRFKKLSPKFYEIYESNLQNINQDLKKLNTAEKTIAVTWMKYIRLYVEARGMVDLMPARLVFEFMEARIRELDTGHKLGDQFSDFFKKYSGLEANAKSDEPMIVTKLKDTLITQFKVTPDSRVIIFVTQRATAQRISDFLNKSHIMDQFRDPDDDGYDEMVGFVLGTNNQGGAVQQSQQEQQKVLSRFNNGKMKVIVATSVVEEGLDVTSCNLIIKYNCSSGSAIQLVQRRGRARAKNSRSVLLAVNTKVNEDENNALISEKFMRMCVKKIEEAGPKQLEIDVAAAAKKIAIERKNELTAQLELRERFANSLYKLNCVGCSTTFCMSSDIKKVYSNYMAFDPAAWQFFTVESKKKKPNSYLSEDTQPLSILKCAKCVTTVIGKAYKMRGVYLPQIDVKSVFFVEENSSESKTAKKWSSVEQELFYVGEATQDNFEDMLNALSNNECNMDKKRLLDLDSKQHIRDIELKKYRIRNEEQEKAKAERMKKAEQEGVEFEEGEEPEPEREGATAFSEDES

***Caenorhabditis briggsae* DRH-3**

MKHDPTNRAALYFLNKNYAVELEEREKERDRQRLQILKGQQINEEAVGEQIFKEIRAQPSESGYSPSIEIAELKLRTYQEELVQPALEGKNCVVVAPTGAGKTEVAIYAALNHIKERHNQEKAARVVLLVPKIPLVSQQKERFLKYCAGQYHVCGIHGSEKSDTGEGRRDDVLQAHIVVMTPQILINMLQSVRRNERLYVSDFSMMVFDEVHKTSGNHPYVLINRLVQEWDYEKPQIIGLTASLNVNAKVHTETSAMLGNIYTMLALLNTPCLSSITHQASIDELNEHVTKPDDEVEVLPPGPNVLRAHIETYLHINHGKLCGELEKLSKSRHNCFPAKTYHMFRKANTKDYEYYDALLTNIIQDLNKLNTPEKMCAQKWTKYIKVYVEARSIVDVMPAMTAFEYMRDAIRILDNEHTLTQFSDFFTDRIYEPLRKNSENVEPPIVQKLKDTLITQFATIPDSRVIIFVVQRSTAQRVSDFLNRSEEVMKQLSSSGDKREDMIGYVLGTNKQGAVQQSPDEQKTVLGRFNTGKLKVIVATSVVEEGLDVAACNLIIKYNCSSGSAIQLIQQRGRARAKNSRSVLLAVHSKVKEDDNNALISEKFMRLCMKHIEEKGSKQLESEVSIAAARIQKERKNEFEAQQALRNKLDSKIYRLTCTSCSSFFSRSTAVKKVYSNYMIFDNDVWSRFGIESRRKKPNRYLNEETQALSVLKCLSCKSDVGKAYKMRGVYLPQLDVKAVTFAPENHDEGTTTRQKWSAVENDLFWIGEATQNDFEIMLNALLNTQENMDKKRILDLDSKQHIRDIELKKYRDRETENAARKKKVAEEEERERPVQGKTDFSEDED

***Caenorhabditis japonica* DRH-3**

MDNKNKTDGKHSFKRATVEFLESQILDVDGFIDRYTFFEWINIPLVIRKEICRQRAIKKDICVQSVTNSNEYGEFLRFEKLFKPGEIKTLVDKILRPVPVDNPFKQKIARAALDEKTKKQAEAYAEFRVQHSTRIEKRRYIEFFLNKVLFTETGLKIADELVFYYHKDLYGMVHWESMPPKWASSETFINRFYNEAELSNRCVSQKLDKVAGSARGNLILPHFKTNSRTEAEYGVIMDVLHRPEFTIATPFVKDALEEGPVYMIEIKDGEEVKVPAERWTEHHTRLLFVSIPGFKENERRERGEHEDWYFYLLEAMQNDPYNRAALWFLDKNYEAELEQKENDRQRASLAMMKGLNAEESGLENEVVTEIRPQPNDFGYQPDVETTELKLRSYQEELVQPALEGRNCVIVAPTGSGKTEVAIYAALHHIGEMISNRKAARVVLVVPTIALVAQQKERVLKYCRGKYHVDGFHGSEESKTGNGRRDDVLNSHVAVMTPQILVNMLQSVRQTERLYISDFSMIVLDEVHRASGNHPYVEISRLVQDWAHAKPQIIGLTASLNINATAHVDISRMLESIYEMLAILNTPYLSTITKQENIDELNRHVGKPDDSVEVCDPPSDVDAPLRGFIKRILHTYHTKLCNELDSLVKNRHNAFPTHYLKNFRNAKTDKFELYESFLQSVCQDLNKLNTADKGLAQLYTKYIRVYVEARGIVEVMPPTVALEYMKEALDKLNLNHTLSQFSDFSQNYEALTKNIDHKDPVIVIKLKNTIIEQFRVAPDSRVIIFVTQRSTAQRVCDFLNKSGIMDQFKMRNDGDSVGYVLGVNNSGAVQQSSQEQERVLDRFNNGKLKVLVATSVIEEGLDVTACNLIIKYNCSSGSAIQLIQQRGRARAKNSRSVLLAVSSAVNMKENNALISEKYMKACLTMIVKNTQKQLEIEVEKIKKRLEAERRRELEEQASMRLRHADKSYKVICASCNKHFCKSTAIKKIYSNYMVMEPEIWDHLDLVSRARKVNKMHAADCVSLCNIKCKHCKADIGRAYKIRGVYLPQLAVKELTFVPVHGNRMDTETKGKWKDVEEQLFFVPEAREADFKVMLNALVTSESNMEKKRLLDFDSKQHVKAIEIELVLEN

***Caenorhabditis remanei* DRH-3**

MSLLIHVSTTIRRILAYFVLFNVGLGLLGLPWFSIYIGLSGLCLCFFTCFHVYKKNDRMMFPFYLYVLFTIFYLLFLGGYFFFVNIFHKEMVKGRDFSHLQVTTLILFLTIPGFDMKHRKPEDRDEWYYGLLDAMRHDAANRSALHFLNSKYSEELEERDKERDRLRLQVLKGQQVNEEAVGEQIFQTIRPQPKDNGFIPSLEAGELVLRTYQEELVQTALEGKNCVIIAPTGSGKTEVAIYAAMKHISRREAFGEHSRVVLVVPKIPLVTQQKERFLKYCNGKYVVNGFHGSEKSDSGEGRRDDVLASHIVVMTPQILINMLQSVRRNERLYVSDFSMMIFDEVHYTTGNHAYVNLNRIVQEWEYDKPQIIGLTASLNVNASQQTDINSMLNGIYSMLALLNAPHLSTITHQSSIDELNKYVSKPDDTIEVVQPGENVLRSHIDNYLNTKHYKLVAELEKLSKSRHNCFPAGSFRSFKNAKPKEFMLYESLVQSLIQDLNKLNTPDKMVAQKWTKYIRVYIEARGIVDVMPAMVAFNFMEESIRQLNSEHTLDQFSDFLTDKVYDPLKQRSEGVEPEIVKKLKTTLVNQFKEQPDSRVIIFVTQRNTAQRVSEFLNESGILEQFLNTTTRQKTVGYVLGTNNTGSVQQSPQEQQRVLEQFNSGKLKVIVATSVVEEGLDVTSCNLIIKYNCSSASAIQLVQRRGRARAKNSRSVLLAVHSRVQNDESNAMLSEKFMRQCVKIIEQNGNKMLEKEVHKATVDIQRQRAAEAAELKQQMERNGRNIFTVNCAQCQWTFCKSTDIKKVSSNYMAFHPMVWDNVSVESQKKAPSYRNEDTQPLSILKCHKCQHLVGKAYKMRGVYLPQFAVKNVNFVAQNNDNGTATKAHWSGVQDELFFIGEATQHDFEIMLNALGNTEANMDKKRILDLDSKQLMKLVESKRFLAMKEKKEQEARMKRAEDEENGVVPTNGSTEFSEDED

***Haemonchus contortus* DRH-3 (Likely exon-intron boundary issue with predicted protein)**

NMLKSIRQDERLYVCDFSLLIFDEVHHCTKDHPYNILMQTIHDYQGPKPQVEAALRGKNTIACAPTGSGKTEVAIHVATSHLDERAENHQPARVFCSRYYVEGFHGSGLKGASRRDIVLACDIVVMTPQILL*VYQLYRSGASVYKVAMLVPRTPLVDQQKHRFHKYVRGLSLSRGVRNGSIPKNMVSSYIRVSVRVVTETTVRAYEVY

***Meloidogyne hapla* DRH-3**

MFFNSNKYNYNLAASISIFCSVVVIFSCGFVGIILINELNIIYEEIIADLEISLVTNKETLNNILDLQNKYSKSTQWVKVFIPLARRRSVRDLYLADEVDKCNCHSRNEVCPRGPPGAPGARGEQGEPGEAGVPGLPGAAGVMALLDGMEAGCIRCPTGPPGPIGLPGPAGKAGVPGQPGVNGHPGRDGHPGIQGPHGDLGPIGERGKEGEPGQPGRDGFTSKGEPGPKGENGKAGLPGRPGEKGQNGLPGLCGPPGPIGNAGFPGTPGVPGLQGENGANGVHGEDAKYCSCPSTAKFPEIIGHLPNEAEISKLNRWINELRRKGITVESHIRLFVYEFVENVDNDLDELVKRFANNKIDELNNLYEMALERSLALGKDSIEAKCDALRATKANLFKIISNSNIPDSLPILLSIIARCKNVDPISYSELHLNDLSTIDYYIRLFAGNDHFKVDRIRCVLEPKHIFRWFSSKNINEYDHLLERVERIYSLNDPTKREIEAAACLFIRGLVHCKDIPDSTKNFGDWYFEFLEALLSHGDCRFFARLIDPDYLDDLNKWKANKLIGAGLPNSAELFADLVGDEEEPTMPRDQKDYLKYKTCSNVLEYEPESYDLRPYQNELVLHANKGRNTIICAPTGSGKTLVAVDIIKNHLTNRHRAGKVGRCVMLVPTVPLVDQQSLHFVQFLTEYRDSYRPNIAYWVDGFSGCENILEGRAYRLLSADILVMTPQILINMLESILRSERVYFSDFTLMIFDECHHATKLHPYKILMEMLEKSKLKEKPQIVGLTASMGVGDTSLDIKACCEHMLNLCSNLHSETISTVRHQLDNLKSHVMPPVDYVKRVRRPAEDPFLDYIERAMYKIENDMKPHLTKLAELCKLKKEEIEFPQHSNISRYQTIVGNLKKCAQRVQESEMRFLLVRSIDHLSHYFHSILINDLLPSSYAFQYLHEKMSDYKQITGGSSPIDLINQRLLSYYQDLHPKLFDCVKNEKLQNKEILKELHSILRRQFESDPNSRCLIFVATR

NSASKLADHLKRVPELPIFYKKENVGYMVSSNQSLSAGGQSTQEQQIMIRDFDSGKVKVLVVTSVAEEGVNIAACNFIIKYNNVGSERSMIQRRGRARQKNSLSILLALDTGVEQAEYLNMQKEAMMMRCLLDLQEKSETNLKNQINAKREERRKIEERKLKVLEAKLSRLNNRRYKLSCRSCNNLICKSTHVRSIAYSTFVVCDPTVWKRSKIDIREKPTKDHLFTKCAKWLCGQCGNQEWGVIVKYSNCYLPQLSATLFSLEREDQHDEFDELRGGDNRGRTWNNIQDDYFNIEPINMRNIIDMFSALTNSYSNLTKQMDQQECIANIKFIEKMKEKKTDKKNKIQIFLEE

***Meloidogyne incognita* DRH-3**

MSRFGVGDTSLDITACYQHMLNLCSNLHSETISTVRHQLDNLKSHVMPPVDVVTRVKRPANDPFLDYVERVMYKIENEMKPHLPKLAEMCKLKKEEIEFPLHSNNSRYQTVVGTLKKSAQRVQDSEMRFLLVRSIDHLSHYFHSILINDLLPSSFAFHYLQEKMSDYKQNSGGSSHIDVINQRLLGYYQDLQKKLYECVKNEKLQNKEILKELHLILKKQFESDPNSRCLIFVATRNCASKLADHLKKVPELPIFYNKENVGYMVSSNQSLSAGGQSTQEQQQMIRDFDCGKVKVLVVTSVAEEGVNIAACNLIIKYNNVGSERSMIQRRGRARQKNSLS IAPINMRNIVDMFSALTNSFSSLTKQMDQQECIANIKFIEKMEEKKTDKKNKIQIFLEE

***Oesophagostomum dentatum* DRH-3**

HPYNILMQAVHDYQGPKPQTMGLTASLGIGMATTDESGMESIYELMANIGATSLASVKRHIDILNXYVPKPVDMTKKVDRLPWENSPFLRGLVNIMKRIQDDVEPQLKKLTQDNQTGVKLTKEEVKFEDPLATEKYIQKVNTLSTTLGKVYSGDFKFEPSIALEYLGVLSQAISINDLMPAKYALEYMQRNLRDLSTKFEVECSRKFYSYFADNIVPLTNHAEQERFKPIVEALETELKDQFKKDANSRAIVFVTRRSTAVELMNYLNREKVLGRHDLVGFVTSTSKKNAEYGQTKEEQQRALDDFNRGKRKVIIATSVVEEGLDVSTCNLIIKYNSSSSAVQRVQRRGRARARDSRSVLIVLSENVAQTEYQAIMAEKIMNKCVKRIQEQGDRAVEKKVREVMDRQAKERRIVIEHKMRAQEALKDALFTLCCKIDSKPVCMSTDVRTINGTSYVCVDPEIWSRLRIKTKEISTKMKFVDEVTQILAEVHCLCGQTIGTVMKYAGTYLPTLNISSLMFKVGEVGGADTKNGETINSWSKANDRFWIPEANEQELRQMLVSLAAQDSENKIVLDVMCDKMIAVQEKLLEKERKKKEATVRIKTDWD

***Pristionchus pacificus* DRH-3**

MSRNADSFGFWPLIAALAQDSHNITTTLQYLLPNWQTELAAHRSSLAASSFSFESMARVIMAGVSCRPPAEFEPFRDEMTVPGTSIMPTLRAYQKELVERTDRGENTIVCAPTGSGKTVVGAHVALHHLRTRAKEGKPARVVMIVPKVFLVEQQAAQFNSYAKKEFYVAKLSGESSETGEPQLIKFLSGDIVVLTPQILVNMLQQESEAARLYIADVSLLLLDECHHTDKKNPYNVIMQAVKEATHARPQVVGLTASLGIGDDAGITVEDHIVRMCVRMAADSITTVMRNRDELARFVQLPEDVIRRVMPMLPNERRFHAHLITAISFVSRQLYVEFDALLRERPASIDPNKLTRFPPLEKTEDFTQAVVNVDTELRNNELFNRKLTDGERKWRLKRGIDFVQMYHKTMLLNDLLPAAYAFEQLSADVDELDTLAGRQCAFLDYFRKEQAEFAQRTSTENDKEILQQLRKELAGQFRADSASRVLIFCLRRETAQLLTKYLNEQGIEAMGRAEVLTSTNAASVRNGQSPSEQRAVIEAFTRGACKNPTLDLWMTVRKYCENIEKNL

***Trichinella spiralis* DRH-3 (Likely exon-intron boundary issue with predicted protein)**

PTRPLVRRNLLK*FAQSWC*Y*NSCKLKV*FTRNMDVVSLIESLNEVATHKKTLSLYDFQEDLVAPALLGKNTLICSPTGTGKTYMLLKVILNHIKHQKLTHKKYKICLIVPTVVLAEQHLNLFEEYISDVHYLGNSESGSSTFISARDNELLICTAQTLLNVLNYSIERKFQLEYQLVHVCCC

**DRSH-1**

***Ancylostoma caninum* DRSH-1**

SNSRLLPMSEVIRYLXSYKPLLTEEEVEQLEHSADADFVKAVLPKKFQLATNPRKRPSTIRVDNIKRLPSGSGFGIEHKTSPPIAYANLKNPELVEAQRRLGKLRRIQSSASSGQREQIEDINILLKRISELKQQRATALNATRIIPCNGFFATGLYADITAHALLLILAVKHARFHWSLLEFEKIIGYSFANRTLIELAFTHPSYKNDFGTNVDHIKTTLTNCSFRRYAPFTENNEKKKGFRNLMHIMAQSGSSSAGLSKVAHNERLEYLGDAVVELVVSSRLFFILPHQEEGGLATYRSALVQNRNLAALGKKLRLGDWMMYAHGIDLCDEEDFRKSLANTFEAVLAAIYLDGGIEECDRIFADAMFGDDPTVRSQWLNVPEHPLKIEQPDGDRMLIESTDELTELTDLENTLASNFKTSDFLRRRNTNQA

***Ascaris suum* DRSH-1**

MLYAHGPDLCHESDLRHAMANAFEAMMAAIYLDAGIDECDRIFGNAMFGGNEELLGAWFELEEHPLKRDNPYGDRHLIKEVPALQLLTEFEKSIGVTFKHIRVLAKAFTRRNVGFNNLTLGHNQRLEFLGDTVLQLITTDYLYKHFPMHHEGHLSILRTCLVSNKTQSVICDDVGMTKYLVMPKAMQKNGAPVLRVKDKA

DLVECTDNFDSRL

***Brugia malayi* DRSH-1**

MFSNPGLNLPRINLPQQAIRPVLAPDVSRFGCSSSSGTVNMLSTTGGKAARNVPPLIVSHNTSNLSAMRPLLPVSNSSEQISDVQCRPYRNVVGCPKPLFSDIERYQWSSASTVERSQFVRTPGSRNSRSLPSLRDVSRVAVKQNLHIREAGVSDDTVCTLTSDCGISAAEPIDISDEDFNYVGNEEEHAEHKSNKWNTTKTRYRKRRHEQSSSGESESEQGNFYVLVSFLTKGKDIPRWQKLTKEDFVDTILSDTDDEIDQVEQTVAMSGFHSRSIPEEKFYCINDVGDCCATEELKKLHKRFRIEIIEKIAKEKESQPKSDPPNILAFHDNCKCKWISLLDFSKETSVLLGGHHSDTETESESCDSDDCVDDSDERSGNVSVDEQQTDSKDKITRVKMSESKKHSTVAKQEVIRKRNHPAILHSDVCFNEIGQMNDGPECRCSWAAKKSGLRHNKYAGETVIEKCDLSSSNSHRLWHYILYTEPNPNTVVRRSTKINHRGRTYELEGFSVFFHKSLPSNFPQTPLSKWTSEYNIKFMEENIPTNFTIEDLELFHHYLFTDIMEMYDLKKYPLDNTDGCPYYHCMPRFVYNLSDGGKEILPMSVVMTYLIESYHCLMTESEAQILKHDADAFRDFAENVRGSLVMNASKRPSTIRVDLIDRPDFLPNNSDTLFPLITHFGXRPSSHTYNSNAEYQKMSKEYLRMRKILAMKPRVSAEERGKLAQKASQLKALRNDSQLKRDFVMSVSSRSFYSTGLFPDIVQHGLLLILACAHVRFQWSLQVYEQERIHYVFKNRSLLELALTHPSYRTNYGTNSDHARNTLNNCGVRSSKQRVHDRLVQQQLSAKKRGFHTLMEIMSKLGSKKAEQSPLNHNERLEFLGDAVIEFITTIHLFYMFSELDEGGLATYRSTMVQNKNLALLAKKIGLDEFMLYAHGPDLCHESDLRHAMANAFEAMMAAIYLDAGIDECDRIFGHAMFADSKELLSTWFDLEDHPLKRDNPNGDRHLINGVPALQXLTELEKNIGITFKHIRVLAKAFTRRNIGFNNLTLGHNQRLEFLGDTVLQLITTDYLYKHFPYHHEGHLSILRTCLVSNRTQSVICDDIGMTKYLVTPKVMQKSGMPVLRVKDKADLVEAFLGALYVDRGFDYCKVFCRICFFPRLKFFIIAQHWNDPKSQLQQCCLTLRQMDGGEPDIPEYRTIAVEGPTNTRIYKVAVYFRKKRLAVGCGHTMQLAQMRAAENALIKRSDLFPTLKNTAKHSGVQNRRENGLGAKVKCELADRTKQRMLFDSTHVLQSDFATGSSLTDGTQTSRPQIISLLDLKFDADGCLI

***Caenorhabditis brenneri* DRSH-1**

MVPFKPSDRNRRTNQDGAEGSYRYELRKCNQKSLDEECILDDYERNDELNAESSTAFYNHPDAKENSDDTSEYGPSDDETLANYPLRKPSYCVKAAIGDLFSRNPHPSVSEMNELTEFYSVKYRTIFETMELKRNSQKIFVIAETIVNVLAITFDNNLLSRTHLNKNPEKDAGIQANVPYVEFPCHSLDNKHPIREKMKKENNENNESSSDSSQSSDAESKSEGSESEKEEEEEKEEAEESGGGVTRNEKRKEQRRIALEKKREEKEKLSVLLKMAAERKKTHPNGLHPDISFNEKDIGNDGPECRCPPSIQNNGLKHGYYAGENKVIDCTRTGGANLYYYTMRVFPAPNDTQIHKTQMQINGDMYTFQGFSLVTHAPLPICMTRRPICMYAMDYEFQLVEEKVPDECFAPEDCDDLQEYIFHEIFEMMNFQLRPKHVPAGIETCPMIHIMPRFECFKDGLVHLWSSKTVLAYFLSKGGTPLFDKEEVSSHSRLNDEEFTRNTGRLKQSIILNTQFKPSAVRADWFERDAEKREVYFVHNAIRSASYPIGIAARIAKLENTLNKLKNEKRNSYGMPNPEYEATKLEMDKLKEEARAARILKLREPVAGYIDTGLKPDVVAHIAMVTIACHHIRYNFSISVFEEIIQYQFNDRRLIELAFIHSSFRTSYGTPIDHVKNIVNNCGYRRKYGTEEKREKKKGIVSLFNIMGGEIDGEPILHNERLEYLGDAVVELITSHHLFFMLPHHFEGGLATYRTALVQNRNLAKLAKSCRMDEMLQFAHGADLINEADFKHALANSFEAVMAAVYLDGGLGPCDTIFSKAMYGNDPELKQIWDHCNEHELRREDPLGDRELSQITPALASFHELERRLGIQFNNIRLLAKAFTRRNVPNNDLTKGHNQRLEWLGDSVLQLLISDFLYKNFPNHHEGHMSLLRTSLVSNQTQSVVCDDLGFLEGERLSTGAASNVHKAELKAAESALADLESVSASRGNTKSKKHRRLRRFFF

***Caenorhabditis briggsae* DRSH-1**

MDFTEIHKRSRRKKFQQIHQDRKDEMIQQLGRRFHNQPSTSATYPSAVEDIPLPSEVPNVFGAPPPLTNADFHRNFLVDPDVVVSHSASLIRSNRHIVKAEDAEQYMMVSRERVGTTAERVLEDFNSRVIKPLKAKRRLQIDVPYIDHPLHSMRSKTPERKENEEDSDSEIRSSDSSSDAEYGSDVEEEPDSCRRKKRTHKIQKADSSQTKVEEKERQNTLLRMGIERKRNHPNAIDPHISYNEKGLGNDSPECRCPFPIRNRGLKHGYYAGENQVLKCSKNDRANLHYYTLHVTPAPNESQIQKTQMLINGMEYHFEGFTMVTHAPLPDCMTRRPVFKYSIDYEFQLIEEFMPTECFDPEDCNSIFEYIFHDIFELLDFDLYPKHLPPGTASCPTIHIVPRFVAMENNTTFIWSSKTVLAFFLLHGKNNMFSPEDVEKNCAMSDDAFGRTIAKLKQSIVLNPMKKPSALRADWFSRDLENKEMFLIQNTIRSQNFASPFLPQIAALEKKMSRLKQEKKDSGNKNPHYENLKAELIVLKDKHREARQLKLKLPVKDYIDTGLKPDVVAHVAMAIIASHHIRYNFSLSVFEKVIEYKFNDRRIVELALIHSSFRSYYGTTPDHVKNMISNCGYRKKYGAEERREKKKGIISLFNIMGGETSGGEPILHNERLEYLGDAVVELIASHHLFFILNHHFEGGLATYRTALVQNRNLAKLAMNCRIDEMLQFAHGADLINEAEWKHALANAFEALMAGVFLDSGIAPCDAIFSKAMYGKDPEMKKVWDHLNEHELKIEDPLGDRDLSRITPALTDFHRLEQIIGIEFNNIRLLAKAFTRRNVPFNDLTKGHNQRLEWLGDSVLQLIISDYLYRNFPLHHEGHMSLLRTSLVSNQTQSVVCDDLGFQEFVIKAPHRKNDLKMKDKADLVEAFIGALYVDKGLEYCRSFIRTVFCPRLKHFINSEKWNDAKSHLQQWCLAIRDSRNPNPAMPEYRLLGIQGPTNNRIFRVAVYFRGERLSSAAASNMHTAELKAAENALAALEKASFSRMREKYMSG

RQHRLHRIFFS

***Caenorhabditis japonica* DRSH-1**

MENDEQGGFEYRRHHKRGRRKKYQKQYQDHQRAQLIGQMSRRLQNEGPTSSASVEDIPLPSDMDSMPCGPATVLTTKDYEMNYMEDPQVIATHSGEIQRSNRVVRWAEDVEKYLFSRAGTSHADVLNDFRERVLKPLRETRRRQTDVPRINHQRLVWNPVKSDVAASGDKKEKEKEEEHEEIESLEEFSSSESNSEGDGVPAVSGSKAERQRQLAARQVRKERKEAEKAQQVNRMQLEIQRKRRHPNALDADTNYNEKGLGNDGPICRCPPETLDRGLKHGFYPGEDKLVKCTRQDAQHLFHYTLKVTPAPNENQAYKTAMMINGKEFLFEGFSLLTHSPLPPAMTQRPICKNPLECEYELIEEAIPDNCCYPEDTTHLYEYIFHDVFEMLEFDLYPKRLPEVFESCPIIHIMPRFAFESNGVVNLWSTKSVLAYFLLKSEFDIFSQRDIQEFLTHSDDYFSRQVQKMKQTILINPIFKPSAIRADWFEKEATEAVCWVNNSNRHHSYAPKVHSRITYLERKLKKFESGASGTKHLQYDSAKKELDSLRSKVKSSRSHQIRKSVEGFKDTGLKPDVCAHIVMTILACHHIRYNFGITEIEEKIDYRFGDRRVFELAMIHSSMKSTYGTPADHVKTLLTNCGYRRKYGAEDRRERKKGIVDMFDIVSGEASIEPILHNERLEYLGDAVVELIVSHHLFFMLPHHLEGGLATYRTALVQNRNLAVLAINCRIDELHLFAHGAELCTEPEWRHGLANAFESLLGAVYLDGGIDECDRIYSEAILCFQSTCDMKTQVKVASLQIPLCSRGHTQISGRSFVSRSHVASRLKKWAALHNSLDILFNFQHFINSEKWNDAKSHLQQWCLMLRDPSNPIPEVPEYKVLSVEGPTNTRLYNVGVYFRGKRLASATSNNVHRAHLEVAELALKKLGSARHRINVEKKTRQLLGLATIQLFFHFAGAQLAAASRFAANP

***Caenorhabditis remanei* DRSH-1**

MSDGSGEHFAHLPKTKRSRRKKHQKNYQDMHKDQMIQQLGRRFHNKPSTSSCSPTNVGEIPLPPEAPNTFGAPPPLTEEDYQVNYMVDPEVVSSHSAELIRSYREVVKADEAELYMLNRSRTTTVSVLSEFRTRVLNNVKSKRNLQTNVPQIDYPLHSMTSRKARMRGDGSDNSSSDETGSESDSESEETESGSDSKPGPSTGNLTRSEKRDERKKESQKKKSEEKERQMTLLRMGVERKRNHPNGVDPDISFNEKGLGNDGPECRCPPVIKNRGLKHGFYAGENKVLNCTRICGSNLHYYTLQVKPGPNEGQIYKTGMTINGETYEFEGFSMITHAPLPECMTRRPICKYSLEYEFQLVEERFPIECFDPEDCNHLFDYIFHEIFEMFDFNLHPKHLPPGIVSCPVIHIMPRFVAMKNGVAHLWSTKTLLAYFLINGDKDLFNSQEIIDHCEIPDDAFSRVSNKMKQSILLNTLKKPSALRADWFERDSERREVYIVHNSIRSQNYSTPILVKIAQLEKRLNRMKKDKKFAGKNKEYDVVKRELDQLKDEHRNARYLSIRKSVAGYIDTGFKPDIIAHIAMVIIASHHIRYNFSLSVFEEIIEYQFNDRKVIELALIHSSFRSHYGTPPDHIKNIISNCGYRRKYGSEEKREKKKGIVSLFNIMGGETEGGEPILHNERLEYLGDAVVELIVSHHLYFMLPHHFEGGLATYRTALVQNRNLADLAKNCRIDEMLQYAHGADLINEAEWKHALANAFEAVVAAVYLDGGLGACDRLFSKAMYGSAPNLKKVWDHINEHELKREDPLGDRELSNITPALASFHKLENILNIKFNNIRLLAKAFTRRNVPHNDLTKGHNQRLEWLGDSVLQLVVSDYLFRRYPLHHEGHMSLLRTSLVSNQTQSVVCDDLGFLEFVVKPPHKTPELKMKDKADLVEAFIGALYVDRGIEHCRSFIRIVFCPRLKHFINSEQWNDAKSHLQQWCLAIRDAQNPNPAMPDYRILSIEGPTNNRIFKVGVYFRGKRLASAAESNVHKAELKAAEIALASLESTSVSRMKEKNMGDKDHHRRHRIISDD

***Meloidogyne hapla* DRSH-1**

MASDSLVDVNAALDDIISKRRRGGKRSAPRIDEPLPLGGGRGGGRRNQRRNSGGFNNSGFNNSGFGNIGARRQLGGGGGRRTSNTAGEDVVWINISNLPDSVITGDLQELFQEFNLLGVGVHYDEFGQHMGTADLFVDGRSAKAILREYANIAIDGQKIRFAIVNEQAAATPQFQNQQRRDAVGGGRRRGNAGGGGGRRGGGGGGGAGRRIRSNSGQSSRTYSRSRSPITRAAGGSVSKPRRAGRNASSTNQVKTAAELDRELEAYMNGMKISSDEEGESVMLCDTVNEAVLLSKEQDKRTKSNDKSLQDPISSNSQTWDEQELHEEEENEQLMEDESLNGISNSPFWTQSIAPGKYYNRARGGGQTATPALLSLQHQFKTCIIETIQKGMATQPKSDPPQNLNCSHNCAANCSKHIAGSESDSDGENSGNSSSSSTSSITNSKNAKCTPGDSNNSSPLKKRRGRRRRKRQQIKSFARDEILRKRSHPAALHPDVGFNEPGQLNDGPQCKCSWAAKQTGVRHGKFAGEKAVPRCDLNSSNIERLYHYVLHVEPNPASLSRRPTQIQFDGHCYQFDGFSVFFHKPLPERFPQRPINQWTQQFQVRFLHENAPESFTIADLELFHSFLFEQILELYDLNRQLNIPKEDINFSCPFYHCLPRFARTLPDNGKELLPLSSILSHFISNFSPLVDDRLAQYFHINPLALVDFACQKKGEICINPKKKPLAIRLDLLEQSTDNREFYPIITHFGMKPNAYAFLARPQMQDALYKHLQLRKQLISKPSITFEEKWLLRKSEAHLNSLKKEWDFYSTGICSDMVLHAILLVLACQHVRFHCSLNYLEERLSYSFKNRSLLELALIHPSFRANYGTNSDHAKNILNNCGLRIGLTTTINPIKTPTIPSQNISSPQKKSLTEDLAFKQQLIQKTGRRRGINVLMEIMSMQRSSNNKMKENGIVSRNEPIIRHNERLEFLGDAVVEFLTTIHLFFLFTELDEGGLATFRSALVQNRHLATLGDRLGLQHFM

VYAHGPDLCHSTDLRHAMANTFEALMAAVFLDSGLEQCDKIFASALFVNEPNLLEIWTNLPEHPLKKDTPYGDRHLIPSVPCLKLLVEFERCIGITFKHIRLLAKAFTRRNVPYNFLTLGHNQRLEFLGDTILQLLTSEYLYKQFPYHQEGHLSLLRTCLVQATTQAVVCDDLAMPKYLVIPQALLRKCPQPNLRTKDKADLVEAFLGALYVDRGLLYCRHFCRVCFFPRLKYFIQSQRWNDPKSQLQQSCLTLRNPTSSEPDIPEYKTIGIEGPTNTRIYRVAVYFRNKRLAVGTGASMHHAQMNAAENALIENAHLFKSNGYINNDNNKNNSSTPLTSLSNNRKRHFSSSQQQSSSTSQYFNNHHIYKNNNKLQFFNQTITKQFINKNNNIKNNLITNPIIPPTTTPSIFFPSPPINVLNAQQQRKQPPPSSFLLLPPPPPPTLPPKIYLAANNNEKVVNNNMDTAMNEEEEDSILLPPPPPPPKIL

***Meloidogyne incognita* DRSH-1**

EESPQKPTFTTSQIWDEQELHDDEEGENEQVMEDESLNGISTSPFWTQSIAPGKYYNRARGGGQTATAALLSLQRQFENCIIQTIRDGMATQPKCDPPPPTNSNNCSSHNCDAANNNCSKHVAGSDSDSDNGGDNSDNSSSSSISSHNTSKNVRNNSGGGNSNNSSPLKKRFGRRRRKRQQIRSFAREEILRKRSHPAALHPDIGFNEPGQLNDGPLCKCSWAAKQTGVRHGKFAGEKAFPRCDWNSSNIERLYHYVLYVEPNPASLSRHPTQIQIDGHCYKFDGFSVFFHKPLPERFPQRPINQWTQHFQVRFLHENAPESFTVADLELFHSFLFEQILELYDLNRQLNIPKEEMNSSCPFYHCLPRFARSLPDNGKELLPLSSILSHFITNFTPLVDDRLAQYFHINPLALVDFACQKKGEICINPKKKPLAIRLDLLEKSADNKEFYPIITHFGIKPNAYAFLARPQMQDALYKHLQLRKHLSSKSSITFEEKWLLRKSEAHLNALKRECKSKRNTIVHISSRDFYSTGICSDMERLAYTFKNRSLLELALIHPSFRANYGTNSDHAKNVLNNCGLRIGLTTTTTKTTTTSKSPIIPPSPQQQNLNFSSSPLKNSITENNSLELLTKQQLIQKTGRRRGINVLMEIMSMQRSSSSGNNKIKENNGGVGDGGRIVNRINESIIKHNERLEFLGDAVVEFLTTIHLFFLFTELDEGGLATFRSALVQNRHLATLGDRLGLQHFMVYAHGPDLCHSADLRHAMANTFEALMAAVFLDSGLEQCDKIFANALFINESNSLQIWTNLPEHPLKKDTPYGDRHLISSVPCLKLLVEFERCIGITFKHIRLLAKAFTRRNVPYNFLTLGHNQRLEFLGDTILQLLTSEYLYKQFPYHQEGHLSLLRTCLVQATTQSVVCDDLAMVKYLVIPQALLRKCPQPNLRTKDKADLVEAFLGALYVDRGLLYCRHFCRVCFFPRLKYFILSQRWNDPKSQLQQSCLTLRNFASSEPDIPEYKTIGIEGPTNTRIYRVAVYFRNKRLAVGTGASMHLAQMKAAENALIENSNLFKSNGYINENNNKNIFENKSSCGGGDGNQIENYITSLNKNNSSTPLNFVNNRKRRLPPSFQQHQQQFTSSSPICHHQYFNNNNNNKLQFFNNSTTIPKLFNNNKNNFLPNNNKQILIMPSQTTATTTTMPSSSIFFPSPPPLNVYSNGHQQHQQQKRPQQKQQNPSPPPTSFLLLPPPPPPTFLSPLPSKICIMAANNNNNMDINEEKEEDSSSASLLPPPPPPPKIL

***Oesophagostomum dentatum* DRSH-1**

AVKHARFHWSLLEFEKIIGYNFINRTLIELALRILLQKXFWNECGSCENYSYQLLFSTVCTFYGKQRKEERFSQFNAHYGAVWFXLRRIVEDRTXXTLEYLGDAVVELVVSSRLFFILPHQEEGGLATYRSALVQNRNLAALGKKLRLGDWMMYAHGIDLCDEEDFRKSLANTFEAMLAALYLDAGIEECDRIFANAMFGDDATVKSQWLNVPEHPLKMEHPDGDRMLIASTEELSELTDLENILGFKFQNIRLLAKALTRRNVPYNTLTCGNNQRLEWLGDAVLQLIISDYLYHHFPNHHEGHLSLLRTCLVCNETQSQICEDLGLHYFIIEPPBRVPELSLKDKADLVEALIGAIFVDRGWDYCRVFIYICFLPRLKHFIESKKWNDSKSQLQQCCLALRDVDATDSSVPDMPEYRTIGIEGSTNARHYRVAVYFRNMRLAVGEACTVHLAQMDAAKRALEEYKEMFSSLSKNNVKNAANAFMKKLQD

***Pristionchus pacificus* DRSH-1**

MEHMPGVKKLQLNPGTHNERLEFLGDSVVEMIVTVHLFFLLPQHDEGALATFRSALVQNRNLAALAQEIFLDKLMLMSHGVELLHEPEYRHATANSFEAFMAAVYLDTGKNMDHCERIYGEAMFGREPEMLKLWTNLFDHPLKAEIPGTDRGEIPKAEVLKNLVEFEDSIGVKFAHIRVLYRALTRASGAVNNLNKGTHQSLELLGDTILQMATTHFLYKRFPLLHEGHLSLLRTCLVSNKTQSVICDDLEIGKYVVDAPRRNHAKRELKMKDKADLVEAYICKFCYL

***Trichinella spiralis* DRSH-1**

MKSTRCVREELSVPQSKESDSMDQNPSSSFEEPLLVGLNDFIDQDFDIDDEEQEEEEVTIFSQSSPRLTVSCVSAHDYYKTIACGQSDVKVATQKLTALHCEFDQHVVQLFRSAKQTWQKDENDGASQSPVKLQCSAESDEQDEENACEGGKQPNGNLNGDSHNNYDVNDNGRFKDALKELERKRRHPARLHPDIWYNDAGQLNDGPSCRCCWRARQKGISHGRYPGEARVEPCDPLSNNANRLHYYRVVIKPQANLFQKFSITEIEYKDKVYSFDGFGILSHELIDRNMPLCRLNRWNIDYEVSLVEEVVPDFFSVRDLDLFWEYVFVELLELYDVKLFAANVEDGCPIFHIYPRFVCKRKEKTGCTQQMLPMSVVLEHLLQNCRLIFDQPAERGDFNDRVNKHLDKFSEKLKGQVVANPWKRPVALRLDQLDRMQKAPDGYSVIVHFGRHPTVTTYAGTPEYQRAWKRFAKFRRVVMLQSYPRTEDRAKLAKMEAHLTELKAKNSVKRDLIMEVSCQGFMQTGIWSDVVQHCLILLVLLNHIRFHRCLRVFQQRIDYSFRKPSLLELAFTHPSYRADSGTNADHVRNVLNNCGLRQLEFGSKRTNPWNSRKRGVSCLVNVMSRMGEKKSRASSTVNNERLEYLGDAVIEFLTTIHLFYIFPDLEEGALAPYRTLMVQNSHLSKLAAKMGLDEFMLFAHGPDLCFEEDMRHAMANAFEAILGAVYLDGGVHVADRVFGRFLFPPDWNQALHWVWFHPPRHPLQQEYPDGDRHLIHQCPTLQKLTEFEQRIGVRFNHIRLLARAFTRKCVGYNFLTLGHNQRLEFLGDSVLQLVTTEYLYKHFPDHQEGHLSLLRSSLVNNRTQAVICDDLEIPNFILQPPAFLRNEPQELRMKDKADLVEAFLGALFVDHSLDYCAAFVRHCFLPRLKQFIEEQRWCDPKSQLQQCCLALRNPSGAQSTVKEEIWSKILCETQSRSKCALPTASVIVLGDSDSGKTSMVARLQGVEEPKKGAGFEYHYLEINPDYKVGSYAYQLSSSLPDISAGDVPRLGVWVLDGDPLYAPLLRFALPAENLKHSVALICCSMAEPWNIGQSLQRWTDVLEKHLNQSGLYSTHVLSECRERQVRFWQEYVEPLDSSSHSELGQKVPSMEPDQILLPLGQSTLTRNLGLPLIVVVTKCDLLSSYEKQFDLKDEDCDLIQKQIRQFCLKHGAALVYTNVKEGRNCDLLYKYILHRVFGFPFTQPACVIDKEAMFIPAGWDNEKKISIIEDGLPEGAEEAFLNRFSDQLVNPLNNSFNDNNITAARLQTLLNTTLPNTQSPGQHRPDGSSPMPMSITSPRHSSDKSRPSPIGGQSSNGLFSSASVSAGGSATSAQSKKADSIGKIAMQTVSGTGTGAGAAGSVGGGPVGPSSEGALAQFFNNLLVKRPASASTAAGSAKLTSESSVRERTFDDSESETQTE

**XPO-1**

***Ancylostoma caninum* XPO-1**

VHVFYEAVGHIISSASDEPDQQADLIEKLMALPNSVWDEIIANAGENMSVLEEPEVTRNLLNILKTNVACCKAAGNPFITQLSRLYIDLLSLYRILSEKVSVAVEQNGQEVLKMPLLKTMRAVKREILILISTWVASAKDRQMVLENIVPPLFDAVLFDYQKNVPAAREPKVLSLLSIIVTKLGSMLASQVPQILAAVFECTLEMINKDMEAFPEHRTNFFQLIHALTVECFPVFLALPQEQLSYIIDAVVWAFQHSMRNVAEIGLDILKDMLDRVEHLPRDQSQPFYKRFYMQILQHVLAVVADSSQVHVAGLTYYAEVLCRLFKACEFLITVPLNDENPKQSNVDYIYEYIASIFVQHFTNLTEAQIRVIIKGFFSFNTDQGGMRNHLRDFLVQIKEFNGEDTSDLFLEEREAEIQAVQAKKNAVPGMLDPNNIVDEDEMR

***Ascaris suum* XPO-1**

MSIIDPSVDLADAYKRGTDADQKFIANLAQYLGTFLKENAPIVEVLEETDANTDLKRAHQMALQYLLKISMVEDVEIFKVCLDYWNWLCAELYREFPFQIDRPLISSFPLLNRTQQEPPRRALYNSVLSDLRLVMISRMAKPEEVLVVENEQVYLTHLDYKDTEMKMTEKLQNQLCWAIGSISGAMMEEDEKRFLVTVIRDLLGLCEQKRGKDNKSVIASNIMYVVGQYPRFLRAHWKFLKTVINKLFEFMHETHEGVQDMACDTFIKVAHKCRRHFVVIQAGEANPFIDDILGGLSSIICDLSPPQVHVFYEAVGCLISAQNDPPIRECLIERLMQLPNSIWEEIILHASQVGHLQNR

***Brugia malayi* XPO-1**

MTMMTVAALQKAGEALLSSEKIEIPLLDQVVNIMNQSTGETQQLASKILTELKQKDTSWTRVDGILEYSQLMETKYYALQILESLIETRWKSLPREQCEGIKSFIVELVIKISSEEITSPQIKTYLQKLNLVLVQIVKQEWPKHWPTFMADIVGASKVNDNLCLNNMIILRLLSEEVFDFDGEMTQAKAYHLKKTFCSEFQAVFNXCYTVMESSDNAPLVDATLHTLHRFMSWIPIGYIFETSLIDLLTKKFLGVAMFRCITVQCLSEIASLSVAQMEQQNALYVNQVKSLFRNSMVQIMNTIDPGVDLSDAYRRGTDADQKFIANLAQFLGTFLKENSQLVEVFGDKTDELDLKNAHEMALQYLLKISMVDDVEVFKICLDYWNWLCAELYREFPFQIDRPIISALPMFVELQEAPRRLLYSNVLSELRLVMISRMAKPEEVLVVENDQGEVVRELIKDTDSITLYKTMRETLVYLTHLDYKDTEMKMTEKLQNQVNGKEWSWKNLNTLCWAIGSISGAMMEEDEKRFLVGVIRDLLGLCEQKRGKDNKSVIASNIMYVVGQYPRFLRAHWKFLKTVINKLFEFMHETHEGVQDMACDTFIKVAHKCKRHFVLTQAGETGPFIDEILGGLNSIICDLSPQQVHVFYEAVGCLISAQTDPAIRESLIERLMQLPNSIWEEIISHASQDINVMKEQEVVKNIVNILKTNVAACRSIGEPFICQLSKIYLDMLNVYKVTSENISGLVAQSGEEVLKQPLLKQMRAVKREILTLISTWVGKTQDANVVLENFVPPLFDAVLFDYQRNCSAAREPKVLSLLSIIVSQLQSSINPEVIRILDAVFTCTLEMINRDMEEYPEHRLNFFSLLQALNHECFDVLISLPPEHFRLIVDAVVWAFKHTMRNVAEIGLDILKDMLTQFGVHRDKERAQTFYKHFFMEILVHVLTVVTDSNQIKILGLSCYADILCALFYAAEISITEQLNPPQSNIDYIYMHISETFAQAFDNLTPDQIRVTVKGFFSFNI

DSVKMKNHLRDFLVQIKERVGEDTSDLFIEEREQEIQNVQNAKKEVPGMLNPHEIADDDSMK

***Caenorhabditis brenneri* XPO-1**

MEVLAQAKTQFFEGDRIDVGLLDEVVMIMNQRSGREQAEANQILMQLKADRDSWQKVDAILQYSNLNESKYFALQILEAVIQHKWKSLPQIQRDGIKQYIISKMLELSCQQSTMEQNQLLLHKMNLVLVQIVKQDWPKQWPTFISDIVESSKTNETVCINNMNILSLLSEEVFDFGSQNLTQAKEQHLKQQFCGQFQEVFTLCVSILEKCPSNSMVQATLKTLQRFLTWIPVGYVFETNITELLSENFLSIEVYRVITLQCLTEISQIQVETNDPSYNEKLVKMFCSTMRHINTVLSLELDLAALYKEASDQDQKFISSLAQFLVAFIKEHVHLIEVTDEPLTDMKTMMRDAHQYAIQLLLKITLIEEMEVFKICLDCWCWLTAELYRISPFIQPSSLYGMMNTMREHPRRMLYREYLSVLRTTMISRMAKPEEVLIVENDQGEVVREMVKDTDSIALYRNMRETLVYLTHLDNKDTEMKMTEKLASQVNGGEFSWKNLNRLCWAVGSISGTMIEEDEKRFLVLVIRDLLGLCEQKRGKDNKAVIASNIMYVVGQYPRFLRAHWKFLKTVINKLFEFMHETHEGVQDMACDTFIKIAIKCKRHFVIVQPAENKPFVEEMLENLTGIICDLSHAQVHVFYEAVGHIISAQIDGNLQEQLIMQLMEIPNRTWNDIIASASVSDSILEEQEMVRSVLNILKTNVAACKSIGSAFVSQLGNIYGDLLSVYKILSEKVSRAVTTHGEDALKLPLVKTMRAVKREILILLSTFISKNNDHKLILDSIVPPLFDAVLFDYQKNVPQAREPKVLSLLSILVTQLGSLLSPQVTNILGAVFQCSIDMINKDMEAFPEHRTNFFELVLSLVQECFSVFMEMPPEDLGTVIDAVVWAFQHTMRNVAEIVA

***Caenorhabditis briggsae* XPO-1**

MEVLAQAKQQFAQNDRIDVNLLDQVVRIMNQMSGKEQAEANHILMSLKEDRDSWTKVDAILQYSNLNESKYFALQILEGVIQHKWKSLPQVQREGIKTYIISKMLELSSKQETMASNQLLLHKMNLVLVQIVKQDWPKAWPTFITDIVDSSKTNETVCINNMNILSLLSEEVFDFGSQNLTQAKEQHLKQQFCGQFQEVFTLCVSILEKCPSNSMVQATLKTLQRFLTWIPVGYVFETNITELLSENFLSLEVYRVITLQCLTEISQIQVETNDPSYNDKLCKMFCSTMRHISSVLSLDLDLAAVYKEASDQDQKFISSLAQFLVAFLKEHVHLIEITDEPLSEAKIMIRDAHDYAIRLLLKITLIEEMEVFKVCLDCWCWLTAELYRISPFIQPSSIYGMMNNTREHPRRQLYREYLSTLRSAMISRMAKPEEVLIVENDQGEVVREMVKDTDSIALYRNMRETLVYLTHLDNKDTEMKMTEKLASQVNGGEFSWKNLNRLCWAVGSISGTMVEEDEKRFLVLVIRDLLGLCEQKRGKDNKAVIASNIMYVVGQYPRFLRAHWKFLKTVINKLFEFMHETHEGVQDMACDTFIKIAIKCKRHFVIVQPAENKPFVEEMLENLTGIICDLSHAQVHVFYEAVGHIISAQIDGNLQENLIMQLMDIPNRTWSDIIAAASTNDGVLEEHEMVRSVLNILKTNVAACKSIGSAFVSQLGNIYGDLLSVYKILSEKVSRAVTAAGEEALRNPLVKTMRAVKREILILLSTFISKNGDAKLILESIVPPLFDAVLFDYQKNVPQAREPKVLSLLSILVTQLGSLLCPQVPNILGAVFQCSIDMINKDMEAFPEHRTNFFELVLSLVQECFPVFMEMPSDDLGTVIDAVVWAFQHTMRNVAEIGLDILKELLARVSEQEDKVSQPFYQRYYTALLKHVLAVACDSSQVHVAGLTYYAEVLCALFRAPEFSIKVPLNMENPQQSNIDYIYESIGSDFQNHFDNMNADQIRIIIKGFFSFNTEISSMRNHLRDFLIQIKEHNGEDTSDLYLEEREAEIQQAQQRKRDVPGILKPDEVEDEDMR

***Caenorhabditis japonica* XPO-1**

MSGAMDVLHEARRQFTQSDRIDVNLLDQVVQIMNQRSGKEQAEANSILMELKENRDSWTKVDAILQFSTLNESKYFALQILEGVIQHKWKSLPQVQRDGIKSFIITKMFELSADQSTMEQNQLLLHKLNLVLVQIVKQDWPKQWPSFITDIVDSSKTNETVCINNMNILSLLSEEVFDFGSQNLTQAKEQHLKQQFCGQFQEVFTLCVNILEKCPSNSMVQATLKTLQRFLTWIPVGYVFETNITDLLSDNFLSLEVYRVITLQCLTEISQIQVETNDPSYNEKLVKMFCSTMRHISSVLTLDIDLAAVYKDASDQDQKFISSLAQFLVAFIKEHVDLIEVLDEPMTEMKILIRESHDYAIQLLLKITLIEEMEVFKVCLDCWCWLTSQLYRISPFVQPSSLYGMMSQVREHPRRQLYREYLSVLRSTMICRMAKPEEVLIVENDQGEVVREMVKDTDSIALYRNMRETLVYLTHLDNKDTEMKMTEKLASQVNGGEFSWKNLNRLCWAVGSISGTMIEEDEKRFLVLVIRDLLGLCEQKRGKDNKAVIASNIMYVVGQYPRFLRAHWKFLKTVINKLFEFMHETHEGVQDMACDTFIKIAIKCKRHFVIVQPAENKPFVEEMLENLTGIICDLSHAQVHVFYEAVGHIISAQIDGKMQEQLITQLMEIPNRTWGDIIAAASSNDVVLEEPEMVRSVLNILKTNVAACKSIGSAFVSQLGNIYNDLLSLYKILSEKVSRAVTTIGEEALKNPLVKTMRAVKREILILLSTFISKNNDHTLILESIVPPLFDAVLFDYQKNVPQAREPKVLSLLSILVTQLGVSLTCYFSSLFSKLQSLLCPQVPSILGAVFQCSVDMINKDMEAFPEHRTNFFELVLSLVQECFPVFMEMPADDLRTVIDAVVWAFQHTMRNVAEIGLDILKELLARVREQDDAIAQPFYKRYYIDLLKHVLAVACDSSQVHVAGLTYYAEVLCALFRAPEFSIKVPLNDANPSQSNIDFIYEHIGGNFQAHFANMNQDQIRIIIKGFFSFNTEIVSMRNHLRDFLIQIKEHNGEDTSDLYLEEREAEIQQAQQRKRDVPGILKPDEVEDEDMR

***Caenorhabditis remanei* XPO-1**

MEVLAQAKLQFAQNDRIDVNLLDQVVRIMNQMSGKEQAEANQILMALKEDRDSWTKVDAILQYSALNESKYFALQILEAVIQHKWKSLPQVQRDGIKSYIVSKMLELSAEQSIMEQNQLLLHKMNLVLVQIVKQDWPKQWPTFITDIVESSKTNETVCINNMNILSLLSEEVFDFGSQNLTQAKEQHLKQQFCGQFQEVFTLCVNILEKCPSNSMVQATLKTLQRFLTWIPVGYVFETDITELLSANFLSLEVYRVITLQCLTEISQIQVETNDPSYNEKLVKMFCITMKHISSVLSVDLDLAAVYKDASDQDQKFISSLAQFLVAFIKEHVHLIEVTEEVIPENKRIVREAHDFAIQILLKITMIEEMEVFKVCLDCWCWLTAELYRISPFIQPSSLYGMMSTNREHPRRRLYREYLSALRTTMISRMAKPEEVLIVENDQGEVVREMVKDTDSIALYRNMRETLVYLTHLDNKDTEMKMTEKLASQVNGGEFSWKNLNRLCWAVGSISGTMIEEDEKRFLVLVIRDLLGLCEQKRGKDNKAVIASNIMYVVGQYPRFLRAHWKFLKTVINKLFEFMHETHEGVQDMACDTFIKIAIKCKRHFVIVQPAENKPFVEEMLENLTGIICDLSHPQVHVFYEAVGHIISAQIDGSLQEALIMQLMEIPNRTWNDIIASASTNDSVLEEHEMVKSVLNILKTNVAACKSIGSAFVTQLGNIYSDLLSVYKILSEKVSRAVTTAGEDALKNPLVKTMRAVKREILILLSTFISKNGDAKLILESIVPPLFDAVLFDYQKNVPQAREPKVLSLLSILVTQLGSLLCPQVPNILGAVFQCSIDMINKDMEAFPEHRTNFFELVLSLVQECFPVFMGMPQEDLSTVIDAVVWAFQHTMRNVAEIGEFIHKKEKNMRTNTKSYWLLLFNGL

***Haemonchus contortus* XPO-1 (Likely exon-intron boundary issue with predicted protein)**

NLTGKLIRHFLFSIFTIQMREVDKTFTHVFIKRDGIRVLDNFTYNLNFSLIVLHY*HLFWFCHSGNHDGTQMRQMLPE*LIQLSPRMFFTASWTSAGKRYSDGWRWWCKWIQTIEFRSQGTPVVKTYLNRGPDDQHGNIKNNTTNLEDFNVFHLADR*Q*LYGVLVIFGNLGTGFNTFGRVFRQQLDFANKNSKLKSS*IF*L*RSAIALITKPGRNNETDRNLALF*RVEVYLNQLRARDSIQ*SHCHTGGPVIPCVTNHFGK*R*RTLISHFFRNNIYCHKKVFLMLIKICSDLLGLCEQKRGKDNKAVIASNIMYVVGQYPRFLRAHWKFLKTKTFQAFPEHRTNFFQLIHALTVECFQVFLALPPEQLSYIIDAVVWAFQHSMRNVAEIGSFCSEEVFDFGSQNLTQAKEQHLKQQFCGQFQEVFTLCITILVSFRFCCLISFPVQLFFVF*EKCPANSIVEATLKTLHRFLSVINKLFEFMHETHEGVQDMACDTFIKIAMKCKRHFVIVSFFLSKSFKVNGSEFSWKNLNTLCWAVGSISGTMIEEDEKRFLVLVIRGWSFFCVIGLTYYAEVLCRLFKACEFLITVPLNDDNPKQSNVDYIYEYIANIFVQHFTNLTEMVLENIVPPLFDAVLFDYQKNVPAAREPKVLSLLSIIVTKLGEKCPANSIVEATLKTLHRFLSWIPVGYVFETDITQLLSENVCFLVYRNLLNILKTNVACCKAAGNPFITQLSRLYIDLLSLYRILSEKVSTAVEQNGQEVLKFLSLEVYRVVTLQCLTEISMIQIEAEDNAYKEKLCAMFCATIKEVSVHVFYEAVGHIISSASDEPDQQADLIEKLMALPNSVWDEIIANAGENMAVLEDAEVCRSVKLFVEKLYYFQIGSLLGEGADLAAAYQKGSDQDQKFISCLAQFLVAFLKDHSALVEVQIRVIIKGFFSFNTDQGGMRNHLRDFLVQIKVHVFYEAVGHIISSASDEPDQQADLIEKLMALPNSVWSMLASQVPQILAAVFECTLE

MINKDMEMQVGEQTPFIDEMLKNLSGIICDLAPSQVRLTVFPRILFTYVNSNLDMSFWESTAVEQNGQEVLKVTSTCTFCIESFGMALLVCLLTVFKMPLLKTMRAVKREILILISTWVASAKDRQVHKLIYRFPFQEFNGEDTSDLFLEEREAEIQAVQAKKNAVPGMLDPNAMADEDEMRGLDILKDMLDRVARIWLFLKTRRYAAQFGLQIPEHSFQP*HSRPKQPVLPYFYSH

***Meloidogyne hapla* XPO-1**

MELDFLRQAQYKLLEGGEKFDVSSLDKVVGLMNNTTGEAQKAASDLLARFKENPDSWTKVDAILELSALLETKYFGLQILEQLIQTRWKALPREQCEGIKGYIVNMILEISSDAEKSERMKLLLQKLNLVLVQIVKQEWPRLWPSFISDIVGSSRNGQSLCMNNMTILRLLSEEVFDFGTGLTTARAVQLKQQFCGQFEEVFMLCYEILENSDNAQLVYATLSTLHGFLDWIPVGYVFENNLIDLILKFLPFPAFRSISVQCLVEISSISTEDNPQYGSRLVHLLKSVMNIISQQLPLTVDFADSYAKGRSEDQKFISDFAQLLGTFLKEHSNLVEVLELNPTQDQLEVKQAHALALKYLLKIGQVEDVEVFKICLDYWNWLTMELFRESPFEQAEHPLMDTLRRYNRNESPRRKIYADILSDLRVLMISRMAKPEEVIIVLNENNEAVRELVKDTDSMMLYKTMRETLVLLTHLDYRDTELKMTEKLQNQVNGTEWSWKNLNTLCWAIGSISSSMHEDDEKRFLVTVIRGCLLFLTYNNCYNLIDLLGLCEQKRGKDNKAVIASNIMYVVGQYPRFLRCHWKFLKTVINKLFEFMHESHEGVQDMACDTFIKIVIKCKLHFVVIQTGEAQPFIEEILQNLNNIICDLSQPQVHVFFEAVGHIIYAANTHQAQERLIEKLMVLPNSIWTEAIEAASKDVSIFTDPEVLKNLTHILKTNVAACKSIGAPFFSQLKRILNDMLSIYQVISGNLNKAVNEQGEHVLKWPLIKHMRVVKKEILTLLSTWISRAFEGRIMEEALLPIPLVIENIIKPLFSTVLRDYEMNVPQAREPKVLSLLSITIVSLKEEASSQVPEILDAVFTCTLDMINKDMEAFPEHRTNFFQLLKALNTHCFNVLIALPDKVLGLIIQAIVWAIKHTMRNVAENGIEILRDLLGKVASMTDRAQARIFYQKHFMTIMEHVLGVLTDNNQVQFVGLTNLAETVCILFQAAENTIDVPLNPSNPSQSNTDFVYETITTLFV

NHFKNLTEAQIVVTVKGFISYNRILNKMREHIRDFLVQIREEAGDDTADLFLEEKEAEIQRIQAEKQAIPGVRNPNELVEEDMA

***Meloidogyne incognita* XPO-1**

MELDFLRQAQYKLLEGGEKFDVVGLMNNTTGEAQKVASDLLARFKENPDSALLETKYFGLQILEQLIKTRWKALPREQCEGIKGYIVNMILEISSDAEKSERMKLLLQKLNLVLVQIVKQEWPRLWPSFISDIVGSSRNGQSLCMNNMTILRLLSEEVFDFGTGLTTARAVQLKQQFCGQFEEVFMLCYEILENSDNAQLVYATLSTLHGFLDWIPVGYVFENNLIDLILKICLDYWNWLTMELFRESPFEQSEHPLMDTLRRYNRNESPRRKIYAEVLSDLRVLMISRMAKPEEVIIVLNENNEAVRELVKDTDSMMLYKTMRETLVLLTHLDYRDTEL KMTEKLQNQVNGTEWSWKNLNTLCWAIGSISSSMHEDDEKRFLVTVIRDLLGLCEQKRGKDNKAVIASNIMYVVGQYPRFLRCHWKFLKTVINKLFEFMHESHEGVQDMACDTFIKIVIKCKLHFVVIQTGEAQPFIEEILQNLNNIICDLSQPQVHVFFEAVGHIIFAASTHQAQERLIEKLMVLPNSIWTEAIEAASKDVSIFTDPEVLKNLTHILKTNVAACKSIGAPFFSQLKRILNDMLSIYQVISGNLNKAVNEQGEHVLKWPLIKHMRVVKKEILTLLSTWISRAFEGRIMPLFSTVLRDYEMNVPQAREPKVLSLLSITIVSLKEEASSQVP EILDAVFTCTLDMINKDMEAFPEHRTNFFQLLKALNTHCFNVLISLPDKVLGLIIQAIVWAIKHTMRIEILRDLLGKVASMTDRAQARIFYQKHFMTIMEHVFMTIMEHVLGVLTDNNQVQFVGLTNLAETVCILFQAVENTIDIPLNPSNSSQSNTDFVYETITTLFVNHFKNLTEPQIALTVKGFISYNRILNKMREHIRDFLVQIREEAGDDTADLFLEEKEAEIQRIQAEKQAIPGVRNPNELVEEDMA

***Oesophagostomum dentatum* XPO-1**

VCLDYWCALTSELYRLNPFTPPSPPIAISFSCGSAVGGKEHPRRKLYKEHLSNLRTIMISRMAKPEEVLVVENDQGEVVREIVKDTDSITLYKNMRECLVYLTHLDCKDTEQKMTDKLASQVNGSEFSWKNLNTLCWAVGSISGTMVEEDEKRFLVLVIRDLLGLCEQKRGKDNKAVIASNIMYVVGQYPRFLRAHWKFLKTVINKLFEFMHETHEGVQDMACDTFIKIAMKCKRHFVIMQVGEQTPFIDEMLKNLNGIICDLAPSQVHVFYEAVGHIISSASDEPDQQADLIEKLMSLPNSVWDEIIANAGENMAVLEDPEVTRNLLNILKTNVACCKAAGNPFITQLSRLYIDLLSLYRILSEKVSVAVEQNGQEVLKMPLLKTMRAVKREILILISTWVASAKDRQMVLENIVPPLFDAVLFDYQKNVPAAREPKVLSLLSIIVTKLGSMLASQVPQILAAVFECTLEMINKDMEAFPEHRTNFFQLIHALTVECFPVFLALPQEQLSYIIDAVVWAFQHSMRNVAEIGLDILKDMLDRVEHLPPEQSQPFYKRFYMQILQHVLAVVADSSQVHVAGLTYYAEVLCRLFKACEFLITVPLNDENPKQSNVDYIYEYIASIFVQHFTNLTEPQIRVIIKGFFSFNTDQSGMRNHLRDFLVQIKEFNGEDTSDLFLEEREAEIQAVQAKKNAIPGMLNPNTIADEDEMR

***Pristionchus pacificus* XPO-1**

MAQLAFIERAKKQFSEEGKFDVDLLDQVISAMNVGVGEDQRQANLLLMEVKENPSSWTKVDAILEYSKQAESKYFALQILEGTIQTRWKTLPTVQRDGIKTFIVNLVLKLSESTEEAAANALLLHKLNLVLVQIVKQDWPKNWPTFITDIVAASKTSDSICVNNMNILRLLSEEVFDFGSQNLTQAKEHHLKQQFCGQFQDVFDLCLAIFEKCYDTAMVEATLRTLHRFLSWIPVGYVFETNITELLANNFLPLEMFRSIALQCLTEIAQINITSDDVAYQQKLKAMFVTTMAEVQRLLGGVQDLNAAYKSSNDKDQKFIANLAQFLVSFLKEHSSIVEALGKHEEVREVHAYALQMLLQISEVEETEIFKICLDYWNWLTAELFRACPFPASGGTSLGGFLGMGGGFMGGQTAGTPEERRKQLYAPSLSRLRQIMICRMAKPEEVLVVENDQGEKHSLHDHVIREVFKDTDSIALYKNMKETLVYLTHLDYKDTELKMTEKLATQVNGNEFSWKNLNTLCWAVGAISGTMSEEDEKRFLVLVIRDLLGLCEQKRGKDNKAVIASNIMYVVGQYPRFLRAHWKFLKTVINKLFEFMHETHEGVQDMACDTFIKIAIKCKRHFVITQMGEGQPFVDEMLCNLSSIICDLSPPQVHVFYEAVGTIISAQTEPSIQALLATERMVATGQLVEASGERVAEERMALIDKLMTLPNTVWDEIIAQAETNIEALKDQEILRNMINILKTNVAACKAIGMPFACQLTRIYDALLSVYRIHSQDVSEAVKEHGEDVLTNPIVRQMRNVKREILVLLSTWVAKADDDDTVMDSFVPPLFDSILFDYQRNVAAAREPKVLSLLSIIITKLREKISPEVPRILDAVFNCTLEMINKDMEAFPEHRTGFFRLLLSLSQSCFPVFLALPEETMQFVIDAVVWAFQHSMRNVAEMGLDILKDMLTKVAMLPEEQAQPFYKNHYVQLLQHVLAVVADSNQVQVAGMTYFAEIMCLMFRALESSITVPLNPTNPGQ

SNIDFISDHIGNIFVQHFTNLTT

***Trichinella spiralis* XPO-1**

MAEEILVKASKLLEFNTPVIDVSLLDAVINLMYCGTGEIQRKAQEILTMLKENNEAWTRVDAVLEYSRSLQSKYFALQILENLVNTRWRRLPRDQCDGIKKYLVDRIISISSNPSLSEDEKVFLNKMNMVLVQIVKREWPKHWPTFISDIVGSSRSNESLCRNNMVILKLLSEEVFDFSSGQMTQTKANHMKQQFCSEFRAIFELCQHILESSTNVMLVEATLNTLLGFLVWIPVGYIFETNLIESLTAKFLSILPFRNVTLMCLTEIAGVTFPKNAPPAYSSTICRLFSRTMQQLNTVRLNMLPPHTNIPEAYAMGNDNDQKCISNLALFLSTILRQHCKIIEAECKEKIGELLGTPFDLAMNYLLAISEVDDMEVFKICLDYWNWLVAELYREPPLTNPLLTMNLAIQLIRKDSLCTRYHYVPYLSRLRSVIISRMAKPEEVLVVENDEGEVVRETIMDTDAISLYRTMRETLVYLTHLDCADTERIMTEKLQNQTNGSEWSWKNLNCLCWAIGSISGALMEEDEKRFLVMVIRDLLGLCEQKRGKENKAVIASNIMYVVGQYPRFLRAHWKFLKTVINKLFEFMHETHEGVQDMACDTFIKIANKCRRHFVIIQAGEKEPFIEEILASLNTIICDLSPAQVHVFYEAVGYTISAQPVQIVRDNLIERLMSLPNHTWDDVILKATTNVEILKDIEVVKNLVNILKTNSAACRSIGYPFLPQLCRIYLDMLNVYKVTSENINSAVTLHGESVLKQPLIKCMRAVKTEVLRLINTWISTLSSISESARIPELPSIYMSFVPPLFDTVLFDYQRNVPSAREPEVLSACTVLITQMKEKVSEDVPKILDALFGCTLEMINKDFEDFPEHRINFFQFIRSIIVNCFTGNIEELKKKPLMLIPPAQFTLIVDAIVWAFKHTTRNITEIGLEILDRLLDSFSTKVSPDMAQSFYQQYYLTILSHLLSVVTDSTMAQVAGLTVFAVTLGRMFRELEEGLIKVPLQGPGQVKSNVEYVLEYTFELLKKAFPHLTDEQVRIIVQGILSYDNDVEKLKEHLRDFLVQIKEYTGEDTSDLYLAEKEQEVKAAMEAKRRAAEAVPGILNPHEISEEMIEEPMGNGPALNA

**XPO-2**

***Ancylostoma caninum* XPO-2**

MHFRLQVCYSLLHSLVLEVLIKNTSXYLPYLLSTELWARSANVPAALSVLETFLKRCPEAVMREHGALVMQHYSRLVGSKSLDQYGFQLANAILPVIETVQGVENPMSVLLNNMFRRVQFSKTPKFMKHFVVFLCRFAIVRGAELLARSVEAIQAGMFRMLLEKVVVAELTNLQNLTTTDDKRTIAIGIANMLADATNYVGDQYGALAVGVAQLVEAPSASDRPVLSPEEEQASMYNAEGEFTNPYCRLSYAPRPDPLVPEITNYKNYLARAVLQRGPAANSAVEACIPAELRTHLMAYA

***Ascaris suum* XPO-2**

MPADRGEPLEHFAVLPHIRKQLSQVVCIMGSHDFPGSWPELIDVLAGHLSGADLDKLMATLSTMDELFRHYRHEMKSNKLWSELAYVLQHVAAPLTELFTKMVVYIEQKDSMPLDQCVVWLTVLLLIAENFHSLNSQDLPEYFEVACLLSLCLISDFLSLTIFCSRLDACEVRRSFRGIVAHGLVEVIKDHLGVWMNAFLELLKLKVAALDAQADDSEASVLDKLKCSICEIVTLYSQ

***Brugia malayi* XPO-2**

MAAGAEAVAECLKRTLEPNAQIRRIAENDLKQMEQLPGFGMVCFELIFSQQTLPAVALAXAVSLKNFVKENWNKEKCLVEINDEERSQLRSRALESMLSTSGNIQKQLSQVVCVMGKHDFPEEWPDLITILAQNLTGIDLDKLTSTLYTLDXLCKKYRYEVKSNRLWQELVIVLQAIAAPLTDLFAKMIECIXNKDLMSETECRSWIEVTTLITKCFHSLCSQDLPEYFEDHLNTWINGFMALLRLQIPQMDAASIDSEANILDKLKCCICEIITLYSQRYEEEVMPFMMPSDKSSATHEKVEQEYWLIECIWQLLVSIDEKARYDTLVNASLGFLSSVCQRSQYSTIFDHEEMLRTLCEDVIIKNVMLRKCDFELFKDDPFEYMRKDIEGSDIGTRRRGASDFLRSLCRRPDESRILAILSRVLQSFLQESVADLTNNWLKKDVVYCLITAVAVKAETVKHGATVTSDLVDVVDFYQTHVHSDIFMDDVNALPILKTDALKYIVVFRNHLRPDHLIGVVSAFLKLLSSRHTILHQYTAYALERLMLVRCKETGKVLLTHENVPLGSLIVALFACFEADPKAQNSHYLMKALMRCFNIIDTETAKSSAHIVDKLATMIGVAVKNPVNPLHLHFVFESLCVLIKQVYAVVDGGIDKFVVPLIENIFSSDAVDFVPYALQITALLLDQAEAQKQKTGTSCVDSYLPFFGNLMKGELWLRTANIPAALLVIESFMRSHGKHILDNYSNSLLAVFQKLISSKALDQHGFQLASIFLHYVNQVDVLTESALLIPMLRRIQFTKTTKFMKNFVLFLARFAILRGSVVLCQVLESIQTGMFMMVVEKILIPELGKMYNTTTYDEKRLCCIGFANLAADTVDKLGLQYGILVESLVRLVEASACGPTPLNADDVEEQGIGLSTLELERNDPYCKLSYAQHPDVIAAEIVNFKAYLAEAVMVRAVILKADSASCINEEIRGFLAGYAQQV

***Caenorhabditis brenneri* XPO-2**

MEQIGAALQETLEPTAEIRKRGEESLRTLQSSPGFIIQILQLVVNEQQQIAPQIRMAAAVALKNFVKRNWGPAPEVEMSQEDEEQFRNMLLEAMFNTKSNVQEILSNALYLIAQRDFPEKWPELVPYLSKFLTGSDLNHLVASLASMDQIFRKFRYQSKSTELWKELAKCLQSTQEPLTLLLRNMMEVCQQKDNLGAAEIAQWLKVLNLIAKVYHSLCSQDIPEYFEDHLNDWMPHFLQLVQIEAPTQTSSAGEPTTLDELKHEICEIFTLYSQRYEEEIAPYVPDIISAVWRLLETTGPDTRYDTMVCAALEFLSMVSQRQYYESHFTGEGVLRTLAENVCVQNLLLRQQDMELFEDEPLDYMKRDIEGTDVGTRRRGAIDLARGLCRRFEDKMFPCLKEIIGILINSGEWIKIDIVYSLITAIAVKTETAKNGVTATNPIIDINDFFIAQVASHLNSNVNDTPILKADALRFAVTFRNQLAPEHLMTTIKASDALLSSNTPILHKYAAYAIERILLADTKKIFSAHNLPVASILQNLVAAFDKDPKAQNSPYLIKAILRIIVILDDETIRHADAIATKLAQLIESATKNPADSVHTHFLFETICVLVTKTRTIGASLDAQLLPLIEVIFREDIEDLIPYALQITGVLVSSCIARNASIDQFSAFLPFLLSERLWARSANVPAALSVLEVLLSVNAQRVVSENSSLLLNHVSRLLGSKTLDQYGFQLAAAILPSIEHFEGQAMTFLLNTMFRRVQSSKTPKFIKLFIVFLCRFTILRSAQDLVRSCENIQTGMFGMLIEKVVCIEMPALKQTTTAPEKRIIAIGMANLLAEATQQLVGQYGILSYETAMLLEAASASDRAVLSPEEEQASMYNAEGEFVNPFCRLSYAPKPPQVAASITNHKAYFAQAVLVRGPGNVPDTLRAVPPEIVNYLQTIQQ

***Caenorhabditis briggsae* XPO-2**

MEQIGAALQQTLEPNAAIRKQGEDALRTLQATPGYIIQILQLVVNEEQQVAPQIRMAAAVALKNFVKRNWVREKAIHGPAPEVEMSQEDEEQFRNMLLEAMFNTKANIQDILSNALYLIAQRDFPEKWPELVPYLSRFLSGDDLNHLIASLTSMDQIFRKFRYSSKSTELWRELLKCLQSTQEPLTMLLAKMMEVGQQKDQLGDEMMSQWLKVLNLIAKVYHSLCVQEIPEYFEDHLNDWMPHFLLLVSIDVPSQTSSGGEPTTLDELKHEICEIFALYSQKYEEEIAKFVPDIISAVWHLLEKTGPDTRYDTMVCAALEFLSMVSQRQYYESHFTGEGVLKTLAENVCVQNLLLRQQDMELFEDEPLDYMKRDIEGTDVGTRRRGAIDLARGLCRRFEDKMLPCLGEIAQNLLASGEWIKVDIAYSLITAVAVKSETAKNGVTATNPLVDINDFFIGHVATHLNSDVNQTPILKADALKFAVTFRKQLAPEHLMTAIKASDALLSSATPILHKYAAYAIERILLSDSQNAQKVFSAHNLPVASILQNLVAAFDKDPKAQNSPYLIKAVLRIIVILDDETIRHADAIAKKLAQLIESATKNPADSVHTHFLFETICVLVTKTRTIGASLDAQLLPLIEVIFREDLEDLIPYALQITGVLVSSCIARNASIDQFAAFLPFLLSERLWARSANVPAALSVLEVLMSVNGQQVVAGNSNLILNHLTRLLNSKTLDQYGFQLASAILPSIEHFEGDAMKHLLTSMFNRVQSSKTSKFMKLFVVFLCRFTIIRGAQDLVKSCESMQTGMFGLLIEKVVCLEMPALKHTTTATEKRIIAIGMGNLLAEATQQLINHYGILTHETAMLLDAAAASDRAIMSPEEEQASMYNAEGEFVNPFCRLSYAPKPKPIADQITNHKAYFAQAALVRGPGNVPDTLKTVPPEIASYLRSLHNV

***Caenorhabditis japonica* XPO-2**

MEQIGLALQETLEPDANVRRHGEDSLRTLQTSPGFIIQILQLAISEQQQIAPQIRMAAAVALKNFVKRNWGPAPEVEMSQEDEQHFRDMLLEAMFNTRGNVQEILSNALYLIAQRDFPEKWPQVVPYLSKFLTGSDLNHLVASLTSMDQIFRKFRYQSKSTELWKELLKCLQSTQEPLTLLLRNMMEVGQRKDQLSAEEVNQWLKVLNFISKVYHSLCSQDIPEYFEDHLNDWMPCFLHLVQIDAPTQTSSAGEPTILDELKTEICEIFTLYSQRYEEEIAPFVPDIISAVWRLLEVTGPDTRYDTMVCAALEFLSTVSQRQYYESHFTGEGVLKTLAENVCVQNLLLRQQDIELFEDEPLDYMKRDIEGTDVGTRRRGAIDLARGLCHRFEAQMLPCLGEIVQTLLASGDWIKTDIVYSLITAIAVRTETAKAGVTATNPLININDFFISQVATHLNSDINQTPILKADSLKFAVTFRKQLAPEHLMTAIKAADALLSSNTPILHKYAAYAIEKILLADTAKVFSAHNLPVASILQNLVSAFDKDSKAQNSPYLIKAILRIIVILDDGTIRHADAIANKLSQLVESATKNPADSVHTHFLFETICVLVTKTKTIGASLDAQLLPLIEVIFREDIEDLIPYALQITGVLVSSCIARNASIDQFSAFLPILLSERLWARSANVPAALSVLEVLLSVNAQHVVASNSNLILQHLSKLLGSKTLDQYGFQLAAAILPSIEHFEGNAMTFLLNTMFRRVQSSKTPKFIKLFIVFLCRFTISRSAQDLVRSCENIQTGMFGMLIEKVVCIELPGLKQTTTLPEKRIIAIGMANMLAEATQHLSGQFGILSYEVAMILEAASASDRTVLSPEEEQAAMYNAEGEFVNPFCRLSYAPKPTPVAPNISNHKAYFAQAVLGRGPENVPDVLRAVPPEIVTYLNSIQP

***Caenorhabditis remanei* XPO-2**

MEQIGVALQETLQPNAATRKHGEDSLRTLQANPGYVIQILQLAVNEQQNVAPEIRMAAAVALKNFVKRNWGQAPEVEMSQEDEEQFRNMLLDAMFNTTKSNVQEILSNALYLIAQRDFPEKWPELVPYLSKFLTANDLNHLVASLASMDQIFRKFRYESKSTELWKELSKCLQSTQEPLTLLLANMMEVAQRKDSLGAEETAQWLKVLNLIAKVYHSLCSQDIPEYFEDHLNDWMPHFLQLVQISAPSQTSASGEPTTLDELKHEICEIFTLYSQRYEEEIAPYVPDIISAVWRLLETTGPDTRYDTMVCAALEFLSMVSQRQYYESHFTGDGVLKTLAENVCVQNLLLRQQDMELFEDEPLDYMKRDIEGTDVGTRRRGAIDLARGLCRRFEDKMLPCLSEIVQTLLASGEWIKIDIVYSLVTAIAVKTETAKAGVTATNPLIDINDFFIGQVAGHLNSDVNQTPILKADALKFAVTFRKQLAPEHLMTAIKASDALLSSSTPILHKYAAYAIERILIADNSKIFSAHNLPVSSILQNLVAAFDKDPKAQNSPYLIKAILRIIVILDEETIRHADSIATKLAQLIESATKNAADSVHTHFLFETICVLVTKTRTIGASLDAQLLPLIEVIFREDLEDLIPYALQITGVLVSSCISRNASIDQFSAFLPFLLSERLWARSANVPAALSVLEVLLSVNARGVVADNSALILNHLSRLLGSKTLDQYGFQLASAILPSIEHFEGQAMTFVLNTMFRRIQSSKTPKFIKLFIVFLCRFTILRSAQDLVRSCENIQTGMFGMLIERILCIEMPGLKQTTTAPEKRIIAIGMGNLLAEATQQLV

***Haemonchus contortus* XPO-2 (Likely exon-intron boundary issue with predicted protein)**

HCFLIGRDGASRATNCVETFVLGGSSDKESC*CSSHTFLV*DYVCAHQEDGVENPMTVLLNNMFRRVQFSKTPKFMKHFVVFLCRFAIVRGAEHLAKVREFFGRLIHYAVSLEEVQASMYNAEGEFTNPYCRLSYAPRADPLVPEITNYKSKFQLVQSLCSDGDWTKLDVVYCLVTAIASKTETAKSGVTSTSQLVLMCSGVLLSSSLTRSQTVDQKYISFLPYLLSTELWARSANVPAALTVVEVSCLVSRSWLENSLLTARVVPVGSLLNNLVAGFDKDPKAQNSPYLIKAILRCVAILDRSIESGLFQSVEAIQTGMFRMLLEKVIVAELANLQNMTTLDDKRTIAIGVANMLADATNYVG*VFVVIFELISEDVCVKNLHLRQEDLEMFEDEPIEYMKKDIEGSRFDGMVCAALEFLSIICQKNHYESYFVGEGVLQTIAREFSHCFHGLLGTDTLTRRRGAIDLVRALCRKFEERLVPVLAQVLSLAVFPLR*LKIWIIFQLVQSLCSDGDWTKLDVNLDAELMPLIETILSQDIADLIPYALQITGICRPRCQYGPLAVGTAQLIEAPSASDRPLLSPEEEQVGSYSSRLVGSKSLDQYGFQMANAILPVVEPLQINVADYYAGQVRGHLSTNTDDMPIIKADALRYSYSDYLARAVLQRGPAANSAVEACIPAELRTHLKYDTVSCGLLFFVLLIRDWSEFILYFSGEIYLLKKCSHFMNQNFIVQQVTLALQGTLQLDPNVRKQAEARIWEFEKVSG

***Meloidogyne hapla* XPO-2**

MSNQVTTEQIVGLLQATLSPESVKQAQEGLSQQASVPGYARVLLRITCDANYSNHIRNIAAISLKNFVRMNWSSDGDIAIAEDEREELRNVLLQVMFQIPQFLRSLVTEIVCQVSRIDFPERWPRLVELLAENLQKATDFDQLVVSLGTLEQMVCRYRHEMRSDSLWKEIIFVVQNVAEPLTQLFSKMQFYLPDQERGAQLSPTDRISWLQIVLELTRIYHSLISQDLPEYFEDNLTPWMEGFLQMLSLKLPEQSAQITTEPTEHDKLCTEICEIATLFSQRFEECFVPYTQRFMGTVWNLLVFADGKIRFDALVNSALGFLAAICQRPQYVPYFRDEGVLKAICENVIVKNLTLRPEDFEIFENEPFEFLKRDIEGSDLETRRRGAVEFVRALCKHFEKELCVLLSNVIQDFLEKYKQNPAQNFVFKDLVYFLVSALASKGSTSRSGATDTRQLIDLNHFYQQNVRPDLIFGSINELPILRCDALKYLVLFRNQLSTEQIIECFLGENCQFETSIFRFLSSNHFILHHYVAYAIERLILMRIPNSKDLLFTASNFQLALAIERLFDCLNSPQGYETHYIMKALMRLFVVMDDGLSRSSAHIYLDKLSQIVADAIRVPKNPVLLHFLFESICVIIRKAYVKVEGGVDKYIIPMVESIIQNDVAEFKPYAFQLIALLLDQCQQEREKNVAVSQDAYIAFFPSLLRPDFWSRSANVPALILVFESFIRCLPQLPFGSEYSDQVLAIFQRLIASKAYDQHGFRLANALLPHLDTYERMTSSAIFLAMLNRMHQNKTAKFSKQFTIFIFRYSAIKGGLALANSLEQIQNGIYNMIVERILLAELKGMPQTTTYDEKRIIVIGAARLISETIQVLGNNYALIIEAIVNLLEAFEHKPKPVDEPVPEDGEVNEMEYNDPYCKLMNAQHNEPFAAEVINIKKHFAQAVFMATQSNPESLGCLNARLLSCLRAYSTMV

***Meloidogyne incognita* XPO-2**

MSNQVATEQIVGLLQATLSPESVKQAHDGLTQQAAIPGYARVLLKILCDANYPNHIRNIAAVSLKNFVKMNWSGEGDIPISDDEREELRNALLEIMFQVPQFLRLLVTEIVCQVSKYDFPERWPRLVQLLAENLSKATDFDQLVVSLGTLEQMVSRYRHEMRSDSLWREIIFVVQNVAEPLTQLFSKMQFYLPDQEGGTQLSPTDRISWLQIVLELTRIYHSLISQDLPEYFEDNLTPWMEGFLQMLSLKLPEQNSQLTTDPTEHDKLCTEICEIATLFSQRFEDCFVPYTQRFMGTVWNLLVFADAKIRFDALVNSALGFLAAICQRPQYVPYFRDEGV LKAICDNVIVKNLTLRPEDFEIFENEPFEFLKRDIEGSDLETRRRGAFEFVRALCKHFDNELCALLSNVIQDFLEKYKQNPGQNFVYKDLVYFLVSALASKGSTTRSGATETRQLIDLNHFYQQNVRPDLLFGSINELPILRCDALKYLVLFRNQLSTDQIIECFLGENCQFETSIFRLLSSNHFILHHYVAYAIERLILMRDLLFTASNFQLSLVIDRLFNCLNSPQGYETHYIMKALMRLFVVMDDELSRSSAHIYLGKLSQIVADAIRVPKNPVLVHFLFESICVIIRKAYVKVEGGVDKYIIPMVESIIQNDVAEFKPYAFQLIALLLDQCQQERE KNVTVSQDAYIAFFPSLLRPDFWARSANVPALILVFESFIRCLPQLPFGSEYSDQVLAIFQRLIASKAYDQQGFRLANALLPHLDTYEKMTSSAVFLAMLNRMHQNKTAKFSKQFTIFIFRYSAIKGGLALANSLEQIQTGIYNMIVERILLAELKGMPQTTTYDEKRIIVIGAARLISETIQVLGNNYSLLIEVIVNLLEAFEHKPKSLDTEVPEDGEVNDMEYNDPYCKLMNAQHNEPFAAEVINIKKHFAQAVFMATQSNPESLGCLNARLLSCLRAYSAMI

***Oesophagostomum dentatum* XPO-2**

ENPADAVHTHFLFETMCVLIRKTESLPDGGLDGELMPLIETILSQDIADLIPYALQITGVLLSSSFARSQNVDQKYISFLPYLLSTELWARSANVPAALSVLETFLKRCPEAVMKNHGALVMQHFSRLVGSKSLDQYGFQLANAILPVVEMTQGVENPMTVLLNNMFRRVQFSKTPKFMKHFVVFLCRFAVVRGAELLAKSVEAIQAGMFRMLLEKVIVTELSGLQNMTTAEDKRTIAIGIANILADATNYVGDQYGALAVGVAQLVEAPSASDRPVLSPEEEQASMYNAEGEFTNPYCRLSYAPRPDPLVPEITNFKNYFARAVLQRGPAANSAVEACIPAELRTHLMAYA

***Pristionchus pacificus* XPO-2**

MLLAQADTINTHDRMNTAEVAEVLRNTLQTDAATRKEAERQLARVQSHIGYGMALLELLSSPQVPAEIRVVAAVVLKNFVKTNWGENPEVEVGDVEQDQLRTALLAAMFANTGNLQKQLSHAVFLMAKKDFPERWPELINGLASQMNTANADLERLLAALNTMDQLLEKYRYESKSESLWRELKMCLLAVQAPLTTLYEWLVGFVDNLAQLSAEQANSLFELLNCVMRVFHSLCTQDLPEFFEDNLSRWVAGMAKLFTIEAPSVQSAGGEATPLDKVKTEMCEIVTLYAQRYEEEIMPHMQGLIGAIWQLLVNTNSETRYDGMVCSALDFLSAICVRSQYKDMFKADGVLKTLAEDVAVKNLMLRQEDLEQFEDEPLEYIKKDLEGTDSGTRRKGAVDLVRALCREYEGDLMPILSAVVSSFIADASDQFWRKRDVVYCLVRAMASKTETSRQGATSTSQLFSSSSLVSHAHYLKSEGGVVGCIMFDGDRVFRLLNHVPPPSKINIVDYYSSNVRGDLCAADVNSFPILKADALKFVVLFRNQLPPEAHQEALVAIENLFTSQHTIVHKYAAYAVERLLLVRVNNTPIFTAASVNVASLLARLIAAFDADPKAQNSAYLIKALMRVVCIIDASTARQAGEIATRLAAMVDAAVKNPADPSHTHFLFETICLLVKKTSGHVEGGIDRPLFPLLETILAQDVADLVPYALQGNQCESDIPLPSAGNNTLAEY

***Trichinella spiralis* XPO-2**

MGVCGNYWAEFTVGTHNEMTDLHKCNNDFLTLKKTVEELFSVDAEKRKKAESVLQESLKCPGSIGMIIVAVICCCLFLIISLVRSVSARQLAAISLKNFIKSSWISDLEGSTQIGEEDKIYIRDSIVGAMVNSSPLVKKQLTEAICFIGKYDFPSNWKSLLDALVKCIESGDLSIVNSALVTAEQLFRRYSTESKSEKLWREIKYVLDNFADPLTKLFTSLTSKVSGEEMKHFDNGCTMQIYETFVDTVKIFYHLNFQDLPEYFEDHLDEWMGGFKVLLELKNVYSCPEIGNLKMSFCAQICDNLAMFAEKYEEEFFNHVMNFVKIVSQQLLSVSAEEKYDEFLSKGIDFLATVCGKPPYKLLFENGEILSQISECIVLPSLELRACDVDNFENSPNDYVLFDLEGSVAESRRRSACNFISAVCKQFSDTVEPMFTLHLHNLLVQYSEDPAENWSRKSIAINLLLAICCRGTTQKATISPILNVNEFFLTQLKLVFESALGNALLKADILKFLILFRTEIPKPTFVNILPTVRNLLCHDSGPVAVLELSDVTGSYKIIFENLLKALELPDTVHCEYVMRCFMRLIEAIFNLGADAVREYFNTIAMKIYSLITSPGPPMFNHLLCESMCLLIRLCGPTDNFNAEDILFPIFQQILQSESNYLLPWVFQMLALLLNRRTGEAQIPPVYMVLLPHLLNPEVWSNPVNLPSVTHLLTVYMRVNSGELSKEDYLIKVLTIFQRLVFSKSFDENGMRLMNAFIDYGLRNHVDMYLDDILRVVFKRQQENQTYKFSRMFVILICHMVVRFGAMAALARIENIQNGLFGNIVEKLFIAKSYTFKRSEDAMIFIYSVLQLLYCCIEFKINGVYSKYTVDLLQMVHASFHKHSEIIFVSTEGVHNAIDEDMVSNVLYHADVIEFHIPGTENFAKLYTHAIGQMLRDAALKDAVESFLSRLTEQERELLRMMSLR

**DCR-1**

***Ancylostoma caninum* DCR-1**

PRLLLTALTTSNANDGINLERLETIGDSFLKYSVTDYLYHSHPDQHEGKLSFARSKEVSNCNLYRLGKRLGIPSLIVASKFDVYDSWLPPCYMPNNDFKAPNSADAEERDKFIEDVLEGNETAQKIPKXXRDGTRPTXQRCSATREWCGNHQLRKAICQQCSSRGTSAIAYNMLTQQYISDKSIADAIEALIGAXFDSGASTNVEGHEMAWPEGLTDEVTAIEPLLEFVNTPECPDMAQRLLKDMWQQFNFSLLEXKLVPFXQQSLPPASVHPRXLLQEQNYGLLSASRIPGXCVLDYMITRYLFEDERQYSPGVLTDLRSALVNNTIFASLAVKYDFHKHLXLCALVSIIXLRNFVKLCSERNFFDANFNSEMYMVTTEEEIDEGQEEDIEVPKAMSDIFESVAGAVY

***Ascaris suum* DCR-1**

MGCAEEISPLPYNMLTQQYLGDKSIADAVEALIGAHLLELGPTATLKFMKWVGLKVLTEPVTLDPPLLRFIDTPQEPELAMCKLNEFWVQFQFSKLEDSICYRFKDRAYLLQAFTHASYYKNRITGCYQLIFIYGAVVYYLD

***Brugia malayi* DCR-1**

MVVRTSDVDKNFFTPRDYQVELLDKACKRNIIVPLGTGSGKTFIAVLLIKEYTTKLVTPWKNGGKRAFFLVDKVSLVEQQAAHIEHHTTLNVGKMHGHLNQDIWSEPAKFDTFIALHEVTVLTAQIFLDLLDHGFFNMSNAAVIIFDECHHVLGSKHPYRLIMHRYGQLTEVDRPRILGLTASLISSKIPPSNLEHLLEKLERIMHSSIETASDLVSISKYGARPKEYVIMCHDFFCCTCETSKKVISTLESLRTFCLKCTEFHPEFDVDPRKPVLEAVSRTKSVLEQLGPWCAWKLCQLFQRQLKKQSGQGFLPEKQIIFLQMAYTTMRFIKRLLDVKVANIRCFSDVKPILPDRLARLFEILKFFSPSNMEKVDPDFTFCGIIFVEQRYVAYVLNTLIRAISRWDNDKFGYLVSDFVIGYNSANIGTEETMALHKRQELVLRKFRQRHLNLLIATSVLEEGVDVRQCNVVIRFDRPTDYRAYVQSKGRARKDGASYFLLVEERDREQCSCDLKDFLQIERIGDRVMYRAELLLPINSPIKETIKLKKPLESKKLAQMAVALEACRRLHKRKELNDYLLPVGKDTIMLTALDEDPDEFIPNMSYKVGSARRRQLYDKRMAKALHNAIPRAGEECYIYVMEMDLIKAVTGAANPKNRRIINPLDTEFCFGFLSNKKIPKVPSFPLFLRQGRMQANIFLVKSRLLVDTQMLELLKAFHHYLFDNVLRLVKGGLVFVPDKAPVNVLIVPLRRERNSETSEVDFKLDYAYVRNVVSSIDELPRIPTEAERLAFKFDAAKFQDAIVMPWYRDRDHPSFYYVAEIIDAKPSSKFPDDKFVTFNDYFIQKYNIIIYDQQQPLLDVDYTSSRLNLLMPRHWSRSKSRVTEEKSSESGGISQGQILVPELVDVHPIAASLWNVIAALPTLLYRINSLLLADELRELVMREAFSNPNYNTSDDVYWLPLDYPTPMDDLEMKSVQKICDLKKKHVEQKSQENKEMEAKDSGAEMTDFEIGVWDPELAKGL

DDFCLNRERHDEIKGFEDIDRDALGLMNGSALRQHGDMSDDDEDDAVVLFDFINSVHERLGKESGDIFAPRENITSSGWDDLIVIEESAPNGINMPLSVNSGDSHIDSRGLMADLSRMSWLLDLPTVLPSTAVDTANQNDFGIKKETVKLSNKRHPSVARKPAQLYLDSLERLEDSDRGSSKSNRQEECIDLIDFCDEVEEILSNVYDGTAPFDLRYFNGCLLDTDVELDTAEVLSPKKISMRQDRKLINEEMVRSSSDLELFLQISHDNDVIKLVSSTTDTNAVDRKQEISAPVLDWMTFSFEEDTFTDHPDGVSPCTLLQALTLSNASDGINLERLETVGDSFLKYAVTDYLFHTNPEQHEGKLSFARSKEVSNCNLYRLGRKHNLPSLIIGSKFDPNDGWLPPCYAPTSDFKAPNTLDAEERDKFIENVLEGKAVEGQETVKIPTGWDEADRNGQVRRIANGIETIEFPKNMTTSWDGEEITPLPYNLLTQQSLGDKSIADAVESLIGAHLLELGPTATLKFMKWLGLKVLTXPVQMEPPLLRFIDTTDQPDKSLRKLNDLWIQFQFSKLEDCIGYRFHDRAYLLQAFTHASYYKNRITGCYQRLEFLGDAVLDYVITRFLFQHSAHYSPGVLTDLRSALVNNTIFASLAVKYNFHKHFIAMCPRLHHMIEKFVCLCAEKNLSSANFNEEMYMVTTEEEIDEGEEEDIEVPKAMGDIFESVAGAIYLDSGRSLNTVWRVFYNLMKETINECCSNPPRSPIRELLEMEPERARFSKLERILETGKVRVTVDIQGKCRFTGMGRSYRIAKCTAAKRALRYLRSLKKEKERVAGKE

***Caenorhabditis brenneri* DCR-1**

MVRMRPDLQCFNPRDYQVELLDKASKKNTIVQLGTGSGKTFIAVLLLKEYGVQMFAPLDQGGKRAFFIVEKVNLVEQQAKHIEVHTSFKVGQVHGQTSSELWKSPVACEKFMKENHVVVITAQCLLDFINHAFIKIQDTCVLIFDECHHALGSKHPYRLIMTKYKELKKAGEPVPRVLGLTASLIKEKVAPEKLSEQLVKLESVLDSVIETASDLVSLTKYGAKPYEAIVLCRDFETDKLGVPNYDKIMNLLYETDKFIRETTLFHPDLDMDPRSYAREALKATKSVFQQLGPWAAWKISQMFEKDLMKHINLQQLPEKATLFLNLAKTTIITVKRLLEPEMRQIKSLNELERYVPHRIIRLFQVFEMFNPDFQKQRMALEKTEHLSAIIFVNQRYIAYSLHIMMRAIRQWEPKFKFLNTDFVVGASGPNMANSNNQGLQKRQTDALRKFHRGDLNVLIATSVLEEGVDVKQCNVVIKFDRPTDMRSYVQSKGRARKAGSRYVVLVDQKDTSACDSDLKDFQQIEKILLSRHRTVNNPTEDDSLTDLGDVDDLMAPYVVEKTGATIKMSNAIALVNRYCAKLPSDIFTRLVPNSTIIPVEDHGVTKYCAELLLPINSPIKHAIILKDPMPNKKAAQMAVALEACRQLHLKGELDDNLLPKGRESIAKLLEQMDDEPDEYAPGMAAKVGSSKRKQLYDKKIARALNESHVEPEKECYIYALELERLRDSEPSPNTRRRRFEDPKEYEYCFGFLSTKEIPKIPPFPLFLRQGNMKVRLTLAPQKTTVTKEQLEEIQYFHNYLFTQVLQMCKTGNLEFDVTANAPLNTLIVPLNKSKEEGYSINMKYVSGVVANMENMPRVPTDEVRRKFKFDPENYKDAVVMPWYRNMEQPAFYYVAEILTDLRPSSQFPDHNFTTFNEYFIKKYNLEIYDQEQSLLDVDFTSNRLNLLLPRIQSQARRARSFSGSSTSSAPPTPSESRESGPHPSQRQILIPELMDVHPISATLWNVIAALPSIFYRINQLLLSDELRETILVKAFGKGSEETKLKDSFEWSCMTYTATYEEKQSVIVKKIQQLRDLNQKKLDAQQEKEKKEETKTDGEEWTIGVWDPEDAVRSGVDVDMFNNKENMDGEDQETVGLSQGLHDGNISDEDDDLPFVMHDYTARLNSHNLNGNGNGIQQASWEDTVEIVPSGWGDLDGVGEVDAIPFQIIGGSGGLNVQALMEDVGRVFDPKTPEKSDPAVPSATTTAASASSTSTASSTVSKLTKEELQRKKIQEEMLAKAQERLEALEISEEKEKPRRLEETVDLDEFGDDLPLEEEENQIARAKTMDEEIEELKRGALEKHKIDNDTVKTDASSREKCEVLPIAAQDLQPRPFSFEKESQTMHGRLLKERDQEAISHVDSDVGMGVSPCLLLTALTTSNAADGMSLERFETIGDSFLKFATTDYLYHTLQDQHEGKLSFARSKEVSNCNLYRLGKKLGIPQLIIANKFDAHDSWLPPCYIPTCNFKAPNTDDAEEKDIEMERLLNGETIEEKPQNKTGWDMELKDEAKTTTEKIETITFPKRTALVNEDISPLPYNLLTQQNISDKSIADAMEALIGVHLLTLGPNPTLKVMSWMGLKVIQKDAVTDVAAPLLRFIDTPTNPNASTIQLDNLWQQFQFAKLEEKIGYRFKERAYLVQAFTHASYSNNRVTGCYQRLEFLGDAVLDYMITRFLFEDVRQYSPGVLTDLRSALVNNTIFASLAVKYEFQKHFIAMCPGLHHMIEKFVQLCADRNFDTNFNAEMYMVTTEEEIDEGHEEDVEVPKALGDVFESVAGAIYLDSGRNLDTTWQVLFHMMRSTIESCCANPPRSPIRELMELESSKARFSKMERILETGKVRVTVDVGNNMRFTGMGRNYRIAKATAAKRALRYLHMIEVQRRQSPSDSPTQ

***Caenorhabditis briggsae* DCR-1**

MVRMRPDLQCFNPRDYQVSRGIWIFGLKDNIFQVELLDKASKKNTIVQLGTGSGKTFIAVLLLKEYGVQMFAPLENGGKRAFFVVEKVNLVEQQAAHIEVHTSFKVGQVHGQTSNELWKSTETCDEFMRQNHVVVITAQCLLDLINHAYVRLQDTCVLIFDECHHALGSKHPYRLIMSKYKELKKAGMPVPRVLGLTASLIKEKVAPDKLSEQLNKLENVLDSVIETASDLVSLSKYGARPFEAIILCRNFEADQLRLRNYETIRDLLKDTEDFVNQTSVFHPDLDLDPRRSIRDSLKTTKAVLRQLGPWAAWKTTQMWEKELSKLTKTNILPEKALTFLNLARTTMITIKRLLEPEMREVRSLADLQKFVPHRFVRIFEILEMFQPGFQTERLRKEKPENLSAIIFVDQRYIAYSLHIMIKAIRSWEPKFKFLNSDYVVGASGQNLANSDNQGLHKRQTDTLRRFHKSEINVLIATSVLEEGVDVKQCNVVIKFDRPLDMRSYVQSKGRARKPGSTYVVLVDQKDVTACDDDLKNFQQIEKVFKAIFGQKRAKFRCSWGIFDSKSPIFHPKILLSRHRTVNNPTEDDSGDFNLDDVDLLMAPYVVESTGAELKLSNAIALVNRYCSKLPSDIFTRLVPHSRIIPVEDRGVTKYCAELLLPINSPIKHAIILKDAMPNKKIAQMAVALEACRQLHLKGELDDNLLPKGRESIAKLLEHIDDEPDEYAPGMTAKVGSSKRKQLYDKKIARALNESLIEPDKDCYIYAFELECFREPEPVANPKRRKFQNPTEYEYCFGFLSTKDIPKIPPFPLFLRQGNMEVRLTVAPEKTRVTEEQLEQIQYFHNYLFTQVLQMCKTGNLVFDATVNAPLNTLIVPLNKSKEGTYSINMKYVTEVVANMENMPRVPTEDVRKGFKFNADAYKDAIVMPWYRNVEQPVFYYVADILTDLRPSSQFPDSNFRTFNEYFIKKYHLEIYDQDQSLLDVDFTSNFFSSSVNADSGSGAAAGAGVSVIGANDAPGAPGGPTGSNTRPTRLNLLLPRSQPQPRRARSNSASSTTSNPVTPSESRESQASGGHHSSQRQILVPELMDVHPISATLWNVISALPSIFYRLNQLLLSDELREIILQKAFGIQTSRLQNSLEWSSLAYPTAYEEKQSIIVKRIQQLRDLNQKALEENEKGPEEKKGKKKVEDKEEEFAFTIGVWDPEEAVKIGVDMTSTMRAEEDQETIGLTQGLHDGEMSDEDDDLPFVMHDYTARLTAANNKGLSNPQWEDVVEIVPTGWGDDDGPGSGGPDDNELPFNSVNSQNMKFQQIIGGTTGGLNMQALMADVGRVFDPVGPPGAPGASLAPMTETPAPAAAPLPESALTEEEKKLKKIQEELLEKAKERLEAMEMSEEREKPRRIEETVDLDEFAEKDAVEEEEDEALDFPRTMDDEIEELNLGAQRKQDLDDTTVKTDASDRSTCQVLPTAAMDVPPRPFSFEKESQTMHGRLLKEREKEIVSHTDEDVGMGVSPCLLLTALTTSNASDGMSLERFETIGDSFLKFATTDYLYHTLQEQHEGKLSWARSKEVSNCNLYRLGKKLGIPQLIVANKFDAHDSWLPPCYVPTCDFKAPNTSDAEEKDKEMERILSGQTIEEKPENKTGWDLGQDEAKKTVDGIETITFQKQTRILNEDITPLPYNLLTQQNISDKAIADAMEALIGVHLLTLGPNPTLKVMNWMGLKVIQKDQATDVQPPLLRFIDTPINPDASTKALDNLWQQFQFAQLEEKIGYRFKDRAYLVQAFTHASYINNRVTGCYQRLEFLGDAVLDYMITRYLFEDVRQYSPGVLTDLRSALVNNTIFASLAVKFEFQKHFIAMCPGLHHMIEKFVKLCGDRSFDTNFNTEMYMVTTEEEIDEGHEEDVEVPKALGDVFESVAGAIYLDSGRNLDTTWQVIYHLMKGTIETCCANPPRSPIRELMELEGTKARFSKMERILESGKVRVTVDVGNNMRFTGMGRNYRIAKATAAKRALKYLHQMEEQRRLALTTTSQA

***Caenorhabditis japonica* DCR-1**

MRASVQRPTIEHLLKRGMRSSDVVRTLGISDSTVRNISAALKKYGSSSERSKTRRPQTVNTRRIRGVIKRRIDRIDGLSLNNVAGDLKIGGRTAQRILKDDLKVDSYKLARGQYLSDTSKPNRIDKAKKLLAHFRVRRVSDVIWSDEKIFTIEPLPNRQNQRHLLCKGDNKSPKRRQVSNRLFPKSVMVWTGVTSTGKTLLVFIDRNVKIDAEHFIGRPFILQQDWALSHGAKSTKVALDTHFPGYLGKDLWPARSPDLNPIDFSVRLNLLLPRLPPHQQRRVRSLSNSSNTSTKAATSSESKDSDVGQTSHSSQRQILVPELMDIHPISATLWNVIAALPSVFYRLNQLLLTDELRETILVKAFGRERDAAQLDPNFEWSPLAYASIYEEKQSIIVKKIQQLRELNKKSVEEQEVKKKVVAPEDDGSFAIGVWDPQEAIRSGVDITRSEMKNEGAAEDMDTVGLNQGLHDGNISDEDEDGLPFVMHDYTARLTSQRQGAGEVELWPGCDQIVPTGWGDDGIGSIPGETFHDVNEQKLPFEILGGSSIGGLNMQALMADVGRVFGPMGSSSTPNVETTIPENPKNSKASVSSNELAAKEQENLRKIQEEMLAKARERVECMEMSEEREKPKRQEEFVDLEQFTDELSSKSDEEQDENDTIRIATLQIRTLGLHDGNISDEDEDGLPFVMHDYTARLTSQRQGAGEVELWPGCDQIVPTGWGDDGIGSIPGETFHDVNEQKLPFEILGGSSIGGLNMQALMADVGRVFGPMGSSSTPSVETTIPENPKNSKASVSSNELAAKEQENLRKIQEEMLAKARERVECMEMSEEREKPKRQEEFVDLEQFTDELSIKSDEEQDEDNISFRPRTMDEEIEELTLGASKKQEMDDITVKSDMCDRETCLVLPVAATDLPSRPFSFAKESKTMHGLMRLLKDNDEENHVSHVDADVGMGVSPCLLLTALTTSNAADGMSLERFETIVGRVCSAPPTFQILDTVRLGKKLGIPQLIVANKFDAHDSWLPPCYIPTSTFKAPNTEDAEERDNEMERILNGQQIEDNISFRPRTMDEEIEELTLGASKKQEMDDITVKSDMCDRETCLVLPVAATDLPSRPFSFAKESKTMHGLMRLLKDNDEENHVSHVDADVGMGVSPCLLLTALTTSNAADGMSLERFETIGDSFLKYAVTDYLYHTLLDQHEGKLSFARSKEVSNCNLYRLGKKLGIPQLIVANKFDAHDSWLPPCYIPTSTFKAPNTEDAEERDNEMERILNGQQIEVKEEQKTGWDIGGDNLKSTADGIETINFPKQTRQIPDDISPLPYNLLTQQNISDKAIADAMEALIGVHLLTLGPNPTLKVMSWMGLKVLEKDAKTDTPPPLLRFIDSPINPNASVNALNNLWQQFQFAQLEEKIGYRFKDRAYLVQAFTHASYINNRVTGCYQRLEFLGDAVLDYMITRYLFEDVRQYSPGVLTDLRSALVNNTIFASLAVKYEFQKRVLFCLKTSEYGSSYFKYSCAMHRVENTKNCFFLYRFKDRAYLVQAFTHASYINNRVTGCYQRLEFLGDAVLDYMITRYLFEDVRQYSPGVLTDLRSALVNNTIFASLAVKYEFQKVSILKKSSSEM

***Caenorhabditis remanei* DCR-1**

MVRMRPDLQCFNPRDYQVELLDKASKKNTIVQLGTGSGKTFIAVLLLKEYGVQMFAPYGQGGKRAFFIVEKVNLVEQQAKHIEVHTSFKVGQIHGMTSTELWKSPESCDEFMRQNHVVVITAQCLLDLINHAYVKLQDTCVLIFDECHHALGSKHPYRLIMIKYKELKKAGHPVPRVLGLTASLIKEKVAPEKLTEQLNKLESVLDSVIETASDLVTLSKYGAKPFEALVLCRDFETENLPLPHYETIMALLLDTEKFVNHTTVFHPDLDLDPRRSIRDSLKTTKAVLRQLGPWAAWKIAAMWEKELSKLTKSQILPDKALLFLNLAKTSMTTVKRLLEPEMRKVKSLADLEKFVPQRFVRLFESLEMFEPEFQMKRMNREVPEKLSAIVFVDQRYIAYALYVMIRNVRQWETKFKFVQSDYVVGASGQNLANSDNQGLHKRQTEALRRFHKNEINVLIATSVLEEGVDVKQCNLVIKFDRPLDMRSYVQSKGRARKMGSRYVVMVDHKDVPSCDSDLKDFQQIEKILLSRHRTVNNPTEDDSIDFNLDDVDHLMPPYVVESTGAELKLSNAIALVNRYCSKLPSDIFTRLVPHSRIIPVEDRGVTKYCAELLLPINSPIKHAIILKDPMPNKKAAQMAVALEACRQLHLKGELDDNLLPKGRESIAKLLEHIDDEPDEYAPGMALKVGSSKRKQLYDKKIARALNESRVEPEKECYIYALELERFREPDSILNPKGRVFQDPIEYDYCFGFLSTKDIPKIPPFPIFLRQGNMTVRLTSAPKKTSVTERQLEEIQHFHNYIFTQVLQMCKNGLEFDVSATAPLNTLIVPLNKCKDDEGTYSINMKYVTEVVANMENMPRVPTDDVRRKYKFDAENYKDAIVMPWYRNVEQPAFYYVAEILTQFNPSSQFPDTNFETFNEYFIKKYNLEIYDQNQALLDVDFTSNRLNLLLPRLQPHQRRQRRDSTSSVTSVTDRASESKSSESVTSSSGAHSTQRQILVPELMDIHPISATLWNVIAALPSIFYRLNQLLLSDELREIILQKAFSREDTKLKASLEWSPLTYPNAYEEKQSIIVKKIQQLRELNQKALEATQEKEKKEQILDEGKDTFAIGVWDPQDAAAIGVDISARDVMGTDGEDMDTVGLTQGLHDGNISDEEDDLPIVMHDYTARLTAANPIFGIPPQQPWEQEIEIVPSGWGDLEGVNPSPMPFQIFGGTNEVNIQGLMADVSRVFDPMAPIPGAPRPPPPTSISTSSGAPPTSTTSSNSATSQTDVPKKLTKEEEKLKKIQEELLAKTKERLEALETSDEREMPRRVEETVDLEEYGDDLIEDEEEEEYPQHRLKTMDEEIEELNSGAQNKQNIDDITVKTDVTDRQTCQVLEVAARDLPSRPFSFEKESQTMHGRLLKERENETVSHIDEDVGMGVSPCLLLTALTTSNAADGMSLERFETIGDSFLKFATTDYLYHTLQDQHEGKLSFARSKEVSNCNLYRLGKKLGIPQLIVANKFDAHDSWLPPCYVPTCDFKAPNNSDAEEKDKEIERILNGQTIEEKPEDKTGWDIGGDTAKSTADGIETINFPKQSRLLNEDISPLPYNLLTQQNISDKSIADAMEALIGVHLLTLGPNPTLKVMSWMGLKVIQKDAKTEVAHPLLRFIDTPANPDASLKALNNLWQQYQFANLEERIGYRFKERAYLVQAFTHASYINNRVTGCYQRLEFLGDAVLDYMITRYLFEDVRQYSPGVLTDLRSALVNNTIFASLAVKFEFQKHFIAMCPGLHHMIEKFVKLCADRNFDTNFNAEMYMVTTEEEIDEGHEEDVEVPKALGDVFESVAGAIYLDSGRNLDTTWQVLFHMMRSTIDSCCANPPRSPIRELMELEASKARFSKMERILESGKVRVTVDVGNNMRFTGMGRNYRIAKATAAKRALKYLHQMEEQRRAALAATSV

***Meloidogyne hapla* DCR-1 (Likely exon-intron boundary issue with predicted protein)**

EITI*YFYSIGATLYNKNNIRKWTNFVYC*IISPNQFSNKRANNI*TNGFKKIIFNDSRIGGL*NF*K*FKIF*KACKRLHQRKELNDQLLPAGKEIVLDLLGEVDDDEYLPYLPSKMGSSKKKRLYDRKV*KL*IS*FWENDTFLLDV*NPLFNITCTRK*MYSLCYGNETDQAR*RGTKSEKKKNY*SIRIKFCIWIFIFKGASKSRDLINLIHNIKEFF*CFRFPDFQSFNEMVK*LYKFVKLKINLFVSHLNFSNSFVSSISIYLKIFYALLVVVLFLRLVILQYHY*LFH*KNVCFNYFYKLFEISWNCRSGL*N*QRLPQLGYP*TSNYTF**N*KTIRL*RVELFKCCCFTMV*ENNYLIIFYRYRSEDQSAFYYVAEVKTFLQ***NFGFQIMTDQFPSSSFPDEKFTCFNQYFMSKYQLEIYNQKQNLLDVDHTSARMSKKIFNL*IQKSFKLLDIKIFRSFITKSNNRKISTSFIRPISTPNSCSRAATYSSSFSNSLVNYCYTSYNSL*VQFRFLLSLC*LKKN*NLRLNSLLLADEFRSKVLEDALKLGSQTPSDFEWTPLQYVTPNDDQNQKSIRNLDQLRKINQQEKENEVAMECDTVEEKGNETAASGIVG*FKYFLILENLIILILSKMFLILINIYRMISKSAFGIQHLTLTISWMSLLQLFIMLL*MD*SLDEGMDFEEL*LLGTKNSLKLLLLVMIQRFIIMEIFLMMMMLQLNMINLNF*CTIK*LV*FYFLTFKFST*ASDIGELGEMDVRPAGWNDDSNVNVIQIENESLPLVI*TYDNTKFSFKDNIYKQSSYQYCKFNE*LGEKLCCFWYINKHCKQ**S*YSRHSKCYYH**NGHPKERGLFNIYY*KYNKIYFSLISIH*MSLTRMIHQ*KGKMKIFYLRKFFGEWMKWKRILTMKVC*YTLFRQKMIIFYI*GTSKIIQTKSADSISPTRDLRDLEKQVIENI*IIILIYFRNLK*RHLNLTGCPSLLCGT*LIRIPMVFLLHYYFKVDCFNK

LLKIFLIILSINNFKCC*RNKLGKARDNWRFFPQNGCHQLFLP*TH*ATRRKAKLCSFKRSNLANVLIYF*KLILGFKLSFILSWSATRHSSSNRNIKI*STC*LASSLLCFYFRISCCQSI*LHRFR*RTRPT*SANGGC*N**NCRSTTKE**RNNSNRLGYS***QTEL*M*KWCGDINFSTSIFIFI*NLEILFLF*QTKSEIPDLPPMPYNMLTQQWISDKSIADAVEALIGAHLIQLGQSSTLKFMNWLGIKVIK*IINSKNKIISQVLTDISSLPSPLLRFIDTPEDVDK*FLFF**SHFLTA*SFAQTFGPFL*EI*LCYG*EQYWL*ICK*GSIYQGWR*SNKTRDSRDTRFSKKYENEIIRDLSKNTRFHETRDINNFY*AENY*KADGVLYLWLSGPIN*GV*DLANQKISIGFDLNL*ATQSNKLGRY*GWKG**RTEAGVTSGEINFKKFLWFLLPG*ILKFSGAEGDLT*NF*RRRRKFLILENRSGKI*KRSSQNPIYLRVYL*G*KYS*RLK*IEYISCLVAKYETRDTRPLKNTR*ETNATPGIYLFVFIVCLFI*AYLVQAFTHASYYNNRVTGCYQVFFNNFLLENELIF*RLEFLGDAVLDYMITRFLYEHKRQYSPGVLTDLRLII*FKFDLN*YCNFQLRSCQ*YNFCFFSSQIFFS*GFLLKKYFLNLFI*HFVMICPPLYQMVEKFVNFCKQKDFLHCANFDDEVNSY*NKFSKLLSF*KFLNF*IFMLTEEEIDEEDLVSEEDVEVPKAMGDIFESIAGAVYLDCGMDLDIVWRVFYNLMRDVIQKCCENPPQSPVRELFERKNCRAKFS*I*LLKKIFKKILENWNEN*KLAKLE*L*QLMIISNLLVWDVVIELLNVQLLNEHFNI*EN*MLQRIN

***Meloidogyne incognita* DCR-1**

MSPPRDFSGKCIPPRDYQVELLDRAKIQNTIISLGTGSGKTFVAVLLIKEYSQRLLHQNEKAVFLVNTVELVAQQAEHIEFHSSLSVARISGSTIKRKYERKEVEKITNTNQASHLFDFSSLALLIVDECHHCLGELHPYRLIMNHYKKLQGQRPRVLGLTASILNKKVPSSRIECTAQLLEQIMDSQIETSSNYTQICKYVTKPKQFIVCTKDDCTNERFVVDLLERLRSFIEKNEDFHSELEVDPRRKIFETISRSFSTLQQVGSWAALKGFILWQKNLLKHVDDPIIGNKQKCILRMAETAFRTCSKVLSHKINPLNSYDKLSNVNKSFISDRVRKLLEILKSYSPSKRELSGIKDTLFGLVFVKERFIAFMINNLLRFLVKQNPEEFGHLKVDFIVGHTGNSETGDEDRRLVNRKQEQTLTKFRNGQLNLLITTNVLEEGIDLRNCNLVVRFDPPMDFRSFIQSSGRARRENSAFYILIEEKNYLDFMMDLTGYAQAEEFVLRRYRSGNDFTLEGNDETKILHPHLDDAVAPYVVTTEKGTAKVSLSNAIQLVNRYCAKLPSDIFTRLVPRYTIQTLSENGQTLYIAELYLPINSPIKEPIKSKPMNSKRLSLMAVALEACKRLHQRKELNDNLLPAGKDILDDLLGEVDDDEYLPHLPSRMGSSKKKRLYDRKMSKTLNSTLPSQDSECILYVMEMKLVKPVTDEGNPKRRKIIDPFESNSAFGFLSSKELPKIPGFPVFQRNGEMNVQIRKVKNQQFRPTFELLQLIYLFHQHIFEDILRVARGGVVFAPGHSPIPLLIVPLKKLSNADLDYEIDKDYLNWDIRESPTTPSDEIRKQFSFEESNYLNAVVSPWYRSEDQSAFYYVAEIMTDQFPSSSFPDEKFTCFNQYFMSKYQLEIYNQKQNLLDVDHTSARMNLLLPRAITGKSATRSLDPSQRQILVPELVHIHPLSATLWSIIVTLPTILYRLNSLLLADEFRSKVLEDALKINSQTPDDFEWSPLQYVTLNDDLTQKSIRNLDQLRKMNKQEKENEVPMECENIEENVNETAASGINDFEIGVWDPSDGAQMSNNDNTLNEPSPVIHNAPVNGLIPGRRNGLRGVIAARDEELSEIIAVGNDTTLHNYGDISDDDDVAAEYDKFKFLMDNKMTTSDIGDLGEMDVRPAGWNDDSNANTISTNNPHINIASLMNDLEKNYAAFGTSSHISNNTRTNTPIISSDTAVDKTALQRKELNLDSLNAIDPNDQSVKRQNEDILPEEVFWGMDEAEIGENISSEETNKIIQTKSADSISPTRDLRDLEKQNFKIMAPELKWMPFSFMWNLLDQNPHGVSPALLLQALTTSSAADGINLERLETIGDSFLKMAVTNYFYYKHTEQHEGKLSYARSKEVSNSHLFYLGRQRGIPLLIETLKFDPHVNWLPPCYASTSEFHAVNPFDYTDLDEDQCQVPMEGVDTTETLDQQQKTNKETIATGWGTLDDDKQNYKRENGVETLTFPQPTKSEIPDLPPMPYNMLTQQWISDKSIADAVEALIGAHLIQLGQSATLKFMNWLGIKVLTDISSLPSPLLRFIDTPEDPNLSLKHLALLYEKFDFATVENNIGYKFANKAYLVQAFTHASYYNNRVTGCYQRLEFLGDAVLDYMITRFLYEHKKQYSPGLRSCQ

***Oesophagostomum dentatum* DCR-1**

PTLKVMKWLGLKVLTDDVVPVEPLLGFVNTPECPNMAQRLLQDMWQQFNFGLLEDRIGYRFNNKAYLLQAFTHASYFKNRITGCYQRLEFLGDAVLDYMITRYLFEDERQYSPGVLTDLRSALVNNTIFASLAVKYDFHKHFIAMCPGLHHMIEKFVKLCSERNFFDANFNSEMYMVTTEEEIDEGQEEDIEVPKAMSDIFESVAGAVYLDANRDLDVVWRVFFNLMRQTIEECCAYPPRSPIRELMELEPGKTRFSKMERIIESGKVRVTVDIGNKMKFTGMGRNYRIAKTTAAKRALKYLKSLEEQKLREAERNNGNENNCTNGN

***Pristionchus pacificus* DCR-1**

MILDGGEGSQINLNGLMADIEKVLPSTSPPSSPKEEVTTTAPGDTKYIPKQLLELWDGPVEVDDDREKGRKEEVIDLIAFDNDVEPFDSKSASPTAREEKKEGTIDDELSQLERGEEEKARRDHEAAMIVSEKEIKEGEVLASPLADPRFSFHHPSRSVAMGGYSLEEKRERWERRKLHLEYHPHSFLLHSRRAMLMMTIGDSFLKYTVTDFLFHSHPDLHEGKLSFARSKEVSNCNLYRLGKKIGLPSIIIGSKFDVQDGWLPPCYVPSINFKAPNNDDAEKKDEIMERMLNGEEMIKENIPVTGWDERPDSGIEDGVETINLTKPAPDALDDLSPLPY NLLTQQYISDKCIADSVEALIGAHLLSLGPQRTLQMMKWLGLKVMTEKIPIAPPLLRYIDTPLYPHRSEEMLTEFFAKFQLAQLEEKIGYRFNNKAYLLQAFTHASYYPNRITGCYQHFVAMCPGLHFMIEKFVKLCRERNFLDANFHCEMYMVTTEDEIDEGQEEDVEVPKAMGDIFESVAGAIYLDCGGDLDVVWRVFFNLMRETIEECCRNPPRSPVRELLEREHDKAKFSKLERIMEKGKVRVTVEIVSGKENFRFTGMGRNYRIAKTTAAKRALKHLKGIDEDRRKKAERKKQF

***Trichinella spiralis* DCR-1**

MNRLYDFETDFFTPRDHLTRLVDIGRRCNLIAPLGSAVDRLYVSVMLIREFSSMLRKPCSKNRRWCVYLVDRATSIKSVADRIRIYTNLNVGEIFCDIHLESEAKQYCAAQMEGNEIFVTTGTHFMKLLDLSFIPRNCYCLVIFEDCHLAIRSHPYRNIVKEFLNIEKEYRPRLLGLTMSLINDEVKGDCLQYGIDTMQEVLCSKVVLTMLSPAKKFLKRDTKVVLISYPSTSLFSWDMFDNYILHLLVSPTFILENITLPNDDDMICMQHVIESLNKVKWIHDEMGPWCAWKVCQKQEMQLNRMKRIKPGTMQYLLIEMGQTYLRCLRKVFENHVKNLKNFSSLCAYITGHVYSFIELLSHNKMRSVFADDENFPENFCCIVFFKHRYIAYVYKILLKTLQNLWPDMFGYLKVDFLVGYDSETADASKEALHERQHEVLKKFRTKELNLLLTTRVLAKGIELRGANCVIHYDEPESLRSFIYVKNRANKPNSHYFILLSECTSALSASVINTFVDIDKIIRNYSLLDYEDDNLELPDDLDDTFKPYYPKKSEEVGDDDEEADKLKNNNVCATLANSIRIVNHYCQRLPCDIFSRLVADCHVEKVMSGECAPSPKYRATLKLPINSPGPTVDSVMLARRSVALETVKLLHRRGELYDSLMPIGKEMVASLLIADEDDEEWPVTGKACPGSSKRRQYYNKAVRAGACKSLIECLKRAIPVFDEPGYFHAIIIREVSEICNGEANISQPEVGKRILGIFSSKPIPQIPAFMIYEKTKSFLVEIRPCASTITVNKGQMAVIAEFNMYIFSEILKLEKYSMKYQPEEAENAFFIVPSISTETDYILDWQFIVEVVNYWDRVPRRPDEEQRKNFVFDISNLVKIGFVFFNAFRYKDAVVMPWYRSQEMSHCYFVLNVDEGLTPLTKFPDDEYESFKSYFWQKYGLEIYHDDQPLLNVDYTASRLNRLFPRRQARRESLEADYDTMSVVAQKQKLVPELVDIHPIPASTWRCLQYLPSILYRLSSLLLANELRLQVLLDEYNNDPTPDGYCWAPIDKAWLKETMRMTDHGCPWAKMVNKSSKNSGINCSSNSIDSGKNKNNNNNNNSNNLSNSNIKSEDSARFEQMDFEISVWEPVQEFVFNGFTDDGKHDCLDANSADFGSDQLPMSDGQQSFEDCLMQMGDYDGSMLDSDEEIYFDTEFSRKFMNVMSLHGSYGQHPFGLLFGQPIEPSGWEVDDVPTMPAADEHCDVGLHFVSAGQSLNVSTLLADVRDADEKVRGLQQQQPNAKEEDTVPTSNATAQKVPVDRNGTEAKITSRHKIQAVDTLAEEETVDLNMFKRAVYNRKGNVLMNGNESWKNFTNEQMLTLDSSWYEWPGEFRTAALLPAGETCTFLEIANEETPFGPNPREVLHAITASAVAETFNLEGLEILGDSFLKYVTTVYCYKAYSKMHEGKLSLLRSRMISNYNLYKLGKRKNIPQYMIAIKFDPSDTWLPPCYVPLNCEVQDTAIEEEDKLMEQRLMNDESVVEQQRNVETTGRQATTGSKSTKWVPEDLSQLVPFNLLAQQGISDKGVADCVEALIGAYLLFCGTRRTLDFLHWLGLKVEDEVSLRGFSRCIVSPTPISFANFDLYGVTKSALINNSADSEACLKTLWNRFSLSQFEDIIGYRFKDRSLLVQALTHSTYFYNEVTDCYQRLEFLGDAVLDH LITRHLYEDKRMHSPGMLTDLRSALYFMYLCPGLMMMIEKFVKTLNIIKENANFDREVRVHLLNLYLLEGEEENAEEQVEVPKALGDIFESVAGAIYLDSGCSLHTVWCIYYNMLREEIEKCCLNPPISPIRDLLELEPDRVKFSRVERNAVEGKVKVAVTVEGKGRFVGAGRSYRIAKSTAAKRALRYLKGSPQMSLISKIH

**DRH-1**

***Ancylostoma caninum* DRH-1**

ELVNQLCGNKDVDFRAIVFVRTRKGASVLANLLNSHADLISCGIRVETVAGLNSSGVDTTTKREQLEKLKRFRDGETRVLVATSVADEGLDVAKCNLVIKYNYASNEIAHVQRRGRGRAENSRSILLTQNTKLKEQEEKNIVKERLMRRVLRAIEENRINLVARVRKAAEDLWVEIQREDDIESRRIAEQKSLGIIYXLLCSKCDETLCTSKDIKTRNSQYCVCNPSFWSKVRNEEICGDAREARYGAVAKLFCVRKNCQNLLGRVVCIEGTLMPVLSSSAFVLEFKEGGTGNTTRRTVRKWKEVLKDYFTPENIRNYDLAVMTEASNKPVVNXYRSVFPIRVIVFQPTHH

***Ascaris suum* DRH-1**

EIRKEFLAKPDGRAIVFVRTREFACRLREAINTDDSLSDIGVMSEMVTGINASTEEGGQNVNVQREKLMQFANGDVKVLCATSVAEEGIDIQKCTLVIKYNYATNEIAHVQRRGRGRAEGSRCILLTHDSSLEKRENDNLTRERLMNIALEAIDRKPKDWFRREVYSRV

***Brugia malayi* DRH-1**

MSSRSNELDKTCIVDLRYEHFPVNASDAAKDDIYEGEVMVLRKYQEEIAQPAYXGQNTLICAPTGTGKTVVAASIARNHLVMGRKNNLHTKICFFVTNVVFLEQQTKLLKRFVGHRWKVVFLCGVAANTPVAETIATHDVIVITPQLIVNLMNDSNEKNSVLFSLSSFSLMFFDEAHHADGNHPYNVIMNNYHDMKHTGKILDGKRLPQIVGLTASLGIGSAQNASEAVEHFIKICANLDITVLSYVRENIDELRAFSSIAADVRSASMSXKIHDNSNQDLLHKLLNPPHDKCSKVYETWFSQLLVGFVPLAKLDRVIRFHLMTCLQLIGILFRSLDYYIHFPSCVAKKYFENEFSFIRHTADQELVDIMENNELYNELLRELRDQFARLGDGRAIVFVSTRYFAGKLAEELNKDESLHMLNAKSDFITGINASGEFGGQSANQQRNALVRFTSGRGRAANSRCVLITNDAKLQAREEKNINREQIMRNALKLINQKPPDWFQDEV

***Caenorhabditis brenneri* DRH-1**

MVRKKECAALVRLYEREIIRCLEPIFREPDRGDKFEELLTRGRIEELIGESDNEMDFAKKLFVELTSSPLSLADDERLYKDVMAYLHDNLRNSAVHRLMSCSDKSIIRSEFCKILRNLDNFLRFLNPTALLGFLGSYPQHYEDLVRVFNEWRNTEENEEDNQENMKKAILKMVPLFGEFAVYDIMYSIHEHTTNDNRQEAKSFIEQLLHLKEGEFRRYYGIFHFSDSINADRLQRNGQMYICPIHESATEMLVYIGSPNFNTNRYRMINIRHDNIQPEDSVQRLVIQSVRHRIDQQRQLCLRGYQQELCRVALRGENTIVTAPTGSGKTVIAANIIKNHFETRERQGRRFKALFMTPNSMILKQQCDSISSYLDHVYQVTIKVQKIENDEDFQVQIVQGADNIPVRDAIKSKDLIVATPQMIVNLCNEHKDESRSENDIGIERFFLSTFTIIFFDECHSTLKNSPYANIMREYHTLKNMGNMPENHHLPQIVGLTASLGTGEGKNLLGVKDHIASLCANMDVKELSIVKENLDELRDYSPIVPDSELSLEKLLKTAYTTAFHRSQKSAIVKGIPMVPSRNSRSVLNEHIQVQEGQQIQPRFDDREYAPTNTFITAPEDKEHSGYLNWVCNQMNLVSITNFTDRRTKISINEALEVLKECYLTLNYNVNFNPEVALRYLRDEIENRVRNFTPEMSRIWDQYHNRLVTTGTAQNSMILNLEQFVVDQNEQSPDSRAIVFVRTRYEAKILNEILNNSERLRNIGVKSDWISGLNKSTSGSGEIAASKQKQAEKLRKFANGEVRLLVSTSVAEEGLDVAACSLVIKYNYATNEIAHVQRRGRGRARNSTCVLITNSVALRDQESSNRDKENMMNQTLLTIQSNPIAFKETVAVEIGKIWNRIVREDTERAQHVAEQVNQNILYRIVCKKCEVFLCTNKDVRSRNTQYLVCRPEFWSLILCRGANCGATLGRLLDINSVELPCLGAEGMFYELIRFYHHVEYYSYRHHQRWNRGKK

***Caenorhabditis briggsae* DRH-1**

MVRKKQCAALVKLYDREIIRCLEPIYREPEKGEHFDELLSRGRIEELIGEYEDTTSFSSQLFHELQESPISKADDERLYKSVMSFLQANFTDSHVHKLLKCSDRTMRMCQYGLILNHLDGFINYMDPNEVLTYLDSYPQYQDIVRELRRETERIVPEEQQQNTEFLKKSILRIVPLLGESSAYDVMYAIYDKPSNNLNEEARNFIEKILRLRPGSFDAFYREVSDDRRRFNGNIFICPINEIATEMIARIPEVNRHRYRMINIRYDNIQSEPPVPRLIVESVRNRIHLQRQLCLRRYQLELCQVALRGENTIVTAPTGSGKTVIAANIIKNHFETRDRNGQRFKALFMTPNSMILKQQSDSISSYLDHSYQVQIVQGADNLPVRNAVQTKDLIVATPQMIVNLCNEHRDVLKTENEIGIEQFFLSTFTIIFFDECHNTMKNTPYASECFPTIPVIMINFSDIMREYHTLKNMGNMPDGHHLPQIVGLTASLGTGDGKNVLGVKEHIANLCAMMDVKELSTVRENTEELQNYSPIIPDREILNRNDEFLSFYEENIPIQNVQQNVQNSLFDDRPYRPAENFQSAPNDKEHPGYLNWACNQMNLVCTAKFNRDGTKIIINEALEVLKECYWTLSYNVNFNPEVALRYLKSELESRSANFTHHMSRIWESKFQFLLLLLCIEISGYHNHLLNSGTAENPMIEKVEQFIVDQNEQRGDSRSIIFVRTRYEATILNEILNKRETLERLGIKSEWISGLNKSTSSSADISASKQKQMEKLRKFATGEIRVLVATSVAEEGLDVAECNLVIKYNYATNEIAHVQRRGRGRAMNSTCVLVTNSIPLRDQEGANRDKENMMNQALLKITTNPGAFRDAVMAEIGNIWNRIQREDTERARQIAEQISRNVTYRIVCKKCDVFLCTNSDIRARNTQYLVCRPEFWTLVQKIKLTPGEIATNKFHSTGMIKCLGTNCGAILGRLIDVTNTELPCLSAEAIVLINEHEDKRIQAKKWKKVFSNSETILSDHFTPVEIRQLDIQKMRDANQTRTPLNFELNLNGILQNIIREA

***Caenorhabditis japonica* DRH-1**

MARKAQCNALITLYDTEITRCLEQVFRDPEKAGGLVELLCEGRIEEIRMEFEGDASGFAKKLFAELKMSPLSLADEQRLYMEFMVFLQENMRNSEIHRLLKCSDEAVRRSEFKILLNHLDEFLRFTDPREVLKYLDAYPQYYDVVQVLRIEMQHLQQTLAERQQNTTGNEHIMGKLLLRTVPILGNLAIYEILFVIYFNSSQNLDEEAKSFVNRVLQLKPGQFDAFYIFFFLFLPFFPVLLPFGPIFVRPVRPFVRPFVRPVRPSCPMISLRYDHLSNTDSVPRLIIDSVRNRIHEQQPLCLRDYQEELCRVAISGENTIITAPTGSGKTVIAANILKNHFEERGNMGRRFKVQIIQGADNLPIRSIIESKDLIVATPQMIVNLCSEHEQEDLENPGFVNEQFFLSTFTIIIFDECHNTQKKSPYANIMREYHKLRKTGAMPENHELPQIVGLTASLGTGVGKNTIEVVDHIAELCATMNVRDISVVRENLEELESYSPIVADQVCYCERRSDGAIGEFAKFMKDMMREIEMVYITALKSSGEQVQTSVRLGNEGDREYRADSEFQIAPEDREHSGYLNWVCNQRNTISSTHFQQPKLKVKANEALDVLENCHWTLCYNMNFNPEVALKYLRKNMDERSPHFTQEMARIWERYHNRFTSAGSEQNLMITKLEEYILEQNRRQPDFRAIVFVRTRYEAKILKGILNRNGVLKDSNINSDWISGLNKSTAGSAELAASKREQMEKLTHFSDGLVRVLVATSVAEEGLDVAKCNLVIKYNYATNEIAHVQRRGRGRAIGSTCVLITNSPQLKNQESTNKDKERMMKAALAEIEKSPAPFKTMVTEKVEELWMRYVTEDTENAQRLKKQQKSDATYQILCKKCDTQLCTSKDIRANKTQYVVCVPEFWSRVRKIPLSEEEAIRDAKFHGKGKMICRGTNCGSQLGRVIEIASTEMPCLSAEAIVIVTQSGVRTPIKQWKKILEKFFTPANIRQLDIQKMRDASSRAAVNFEIHQMNGLVDTVIRSN

***Caenorhabditis remanei* DRH-1**

MVRKKQCAALVKLYGREITRCLEPVYQEPEKGEYFEELLSLGRIEELIGEFENAVDFSKKLFQELSESPLTRDDEERLYKNVMTYLQACLPGSNVHKLLKCSDRTMRRSQFSTILNNLDGFLRYSDPETILRYLDCYPHYTDVVIALRREIEQNRNDETEDEDFIKKLILRTVPMLGESSAYDIMFSIHENTSNNLNEEVKTFIENVLQLKRGGFKAFYEFISADRRQCNGRIYICPIHESATEMLVRLPEFNDSRYRMINIRYDNIQAEESVPRLVIESVRHRIHLQRQLCLRGYQEELCRVALRGDNTIVTAPTGSGKTVIAANIIKHHFETRERLGQRYKALFMTPNSMILKQQSDSISSYLEHAYHVQIVQGADNVPVRSAIQSKDLIVATPQMIVNLCNEHRDELMKAEGVEQFFLSTFTIIFFDECHNTLKKSPYANIMREYHTLKNMGNMPEGHHLPQIIGLTASLGTGDGKNELGVKEHIASLCANMDVKELSVVTDNLEELQGYSPIIPDEVTYCERGTDGAIGLFTRWLCDMMREVENLITLALAQELIPVQDGQPGRPIDDRQFGPINEFQSAPNDKEHSGYLNWVCNEMNLVSSKKFNESRTKIVINEALGILKECYWTLSYNVNFNPEVALRYLKSEINLRSSNFTPEMTRIWDRYQNHLVTTGTADNPMITEVEKKIVDQNSDQNDSRSIIFVRTRYEATILNEILNKNERLRNLGINSEWISGLNKSTAGSADISASKQKQMEKLRKFASGEIRVLVATSVAEEGLDIAKCNLVIKYNYATNEIAHVQRRGRGRAINSKCILITNSIPLRDQEGANRDKENMMNKALLKIQSNPFAFREAVTAEASNIWNRILREDAERAQRIADQISQNVTYHILCKKCEVFLCTNWDIRARNTQYLVCRPEFWSLVRKVELSPADADRCHSTGKVKCLGRNCGAIIGRLIDMNSSELPCLAAEAIVLVDQRNTSKMITIKKWKQILSKYFTPVDLRQLDIQRMRDAIHARASLNFEFHRNGIIENINREI

***Haemonchus contortus* DRH-1 (Likely exon-intron boundary issue with predicted protein)**

DHHDGFLSFTDCTLVLISTVRKNEKELMKFSPLVFDEAVKQRQNVSKWRNCVGKYLLKRCY*PNFDFSKCDAFLCTSRDIKTYKNSQYCVCDPSFWEKTRNEVIPVGTSKINRKRGHRSCREAVCNVELQGKFTVLMRFYHRLSFSQRIPSKADLPQVVKEQLFIPDEIRNYDLAAMNEAAVKPSIPNFTSKGVAVRLLHCVSPNCQNVLGRIIHLEQTMMPVLSAHGSFLVVFATPGEKCFVVEKAKVEEGIKEILQEMQREDANVSQRLAQQKISGKVYRLLCR*GFS*ACCSRGVTSQTSELLGTNEFDEILVDSSDDADFTHKLWEALMNSPLSVAFYDTILHYLSTIIGLTASLGVGGKSTEKEAIAHVVKLCAMLNCKGRGRAENSRSILITQNLKMKEQEERNDSRAIVFIRTRKGASVLAEMLNRHPGMIEEGIRVECIAGRSVSLFMTPNTVILDQQADSLRKYLGHRYQVSLIKRLVLRTIPLLGDNAIYDLMRSIYYNSEQSTEFVNKLHPNFLRFYNLLMENTVSDGIENAIQPPFSVTVFTLMVFDECHNAVKNSPYSSYCGMCY*VLAVRGSDNIPLREIINAKDVIVATPQLVVKCDAFLCTSRDIKTYKNSQYCVCDPSFWEKTRRFRSETPILLREYQKELSEKALNGINTIIAAPTGSGKTIVAVNIIKNHLDKNIRNGRRAKFRDGETRVLVATSVADEGLDVAKCNLVIKYNCATNEIAHVQRRGELSSFQKMFFSL*CLIAGRGRAENSR

***Meloidogyne hapla* DRH-1**

MEMLEKSKLKEKPQIVGLTASMGVGDTSLDIKACCEHMLNLCSNLHSETISTVRHQLDNLKSHVMPPVDYVKRVRRPAEDPFLDYIERAMYKIENEMKPRLPKLAELCKLKKEEIEFPQHSNSSRYQTIVGTLKKCAQRVQESEMRFLLVRSIDHLSHYFHSILINDLLPSSYAFQYLQEKMSDYKQNTGGSSPIDLINQRLLSYYQDLHPKLFDCVKNEKLQNKEILKELHSILRRQFKSDPNSRCLIFVATRNSASKLADHLKRVPELPIFYKKENVGYMVSSNQSLSAGGQSTQEQQIMIRDFDSGKVKVLVVTSVAEEGVNIAACNLIIKYNNVGSERSMIQRRGRARQKNSLSILLALDTGVEQAEYLNMQKEAMMMRCLLDLQEKSETNLKNQINAKREERRKIEERKLKVLEAKRSRLNNRRYKLSCRSCNNLICKSTHVRSIAYSTFVVCDPTVWKRSKIDIREKPTKDHLFTKCAKWLCGQCGNQEWGVIVKYSNCYLPQLSATLFSLEREDQHDEFDEMRAGDNRGRTWNNIQDDYFNITPINMRNIIDMFSALTNSFSNLTKQMDQQECIANIKFIEKMKEKKTDRKNKIQIFLEE

***Meloidogyne incognita* DRH-1**

MFCVEQLLIFYNAIIWLKLFGVKVSFNYIQKNEVDKLICKFAIESKINVELNNWINSLQEIVNKKGMDKQNSALLDKLILILNEQFQTDSNSLLIFCQQRDHCEKLSTLLNEQTTYRTDFFTGQASREEGGQNARMQKAKLDDFSKGEIKILCATTVAEEGIDISACNLIVQYNYVTNEIARVQRRGRCRAKGARALLLTCEINIKEKEEQNALRERLMHSALEELSRWSPTTFKLRVSIFYWVEDLVQELNKKRKESEALEMEKRIERRKQDNLFKIVCSSCSKFLGLSTKIVLVGSMYVIVDKEFWRRTKGCASELPPEKAQGRECKGSMPHIGEHRC SCNQKLGRIIQYRGGIILPNLNVDRIVFIRCTSDGNEIIKDERVKERKWGKVSQNLFLIDKATTLQLVEMKDAPDKPESLLRTDLLE

***Oesophagostomum dentatum* DRH-1**

TTTKREQIEKLKRFKSGETRVLVATSVADEGLDVAECNLVIKYNYATNEIAHVQRRGRGRAENSRSILLTQNEKLKEQEEKNVVREQLMRQVLHLIQENRINLRERVKKAVEDLWVEIQKEDVLTEQRVAAQQSSGIVYKLLCSKCDTFLCTSKYIKTRNSQYCVCDPTFWSKTRNEEIDDDGAEAKYGAVAKLYCTGKNCQNSLGRVVSLDGGALMPVLGASAFILEYTDGSAVVRKSFRKWKEVVRDYFLPEKIRNYDLVAMNEASNKPKLAS

***Trichinella spiralis* DRH-1**

MGELLISLITSFAYETFSNSSHKVGVDIEIESLNEVATHKKTLSLYDFQEDLVAPALLGKNTLICSPTGTGKTYMLLKVILNHIKHQKLTHKKYKVSTENMNRIVELCAFHWHAFPDMFDRTDCGVGRTTLESVRRFDVHIRPGQRTVDLHCADFAERAQLQYRRKFQLEYQLVHVVAVRRMPPRCRLPSVQRVDGFLLQGSLPGAADCRHHGFGMLRQRGNSTSDYFQISLILPDPIPNATFLKDLVHILRTLEVTLIQRTDQTAINISTLTRLQDKMQTNYRSELGQLKQAVVTIKDPVVRRNALSYLNAMLHTHESLDIAVHTSFILGFNHFVNSYICHFENFSQADSMFYQIQQRKIQALEGELVRAYKSPLYESLKKIVLNQFNLDSDSRVLIFVRTKKLAAQLCQCIKDDSDVDIYGVNFITSCSSGATDPGSKVIVVNRLQQFKEGEFKILIATSLLEEGIDVAACNLVIRYNYVTSVISKIQQRGRTRKGNGAVVLIAYEGFYSKKEKTLEMQESLLLEAVKKIKLNGNVWFKNELSKLKAKRNLITFPPKTSHSFHSLMMDSKIFCRMCDYYFAPASSLRHVNDTLVVCVDRDAFRQLWPSEAFRLEPDIVLHCNHCRSKVGQMLRQMNANVVFFSVKSVLFSFGSANERKAFTKWSKARLSLPPLSELLVKLQWILMGKEEEEEEQQQQP

**PASH-1**

***Brugia malayi* PASH-1**

MTGIMRTNPSNCSPFIIHSTEMCNASLLMADIPEDVKLEQLLELREQLREQIDQLNANNQLESSSSMRKISKYHHANKYQTGAMKKTEKRPNSWIVDGYANNKFAKVSLPVCMEDGRNDEPSCSDMKKFNEKDRGASLLDIPTTHGQNLHQCPFRHKSVLSSDITGTENGSKKLSLHSEIAELAQNDVQNAGIVGAAKIDNIPTEEKMEEISLSSLIGSMENETAKKESSSVFNAHTGSAVLCKSTENNFSIFPEKLIPLPPPPPPPPXTPPLAPPPPQLQFLMSNEMNSTNSESALLDEQQSEEPEERSRSLSRPAEDSGSELNGIDGRNNKDEKDTFLSDEDDGDDIDLLLEKPIEDAQTGKVIMPEDGEVREKIVLAYRGTDYFDVLPEGWVEVTHSSGLPIYLHKSTRVCTFSRPYFIGPGSVRHHNVPISAIPCLHQRRVLKEIEDGAAASAAALQNALSESVGVQSSDSALEDSALVMARLQAPGTKVQTAEDFKERQLDAEALHDYAKAVFKFRKIQTYRFNKWAATRNFHRQRKLAEAGRLGKNASSIELSVGRPALPSNVKLITVPALEANLKPQHRGFFLNPQGKTSVSVLHEYVQKVLKSTINYHFSETRNSSMPXGCTARLKMNLNNRVLCATSVKEKLMLLQEKQRREQRLQEHGEQPDADFVVLGTGCGNSKKTAKLDAARNALKVLIPDVEFDPEGIARSDKKEKDDAESDKEDAVALFDMLPIEDSRIFDLSARAGQPSPYLLLQECLKRNAAYGDTEIKVHSVRVKHQRHQFDMEVGKHRVSVICANKREGKQKASQAMLKKLHPNLDTWGSLIRLYGHGTQQKQQEARKSRQSIIKLQGNRTKESSVLEPNNAILEKLRTEMLKLSKSKQSIENRNMVPKRPKLSATNEPVDEIQSAEASASYAATHPDALSALRIDL

***Caenorhabditis brenneri* PASH-1**

MDEGIGNSQSSPEQVDDEHQPALLDDEENMECDDQDLPRAEKNDDQTEECVVDRADVDGPLPEGWKLVYHATGLPMYYHEETGVVTHSKPFQLEGTVPDHEVPIAAIPCLFQKVMSDEMKQNNDVTLQDNECPISAERKQELLECSYGSRDTKCILLQFTACSENVMPTDESVASPRRERRSHRKSIESIVPRSSSKCRKRLVFVNSLMFYMFFGFFSQISERVNRYTRKERNVEKIEKYKNDRFLCESGYNITYDELRKDYDPQEIILEIGDGKVLIDFTPVLLSANGKKGNSKKPLLFDPMGKVALQLLNEFLQRLGGGQITYNHVDNKSSSYPYLVTASLTMKKSELDEMVGECRERLVVLCEIAEATGDSALISANVSSENKSFPIGSGVGKSIKEARQASAKDALSKLLPKLRINENWVCDGEIGDEEQRGFDEESKEIFKKVKIEQPNLLNICKAHGVPTPLSLLETAAKCSKRWAGRKIDFVKERVGDQISKVTLTFGDMQCQAKAIGTSEATLACQLMLKQMHSGLVSYGALLDVYANVQKKSEMNRAKQQHDELVRVQEVENRTEPLLCIFEKLQEEMKKVNLKYPKQLLNQTPLPKQTSNHFSQTDVEPLLPSPMMAMHPVPLPPSMTHWLSNDVPRAQSSSRSIPPPSSSMDRWSPNHHPRAESSRRTLLPPPSNGHHSQHHSLPRPPPPPPPPSNNMPRHAQSSSRHQHGSYSSQSRNQDFPRKRSRWDNPPSSSSSYYSDRRPPL

***Caenorhabditis briggsae* PASH-1**

MLRVYANRAVALVSVHPLKPVIMESESVGKYEEALIAEREAILRQLALLGSDSEDEEKDENEEQSEEPQNVKTEPCEIEPTTCSESVEDLNAVGPPDEKKVLDSCPQETDSFGLDEDNEVDVDEEEKPCSAEPPSTGTSSENSRSPPVPVEKVVLDRGPADRIDLDKNHPLPDGWTVITHFSGMPVYYHRFTRVVTHSRPYQVEGIVRDHDIPLPSIPCLYRQIMDEKQKNLENQSEMTEEEIQENLQLHESEKTMSPERYRLYCEGRFKFKKITSASFQVHRYTNPEEKLGAVQKKRLNSMLKKKGFELDYDQLKKNNTPGEVLLSSQSGACLIDLTPAQAKLGTKKGGPRKPYLLNPMGKTTVAVLNEFVQRLAKGTLVYEVEDTRNVSNPYQATAMLTMRISTLRELVGQCKESLLVLSEKADLTGNSQAALDPEFKRFSIGCGVGANKKTARLVAAKDALTKIIPKLRISKEHVCDGVLEERLQKGFEEEAAELFKQVKIDSSSVVSMCTRFAIPKPFNLLRDAVSRAVRWNGMELKTKKEMVGNGSQLSKVVLELGDMEAGAEAIGVKQATQLAAQLLLQKMHPELPTYGSFLTLYGRFEDKKQLDNARRQHEEVRYDVVRLQDTGNLLQPNTNVLDKLANEMRNISLIYPPRRYLYGLEPQSSGIKTKTETATTQTDIIDYQFMIQPHMVPVQPPYGLPIPPLLQPSMSSPAFVGAPPQDYYYYQQPFQNQRNPRKRGHPTTPPYPHPGPPPPPNV

***Caenorhabditis japonica* PASH-1**

MQDGSKSQEQLLMEREEILRQLAEMGDDGEDISDSDEEEGEEECINMSDGEVQVKKEEHDSIETSPVENTLIPPESPPNSVKSELKVENENENENENEEEEKSTAKGDSEPPVPVKKIVLERIDHDKTIPLPDGWSFITHESGMIVYFHKQTRVVTHSRPYQVECPVRDHKVPVFAIPCLYRSIIDEEKNNADSQNAVDSLESVEQADTPCEKIPPANKEIPYCWNQNENLSPMDFRKFCEKRFRFKEMTIMRYTNPREREKAQQERKANAILKRSGFGVDYKNILEKNEPGNFVLSSPSGAILIDLTPLEKGKRSGPRKPFLLNPMGKTTVSVLNEFVQRLVKGALVYEAEDTRNVSCPYKATAVLSMKRSTLQEMSQECRESLIVLCEIAAMNDDPNSGTEDIAKFPIGKGVGANKKTSRLSAAKDALSKLVPKLRISDDYICEGVLDEATKNDFDRESRELFKKVLIDSQNLAQLCTQFMIPKPYTLLTNAVSRYIRWNGMELETIWKGRRLRWA

***Caenorhabditis remanei* PASH-1**

MEPESGGVNQQLLAEREAILKQLALLGSGSEDEGDGDEEEEAKKVKFEVKEVKVEVKEEDNIKVEIVETEVGYSEPVLFNSSPDSIQSSTALEEEEEIISDRKTPIDCDEREKREADSSNTSSENSRTPPVAVEKTVLDRVDFDKSYPLPDGWTRIAHYSGMPVYYHKFTQVVTHSRPYQVDGMVRDHEIPVSAIPCLYREVMDKKYEDLKNHRTENSENCDEKEKRCPIEIPPNESKMNPEQYRDYCARRFKFKKVTVHRYIDKEGKQSAAQKRRVNSMLKNKGFELDYDQLKQKNQPGEVLLTSASGACLIDLTPIQPNLSLKKGSGPKKPYLLNPMGKTSVAVLNEFVQRLAKGTLVYEVENTRNVSAPYKATALLTMKMSTIRDLAGQCKESLIVLSEIATASENVPAPGIADDFKRFEIGSGNGSNKKTARLVAAKDALSKLIPKLRMSEEYVCDGVLEQELQKDFETSSKVLLKQVKIDSSGLVEMCNRFGIPKPFALLKEVVARSMRWSGVEFKKEREMVGSGSQLSRVILSLGEMKSSAEAIGMKQANEIAAQRLFKQMHPDMESYGNLLKVYGHLVDKTPLENSKRQHDEVVRLQDTGNLLQPNLTVIAKLTEEMEKVTLIHPPRNFLYGLSPNATGVRTNKASMDTQTEILNYNFIPHMNPNPIMIPPPYFHVPPQMQPPMQPLLHMPMVHSPQFSAAPPPVQDYGIYQSPQYQSPQYQQRNARKRSRPDETLSPCSYSMHQKPPYN

***Haemonchus contortus* PASH-1 (Likely exon-intron boundary issue with predicted protein)**

HRKTRVVSLSKPYFIGTQGVRDHRIPVTGIPCLHQRKYVPERLEHDHFMNLPEGWARITHNSGMPVYNLHFWVRATALTVMRMKVISFRCS*CTLGVLSTLLEAIYCSNKFRNLLDSIIVGRGEGPSKRVAKMVAAKQAL

***Meloidogyne hapla* PASH-1**

MDSDNSDNADEFTVDELEQLEQMRNALLAELHGDEDQEEDQICDEGNAEEEMNGFPNNNEENNFNSDNANISEDIHQNEHSFAGVIQDEDDEDSNSEEEFDQEEISKHIDNLLEEPIKDSAKTTPKHLSKRFKRVLEYRANDHFNVLPDGWVEITHASGLPVYLHKPTRVCTFSRPYFIGRSSIRKHKVPESAITCLYQKKYQDEVEASLKAQEPSVVNEDEVDKDLREDSSNILSAKLITPDVRVKTELDQRKMQLTSDQLYNYAKNRFKFKEICIYRFGKWTEARNFYKKRKMRQLLVGGEARDCGQIKTGFKTREERPGLPSDVKLITVPSLEIDSKPNKRLFYLNPQGKTSVSILHEFVQKALKCTVRYFFSETRSSATPYHCAVKLVLNNTQTTPQSTGRKRFHNRQQNQQPTAEIVKKKLALMHEDFNKINSEQQLTSNTDAENKSDDKAVDDLSPDSEFVVLGEGFGPGKKQAKMIAAKAAVEKLVPGVEFDADGIACNSNSKNELAENTPTSSNNPNLITSCPLMASGTSVGSSITGGDRNDLHIFDMIGVTDTRIPELCARAGQPSPYLVLQEYLKRHSAFGDTAINLTSRLLRHQRHEFKLSIGEELSVKVISGNKREGKQIAAQAMLKKLHPEVETWGSILKLYGYEAQQKFRDARKNKDSVVKLQGIQDEANVRQFQPNSLILEKLRNEMLKLSGDLIKNCSRQQNDGEEPLAKFRRSDFCSELPVLSEEKLSELSAKFMSERPELQKCLPSVKF

***Meloidogyne incognita* PASH-1**

MDTDNSDNDDVFTVEELEQLEQMRNDLLAELKGSEDQQEDSIDYEGIENEKMNEFSNNNVVKDLKDTSGSANPNEAIQQNESFEEEVIQEEDDDSDSEEEFNQEEISEHIDNLLEEPIKEGVKTTPKHLSKRFKRVLEYRANDHFNVLPDGWVEITHASGLPVYLHKPTRVCTFSRPYFIGRSSIRKHKVPESAITCLYQKKYQDEVEASLKAAQDPSVVNEDGTNKDGGDDSSNNFLPGKLITPNVRVKTELDQRKLQLTADQLYTYAKDRFKFKEICIYRFGKWTEARNFYKKRKMRQLLVGGEGREGGHLKTGFKTREERPGLPSDVKLITVPSLEI DSKPNKRLFYLNPQGKTSVSILHEFVQKALKCTVRYFFSETRSSATPYHCAVKLVLNNSQTTTQNTGRKRFFNSQQNQQPSAEIVKQKLALMHEDFNKINSGQKLSNSTDAENKAADDPSDSGFSFNSNIDN

***Pristionchus pacificus* PASH-1**

MDNYDDVDDAHSIEELEQRRLALEKALMSGGDDSSDESDDEGGENMSKSEEESASEGDNSQEDEDNWEVVPAETDEGPETSSSPKRPKMECPMKSAERQNYPDEGSSSTPTINVDQINEAAGGNMLKRMKRIPDYGSSDTDLPLPDKWIKTLHDSGVPIYMHLTTKVVTLSRPYELKTDSLRRHNIPLCAIPCEQQRRMTEHRENNRLRISGTNEITLPQVQLKTMDQIQVERLSPDHLYDRAKSTFHIKEIMVYKKNDWKTIRNDRKKEAIETALKDETTIDMDVQNPMITVAAGCAAQGKKIKKRGYILHVVGKTSVTLLNEYVQKILKGSAVYKSDE VKSSINPYSTMCYLKTKNDPNAVGDEASNLFLIGRGSGDSMKKSKRQAATDSLRNLIPGINIDANHIVISNVSDKDTMEGFDMLAVEDPKVIEYSKRTGRQLPHTILLECLRMNASWGTRAPEESNTRLGHQQYELVLKVGEHEAKVVCKSLKEGKQRAAQSLLKKLYPVMETWGSIFRLFSASAEDKETRRVREEIVRLAPEIGGKEYARNEAVLDALRNEMKRASARVVARLERKRGLQESERRYEEAPPTTADAVKMEEE

**RDE-4**

***Ancylostoma caninum* RDE-4**

MKAXRRVYGVAPEYQLICEVPNFGYSCQLAGKSVTSTAKTKKLAKHLAARAMLHEMIEKDRYSEFGIPGSTKEEAHQAVNALLCGDSREPSEEDPSSPGSQNENYSGKLLQLCQRKSFELPQYSVVEEGPPNDRIYTTTCKVVGQEVKGVAKQKKASKNIASQKMLEILMKGVADMNEEPSLDKLLDDEVIAPAGSSPPPSSPPVSDSIKAIEEILSDTKRFDTPSQEYRILENKSAFGLTQCLLKIIFAKPGEQQRTPYVFPGEHSTEEGAKQQAARRAVLFLTS

***Brugia malayi* RDE-4**

MEKLNVMDTDNERIVEMQQNEELLTSSTGISVTPTKTFVSILEEGCKKYYDGSPPVFTSNFVEMRNLFATRCELYGIYTMGYGRTKKIAKQMAAKDMLKKMIEKESFSDFGLGKTKEEALANLCQKLKLLNATYEIDEEGPTNQRIFIATCTVGKIHVVAHGKTKKIAKTLAAQKMCTQLENWKDLAYDVENAMFPLAAAAAASQETTSSNESAANNLSPSASRDAVMPLQSLQKVRAAFQNGNGTQDFTKKLTELINSDDENINRIFLNQKLKFIFLEPDLDGNLQCMLSINGPNDMKNAFYGFGATEEDAKDYAARIALIHLELFMRTPEPTRNSTNSSETQVAVNSLTQAKNPPENGSQNSSPEKQ

***Caenorhabditis brenneri* RDE-4**

MSEFGKLTFDSVFNGSDAPLRPVLITPESPNSKIQKNKADLENFLKKTPLMVLEEVAKGTFQQTPTWKTDEYPSTVGPEFEMTLVFRGKTVKGRAGSKKAAKQKAAFEYLRQIINEGKRLEYSIPGETDEEALANINQISERPEDPKRQLPFSAKDVDKSIPTIKEFPPGVDSGNWVGALQERCQKYKLEAPSYEDSKIESTSEFMVTCTMRSQKTRGIKPKIKLAKNLAAWLMLKSLEEGIESVQNFDMTEQFEELETEENLAAVRQDVLNTKDNKAALTDLLSDKARFAEFTMDFKYPSMSTLGVHQVLLEIQISRPVFSDLDDLQMGAEHTQTEEIIKATAEKEREQKRNMPESGLKVFSGHGSSEEAAMQSACTSALLHFHTFSFTD

***Caenorhabditis briggsae* RDE-4**

MSGFSFESVFCGSDAPFRPSMTERPNSPVSKLAKSKAELEMFMRKTPLMVLEEGAKGSFQQTPSWACSEIPNTVGLEFEMTLNLRGKTVKARGNSKKIAKQKAALDYLHHMVNDGKSAEFSIPGETAEEAHANLNTIAERPVEEPKRAAAAVSVKDVDKSIPTAKSFPENVPEKNYVGALQEKCQKHKLEAPAYEDSKIEATSEFMVTCTMGNQQTRGIKPKIKLAKNLAAWLMLKTLEEGVEAVQAFDLTEQFDELEEEEDRATAQKAAFETKDKKSTLIDLLSDKARFADYRMEFVYPSVSTHGVHQVLLNVFISRPVSPDDDLQIGAEHTQTEEIMKATAEKEREQKKNMPDVTQKTFSGHGPSEEAAQQCACKSALIHYYTFDFTN

***Caenorhabditis japonica* RDE-4**

MSDLSGLTIDSVFTASTNYVVPSSPKEKPIKSRTDVTAFLRKTPLMILEEGSKGTYQKNVTWHNQELTTTSTTEFEMTASLRGITVTGRGNSKKVAKQKAAVLYLHEAIKKGKRDDFFLPGATDEEAHGFIDQIAEKCEESKRSPIKLESQEAGGLEKDIPTISRIPDGAADQNFIGKLQERAQKMKLDAPLYEERQLETSRFAFYCTMCKQKTCGVNSTKKAAKNAAAYLMLELLEKGVDAIQAENFGDDFEELEAEEKGAAVRAQAFADQDKKTSLLNILSDKRRFSEYWMEFYYESLFRKIPESTLTLLVKKVEENPCLWEIEHTDYKDTVKKDRIWWNIEKEMKILKVVKGATVKKVWIQLVTEFKKRTHQCLLQVFTTRPVSPDPDDLALGAEHTQTDELKKANAEKEEQRKNLPESGKKVFSGHGRTPEEAHDSAYTAAIIHFNTYPTKIREF

***Caenorhabditis remanei* RDE-4**

MSNISGLTFESVFCGSDTPLRPSHCDNQITQISKAAKSKDDLELFMRKTPLMILEEGAKGAYQQTPSWSCAEIPSTSGPEFEMTLTLRGKTVKARASSKKAGKQKAALEYLHQLINDGKRVEFFIPGDTDEEARANVNAIVERPQEDTKRGVSITAKEVDKSIPTVKEFPPNVDGGKNWVGALQEKCQKLKLEGPGYDDCKIESNSQFMVTCSMKNQKTRGIKPKIKHAKNLAAWLMLKSLEEGIEAVQNFDLSTEFEELEVDEHLASIRNEVFNTKDKKSALIDLLSDKARFSEYTLDFKLPTVNNFGIHQILLQIEICRPDTPDSDDLQMGAEHTQTPELMKAMAEKEQQRKKLPDPGTRVFSGHGTSEDEAVQSACKSALIHFHTYDFTD

**XPO-3**

***Ascaris suum* XPO-3**

MEKTRLRGPQRLLCLHKDSGNDKARELCLDVIACYVDWIDIELVVNDTMVPLIIGCLNDENASESAVRAICAIIEKGMDAQKKFALVSALSMLLQQNGSLSITPFYAYIYVLGSKGY

***Brugia malayi* XPO-3**

MISIASLANTAQHHLIFEYIEKLKNERDGWKNCIEKIISGCDPEEHFMLLQVIETYLTVRYADNDQDQDIIRRWMYGWLRRLSSPENLPSYLVNKMAQLFALVFATDFPKRWPNFMEEASFLYICAVFFQQMVSSPEITVFFLRTLIAIDCEVVDREIRRTKQETERNMRIKDAMRDNCNYSLALIWTQILVSLTSFVELLQFEAENNDLKTRELCLDVIACYIDWIDLELVVNNTTIPFIISSYCLKNEHTCESAISAINAMVTKGMDAEKKLVLTASLCTILRENGSFDLRENDADDVLRTGALISSLGCALIDCHTCFSKTGDVEKSMQCEVLLQEQADIALLCFSNEDIDASETVVEFLRRYVCILKTQEFEKRHDFTSRMICIAIDRYKAASDVDLSNTDGEVVAEFMAYRRQLRNMLSAVGNFEPEPILMKLEPSVQNVCENWKQCQMNVVEATLALVYDLADFIHTNFGSNSGLISERAKILAIQILQSTINHCGLPCINTLFFEIACRYERILQNSTGLLPLILEAFLDTRGIRQASLRSRSRIIYLFCRFVKAHKLFVGSQAENILAQLESFFTVTSEEDYQLSKEDQMYLYESTSVLIIHSNLPVERKQECMKILGLSLLQKFSRITERLSRTKNVDEVQQLHQRFADVIGYSARVTKAFSNNYTMQYCQCTIIFIELMEVFLDKVTPNNVEGLDALRQYLHRMITCLDGEILAMLPAICNKYLDVDLDLKMMHDFLILLQQIFSKFKKRLLEIGLNIPALFDILWAAQRTSVDTCNESHVKDMVYYNRAFLQTILSILTHDLTVMLTDCGVEFLNKIADSLISFMSLNDVPTQRMTFQIISKLFLKLSNDGSVACIEFLWEKAVCGALQTPLNGNHDLTDAQCILLQHEIYVCLQTLRSCSLERFDQCLQLSLPAHFARNFCQCMSSYKGKQLEKALDTQYAQLRRMKADTIAQ

***Caenorhabditis brenneri* XPO-3**

MFGINGNAGIAVTDPTKQQEIYQALEALKKDELGWKKSVESFMGPLKPPPEEQFLLLQVIEDYLNKRYHLSNDVDVMVIRNFLLHYTKVSRSSPVDQPAFLTNKMAHIFSLVFAADFPERWSSFFNDLFFADNINERKVAFFYLKVLLAIDTEVVNRDIQRSKNESDRNIKIKDAMREICINEIAKSWLSIANAHQDDNVIQCLVLDNIASYVDWIELDLVANDYVMPLIISKFQNSATSESATAAVCGLLEKGMPAEKKVGLALTVMRVLRDNGLLTVNDNNDEDEVTRVGSLVNTLGLVLLDVQNKLCASAILEKEQACCVQEMAGLADSALIVLNNDDPALSDMCIDYIRAYSSFLIKFHPTETNFIEKVIRTGFLRYVMGDDLTVGGDGEDEVEFQEYRRELRSMLNVIGLKRPEAIINAIEPWTSEVTAGGSSIPINRVEALLNVIYHLHEIIPSNMLQTPREGISQRAARLPIAILEGLVLDGRSAAVHVLYFELACRYERLLVLQPQPVVIPHIAGAFLDQRGISIPSASVRTRIVYLFCRFVKSHKTVLGPLVSEVITRLAPLLAVSPQSDVNQFLSPDDQGYIFEATATLIVFGDLTSEMKSQYVGELVSTLAMKFENGITELNAARARKADDETIQTILQFMSNIIGYCSRMSKAFNNAQSMKACNCIEIYLKLIKLFLETLTPENTFLLETTRQFAHRLVVSMEEELMPYMSGIFEKLALVSTDLDSMHHLLIFCHQTVAKYKKAMLTSGVDLGNVLVIAARASIQEQENNLPAKDDSQRALLYVQRAFVQLLLTVISSDCTPALNTTPGLMDHVLEAAARLALSSDQTAQKVALSCLSKISLIVPEWSARTLRVALEIPSLPHITPSDAGSTLVVHEVCATLSSLHQS DPDGFSRALHGLVPSAFSDQLLSALTNLKGKNLDKQVMNLYASLRNQSS

***Caenorhabditis briggsae* XPO-3**

MFGTNGSAGIAVTDPTKQEEIYRALETLKKDELGWKKSVESFIGPHKPPPEEQFLLLQVIEDFLNKRYHSSNSQDVAIIRNFLLHYTKTSRSSPEDQPVFLTNKMAHIFSLVFAADFPERWSTFFNDLFFNDNITDRKVAFFYLKVLLAVDVEVVNRDIQRTKLESDRNIKIKDAMREICINEIAKSWLSIANSLSGDDVIQCLILENIASYVDWIELDLVANDYVMTYIISKFQNSATSEAATSAVCGLLEKGMPAEKKVGLALTIMAVLRNSGLLTVNDNNDEDEVTRVGSLVNTLGLVLLDVQNKLCASSILEKEQSCCVQEMAGLAESAIVVLNNEDPDLSCLCVDYIRAYASFLVKFHPNVREDRQQDFIEKITFQDTNFIEKVIRTGLLRYVMGDDLTVGGDGEDEVEFQEYRKELRSMLNVIGLKRPEAIVNAIEPWTAEVTAGGSSIPVNRIEALLNIIFHLHEIIPSNMLQTPREGISQRAARLPIVILEGLVLDGRCPAVHVLYFELACRYERLLVLQNQPVTITHIAGAFLDQRGISIPSANVRTRIVYLFCRFVKSHKTVLGPLVSEVITRLAPLLAVSPQSEANQLLSPDDQGYIFESTATLIVFGDLSSEMKSQYVGELATTLAMKFENGLVELNAARARKADEETIQSILQFMANIIGYSSRMSKAFNNAQSMKACNCIEIYLKLVKLFVETLSPENAFLLESTRQFAHRLVVSMEEELMPYMSGIFDKLALVSTDLDSMHHLLIFCHQTVAKYKKAMLTSGVDLGNVLAIAARASLQEQENNVPAKDDSQRALLYVQRAFVQLLFTVIVSDCTPALNNTPGLIDHVLEAAARLALSSDQTAQKVALASLTKISPIIPSWSARTLRVALEIPSLPHITPSDAGSTLVVHEVCATLTSLHQSDPDGFSRALRELVPNGFSDELLSALNTLKGKNLDKQVMNLYASLKNQTSQRQHRLHRIFFS

***Caenorhabditis japonica* XPO-3**

MEKEQQCCVQEMAGLAEPALLVLSNKDPDLSSITIDYIRAYSAFLIKFHPNDTAFIEKVIRSGGDGEEEVEYQEFRKEIRSMLNVIGLKRPEAIINAIEPWTAEVTVGRSAIPVNRVEGLLHIVFHLHEIIPVLSISKRIIFGKKNAGRDFVYSPLSSIFFVSNMLQTPREGISQRAARLPIIILDGLALDGRCPAINVLYFELACRYERLLSLQPQSMVITQIAAAFLDQRGISIPAANVRTRIVYLFCRFVKSHKTVLGPLVSEVITRLAPLLAVNPQSDANDLLSAEDQGYIFEATATLIVFGDLTTDMKAQYVGELASTLAMKFENGLNELNAARSKQADEETIQAILQFMANIIGYCSRMSKAFNNAQSMKNCNCVEIYLKLIKLFVGTLSPENSFLLEMTRQFAHRLVVSMEEELMPYMSEIFDKLALVSTDLDSMHHLLIFCHQTVAKYKRAMLTSGVDLGNVLVIAARTSIQEQENIVPAKDDSQRALLYVQRAFVQLLFTVIASECTPALTATPGLVDHVLEAAARLALSSDQTAQKVALSCLARISLIIPEWSARTLRVALEIPSLSHITPSDAGSTLVVHEVCSTLSSLHQSDPDGFSRALRELVPSAFSDQLLTALNTLKGKNLDKEVSFFIFTFMTCLYSGDESVFFASQLKF

***Caenorhabditis remanei* XPO-3**

MARLAEPAVVVLNNEDPDLSYLCTDYIRGYASFLIKFYPNDTNFIEKVIRTGLQRYVMGDDMTVGGDGEEEVEFQDYRRELRSMLNVIGLRRPEAIVNAIEPWTAEVTAGGSTIPVNKIEALLNVLFHLHEIIPVIFHRNQIYFQSNMLQNPREGVSQRAARLPIVILEGLVLDGRCPAVHVLYFELACRYERLLVLQAQPVVITHIAAAFLDQRGISIPTGNVRTRIVYLFCRFVKSHKAVLGPLVSEVITRLAPLLAVSPKSEDNQLLSPDDQGYIFEATATLIVFGDLTSEMKSQYVGELATTLAMKFENGLVELNAARARKADEETIQIILQFMANIIGYSSRMSKAFNNAQSMKACNCIEIYLKLVKLFVETLSPENSFLLESTRQFAHRLVVSMEDELMPYMSGIFEKLALVSTDLDSMHHLLIFCHQTVAKYKKAMLTSGVDLGNVLAIAARTSLQEQENNLPAKDDSQRALLYVQRAFVQLLFTVIVSDCTPALNTSPGLLDHVLEAAARLALSSDQTAQKVALSSLAKISLIIPAWSARTLRVALEIPSLSHITPSDAGSTLVVHEVCATLTSLHQSDPDGFARALRELVPNGFSDQLLSALTNLKGKNLDKQVMTIYSSLRNQNAQ
