# Supplementary material for: RNAi Effector Diversity in Nematodes
Source: PLoS Negl Trop Dis. 2011 Jun 7;5(6):e1176. doi: 10.1371/journal.pntd.0001176 (PMC3110158; doi:10.1371/journal.pntd.0001176)
Supplement: Dataset S2 — Nematode proteins putatively responsible for secondary amplification, uptake and intercellular spread of siRNA; domains and sequence data. (*, putative stop codon) (DOC) [file pntd.0001176.s002.doc]

**Dataset S2.**

**Domain analysis**

SID-1 (System Interference Deficient 1) is characterised by seven carboxy-terminal transmembrane domains and an amino-terminal signal peptide. SID-2 again encodes an amino-terminal signal peptide and a carboxy-terminal transmembrane region. RSD-2 (RNAi Spreading Defective 2) contains three unique domains towards the amino-terminus. RSD-3 encodes an amino-terminal epsin domain. RSD-6 is characterised by a carboxy-terminal tudor domain. As before, these specific domain topologies were required of any putative orthologs identified. Minimally, one matched domain was required of short sequences. The RNA-dependent RNA polymerase proteins EGO-1 (Enhancer of GLP One), RRF-1 (RNA-dependent-RNA polymerase Family member 1) and RRF-3 are each characterised by a single RNA-dependent RNA polymerase domain. The nonsense-mediated decay protein SMG-2 (Supressor with Morphological effect on Genitalia 2) contains an amino-terminal zinc-binding helicase domain and a type III restriction enzyme-like domain. SMG-5 is characterised by a carboxy-terminal PIN-like RNase domain, and SMG-6 by a carboxy-terminal PIN-like RNase domain and amino-terminal telomerase-interacting domain. In all cases, the same domain topology was required of putative orthologs. In cases of short sequences, matching of one domain was minimally required.

**Shared protein domains**

None of the above domains are shared with members of other protein families considered here.

***AMPLIFICATION PROTEINS***

**RSD**-**3**

***Ancylostoma caninum* RSD**-**3**

LWKRMLEDNKTAWRRVYKSLTLTDHLLFHGSERVIGNARDHTFQMRVLEHYKYVDDRGRDQGLNVRHRVKLILELLGDDDKLRAQRKKVKSDNKDRYQGYTSEDIRMGRGGSYNSSSVGGFDDDWDDGANQRHKKNTSYGDYREDYTAKEVNSFQFPEEARRGSISPELGFRQDPISDDDFGDFATARSNPQPVPTARTTDITRVGAAHVPALRPPAPASSSTTTATTTSSASFDLLGLDAPSVPSDLTSPAKAIDLFSTPLTTFTSDSPVKPPGVFDSVKLTTGAPTATEPSVNDLLGASSSTIPVNNVFVADWTAAPSALQTGMPQPQSAFTAYASPASANPTGSTSDITCDFSAPLSPSRPQIIDSPGKSKTLADEKPTNIAKIGSTWAGASSLIDLDNLGSKNTPVKQGVSLNQMQLNKQCANKTSNAPW

***Ascaris suum* RSD**-**3**

MKRFYDATTNVSLAFAGLTLVHGMSYWFQQSLTLLNYLLKNGSERVVGSARDHLFEMRALENYRYTDERGKDQGLNVRHRAKLLIELIQDEEQLRVARKKAKMEGKEKYQGFSKDEMRMAIGSGSFGELNISTF

***Brugia malayi* RSD**-**3**

MEWASVDDENYLYCGLVDSRRFFFSIHFIILFVSTDKTRNSEAMSDLLSGIASFTRTVADSFNTYEIRKLGDKVQGIVMNYTEAESKVREATNEDPWGPTGPQMAEIAHMTYQYDAFPEVMSMLWKRMLQDNKNAWRRSLTLLHYLLKNGSERVVSNTRDHLFEMRTLESYKFIDEKGKDQGLNVRHRVSVLFDLIQDDEQLKTERKKAKLEGKEKYKGYSKDDMRLCGQITFSSNNTENFGNWRNSSDISKRNSCHEDGRDSYHREVNSFQFPDEEIIHGEGDSPELGIREHTLDPIDHEIEDDEEFGDFALARNLHSQSQKVSLPSLITAQPYPIHKPYQLHTVDVPIPAAALPPPPPSLPSFGPSAIATRVKHRDNGILLQNKPVSDLLGLDEFNDNGNSSNSSTSFFKDSSKTTTLQLSSLMKAMNQLTSTQQLTSQLRFDSLSSESVSDAAFSNANQQGVSSAARSVGLADSFTVPSVAAPVQATFPSLLSSSAAFSPCWGDVSAAHVSLANSTVQSSLQNTVLLAEATPLVPVPTHSVSVIPSHVNVPSLQVDQDSKSAVKIASTWADASSKVNINLDDLGMKKKPQKHSLPMNQMTPSMSVPATDRDILGASTVFTNASQERVSSDDMLDLLK

***Caenorhabditis brenneri* RSD**-**3**

MSDLLAGLTTSIKSTAKAISSNEYVRKVTESMNDAIMNYPKAIMEVREATNEDPWGPTGPQMKKIAEYTRSRYMEDFYNVYTPLFSRMLENNKDAWRRVYKSLVLLDYLLKNGSERFVQEAREKTYELRRLESYKYIDEKGRDQGINIRHRVKQILEMMNDDELLQAERKKANADDKSKYRGFDQYDMKMSSSSFKSSSSSSKFDDWNGGSSSSTTTKRYDDDGFKKKEVSNFSFGGNRSPSPELGFVDEKKKPDEDDGFGDFVSSRSSLPAATNKSTPNPPRGFFDDPVPAIPPPSATSNNYVAPPLSPVAKTGSSNVDLLFDVSSPTPAAAPQVASPQIDLFAVSVFYSKNQRIFKVPNAYETYSYRQLNAPVLNSAPAPAVAAASGGLDLFDFGAPPVAQPAVVTSPITTPATNGFNDFDFGASFGSTAPTSAPMQPMCAKPQQPPQHTANAGSGAPKVGNSFAGLDQFMSLSLGSTGSNAPKAKTLNQMKGN

***Caenorhabditis briggsae* RSD**-**3**

MSDLLAGLTTSIKSTANAISRNEYVRKVTESMNDAIMNYPKPHMEVREATNEDPWGPTGPQMKKIAEYTRSRYMEDFYNVYTPLFARMLENNKDAWRRVYKSLILLDYLLKHGSERFVQEAREKIYELRRLESYKYIDEKGKDQGINIRHRVKQILEMMNDDELLQAERKKANSDDKSKYRGYDKYDINSSGMKSSSSSNFDNSWDRNPSSTTSKRYDKEVSNFSFSSSTANRSPSPELGFVDDTTKKDTAANDDDGFGDFVSSRSSASSGSKVKAEPPKAARSFFDDPVPVIPPPSGAAQNSYVAPALSPVANKPASNFDLLFDVSAPAPTSAVTQNNGGNIDLFSQMCEPNFSTPKPTAAAPAASAPTAGGLDLFDFGPAPVAQPTVMMSPMNAAPAAPAANNGFSGFDFGPSFGGSTPLQPMGAMEATPPASQPAASQINLGGPSPKVGNSFAGLDQFMSLSLGSTNANTTKAKTLNQMKGNQ

***Caenorhabditis japonica* RSD**-**3**

MSDLFAGLTTSIKSTATAITRNEYVRKVAESMNDAIMNYPKAEMAVREATNEDPWGPTGPQMKKISEYTRSRYMEDFYNVYTPLFTRMLENNKDAWRRVYKSLVLLDYLLKNGSERFVTEAREKIFELRRLESYKYTDEKGKDQGINIRHRVKLILEMLNDDEKLHAERRKANTDDKSKYRGYDQYDIKMGAGGSYNKSSSYDNNSWDKERSSKGNGDFNNYKKDSREVSNFSFSSSNQGNRSPSPELGFTDDSAKKVNNDDDGFGDFVSSRSSQPAATSKSTPNNTVNSPASFFDAPPIIPPPSAQSNYAAPPLSPVAKQSNIDLLFDVAAPTPAVSSNVAPASGGQIDLFAQLSSPVANPAPIAKPTAAPASGGLDLFDFDAPVAQATAPIAPIAQLTQAAPVAASNGSFNGFDFNTSFGSSSAAPSFGNAAALQPGIPQQAQPQKQQDNGGPSPKVGNSFAGLDQFMSLSLGSTNSNAPKAKTLNQMMSGGH

***Caenorhabditis remanei* RSD**-**3**

MSDLFSGLTETIKSTANAITKNEHVRKFAESMNDAIMNYPKAQMDVREATNEDPWGPTGPQMKKIAEYTRSRYMEDFYNVYTPLFARMLENNKDAWRRVYKSLILLDYLLKNGSERFVQEAREKTYELRRLESYKYIDEKGKDQGINIRHRVKQILELLNDDDLLQAERKKANSDDKSKYKGYDKYDINNSGMKSSSSSNFDNNWDRGGSSSSSTNKKWEDDFKSKEVSNFSFRSSTNNRSPSPELGFVDEKKQKEVEDDGFGDFVSSRSSNPAAKSRSTPNPPKSFFDSSVPAIPPPSAANYVAPPLSPIANKKTSNVDLLFDLDAPTPSASATTTSGSVDLLSNMSFPVTNAPAPNVPAASADSLFDFGAPPVAQQPVSQMVAPIAPKTTNPNGAIGFSGFDFTSAVTTSSIPPFAPLQAMSGQQQQPTQAANQINLGGPAPKVGNSFAGLDQFMGLSLGSTNANAPKAKTLNQMKGGPQ

***Haemonchus contortus* RSD**-**3**

ISRSSHTIVMNYTEAENLVYEATNEDPWGPTGSQMREIANYTRYDGFHQVTNLLWKRMLEDNRNAWRRVYK

***Meloidogyne hapla* RSD**-**3**

MSELLQGIASLTKSVQQTLNSYEVRKLGDKVQGYVMNFTETEQKVREATNEDPWGPTGPEMQEIASLTFQYDQFTEVMGMLWKRLLQSLILLNYLLKNGSERVIATARDHSFEMRALESYKCVDERGKDEGANVRHRVKLILELLNDDELLRNERRKAKADGREKYQGFSKDDMLYRGGSNSSKFDSFERWNEKKGEKDEQEKKQKEWKGTPRSSVRREVTAFDFDTADNSRSRASGSPELGIRERTPEPLDEAEDEEFGDFTSARAAVPTNGKQSIPTFSSTKNISLASKPVDLAFKKPSGGSGDVDLLGLDLIVDVTNKTTKTQNFGNFDSFPSKNQSESNIIFIERPKSQQQTQPQTQPPISPQNVNFIQSTPSIPSAAPELLDLFSSTTQPTIQNQFVPAAPIHPINQIDDLLNSVQIPQQNASNISSSPAQPTITKNVQNQPKLPKTWEDFKGKVNIDFDNLSLKSPVKATPTLNELKKSSSGGQNISSSSGIGQWQ

***Meloidogyne incognita* RSD**-**3**

MSELLQGIASLTKSVQQTLNSYEVRKLGDKVQGYVMNFTETEQKVREATNEDPWGPTGPEMQEIASLTFQYDQFTEVMGMLWKRLLQDNKMAWRRVYKSLILLNYLLKNGSERVISTARDHSFEMRALESYKCVDERGKDEGANVRHRVKLILELLNDDELLRNERRKAKADGREKYQGFSKDDMLYRGGSNSSKFDSFERWNEKKVEKDDREDKQKEWKSTARSGSNSARREVTAFDFDTADNSRSRASGSPELGIRERTPEPLDEAEDEEFGDFTSARAAVPTNGKQTIPTFSPPKNISLANSKPVGGVGGGDVDLLGLDLIGDX

***Oesophagostomum dentatum* RSD**-**3**

LLWKRMLEDNKTAWRRVYKSLTLLNHLLLHGSERVIGSARDHTFQMRVLEQYKYVDDRGRDQGLNVRHRVKLILDLLGDDDKLRAQRKKVKSDNKDRYQGYTSEDIRMGRGGSYNGSSSYGYSDDWKDDSNRSYNRRPSFEENRDDYATKEVNSFQFPEEGRRGSASPELGFRQDPVDDDDFGDFATARSISQTTTPQKPAAARAGAANIPAIRPPGSATSPVTATNTTSSASFDLLGLDTSSSALGAPSPVKPMDAFGEPYKAFASPAPSLNLASPSSAVEKSTPADLISTTDLFATTSSSSKSLAADPFPADWAFVPPVPQPGAAPAVSPQSGAQASTGFISPTPVFPANKQQTTNADDIFGSFAAPLKPDRLPVTESPAKSATPAPTNGGSPSVTKIGSTWAGATGLIDLDNLAAKNTPTKQGPTLNQMQMSKHNAGI

***Pristionchus pacificus* RSD**-**3**

MSELLSGIGNLTKSLTSNISTYEIRKLSEKVQGMVMNYTEAEQMVRDATNEDPWGPTGPQMKEIAHLTYQYDNFHQVMNLLWKRMFEDNKYAWRRVYKPPEIINSR

***Trichinella spiralis* RSD**-**3**

MNYTEAESKVREATSDERWGPTGSMMADIARYTNAYDQFNEVMAMLWRRLFQESRKNWVRPYKCLILLEYLIKHGSEKVINDARDRMFELRILESYQYNDDPTCDHGQKVRSRVKAIIELLQDDDRLYEERKAARHNKTQYIGISSSEYSQGGFHNYRRRRDSSEDRVVESNASRRVENRHAIATVDPIEECTVGSDRNSDLLADVFAKPSTTVGVDNSWPASDDPFAAPQSSAGGNFKYANKLPDTWSDIGKIGLNLDSLSLSDRKDKPNRVTMNEMQNQKKQAAFDKQSSNSTSTVEVSCWFQPLSVELCNRI

**SMG**-**2**

***Ancylostoma caninum* SMG**-**2**

EKIHRTGLRLCDFMKXXLWTQMSNSXLHAQLKALKESAELQKLQQLKEEIGELSAADQERYINLKKMSEHKLLAAADVICCTCSSAADARLSRIKSNAXWLTSPLSYGTRGSRFDVRGVRQLICRXSCQLGPVILCKKASKAGLSQSLFERLVLLGNRPIRLQVQYRMHPELSAFPSNVFYEGSLQNGVTQTERQLRGVDWEWPVPGRPMIFWSCYGQEEMSASGTSFLNRTEAANVEKLASKLIRGGMRPEQIGIITPYEGQRSYIVQYMHTQGTLNSKLYENMEIANVDAFQGREKDLIIVTCVRSNDHSGIGFLNDPRRLNVALTRAKYGLIIIGNAKVLARQPLWNDLLTTFNQKNVIVEGPINNLKSVQITLPKPKPQRANPAYPTDRYGIQRATYTLREYRGGYAREAPAMDPHSAISAHSLRHAANLPVPLHMLQMFPPPPFPQQPPQQRFPSGRRNVPAWPPTPQSMAAGPVRMGQSQNDYGGMYASQASQDPLIGVDAYGDVSMSGWTQSQSQNVASVSMQSQRPVTAVSQQDDYRHIAFSQDIENDMANLLLSQGP

***Ascaris suum* SMG**-**2**

MMFWSCYGQEELSPSGTSYLNRTEAANVEKVATRFLKAGLRPEQIGIITPYEGQRSYIVQFMQTQGALHSKLYLEMEVANVDAFQDCCDGIYDVKCLADRGREKDIIIVTCVRSNEHQGIGFLNDSRRLNVALTRAKYGVIIIGNAKILSRHPLWNQLLTMFKEKNCLVEGPLNNLKPSPITLSKPRGVNSSIGHLFSTSFQCIKDLHSITYKIVREISDEPFYSSGDMEREGGDDSGIRVRSKQEWDTKRCTRSAGSAGYNFTGKASCAEWHKHTSSDTDVHATTTNFEYTLLSATSTTPQFVLYKLAGIIGQL

***Brugia malayi* SMG**-**2**

MVDSLEDEYGTPSLSLNFVDTPDGDSSLLHGATQDSQFEFDQQFTLPSQGWTSTLPEVGGDQSQELVFVDEEEFSVPGQLPTHACRYCGIHDPATVAMCMVCEKWFCNGRGSTSAGHIVIHLVRSQHKEVSLHKEGALGETVLECYQCGSKNIFMLGYIPAKADTVVVVLCRSPCANQAVLKDHSWQVDEWKPLISDRQLLSWLVKIPSEQEQLRARQISAVQINRLEELWKEDPKAVFEDLERPGMDEEPEHVQLKYEDAYQYKRVFEPLVKAEADYDRREKESQTQSVGHVRWDIGLNRKPQAFFQLPKFSEGSMKLMLGDELRLKHSQTAGGEWSSVGCVIKIPDNHNDEVGIEMRLKAENVPTDTRTNFTCEFVWNSTSFERMQAALSLLGQDEDCVSQFIYHKLMGHDIDDIIFKVSLPKRFSVPGLPELNHSQVHAVKTVLQRPLSLIQGPPGTGKTVTSATIVYHLVKQTNGQVLVCAPSNIAVDQLAEKIHRTGLKVVRLCAKSRETLDSPVAFLALHNQLRALHGAAELHKLQQLKEEMGELADADERRFRALRIAKECQLLTAADVICCTCVSAADSRLSHMRIKCVLIDESTQATEPEVMVAVVCGVRQLVLVGDHCQLGPVIMCKKAAKAGLSQSLFERLVLLGNRPIRLQVQYRMHPALSSFPSNVFYEGSLQNGVTEGKHLSMKRKEVINLSHMCFISGERQLIGIDWQWPVPDKPMMFWSCYGQEELSSSGTSFLNRTEAANVEKLATRFLKAGLKPEQIGIITPYEGQRSYIVQFMQTQGALHSKLYLEMEVANVDAFQGREKDIIIVTCVRSNDHQGIGFLNDSRRLNVALTRAKFGLIIVGNAKVLSRHPLWNYLLSMFKEKGCLVEGPLNNLKPSPITLSKPRRIPNIMSMNRFIPRGVVLAKDMQGNAFGTRNRDLRSIQDPFAAITHGQLRPQNGINIPVPIQMFVQPPPHYYPLHTRHGDLNSVDSTTRNRKHLEGINMFSSQQSQDPIYMQQLGEMSGISQDVSDWTQSQTQHGASQGGVPYSQDQLLVSQMESLFLSQDANAENYNFGDQAVFRLITTNIESFEKALWNELFEAENEVFLLIAENFLL

***Caenorhabditis brenneri* SMG**-**2 (Likely exon**-**intron boundary issue with predicted protein)**

LSPYCNLMRTKNRSNTIILQKLPVFILTDPKIAIKSDFGKKNNSSMSSLKIETFQIPAVKRRTGLAAISEKELEKMLNFFTFFCHFRHP*MRKKMFFNLFFSPLHEYCHGPWHPFLFFLCYFPQF*HYFSSAMVDTDECGMSAATQDSQFYDNQFSVPTQSSQVKKTNISNFVRKIIFQVTDFLPGVDATSELTFHDVEDESEDEKSLTDDQQKLPEHACKYCGNPTKTTVFLFFVKKRKGFRREFQLFQFEMPEI*RYLWRFQLKN*QF*VFF*SKFEHFEGNCYESCYEIYCYVGTASVSTAIFSSSAMDQLLEP*QLRHQKWLK*QLIHSRRAKYSS*ERAVTT*QLSINPIFGVFLAQKLCSNQLFFSAAQAVRLEELWREHPDATLDDLNKPGLDREPDHVQLKYTDAHHYSKIFRPLVAIEAEYDRRMKEQASQAVGTVRWEQGLRGSVCAFFHLPQFADGVMKLAKGDELRLKHNQTVDGSEWAKVGNVFKIPDNHSEEIGIEIRGQVDRSVQESRIMFTVDVVWNATTFDRQYRALQALQTDNQSVSPYLYAKLLGKPVDELMLKFELPRRLSAPGLPDLNSSQMQAVKQVLTRPLSLIQGPPGTGKTVVSATIVYHLVKKTEGNVLVCSPSNIAVDHLAEKIHKTGLKVVRLTAKSREHTDTTIPYLTLQHQLKVMAGPELRKLIQLHPVLSEFPSNAFYDGSLQNGVTESKFFMSYGSATFRNRDIRNRAVSKPRHFETQTFRNPDISKPRHFETQTFLNRDISEPRHFETQTF*NRDKKFRDVAVTKCPVYKFPGFGMSRFRDVSVSKYRSFEMSRSQFSMSYDF*NNSIFPDDRYMKGVDWHWPTHNKPAFFWHCSGAEELSASGTSFLNRTEAANVEKLVAKTAKFRVKLLILVQRSFSQKKNNRFRHKKTIFSKIWFFQLFFGLKFDLFILKNILKCYVVTASVSTAIFD*KLSKFV*K*LKFG*KLVST**K*SLFSR*MI*LYVLTSDQKDLNSTENSLNLVENRPNSS*KWCKFS*KLSSSAQNWSILGEKSTCFYCE

***Caenorhabditis briggsae* SMG**-**2**

MDDSDDDYVKSQDEILTFVDTDDCAMSAATQDSQFDLDNQFSVPTQSSQTTDLLQGTDGTSDLPFHDVEDESDSEKSLTEDQAKLPEHACRYCGISDPLCVAKCTVCMKWFCNSNDGTPGGHIVHHMVRSQHKEAYTHKDSPCGDTQLECYRCASKNVFNLGFIPGKKDQVVVIICRTPCANLAFQNDDNWSPEDWKSVIAEKQLLSWIVNVPSEEQVARARKITAQQAIRLEELWREHPEATLDDLNRPGLDREPDHVQLKYMDAHQYAKIFRPLVAIEAEYDRRMKESASQAVGTVRWEHGLRQSVLAFFHLPQFADGVMKLAKGDELRLKHNQTVDGSEWSRIGSVMKIPDNHSEEVGIEIRGQVDRSVMESRIMFTVDVVWNATTFHRQYNALDALVNDQKSVSPYLYHKLLGKPFDEMMLKFELPRRLSAPGLPDLNSSQSQAVKQVLTRPLSLIQGPPGTGKTVVSATIVYHLVQKTEGNVLVCSPSNIAVDHLAEKIHKTGLKVVRLCARSREHSETTVPHLTLQHQLKVLGGPELKKLIQLKEEIGELESKDDLRYRQLKRVKEHELLAAADVICCTCSSAADARLSKIRTRTVLIDESTQATEPEILVSIVRGVRQLVLVGDHCQLGPVVICKKAAIAGLSQSLFERLVLLGIRPFRLQVQYRMHPVLSEFPSNAFYDGSLQNGVTENERQMKGIDWSWPTPSKPAFFWHCSGAEELSASGTSFLNRTEAANVEKLVSKLIKGGVEPRQIGVITPYEGQRSFIVNYMQTQGTLNSKLYEGVEIASVDAFQGREKDYIIVTCVRSNDILGIGFLSDPRRLNVAITRAKYGIVVVGNAKVLSRHELWYELINHYKKKDMLYEGPISALKPFTMTLPKPIQKAKNNIAGANNRFGIKRMQYTYNEYKVVDPSQPRLPPTYANTQNLLSMSKSSRNFNQNVPVPAYMMDPLIYAARGQKDRRREQRRQQAEAMDFSQDMSQSQANYGPGASQSQSQSQYMDGSSLSAWSQSQTRPRRGYQGSTQQMSQDMDDMEQKMSDLLMSQDC

***Caenorhabditis japonica* SMG**-**2**

MDDFEAYGEEGAEKLTFIDTDEDGIIAATQDSQFDFGSQFSVPTQSSQATDIVPGVDSSADLPFHDVADESDSDKSSYEDGPKKLPEHACRYCGISDPVCVAKCTVCNKWFCNSSSGTSGSHIVHHMVRSQHREVFVCKAFTHKDSPCGDTQLECFRFSSTQAVRLEELWRDRPEATLDDLNKPGLDSEPDHVQLRYVDAHHYCKTFGPLVQIEADYDRVLKESASQAVGTVRWEQGLRQTTLAFFHLPKLSDGCMKLMKGDELKLTHNQTVDGSEWNAIGSVFKIPDNHSDEVGIEIRKKVEKSVMENRIMFTVEFVWNGTAFQRQYQALGNLMRDSKSLSQYLFQKIMGQQTEDLMFKFDLPRRFSVPGLPDLNSSQMQAVKQVLTRPLSLIQGPPGTGKTVVSASIVYHLVQKTEGTVLVCSPSNIAVDHLAEKIHKTGLKVVRLCAKSREHTETTVQYLTLQHQMKVMAGPELKKLIQLKEEIGELELRDDARYAQLKRVKEHELLAAADVICCTCSSAADTRLSQIRTRTVLIDESTQATEPEILVAIVRGVRQLILVGDHCQLGPVVLCKKAAIAGLSQSLFERLILLGIRPFRLQVQYRMHPVLSEFPSNAFYDGSLQNGVTESKSIFCGLKYAIAQKIKFFLRFKKKIFYNKTKKAEVQKSVPLQEKKHVRKVKKNDRRMTGIDWHWPRLDKPAFFWHCCGSEELSSSGTSFLNRTEAANVEKLVSKLIKAGVEPSQIGVITPYEGQRSFIVNYMNTQGTLNSKLYEHVEIASVDAFQGREKDYIIVTCVRSNNVLGIGFLSDPRRLNVAITRAKYGLVVVGNAKVLARHELWYELINHYKGKELLYEGPISALKQTDLTLAKPTIKAKNKIVGNMNRFGVRRMQYTYNEYKSTDPSQPRLPPTYANSQNLLSTSKFARNVNSSIPVPAHMIDPMVYAPRGQKERRRDPHRQVAETMEFSQDMSQSQSQFPPISQSQYSLDGNSLSGWSQSQNGEGGTSSKYQRNYHGNTQPMSQDTDDVEQRMAALLLSQDI

***Caenorhabditis remanei* SMG**-**2**

MDDSDDDYGKSGGETLTFVDTDEMGISATTQDSQFDFDNQFSVPTQSSQATDLLPSTDNTSDLPFLDVEESDSEKSLTEEQQNLPEHACRYCGISDPLCVAKCTVCNKWFCNSNDGTPGGHIVHHMVRSQHKEAYTHKDSPCGDTQLECYRCGSKNVFNLGFIPGKKDQVVVIICRTPCANVAFQNDDNWSPEDWKSVIAEKQLLSWIVNVPSEDQVARARKITATQAVRLEELWRDHPEATIDDLNKPGLDREPDHVQLKYMDAHQYSKIFRPLVAIEAEYDRRMKESASQAVGTVRWEQGLRQSVLAFFHLPQFADGVMKLAKGDELRLKHSQTVDGSEWTKTGSVMKIPDNHSEEVGIEIRGVVERSVMESRIMFTVDVVWNATTFDRQYRALHALLNDPKAVSPYLYHKLLGKPVDEMMLKFELPRRLSAPGLPDLNSSQMQAVKQVLTRPLSLIQGPPGTGKTVVSATIVYHLVQKTDGNVLVCSPSNIAVDHLAEKIHKTGLKVVRLCARSREHTETTVPYLTLQHQMKVMGGPELQKLIQLKEEIGELESKDDSRFLQLKRVKEHELLAAADVICCTCSSAADARLTKIRTRTVLIDESTQATEPEILVSIVRGVRQLVLVGDHCQLGPVVICKKAAMAGLSQSLFERLVLLGIRPFRLQVQYRMHPVLSEFPSNAFYDGSLQNGVTESELELFFVYLRPIFADDRQMKGVDWHWPTPNKPAFFWHCSGAEELSSSGTSFLNRTEAANVEKLVSKLIKGGVQPNQIGVITPYEGQRSFIVNYMHTQGTLNSKLYESVEIASVDAFQGREKDYIIVTCVRSNDILGIGFLNDPRRLNVAITRAKYGIVVVGNAKVLARHELWYELINHYKKKDMLYEGPINALKVLNMTLPKPTLKTQNKIAGNRFGIKRMQYTYNEYKASDPSQPRLPPTYANSQNLLSMSKLARNFNQNVPVPAHMMDPLIYAGRGQKDRRREQRRQQSEAMDFSQDMSQSQSNFGPETSQSQSQYHDGASLSGWSQSQSTAPFSKNRRPQYHGNTQQMSQDMDDMEQKMNELLLSQDC

***Haemonchus contortus* SMG**-**2**

KLGESALLSFMNFFKGREKDLIIVTCVRSNDHSGIGFLNDPRRLNVALTRAKYGLIIIGNAKVSTYKISLSFLNENVGTSKHMLHLGRGGMRPEQIGIITPYEGQRSYIVQYMHTQGTLNSKLYENMEIANVDAFQERQLRGVDWEWPVPGRXMIFWSCYGQEEMSASGTSFLNRTEAANVEKLASKLIRRYEDAFHYRRIFAPLVREEAEFDRKTKESQTQSVGHVRWDQGLNKKHLAFFHLPKFMEGRYCGIYESSCVAMCTVCNRWFCNGKGSTSGSHLITHLVRSQHKEALNFVLQVQQPKRLSAPGLPELNHSQMHAVKTVLMRPLSLIQGPPGTGKHRESPLGETQLECYQCASRNVFMLGFIPAKADSLVVRLFRAAQASRLEDLWKEHPKATVEDLDRPGVDTEPESVLLRYLDFDEVYGGGVGETLTFVETVDDDGVLHGGTQDSTFAFDHHFSIPTQSSQLYSFQAVWNSTSFDRMYQALNTLEKDPHCVSQYIFHKLMGHDIDEILFKMKLMIGDELRLKHSQTIEREWQCLGQVFKIPDSQLLSWIVNIPSEQAQLRARHITFHIASDHSDEIGIEMRAAASEKMPTDPRINFTCEVQVLARQPLWNDLLTTFSQKNVIVEGPIDRYGIQRATYTLREYRGGYSRDAPPMDPHSAISAQSLRHAANLPVPLHMLQIEKLPEHSCRYGFS

***Meloidogyne hapla* SMG**-**2**

MVDSLLAAYGSSQNYQLTFVDTDEEFQAGVVTQQDASQGDFGGHLSAASQDDGASRGVPSTTVLSESQTEPDLNFQDDDEFGDYIPPHACKYCGLSDPPSVARMLSLWFEVSLYLSFTINIYKCRNIFLLGFIPSKTDSVVVILCRQPCAQQNTLKNANWSAEDWKPLIHERQVLSWLIKIPSQQEQLRARQITAALINRLEELWKDNPEATADDLERPGKYTSEAEHVLLRYDDASMYKRIFNPLISMEAEYDKKIKESISCPVGQVKWDVKSANKVHATFQLPGFRDGNMKLMIGDDLRLKHYQTLDGNHNEDFTLEMFASSNKIPTDKRTNFICEYVWNSTSFDRMFAALGRLESKENCVSQYIYHKLMGHDVDDILFKLVMPKKFSAPGLPELNHSQISAIKAALQRPLSLIQGPPGTGKTVTSATLVYHLVKQTSGQVLVCAPSNIAVDQLAEKIHKTGLKVIRFCAKGRETVDSSVAFLALHNQLKALQSGELFKLMKLKEEIGELSQADQNRFMSLRRQKENELLSKADVICCTCITSADVRLAKKEFRCVLIDESTQATEPEVMVSVVKGARQLVLVGDHCQLGPVIMCKKSANSGLGQSLFERLVILGNRPLRLQVQYRMHPVLSAFPSNVFYEGSLQNGVTEMERKLDAFSWQFPNPDKPMMFWNCNGQEELGSSGTSYLNRSEAVNVEKFVTRLLQAGFSPDQLGIITPYEGSLHSKLYLDIEVENVDAFQGREKDIIIVTCVRSNETGGIGFLNDPRRLNVALTRAKYGLIIIGNAKVLSRQSLWHHLLVTFKENECLFEGPLNNLKTSPITFPKPKPLTAQHMPGSRYVNRTLGFTMKELGIGPYAGIRSVPVVQQHPLQQFQDPQGMIYPNFPMGTRTLGSQLPVPIHMFSSFVPAPPQMPPMPPPYPVPLNSIHLPGVNNGMGGTTRKKMSKRSGQSQRSLNSSSSSNIINNFNSQASQEQFLPGFISQNSQLSQGGIYSDYDGSQLGASQSQQFSFQQPPLHSMQFSQQRELVEQQLQNLMLSQQSEARKH

***Meloidogyne incognita* SMG**-**2**

MVDSLLAAYGSSQNCQLSFVDTDEEFQAGVVTQQDASQNDFVAQLSVASQDDGVSRGVPSTTVLSESQTEPDLNFQDDDEFGDYIPPHACKYCGLSDPSSVGQCTVCGKWFCNGKGNTAGSHIINHLVRAQHKEVALHKDGPLGETQLECYHCGSRNVFLLGFIPSKTDSVVVILCRQPCAQQNTLKNASWSAEDWKPLIHERQVLSWLIKIPSQQEQLRARQITAALINRLEELWKDNPEATVDDLERPGKYTSEAEHVLLRYDDASVYKRIFNPLISMEAEYDKKIKESISCPVGQVKWEVKSATKVHATFQLPGFRDGNMKLMIGDDLRLKHYQTLDGSEWNCMGRLIKIPDNHNEDFSLEMFASSNKIPTDRRTNFICEYVWNSTSFDRMFAALGRLESKENCVSQYIYHKLMGHDVDDILFKLVMPKKFSAPGLPELNHSQISAIKAALQRPLSLIQVYKLNQIF

***Oesophagostomum dentatum* SMG**-**2**

MSEHKLLAAADVICCTCSSAADARLSRIKIKCVLVDESTQATEPEVLVSIVRGVRQLILVGDHCQLGPVILCKKASKAGLSQSLFERLVLLGNRPIRLQVQYRMHPELSSFPSNVFYEGSLQNGVTQTERQLRGVDWEWPVPGRPMIFWSCYGQEEMSASGTSFLNRTEAANVEKLASKLIRGGMRPEQIGIITPYEGQRSYIVQYMHTQGTLNSKLYENMEIANVDAFQGREKDLIIVTCVRSNDHSGIGFLNDPRRLNVALTRAKYGLIIIGNAKVLARQPLWNDLLTTFNQKNVIVEGPINNLKSVQITLPKPKPQRTNPAYPTDRYGIQRATYTLREYRGGYAREAPPMDPQSAISAHSLRHAANLPVPLHMLQMFPPPPFPQQPPQQRYSSGRRNAPAWPPTPQAMGGPSVRMGQSQNDFGGMYASQASQDPLIGVDAYGDVSMSGWTQSQSQNVASVSMQSQRPVTAVSQQDDYRHMAFSQDIENDMANLLLSQGP

***Pristionchus pacificus* SMG**-**2**

MAECAEEGSILARLGKQQCSYQLATSSMADLDDVYGDGGGALTFIETVDDCGDLPAATQDSQFDYRDFTAATQPTLPHHRHHSDHVGSGTGTQAQTDDLHFQDVDEDGAAMPVDLPPHACSYCGVHETASVAQCSVCKKWFCNGKGATTGAHLVHHMVRSQHKEMCLHKDNELGETQLECFQCGARNVFQLGFIPAKADYVLVILCRSPCAQQASQMGENWVSDDWTPLIQEKALLPWLVSVPTQSVQSRARQIKAAQIFKLEDVWKENPNATLEDIEKVGYTSELETVPLAYQDAFQYRRIFAALVEEEAEYDRKMKESQTQTVGHVRWDVGLNKKHIAYFKLPKFQEGSMKLMIGDELRLKHLQTMNGQPWEKTGQVFKIPDNHTDEIGLEIRAGYQERMPTDVRINYTCEVVWNGASFLRQTSALNLLQNDEKCVSPYIYHKLMGHDVDDIMFKIKLPGRFSAPGLPELNPSQIAAVRMVLPRPLSLIQGPPGTGKTVTSATIVYHLAKQTSGQVLVCAPSNVAVDQLAEKIHATGLKVVRVCAVSRETLATNIEFLTLHNQLKFVKGAAELKKLQQLKDEMGGLDEADLSRYTKLKRATLCTVMQEAELLMAADVICCTASTAADARVSKLRIKCVLVDESTQATEPEVLIPIVRGVRQLILVGDHCQLGPVVMSKKAAKAGLTQSLFERLVMLGNRPIRLQVQYRMHPVLSAFPSNVFYEGSLQNGVTEAERTLKGVDWRWPVPDRPMMFWSCYGKEEMSASGTSFLNRAEAANVEKIASRLIKGGMRPSQIGIVTPYEGQRAYIVQYMQTQGSLSTKLLLDLEIANVDAFQGREKDVIIVTCVRSNDNNGVGFLNDPRRLNVAITRAKYGLVVIGNAKVLSSQPLWHDLLSSFLERGLVVEGPLNNLTRSPLILPKRREDPQESHLHQRQHKPERMQYTLPEYKNRPVPMEMRTEEYKNRPVPMVMRTEPTNVDGDEGW

***Trichinella spiralis* SMG**-**2**

MDVREQQRRKKNSPQSLSFYNTGQRMQMLDQTQDSELDYLDYSSDNDTTLKSVLKIATNAITQLSEEELPVVLPEHACRYCGYHEPSSVVLCNYCKKWFCNGRLTNSAGSHIVTHLVRSRHKEVTLHSMGPLGETVLECYNCGCRNVFLLGFVSATTESVVVLLCRQPCANQNSDHKQWKPLICDKAFISWLVSIPTLKEMKNTRRIAPADMVKLEELWKENPEGTLEDLENPQNEAEPEAVSLRYDNAEHYKSVFGPLIFMEADYECRLKEAQSQQNITVRWDVGLNKKRIAYFSLNKISDTGRVSHKSYMQHLNVCEVKLMTGDELRLNSNFDGKVWRASGHVIKVPDNFGEEVGIELINAHDAPPPDVTYGYSVDFVWKSTSFDRMFIALRKLTDDGFVSHAIHRKILGHDYEAPPLNILYPKHYSAPGLPELNHSQVMAVREVLTRSISLIQGPPGTGKTVTSASIVYHLAKAGGTPILVCAPSNVAVIRMCAKSREAIDSPVSFLALHNQVRYLEGEEELDKLWQLKEETGELSSLDEYRFRFLRSKCERDLLKHADVVCCTCVAAGDPRFNHIRFRAVLIDESTQATEPECLIPIMTGARQVILVGDHCQLGPVVMCKKAARAGLNQSLFERLVILGNRPIRLQVQYRMHPLLSSLPSNLFYEGTLQNGVTEQERILEGVDFRWPNPTVPMFFWCTASQEEISSSGTSFLNRAEAAHIEKIATKFLRSGIRADQIGIITPYEGQRAYIVQHMLLSGPLNNKLYQEIEVASVDAFQGREKDIILLSCVRSNEHSGIGFLNDPRRLNVALTRARYGLIIVGNPKVLSRQPMWHSLLRFCRENHCLLDGPLNALKEYKVDFNKGKSNLPVMKTITVKDMLLNSRPKVSSSYRDELSLISVGRSQAASSLLNSFDPPIPYNMFMLPTSSQRNAKEGGEMRDRRRRKMRSASGRRLVPSDEASASSSQSMAFQSQSDFTQEVEVDESLRIQLESMMLSQDTNYEHSEEVSSSSSMDDGSSSGFYPASQQ

**SMG**-**6**

***Ancylostoma caninum* SMG**-**6**

DLEIRRRIFYCVRALSAGHPLEAARDRLYARLAAMKRKVDKYEPLLDTECGEVHEDAELAASADRPYEVWLDMEGPNIEAADDDAHVFRSFLEQQPSKLHRRAITYVISTVGLLITKIGMESFPSVSERAIGQLAALIEQDASPVTAGQLIQIASLFIYAVHCNGIVGEADVCSVQQQQAVRALVSVFGAYLRPIVIRLDEVRSWIQGTSPVPRVVSRVLPAICVLCEWFSCPLASAIYRTMPSIEALPLSVVDIDTWHFLAKIANELMRWQDTNMLAKIENGGASNKSFILPELAYMSSFSSVFPAFPRILHCTLPATDSVEQLIPLHVRLAQLLLAAEYLDGSDMSCFFFCEKTGKFTRSERVDGEHGEHPDVSSNQRDEESENSLPPLTREELLEREIRKHEQLIVVKPLYVVIDTNAFIDQLEAIQKIQQCERFRLLIPTTVMEELMELQHGEINSTRAEAATAGARQAMVWLREQTRQKCPRMFTLTMRGRRVPIAVVREESADDREMVNDDRILRSCVNFTQLEPAQESTFSEFKKPQGRDTPIIYRNVILLTDDRVLNMKAMSEHIPCRTMMRFMKWAKIS

***Ascaris suum* SMG**-**6**

MQWSLKSSYPYPSSANGQWSKSAASVLEDYQARFISALSELFTSIVTRDIHYTYAMNLEQHMWKQCFYKPIEALRTASNSSSERSQMLRSILKRFLQQVFCLISFITCTYFHVGNNSNKGLDFYAHLLNKYEVVFGFLIEDFLYWPSALPGDDFAACTCVGCGAHEFSDQSLKVAVMSAQRLAVSMGDLHRYNAMVCGVKDYSSSRGWYLKAAQLAPGNGRSYNQLALLAVYAVSVRSTLQLT

***Brugia malayi* SMG**-**6**

MSKDSPKTTRNDRTRKVRPTMQLYCPRMLRTAAVDRKVDSREKNANTEHLAKSVNTVKDYVNLNSHVDSLLRNNNCSLNGGTYKVGENSSESLRKSSNSINKFYDHLADNFGSPKRRPGRRKYSSSNNCYSSESLCEGCSTDNEIYHCNGPASSQIGSKVSSRNQKETFDQNSALPGGPLYADGTGLSRTSFDRRSPLNRQRDWSFRSNQSEDFKHCRHRNNKNSCIDRTSNIPQDNQDLYASLDSIDLASFDWSSEVEAEERRKKEREEEGRTAEKHPSQTNPSGSQNKRSRRALESLSKFQNRSGASNGSNGFSRLYSRNVKLPSVGRGRRFTDLSRQRNDSFTSQASSIAESIEEQSDVESGSGERTPTNNDRKTTLNFSTQDNENKVESSNLNVNQVPRSDDSTINNTHRDGHGNRGRSKRENRWKMRKQHNLGEIEVVVERRSTTSQYTRNNTRTSNRDSDLSAPSSNILKATSSAPIKGISGLKNVASRNFREEEKYCDVPSLSIGSATLLAELSNEPLKASESSLGRTKSNRFRRELPPTEWPIHLEISCKEGPQIQILNDTINQLIERITEFADLSAGEEILVASESLSELYLSIVTRNISYTYTMNLEQHLWKQCFYTSIEALRAASNSTGNSSRIFRTNLSKLIQQGLAFYALLLNKYEVAFGFIIDDYLYWPSNLPSDDFAGCVLTDSAIHEATDHIIKVALLSAQRLAVSVGDLHRYSSMVSGIKDYSHARVWYQKAAQLAAGNGRSYNQLALLAVYESKWIDVIFYYVRALAARYPFETARQPLFTAFSHVQKKVSEFEIEFSARVGDTAIREAEAAAARTDRPQEIWIAQDGKLSSRNYADITDHRAIHALRSVSTVELFRQAVPYLLHTAGLLITKIGMEEYDSISERALFQLSQLISRDECPLSSLHLIQLCTLFLYAVHSLTLKNSEPGTCSLQQQQAVQMVLSLFGVILKPVYDVLSDLSKILNGSVRLPSKTRRVLPAVFVISEWLSMPSVNRLYRSMPSLESIQTSLIQVDTWKLFADVANTLVNAESRGILSRTVTQSDGKSEESVEIILPEAVFLASFIDVFTVLPKSLQMLSLENIDLRNNLSLLALHARLSSLLSTAEYLDGSELPCFGFDEYLRCFAATRCTSPVTVQSTETRKSIANFDRQFDNLEFSHEAMEQDLSHFSDEYKHYRQTVRENQVKQKLLERETMGNRVIIEVRPKYLVPDTNTFIDHLASIRKIVESHRFTVLVPTTVFSELENLSRLSPSPKPAVGVLGDFQDYWVGERAKEAVAYLKDLVGKQVPQVYTITSKGNLLTSLMFATEETCASGELKAVNDDFILSSCVNFAKTRAKPPNAIATAQSSSISMMDTEQLTKLYRNVVLLTEDRALNIKAICENIPCRTVPSFMKWASLR

***Caenorhabditis brenneri* SMG**-**6**

MANRAELHRRRQEEIDRNEGKRIREIGEKMDKLSGQVKKRDLKAAEKISEFSLELANIYSQIILQDIIYSFTAGLEQKLFRQAFYKSIECLRTGSNSTSPDARLIRAATQKLLLNGILFYEQLISTYEKNFEIELSETLTWQTGFPTDEELCSDGVELPLGIQKYENSAQKTALKSLSRHLISLGDLHRYKSLIDGSENYETSKSCYQKSSQLWPSTGHPYNQLGIVVYYSMLYRSARRARLVPVDVLSKQRQKRVIDEFFYLTRALACAHPYEAARDRLKQRLDAMRTKVSKYQPVLDKECGIVKEQGNVLRKLQHVRQIWIHPVTEALQDGTGEKAVNDVLSHFLEHSKAKLHRRAVSYLSDTFGLLVTKIGMEHFDSVSERAFGLLYASLAKSKTDFTPDQLVKLAALFIYSVHDNFEKASTSSSTFQLQLSVNTLFTYFLTLIEHISKHQTLLLPAINVLATWILNEETEEILKTIGSLEPLDSTILPGFSGFKQSFVSKETEKLPAETPETAYPEAILLASFFKIFEAIPVATCRSRLENLVLAIDKLRSYVKQEGDLEGNQRKFESLELEEPKSREQIVAEERGKGTGVIVIHPEYLIPDTNVFIGELELVKELLETAQKLKKFQILVPTIVLDELQCISKLSATDSKSNSEAHDPDRISKAKIAVAWLKEQSKAKTTNLHTLTSTGKRLQSLILAVEDLEGAPKITNDDMILNSALKWFESLPEPSSNSILQQVASGITSSES VFLRSCILVTGDRGLTIKAIGNNFPCRGIINFAKWMGV

***Caenorhabditis briggsae* SMG**-**6**

MVNRAEINRRRQEEIDKNEGLSIREIGERMDKLSGVKKRDLKSAERISEFSMELANIYSKLILEDVIYSFTAGLEQKLFRQAFYKSIECLRSGANSASPDARLIRAATQKLLLNGIVFYETLISKYEQQFQLTLADVLTWQSGFPTDEQLCSTALDLPVGIQKFETATQKTAIKSLSRHLISLGDLHRYKSLIDGSENYEISKSYYQKSSQLWPSTGHPYNQLGIVVYYSMLYRSARRARLVPVDVLSKQRQKRVIDEFFYLSRALACSHPYEAARDRLKQRLDAMRTKISKYQPVLDKECGIVKEQGNALRKLQMIQQIWIHPTTESTEYGTGEKVVDDVLSHFMTHSKAKLHRRAVSYLCDTFGLLFTKIGMEHFENVSERAFGLLYAALAKEEIDFEADQLVKLAVMFIFVVQTNFEKASTSSESTSQIQISANNLCTYFHILLEHMPRHRDLIFPAINVIATWIRHDATEEILKTLGHHLETLTSSILPNFQEFPGFPEDGIPELSENQVYPEAVLLASFFKIFDAVPTAATSSRIENLNLAVEKFRILEAKSENSEEKTSKIERNSEEPKSREKMIEEERDKGKGTILIHPEYLIPDTNVFVGELQLVKEILEMAQTAKKFQILVPTIVLDELQSISKLSPTDSKSQSEQHDPDRISKAKMAVNWMKEQSKLKTPNLYTLTTTGKRLASLLLAAEDLSGIGKMTNDDMILNSALRWSESLPLPSQTSVLQKEATTMNCVLITGDRGLTIKAIGNNLPCRGIANFVKWM

***Caenorhabditis japonica* SMG**-**6**

MPPREVLNRRRKEEIDRNEGVQIRQCAEKIEKLANLVKKRDVKAAESMVKISIELANIYQQVILPDVIYSFTAGLEQKLFRQAFYRTIEVLRSGANSADPDSKRIRAVLQKLLLNGILFYEQLIRQYEQEFHVELDTALTWPSGSPTSSELVENCINLPVGIQKFESAIQKTAIKSLSRHLISLGDFHRYKSLVDGSENYEVSKTYYQKSAQLWPATGHPYNQLGVVVYYSMLYRSARRARLIPVDVLSAQRQKRVIDEFFYLMRSLACTHPYEAARDRLTQRLDAMRAKVGKYQPVLDKECGKVKSDAATLKKNMRLIRQIWIHPISQKVEDGTGERIVDDILSHFQAQSKTKLHRRAVSYLCDVFGILVTKIGMDHFQSVSERAFGLLYASLSKPETDFSPDQLVKLAAMSIYAVQVNFTKSDNLQVQIALNTLITFYTILIQNYLQDTNKTLLLPAIHVLSIWICHPETQVLEKTERLESLPSTILTDSAKNTILAVLEQIPAEKTVIDATKTYPEAILMASFFKCFEANPTGATFTTSGDLNSARLQAMRKAAERVRELVENEKVEDPPASTDSERLREEPRSRQQIMEEEREKGTVTIVTLPEYLIPDTNVLIGDLQLLKDLLDAKMYQILVPTTVIDELSHLAQPSSSTSTSDDAHDPERFSKARQALVWLREQAKLRTPGLHTLTTTGKRLATLILASEEVEAEKNTNNDDKILKSAIEWTNQLPTPPTSSPLHTVATQFQQADGNTGGGVQLKSCVLLTGDRGLTIKAVGSHVPCRTVENFVKWAVPGAQ

***Haemonchus contortus* SMG**-**6 (Likely exon**-**intron boundary issue with predicted protein)**

QINDSITSLLTKVQQRDVAAAEKIVQLQVDKYEPLLDIECGEVREDAELAASADRPYEIWLLSSYC*PCSPIPPWDCSSTREHPPNFPFLDSHGS*WTKPNELLLVVFFKKKKNKENVHLVRSSRRDQEDFRL*VVNCSGVIQELSMENSTQSLYDPQQPEGYLGYRSQPLIFKGIVFYERLIALYEKELDVDVQKAALFQMLYRSRRRARNTPLEILSNKRQVKPVYLVIDTNAFIDQLSAIQKILQCERFRVLIPTTGLSVSDRAIAQLAALVEQEDSPVTAQQLVQIAALFIYAVHCNDGSSSNTTRTRKLRPEIQIYRPGQSDPINP*DIRIKVFVQDRLNVDTSAETRSVAGDAPQSPSMSYMQLCQSFESIGSFDWSREVESEYNDDRILRSCVNYTQLEPAPESLFSDVKVPQGREMPNIYRNLVLLTEDRVLNMKAMCQHIPCRTMVRFMKWQLHRRAISYVINTVGLLITKIGMETFQGHALDEIFFCIRALSAGHPFETARDRLHARLAAMKRKASARQAVTWLREQTRQKNPRLFTLTMRGRRLPIAVVREENERASMRAEGGQRRNVGNRRRNDSITSTQSECLPPYLRSAQLWPASGHCYNQLAVVAYFSVSYALKSCSRHLISLGDLKRYRTLVEGSEDYSGSRTDLSVSNLLI*FSGDLCEIYYDVMLRDIVFTFSMNLEQHLWKQAFYKPIEVFKTMANSPKDSSRTFRAQLLVLINKVRY

***Meloidogyne hapla* SMG**-**6**

MIKNDLENQKALRKQIIAPLQNAEKMEALLSTLQGYSRKLASGPNDKAGRGILRIRFLALCSSFNAYFIFSFELSQKYAEKLLSDFESCFRRNVEYELWKGCFYSPIEVLRKRSEDGGPHAELFKGFLVELIENAITFYENLLNNYEANFCIDFSNFYQPYETLLSLEKWDECAVVNGDLPVVDSMVLRSAQRLIICFADLYRYKAQISHKDALTYEKWIFELIEEESKNIRATTNNYESGPYSDILNKENKLSNNYREVWIHPSNESLVKVSHKNQQPVKLSIEDTLAQLFVGENGKNIQKKAICHLLHCAGVLISTIGLDQFKRSYELILNELLTLVHIDDTILSPAYLLKIIVLYTFPIHSPKPGFYSKGFI

***Meloidogyne incognita* SMG**-**6**

MIKNSQEDRKSAARKQVIPLQNAEKIEALLATLQGYSRKLASGPNEKAARGILRIRFFSFTFCFYFVFSFELSQEYAEKLLSDFESCFRMNVEYQLWKNCFYSPIEVLRKRSEDGGPHAGLFKGSLNELIEPAITFYEGLLKDYEVKFSVDFSKFYQPYASSLSLDKFDECAVIDSELPVVDSMVLRSAQRLIICFADLYRYKALSTHKEARTYSFAKSLYWQAHFLEPLNGHPFNQLAVVACYEKKWVDVLFYYIRALSVLLPFKSARESLELALNSLRQPANAYEKWVFELIEIENRNVQAATNNYESGPYSDILNKENKFHNVCREIWFHPSNDVLVEESLKKHQPMSTIEGTLTRLFVGENGNNVQKKAICHLLHCAGVLISTIGLDQFKQSYELILNELLALVDNDDTVLTPAYLLKIIILYTFPIHSPRPDASQVLFENQRCAAFELVFSSLKFLLEVFYKDMDKVGLFILTGQLSEKFSCIIPSICFLVFWLYRIDKCLILDDVLKHYRIFELLVLIGNRLNLLRQRRTLCDLRVFSDVGDPNKYFLQIQLPEFMLLTPYFDILSKSSVQFFIDVNSLDMEITDKDHKWIAVQARFALILRLCEYLSEGEYFPIKFDVNNITDGPFRLLPHHKGLIFFILQWIVLRNEEFG

***Oesophagostomum dentatum* SMG**-**6**

KWSEQAKSSSKIVLATFISMGDLKRYKTLVEGSEDYQDARKTYLQSSLLWPSSGHCYNQLAVVAYFSGHALDEIFFCVRALSACHPFEAARDRLYARLAAMKRKVDKYEPLLDAECGEVHEDAELAASADRPYEVWLDMEGPNIETEDDGAHIFRSFLEQQPSKLHRRTISYVISTVGLLITKIGMESFPSVSERAIAQLAALVEQDMSPVTSSQLIQIAALFIYAVHCNGIAGDADVCSVQQQQAVRALVSVFGAYLRPIVIRLDEVRSWIQGTAPVPSVVSRVLPAICVLCEWFSCPLASAIYRTMPSVEALPMTIVDIDTWBBFAKISNELMQWQDTGMLAKIENNGASTKSFILPELAYLASFSSVFPPFPRILRCTLPDMEPMEQLLPLHVRLAQLLLAAEYLDGSELSCFFFCEKTGKFTRSEHAEGEHRERPNNLYIHKDEELENQPVPLTREELLEREIRKHEQLLVVKPLYIVIDTNAFIDQLLAIQKIQQCERFRLLIPTTVMEELMELQHGEINSTHAEAATAGARQAVTWLREQTRQKTPNMFTLTMRGRRVPSLLSERSRMTTERW

***Pristionchus pacificus* SMG**-**6**

MHLLYPILTVIPSHSQLHRRTVSYTAYVHALLVCKIGMERFASAASRMFAQLSSIAYIGTDFFLPISAQLETRLPLVHSRELLHRPDCPMSAAQLVQCAALSIYAVHAASGSGSASATPTPHSRIAVRVLLTLLGVLLERVRADAPALARAAESGAPPPPAGARVLPALHVLAEWLASSVGAAHYAAAERLRPLELAMVPKAAKGVWEDLAEVANVVEGLEREGALKEAVENDGASDDEHQIVGLYKWNDLSSRVVLPESLLLSSFTSAFPSPPKHFSFMDKPESTTVAALHVRLRGILELARFLDGTDACAIVYSMERRRFESTDAFENNNEDEEAREIRRRKSEKDDDRSVSPEPVTDSLRRPIVVCPKFVVIDTNMYVDDLSDVKRILDSGRYQILVPTTVIDELLGLERGRGSVEDQREAAHAVKTVEKAKEALSWLREQTAKKQPKMGTLTLRGQRMAISLANEDADEETAKLVNDDRILEAAAKFTATLPTTASAAAAAAIPAAVAAARPLHRQLALITGDRGMNIKANARSIPTRKSSILFTSLCVLCVFTALLLVIQY

***Trichinella spiralis* SMG**-**6**

ISAYEDSFGKMKHQEKKKSRSKKDEMSSCFAQEVWYSFEGIRIKDYESGIERGRAIDEILISGSTDELYRRCVAYILHLNGILFTKIDVDTFSNVARTAVIQFVELLRRAESPLTATGLLKLILITLFTVQHIQMKMKQNKNNESCSVLLQLAVQLTLNIFTILLFFIEKQLEAILLGIDDPSALRMLPALHIMCRWFSLPSSLKLIQKMNSIGRIEFDGLEMKLWASFMDLCKMLLIAKTEGKLCFSESQEGDETIAVHLPEDVYLLLYLLILNSVVLNHLRLESVLKLGGHLNVNNIYINRDIGAVEAEPVDELSNLSKKEEEYDYELHIYPQRLVIDTNTFVDHLKLIIKLVKMKKYTILIPLVVFNELYGLGKYHESDWVQTQCTLAMKLIKEWLQDDSLHVKGVTTSGTLIKTFDFFNEATEKIQNDDKILHCCEILINNKSVSSITASCAHPEIVHRDIVLLTDDRNLRLKAYSMCIPARTLQIKNLFNAFSVCRIVDVCLLNYVERGNEAWTCFIHCRRRYAHKRANMSSDGHVYLQESTSAADQADRSTSVEVRDLDQLIRIAENLRSKRDALQKELEIRNMQLRQSSENVDCLARDLQACQNKLASAVNMVLPLRRQIIDLQNQLDANKCRTELFNLMKEIACWKAKYEAMESFLDEDRQQNEKLLLKYEELLAENSSQKVLINKLNKDVSVLKEENVNLQNDSDKWKLMFTTLKQLTESALQFGCLNYLSVTTDPSFWDEVVSSAFKGADVKVDEISAEQKAKDSLEEIIKRAAFSPK

**EGO**-**1**

***Ascaris suum* EGO**-**1**

MFGVVDETGILQYGQVFVQYTKSVENKTPGPNAAKVILKGTHSIVLIMCSAECLMIETNASTCGISRVLMTKNPSIVAGDARVFDAVDVPELRHLVDVVVFPQYGPRPHPDEMAGSDLDGDEYCVIWDEELLLDCNEEASDFTKHPESPMTSLKIKWLVRCV

***Brugia malayi* EGO**-**1**

MVPEYERHQMTGIKGWAEPLQIKIIILQNEQLIEELKNIIPKIGSTEVKNLRVGRDIIEHRVDIYDYGSPWVELHVDVMSSCWKRIFLKMVSMFQTLTSKFSGKHTPILQTTERGFFMDDCLSAELQVDLHSIHHGNLLDLGTFCSHFTREIPKGINGTKLSEYIKVLSNLSSRNVRSAMYVDFEHDRNIFTVRFAVLDGYKAREMNNTSNEKQKLVDSMFALKVNYTSLRRILVDTNQESCAARIYFHLNYPPEIRRFRQKMNVTQGPKVELISDRFRYYPEADYQKDIGLAITAINDSPIFCLQFTEMMDDNLLYRLLSRLHARVNLPIEFANVQFSYFPVDNYVPLPVRMIGCDYRKCATEEGISQNDQXXQPVEPKVDKAWSKKLKSLSFALEYLIAALLSRGAVVKDQLLMTLEVLERLINMIDEMKNIPPLISAFERIQNSIFVKKELLAEIHGRSADEGFQRVRKAVITPTRMLLVIPELLMGNRVLREFDESGDDALRIQFRDDDGAHLRRSRAGLYIIETTVHNSLLRGVYISDRHFVYLASSNSQMRDNGCYFFNDGDDGKVKMIRDKLGKFDHTNIPKLMSRMGQCFTQSKETDVTLKRRKYNKTHDVIGGKDSCGEPFIFSDGVGKLSEDFAEQIANDLGLAKCVPSCFQFRYRGLKGVLSVDPALRQRRLWAERNGLEDKHAKTAKVNDLDIIFRPSQDKFHAPRKEIIEIVKYSSPTSVYLNRPLINILDQVSDMQGFDVHSRIVNRIHGLLDRQLLKLSDALMSEDRCRERLAEFPRRINVQYLSVARGFTLTQEPFFHGLLIASVRFTLQKQLSKEHIQIPANLGRTMFGILDETGLLQYGQVFVQFTNDVFLKTPPKAAAKTIVKGFVLITKNPSIVAGDVRIFEAVDLPELRHLVDVVVFPQHGPRPHTDEMAGSDLDGDEYSVIWDDQMLFSHNEEPMDFSKLIRPPDILKEDEVVREMRRFYVEYVMQDSIGIIANAFLVNSDTFGIASDVCMSIAEKHSQSVDFPKTGQPPKPLVKEWSNGPDGKMIPPERPERWPDFMCKTHEPSYVSTRLVGQLFRRIRLIDDVLTITRAHETQSKVTVDPLLEYSGWESYENDATLNLIAYSAHIRALMDNYGIEDECQLYSGCFCKVRNRISDRDGDDMSQFNTSFIIERKLTNIFMCFRCNFFAEFGDFMSCTQQDDDLRNKDVIERRYCKYPTVEMKKKASAYYIVCYRKVMKEKGERLLSFPWIVWDILADIKKENAVLTSQNISTIDPLCELASNTDEIPSENSNECRDLNSLIGGIGKCFLQFLQYLSSRSFEKAKIFNFMKPNLGYHSILMRGQWLDLHKAAIITFYQIVLTNRFNEMSIFKREQLHSCIEAEPFTIELPEDLCSFDENLREKMMEYSNVRYLYLRRIPDRKNRVVVSAFGTLESLYRLRDILSVKPISTVQADSKKRSDMMLRLVYERIQELP

***Caenorhabditis brenneri* EGO**-**1**

MGDDGYRGWIKLEVPCSFPERQMNNIIRCHTSKLEPALNHMNMRIQTKGQVQVAEEQDCEPFYEANYEVVSDRFSHHLIVAIQNYLKDLAADHLMPFQRGNLVLHSSDFWSTELTCHLVDIPLAAFFFGNIQGGTFINHWEVSFWEDTLRRNRDRVKNIAPTVSDKIGMNQIKVEFEFDKVDFMTVHFKHKEADFEVMDKDQRKTRQTVTMYYQITVRRTSIRRVIVDPVVHDVNGTDRVRVHFELNCPVLIRRAFRTEKQEAEFRHVVPHYKRNLIINRGRTANQYPTAKAITDSPVFTIEFDSSVCMVEIYRLLSRLRVRTGVSIEFADIPSVDCLIWRENPYHRWTFSNGQHLSPTHFSAPIYRDFITTAFPRRHEVCGSREIDTNRERKFAITYLLECLISRGAVVKDQILLDEIIWHRFLEVILHYYTKDDKLCEAGLEDLVHMIDGRKRIGSLIKCFDRICQTRQKNSLVNGLTTEEMREGYQRVRKIIFTPTRVIYVAPETLMGNRVLRRYDHDGTRVLRITFRDDDNQKMRTNKTSTLLEKTVNNYLHSGITVAGRNFGYLGSSNSQMRDNGAYFMEKYSSSQCREYEAIHHVKPPITYNPKIQAARKNLGRFETIDNIPKMMARLGQCFTQSRLSGVNLDRCTYMTMYDLTGGKNIKRDEYTFSDGVGMMSLRFAQMVSQVMDFGKGVPSCFQFRFRGMKGVIAVEPLLDMIRKWSLEFALEKPSDDVAWGLNCVFRPSQIKFISKRHPRDQVEIVKYSSPVPVSLNKPFINILDQVSEMQSLECHRRVTNRIEELLDRQMLLFAQQMVDETFCRNRLKELPRRVDIDYLRTTWGFTLSSEPFFRSLIKASIKFSITKQLRKEQIPIPQDLGRSMLGVVDETGRLQYGQIFVQYTKNLALKLPPKNAARQVLTGTVLLTKNPCIVAGDVRIFEAVDIPELHHMCDVVVFPQHGPRPHPDEMAGSDLDGDEYSIIWDQQLLLDKNEDPFDFTSEKQKSSFKEEEIDDLMREFYVKYLKLDSVGTISNSHLHNSDQYGLNARVCMDLAKKNCQAVDFTKSGQPPDELERKWRKDEETGEMIPPERCERVPDYHMGNDHTPMYVSPRLCGKLFREFKAIDDVLKISEERDEQVEISIDENIMMDGYESYMHSARDELSRYNAQLRSMMENYGIKTEGEVFSGCIVDMRNRISDKDQDDMSFYNTNQMIETKVTNLFKKYREHFFEEFEGGWEANTESSSRIGSDANILQRQCRAPTQKMMRKAVAWYRACYEEARITRENKKLSFAWIAFDVIAMVKQTRSLASDDVQMGGANPLFTMLDDHRSQYLIDNEEAFETFSRFKTSKSAGEQVRRANGIIQMYVEMNPGLDEVLYILNEWATKSKLFDSQPLRNYHFSLLFLLFATRQISSVDGNAGAFFNKIDENNFKLSKESGAMVPNHELTNKQKSHMTVKFLEFLASRKFRKMANLSFRTLDFTSIFLRGEWQIFHVAALKTY YNVLFNLRFEELPVSTDPTMTIRSTIRENEPFVIELPAKCDQQLVHRKLMEHTGVEEIFMRCMEKSVRAENEPQKANVRLLVSTRGTLEAMYKLRQLVAVKVPIKTYVTGQEISSQMARLCYEKIIKGHINN

***Caenorhabditis briggsae* EGO**-**1**

MGDEGYRGWIKLEIPCFEPERHMNNIIRCHTTKLEPALNSYNMRIHTKGQVQAVEEQDCEPFYESNYEIVSSHFSHHLIIAIQNYLKSLSMDRLMPFQLGNLVLHSSDFWSSELTCHLADIPLSAFFFGNIQGGTFINHWEVSFWEDTHRRNRLRQKNMQPTASDRQGLNQIRVEFEFDKVDFMTVHFKHKEGDFEVISKDFRKTRETVTMYYQITVRRTSIRRVIVDPVVNDTNGGDRIRIHFELNCPVLIRRAYKTEKQEAEAKHQVPHYRRFLVINRGRSANMYPTPKAITDSPVFTIEFDTSVSLEEVYRLISRLRIRTGVSIEFATIPSVDCLIWRENPYHRWTFHGSSHLSPTHFSAPIYRDFITTAFPRKHEVCGSREVDTNRERKFAITYLLECLISRGAVVKDQILLDDTIWHRFLEVILHYYTKDDKLCEAGLEDLVHMIDGRKRIGSLIKCFDRICQTRQKNSLVNGLTTEEMREGYQRVRKIIFTPTRVIYVAPETLMGNRVLRRYDHDGTRVLRITFRDDDNQKMRTNKTSTLLEKTVNTYLMNGITVAGRNFGYLGSSNSQMRDNGAYFMEKYSSSQCREYEKMCRMAPPITFNPKIQAARKNLGRFETIDNIPKMMARLGQCFTQSRLSGVNLDRCTYVTMYDLTGGKNIKGDEYTFSDGVGMMSMNFAQMVSQVMDFGKGVPSCFQFRFRGMKGVIAVEPFLDDLRQWSIENDIQEVSEDTAWGLNCVFRPSQIKFISKRHPRDQVEIVKYSSPVPVSLNKPFINILDQVSEMQSLECHRRVTNRIEELLDRQMLSFAQQMIDETFCRNRLKELPRRIDIDYLRTTWGFTLSSEPFFRSLIKASIKFSITKQLRKEQIPIPADLGRSMLGVVDETGRLQYGQIFVQYTRNLALKLPPKSASRSVLTGTVLLTKNPCIVAGDVRIFEAVDIPELHHMCDVVVFPQHGPRPHPDEMAGSDLDGDEYSIIWDQQLLLDRNEDPFDFTSEKQKSSFKEEEIDDLMREFYVKYLKLDSVGTISNSHLHNSDQYGLNARVCMDLAKKNCQAVDFTKSGQPPEELERKWRKDEVTGEMIPPERAERVPDYHMGNDHTPMYVSPRLCGKLFREFKAIDDVLKISEERDEQVEITIDETIMVDGYKEYMHSAREDLARYNAQLRSMMENYGIKTEGEVFSGCIVDMRNRISDKDQDDMSFYNTNQMIETKVTNLYKKYREHFFEEFEGAWEANTEGTSRGESDTNILQRHCRAPTQNMMKKAVAWYRACYEEARVTRENKKLSFAWLMYDVLAMVKQTKSLTSDDIRMGGSNPLYTMLDAHRSQYLLDNRSVFEDYIRFAPSKNQGEQVARALEIIRMYVETNPGLEEVMFVINEWARASKLFENQPIRNYHLSLLFILFATRQFSSVDGNAAAFFPKIDERGWREEKANGEFKQGLEFMEMKKSQMIVKFLEFLASRKFRKLPNVSFRTLRFSSIFMRGEWQIFHVAALKTYYNLLFNLRFEELPVSTDPTMTVRSIIRENEPFVVELPADADRTLVHRKLIEHTGVEEIYMRNMEKSVRGVDEQQKANTRYLVSTRGTLESMYKLRQLVAVKVPIKTYVTGQEISTQMARLCYEKIVKGHIS

***Caenorhabditis japonica* EGO**-**1**

MTSTYQPPPPQPPPTTHTRTRTRTLPNNGYVRLTIPRSDPSVSEEEWQFYAKKQEDDLLACFKYYSFRIFREGESIRVSGELEDPLFETTYMIECDNGFDYKILEAVFIFTQETLKYDKSHRSSLALYCTDLFEKKLTFVPFLPDEIHNIQSSFSSTLLDVPTYRIFFGNIHDNEFYNHWQVSFPPNYKQKLAPNRLKKNCQYNVPKLQTHFEFDKSSPITVEYCTSWRDTLQKYRIAIDGESLHKIIVDCLYYPHEPQFSGQSRARIYLVLNSPAKVSRCFLRNHSGRKHMGFQRVLELYWGADRAEESSKKVLTENRIFCLELPTLSFATFYTIISRLRLRWNTQIVFGKLTDHVSSTCLFDAAIPKPFTNQSVYGKWVERVYDPPSMYKSTEPVYPCDIDEKLFKSFLNSVFPKPPAQTSVRNKAADRNEERKFTYVYLIEALLSRGAVVKDQLILCEEDWKRFLDIIKNHYWDDNDHYLCESALEDLLNHVDLQPNVGNLLNTFERFCEARRITQVSNQMTKEEWNKGFRKVRKAILTPTRILYMVPEIIMSNRALAGADHDGTRIIRTVFKDDSGLPMRPNMLKGLLKPIVLKHLSEGFTIMGRDFGYLGSSNSQMRENGGYFMERYNSRFKKEMLEKNFYTVQPSDKPKIIAYRKSLGKFEELESIPKAMSRLGLCFTQARVCNSFKIGLCEYQLIHDVIGGKNEYNAPYTFTDGIGIVSVALAEKIARSQQFPTRFTPSAFQIRFRGFKGMLVVDPGLDIAAAHFKTLYKQCAIHNQEKLTGDNKVDLSPWTKHCLFRKSQFKFKSATSGQTEWPIELVKWSSPAPVNLNRPFINILDQVSANQSYKCHKRVVERIETLLDDQMTSFGKWIMNEESCRSRLSDIPHRINFSSLEQKFGFNLSTEPFFRDIIKAAIDVSMSRLSGKIQIQIPQNLGRTMFGVTDETGQLQYGQVFVKYTTNMNDKFPSQMANSIILKGDVMITKFPAIVPGDVRMFEAVDVPELHHLTNVVVFPQSGPRPQPDEMAGSDLDGDEYAVIWDKELFLEKNEQAFEYCSDKPPKEFKVEEMDSQFHEFFAEYMSLDSVGQTSVNHLYQSDQYGLTSEVCFNIAKKNSMALDFSKSGVAPEPLTNRWTYNTITQTREPPERSDRQPDFSIQQCRSSFVPVYTSSRLLGCLWRELRLIKDVIVASTERQIEVVPDEFLVWNGWQDYEQIADEQMKRYNSRLRSLMDSYGIQTEAELFSGCFRSLTNRVSDREADDMSLYNTEYIIEAKMTDLYRTFREEFFAEFVPEEKGYMKVTEPECDRYNDDCEDVLRRVCRLPNERMMAKAVAYYKVCYNAVAKNSERKLSFAWIASDILGYIKSKNVLVSEVVPGISHPLFRTIYKHRQAFISENTQTFHEFMNRIQINNLESNNQERTAQQIIQNYIQMYPGLEKYMFIIDTWARAARVLQDADEIGEGLSGSELRVVLKDAAASRFKWYHLSLLTIMLATKRIDKRGQIDSNNGYAVVDVLSEPLNHNNSRMIPEDDLDRLTLSFFRHLSSRWFKSLRYISFQPIGFPSIFMRGEWLVYRRIAIRTYFNILLNLRFDDLPLLSESAIDRRDVIKEGVPFVVNLPVSVDVENVLEHLRLKSKCTEIKGRLEFTDRVLISARGTVEALEDLRNFVSVK

***Caenorhabditis remanei* EGO**-**1**

MGEEGYRGWIKLEIPCSTSERQMNSIIRCHTTKLEHALNSSNMRILTRGQVQAVEEQDCELYFESNYEVASDKFSHRLIIAIQNFLKDLKQERLMPFQLGNLVLHSSDFWSSELTCHLVDIPLNAFFFGNIQGGTFINHWEISFWEDTLRRNMNRARNRPPTDRDRAGFNQIKVEFEFDKVDFMTVHFKQKETDYISEDRQTVNIYYQITVRRTSIRRVIVDPVVSDLNGHNRIRIHFELNCPVLIRRAYRTDRQEAEFKHVIPHYRRHLVINRGRKADQYPTAKAITDSPVFTIEFDNSVDVSFGNAFCCELAHFLGDLVRVRLKSYSRSIENLIMFIRKVLYSKIAKSERNVIIMLCIFQLPVVYRLISRLRVRTGVSIEFADISSVDCLIWRENPYHRWTFQNNQHMSPTHFSSPIYRDFIATAFPRKHEVCGSREIDTNRERKFAITYLLECLISRGAVVKDQILLEDVVWHRFLEVILHYYTKDDKLCEAGLEDLVHMIDGRKRIGSLIKCFDRICQTRQKNSLVNGLTTEEMREGYQRVRKIVFTPTRVIYVAPETLMGNRVLRRYDHDGTRVLRITFRDDDNQKMRTNKTSMLLDKTVHTYLRDGITVAGRDFGYLGSSNSQMRDNGAYFMEKYSSSQCREYQSRTGVKPPIDFNPKIQAARKNLGRFETIDNIPKMMARLGQCFTQSRLSGVNLERCTYMTTYDLTGGKNVKRDEYTFSDGVGMMSHHFAQMVSHVMDFGKGVPSCFQFRFRGMKGVIAVEPLLDNIRQWFLENGIPEASEETSWNLNCVFRPSQIKFISKRHPRDQVEIVKYSSPVPVALNKPFINILDQVSEMQSLECHRRVTNRIEELLDRQMLSFAQQMVDETFCRNRLKELPRRIDIDYLRTSWGFTLSSEPFFRSLIKASIKFSITRQLRKEQIPIPSELGRSMLGVVDETGRLQYGQIFVQYTKNLALKLPPKNAAREVLTGTVLLTKNPCIVAGDVRVFEAVDIPELHHMCDVVVFPQHGPRPHPDEMAGSDLDGDEYSIIWDQQLLLDKNEIPFDFTSEKQKTEFKEEDIDALMREFYVKYLKLDSVGTISNSHLHNSDQYGLNSRVCMDLAKKNCQAVDFTKSGQPPDELEKKWRRDEETGEMIPPERAERVPDYHMGNDHAPMYVSPRLCGKLFREFKAIDDVLKISEERDEQVEITIDETMMIDGYKEYMHSAREDLSRYNAQLRSMMENYGIKTEGEVFSGCIVDMRNRISDKDQDDMSFYNTNQMIETKVTNLFKKYREHFFEEFEGGWQANTEVTSRHGSETNILQRQCRAPSNRMMRKAVAWYRACYEEARITRENKKLSFAWLMFDVLGMVKQIRSLTSETIMLGSSNPLYTMMDSHRKQYLIDNSAKFSEFREFTAHPEDGHQVERALEIVWMYAEMNPGLDEVLFLLNEWARASKLFDNHPIRSYHFSLLFILFATRQFSSVDGNAAAFFNKIDERQWRMGEEHQMEQREPFSEKEKSNMTVKFLEFLASRKFRKMANLSFRSLDFSSIFMRGEWQIFHVAALKTYYNVLFNLRFEELPVTTDPSMTVRSIIRENEPFVIELPADANRELVHRKLVQHTGVEEIFMRTMEKSIRAHDDQQKTNIRYLVSTRGTLEAMYKLRQLVAVKVPIKTYVTGQEISIQMARLCYEKIIKGHINT

***Meloidogyne hapla* EGO**-**1**

MEENVKKFKIIFWPIHEYQHIPPDFDVKTREIVESALSDSYLLNTLQFRIVIDIEPQRLGEEELDARLEYSLKLTEKATNDHLMHMINFFNLFLRHKIELAQYTFTCAFVLTTREFFKVDFNSANLEIPTQNCSFLNMIDRSKPFLHRKVAVNQDPRRIMNRYYNKADIVWPMLTDFEHDKRQLVIRFPFGDRQRREEGSEDVDYVVSLTIRYRQIKRVLADFRHEKGRGQFSLELYFHLSSPPFIRRVKLYAQDPKKPASNNNLLHKQGDRYISWDLRDPYLAGEVNESPIFRIMLCDLANEEYCQLLNRLAMSAERFVEFRTFTPDAFFPFRKFIQSPLENENCRQICENNYKLMYMIAALLSRGAIVKDYLMVTEATRDEFVQRCAKDFKRDKALSLEVIEQVLAEIDKRLDCIHPLQIYKWIYHRLSRNVDAMKQIHEDMMREGYVRVRKVVITPTRMLFVAPELLMGNRVLRLDAEKYPLDKFLRVVFRDDDGQSVHAVNIGPVLIDRFIGLRLRNGVELAGRKFNYFGSSNSQMRDNGCYFINATFEEIEDIRKQLGSFKIQSAPKMMSRIAQCFTQARETGLELERRHYATIHDYLGGRDTNSEPYNFSDGVGRISYETAKELSRELKLDGCVPSCFQIRFRGYKGVLSVDKSMDELREWGARNGVQDTTNWEKRDCWLDLHIQFRPSQKKFKAPRANQKLEIVKYSSPVSLCLNRPVNNILDQVSAQQSKASHQRICNRIHHLMDLHLHMLTRSLIDETKARNRLGEFPKVILYDQITDLNLTKEPFFRSMISASVRASLKKLRLKLQIPIPTTLGRAMFGIVDESGQLQYGQVFIRYTKNAALKLPMPTAERQVLKGPVMITKNPSIVAGDIRMFKAVDIPALHHLCDVVVFPRYGPRPHTDEMAGSDLDGDEYTVIWDEQLFLDKNEDAFDYTCKAQEAAAISEKDLRDKMAEFFVDYIKQDSIGRIANAFLVNSDLYGIKSEICMRIAAKHMEAVDFPKTGVPPQSLTKNWETEKLPGENGEPGQPAMPPERAERSPDFMEKNNEPIRAKEIDDILTIATQDEEMANIELDPLITYPGCDDPAMLDLAQQHYESYSANIQNILDCYGIKTEGELFSGHFASLRNRLSDKDSDDMSFYNTTNAIEQQLFSIFSRFRRQFFETSFGSTPAYRYEDVTVPVHSYSRTNGVKKNYEDSTTHHLLSFPWLAWDVINALRRVNAVNSLKPDTRYSFDPLSDKISVHIEEYLLNRKKQFKEFIRVMQAPESMPTEEQEPYRILHLYCSNYEGLDKLLFFCVKWARMWQLLGDGQSGITQLHICILLIQFGLNHLPCEAIESRPFWLEQIQSAEIDSQRVISLERQHGGLGNKFFLFMEFLASRQFHLLPFLDFSEPGMHYQSILMHTQLEYIHKAGLQTYHQVAFTFRLECIPGTAAQRERNGITSPQERECEPFIIEVPASRQEQVINNYDLLNKLCDATGCTNIVFRDLPKYKDYETVRLYVSATGQLQALNKLRELLAVRAPLNLVGDCRQIFTQMAEQLYDRLINL

***Meloidogyne incognita* EGO**-**1**

KWTNFLKGTVKKRWKKMKNVSKFKGLPKIPKIFPSRLLKPDSKHNPCVVAKTNCLNGGLCMSVGHEWYCECPKSHYGRFCEFVADQTECERNLCQNNSTCYSTEDYRQVSNPNVSQVKACADRKMKDSTNNETCLFSLNVKYMCWCKYGNEGAFCEFTEGMRCCAEDHCNYHGLIDYTDVFVKKKGKFDLKERKYKFKPVCEEVVCKCVCEEFYSAEDNCSTPDPCRDLECVNRGICKTKMNGTDKIGVCDCPLDYEFIAPKQGIEIWGERCEFINVTDDDIKEDQKACIPCEEGNGFDSYVNCINKHWNKLDISKYFPKECFDNGKRKVCTEALKGKFCLNGGKCEAIVYNITLFTGEDEPNNKPFFLVPKCECPSGYDGDFCENRMLDECDRRDIEEHKNYLAEIGNPNKILLNFSQEAFIQTLKNRLGWKPKYFETRRDAQLQGLEKALRTRGMKTYCGPHGFCRPYIEEDGMSCKCDFGFKGPRCELVNPCEPDPCKEDETCIEFPIADDSVEESELTYPMTFRDIICFFSKSNLVLMTDLKLVFFHNNSKKLEKASHVLVESVLGDSRFNSRKGTILGFVGSPLCYDDSGEEPRVEYLLGIGGRWKHISIIFILEHLLHLLKETTIKMGKPFTKLQYTVVIPSASFSNVHMLPFKRHFSAVDEYYFEQISFGNILHHNQLYVQRAINILEDVSTTLKINKKFLPSIAISRLTQTQMIKQTKSTSFDSGTGSSLVNGESNEQKQSCSYHISAEDSDHDDYRVCASTPPPESLTLQEESHIIGEFEHDLRVLNLRFFVTYDSRKDQKDRWRVIDQIRLVIKYESIKQVHLELEKRTPNTFWARLMLRLKHPVQIWRIGDVFALKTGKGIDRRRQKGIRVNKWGGDNKITEDVANSSVIVLDFMEESPERLSALLGRLMGLLKLDLELRNIEQRFIYAMPQVSLPSQFDTDFEFYYWIEALGSRGSVVIDHWVNESEQKKFFKTLIDCYNQDRQRTLTALEMLSDRLLDRSEVRHGRGLTGLFQDIERDLAKNKIEQEELKNGFVRLKKVIITPSRMILCGNEVLMGNRVVRIDPVNYPTHKFLRIVFRDEDGSRVHQTCIGGSLIDNFIRERLRGGMSICEKPFHFLGTSNSQMREGGCYFLQVAGFRKRSKLFKPRLQLRDLHSLLKPMQTLKAELEEVGEFRKRLGLFDQMPTIPKMMSRLGLCFTQGRDTNVDMSLQMLGFDFIGGPNSNGEHYTISDGCGCMSMAAARKVAEEMELDPMNPPSCIQTRESYMAVDFYFRPSQKKFNIQEEEERLCRFEVVKYSAPVPLFLNKPLINILDQVSKLHSVESYERICKRLHMLLDKHIFSLCSTLTDELRARKRLSELPRYVEYDMLDAINFTQEPFFRLVLRAAGRVSLFKLRQKLAIAIPYQLGRVMFGIVDETDSLQYGQVFVQYSNEMSAFNHGKANKKHFGKKIIVTAIVGGDVRMFEAIDVPALHHLVDVLVFPRFGPRPHPDEMAGSDLDGDEYGVIWDPELAFNKNEPPEAGDSFEHDDFQDKMAGFFVNYLKHDSIGRIANAHLACSDLYGIKAKVCGNIALKHQRAVDFPKTGKHSAPLEYNWKGSDPPERFERAPDFMEKESSEPQYHSSRLNGQLHRRVSELSDFLSSVIVQESTNLPDPDEMLLSNDGHDAYLDKAQTDYNLFSYRIKRLMDEYGIQCEGELFSSSYSVLRNRISDRETDDMSFFNTTYVIGQRLWEIGRLTRKQFFASAGGKIEKLTDARGICVDPSEKLRLLSRAYYTIAYYANKRQYLSFPWVACWDVLDKVKRTTILKRGVTNLAIDPFADRLLSHIKLFVKKYKTKLEEFRERVFQIDFEGPEEEYERKCFLRRYAERYLGLDILLFFCSEWGQKQKLFELSPIREEHLCLLVVSIELSRGSFYETPKDWSDSSSSPSADISSSLGRRFFMFLEAMASLQSNRKEVFDFRHFGLEYQHFFNNGEWRVLQQVALNTYLHLVFSDQFDIVLPSTVEKENLSVKEEILEGSCFQLELPSTTVNDNFVEYYKLLEVLCKESGCERIMMRCHPRYRIWRRRLVPNNIEFPRAMRILVTSWGKKQAIGQLRQLLAIKPQMDLSTDHRLAVRIIPEVTLELFNELLDREKVNWAMEYQFNGINQIEEDEEISENSQEEEELVEYYDDSE

***Oesophagostomum dentatum* EGO**-**1**

VYGEYPPXDAASQSVQKSLTGTVLLTKNPCVVAGDVRIFEAVDIPELHDLCDVVVFPMHGPRSHPDEMAGSDLDGDEYSVIWDQELLLDRNEAPFDYTADKPETKPINRETMNSDMVDFYIKYITQDSVGTISNSFLFQADLYGLNSEVCLRLAKKISQAVDFTKTGLPPQPLVKDWTEDEETGKEIPPEKSERQPDFHFGNDYEPTYRSARLMGSIYREIKAIEDBLRISEDLDEQDPVECDSYLMVDGWTRYREVAEAQPSKYKGRMRAIMENYGIKSEGEIFSGCICEMRNRISDKDQDDMSFYNTNEVIEKKVTSLFREYREEFFQEFGGWQGCTRLVAKKFAIEENIFHRYVQHPSLEMQQKAVAYYRVCYETAQQTLERILSFAWLAYDVLALVKQERIINEENHIPAATPLYEMLKNRINNFCEKNLASFYSFADNLSGEAKQPIVMYMNHYTGLRRMMFIVCEWAARNRLLLGRLQSHHVCLILILYATGQLQGSLNRQKPFLEEIEGSDVNCENPEDIDDDSQMEIIVAFFEYLASRAFRKLPHISFDTLGYACVFLRGEWIPMHETAVKTYYNMVFNLKFDELDDNVFLDPSRSLAIRESEPFVIELPERADILEVTDRIKEKTGVDEVSLRRLPCRQEGRVAVSARGTVQSLRMLKDLVTVKPLIKTAARGKEISDQLCRLVYENIMRH

**RRF**-**3**

***Ancylostoma caninum* RRF**-**3**

DNHLFFDRVRXCMKECSRACEEALENLLCAIDERRVMDLLRAFHKMYMIRVSAMKRYQHGEYRDIKRTLPRNXVLVRKVMVTPLRTLFMAPEVMMTNRVVRRFGEEXALRCVFRDDSGARLIVKDFVQGPCYDQQSSIVANIVQRTLSHGVEINNRHYHFLXXSNSQ

***Ascaris suum* RRF**-**3**

MGRTMYGVIDDTGVLQYGQVFVQYSPSVRTPSKKVITHVGPVLVTKNPCLVGGDVRMFTAVYQSSLSHLRDVIVFPRYGPRPHTDEMAGSDLDGDEYIVIFDKDLFLDHNEEAMHFPKPVASDYDTPPTVRLIFHTIHFIQQPYLFLLRSYQRR

***Brugia malayi* RRF**-**3**

MFPCLSIERIRFLLWEANGNVEETVEMAFELLNDRPSMSGVAPLEQQPINEDYRMCNCSIRFFDELIPCIQAAYEEKCVEVHVGIELPCDKSSNQDALIEFTKSFTVESGYGLEMQPYMEIQNIDRILTDIRPDHTNVDILWIGIGNMPNMGLFFIRGEYVTKYNASSNKLIVNSVCETKINNAAGNHTLLSWAHFEHDRRLLTIYFAIEKVTQDHDGLSYIGYKFVITYNSFHMVIVDCDSDPDERDNYIYIYLRHPPQLWEALPRIMTNGKRVLNIEQCRDWIRVGSFPGSKHFAGCSQETLAGSSWFSFSISKEAVKPERMFPDEVLEWQRRPLNVKLAPTRQLFDIIARWKCRAKCRIFFAAVMKIPRRDISDIPILELPSFRYQEMLFCTFCKRKCNQNSKELLLQECLLACEVTLDSALAAVDERRRISLLNFFEYNYFRKLNALRRISDDDEAASLSDLPQNCVLIRKVIATPLRLLLLPPEVMMTNRVIRHFGEEYALRCVFRDDNGQRLVPKEFTRGRSLQDQSLIIPDLVYRILGTGLRIASRHYQFLAWSNSQMRDGGCYMYSDAIVNDEVHGEVVCNVEDIRRWMGDFTASKTVPKLMSRMGQCFTQAQPTIKLKKDDWKVEDDIMGGIVHPETGEVFNFSDGVGRISKKYADRIAKTLGLYPTPSCYQVRFKGFKGVLCIDPLLDELDQVNIVFRKSQKKFEEDDESAAEVEVVKYSMPSPVRLNRPLIMILDQVSDKQGRHLHKKVCNRIHSLLELELDKLAAMMFDENVATEELSSRLSLPIDFHQLHSNGITFTNEPFFRSLLTAIHRYNIKLHLSKSKIFLPCSMGRTMYGVIDDTGLLQYGQVFVQYSSSMQYVSAKKIVYTGSVMVTKNPCHVAGDVRMFEAVYQDALLHLCDVIVFPRYGPRPHSDEMAGSDLDGDEYTVIFDKELFFENNERAMFFPNSLPTEYGTPATTGDMIDFFLKYLSQDSIGRMSNAHLIIEVCDDIARKCAVAVDFPKTGVAAKPLLSHEQCEVMPDYMQNMIKPSYRSKRLLGELYRKARRVEDISEIVQGTKFADYCDPQLCDESLFHEQSELIQESLRLRDEYHSRIQQLMDEYGISDEASVISGHSMTIKRIIEMEKDDYSFYNSDKIVELRYSRIFSYFRHEFFKEFGGESDFQTVDAFGKREMRWNVALIRKAKAWYTVTYTSAFRSSSEFRSFPWIVWDILLIIKRQIALTLKSPALSTNPLAVHLTRAIETFCYYHHASLKQFINEMSLGPMRLEAFVRYSQRYGSNVEMLCFVIDNWLRVEGIYERSALRRIHTVVLFLQFAVGILHGKHTSNELMNRHPIYFEKLDDLRDNPGTVDLACSTGEILFAFLRYLASRQFACANFIRFRLGGMMKGKKGASVFTRSEQWSALHAVAFRAFHHIAITANFDALRIMEHGDDVARHEWDFGESDTPIIVSGQLCSHKDLSLQRINAALKKWSGVYEIMSRATRYEQLLITCTGSILARQRLHRILLMEPERLFDAVLTDTMPKEARDDFL

***Caenorhabditis brenneri* RRF**-**3**

MNQFDNGESSEEAITVVRPKHPRGVPQSQASFPRGRSNFSSGTLPNRKSESTPVNTLATGHSNKQLLTTFKMDRTAAVPRIDLEPVNRGYLVDFNLQPQRTSRNHDFMSSTKRHSLPSTHILFEKTKHRGGPNVEEQQRMGRLIRARAEEAEMNNNTRIEKEAVKLSFDVKIVGSMTHETFGYTRTHMESMKDFFSTRLKDSDVDEVTWSKTGMAPRAAYEEKAFVIEAHLVLTPNREVQDESKLFADFIGKFTGKISGMLHDQVFVEVPKMQNLFTKILPTHSSINISGIAIGNCPNPGLFLVRGDFISKENTESNVKIPQHVHENTACKVAGSNDYLSFARFEHDKRVAHLYFGVSLPEFSEDGLNHAGFRLSLYYNSITKIVVDFSNEKMNTFYIQLKNPPHLWEGVPKINMSGPGKSKVLNLETCTDWIRVLSWPGDADRRGIGCSTEAYSQSTWIRISFMKGDDMSSTKPSQLVDILSRIASRSNNTKIHFGSVFSIRRKLAPSPALLSLGTFRANYALQALITRGSVFMDQLFDATDPNIPEEKSREDGDDEEEEPAKTPMDPKDEPLFLKLVRRGMKECPQAVEETLEQLLNAFDERRSIDVLFAFSTMYKARKTQYERLLSGESLQDVGLAKPLPKNCVQVAKVIVTPSRILLMAPEVMMVNRIVRRFGPDYALRCVFRDDNLGRLAIRDFSVNNIDHLSNIVTESIFNTLKYGVQVADRKYSFLGWSNSQMRDQGCYLYAPEGGLSGKYCFSDGCGRISVKLANHITRILELKETPACFQVRFKGFKGILVIDPTIDDIIGMPKVIFRKSQQKFGEGGGDQQDEYLEVVKYAMPSPVCLNRPFITILDQVSAKQSASSHRRITNRIHYYLERELCSLSNMLLNENQAAEELVVDETGLLQYGQVFIQYSQSIRQTSATPILKTGKVLITKNPCHVPGDVRVFEAVWQPALAHLVDVVVFPRHGPRPHPDEMAGSDLDGDEYSIIWDQEMLLDYNEEAMIYPSSTAPEEDKEPTTDDMVEFFLRYLQQDSIGRMSNAHLTYADLHGLFHENCRNIALKCAVAVDFPKSGVPAEPLASFEQCEMTPDYMATGGKPMYYSPRLNGQLHRKARKVEEVLEEYETRGSIFEGEYDKIICPDDADVFFGSEAKLVQAMMIRDEYIDRMQQLLDEYGIEDEASVVSGHAASIKRLAGMERDDYSFYHTDKIVELRYEKLYHVFRAKFFEEFGGEAAHLVNDGSKMRLNCTKAMREKVRQWYFVSYVQPKKNKTGKFIGQSLPWVVWDALCDLRRELMVSKTNAIPRVKYPIGVRLEEEIHKSIEQRAERFERFKEQAQGLRDAHYLRRYTKFYGDGILRTLFIIQEWLLKEGILPSDGLTIWQIGRLLIRSGLGDLKGNPTIDYEKNILNPTKIFQQWIPKKPEVMEEEVMVTTQFEMGTLILEFLRYLASQSFALAETIPLRVFREHNIVEPILYDSNQWIPFHLVAYRTFHSISVSGRFDALHLEDSDADELISESKDPILVNESSFSSQNYTDSPINRPQVLNMLKEWSGVKEMHTREVTGTRKSDLQYVTSVGTVLARQRLARLLLLSGETIRDAIANKTIPAEVRDEFL

***Caenorhabditis briggsae* RRF**-**3**

NGESSEEAITVIRPRHPRGVPQSQSQFPRGRSNFSSGTLPYRRSESTPVNTLATGHSNKQLLTTYKIDRASGSRQRMDSDYLDPSTSSAFPSNPPSARLNNLSLPVNRGYLQDYHLYPQRPSHYNNQRYNNNHNDGNTKRHSLPSTHILYEKTKHRGGPNVEEQEKVIRMLRAAEESEALAKPAVVKKAPIELSLDVKITGSMNNEMFGYCRTHMETMKEFFSGKLKESNIDEVNWMKTGMAPRAAYEDKAYVIEAHLILTPSVAAFDKNDLFAQCITKFTRKTKGMLQDQCFLEVPKMHQFFPKITPQHVDINVSAIGLGNCINPGLFLIRGDYISNDNTVCSVKLQSQHNPSDASREFNSYKVAGSSKYLSFARFEHDKRVALLYFGVRLSEFADDGLDHAGFRLNLYYNTFVRIVVDLSAVNTNSIYIQLKNPPQLWEGIPKSTMFHPSKSKVLNLETCTEWIRVLSWPGDAERRGIGCTPEAFYQSNWIRIMFRKETDDEKLCSANLIDVVTRLASRSNAKVSFGHIFSIRRQLAPSPCLASLGSFRANYALQALITRGSVFMDQLFDCTDTNIPDPLEPEDEEGEGELLKQPMNLKDEPMFLKIVRRAMRECPQAAEESLEQLLNAFDERRSIDVIFAFSTMYKTRKVQYERLLNGESLQDVGLARPLPKNCVSVAKVIVSPSRVLLMAPEVMMVNRVVRRFGPDYALRCVFRDDNLGRLAIRDFSINNIDHMSNVVTEAIYNTLKNGIQVADRHYSFLGWSNSQMRDQGCYLYAPRVDPISGKVSGTVEDIRVWMGDFREAVSVPKMMSRMGQCFTQAQPTVKLARHHWLVEPDMEGGPGNKFCFSDGCGRISYKLAGQITRKLKLKEIPACFQVRFKGFKGILVIDPTIDDIINMPKVIFRKSQQKFGEGGGDQQDESLEVVKYAMPSPVCLNRPFVTILDQVSKKQSAKCHQRITNRVHYYLERELSSLSNMLLNENQAAEELVNRTNLPIDWNLASKRAGFQLSVDPLIRSMLFAIYRYNIIHHISKAKIFLPPSLGRSMYGVVDETGLLQYGQVFIQYSPTIRQTSPEPLLKTGKVLITKNPCHVPGDVRVFDAVWQPALAHLVDVVVFPRHGPRPHPDEMAGSDLDGDEYSIIWDQEMLLDYNETAMVFPSSQAQEEDKEPTTDDMVEFFLRYMQQDSIGRMSNAHLVYADLHGLFSENCASIAQKCAVAVDFPKSGVPAEPLTGFEQCDVTPDYMMSGGKPMYYSASLNGQLHRKARKVEEVLEEYESKGSIFEGEYDKMICPDDADVFFGSEPKLVQTMNIRDEYIDRMQQLLDEYGIEDEASVVSGHAASIKRLAGMERDDYSFYHTDKVVELRYEKLYSVFRAKFFEEFGGEEAHTVNDGKGSRVVCIPAMHEKVRQWYFAAYVQPKKNKTGKYIGQSLPWVAWDVLCDLRRTLMLQIDDAIPRVKYPIAARLEEEMEKSIESHKEKFEALKKQLATKKEAHFMRRYQHFYGPQIIKMLFIIQQWLEREKVLPSKSLTIWQVGRLLVRFGLGALGGNPTIDYEKSILAPAVVFKKWITKKAGGDEEPIMSRFEMGSMMIEFLRYMASQSFALANQISLRVFKEMEIVEPSLLYSYQWTPMHNVAYRTFHSISVSGRFDALRLDDDGAEEQVSESKDPILVNESLFSNRNYSDTTPITRPRIFGAL

KDWSGVKEIISREITGTRKTDLVYVTSVGTVLARQRLARLLLLSGEAIRDAIVNNVVPPEVRDDFI

***Caenorhabditis japonica* RRF**-**3**

MLYVFGLCFVFVWTGTDSNARNNVISPPYLCAIVLLALCAVLCVPVIGLTVFHLVLVARGRTTNEQVTGKFTSGYNPFTIGCWGNCKRTLCHSQIPPFKSYVIAFRKQRRAEQKMLAARQHAPLEPKNAETDEENEYVPDEREAVGEHIPLVKVIRNQRSASGAVSASDSQQKIAESQSMSMSSCDESSRILAGVDVAAKADGSTCNLFDLERGGTPRLTASSSIRGSRHNETVSPSRAHQSYEEALEEALRSNPTSEGAQSPAETPTNSGATTSRTIAPSSEFIGVPQSQSSFPRGRSNFSSGTFPNRKTESIPVNTLATGHSSKMLLTTFRMDRCSRLRPHTTETSEYDTVTDQMTDGASTSRQDFHPNHVNNFSCPINRGYLQDYHLFPTRNGSKQEGSKRYSLPSTHILFGKTKHRGGPNIEEQEKMVAMLQAAAKITEIDSVKEEVIREPVQLNVYVKICGTMNNSTFGYCRAHMGSMKEYLEKCLKHSGVSQVEWLTTGMMPRAAYEEQVYQIEAILVLNETEDAEYTSEIFSNFITKFSRKMHGMLHDQIFLEVMKLQTLFPKILPQHVDIGISGLAIGNCPNTGLFLVRGDYITQDNTVCSVKLQSQYNSDIARENTAYKVAGGNKYLSYARFEHDKRVIVLYFGVRLCEFGEDGLDHAGFRLNLYYNQFIRIVADFSKETTNSIYIQMKNPPHLWEGIPKNGMYHPSKSKILNMETCTDWMRVLTWPADAEQRGIGCTMEAFSQSSWIRIIFKKDDDSDSVNSPFLMDVIARLAARSNAKVSFCSIYSIRRKLAPSPAFYSLGSFRANYILQALITRGSVFMDQLFDVADPNIPTEDGTPLDIEAEPLFLKLVRRCMKECQQAAEESMEQLLNAIDEKRCVNVLKAFSILYKTRKTQYERLLSGESLQDVGLAKPLPKNCVSVAKVIVTPSRVLLMAPEVMMINRVVRRFGSDYALRCVFRDDNLGRLAIRDFAVNNIDHNSNTVTESIYRTLRHGIRVADRLYRFLGWSNSQMRDQGCYLYAHRVDPLTGEVSGTVEDIRKWMGDFRDAISVPKMMSRMGQCFTQAQPTVRLERKHWIVEADIEGGPNNKYCFSDGCGRISYRLATQISKILQLKEVPACLQVRFKGFKGIVVIDPTIDEIINMPKIIFRKSQQKFGSSTDDSHDDYLEVVKYAMPSPVCLNRPFITILDQVSAKQSSISHSRITNRIHYYLERELSLISNMLINENHACEELVNRTNLPIDWIYASRQAGFQLSVDPFVRQMLFAIYRYNIIHHISKAKIFLPPAMGRSMYGVVDETGLLQHGQVFIQYTPNIRQSSSNPTLLTGKVLITKNPCHVPGDVRVFQAVYQPSLAHLIDVVVFPQHGPRPHPDEMAGSDLDGDEYSIIWDQEMLLDYNEPAMVFPSSTAVDDEKEPTNDDMVEFFLRYLQQDSIGRMSHAHLAFADLYGLFHEHCHSIALKCSVAVDFPKSGVPAEPLAAYEQCEVTPDYMMSGVKPMYYSPRLNGQLYRKARKVEEVLEDHNSRGSTFEGEYDKLICPDDVDVFFGDEAKLFQTLSLRDEYASRIQQLLDEYGIEDEASVVSGHAASIKRLAGMERDDYSFYHTDKVVELRYEKLYQVFRGKFFEEFGGEAGFVIQDGKASRVVGNAAMLKKIRQWYYVAYVQPRKNKTGRLIGQSLPWVCWDVLCDLRKKLMVEKNDAIPRVRYPLSARLELEMRNSIEEQHDMYLEFKKLVEEEEEALCIKRYAQVYSDQLIEMLFLIDIWLKHENILPSDSITIWHVGRILIRFALGVLQGNPTIDYEKSILKTNVVFKRWLSMEADEDESPIFLEFDVGSLMIEFLRYLASQSFALSEHVTLRVFNDTGITEPFLLRSFQWLPLHHVSYRTFHSLAVSGIFDAFHFSDGNYLELVSESRDPILVSESLFSSKNYSDDNPINRTRILQSLKDWSGVKEIIPREITGNRKATDLVYVTSVGTVLARQRLSRLLLLSGETIRDAIANDIIPAEVRDECL

***Caenorhabditis remanei* RRF**-**3**

MAETANPIDNGEPSEEIITIVRPKHPRGVPQSQSQFPRGRSNFSSGTLPNRRLESTPVNTLATGHSNKMLLTTFKMDRTASRPRMDSEFPDNPGPSTSTAVPPPAPSHINNFSCPVNRGYLHDYQLHPQRPSNPRNIDYTSLKRHSLPSTHILYEKTKHRGGPNVEEQEKVVRMLRAVAEESEAIAKPKMTFRKQPVELSFDAKIIGSMNNDAFGYCRVHMETMKEFFSAKLKESNVGEVNWIKTGMMPRAAYEEKAYVIEGHIILTPNDEVEDKAELFSEFVTKFSSKITGMLQDQCFLEVPKMQKLFTKITPQHVDINVSGMAIGNCVNPGMFLVRGDFISQENTVCSVKLQTQHNQDVSRENSSFKVAGTNKYISFARFEHDKRVAMLYFGVRLSEFADDGLDHAGFRLNLYYNNFVRIVVDMSQENMNSVYIQLKNPPHLWEGIPKSTIFHPSKSKVLNLETCTEWVRVLTWPGDAEQRGIGCTPDAFSQSTWIRITFRKDDGIDSVPSEHLISVITRLAARSNARVTFGSIFSVRRKLAPSPALASLGSFRANYALQALITRGSVFMDQLFDCADKNIHVPDDDQEEKRPMELEHEPLFLKLVRRGMRECPQAVEETLEQLLNAFDERRSLDVLFAFSTMYKARKVQYERLLSGESLQDVGLAKPLPKNCVSVAKVIVSPSRVLLMAPEVMMVNRIVRRFGPDYALRCVFRDDNLGRLAIRDFSVNNIDHMSNVVTEAIYNTLKSGIKIADRVYSFLGWSNSQMRDQGCYLYAPRVDPITGAVTGTVEDIRIWMGDFRDAVSVPKMMSRMGQCFTQAQPTVRVSPILFQLNFLFQVKLQRHHWIVEPDMEGGPDNKFCFSDGCGRISYKLAGHISKILDLKAIPACFQVRFKGFKGILVIDPTIDDIMNMPKVIFRKSQQKFGEGGGDLQDEYLEVVKYAMPSPVCLNRPFITILDQVSAKQSTASHRRITKRVHYYLERELCSLSNMLLNENQAAEELVNRTNLPIDWNLASKRAGFQLSTDPLVRSMLFAIYRYNIIHHISKAKIFLPPNLGRSMYGVVDETGLLQYGQVFIQYSPTIRQTSDTPILKTGKVLITKNPCHVPGDVRVFEAVWQPALAHLVDVVVFPRHGPRPHPDEMAGSDLDGDEYSIIWDQDMLLDYNETAMVFPSSSSQEEDKEPTTDDMVEFFLRYLQQDSIGRMSNAHLVYADLNGLFHENCHSIALKCAVAVDFPKSGVPAEPLTSHEQCDVTPDYMISGGKPMYYSARLNGQLHRKARKVEEVLEEYESRGSIFEGEYDKLICPENVDVFFGSESKLVQVMTLRDEYIDRMQQLLDEYGIEDEASVVSGHAASIKRLAGMERDDYSFYHTDKVVELRYEKLYSVFRAKFFEEFGGEEANTVHDGKDTRVACTPAMHEKIRQWYFVAYVQPKKNKTGRYIGQSLPWVAWDVLCSLRRQLMLEKNDAIPRVKYPIAARLEEEMEKTIARNQEKFDEFEKIMETRKDTLFIRRYQHFYGKQIVRLLFILQGWLERENVLPSISLTVWQIGRLLIRFGLGALRGNPTIDFEKSILAPTMVFPEWISKNPGDDEVPILNQFDMGSMMIEFLRYLASQSFALADSISLRVFKDRTITEPSLLKSYQWTPLHHVAYRTFHSVAVSGRFDALHLDEDEIVEHVSESKDPILVNETLFSSKNYKE

NCPITRSRILQALKDWSGVQEIIPREITGSRKTDLVYVTTVGTVLARQRLARLLLLSGETIRDAIANDVIPPEVRDEFL

***Haemonchus contortus* RRF**-**3**

LQDQTALLQVRFKGFKGVLAVDPSLDSSQKCPKIVFRVEDDVDGGSGHPETREPYCFSDGCGRVASSLARRIALALQLDVVPSCYQVQMRDHGCYMYASTISGHTGDVSMTVEEMREWMGDFSSSKNVPKLMSRMGQCFTQAQVISSDLRDI

***Pristionchus pacificus* RRF**-**3**

MLSAGIGRWKRGNQKRREREWNEGSGDSPESRIKIHGGMQNRVEEEFGKRQRVDACTRRVLCSRECQAACEEALELVLTAIDERRNITIERAFDRLYTTRVELYRRSASSHRSVQAVRTIPPNCVLVRKLMVTPSRLLPMSPDVMMTNRVVRQFGAENALRIVYRDENGQKLKVNDFGQDQMAPLLIDMVKNTMDKKVTICGRDYQFLAWSNSQMRDHGCYMYSQVEKEDGTIITIDKIRDWMGNFSSSKNVPKLMSRMGQCFTQAQPTVSLNMGEWRIDPDIVGGSGHAETKELYTFSDGVGRMSMSLARELAEMLELKIVPSCYQVRFRGFKGVLTIDRTLDAPGEPRVIFRNSQNKFIGSAEDDSSILEVVKYAMPSPMCLNRPLITILDFVSENQGKDYHENMCSRIHYYLEKELNTLSQMLLSDKEAATALTSRVPLSMDFHRLLSAGFTFTNEPFFHEMLVAIYRYSVSQHLAKAKLEMPSEMGRSMFGVLDETGLLQYGQVFIQYSPSVRNASDKPIVHLGPILVSKNPCHAPGDARVFEAVWQPALSHLSDVIVFPRYGERPHADEMAGSDLDGDEYSVIFDPKMLLGYNEEAMVFPKQTAANYNFTPTTSDIADFFLKYLQSDSVGLASNAHLHMADKRGLFTSVCESLARKCSIAVDFPKTGEPADALEQNERLDALPDFAGNRKKQSYRSTRLNGRLYRHPPGKKLQPDAR

***Trichinella spiralis* RRF**-**3**

MSDVGDCFEEFHASVYIAKEVSGNGGLFDKVKNFVSSVCEPLSLHFSIRPCLTQENPDLFTEKFHTSISSDWEDVEQYHESVSEIIKRWCSTIRGNLHEQPLLELIQDATFPFQLIPLHRYNLQGKVSFGFMLNFGIFTEHCVVDLSGSWTAKAIFEHDRRCFSITIFRKFSKNCSMKLLILYDSVSAIQVDFNDGKADVYFTLCHPPFLFGPDGGGEQKNQQRRLVRLNKFFSCDMENFGKCKVIKMTKSVTKCIAFKKFFWKFLLRMKKRCWNAKIFFGAVQTISVDNLVREDFAKREIGDWPFWCQYYWLALGSRHHSIVYQLTNHFFDEVSRCLTENLRSTEVAFIQLLCIIDNNSQLIVNVRQRFRQVYDKQCSLTDNDYSIELDLAAPYVSVRKVVITPTRVLLSVPDKMMSNRILRQFGSDLALRVIFRDEGGTRIPASKFSLTSLKQIVTTVMMKGLAIGPRLYQFLGWSNSQLRDHGCYFYASTEQVNVEEIRRWMGDFSSIRSVPKLMSRMGQCFTQAFAITAIPFNPDSKEVLIETDIQSSDSKFCFSDGIGRMSTKLAEEVAERLELWPVPSAFQIRYAGFKGMLCVDPRLDGDVKMVFRQSMHKFHTEGAHTLEIVKHSQPCEVNLNRPLIMILDQVAFKEGVPVNGRVQNRIMQLLDQQICELSQMLLFEDQAKSVLSATMHSEMNFEKLADAGFRFTEEPFFRNMLLACYKFKVKEKLFRMKIEIDPSLGRTMYGVMDELGLLHPGQVFIQCSANILRPGEDKRVITGKVMVTKSPSVVPGDVRILTAVDLEIYHYLNDVIVFPKIGYRPVTDQMAGSDLDGDEYAVIWDEELMFCNNYPAMLYEKVISSEIDGDITSDKMVDFFLHYICSDSIGRIAIAHLVASDSYGIFDDVSNCIARKHSMAVDFPKSGVTPPKLENDEIPLKYPDYIPKCYNSLYRSKWLLGSLYRKVYMLNSIAELSLARKVTTGKSDPHLKIAEAKNFEEKARAIFQRYKSELHSLLDEYGIENEFQLITGKITKMHSRLSDGERDDFSYYNTEKVLKVRLGMLQKKYKKLFVSQFSSVVSKEQLANYTEVQAAASAWYSVAYRDAEISNRTALSFPWLVWEVLLDLKLKNCPSERIDPVVVNLQNFIQIQRMNNEQLMEIIDRILENSKVNRENRQHFENVQRILYTLFNWCDSVGMFVKSGGSAEVVNVVGQLFVDFVAFFEKSIENEMALLELNDTNIRSNGAISDHYCARVLLKFLEHCVNRRFDFSPHGQQFSKSSEANILYRSAFKQYHGMAVSGNFRGLFPGDGINEETDILLSDVREMEPMMLNLSRETLACLKFGAIVDMLKKWSECEDVAFRSVNIESDRDDRFMISAYGTLQARRRLQNILLSAHIEQWLESGECNCDVDEIAWL

**RRF**-**1**

***Ascaris suum* RRF**-**1**

MRATDQALPLCTINPMKVSGMQGHKVHTRVCERISALLDRQLMQLAESLMTEDRCRDRLKEFPRRISIDELSLARGFALTQEPFFQSLLRASVAYTVSK

***Caenorhabditis brenneri* RRF**-**1**

MADPPRHGFIKLEFPESASTRREMDEVVERLTSRLEPSLDAYGIHIVKRHPTQIVEEQDCDCYFEVNYEVTSRRFNERLIDAMKEYLMEINHTARYQLPNIVLHSPEFWETEIESLVRSIPLSAIYFGNVQGNTYLSHWEISFWDKIAPKSKRSKKDLPMYQINVDFEFDKTDMITVNFQCQEEKKQNNNGNNSTNNGNNNNNRKWMTVNYQVTVRRESIRRIIVDPCATDCYGERVRIHLELNSPPLIRKGRIDEQSQANPFHQPYYKRWKTFENLEWKNHGYPMPEAISDSPFFTLDFDETETNDQIYRVLSRLRDRTRVSIEFGLLPIVNIPLGRKFPYNRWTIQGGVMKTATDENAAIYREFLQELFPPKYQIIDGKSTDINEERKFAITYLIECLLSRGAVVKDQLLLNEIHWENFLKIIILYYKKNDKLCEAALEDLIHLIDGRKRIGCIMKCLDKICQKRERMQLINGLSEKEIHDGFQIVRKVVFTPTRVIYVAPEAIMGNRVLRRFDKDGTKVLRITFRDDNNGKMRTNTTEELLEKTANKFFREGVRVANRDFGFLGCSNSQMRDSGAYFMLKATPSQLERFYRKNPRATREQLLNYKPLIDEVRLNLGKFSEVENVPKMIARMGQCFTQSRLTGVALERKNYCKIFDYEGGKNSKQATYTFSDGVGMMSCRFAKIMAQSMNYDRSVPSCFQIRFRGNKGVIAMEPQLDDLRNWAIRHALEEIIPEINVAFRPSQIKFQAKHIEGDQIEMVKPAGPVPVSLNKPLINILDQVSEMQGVVCHKRMVSRIEELMDRQVQSFAKQMNDETYCRNRLKEFPRRVDIDYLRTTWGFTLSREPFFRSLIKAAIKFSITKQLRKEQIQIPKELGRTLFGVVDETGRLQYGQVFVQYSSNILNKHPVRLADQSVQNGVVVTGKILISKNPCIVPGDVRIFEAVDIPELHHMCDVVVFPQAGPRPHPDEMAGSDLDGDEYSVIWDPQMLLEWNEPAFDFSVEKQRIEWDPNEVDNLMREFYVQYLKLDSVGQISNAHLHNSDQYGLMSTVCMNLAKKNSQAVDFTKSGIPPADMTRKWEKDPITDEMVPPENPERIPDFHMGNERNPQYVSPRLCGRLFREFQAIDNVIKISEEKDELYDIEIDEDIMIYGYERFMVEARTELANYNGQLRSIMETYGIATEGEIMSGCIIEMRNRISDKDQDDMSFFNTNLMIETRVTTLICRFREKFFEPFGGYQSCCSIIENADENNSTCLAYRCRSATNSMMQKAVAWYRACYEFAQSNRETRKLSFAWIVYDVIAKVREAAMLSQENLQIGGANPMYTFLEKHRKQYLEDNLDDFELFQSLNQFRNDERAKRPLEILKMYFDSVPGLDSVLFMLYKWAHLSKLFVDQPIRPYQFFLIFILFATKQLPSPDVRLFEIVDEEVYREEMEKNGRVKPQIEEELREEKKSHMMVKFLEYLASRNFRKLPSLSFKSLGYSSIFIRGEWQIYHVAAMKTYYNIMFNLRFEELPVSTDPTISKTTVIREVEPFVIELPDKIDIMALHSSMQENSKVTELEMRRQAPKKEPSGREREEERVETRVRYVVSARGTLEALQRLRQLTAVTIPIRSHLEGDDVSRQMAFLCHHKIMENYDENNLM

***Caenorhabditis briggsae* RRF**-**1**

MNPEELIDEDAFAGGKMNVIRSPQEADMMKRRSHLFLLRPKKDPETLSLRKMTESSSPELPRKLRHGFIKLEFPESTNSRKEMEIVIGKLLAGLPESLQRYGIQIEREHPMQVVQEEDCDACFEVNYEVTCEEFKHNIINAMQRYMDGWRHCVAYAKPTILLHSLDFWRTTLHLQVNDVPLSAIYFGNIQGNAFINHYEVSFWKENEGKSIRRKTPMNNITVDFEFDKCDMICVNFQCVQIEEDRNANAGGDKRNQFNKYDNKNQKKMKKMTVNYQVTYFRNYEFSEISEFGITVFSKFQKFLGFLNFCHFQVRSISIRRIIVDTNVTDRYGDRTRVHFELNCPPLIRKGIINADHADNPYVKPFYQRWKVIKKEWDGHGWPNEAAISDSPIFTVDFDPIVQVRNFWNFELHRILSRLRVRTGVSIEFGMLPAISVPMCKRWPYHRWTIKDGKQLAAADTDAPIFRDFLADLFPKKFEVNDDDKLIDVNDERKFSITYLIECLISRGAVVKDQILLDEQHWRSFLGIIWRYYKVDDKLCESALEDLIHLVDGRKRIGSIIKCFDKICQKRQRMQLINCLSESEIRSGFQRVRKVIFTPTRVIYISPETIMGNRVLRKFDKDGTRVIRITFRDDNNNQMRANDTGDLLEMTANKFLGEGIRIANREFGFLGCSNSQMRDNGAYFMAKYSQSQLEKFLRTRPNEAQLKAFKPRIMEVRKHLGKFDKLENIPKLMARMGQCFTQSRLTGVELNRSDYIRIPDFEGGKNLAGKPYTFSDGVGIMSYRFAQKVAHAMQMGNSVPSSFQVSSFFFFSDLTHIFLQIRFRGMKGMIAIDPYMDLIHQWNVSYDIPYQVPNLELKCAFRPSQIKFEAKSLPGDQIEMVKYSAPVMVALNKPFINILDQVSEMQSLECHKRVTGRVEELMDRQILSFARQMNDETYCRNRLKEFPRRIDIDFLRPMWGFTLSSEPFFRSLIKASIKFSITKQLRKEQIQIPKELGRSMLGVVDETGQLQYGQIFVQYTQNAAKKLPMRGPNMTVPDAKIVTGTVLVTKNPCIVTGDVRVFEAVDIPELHHMCDVVVFPQHGPRPHPDEMAGSDLDGDEYSIIWDQQLLLDRNEDPFDFSVEKKDPVPFDFDQIDDLMRDFYVKYLKLDSVGTISNNHLHNSDQYGLTSTVCMNLAKKNSQAVDFTKSGDPPAPLTREWGKDPETGENIPPEGAERIPDYHMANERVPVYVSPRLCGRLFREFQAIDNVIKISEERDEQFEVQIDETLMIQGYEAYMEGARKDLANYNGQLRAIMETYGIRTEGEIMSDCILDMRNRISDRDQDDKMSFYNTNQMIETRTTALFCRFRENFFKEFHGFQAACTEIQNSNDSTNPLNFRCDGPSVPMLQKAVAWYRACYEFAQSSRETRKLSFAWIVFDVLAKVKEHNMLHGENVEMGGGNPMYIFLEAHRAQYIADNFEEFQEFRTMSCLIEDDRSVKGVNILRRYMARYDGLDTVLFVLMKWGEAMHLFHNQPIRKYQFFLMFILFATRQIGLADLLSETFIARIVEDDVENASTVPLTEDKKSHMVCRKEKKRMSKFLEFMACRKFRKLRSISFRALGIPGVYMRGEWHIFHVASIKTYYNILFNLRFEELPISTDPTITLESMIRESEPYTIELPERTEANECLKMLTTKSGCQEVHMRMQSNRAEDKKRADEENGKEKCDRGKELVEKRIKKISIHREVRTLRYMVSARGNVESLQRLKQLTAVTIPIRSHIGGKEVASQMANLCLDKIMEESDRG

***Caenorhabditis remanei* RRF**-**1**

MSVSQLRHGYLKFEFPESMKTQKDMEEIIETIIEQFEPSLEDSNIRILKRHPTQIVEEQDCDCFFEVNFEVVSEQFDFKLIDAMNEFMKKLTPVAAYKRPTIVAHSSDFWISDLRLSAKDIPLNAIHFGNIQGNRFLNHYEVSFWNETKRFKNGPAGDPLYKIFANFEFDKNDILMVQFQCTQMEEDRTTSGSDSSDKENKNKCNKNENKNQKKKIERTVNYQVTVRRDSIRRIIVDPYAKDDYGERVRIHFDVNCPPLIRKAILDKKMYNQNPHYVPFYKRWKTIKHVWEDHGYAEEAAISDSPVFSLDFKHPQFAEIYSVLARLRARTKVSIEFASLPSVNVPICRREMYHRWTISGYGIIRSSTDPTVPIFRDFCAEVSIYHFPKPNTINIFQIFPVKYQIVDNREIDINEERKFAITYLIECLISRGAVVKDQILLDEGQWSNFLEIIIHYYKIDDKLCEAALEDLIHMVDGRKRIGSILKCFDRICQKRQQMNLVNGLTENEIRDGFQRVRKIVFTPTRVIYIAPETIMGNRVLRRFDKDGTRVIRVTFRDDSNGRLRASTTGEELIDKTAMKFFSEGVKVANRDYGFLGCSNSQMRDNGAYFMEKSTFGQRQRLLKNNPNANLIGFQPKILEVRRHLGRFETIENVPKMMARLGQCFTQSRLTGVELERSDYCTTFDYEGAKTKPIVGKCYTYSDGVGIMSYGFAQRLARSMNFGISVPSCLQIRFRGMKGVIAIDPFYDEVSEWAQANGIQSYDVPNLDLKCQFRPSQIKFPAKSIPGDQIEMVKFSSPVLVALNKPFINILDQVSEMQSIECHRRVTGRIEELMDRQIVSFAKQMNDELYCRNKLKEFPRRIDIDFLKRMWGFTLCNEPFFRSLIKASIKFSITKQLRKEQIQIPKELGRSMLGIVDETGRLQYGQIFVQYTRNSNEKLPPRSNMQHMKVQGSQVVTGTVLLTKNPCIVTGDVRIFEAVDIPELHHLCDVVVFPQHGPRPHPDEMAGSDLDGDEYSVIWDQQLLLDKNEAPFDFTVEKKEMPYDREMIDQLMHEFYVKYLKLDSVGTISNNHLHNSDQYGLNSRVCMDLAKKNCQAVDFTKSGEPPAPLTKKWARDPETGEAVPPELAERVPDYHVGNDNAPVYVTPRLCGRLFREFQAIDDVIRISEEREEQYEIEIDESIKMEGYENYLTCAEKELANYNGQLRSIMETYGIQSEGEIMSGCILEMRNRISDKDQDDMSFFNTNQMIETRMTSLVCEFRKRFFEEFGGYLAVCTQLPNTFDANNSLTYRCEMPSELMMQKATAWYRACYKFAQKSRETRKLSFGWIAYDVLAKVKEHHLLKSEEIHINSSNPMLKFLEQHRKQYLIDNHEEFEAFRSMDQFVQDENSGKAIRIVTMYTTRLPGLDTVLFMLMKWGESLKLFEDQPFKRHHFFLLFILFLTRQLKSADGFTKESFFEKIDVNSEESDDSEEEDEQNVLTEERKSHLMIKFLEYLASRNFRKLPNLSFRSLDFSAIFMRGEWQPFHAAAIKTYYNILFTLRFEELPISTDPEITFQSMIRECDPYVIELPGKKIQDPTIRTKLHFSDLPDRLEDITAMMATNSGCDTVSMRRQSNKGKKEDKDDMTDGRNRYLVTARGTLENLQRLKKLVSVTIPLKSHLGGADVAKQMAYLCFRQIMVGFE

**SMG**-**5**

***Caenorhabditis brenneri* SMG**-**5**

MISKSSKNIVANWKSMVNPKVYILHRWRCYEESERKLIDDLIFCLRARKQLIALMKHSSDCTSNINKLWIVGYYHPFQYYIRENDNAMETTTLLTMFCGELQEMLMLSNQRYYSLWNLYIGDLHRYMPDDELQKTLAVGFYSRAIELDRRQGRAFHVLATLGRGLSLAEKLRLMILGQIAEIPHKKSSELVDFLKLNGADENIGGSEKFLIEFVNWAWKDAKSVDHQLLGLKLGNDFKTIVDNDTINDLPMIMSVCRCAGVFVFKNYGYTQFQNCFDVVSNFYLTIFSKASINRSLLPEAISWITDAGSIFGSLDSTKNEFHYQSLSIFMKTKWNELNDSIMECINKQFKSEPFEIAPSPNEDIGFLINGPKSSPSKELLGSLVNKLIEISHPTVQLNQANSITSGPLLSRINQTVQKRLDIPLESLSLKENTSDREDWRPVYVMMDFDTLVDNIRIARKVWDIDDFICVLPSSVLDQLDKEKTKNRAVRPIIRSLMELQAEGKMVLRKCNNDRHCAEQLVKSAKRSSNDHKNIVAFLCKNSEDQEAMEGVTFYDIHQFYKKYLE

***Caenorhabditis briggsae* SMG**-**5**

MTMTAADNFKRYCSQLEKYGQTESVHSPVMALLRRKARKQLIELMKTDNDCTSSINKLWIVGYYHPFQFYAREDNSDMDTITLLTMFCGELQEMLMLTEQIYASIWNLYIADLHRYMSDDEIQKSLAVGYYTRTIELDPSCGRAFHMLALVQPNLLVAQKVRLLMLAQLAENPVKKRLDVSEFVKSSESDSNSSDQLLEDFANWALNENQKRVDHQLNGLKMINQFESDFKESPISNWSMTMSGCRYVAQLANKNLGFNQFIDCFDVITSFYLEIYSYPSPTMSLCAEIISWICDAKEIFDVNDPVKKEPYFQSLSVFAKTKWNELNDLAIEQINTVFGSGSLSEGVNSIRYLIFGPESEPSLEELSLLVKNYIRLNQSTVELRSEKGDDSRLLKRINRSETKRLDIPLEVLAEKVSQMNREDWRPVYVIMDYDTILNKSRNARKVWSIDDFICILPSSVLEQLDSQKMKIKSVRPVIRTLMELQAEGRIILKACENERRCAEELVQSVKGTANDHKHIVAFLCKCPEEENPIEGVTFYDIDKFYTKYLNS

***Caenorhabditis japonica* SMG**-**5**

MAKSEVKLKFDRYCAQLEKYGTTETIHSPNMALLRKKARKLLIELMKEEADHSHELNKLWSTGYYRPYQFFAQTSDSSVETSTFLTIFCGELHELLALSEKYAAQLNLFIGDLHRYMPDDEIQKTLASGFYARSIELDPRQGRAFFVFSTTRDDLKFAEKLRLLILAQLAEKSYDTDGKLMDLIRDNINCEEENPDKFLAEFINWALFQKVSKSESHKIGSKLSETFKLMIEKKSEADWPMIMSACRLASSLAMENAGFSRFMDCFDAISEIHLTLYSKNLTSIRSLFEAVAWISTVGNTFESLDMLKNEPYRMSISSFAKTIWSNLNDAVCNHLNNVFSSSSSSSSVSTFSDSESIAFLLHGPKLESLSILSSLVHQILSLKNPTMKKSDNNGETAFCRINQSSPRRLDIPIEAIIEKVDLSNREDWKPVYVIMDLNTIIKNINVAIKIWKMDDFICILPSTVLRQLDDLKTQNRSIRSVIRTLMELQLEGRIIMKKCEDERKCAERLVESVRKNSKDHEEIVGFICEHPDSEEQLEGVTFYKIDDFWKRCLD

***Caenorhabditis remanei* SMG**-**5**

MSEDAEKFKRYCQQLEKYGQTESVHSPVMALLRRKARKQLLSLMKQDIDCSSSINKLWIVGYYHPFQFFIRVRYKLQNTFKLTVYFQEDDNSMETVTLLTMFCGELQEMLFFTTKNEYSALWNLFIADLHRYMPDGEIQKLLAPGFYSRAIELDPRHGRAFHMLSVTLTGADYATKLKLMVLSQLAEIPHKKSSDLNDFLGKANGDKFLAEFCNWALNENPKRVDHQLAGLKLINQFKTEVESENDWPMILGVCRLVAKLAYKKFGYNQFLDCFDVISSFYLEYYSKSETTKSLLSEVILWICDVGEVFGSENPVKKEPYFLSLSVFAKAKWNELNDLVMDHINSLFASEHLLEDSSTPIPMINSSEPSIQILSQLVHNLLRIGHPTMELLKQKGQPLLRRINQTESKRMDIPIEALAEKISDLSNREDWRPVYVLMDTDTILNKTRFAHKIWDIDDFICILPSNVLDELDNQKMRNKAVRPVIRSLMELQAEGRIILKKCTDERHCAAQLVQSAKGSSEDHKNIVAFLCKKPEEEEPMEGVTFYEIKQFYTKYLE

**RSD**-**2**

***Caenorhabditis briggsae* RSD**-**2**

MCLLRVEKKLGKLKSNIDVTEFIEKMYEFEKKSKMGESEVTAIVLSFDEASHTAMCFSTSLEAMINIKLPLELPITTGKLIRFTDYDYSNENAVYTPVSKPDSISVTEDDGVRLDGNRLLIEAMIAFSVESNHPTYSKKKAYAGVFGHVEVDENVALRMERNQVYKAELTFNNSTELSQKSTYGFKVWSICGDASPEESEAFMTSLYLYEDKIRVEPVEPENTNLLPMNEFSQLPSGHESVGNSYRPPNKVNEDSERLQAGADNRSIASSESSRSSQRNLGASERSERLQFGRQTSRNNFGAQNPTSSGQNMIPEDKEIENCAVVLDSSFREIFVYETTLLKLMKLENPNNERCAKWGVVRFIPEGPQNGTTFVKANSFRYSPPGAAVILTPNGPPQMKTHLVFSNNNGYKTYNRSCGFSNRFGLVEIADPADGLLGNTVYSTNIEIKQWLDPLEPVFRSVSKLKEEDVEFSDDFANSMVQFEENLKKQIQSNGLSASKMPQMDRLPSRPQKSSRNKPDRTNLPDWINGLVVSVRKVGNRRWGRLLTPVGDAVFSEMPTHGSTGKHVQVLSWISVRITNVSGELWVTKIEKDAHEFLDTSVAMTGDEIEVTTVLTSTSNFSTVDNINYFKHKPLGNVQTENNLDFGQNGIAHVVCRRGFGRPIPSKKGEECYWFARQIFPQSRSPERRSRASEYPSNDSGWDNRQGNRQQPQDPFSNPEKNIFADHESQDPYGNPEKNVFADHGSQDPYCNPEKNMYAGHGSQDPYRNPEKNTFADHVSQDPYYNPEKNKFADEGSHYGLSSFTPTNGNQYDLRQNGFNNPNSYCEHSGPQSYQDHSRPMSQCQPQFPQPSPEQYRPQQPFGEDTMSFSGRNDGTPLNCLIVSRRKNIMLAYVFKYRTSADIVEKRDVGLSILVNASECYCNLPIDLSQPLTMYQDYSNRTGNFVLASGEVGSILLDNSTRDVTGNKGAAAITDRYRGKPGSSDMVAQCQYTECLVPPAMLNRAGDNEDVFFAWKVIQVIGTNQSFEFNEAESKKRNLGPDAHELEEDDDISRQKRQAKMAYEASLRREQEMHSSASSNIGPFQNQPSGSQYANSERYGTPNGSRMFGSEQGFCTAPQSINAGPSRSETPVAAAFNADPYQPSAVVNLAQSIKNRIASYTRNPDLAAHMEAVVPGNLTSLEYEIENLLRMCQSSNRS

***Caenorhabditis japonica* RSD**-**2**

MSQSQGSKKELELSALVLDPCPKMGSKEIICYETNQLRLLRVNWTDQADDDIHRYDTLQFITDGDDGVQCIVKPGTTPRITSPPANGLVLRPDRSVKIEAYVAFSSDRDHLSHNKSVAVSDQYGYIQLPPDCDKDRQYWEEQTIYKVEIVISQFTDPVQPIFKVVSPLVTKFQRTQAADEMFFSTIRKFENQYAKNFRENHLGSTHAPRGTSLKERKAETRNIKPGARRLTRDEVKQRVDAVVLHLQPQYGGVCGVVSTPLGEADLTTMTITGAEREEDSEAGQAVRVGDWIKVKLEYREKQRHLFASKIMSRERIPLVLRMVNDEFGQIQVLHGPFNKNSLRYDHHEQGIRYYKHPRLGHVEVDATFYNDEASVFDVEYRRVRRPTEQIVGNASIWVYWYISKRLEDVEQPSQQSGPRGQQQVHGCAASSSSSDISPVIREADNMSTRGSSYASVPPASRPDSMMGSIGEGFRQQSGPSGPSGPSRMSSSSYSTITNNEGQKYNGPMYDDTAMVLKRFPTFFVVYLRERNMAAWLPRQICRSSVTLGKCITVEYIPFEHHPGKDFIHWEVTRVVNVIQGRSEVREDGDDALPLFHVDLYDIGTEWNDYREFGGQPIVKSDDCIYATVADRVKTAQGETISANELIRRYQSKKQAKRLCGFMAYKVDVVPQRMLDQEYHGEDEVEAYTFQLIELVGSHEHYTRKQNNLDSTNLGYENRNLQEDDEIARAKLAIREEQERRMNRSSPTGSFATGQSNYDERSRYASGRTSSASYYTSAPNQDPFNTGAPREFNDRGSTSSYQSASAYPQAHPQPQPQHQYRGKVDVDSFVRITPLIMNLKTVAHPMLYWGFLDGRAKPFRKGKWKEPEWNLCDRYLPYQLGGGYVLSYELVRFLATNARMLKMYKNEDVSVGAWLAEAAAAGFLGHGHDFRVLLVVFYFFL

***Caenorhabditis remanei* RSD**-**2**

MTEDTQVRAIVLSVDETTNTIVCFSEPLESKINVKLPPNFIAKQGQRLTFGDHDYNNETYFPGSNPEWITIEDDPSVRFENNELLITTRAAFSFLDTHPTYSKKKVYAGGFGHVEIEDHLSEKMDRAIVYKVELSLNVLETKDNEKPVFKVTKVHELLESEDVEAFLNDLYEFDERTPAETGDEVVDNNQREIELPHVHMNENTESSARSESLTSYNRETLLTVAERSMPVEEPLEQETTKNVLIDVNNDASNENVTSTSTEETGLSSSSSMSLLNCQSIDLSAQKSDGSGETSPEIVPFQEDRFEMVQDGIREDSIVQSDSSSMDYPATEASSLGKDVRDSKPKPFSFGRMPTSFSERAQTTNSVGVNEVQKNDIEYCVIVLDNKNGAVIGFETNLLKLMTIVMEETDLKRYGTLRFISEREGNGKVYPKLGTVRFTEPVDSVIVNGATSIPARVMFSSNQKHGSYNKTVAFSDSYGLVRIPESNASLKHMAVYNTFIQLLVLFKLNIQSDKVRFRCQNEDKLKPIFISVSPLKQDENDPNARFIESMYNYEDNIFTETRSRGIASTRPPQNSWKSVGIQKGQKGNFKPRRVTLPNFYKALYVSDKQHGKLFGVLYTSVGDASLRVRPSIIDTGVMLNLSEWASVKLEAIGPEGREDFFVSKCSSFVDPLGCTTKFVNGEIVVEGTLTLKPVAKYYDHLYYKHGLLGLVQGDTDLYLGYENEDVHVQFTRLEPENYRKLTIHEYKAVNKKFDKTLDEWERVCWRARRLDRSSLDSSNRSREEDLSDCNKGKDFCNNSSSIPQDPLKTPEKMKYVESRNDCQTCEHPNHTPQRQNFYDNSYEESSDNGIPYQQSYQNFSQSSSQYYSPRPTDFNHLESERQYYNHSFVPSEMDTASVTTDSNNLISSIGMIVSRHKQMMVVYLDDFRRAGILRWKHFDSSFIKLGNLFRCVCKPIEIQEETLHASYEVIKIIELVNERNETIVKDEECQVLVNVNLFNQDPVFNDFTRKSGRFVLSSEDFGNVLMEDTVVNVTGQVVPSAGVIEEYRGLPGSDTMVGYCVYKKCLIPPMLIDSSCRETAEVVAYAWRLVKLVSTLTHYSFNQQDIKIKNLGEKASTLEKEDDISRQKKQAQIQFELDNQNRQGSSSVGFHQRPQSSRYEQSSSNYENFATPQGSVYNHRMESSQSYRTANEYRLGSQRASSPSLRSEITQYNVNMSPTFRSTPQPMQHYDLQADNEKILRMLEVLNERVSVFTRNPDTREVMHSLAPGHLAMLEEAMESSEKQLQEWRRSTHH

***SPREADING PROTEINS***

**SID**-**1**

***Caenorhabditis brenneri* SID**-**1**

MKGSGSILGCLLIVLLGQLGLSDNITDKEPSELDFTKNNETGPIIVRQLHSKTKMFNATLKPGKVHLYYLSLDDSYVLDLMRIAAEVIDPVLYKNEKDAILEVTVSNGRDNFNLKLPVIYPNLTLYSYGKLLNPLISDDFGPKISKKRRNSTGNQTLVISVQSRLKVDLDYRLHLTRLDRSQYNLKFKPGQSTKTLSNQKLTFVKPIGFFLDAIEQNVKSFHITLLSDDDFCASLITVPANESIYDRPVDADKADNNRVITFTKRADIFFSQTEIETFKSFRIFVFISPVDSPCSTNSSRKTFNENKKVTFEFAKLEPSSYFFPTALMMAFLATPVLLFVPALILNRIRNTSTSQSTLISFAPDTPDQSYQVEEGPSTDNDIVVPEEENLQNQEGNIIPLPEDSLSLHGQMLKYPLAIILPVFMHTAVEYHEWTTSTMANRDEMCFHNNACARPYGELRAWNNIISNIGYAIYGLVFIAITMCRRWRHGSPVVGIYECTLLDVTIGVFMILQAIASATYHICPSDVAFQFDTPCIQVICGLLIIRQWRVRQESPSASYTNLLLFGVVSLNFLISAFSKAKYVRYLIAMIHISAIASMCLAKRRTLETKKTFQVFTACFAAINFLIMLTYLAPSILHLNQIVTYCFISNCIMYLVYYAVMKLVSRERIGLKGKVCGGLAVIGWIVAAVFFFQDDTDWTRSSAASRALNKPCLLLNFFGSHDLWHIFGALAGLFTFLFVSFVDDDLVNTPKSAINVY

***Caenorhabditis briggsae* SID**-**1**

MIGSRYFVSGVLLLLLGRFGLSEQDNNETSPISVREFPARRRQINDTLVANTVHVYYLKLDETFILDLTRISADVSEPALFRKEVDTILEVTVSNGRDNFILKLPMIYPDLTLYSYGKLLNPLRKEDFGPKRSKKNKNSTLSQNLVITVQSRLRVDIDYQLHLTRLDRSQYNLSFKEGQSTKTLNNQKLTFVKPIGFFLDTEEQIVKSFHITLTSADDICANVITAPANESIYERPVDSDRADNRRVLTFTRRADIFFSETEIQLFKTFRIFVFISPVNAPCSGNTSRKNYNEIKKITFDFTRLEPNSYFVPTLAMLVFFASPCLIFIASLTVNVIRNRSDLVADLISFSSDQSANTSAIAENNMAHNEIAVIPEEENLQVQEIEPIPIKQDSLSLHGQMFKYPVALILPVLMHTGVEFHNFTTSTMANRDEMCFHNNACAKPLGELRSWNNMISNIGYAIYGLVFIMVTMCRRWRHHSPLVGTYECTLLDITIGLFMILQAIASATYHICPSDIAFQFDTPCIQVICGLLIIRQWLVRQESPSPAYTNILLFCVVSLNFLISACSKASGIRYLIAIIHFGVVATVCLAKRKTLRSKQVYKVFVGTFAIFNFFAITIYVTSSYIHLNQISTYCFILNCILYLTYYALMKFASRESIELKAKVCGVSAIFGWLIAGFFFFQDDTDWTRTAAMSRALNTPCLLLDFFGSHDLWHMFGAIAGLFTFLFVSFVDDDLINTPKSSINMY

***Caenorhabditis japonica* SID**-**1**

LADPNVTQYASNNEPESIIIKHLNKKLTNETLKAHTVNVLYNSLYTQSILDLTRVSVTIDEPWKFDTVHGTILEATVSNGRDNFLLKLPVVYKNVTLYSDGKVLNPLLPEDFGDRKLRKNKQRYNDENYHQNLIITVHSTLHEDIKYSVSVTHLDRSQYYVKFKPGETRINQNDQHLTVVKPLGYFLDAKEQNVNAFHITVTSDDDICANLITVPADEPLYDRRVDLDKVDNRRILSFTRRADVLFPNSEIANFEKFRIFVLISPDDSLCGASSRKTALQNKKLSLEFVKLNDASYSVPLIAMFTFLAIPALIFLPFVTLTRFRSMPIATDHLLDLSTDLSSSSQPSPLIVLDESATDNSLGIAEVNADITVLEDVKLTDSLSLHGEMLRYPVAIILPVLMHTAVEYHNWSKSSMANRDEMCFYNHACANPYGEFRAWNNIISNIGYAIYGLAFVAITVGRRRRSRQLVGYGVHESTLIDVTIGVFMVLQAIASATYHICPSNVSFQFGG

***Caenorhabditis remanei* SID**-**1**

MVGSRFFASCLLLVLLGKLNCSGNNTDNGVQETDEENPVITREFPSKKKVYETTLKPNAVHVYYLKMNETYILDLIRVAAEITHPTLFEKEEDTILDVTVSNGRDNFVIKLPVVYPEGSLYTYGKLLNPLIPDDFGPKRSKKNTENSTGTQNLIVTVQSRLRVDIDYKLFLTHLDRSQYDLKFKPGQSEKTLQNQKLTFVKPMGFFLDAKEQDVKSFHITVTSEDDICANVITVPANESIYDRPVDSDKADNQRVITFNRRADIFFSEKEIELFKSFRIFVFVSPVDSPCSLKTSRKTFNEQKKMTFEFKKLEPDSYFMPTLAMIAFFISPCLIVASILAVNVWRNRDPSNTSADLVSFESDEPNHPNANTSDEQLVVMEEEEVNLQNHENLQNHVEAVSVKQDSLSLHGQVLRYPIAIILPVLMHTAVQFHNFTFSTMANRDEMCFHNSACSKPLGELRAWNNMISNIGYAIYGFVFILITMFRRWRHHSALVGTYECTLLDITIGVFMILQAIASATYHICPSDIAFQFDTPCIQVICGLLIIRQWLVRKESPSPAYTNMLLLAVVSLNFLISTLSKSSRVRYVIATIHFIVVATICLKKQKKMGSRKDAAKFMIFFAVANFILMMMYLTSSKIHLNQITTYCFILNCIVYLTYYATMKAVSRESIGLKAKICGTLAIVGWITAGYFFFQDDTDWTRTAAASRALNKPCLLLDFFGSHDLWHIFGALAALFTFFSVSFVDDDLVNTPKSSINIF

***Haemonchus contortus* SID**-**1**

ACRQCRYGIVYGQCNQCHQRSQCGCDMPEIMVIVKQNDTWAIKSTTLSPVCLVGEKIESRHPKNGGTRRAYTRCYHNYACSEPFWIFMSFNHMFSNVGYMLCSIVFLAFVHMRKGKGTYKHHGLEVCMGLSLLCEAFASTVYHICPNSTTYRDSSVSKRWSIILSLGVVTLNILVVA

***Oesophogostamum dentatum* SID**-**1**

AQFALILLPVLSLVVSSPTFGVEWKNDQCFHNYACAEPLRIFTSFNHVFSNVGYAVSGVFFLIFARMRKNKPTLGYGVHANYGIELSMGLSLFCEALASSIYHMCPNSVTYNLDTPFIEVTCVLLMLKLYGSRRRVITPQFANIAVTSVITLDSIITMFAEKFAVRGFAVAVMIAAVLFAVSSLLFGPRFSFGFLKSNNDRNIAVSLAVGTFATNIITIVVCNLTSYRIETTQFVTVCCITNALLYLLYYTIMKYRKREKWCKFSKRSVVAATLLWFIALLCFFKEETDWTLTIAQSRAVNRPCILLNFFDYHDLWHMASALASLLLLVGVSSLDDDLCAIPTRELSVF

**RSD**-**6**

***Caenorhabditis brenneri* RSD**-**6**

METKDDLRKNLCAALLNKDPGLTEKEIEKEYDEFKGPGQLRLRLQQFNVTLRQLLTSDKEKFKDLGGGRWTGLPYIENLLAIKATLDNKKPEVRYAEERERARRERMAYYNGSQPGPSEGSRNERGPPIREMYHSMNNTFDSENPHQRQAEEEKRRESGRYFDIGVWTGSSKEPGIGREEPKRVMMRHSLQNTLDTETPPNRYGRYGEERGCTKRGSWNFNNENSAGPSEVSRSERERSGRDMVHQSMSNAPYPVKPQQNYGKGDGMWKIGYWNGGRAGTIDQSRNERHQPEREMMHHRTLHAEQPQKRYEDENGIWKVGNWNDENGDQTGMIEESKNGRQQPERETMQATNNSPSDKKPEKRDGEENEFFEIGNWMDENVGRGGTIEESRNESQQPREETMHQPMGKAAYAVKPSKRYYEEKGKTKRIRWIYEKENRTGTSSEESKSESSQPRRGEMTYDQVAPKDPRYYEHDYRNSSAEERRFTFRTENHSEVMVTRTPVPIEKSQMFTASWSHIIYSKPSTDLFSFELVQYSGLGDFTVRVTDYGSSIETITKELKQHASSPETSLIYDSWAVGDGCMVTLLQEEEEILCRGVLMKSTSENTFSVYLIDIGISVDRKIPQLKTLPPDHLDIYPLAIRCTLDADQEKLLSKAAELKEDFHTILEMEGTLMRRNTPSYSCDEELTHLRYHNFDQNIIPICKVHIWMKLGEFYYDFNCLLQ

***Caenorhabditis briggsae* RSD**-**6**

MDCDELSDEDQLAVNIYSVCTAHGFQNSFSERNLIELVDGEYDRGYTESELRKLNKTLAQVLQNSEHFRSCGQGEWKAISAGGQMDNIHDSVADNKNAKKGGARGRGGAVRGRGAAVPRNQFNRNGREGFRGFNNSRPPRGGLNQRGAAGTHGSSFRPQENHSNSQPDYLANQRPLGRNGSSNASNQSSNYDGSFNRDNSRNSGYQENVRPQSPPRYRRSSRPPSPRSRNSNYYDRDSQRYNNDSRYFDNDRREYQNDKQSNYGGDQYGGNERYGGHDNGRRSPPPMRYDDTRYNDYRQNSYERQDRYNDGHGYNNGRPSSPQYRQYSPPRNGNGYRDDSGFRMNEQQFRHDSRDEHRGRTPPLPNRLHNSPPSDHRQRRSDSRNSNGTNFSQFSEENNDSPPASKFRTEYGNTQASVQTRDPITVGPSPANTNPSGSLNSNELLNRKLCKQLLKLKKGGQMDLTDFVYFLKDKGIIIPGDNYDEQENYLCALKESMPEFFKNMTIDRDESLIIWNETEMHQNKENEDEEDVNKPHRIDDAVWNYIKSISTNSFKMSDVILHLVQETGEKAELIKSRVAHAMFYTHQGEYRYVPGEMDLVQKIERDLKGPSTDLFSAPLVHPAFSRPQTSLNVTFCFFRKFAHFAVRPVEAVEEFEKMEKEIKVSMQGANVKEQPNGGWQPRQGCLVRLHDSDIGSKWARGMIIKEENKELYRVYVLDFGYRLMVRIADMAMMPQRFLNIPPFCITCKVNATNEEHTELNTLNWKPEETNNSDRINVSNLQRGVPVEGIPTFTVDLATESFNGGMTNILDKLL

***Caenorhabditis japonica* RSD**-**6**

MSIPPDEIAAELQGKMVIDDKTGQKAYYVGELFKLYNSTEGADALQNNLAACGMSLAQLLAEFTDMFSRVGDNSRAVKTGQFQTILNSQDGGGGARRKTRGGAFGGGGGGGRGYMRGGRGSNIPRAGRPGGAGFKPGVSEFFIGFLAISPSCLVRKICANRQFGARNSRADG

***Caenorhabditis remanei* RSD**-**6**

MEKSPDEELVDTIYSACLIDGPNSLGKVYTERELKKGTEDLIGKGEIDRILRDVQLTLHQLLDRCGKFVNLGPGQWRVIADNKDAGLLESIADKKNGNRSSRGGRSGGGGGGSARGRGGASASQRGNLGGGNRGFGAVRRGNQNQRSHGSAFRGAPPAANNRPPPAYNRAPPPPQNDYSNQRPLVRPAPAAPPDYERDRDSSYRDQPYNRGPSRNSYYDEPRYDLPRTPPYQRSPSPPPGLSHGNHNSHYDRNEPRGYYVGPPSNRHPDQYDRPNNDYDNTYDNSYNDDYNNDRRGYNDNRGYNDRRRSPSPPPAYSDYRDRSPPRGNGYGPSYDASPYRDDRASDSRSNYNGSNYDGPYRAPSNRSYSPPPPPPPRSPSPVYRNAPPMEPPGLGSRHVPSQKSFRGDPSPPRRPDSRASNVSEYSQFSTDTNVPPAPSTSRQRAKERAEQSSSAPLKMEDLAAKFNAKVVVSEPAVDKSSPEYQVAVKVAEALRGGRRLFDSNKLYTYEAFNYFSAKEIQLPTDKTILEKEAFLAKIWKEYPELFKDIQLDLANDEIKFIDSPVSSAPPVPKPAASIEAPSRNSDFDIEYERSKAHVEDENHFIIMRDIRSIIANKFSITKLIDRLCHELALYRSIITEKVTYVLFVTFEGKYRYADGSQEEVVVLKNTGLRNPDYFEAPLVHDVFGERRDFWKVDVCRFMKFEKFSVRPVEAIDVYENIDKEINSVENPKIDRPIGGWKPNYGCLVLHKRDNSGTLKWGRGIIIRDQSGSSSDENRLNYRVLLLDQGHWVMVSPTQMRVMPEKFKTIPPCAIQCRMETDDEEVLMEMNLRFIGKPWRDTIRTYSAKTYITFTGNVTRFDGITTYGVQLFVEKLDGEKGNITELF

***Pristionchus pacificus* RSD**-**6**

MNEKELADSVFGGLMAKAMGKMVHESEICPLVDQIEGKGQTNEELQGLRLSLSDLLLRHPDKFINCGNRNWRLNQNNHAFQQLAESISDKKSGKGGKRSARGGSNLPSFNRARSGGIPRNHNQTHGFRQSAPAHRGHHRPPAGNHGSSFRPNLNISQPNPSNDYSKMTGLHYPVSRNVLASNNGSNSRFSNQPPRREDHHEKMIMRTPYRDPSPRRGNNYGDIRGRPDSRDGYNGDRDSTRYSSPRRNEQPGRPSSRGSNISDYSQFSSDTNAESNTERRSRPDASSPDLADLARRFNSSSINVQPQPLESVASSDVLSKKIAKAVRKIMKKLNEGEKLSLENLFDEIERKNKILIPGKNGKEKEDFLKDLKKIMPKLLSALTIDTETGDLTWNEAAVTTDSPIHEYMEDDLHYYVVDYVKMLDKARFRVTDEAERLSNATGVRMERTCEKLTYVLFITFEGEYRYASGSKTEVLKISKITTVGDAPEFYQTSFEHKIFENVPANALVKCGIVRYQNVQKFAVRPEEYIKPYEKMNQEIQDFMSQIRNEKSVPFEMWRPNHACLVQLNDSTTILKWSRAMIIKIETQLIFLFILDSGFRKVVPSSDLRLMPQKFAKLPPFAIPCTLDESEDDKDNIEFGGKEYLSNGPGNLSVTCSGAATASDGILTYPIKLFSTCHGGPLTNIKDVFI

**SID-2**

***Caenorhabditis brenneri* SID-2**

MNRQRFILITLFMLPVYFCMDGFILFSVEIQSDVGIIDCKGLDLTIDGKKGDYLCKNGSQIEDHSYVIVSYNNGTNATTYSANATVWLSAEITQNLNETETLTYNGTFTFDTTSGPNKVLIGSAILNKNQSANYTVTSKTTTLTINYLHLKYNENPTVNKSNGGAVAVAIIEGIALIAILAYMGYRTMVKHRMKETTMNAAMYGYDNNSRITVPDSIRMSDIPPPRDPTYATPPTPTAQPTITPTRNTVMTTQELVVPTSAVSPTRPNTTTTTTNQQFRDPFDSLENW

***Caenorhabditis briggsae* SID-2**

MIRNQILIIALFLIPVYWCIDVILISSIEVRNDVGSIDCTNSKLMINNQNFTPICEVGYDNTKSISYITLAYNASNSVQEGNTTYHLDTKVTVPNGNKTKTDDYQYTGVFVVDKTVQPNTVAVGYLTLEKFIPATTAAPPTTKPKKREAGFPQEQLDAEPTAPVSNKTSLTINYIRLKYEETSKQSNSNGGAVAVAIIEGIALIAILAYMGYRTMVKHRMKESSVNAAMYGFDNNSRNSIRMNDIPPPRDPTYATPPPAPFSQQPPARNTVMTTQELVVPQTSASVTRPTTTSNTTSNTTNGQFNDPFDSLDSW

***Caenorhabditis japonica* SID-2**

MKTQVKSALTVLSASLQISLCSRGRAQISGRSFAFTCHISATTSAPATTAATTAAPNASTTTIAPVVLKTNEAHLTIAYVHLQYEENSKQSKSNNNSGAVAVAIIEGIALIGILAYLGYKTMVGDKRKQQQTANLYGYDNNSRITVPDTIRMSDIPPPRDPTYAVPPTRTSQPLPPRPVPPQTMTTQELVVPSSSGAGNSTANQRQNGQFADPFASLDSW

***Caenorhabditis remanei* SID-2**

MIRYQTLVFAVFLLPVFWCFDSFLITSIEIRNDVGNINCTSSNLTVSINELALKPLCQIEEDANTKISYVTLTYNETESIPNGKNITFNLESSVTVKNYEPSQNMTNSANYQFMGIFVPDKSSKANTVLVRNVTLNKVEAPATTSASKFSEADVPISNKTILTVTYIHIQYDDSTKKEGNSNGGAVAVAIIEGIALIAILAYMGYRTMVKHRMKESTMNAALYGYDNNSRSEKMQRFISSFLSLVTVPDSIRMSDIPPPRDPTYATPPTPTVTQQTPTRNTVMTTQELVVPPTQNTSAPAPTRPTTGASGQFNDPFDSLDSW
