# Supplementary material for: RNAi Effector Diversity in Nematodes
Source: PLoS Negl Trop Dis. 2011 Jun 7;5(6):e1176. doi: 10.1371/journal.pntd.0001176 (PMC3110158; doi:10.1371/journal.pntd.0001176)
Supplement: Dataset S3 — Components of nematode RNA-induced silencing complex (RISC); domains and sequence data. (*, putative stop codon) (DOC) [file pntd.0001176.s003.doc]

**Dataset S3.**

**Domain analysis**

Argonaute (AGO) proteins are typified by an N-terminal uncharacterised domain, a PIWI domain (encompassing a MID subdomain) which facilitates binding of the RNA guide strand 5’ and harbors the RNase H-like catalytic residues necessary for target cleavage, along with a PAZ domain which facilitates binding of the guide strand 3’. TSN-1 (Tudor Staphylococcal Nuclease 1) is typified by four staphylococcal nuclease domains and a carboxy-terminal Tudor domain which facilitates protein-protein interactions. VIG-1 (Vasa Intronic Gene 1) contains a hyaluronan/RNA-binding domain. Minimally, in cases of short sequences, we required at least one matched domain in the top BLAST return, for that protein to be considered a putative ortholog. The miRNA RISC cofactors AIN-1 (ALG-1 Interacting 1) and AIN-2 contain no identifiable domains, and thus putative AIN-1 and AIN-2 orthologs were identified as such, based solely on overall sequence similarity.

**Shared domains**

AGO proteins share a PAZ (Piwi/Argonaute/Zwille) domain with the RNase III-like dicer (DCR-1), however, none of the putative AGO orthologs were identified here on the basis of PAZ domain similarity only.

***ARGONAUTE PROTEINS***

**ALG-1**

***Ancylostoma caninum* ALG-1**

VDKKDQVGKAFNIPPGTTVDVGITHPTEFDFYLCSHAGIQGTSRPSHYHVLWDDNQLSADELQQLTISDRNHYV

***Ascaris suum* ALG-1**

YLFLWLKRIVVVDDRVISLSHFFILVILSLVISLFVIDKNPNLKSSAAVDDVVGFYLHMTYVAILCMFILFCRDGVSNSEFVDTMNDELTSLKAAMNRLASDYAPTISYVVIQKRHRTRFFVECDEFARGKHNVPPGTVVDEEITSPNMFDFYLCSHLGAIGTSRPAHYTVLYDSWNLSPDDWQQVAYALCHLYARCARSVSIPAPVYYAHLACQRARFYLKEALSKSSQQTAVSGELERMVKVHENAPRMYYI

***Brugia malayi* ALG-1**

MAPYQPFLQQVPNLLGNPQFPQAGSTQYHIPQQQQQQTALQTSLQDAYQLAPINPAPVQMMLGGSGGAPSFDSRPAGSLAPGGPVGSGDGSEAPLPPPANVPLQRPTGQSSGPIQFQCPRRPNHGIEGRAIVLRANHFAVRIPGGNIQHYSIDVQPDKCPRRVNREIVNTMIRAYQKVFSNIRPVYDGKRNMYTRDPLPIGRERLELEVTLPGDSAVDRQFTVAIKWVSTVSLSALEDAMEGRVRQVPFESVQAMDVILRHLPSLKYTPVGRSFFSPPLGSAHGPSHSAQQYHTESKLGGGREVWFGFHQSVRPSQWKMMLNIDVSATAFYRSMPVIEFIAEVLEVPVQALSDRRSLSDAQRVKFTKEIRGLKIEITHCGSMRRKYRVCNVTRRAAQVQTFPLQLDSGQTIDCTVTKYFYDKYHMQLKYPHLPCLQVGQEQKHTYLPPEVCNIVPGQRCIKKLTDTQTSTMIKATARSAPEREREISNLVRKAEFNADPFAHEFGIAINPAMTEVKGRVLNAPKLLYGGRTKATALPNQGVWDMRGKQFHTGVEVKVWAIACFAQQQHVKENDLRNFTTQLQRISNDAGMPIMGQPCFCKYAVGVDQVEPMFKYLKTSFVNIQLVCVVLPGKTPVYAEVKRVGDTVLGIATQCVQAKNVIKTTPQTLSNLCLKMNVKLGGVNSILLPAVRPRIFTEPVIFLGCDITHPPAGDSRKPSIAAVVGSMDAHPSRYAATVRVQAHRQEIISDLTYMARELLIQFYRSTRFKPTRIIIYRDGVSEGQFFNVLQYELRALRECCMLLEEDYQPGITFIAVQKRHHTRLFAVDKKDQVGKAFNIPPGTTVDVGITHPTEFDFYLCSHAGIQGTSRPSHYHVLWDDNQLSADELQQLTYQMCHTYVRCTRSVSIPAPAYYAHLVAFRARYHLVDREHDSGEGSQPSGTSEDTTLSNMARAVQVHPDANNVMYFA

***Caenorhabditis brenneri* ALG-1**

MSGGPQYLQGVMSTTLPPQTPSSGSSPSSFLGTTSTTTTSQVIPTSGATQQPQTPSAQAASTALQNDLEEIFNSPPQQPHSFGESPPKTTGSLAPGAPIGTTGVTIGEPSSTITQLPGGNAPGAPGSGNQSGVQFQCPRRPNHGVEGRAILLRANHFAVRIPGGTIQHYQVDVSPDKCPRRVNREIISCLISSFSKYFTNIRPVYDGKRNMYTREPLPIGRDRMDFDVTLPGDSAVERQFSVSLKWVGQVSLSTLEDAMEGRVRQVPFEAVQAMDVILRHLPSLKYTPVGRSFFSPPITAATTALTGTTTPSQTQTSISSGSHSAGQYHAESKLGGGREVWFGFHQSVRPSQWKMMLNIDVSATAFYRSMPVIEFIAEVLELPVQALAERRALSDAQRVKFTKEIRGLKIEITHCGQMRRKYRVCNVTRRPAQTQTFPLQLETGQTIECTVGKYFFDKYRIQLKYPHLPCLQVGQEQKHTYLPPEVCNIVPGQRCIKKLTDVQTSTMIKATARSAPEREREISNLVRKAEFSADPFAHEFGITINPAMTEVKGRVLSAPKLLYGGRTRATALPNQGVWDMRGKQFHTGIDVRVWAIACFAQQQHVKENDLRMFTNQLQRISNDAGMPIIGNPCFCKYAVGVEQVEPMFKYLKQNYSGIQLVVVVLPGKTPVYAEVKRVGDTVLGIATQCVQAKNAIRTTPQTLSNLCLKMNVKLGGVNSILLPNVRPRIFNEPVIFFGCDITHPPAGDSRKPSIAAVVGSMDAHPSRYAATVRVQQHRQEIISDLTYMVRELLVQFYRNTRFKPARIVVYRDGVSEGQFFNVLQYELRAIREACMMLERGYQPGITFIAVQKRHHTRLFAVDKKDQVGKAYNIPPGTTVDVGITHPTEFDFYLCSHAGIQGTSRPSHYHVLWDDNNLTADELQQLTYQMCHTYVRCTRSVSIPAPAYYAHLVAFRARYHLVDREHDSGEGSQPSGTSEDTTLSNMARAVQVHPDANNVMYFA

***Caenorhabditis briggsae* ALG-1**

MAAELNNTNTDGPSGTAAAAKAPTVGSNDSMSGGPQYLPGVMSTTLRPTSPSNGSSSSSFLGTSPTSTSQVVPTSGASTQPLNPSAAAASSALQNDLEEIFNSPPTAQTASTFGGDSPPQRQAGSLAPGPPLGSSGVSIGEPSSTIAGAMQAGGAGAVGGNQSGVQFQCPRRPNHGIEGRSILLRANHFAVRIPGGTIQHYQVDVSPDKCPRRVNREIISCLISSFSKYFTNIRPVYDGKRNMYTREPLPIGRERMDFDVTLPGDSAVERQFSVSLKWVGQVSLSTLEDAMEGRVRQVPFEAVQAMDVILRHLPSLKYTPVGRSFFSPPVISASGVPGTAPPPQAAPSISSGSHSAGQYHAESKLGGGREVWFGFHQSVRPSQWKMMLNIDVSATAFYRSMPVIEFIAEVLELPVQALAERRALSDAQRVKFTKEIRGLKIEITHCGQMRRKYRVCNVTRRPAQTQTFPLQLETGQTIECTVAKYFYDKYRLQLKYPHLPCLQVGQEQKHTYLPPEVCNIVPGQRCIKKLTDVQTSTMIKATARSAPEREREISNLVRKAEFSADPFAHEFGITINPAMTEVKGRVLSAPKLLYGGRTRATALPNQGVWDMRGKQFHTGIDVRVWAIACFAQQQHVKENDLRMFTNQLQRISNDAGMPIIGNPCFCKYAVGVEQVEPMFKYLKQNYSGIQLVVVVLPGKTPVYAEVKRVGDTVLGIATQCVQAKNAIRTTPQTLSNLCLKMNVKLGGVNSILLPNVRPRIFNEPVIFFGCDITHPPAGDSRKPSIAAVVGSMDAHPSRYAATVRVQQHRQEIISDLTYMVRELLVQFYRNTRFKPARIVVYRDGVSEGQFFNVLQYELRAIREACMMLERGYQPGITFIAVQKRHHTRLFAVEKKDQVGKAYNIPPGTTVDVGITHPTEFDFYLCSHAGIQGTSRPSHYHVLWDDNNLTADELQQLTYQMCHTYVRCTRSVSIPAPAYYAHLVAFRARYHLVDREHDSGEGSQPSGTSEDTTLSNMARAVQVRLLVHPDANNVMYFA

***Caenorhabditis japonica* ALG-1**

MYVCAAAAAAAATATVVAARSNFHSASVSLNCPVKPANASNEPMSGGPQYVQGALSSALPVTQSGAPGTSPTSSSFVPTSGTSSSPFPPSAASTALQNDLEEIFNAAPPLQLSNVPQRQAGSLAPGAPIGNTGVAIGDTSSSIGGQLQGGGASGQAPGGMQSGVQFQCPRRPNHGVEGRSILLRANHFAVRIPGGTIQHYQVDVTPDKCPRRVNREIISCLIMSFSKYFSNIRPVYDGKRNMYTREPLPIGRERMDFDVTLPGDSAVERQFSVSLKWVGQVSLSTLEDAMEGRVRQVPFEAVQAMDVILRHLPSLKYTPVGRSFFSPPVLSSSGALAAPGVVGAAQSPGQYHAESKLGGGREVWFGFHQSVRPSQWKMMLNIDVSATAFYRSMPVIEFIAEVLELPVQALAERRALSDAQRVKFTKEIRGLKIEITHCGQMRRKYRVCNVTRRPAQTQTFPLQLETGQTIECTVAKYFYDKYRIQLKYPHLPCLQVGQEQKHTYLPPEVCNIVPGQRCIKKLTDVQTSTMIKATARSAPEREREISNLVRKAEFSADPFAHEFGITINPAMTEVKGRVLSAPKLLYGGRTRATALPNQGVWDMRGKQFHTGIDVRVWAIACFAQQQHVKENDLRMFTNQLQRISNDAGMPIVGNPCFCKYAVGVEQVEPMFKYLKQNYSGIQLVVVVLPGKTPVYAEVKRVGDTVLGIATQCVQAKNAIRTTPQTLSNLCLKMNVKLGGVNSILLPNVRPRIFNEPVIFFGCDITHPPAGDSRKPSIAAVVGSMDAHPSRYAATVRVQQHRQEIISDLTYMVRELLVQFYRNTRFKPARIVVYRDGVSEGQFFNVLQYELRAIREACMMLERGYQPGITFIAVQKRHHTRLFAVDKKDQVGKAYNIPPGTTVDVGITHPTEFDFYLCSHAGIQGTSRPSHYHVLWDDNNLTADELQQLTYQMCHTYVRCTRSVSIPAPAYYAHLVAFRARYHLVDREHDSGEGSQPSGTSEDTTLSNMARAVQVHPDANNVMYFA

***Caenorhabditis remanei* ALG-1**

MSGGPQYLQGVMNPLQSPSPPNSSPSSSLVTTAAIPTTASSQVVPTSGATQQPPLPSAQTAASTALQNDLEEIFNSPPQPQTFSDAPQRQAGSLAPGAPIGSTGVAIGEQSSTIGGTLQGAGAPGNAPGGAQSGVQFQCPRRPNHGVEGRSILLRANHFAVRIPGGTIQHYQVDVSPDKCPRRVNREIISCLISSFSKYFTNIRPVYDGKRNMYTREPLPIGRERMDFDVTLPGDSAVERQFSVSLKWVGQVSLSTLEDAMEGRVRQVPFEAVQAMDVILRHLPSLKYTPVGRSFFSPPVVTGSGGIAGSSPPAQAAPSISSGSHSAGQYHAESKLGGGREVWFGFHQSVRPSQWKMMLNIDVSATAFYRSMPVIEFIAEVLELPVQALAERRALSDAQRVKFTKEIRGLKIEITHCGQMRRKYRVCNVTRRPAQTQTFPLQLETGQTIECTVAKYFYDKYRLQLKYPHLPCLQVGQEQKHTYLPPEVCNIVPGQRCIKKLTDVQTSTMIKATARSAPEREREISNLVRKAEFSADPFAHEFGITINPAMTEVKGRVLSAPKLLYGGRTRATALPNQGVWDMRGKQFHTGIDVRVWAIACFAQQQHVKENDLRMFTNQLQRISNDAGMPIIGNPCFCKYAVGVEQVEPMFKYLKQNYSGIQLVVVVLPGKTPVYAEVKRVGDTVLGIATQCVQAKNAIRTTPQTLSNLCLKMNVKLGGVNSILLPNVRPRIFNEPVIFFGCDITHPPAGDSRKPSIAAVVGSMDAHPSRYAATVRVQQHRQEIISDLTYMVRELLVQFYRNTRFKPARIVVYRDGVSEGQFFNVLQYELRAIREACMMLERGYQPGITFIAVQKRHHTRLFAVEKKDQVGKAYNIPPGTTVDVGITHPTEFDFYLCSHAGIQGTSRPSHYHVLWDDNNLTADELQQLTYQMCHTYVRCTRSVSIPAPAYYAHLVAFRARYHLVDREHDSGEGSQPSGTSEDTTLSNMARAVQVRFENLKKLSIYTIKTLQVHPDANNVMYFA

***Haemonchus contortus* ALG-1 (Likely exon-intron boundary issue with predicted protein)**

HRSQSALVFPHPISFVLFLQDLQFHALFQEQSEPLP*SWSTFVYQLIS*CIVALALRDILLVANKYASVLVQPPEGMEDVDTSAECDICHRSTSPLCIQWSDLSPIVVGS*VVLSYVPGSAKISCQLLRFDHPINVNFT*VHTCGEVVLLNTAGAS*CK*RDVFCVLFRCSFTSGRKLGPWGTYWCVCNGILSIHARHRVRRRSFGASRAGAS*SKACRRYRAWNRNTMRPS*KRDQNYTTDSVELVPVFQVCHGSRSS*ADVQILEANFLGNSTCSCYSARQNTRLW*RCWTRTEAYLFATRSMSRSARATMHQEIN*YTNVDHDQERDYLLHDPLFWQVLQYK*TGL*WKEEYVHPGTVADWTRKDGV*GTREWSFPLQLETGQTIECTVAKYFYDKYRIQLKYPHLPCLQVVLQYELRAMREACMMLESGYQPGMTFIAVQKRHHTRLFAVDKKLKVWAIACFAQQQHVKENDLRNFTAQLQRISNDAGMPIVGQPCFCKALTERRALSDAQRVKFTKEIRGLKIEITHCGQMRRKYRVCNVTRRPAQTQTYVINLGAHFECPRRPNHGLEGRSILLRANHFAVRMPGGTIQHYHVDVSPDKCPRRVNRSVRYAATVRVQQHRQEIISDLTYMVRELLVQFYRNTRFKPRRIVVYRDGVSEGQFYYVCSIALVTLPGDSAVERQFTVTVKWAGQVSLSTLEDAMEGRIRQVPFEAVQAMDVILRHLPSLKMCHTYVRCTRSVSIPAPAYYAHLVAFRARYHLVDREHDSGEGSQPSGTSEDTTLSNMARAVQVVAVTKICQSLILVPVFKDQVGKAFNIPPGTTVDVGITHPTEFDFYLCSHAGIQGTSRPSHYHVLWDDNQLTADELQQLTYQVCATARSAPEREREIASLVRKAEFSADPFAHEFGIAINSAMTEVKGRVLSAPKLQYGGRNKATALPNQGVWDMRGKQFHTGIDVKV

***Meloidogyne hapla* ALG-1**

XPDKCPRKVNREIVNTMVDSCKLFTGFKPVYDGKKNLYTKEALPFGQERIELDVVMPGDSAVDRKFRVALKLVSRVSLQALEDAMAGRIRQIPPESVQAMDVILRHLPSMKYTPVGRSFFSSPPLALGAQHITVSGGIGGGGGGPQGVDKLGGLGGGREVWFGFHQSVRPSQWKMMLNIDVSATAFYRKMPVINFMAEVLELPMQALNDRRNLSDPQRVKFTKEIRGLKIEITHCGQMRRKYRVCNVTRKPAQTQTFPLLLENGLSIDCTVLKYFNDKYHMQLKYPHLPCLQVGQEQKHTYLPLEVCEIVSGQRCIKKLSDTQTSTMIKVTARNAPDREKEISALVRRAEIVHDPFAQEFGISINSNMTEVKGRVLNAPKLLYGGRTKATALPNQGVWDMRGKQFHTGIEVKTWAIACFAQQNHVKEADLRNFTGHLQKISADAGMPIQGQPCFCKYAVGVDQVEPMFKYLKQNYPGLQLVCVVLPGKTPVYAEVKRVGDTVLGIATQCVQAKNATKTTPQTLSNLCLKMNVKLGGVNSILLPAVRPRIFNEPIIFLGADITHPPAGDSRKPSIAGVVGSMDAHPSRYAATVRVQQHRHEIISELTFMVRELLIQFYRNTRFKPTRIIVYRDGVSEGQFLNVLQSELRSMREACMMLERGYQPGITFIALQKRHHTRLFAVDKKDQVGKAFNIPPGTTVDVGITHPTEFDFYLCSHAGIQGTSRPSHYHVLWDDNNLSADELQQLTYQLCHTYVRCTRSVSVPAPAYYAHLVAFRARYHLVDREHDSGEGSQPSGTSEDTTLSNMARAVQVHPDANSTMLGHGHNTRDTVCLEFL

***Meloidogyne incognita* ALG-1**

MQIPQQQQQQQQMTSIDQQAVLSILEALTVNESGGMPPTFVPQMTGMIGGGPFGGGITGGVSSQFSMGSQSMQQGAPPFSQQPPQYPESSFFMPPHLLSSGSQMVHGSGGVGGGGGSAFTSATTPMLYERQPQQQPQQQQPQQQPSTISSQQGQQIAMAATTEISGGGGGFQNPPPGQQPPIGQQQQQQRSLVPVQHGQLSLPPSTIQFQCPPRPNHGTEGRPILLRANHFKVTNSGGYVHFYAVEIQPDKCPRKVNREIINTMVDSCKLFNNFKPVYDGKKNLYTKEALPFGQERVELDVVMPGDSAVDRKFRVALKLVSRVSLQALEDAMAGRIRQIPPESVQAMDVILRHLPSMKYTPVGRSFFSSPPVALGAQHITVSGGPGGGGGGGGGGGPQGVDKLGGLGGGREVWFGFHQSVRPSQWKMMLNIDVSATAFYRKMPVINFMAEVLELPMQALNDRRNLSDPQRVKFTKEIRGLKIEITHCGQMRRKYRVCNVTRKPAQSQTFPLLLENGLSIDCTVLKYFNDKYHMQLKYPHLPCLQVGQEQKHTYLPLEVCEIVSGQRCIKKLSDTQTSTMIKVTARNAPDREKEISALVRRAEIVHDPFAQEFGISINSNMTEVKGRVLNAPKLLYGGRTKATALPNQGVWDMRGKQFHTGIEVKTWAIACFAQQNHVKENDLRNFTGHLQKISADAGMPIQGQPCFCKYAVGVDQVEPMFKYLKQNFPGLQLVCVVLPGKTPVYAEVKRVGDTVLGIATQCVQAKNVTKTTPQTLSNLCLKMNVKLGGVNSILLPAVRPRIFNEPIIFLGADITHPPAGDSRKPSIAGVVGSMDAHPSRYAATVRVQQHRHEIISELTFMVRELLIQFYRNTRFKPTRIIVYRDGVSEGQFLNVLQSELRSMREACMMLERGYQPGITFIALQKRHHTRLFAVDKKDQVGKAFNIPPGTTVDVGITHPTEFDFYLCSHAGIQGTSRPSHYHVLWDDNNLSADELQQLTYQLCHTYVRCTRSVSVPAPAYYAHLVAFRARYHLVDREHDSGEGSQPSGTSEDTTLSNMARAVQVHPDANSVMYFA

***Oesophagostomum dentatum* ALG-1**

MYVGIDVTHPTANSGIDISIAAMVSNFDLAATRYTNEIFAQMAARETVERFEYQFRRLMIKFQKHTGVWPRHIVVFRDGVSDSEMLRTAFIELKCIRDSWTTLTHADPDLDVTYTYIVIQKRHITRFYQPSGKDDKGNATYVNVLSGTVIDNTVVSPKLFDFYLASQIGAIGTTRPAHYTVVVDEWMLSADQIYEMCYKLCFLYARCRIPVSLPCPVYYAHLVCEKAKEVYKTLNSRHEFDGVEELELRKNEIERRLAVHEAYPGMHFV

***Pristionchus pacificus* ALG-1**

MMMNSAAAASASAQQTDLAGSLAPGGPIGDAPSAPGGQPVQQGGQAGPGSGSGSGGSGPGAGAQFQCPRRPNHGVEGRAILLRANHFAVRIPGGTIQHYQVDVQPDKCPRRVNREIIATMIRSFSKYFGTIRPVYDGKRNMYTKEMLPIGRERTEFEVTLPGDSAVERQFTVVIKWMGQVSLAALDDAMEGRVRQVPYEAVQAMDVILRHLPSLKYTPVGRSFFSPPMQAPPQMAQHGQYNTESKLGGGREVWFGFHQSVRPSQWKMMLNIDVSATAFYRSMPVIEFIAEVLELPVQALAERRALSDAQRVKFTKEIRGLKIEITHCGQMRRKYRVCNVTRRPAQTQTFPLQLETGQTIECTVAKYFYDKYRIQLKYPHLPCLQVGQEQKHTYLPPEVCNIVPGQRCIKKLTDTQTSTMIKATARSAPEREREISSLVRKAEFSADPFAHEFGIAINPAMTEVKGRVLSAPKLLYGGRTKATALPNQGVWDMRGKQFHTGIDVKVWAIACFAQQQHVKENDLRMFTTQLQRISNDAGMPIIGQPCFCKYAVGVDQVEPMFKYLKQSFQGIQLVCVVLPGKTPVYAEVKRVGDTVLGVATQCVQAKNVIKTTPQTLSNLCLKMNVKLGGVNSILLPAVRPRIFNEPVMFLGCDITHPPADSRKPSIAAVVGSMDAHPSRYAATVRVQQHRQEIITDLTYMVRELLVQFYRNTRFKPSRIIVYRDGVSEGQFFNVLQYELRAMREACMMLERGYQPGITFIAVQKRHHTRLFAVDKKDQVGKAFNIPPGTTVDVGITHPTEFDFYLCSHAGIQGTSRPSHYHVLWDDSSLTADELQQLTYQMCHTYVRCTRSVSIPAPAYYAHLVAFRARYHLVDREHDSGEGSQPSGTSEDTTLSNMARAVQVHPDANNVMYFA

***Trichinella spiralis* ALG-1**

GTILGPGPRHPGKGGKGGKIPHRFVYRKRPEFLNLCDSTYRAHGIWEFGNPILPMCTRSLKGRDQKLQQYQIKGVWGHARQSSFTRGVENKNLGNCLNFTQQLLRISNDAGMPVVGQPCFCKYATGVDQVEPMFKYLKQTFHGIQLIVIVLPGKTPVYAEVKRVGDTILGIATQCVQAKNVVKTTPQTLSNLCLKINVKLGGVNSILLPSIRPRIFNEPIIFMGADITHPPAGDSKKPSISAVRIE

**R06C7.1**

***Ancylostoma caninum* R06C7.1**

HTYYPVELLRVAPSQRVTLQQQTPDQVAAMIKACATLPQNRLHQTKLLKDALAIKEGNPHLSAAGISVVNGFTSVPGRVLPSPSIVYGGNQLVKPVDNCKWNGDRSRFLEPARLHNWAVCATLTQNDSRRLNVKEYVAKIEGRCRQRGMDVEPCAEIFNLQRQNFESLKEWYASQKAKNRRYLMFLTSDGIKQHDLIKLLEIEYQIVSQEIKGSKVDAVVSRNQNQTLDNVVAKINEKLGGVNYNIMLGARPTDDVNKWISDKDRMFVGFEISNPPALSKVEIERGATYRMPSVLGWGANCAKNPQQYLGDYVYIEPRQSDMMGAKLSELIVTILKRFRAATDVAPRHIVLYFSGISEGQWSMVADTYMRAIHTGIKSLSASYKPSLTALTVSKDHNERIYKANITGNRATEQNIPPGTVVDTKIVSPVINEFYLNAHSAFQGTAKTPKYALVYDDSNIPMNVVEGMTHGLCYLHEIVTATVSVPVPLIVADRCAKRGHNVYIANSNQRDAVGSIKEANERLVNQGELQKVRYNA

***Ascaris suum* R06C7.1**

TVVGMAFTCGGPFAMRGDYWMQEPRVATVQCLKSHVVNALNFFKEESNIKAFPEHVVVFRGGVSEGEYAKVMSEEAGAFRNAFKDVTGDNGTKIRLSIICVQVNSNYRLFRDNIPATGNAAQQNVPPGTCVDTAIVHPTQTEFILVAHKSIMGTARPIRCTVLVDDKPRMTLDEVEGITNALCYAHGIVT

***Brugia malayi* R06C7.1**

MKYIESQEQILTQDLKASTALGIVMKRQYQTLDNIVNKTNIKMGGLNYSVHLEENCDRWLGRPGFLIVGLDIAHPSYSRVPNKDRNTVLSVVGYSANIKKHPLDFIGGYRFAKAQMEELMDDAIQQIFSDLLRYFNANRGTPPTHLFVIRDGISVGQYKYVMNTEVEQIKHACQLVGGQNYRPHITFIVLTKMHNLRIYKKNIHKQERAAQQNIKPGTVIDKHVVNPVLSEFYLNSHSTFQGTAKTPRYTLLFDTSKMEADEMQGIVHALAYNFQIVNMAXSLPSPVMIASRMAKRGRCNYVAML

***Caenorhabditis brenneri* R06C7.1**

MPPVPPAPPMPPMPPVTVPPMTPMTPAPADQQKLFQSTGNEACIRRLQQLNVEPGARAYPKPTEPGKMGKPTDVQTNIFGIEVEKETTVFRYMVHAKADLTPTKEATFTKKGKEDFVVLDRREKCCNIFFYAVDNNSDFFKLKDGNQIIYDGQSTLYTTMNLFSQCDPNEKKAKVFAIPGESIDNDDLKSLKLITLEVYAPRDNILVISAENLGKRTADQNIEKNNREYTQFLELALNQHCVRETDRFGSFEHGKVYFLKALQEGFDQRDCVDVGDGKTLYPGIKKTIQYIEGPYGRGQNNPSVVIDGMKAAFHLEQNVYEKCNYIVGKDVGNGMTDYDREKAAQVIKGLDCYTSHTNRVRHLRIDGIHHESARAARFELADGKHCTVQQYFQDKYKLTLKYPNANLLVCKERGNKNFYPLELMTITKNQRVTIPQQTSQQSQKTTKECAVLPDVRQRLIVTGKNAVDITTENELLKALGLKVYPEPLMVRARELDGKELMYDKKVIAESGKWRAPPGSFNKPAAFPDLWALYAVGTQNSRFSANDLNQFSMMFMETLHKKGLAVKGPAESSLLHVDQIMNKLQSVADSKCKFVFVITDDSITHLHGKFKALEQKTEMIIQDMKMTKAISVCRDGKRLTLENVINKTNVKLGGLNYTVNDIKKSMTDDQLIIGIGLSAPPPGTKFIMEGKGHLNPQVIGFASNAIATHEFIGDFVLAPVGQDAMSSIEDVLKTSLSSFENNRKKLPKRIVIYRSGASEGSHSAILAYEIPLARAVIQSFSEDIKLIFIVVTKDHTYRFFKDQLRGTKATEMNVQPGLVLDTSVTNPACKQFFLNSHTTLQGTAKTPLYTVLADDCNAPMDRLQELTFTLCHHHQIVALATSVPTPLYVANEYAKRGRTLWVEKTEGAPIEAEGSESNRLKDITKELAYQMSTLHNKRINA

***Caenorhabditis briggsae* R06C7.1**

MPPHPPAPPMPPMPPMPPVTAPPMTPMAPIPTNPQELHQKSGNDACIKRLGQLNVEAAPKVYLKPSEPGKVGKPADIQTNIFGIEIEKETTVFRYMVHAKAELSPTKEAVFTKKGKEDFVVLDRHEKCCNIFFYAVEKNPDFFKMKDGNCIIYDGQSTLYTTVNLFSQLDAKEKKSRIFEINGAEIENQELKSLPCISLEVYSPRDNALVISSETLGKRTADQNTESNNREYTQFLELALNQHCVKQTDRIGCFEHGKVYFLRATEEGFDARDCIDVGDGKVLYPGLKKSIQFIEGPYGRGQNNPSVVIDGMKAAFHKEQNVLEKFSEIVNRDVSRGMSDNDRDKASQVIRGLDCYASYSNRTRHLRIEGIHQDCAAKARFELSDGKSCTVQQYYHEKYNIALKFPGANLLICKERGNKNFYPAELMTITKNQRVTIPQQTGQQSQKTTKECAVLPDVRQRLIVTGKKAVNITEENELLQALGIKVYPEPLMIKARELEGKEIVYEKKTSSDAGKWRPPPGCYNKPAVFPDLWAIYAVGTQHSRFSVQDLNAFCSMFMDTLQRKGITVKTPAETSLLHVDQAMSKLENIAMNKCKYVFIITDDNITHLHREFNQTRLAVYNIFREMSKATSVCKDGKRLTLENVINKTNIKLGGLNYTVSDTKRSMTDDQLIIGVGISAPPPGTKFLLEGKGHLNPQIVGFASNAVSNHEFVGDFVLASVGQDTMSSIEDVLKNSMEMYEKNRKRFPKRIIIYRSGASEGSHPSILAYEIPLARAIIHGYCKDIKMIYIVVTKEHSYRFFKDQLRGTKATEMNIPPGIVLDNSVTHPACKQFFLNSHTTLQGTAKTPLYSVLADDCNAPMDRLEELTFTLCHHLKSLHSPPRSQLRCMWPMSTPKEGAHFGLRRRKLLTTITTINDGAPIEAEGSESNRLKDLTKELSYQMTDLHMKRVNA

***Caenorhabditis japonica* R06C7.1**

MGPMPPVPPMPPVAFPPVAVPPVTVPPTVTPPILHATAGPVPGDVQETMHQTGNDACIKRLRQLNVDIAPKFYIAPNAPGTTGKQTEIQSNIFGVELEKDVTVYHYMVHAKADLASNKEAVFTKKGKEDFVVLDRHEKCCNIFFHAVETNKDFFRMDEGNRLIYDGQSILFTTIDLFPDQEKKDRIMEINGALTKNKDLESLPIIQLEVYASKIGSMQISSQVLASRTADSNIEANNRAYAQFLELAMNQNCIRETSRFGCFEHGKVYFIQPTESGFHSNDCIDVGDGKALLPGIKKTVQFIEGPFGRGQNNPSVVVDAMKAAFHKEQTVVTKIYEILRKDPSNGLTDFEREKAFQVMKGLDVFSTYTGRTRHLKIEGIHHDCPSKAKFELKDGTTQTVEQYLASKYQIQLKYPNANLLICKERGNKNFFPTELLFISKNQRVSIPQQTSVQSQKTTKECAVLPDVRQRLIVTGKNAIGLNEENSLLMDLGIKVYPQPLMINGREMDGKEINYSSGRAVRSEAGKWRAPATLHTPAAMPKVWAAYAVGTGSSQFNAHDMQTFVAQFVDTCNKRGMSVGPPAEFQLVSIDTIEEKLNFAIKSSCKFVFLITDDSITHLHPKIKLLERNSEMIIQDMKMSKALSVVRQAKRLTMENVINKTNVKLGGLNYTVTDSKKSMTEDQLIVGVGVSAPPPGTKFVMEGKGYLNPLIIGYASNAKSNHEFTGDFVLAPVGQDTMASIEDVLKNTLEMYQENRKSFPKRIIIYRSGAPDGAHAAIIAYEIPLARAIIQEFCPTTKMIYIVVTKDHSYRFFKDYISPGSRATEMNIPPGIVLDTCVTNPACKQFFLNSHSTLQGTAKTPLYTVLADDCNAGMDRLEELTYTLCYHHQIVALTTSLPTPLYVANEYAKRGRTLWTEKTHDAPEAAESNGSQHNRYKDLTTELAYKYTTTLGAKRINA

***Caenorhabditis remanei* R06C7.1**

MPPHPPAPPMPPMPPMPPVTVPPMTPMAPTPANQQDMHQQTGNDACIKRLQQLNVEEAPKVYLKPSEPGKLGKPTDIQTNIFGIEVEKETIVYRYMVHAKADISPTKEATFTKKGKEDFVVLDRHEKCCNIFFHAVEKNPDFFKIKDGNNIIYDGQSTLYTTTSLFSVTDTKDKKSRIFEINGADTSNEDLRSLACILLEIYAPRDNSLVISAENLGRRTADQNTEVNNREYTQFLELALNQHCVRETARFGCFEHGKVYFLKPTEEGFDPRDCISVGDGKVLHPGVKKSIQYIEGPYGRGQNNPSIVIDGMKAAFHKEQNVAEKIYEITGREPSKTLNDFDREKASHVIKALDCYTTYSNRQRHLRIEGLHHDCAAKARFELPDGKSCTVQQYFQDKYNVQLKYPEGNLLICKERGNRNFYPAELMNITKNQRVTIPQQTGQQSQKTTKECAVLPDVRQRLIVTGKNAINITEENELLKNLGIKVYPEPLMVKARELEGKEILYDRKVNTESGKWRAPPGGYNKPCAFPDLWAMYAVGTQQSRFSAGDLNNFSNMFMETLQRKGIKVPTPAETCLLHADQIMDKLQSVSESKCKFVFVITDDNITHLHQKYKALEQRTMMIVQDMKMSKAVSVAKDGKRLTLENVLNKTNMKLGGLNYTVSDAKKSLTDEQLVIGVGISAPPPGTKFILEGKGHLNPQIIGFASNAIANHEFVGDFVLASVGQDTMSSIEDVLKSSLDMYEKNRKTLPKRIIIYRSGASEGSHPSILAYEIPLARAIIHGYSKDIKMIYIVVTKEHSYRFFRDELRGSKATDMNIPPGIVLDSAVTHPACKQFFLNAHTTLQGTAKTPLYSVLADDCNAPMDRLEELTYTLCHHHQIVALSTSVPTPLYVANEYAKRGRALWAEKTEGAPIENEGSESNRLKDLTKELAYQQTDLHNKRVNA

***Haemonchus contortus* R06C7.1 (Likely exon-intron boundary issue with predicted protein)**

ICAHTTIYRTC*RSQSLYGRT*RNDIRAHLRSSNCKSPYLSSNASVCCESLCRTRAQHLQ*SVQLQARACGV*EWEGIHEPSMEFRLSSARLSKRWWWEKTLPWYTEECPVYRGARWT*LQ*PCSHH*RYFRYPHAPLVKVRERGRTNNYPMELGFLRPMQRVTIPQQTPDQSQKATRRPTEQNIPPGVVVDEGLTHPSFKEFYLNSHITLQLEPEAPIMAKNAPPGVLGSRYVVQTNAFGVQLEKPMAFWRYDVVISAEIGSAKRPVFFTKKGRDEYVVVVHDRRMVTMENILLKANLKLGGLNYEIDMNGQMPSEEFK*VLPVSWPTNGETSFLILTHEIPLLRGALAALGAKNVKIVFIVAQKEHNVRLMLERVRTVNTMSDIIRNVLPKFAQNHGNKFPRDLIIYRSGLSEGSFSTVYIIMNRNYKCKLVFDAVVRLYSDFFDDPDQLWYDGQSILFSGKDLFMSLTEKELLTKTANSDLSQNDRSITQFMELVFNQMAIRFEDHEGRRISVRDYFEEKYKIRLSEDELKARLKEAAKHNCKYCLIVSADSITTAHSLFCAVPPGERQDNIARGARALKLLGSDDNPFVSNAGLYIYKDPIKVYAANFKANSYDFVGDYLFQSSRREEVGSLHSTQITVLAKKAAFHEELPLIEKAKHILNDNLLERVTEIGLERLNAGMKGLECGVDSHANLYKTLYLSSILHENLIKGEFSFPRLFLGIGVSHPPPSQNFDERSVPSVVGLFQNMIDIFSKLYSELPVGTS*YRPNARCGCRKEVNSSGLNNHGKGWLFSAPSSTF

***Meloidogyne hapla* R06C7.1**

MSKGGDESVASGPALSSLRVATVIEKQTQSYLKDKMVEFGEPIMAERKGLVREGDVDLTTNIYPLIFQGSVRVSRYDVKVLGQKERSGRIVEFTKKFKDDFTKTERRFRTRDVIITFMNKHKDMAKAAGCIYNDLQSIMYSLTPMAPRQDPIKFNFIPSELHNASEFERFKLGTIILEFNPTNPFQLNLEQVQEQRNLLYANRDLAQFLDIATSQEVFFREGEHVHFSTSNSYMYNPESFGFRSDDTVSFEENLTYLGIGCDKAIHNVQGGKPGGGGLQAVVVQRMTCFSLVLKKSLAKKTPFHADDLLINKVDAMLRSLQRQSLDKKVEYLDKYLKGLMVCTTHNKKNVQYLLIKGFTRENASQKEISINGQQTSVASYFNQKYGVSLSRMDLPLVISSTIDKEGKKQQNYYPIEVLQVCDNQRVKTNQLTPAMTQAAIKKCAVPPAILRAQMEKVARSLHLWDSSYLDSAKIKVLETPLQLRAHELNHPELQTGDGSSFPNKDTAGWRMNKVFSPQAIIGKQYGWASFVFWPKSSGQGCLSQSEMDDFLRRYQQQANSRGIRLDNPVKKEIVMMNKMDDMARVYDWAAKNDIAFLLCFHGDGGDAEQVHHEVKANERRFFVITQCVRTGTVRNVITKNQRLTIDNILNKTNVKLGGLNYTLSRSRIATDTSTLIIGFSANHPGGGIGTVDDTASDPNRAPSFVSGPPTAVGFAANVGPTKMAYDFIGDFLYQQAQREEKVNVIESIVQRCVAFFRSTRDGALPKRVLLFRNGCSEGLYPSILKYEVPLLRKALQDEGIQEPRLTLIVCNKLQSVRFFYKNINSNLKAPDQNLKPGTIIDTSAVHPEFAEFFLTSHRALQGTARTPKYTVVYDDCEQDLDELERITYDLCFGHQIVSSPISLPAPVLVASEYAKRGRNLYNSLSDELKQKNPDIKYSDLNDEITFYGREGTECWISEYRVNA

***Meloidogyne incognita* R06C7.1**

MSKGGDESIASGPALSSLRPANVLEIQAREYLKEKEIEFGLPIMAERKGPVREGDVDLTTNIYPLIFQSNIRVCRYDVKVSGTKRNGRIVEFTKKFKDDFTKTERRFRTRDVLVTFMNKHKDMTKAAGCIYNDLQSIMYSLTPMSPQEKHIKFDFIPSELHNASEFERFKLESITLEFNPTNPFQLSLEQIQGQRDLVYANRDLAQFLDIATSQEVFFREGEHVHFSTSNSYMYSPEMFGFKSDDTISFADNLTYLGIGCDKAIHNVQGGKPGTKKTPFHADDLLVNKVDAMLSSMQRASFDAKVAYLDKYLKGLMVCTTHNKNNVQYLLIKGFARESASQKEISINGQPTSVATYFNQKYGVSLSRMDLPLVISSTTDKDGKKQQNYYPIEVLQVCDNQRVKTNQLTSQMTQLAIRKCAVPPAILRAQMEKVAKSLHLWDSEYLNAARIQVLNKPLELRANELNHPELQTGDGSVFPNKDSAGWRMSKVLSPQALVGKQYGWASFVFWTKNAGHGCLMQNEMDDFLRRYQQQASFRGIRLDNPVRKEIVTLNKLDDMAQFYEWASKNDIAFLLCFHGDSGDAEQVHHEIKANERRFFVITQCVRTGTAKNVIQKGQKLTIDNILNKTNVKLGGINYTLSRSRIAADTSTLIIGFSANHPGGGIGTVDDTASDPNKAPSFVSGPPTAVGAFQFAANVGPTKMPYDFIGDFLYQQAYREEKVNVIESIVQRCVAFFQSTRDGRLPERVILFRNGCSEGLYPKILKYEVPLLRKALQVEGMQEPKLTLIVCNKLQSVRFFHKNINPNVKAPEQNLKPGTIIDTAAVHPEFAEFFLTSHRALQGTARTPKYTVVYDDCEHDLEELERLTYDLCFGHQIVSSPISLPAPVLVASEYAKRGRNLYNSLFDELKQKNPDITYTNLNDDITFYGRTGTECWISEYRLNA

***Oesophagostomum dentatum* R06C7.1**

MIRACATLPQNRLQQTRVLKDALGIKDGNPHLSAAGISVVNGFTSVPGRVLPSPSIVYGGNQLAKPIDNCKWNGDRYRFLEPASLRNWAVCVTLTPNDSRRLHVKDYVARIEGRCRQRGMEVEPCSEIFTLQRQNFDSLKEWYASQKAKNRRYLMFITSDNIKQHDLIKLLEIEYQIVSQEVKGSKVDAVLTKNQNQTLDNVVAKVNQKLGGVNYNVMLGANPNEAVNKWISEKDRMFVGFEISNPPALSKVELERGGTYKMPSVLGWGANCAKNPQQYLGDYLYIEPRQTDMMGAKLSELIVQILKRFRGATEVAPRHIVLYFSGISEGQWSMVADTYVRAIQTGIKSLSPNYKPNLTALTVSKDHNERLYRANITGNRASEQNVPPGTVVDTKIVSPVINEFYLNAHSAFQGTAKTPKYALVYDDSHIPIDVVEGMTHGLCYLHEIVTATVSVPVPLIVADRCAKRGHNVYIANSNQRNAVGSIDEANARLVNQGELQKVRYNA

***Pristionchus pacificus* R06C7.1**

MSEEYEKNVKAQKDEADKRMKAKLDEARNLILKMEVGNDNHMPPPIAPGGAGKGTVDIQTNLFGIRMSEDLRIFKYDVFITSEIGLKPKHVEFTKKGKSDYIVTDRKRSCMMVFSAGIKLMSFFRDDMMYYYDGQSLMYTTERLVFKDTTKPNETVVIEGSKADKDLAAFKYIFLAIKECSDGPHETVPMKEGKVLIPGMNKAIKLIEGPRGRHNASPALSIDSLRVAFHMKEKLIDKAMGIIPDINKATPRDIETFTAMVRGLGCSTIHTQVKRRLEIHGISRENSSKRVEVLLPGELNRNMTVQEYFMVKYHLELKYPLAPLVMSREKGQVNLYPMEVLEVIPMQRVTIPQTTPAQSQECAIPPGARQNAIIEQCKLHDLFNPDQPYLKAAGLGIVEQPLRVTARLLKGPVIRYKHGEMEIFQPAWRPPMQYQFAVPAKCRKWGLYAVLNPRDTFGVPEMNKFAEAFVKRCKERGMQIEKAFEVIVLGQPPHEKSADYPAREKIKEGPFGNYDLMFFISADNISIHKHLKLQERYSNVVTQDLKLKTAQNYLEKGQSLTLDNIIYKTNLKLGGMNYNISLKNACPATFNPFPANRLVIGIGISHAPQANLLDKNGVILPAPAVVGYAANMGDHGSTNFIGDFYYQDNKKDDRCCGLLLCTVEMMQNVMDHFLGKVDGEGKRGAAKREPPKEVLVYRQGISEGFYSMCMREEIPLMKAILAHNGAKDVKITYMVVQKEHNIRLMPVDINITEKPTIQNIVSGVVVDSQLTHPRYREFYLNSHITLQGTARTPRYTVLHDDNEMSMNELEMITYGLCHKFEIVNSPISIPAPLYIANRYSERGCALYLAKATDEMASTEQKKRTSMELLNEDHTFGKTKLGGVRINA

**C04F12.1**

***Ancylostoma caninum* C04F12.1**

HFPMELLEVAPNQRVSLEKQQIANSTPRADKPNVRFEKIHKLLEALNLHDSGYKNQFLKAFGIEVAYAPKRVEGFRRPAPGIAFGGNRISQVDQAKCNWKQERDTKYVSCASVERIIIVHSDQSLRLPGNVERALSMTFKSRGIRCGRFEKILINGGREQEMEKQLQQVFEKYKSSKSVLIIYIDRAENKSHEFLKLMERKYLIPTQQMTAELAEKILEQRQSCGNFVSKTNLKLGGINYEVIPESFAKNRWIAGGKTMVVGYDVAHPGKPTRDEVMNKMPPQKPSVVGFSFNGAAHPECFIGDYHFQFPRREKVDDRVLNARFKWMLELFTKNREVWPENIVITRDGVSEGQYRMVIEDELGAIKEACEEFGKLNGRDPWMPRFTVVVATKRHNARFFAEKGRFIENPQPATVVDTDVVRNDITEFYMQSHRPVQGTAKPTSYQLIVDENDMGSDEVQSLMLALCFHHQIVDAPVSIPEPVYQADEWAKRGKDIWKAYTDRHNLLKMADKGQYADYPIDFEAMTNRLAYWGTELQTSRVNA

***Ascaris suum* C04F12.1**

MQPRLRAIFEPGQYGFNLVRSDEIFSFNGARNPETFIGDYAYHEPRKEQVTTSILEERAYWMVKLFTEHRGSLPKLVIITRDGVSEGQIKMVVEEELDAIKVGIRNYIEHSQKPTDREPKYVVVIATKRHNKRFFLEMRNGMLLPYLYEIVLIDFVECN

***Brugia malayi* C04F12.1**

MEMIGRELWTGINSDTLSRRPPTFERVDTAFRAGRHVSADTRSLQSASSRKNASVLQLRIHESTSPSVSDDSFSASEGAVKCCNSIRSSAISDDCHSQTSFSDLMDSDLLETENLNIHQSVKPEHSRGHIQSAGDQNMFHXRRPTPCLMAPKLPPAHQRETVIVKTNIYPLEVGNQIVYRYDXRMYASRADTSKERTTDLCRGERDDSGVTLRHQKCMLLMRRALELYRVLNESGAYLYDLSNTLFTNEPLNKELLPRLKISMEQLTPKLRDLIGACDVIVEITPCAENSHTFNVIDYCDSVTNDLARQNRSFRQFFEILTNNSALQKGTHYAFGCGKLFLVDGKKYKLGEQPLSESRTLITGVEKGIRIIERSSGSIIPALVIDCMTVKKAAFFDAVPLADMAHNVLMKNHLSSPIPPLDANLFNHFVRKLNDIIRGIIPEEYINIFDCNIQAAPTSHLLHLVCQTSLSDRLGMLITGYKTWLGVPFRLSVGKNTPPIPMLQYYKQQGYIIRADWPAVRLITNFGTSYFPIEVLRVAPYQRVPVSKQTPSQMKDTIKECAILPHARFRDITRNLQALDLGGTTHYNSFMAAFGVRISSAPITVEGHRRLAPKVRYSDVQGGMSVXDIDARNASWRMNGKAYLVGAHLRCWFILYDDARDGDMVMTFSKTLVRECCKKGIQMMDPIIKNVQFEQLEDCLKXIRETYPNERAFVMYIDSRDDTHDDLKLYEALHRIVTQHIHGSRAREAPKKVATLENIVNKLNCKNFGQCYGIIPESFATNKWISTGKTLIIGYDVCHPEPQPRHERRMRMPPTQPSVLGISFNGASCPETFIGDYSFQEPRKEQVTSSILEERIYWILSLFYGSRNGMLPETVIITRDGVSEGQFKMVMEDEVEAIRQGMRNFGKAEAGADNYSPNIVCIIACKRHNKRFAVENNHSLGNCLPLTVIDRDITRPDTTEFFMQSHKIIKGTGKLPAYSMPINEANLTMDEAQSLMMALCFTHQIVNQAISIPEPIYQADEWAKRGRNNFRAMLRRVGGKERLPTLDGGSIDWNRVTRRLCYMDTELELTRANA

***Caenorhabditis brenneri* C04F12.1**

MPSKNNKKLAAAERAAKLRAEAEETAATNPVVEEAVETPADNAPVAPPSSEMAALAVTTEKPSIIPSTSEQKKKKVSAPYKPPAPLTPLTSTGSKTVITNSYRMEIKKHTNRRYDVTVSKNVDGRVTEIVGARGADKQQQNSLFQLITGLLKDDRMDLKNISYDGALSLYNSSNQELAGRTFFLNSGDLPQNLRSTLFPRNENGTLDILIERNAQQPTLDTQDVLQENLNGASCPVTQMLQQVMHQEAKRNGFVVVDNGTEMFERPRGEANNGVHEMNGIGAGLKIAKGGDGKGAVHIVMEFKTTRFFDNGPLSALGSRAKFNDTFHAQRYLSGLKVGTTYSNQVITIHGLSSDSISELTYDGNSILARSAEVAHRDVREYNGNWPAVQTRIRDRRTRQFRVFSFPIENLRVLPDQKLMPKHGSPPTCPGPEVRFAQTTTVGQSAKLLAPNPTLASFGGQILPTPLTVQAVEVPAPTILYKDSQARVIQAMVNRQSVSFEQKNFISIQFFQANWRKPAGAKFFEPASPFKVLILYNSVYLTSQQRGSIGEKVEDVKTQLQSMAARGTGLLITVSGTEDLGGTFGDEVSAGEAIRMKLETLKDLPANEKPVVIYIDPSTSQTHGVLKLQERLCEVVTQQLSIDKSLKRSIGGATLTNFLLKLNQKRGGLNHKVQPDAMIAHLYGEKSNTLIISYDVCHSSGRVYVRGELCDEPSCVGFAYNSTKSREMFIGDFAYQLSRHERVDEQILKEKTKDILNKYGISHSKAPSSIIILRDGVSEGQHQMVYDDEFPSIEQAVTEFVQEKNKFFKEKKMNIVLSQPSIALLVITKRHANRLYEKNEQGAIKNVSPLLAVDTVVVKKAGNEMLFVSHCPLKGTAQFIAVHIIVNQKVFSKNDEIVRLLAALSCARQVSTSVVSLPESIYAADDYAKRGADLFREWRLLMGAEVPTLHTENGMQFDWRAITERLNYQNSIFKNIRIA-

***Caenorhabditis briggsae* C04F12.1**

MPSKANKRKAAAERSAQLAAEAVDTPGSDVTPANVPATAPVSAPTRAAPPTEALAALGISTDAPSTPDQSSPSYGKKKHVPYELPAKLTPLQKTSSFKVITNSYPMEVEKKTCYRYDVHVLARQEDGKTIELTGSKGSDRQRQSELHEIINIVLRQEGTQGITCIYDGAATIYANGQMKNKKTGAAGSIQTTVESSKVSEEVRRNYFCNGQRGSFHVLIEPNTAQSTLLTTDVLHESLNSTSCPVTQMCQIALGEEARLKGFMSTEGGNELFDKPNLARSREGIETMDGVGASVKIATGQTGKGAAHVVIDYKKKQFFAAGPLVNHGINWNDSAEAKKRIKNLKIGTTYSSQVIRATGVSKEPMSQLTFIDRDGNEQSVMKTASELSGIPIAKFNKNWPAVQVRKGRVTFSFPIEVVRVLPNQKLTPIHGQPPKCEKAYKRYQLMDAIGKKACLLSPNNTLSAFGMKIQNTPIVVDAVAAKLPTILYKDNSKTSPAIAKQAKWEIGRSSFILPKTVNSIMILFNNDRAENTKKINILIESLSTFAKQLGVVIGSITSKNLAEDGNYNSLRSEDALRTFYAHLKEQLKQQGSSQKPSPYVIYVDHSSEPYHQLLKYLERKFEVMTQHLNFDKALQIRSGSPLLGKSTMTNILMKINLKNGGLNHKVLPDPSIDHLWGDKSNTLIISYDVCHSSGKVYKKGETCFEPSCVGFGYNGTSVPEAIIGDFHYQLPRNEQVDPSVLTLRAKLMTNAYVKARKMWPANVLILRDGVSEGQNAMVRLEEFPAIQKGVMDAFKGNKDKDAKPAFALLVVLKTNSNRLYLRTQTQSGIENVPPMTAVDHTIVKKEGVEMVMVCHHPLNGTAQPVLINMLVNEKVFETNDQIVNLMGATCCAHQVSTGITSIPEPICAADDYAKRGADLFTKYQEENRNNMPTIEGPDGVLQLDFDKITRELCFEFTRFKLNRMA

***Caenorhabditis remanei* C04F12.1**

MPSKSNKKKAAAERSAKLANEATEVAESAPTPTEPPPAAATTPSQPTPEMPKSSKPAPPKELVAPSKIAPLKKNKPLKVTTNSYQMEVKNVVCYRYDVKIVGQADGRNQFLLTGSKGSDRQKQTELTEILKLAMKKENITQLYIYDGAATLHTPAQFNVKKNGASGTIITTIDSTELSPSLRNSYFSKSHGRFDVVVELNSAQPQLHSSDLLKESPDSTSCPVTQMIQIALCEEAKRNNFLITDGGNEMFDRAGVTQIRGVEDMNGVGAGIKIAMGVEGKPAAHLILDYKKKQFFANTPLANLNLDWTNIPKVKNYLKGLKVSTKTNDRPQTFTIDGLSSLLMGQIKYDGGSVLENAMRCTGKPASAFNTQIPAIESRQFSKREGKKKAFNFPMEILVLAPNQKLNPKHGNPPRCERPHKRFELTKTVGEKAHILSPNKILESFGVKIRPEPIVVDAVTVPIPTIQYKTTCTAPDLSKQAKWEVRGPFVEPATIGKILILYNSTNPQDSDKLIQLKGQFARTARECGVTISEIELENLAQTYPGASVLLAIEKKFAELKSLPVAKKPLVIYADYSSSPTHGFLKLQERLCDVVTQQVSFDKSLSRPTIGRSTMINFMLKLNLKSGGLNHKVKPDPSIAHLYGDSSNTLFISYDVCHSSGILYRKGEVCDEPSCVGFGFNGTAHPEAIIGDFHYQLPRTEQVDDEVLKTRANFILTQYATARKKFPDQIVILRDGVSEGQHAMVVNNEFAAIKDGIMTCLKNNKQTKVPALALLIVTKRHANRLFVKDTQGSPLSLVTSLKIMFSGISNVPPLTAIDQGIVRKNGNEVIFVSHCPLQGTAQPIVINMLHNEKVFKTNDELVQLMSVMCCAHQSSTTIVSLPETIYAADEYAKRGADIFQAYKSQPGVVLPTKVTEETGEQLDFLNITQTLCYFKSSFKGRRIA

***Haemonchus contortus* C04F12.1 (likely exon-intron boundary issue with predicted protein)**

VEHSVLNARFKWMLELFLKNRGVWPEHVIITRDGVSEGQYRMVFSFNGAVHHEMFIGDYHFQIPRREKVANVWISRGKTLIVGYDVAHPGRPTRDEIMNKMPPMKPSVVGFRLERSL*MDARIVFEKSRSMA*ACHHYARRSVRGPVQNGMQPSEFLMQICSDSGFFQVVDDELFAIKEACEEFGNKHGRDSWMPRFTVIVATKRHNARFFVE

***Meloidogyne hapla* C04F12.1**

MFKPIVTQHVTLEKVRDIVNKNQRQTLENVLNKMNMKNFGLNYQPQIEDCGKRFALETGKVLVICANMTDNPHAFVGDYFYQESRREALDMRQLADRVSWILRNLGNDENEKSINFFVDWVVEQRRPEHRRPPFIFIIRDGLSEGQFAMACKEELEAIHIGCKEYDPGYRPRFLLIVGTKRHFKKFFIENGPRLTNSANLPPGSVIDQQVVRADLTEFYMQPHVPLKGTGKAIEYAVLENEVDINMDELQAFLMSLSYSHQIVNIATSLTAPVYQAHELAKRGKNNFIAFRKFYRDAVPERELSLPAQQPIRIVDYRGVTHLLAYWGNGTTILSHRFTA

***Meloidogyne incognita* C04F12.1**

MASDNPNKGWSLTKGSVDLNRRISSELVGIYVKKNSLLSPPNAYVYDGRSIFYSSAELQKCDAVSISYSEVSKLIQQTFDRKTKFNVILKPCQTDNRVLDLSDLSQYEGNTSLIQEDRSLRTFLELAIAQKTLESGRLFKRIPDGCPYFRANHITFHSGICKGVRIVKGNNNGKVQAAVVLDAKVSAFLTSQTVEKTINDFMRYCNNQSDGMEKLFNFLRGVKVQCAYNRSRIFSIGGFGDKPLCEEKFERTIDGEICLVSLIDYFNDTKNIRLNNLQFPGVHPAGKPNEKFPVEQLIILEGQKIPQEKQSPDLVDILLRRNSIAPSERHHNIRQSGNELDLWNGKNNVLSAFSIKVQSESNDTNIDVRPLPHLLYRNNREVAPSDDKGDWSRNSGNFLVPASFPQEWLVLFDSRYRELIEDFIKRLREKCFEKGMNFGNARMFPVAQNLFDSQTAMPIATEWRNAFGHCAQEKIPFILMIDSKANDSHGLLKLFEVYRRSKSTKLITQQITTETVENILVKGHRQTLENIVNKLNVKFGGLNYQPILAQPQLPGLKKFEQRFDLSSGNILVIGYDVSHPTYKLHELKKPRLNEPDLEMKMEEDEFTLTSVEPSVVGICANMAENPHSFIGDYFYQTSRKEAVDLNDLKQRSRWILASLEHNRPKQKLPQYVFILRDGLSEGQYEMAYRSELTAIKSGFELHNPKYMPKFVLVIGSKRHFKKFFAVGNDVDKAENMPPGSIIQEKVVRPDVFEFYLQSHFPIKGTGKPVEYAVLTNEINFNCTVEELKEYAKLLRYKTARQDHLEELMFKRSLYSFLMALSFNHQIVSRPISLPEPVFQADELAKRGHANYLALKRFFPNEVPYTNEQHCGHRLADNAQLTRLLSYWGDGQLILKNPAFSHA

***Oesophagostomum dentatum* C04F12.1**

IRFNGAVHPEAFIGDYHFQTPRRERVENVVLNARFKWMLELFKKNRNGWPERIVITRDGVSEGQYRMVIEEELGAIKEACEEFGNLNGREGWKPLFTVVVATKRHNARFFVEHGRGVDNPQPGTVVDTDVVRNDITEFYIQSHRPVQGTAKPTSYQVIVDENGMNSDEVQSLMLALCFHHQIVDSPISIPEPVYQADEWAKRGKNIWKAYTDRHTLLLKDDKGEYADFPIDFEAMTKRLAYWHTNLQMYRVNA

***Pristionchus pacificus* C04F12.1**

MTGDRGGPGRGGRGGGGGDRGRGGGGGGGYGGRGGGGGYRGGGGGGERGRGGYLGGGGEYRGGGGVSQVSAGFQSMNLGEEPEPLPFRTPSTNEQQGYKAAAKPAPALREGKRKVQVISNVWQLEYEDRLVYRYDVSVGLEAIGNDGSSRIFSVNKGPKDDASVHERNELIREALKRGLEMYNILSRNGCIGVDGAAMMYANEDLGGALKGCQYKMKVPVEGLPEEVRKFIRNAGVKNVVIEVVAEKNFRIKDLSGQTSIDRSDMDQSVKQFFEVITSEHALSTHNYSFFSGGNLYRDDPDRIKMGAGNKFARPIGSGQERRDGMSKGFTLAEKNGSIIGALNIDVTTGTFWSEQKLLDTILEMNGWNHVRDARWDARAIKRTNDLIKNLRVSYTPPGADQEGNSSSSFDFIANGITASTWSTDKGETRQVVRTIEQLKCKNAAGQEEPLYNKFSNANLQPELRSWPLIESKRARRRTDDPTKYDVNTEFFPIELLDVKANQRVPLKKQGADPERAMSVEDRWKGTNQALEALNLFGDTRQNKRNTVMDAFGIRVSRRPLETIGITRRMPNVTFNRDADVDFKTTFRTGQLQFQTPAHITTLVIAVSDNQHSFDRNRFRNDLVNAATKKKMIIDNAIVEICHPNKMHDRLKKISDDRKAGDPKLMKVVFMYIEPEASKRHDELKLFERRHCVVTQHLTMEIASKLGTNRPTMENVLLKLNVKGGGHNYNVNPEKFAQQLWMGEKTMIMGYDVWHPTGQSRAEKMTGSLPEPSVVGFSFNGGIKADSFIGDYHFQEDCKERVEDKVLNARIKWMLEVFEKNRGVLPPLIIVTRDGISEGQYKYGMDELEALREGALEYAVSKARTDATGKAPFHDYKPKFIFVIATKRHHKRMYTEDRRGKGNMPAMTAVDDTITNPSLFEFFLQSHTPLQGMAKATKYTVLKDDVGTNQDAMQSLMGALCFEHQVSTNAISIPEPIYQSDEWAKRGAANMRKFRDIYGEQGQFKKGTTYDDLTTRLAYWKSDLAQTQNYL

**F58G1.1**

***Ancylostoma caninum* F58G1.1**

QSHKIIKGTGKLPAYSMPINEANLTMDEAQSLMMALCFTHQIVNQAVSIPEPIYQADEWAKRGRNNFKAMLRRVGGKEHLPILDGGNVDWNRVTRRLCYMDTELELTRANA

***Ascaris suum* F58G1.1**

MKGTARPIRCTVLVDDKPRMTLDEVEGITNALCYAHGIVTSPISLPAHLCAARDLAKRGRNNWKAANSADDDVSMSSGDGERGKFGNDGTPEFFPNLSIELAPKLKHKFWA

***Brugia malayi* F58G1.1**

MQMEQASVSRLPVRLADKIAPGIRKSELLDVVTNVWGLTSHENIPIYRYDFRVLEEYPPKKDSKEPSFKEVTKQVRNDYVAVDRRAKCFAVYQALLRREKQFFGAADTLIYDRASILYSLRKLSFPRASGDEVRKTFFLIKNELPANIVSDDCVKVHVNIKPCKEDFQLAMHDLKNCVSNNPDEINSSLQQFLEILAMQEVFFMQGRFVSYGTGECYLMDPSQFGFGDRDMPELQEGKYVAIGAAKGVRIIEGLGNFKRGINAGLVLDAKKAAFHIDNQHLLEKVESIFRRTRAEIGHGIDQQSILILSKALKGLYVRCSYGKNREFAIAGISKENAHTSKLALKTGEMSVEKYFMTKYSIKLKYPKLPLIMERCQPKNNLYPIEVLVVCENQRVSKGQQTSYQVQTMIRVSVK

***Caenorhabditis brenneri* F58G1.1**

MSGNSAQAPIQVPPPVPPEFGLLAAFGNKSANDACKERLEQLQLNPGPKIYPKTMDPGAHGTEVKIQTNIFGLEVFKNSDVFQYSVSIKADLTPTKEAVFTKKGKEDYVVLDRHQKCVAILFHAMETHQDFFQTQDNCYVYDGQSLLFSTIDIFRQEGAAKTKVFEIDGSAIDHEDLQKLACIKLEVFPTKNARVKFTQDDIGRRTSDSNIEAVHGAYHQILELAFNQSSLRDSARCVVFEHGKIFFITPANEGYSQEDYIDVGDGKKLIPGIKKTVQFIEGHQGRGANNPSVIIDGMKVAFHKEQTVHEKLEEIMSNRIASGLKDFDRERCAAVIKGLDCYTKYTDRVRHLRIEGIHHECAENSRFQLKNEETSTVAAYYRERYQMVLKYPKANLIVCKDKGNLNFYPMEVLTISPNQRVKISQQTSAQSQKTTKESAVLPDVRQRLIVIGKTAADIRSENEVLRRLGVLVSDEPLLVSARQFPPVKLAVQPTGPMLSMRDNKWRISQYARPATAPNIWALYCVGSQTTRFSHDMLKKFGEEFVGMFRTKGVILSPPADIGLVLFSEIMGKLDDAAKSNCSYVLIITDDSITNLHQKYKMVESSTGMIVQDMKISKALSVLQGKKLTLENVVNKTNVKLGGINYIFTDTKKFMNDHLIIGIGVSHPPAGTKYLLEGKGVLNPTIIGYSYNANKSQEFSGDFVLGPAGQDTIAVIEDIVTKALLGYQKWHDGNNPKRVLVYRSGTGEGSHGNVIAYEVPLARFAINQFSPNIKLVYINVSKDHTCRLFRKDLHEIAPRGNGSTSGSRNVPPPPKSCDLNIGPGICVDSQVTHPGCKQFYLNSHITLQGTAKTPLYSVLADDVNVSMAGLEEITYNLCHLHQIVGLPTSLPTPLYVANEYAKRGRDLWNEHCEKNPVIRQTGSERERLQEISEAINYQTKHGLIDRRLNA

***Caenorhabditis briggsae* F58G1.1**

MPASPLPMPPITAPPGPMPPPPIPPAPGPVLPITSAHKTANDAYNGVSKDLSFHPTAWHIRKGDRGSIERVWHRCRERDRTFPIQCLIRADVALNKEVVFTKKGKEDFVVLDRHEKCCTVLYYASETHDDFFQSEMNCLIYDGQSMLYSSIDLFSNRLNTENKSRTFQIDGVEIGHKDFQKLPCIKLEISATKNPSVKFNRKNVGKRSADQNMLAVNTAYRNILELALNQSCVQDYHRCAVFEHGKIFFIRPTEEGFLSRDCVDVGDGKRMMPGIKKTVQFVEGPCGRGECNPAVVIDGMKVAFHKEQPIIEKLQEVVTSDFSKGLKEGERERCVGVIKGLDCYSNYIGRLRHLKIEGIHHEGASKARFQLKDGNSSTVAEYFQSRWNITLKYPDVNLICCKERGMSISTQWSSYTSLQTSVAVPPDVRQRIIMTGKAAAKISGDAVLNKLGVQICEAPLLVRARQFPPVKIALNPTSSAIPQKENKWRIGQYSRPAVAPKIWAMYAVGTPSSRFSQPQLANFAREFVKVAQQKGIQLDPPKDCQCVLATEIESQLAAAHKASCEFVFIITDDAITNLHQKYKMIESLQSIVIQDMKMTKALSVIDSGKRLTLENVINKTNVKLGGSNYIFTDTKKYLDRVLVIGIGISQPPPVTKFIVEGKGFLSPQIISFSYNGKQKQEFAGDFVLCPAGQDVNGFSASKLIISLQTLSSIEDIMKESILGYKKCHDGTDPETVIVYRSGVSEGNHGNVIAYEIPLARAGINEVSKSVKLVYIAVSKDHTYRFFRNDLIIRKKSFRSSQSQLKPCDINIPPGISIDHMVTNPAIKQFFLNSHIALQGSAKTPLYSILADDTLASLESLEELTYNLCHLHQIVGLPTSIPTPLYVAAEYAKRGRNLWNEANLKNPLIRSGSEREQLRAATHSINYKHTGDFSDRRVNA

***Caenorhabditis remanei* F58G1.1**

MLPLRKTTDQYGVLPPPAVPPPNATITSDSKTANEACIARLQQLQLQPFPKIYPTTKPPGNRGSSVDIQTNVFGIEIQKRSDIYQYSVSIKTDLTSSKEVVFTKKGKEDFVVRERHGKCCSILMHAFDRYSEFFCTNENTLIYDGQSMMYSTFDLFQESSDGETKTKLLPINGNDTDHEDLKSLPYIKLEVHATKNPPVKFSQEDIGRRSSDSRIDSIHGAYHHILELALNQSCIRDFTRCMVFENGKIFFVNPLEEGFSRDDFVDVGDGKQMLPGIKKTVQFIEGPYGRGQSNPSVVIDGMKVAFHKEQPLNEKIREIISKNVTDCISDFERERCVAVIKGLDCYTTHAKRVRHLTIEGIHHEGARKSRFQLKDGGASTVAEYFRDHYKIELRYPNANLVVCKERGNLNFYPMELVFISPNQRVKISQQTSAQSQKTTKESAVLPDVRQRIIMTGKIAAKISSDSKYLNEFGLSVCDEPLMVTGRILPPVKLEGRTSANMLPIKDNKWRLGQYARPAQAPKVWAMYAVGLPSSRFTPALLSKFGDEFSAMCRSKGIEMPALGDIDLVLAQDIEKKLSIAADAGCTFVYIITDDAITNLHQKYKAMESLHSMVIQDMKMSKAHSVVSQGKKLTLENIVNKTNMKLGGNNYIFTDSKKYLDDILVIGVGISQPPPGNKFISEGKGLLNPMVIGFAHNGKQKQEFSGDFVLSPAGQDTLVVVEEVLKQSISGYQKWHDGQCPKRIIMYRSGVSEGSQGNVIAYELPLIRSTIDSFSKKIQFVYIAVSKDHSYRFFKSNLNTLTKSNTAISKGQQSSVTGSRSAMPAAGAPKAWDLNIAPGIIVDSVITNPACSQFFLNSHITLQGSAKTPLYTVLSDDTHASMASLEELTYNLCHLHQIVGLPTSLPTPLYVANEYAKRGRNLWNEAYSRNPVPRGTGSESELLQELSNAINYKAFGSFLDRRVNA

***Haemonchus contortus* F58G1.1 (Likely exon-intron boundary issue with predicted protein)**

GSARTPRYTVLVDDLNLSMDELEGMTYVLTYDHQIVNLPTSLPTPLYVANRYAERGRNTYLEPEAPIMAKNAPPGVLGSRYVVQTNAFGVQLEKPMAFWRYDVVISAEIGSAKRPVFFTKKGRDEYVVSQSVLNC*NFRYPHAPLVKVRERGRTNNYPMELGFLRPMQRVTIPQQTPDQSQKATRHAVFENGKVFMSHPWNFGFHQQDCPSVGGGKRLFPGTQKSVRFIEGPGGRNYNNPALIIDGNIPPGVVVDEGLTHPSFKEFYLNSHITLQYIIMNRNYKCKLVFDAVVRLYSDFFDDPDQLWYDGQSILFSGKDLFKDELKARLKEAAKHNCKYCLIVSADSITTAHSLFTVNTMSDIIRNVLPKFAQNHGNKFPRDLIIYRSGLSEGSFSTVVVHDRRMVTMENILLKANLKLGGLNYEIDMNGQMPSILTHEIPLLRGALAALGAKNVKIVFIVAQKEHNVRLRSLRSLIPS*SCAVPPGERQDNIARGARALKLLGSDDNPFVSNAGLYIYKDPIKVITVLAKKAAFHEELPLIEKAKHILNDNLLERVTEIGLERLNAGMKGRFEDHEGRRISVRDYFEEKYKIRLLFLGIGVSHPPPSQNFDERSVPSVVGGEKALHRSERFVAGFRSSEVETGHWKTKNELVDHR*LVTMFCNSNFGGCPFGLIDILCSLDKSLVALSVIAIPPKSVRF

***Meloidogyne hapla* F58G1.1**

EKCIDKGMSFGNPRLFPKAQTLFDSQTAMPIPTEWRNCFSHCTKEKIQFVLMIDSKFNDSHGLLKLFEVYRRSKVAKLITQQITIETVENILVKGHRQTLENIVNKLNVKFGGLNYQPLLAEPQLTGLKKFEQRFDLSSGNILVIGYDVSHPTYKIHEPKKPRLNETDPE

***Meloidogyne incognita* F58G1.1**

MDLASYVASLKVSANIPPPATQFLSTQNGYANIYGFIVKPDAVVYRYDVELSDKVKDKSLTKGGGDDGKRGLLRDICFELVKDVFDSTQNLNCNGTTCEIKPERMTPFCRKFLRNATITFELQPCKGSSHELNLNDIPSALCPAPHKQADHSLRTFFEMLTSQSFINARTHRLVGNGRLFEEKEVKRLNDAIVAHNGVAKGVRIIANGGDKPVPAIVAEVKTCAFFAKGNLGDIARGMVQSAMNRRRNPLRPNDRKMMESFWLDFGHLFKGVSAYLTYAPSRVIVIDSITTRLVSEISFEVDGRNIPMMQYFAEKKNVRIEGNMPAVRQHVDRDAIYPLQCLEILPFQRVSIDKMQLTDEMAQISSDLLKANAVGPDVRSELIKEQMRQIGKDGDCAKFMFRFGVKLRGDQNNVQIGLRKLPLIQFGNNITANPNDNERGGFDVRGPLRYLEPSDVLRSWIVAFPRKITQDSLNKFIGDIQRMANQRGMKLPQPVFENLEVNAMEQRFAEFSKAKIQFVLYIDERFVKSHAKLKFCERRYTVLTQHVNIEMVSRAGPASVSNVLAKMNMKLFGLNFMPIFDPVTKNKLDLSSGQVLVLGIDTSRPPRATAFEKFKLSKQGMEGFTSEDPLTIGPCKQDVLDVDYFGKRIKAIVGSLKSSRKQLPSTIFIIRDGISEGMVPNAVSGEFARIIETFKGIEQGWVPKFVFVIVDKRHTKRFFFNNGPIRNCKPGSVIDKKCVRVDLHEFFLQAHHPLKGTAKIPQYMIPINEIKANNDELQAFILGLCNMWQIVNMPPALPSPVLQAKELAKRGSNNYMEMKRNAPQYIPRVAGERIIDFSMLNDRVPYGTTGLAKTRFNA

***Oesophagostomum dentatum* F58G1.1**

KGLLEVLNLAMSQKGYLETSQFVTYGSGVHYLFDHRALGFRDQEVPELMDGKYMGIGLTKAVKVLEGDKGQSCGAFVVTDVTKGAFHMDDQNLLEKISQMSMFIDPRSGQSHFNVQSAMQPFNQKAILRLIKGLYVRTTYGKKRTFPIGNIAQPASQLKFQTVDGKQCTVEQYFKQHYNIQLKYPAMFTVSERH

**ALG-4**

***Ancylostoma caninum* ALG-4**

NMILKMNMKLGGINSRIVADSITQKYLIDVPTIVIGIDVTHPTQHEERHIPSVAAIVANLDLYPQSYGANIKIQRKCRESVVYLLDAXRERLVSFYKTTHQKPIRIIVYRDGVSEGQFSEVLREEMQGIRTACLMLSPDYRPPITYIVVQKRHHARMFCKYIRDAVGKAKNIPPGTVIDTGIVSPESFDFYLCSHFGIQGATSRPARYHVLWDDSKFTSDELQAITFNMCHTYGRCTRSVSIPAPVYYADLVATRARCHLKRKMGIYESDVTSDAASLISSSLSSLISAGCITNLRHIIDNDNDISDGESTKDDLVQSITSNSDATLQEYVTVSEKFKGRMYFV

***Ascaris suum* ALG-4**

MVDAMRERLLSFIRRTSLRPAHIIIFRDGVSNSEFVDTMNDELTSLKAAMNRLASDYAPTISYVVIQKRHRTQCDEFARLRLPSYFLTGVFSAFNRGKHNVPPGTVVDEEITSPNMFDFYLCSHLGAIGTSRPAHYTVLYDSWNLSPDDWQGTP

***Caenorhabditis briggsae* ALG-4**

MSRRNATSFVDNTLTSSEYAPVTGISGSGSMSPPITSRPASGQASPLSTNGSLSPPAYADENGSVVYNADSPRDLSPLLLSELACLNMREVVARPGLGTIGRQIPVKSNFFAVDLKNPKMVVIQYHVEVHHPGCRKLDKDEMRIIFWKAVSDHPQIFHNKYALAYDGAHQLYTVSRLEFSEATVRLDCEASLPKDNRDRTRCAISIQNVGPVLLEMQRTRTNNLDGRVLTPIQILDIICRQSLTCPLLKNSANFYTWKSSCYRIPTAAGQALDLEGGKEMWTGFFSSAHIASNFRPLLNIDVAHTAFYKTRITVLQFMCDVLNERTSKPNRNNQRGPGGPGGPGGYRGGRGGARGGGYNNFGNRGGPPTNPRDDFGGNGLTFTMDTLSRDTQLSSFESRIFGDSIRGMKIRATHRPNAIRVYKVNSLQLPADKLMFQGVDEHGTNVVCSVADYFSEKYGPLKFPKLPCLHVGPPTRNIFLPMEHCLIDSPQKYNKKMTEKQTSAIIKVTKNLFLEKRLSYLNNVNFRAAAVDATQREERIKQLAAQASFSSDPFLREFGVAVSSQMIETTARVIQPPPIMFGGNNRSVNPVVFPKDGSWSMDHQTLYMPATCRSYSMIALVDPRDQSNLQTFCQSLTMKATAMGMNFPRWPDLVKYGRTKEDVCTLFTEIADEYRVTSTVCDCIIVVLQAKNSDIYMTVKEQSDIVHGIMSQCVLMKNVSRPTPATCANIVLKLNMKMGGINSRIVADQITNKYLVDQPTMVVGIDVTHPTQAEMRMNMPSVAAIVANVDLLPQSYGANVKVQKKCRESVVYLLDAIRERIITFYRHTKQKPARIIVYRDGVSEGQFSEVLREEIQSIRTACLAIAEDFRPPITYIVVQKRHHARIFCKFPNDMVGKAKNVPPGTTVDTGIVSPEGFDFYLCSHYGVQGTSRPARYHVLLDECKFTADEIQNITYGMCHTYGRCTRSVSIPTPVYYADLVATRARCHVKRKLGLADNVDCDTNSMSSSLASLLNVRSGSGKGKKSHGSTVDDETFSIPDGFPIKFSRIASLLLETSRVVCTSSEDPQNYPNRVCVA

***Haemonchus contortus* ALG-4 (Likely exon-intron boundary issue with predicted protein)**

RILSGTLPKTTQMGMVFPKWPDLVKYGRGRDDVVVLFNEIANECKQTSISCDLVMVVLPGKNSDIYMTVKESSDMIHGIMSQCVLMKNVQRPSPATCPNIIL*VSIKLGGINSRIVADNITHKYIIDQPTLVVGTSYIQHPQVYNRSANIGRWHVIHPTQAEERKNIPSVAAIVANIDLLLQSYGANVKVQQKCRESVVYLLDAIRERLVSFCGNTNQKPTRLSLIVMDVIFH*RQFGFSCD*S*SVVRCNVCACGHTSVASVEKHHKLYSIVDLRDLEGGKEMWTRFFSSAHVASGWKPLMNIDVAHTAFYKAKISMVQFMCDVLKDALVRTVIRRPLRVYRVNSLQMPADQLTSRGVTEDGRETIKSATQHFVEEYVELRFSKLPCLHVGPPTRNIFFPLEVCEMDTPQKYNKKKKLSEKQKSSIIRAAAVDASQREERTDQLCQQAGFDSDPFLEEWSFGLTVFRSNLGCLRQWLGLSNRHRSCSVAIRKKGSKKKSWKGGWTSLTLK*RRLGSVRARMHEGDTMSAIQTTHPNRSGPYLGGHYCSVILWLLCPL*TE*PTVPPCLAKTLYLRRSGMRSR*KCYRQVASTKNFRCPIMR*KS*LKL*RASKSGSLITSAFSSTE*CPNLFRITHPI*NLKLTELGGQYPRI*RHC*SSPMFGGIPRATFFITIFASSY

***Meloidogyne incognita* ALG-4**

NPSKFVGDYLYQPCKQDVLDIDYFGEGIKWIVSTLKANRKQLPSTIFIIRDGISEGMVPKAVNGEFSRIIETFRSINQGWVPKFVFVIVDKRHTKRFFLNTGPNRNPKPGSVIDKKFVRVDLHEFFLQAHHPLKGTSKIPQYMIPINEIKANNDELQAFIIGLYNMWQIVNMPPALPSPVL

***Oesophagostomum dentatum* ALG-4**

DQPTLVVGIDVTHPTQAEERMNIPSVAAIVANIDLLPQSYGANVKVQRKCRESVVYLLDAIRERLVSFYRNTNQKPTRIIVYRDGVSEGQFAEVLREEIQGIRSACMILSPDYRPPITYVVVQKRHHARMFCKYSTDMVGKARNIPPGTTVDTGIVSPEGFDFYLCSHYGVQGTSRPARYHVLWDDNNFSADEMQAITYGMCHTYGRCARSVSIPAPVYYADLVATRARCHIKRKLGVHESDGLSETGSVISSLSSLMSVGRPRRRREFEAGDINANQSNSDAALQECVSVTDKFKSRMYF

***Pristionchus pacificus* ALG-4**

MAGMRGPPGGRGMPPNRGPGGYGGAGMRQPSPPGGYGHGGGGGAGGALSIETLYPEFTLSPHELAVLSEAVKGVRVRIAHRPKVVRVYRVNSLQLPADQLSFPTTSEEGEEKMMTVAEYFEQKYTKLKWPKLPCVHVGPPNRSIYYPIEDPVVYPRDGAWVLDNQVLYLPATCRSYAMIALVSQREQMALQSFCGALHGKALQMGMEMPRWPDVVKYGKGRDDVPQLFHDTLSEYEQIGNQCDLIIVVLPAKNSEIYMTVKECSDMVHGVMSQCVLLKNVLRPSPATCANLILKLNMKLGGINSRLVADGITQKYLIDVPTIVIGVDVTHPTQAEERMNMPSVAAIVANVDLFPQGYGANVKVQRKSRESVVYMCDAVRERLISFFRNTHQKPSRIIVYRDGVSEGQFAEVLREEMQSIRSACLTLSPDYRPPITYIVVQKRHHARLFAKNPRDTVGKAKNIPPGTTVDTGIVSPEGFDFYLCSHFGIQGTSRPSRYHVLWDENNFTSDEIQVLTYSMCHTYVRCARSVSIPAPVYYADLVATRARCHIKRKLGMHDMDGMSDSSATSSMSSLVSLRRRGGSDGGTAAGARSTNRSGGSSAETVSESMAENIASACDAALQDYVTVKEVFKSRMYFV

***Trichinella spiralis* ALG-4**

SSEICGDITFGLVTQCVLPKTISDVAIKKSYSTMLNIAMKINMKIGGINTKLLEDEILDNYLYKNNALVIGVDVVHPSAVETHLPSIASVGIIHVVGNVDTKVTKFHASVKLQPAKQELITGFIEQFSERLLEYLDVNGTAPKNIIVYRDGVSEGQFMQVLEEELPALRRACKSFATNYRPLITFIVVQKRHHARFFCCDEAAARGRGKNIPAGT

**RDE-1**

***Ancylostoma caninum* RDE-1**

MLSTAFLEMKWIRDAWKRLTADDAELEPTYTYIVIQKRHLTRFYQPSKDEQGKETYVNISSGTVVDNVVVSPKLFDFYLASQFGAIGTTRPAHYTVVLDEWMLSADQIYEMCYKLCFLYARCRIPVSLPCPVYYAHIVCEKAKEVYKTLCSNHEFDGIEDVDLRKNRIEDSLTAHKDYPGMHFV

***Ascaris suum* RDE-1**

MKDPFNVLMRACELFGMEFARSYRNPAEEVFKDGWDTDDGDVSSLMPIINAFKKNVALTDVEDVRPLLIFVVPKEDSRIYESSSRNLEELKNIKYRRAKNVFPWGGGGGYAPSYLYVVLYRNRHFSVAAGIKVACDREAGIASQVISTKTFRRMAGRPENNAVAHNIFLKINAKLGGVNNRVLQSCFLPPSLRLMSMLRVVKWKLQRNILVWQKFTDHEKPTLFIGIDVTHPSSGDTTX

***Caenorhabditis brenneri*  RDE-1**

MVNQATSLSLDLKWFPRPNKKCDGNFYEKKVLLLVNWFKFNEKIYDREYYEYHVDMQQQITKKGKDGKVRHKVSEVPIPERAVLFWLHLRHEARGNPNLKIEDYVFDGKDTVYSVHKHNQPITTFLVDPVQKNTKYAMTLSLREGFRLNFSREDPSKDEEANRSYKFLKVVLTQKVRYTPEVNEKVRIEFAKNFVYERNSILRVPESFHDPNRFDYSLEIAPRIESWLGIYIGIKELYDGEPVANFGIVEKLFYNAPKMSLLDYILLIVDSETREDEVRNSKKRQLKNENLTIPLSAAKKIDHYLQGLKLKCTEVWEESLGRMAERHLIFNELSSVNSFEAMVKIKARRDVSSREIPIFEIYKNNRKHIEFPYLPLVKVKSGKKEYHVPIEHLEVHEKPQQYRSWIDKVMQDKVIKAATRKPNEYKTQTIEMLKELELTSEDLNFVDNFGLSTELKMITCVGKVLKEPKLVNKENKSIPMTPVIRGFQEKHLNIVPEKELCFALFILRNEEAKHPCLSENEAFDFYEALVDGCDFRGMRIGEHENRRARSILQDNSGSVKYGFYKDVTLSNGACSFQAAANDAKAMFDRLTDRDRKALVFIVVSDLYRDAYGFIKHYCDVNLGVVSQYVTAAVAKRKTRSICYNLALKINAKLGGVNQELNFSENAEMSLAEKEERKQMPLKMYVGIDVTHPTTGSGIDFSIAAVVASINPSGTMYRNMIVTQEECTPGERPVAHGRERTDILEGKFVHLLRTFAENNNNRIPEHIIVYRDGVSDSEMLRVSHDELKSLKNEVDRFIGERGRSETVPKYTFIVIQKRHKTRLFRKIDEKRPINEEEAKQWDEDMKESDKSGIVNPSSGTTVDKTIVSKYKFDFFLASHHGALGTSRPGHYTVMYDDSNLTQDQVYKMSYELAFLSARCRKPISLPAPVHYAHLSCEKAKEIYKSFKVYKEQLPHKPSRHDIENYLQTNNNYPGMSFA-

***Caenorhabditis briggsae*  RDE-1**

MSSNFPELEKGFYRHSLDPEMKWLARPTGKCDGKFYEKKVLLLVNWFKFSSKIYDREYYEYEVKMTKEVLNRKPGKPFPKKTEIPIPDRAKLFWQHLRHEKKQTDFILEDYVFDEKDTVYSVCRLNTVTSKMLVSEKVVKKDSEKKDEKDLEKKILYTMILTYRKKFHLNFSRENPEKDEEANRSYKFLKNVMTQKVRYAPFVNEEIKVQFAKNFVYDNNSILRVPESFHDPNRFEQSLEVAPRIEAWFGIYIGIKELFDGEPVLNFAIVDKLFYNAPKMSLLDYLLLIVDPQSCNDDVRKDLKTKLMAGKMTIRQAARPRIRQLLENLKLKCAEVWDNEMSRLTERHLTFLDLCEENSLVYKVTGKSDRGRNAKKYDTTLFKIYEENKKFIEFPHLPLVKVKSGAKEYAVPMEHLEVHEKPQRYKNRIDLVMQDKFLKRATRKPHDYKENTLKMLKELDFSSEELNFVERFGLCSKLQMIECPGKVLKEPMLVNSVNEQIKMTPVIRGFQEKQLNVVPEKELCCAVFVVNETAGNPCLEENDVVKFYTELIGGCKFRGIRIGANENRGAQSIMYDATKNEYAFYKNCTLNTGIGRFEIAATEAKNMFERLPDKEQKVLMFIIISKRQLNAYGFVKHYCDHTIGVANQHITSETVTKALASLRHEKGSKRIFYQIALKINAKLGGINQELDWSEIAEISPEEKERRKTMPLTMYVGIDVTHPTSYSGIDYSIAAVVASINPGGTIYRNMIVTQEECRPGERAVAHGRERTDILEAKFVKLLREFAENNDNRAPAHIVVYRDGVSDSEMLRVSHDELRSLKSEVKQFMSERDGEDPEPKYTFIVIQKRHNTRLLRRMEKDKPVVNKDLTPAETDVAVAAVKQWEEDMKESKETGIVNPSSGTTVDKLIVSKYKFDFFLASHHGVLGTSRPGHYTVMYDDKGMSQDEVYKMTYGLAFLSARCRKPISLPVPVHYAHLSCEKAKELYRTYKEHYIGDYAQPRTRHEMEHFLQTNVKYPGMSFA

***Caenorhabditis japonica* RDE-1**

MRWFPRPGEKCSGDFYVEKVKLLVNWFKFSSKISDRSYYEYGVEMKMEKKFKLKNGQWKMKSTLIPIPDRPHLFWQHIRHEKRENERNGRPFREEEYVFDNRETAYSVNRHDTKITSRMVSSTNQNVTHVLELVPRREFQLYFSREDPMRDEEANRSYKFLKDVMTQKVRYSPEHNNQIAIEFTKNFVYDSNVIHAVPESFYDPDRFDYSLEIAPRIETWFGLYIAVKETSEGNPMLNMAIVDKLFVNAPSMSLLDYLLLVLDPETRNDEVRNQRKTELRRHKLTIDGSKRGQLNRYLCNIKMKCTEVWDESKMKMCERHLSYIELSNVDANQHEIKVQVGRGRDATTRKVPLSRIYEDNNKPIEFPYLPLVVVKSGSSQFSVPMEHLSIHEKPQRYKSWIDYAMKDRFIQRATRKPHVYKEKTFEMLESLGLSSEELNFVASFGFSPELKMLPCPGKVLKEPMLVNKENKKIEMTPVIRGFREKQLNVVSEKEMCCALFVLRSADDRGPCLTEKQATDFYGEIIDGCDFRGLNIGKSDKKAERSLLRGEEGGVTKFGFYGNCTLHGSVCTFQDAAQHAKRLFDNLPDKNRKSLLFIIITERESKQYGFIKFICDNDLGVPSQHVAKDTVVRVLRDLDRSHRIAYQIALKINAKLNGVNQELDWSEGSEVSPEEKERRKSMPLKMFVGIDVTHPTSNSGIDYSIASIVASINPGGTRYRTVVATQEECKPGQRPVAHGRERTDILEGKFTKLVQSFAENNQNRMPDHIVVYRDGVSDSEMLRVSHDELRSLKMEVSRCLRERGTPDSPHPKYTFIVVQKRHNTRIFRQIDEKRPSDPEASARWDADMKESENTGIVNPNSGTTVDRVIVSKYKFDFFLSSHHGALGTSRPVHYIVMYDNFGLTKDQTYKMSYELAFLSARCRKPISIPAPVHYAHLSCEKAKEVYRAYKEHLIGQHPVANRQTIERILQPNPDYPGMPFV

***Caenorhabditis remanei* RDE-1**

MTNSELKFYTHHLDPNMKWFPRPGEKCSGDYYVDKVMLLVNWFKFTKKIYDREYYEYIVEMKKEKRVKNKKTGQTTIKSSEIPILDRPKLFWQHLRHEQSKNPFDIEDYVFDDKDTVYSIHKNETGMNSVMPDPENPNITYILRIQYMGPFRLHFSREDPAKDDLANRSYKFLKAVMTQKVRCAPYMGNEIAIEFAKNFVYEGNSILRIPESFHDPSKFDYSLEIAPRIEAWFGIYIAVKELFDGEPVLNFAIIDKLFYNAPKMSLLDYILLIVDPETQNDERRNRRKQQLRNEKLRVSPFQARQIEKWVENLKLKCCEVWDERLNRMTERHLTFLRLSEYNAVEQTIPIPRGRARDAPIDNVPLSRIYEKNRKEIHFPLLPLAIVKSGNREYSVPLEHLEIHEKPSRYKNMIDHAMKYKFLKQTTRKPHIYKQETIKMLEDLGFSDGELNFVERFELCSELKMISLMGKVLKEPNLVNKENKKISMTPVIRGFQEKQLNVVPEKELCCALFVLRAEDEKEPCVSEEDASLFYKTLIDGCEFRSIRIGTHDNSDARSLLYDPEAKRYGFYKEVPLQYGAANFHAAANDAKSMFDRLLDKDQKILLFIVISERSLNAYGYIKEFCDVTLGVASQHITAETVLKALHQMRPESGVKSKRIFYQIALKINGKLGGVNQELDWSENGEMSVEEKEERRKAPLRMYVGIDVTHPTAGSGIDFSIAGIVASINPGGTVYRNMIVTQEECRPGERPMAHGRERTDILEGKFVQLLRIFAEFRDCDLIRTISMIEDEPMLWDMTDADYKNGEKKDSTWHRLESEVGFLKVNRGYTIKKIWVQMVRDYRASKNKNKAVSGSGLEEMTVNEFPFEKEMSFLGNISSNSIPEPCEESTPSVTKRYSSYQRENMMETPKRRRSSEDVKEETDIDKLIKICTMKLLEPEKPTSPHEENSELLVLINDTMKFLTPSQQLDLKLDIGNLCRNAKLQNSKKSASYSSMAEPSRSGFDDYDFDWQKYN

***Haemonchus contortus* RDE-1**

MYVGIDVTHPTNLSTIDISIASIVANVDLAATRTIYYNIALKINAKLGGVNQAVVFDDESQFSGLIKSVCDNEHGIASQVVDASTVRKA

***Pristionchus pacificus* RDE-1**

MASGIGANPPPVVANAVERSRVYRTPVTAPPFPYKKVIIAKRPEKGIRGVPFDLKLYIYKVVVDKEIRKGKGKGVEPVTNPSNIAPCVYKVIHSYPQIFRNPWHFIFDDFEYLFSTELLEDRPGRELFTTDDRNNKVRVRISRFAKFELTKGGIDDIDAQWSAIFIKLLLSQNARFIPPQLKQDHELSIHARFAHFGGAMFYIPKSVNGINIQKSTVRKKMVQPGCEAWTGFYLAYRQWEDGNPVMNVGMVHKFFNSLNLNLLDFYEAIINKMGKATQKVTLTRNTVMSPAGIADFTERLSGLKVKCDIVPEKDRDGLLINGIVVRHYEFVRTMNTSECADNYYLTRFNRARRQKEDVRLDVWFENQGKPLKYPKLPLCEVKSGKRTDFLPMEVLFTHGKPTPYTKRLDTIAKMEIPTLLARSPQDHYRLTMEMTSKDLEYQKDPFMNAMGIKLETQMEECGSRVLYKTGIISKDDSGEKIDMNNETGEFPLSRAYQTTEKEIIFVACNVTNIINDDTAKSFCRKLVDKCKSRGMRVSTHNQYDKNALQFLEKRQRTDTEILVFLFFVGNMDEVLYGEIKVLSDLNHGVVTQVVTEHTVARMISTDPDRELYDYKSMFHHIWLKLNVKLGGVNQIVDFTGANEPDLPRLPSHERTMFIGIDAIHPSPNSPIRSFTLAAIVASLDRNATKYADRIMVNVNCNETVQHFEEHFALLLKEYHGERGHFPDRVVILRDGVSDSEMIKAASRELQSIKGAWKRCTDVKPPPFTYIVVQKRHRTRFYRSDVMENNANPFPGTVVTEGAVSPHKFDFYMISHYTVQGTSRPAHYTVVMDESEFSTDEITEMCFRLCCLYARCSKPVSIPAPVYYAHLRCKRAAVQFQFAARPENKHKTRQEEIEDKGRNEIERRYTERVAKHAQDIEQFLNRDFTKPKMHPGMAWL

**C16C10.3**

***Ancylostoma caninum* C16C10.3**

QALGGGTTQKDMNGRLFHGKMFVGFELSHAAAQSLYDRQNATAVKEPTVVGFSYSVGEPTDYCGFWWYQQPRLHRVQYITIHFERAFLNYHKRNKRLPTEVFVYRSGTSEGEFSEVAEEANEIRVVAEKMRDLNSGRPYRPKITVIVAQTNSNYRIMPASMPPPTGGRPRAQDFNVPSGTCADSVIVHPRYREFILTSQQANIGTSRPTRYTIVTEDKPQMSVDDAEHITHFLCHGHQQSTLPTHIPAVLYAAENLAKRGRAAWKSKINENSDGASSSASHRISLREGETAEDFYSRISQELMNSLPNHYFA

***Ascaris suum* C16C10.3**

MDVEVIKENVLFKFRCDDMMVTVADYFASKYDIRLKYPQLPLVIERRPSGESYYPMEKLIVCENQRVTQTQQSSAQVQAMIKVRQAQALRCLYDYSRDWARDNVL

***Caenorhabditis brenneri*  C16C10.3**

MSDPLDTIMGSMSNSGSRSSSSASGRIPKRDHHTNERDEPSSKRSGESIKSSSKRKKYDFSAPSFATPTSTTPVGRDSFANMGEMDVQMNMFSLNLSKMPSQLQRLHVDTLIKSTNGKEYNLNLGVVAVSGDVNSHNRRLSQHLLMRKFAEKKRSLFSNQSYHHLAYDCAATLYVPLGIYIGIEGDVEEVALTKDDFSSEDWAVVSKICRRKDDTFIIQVKPAGYVNTQGDAALEDSNRLELTRCVEIVTSQMLNSSDFYQFGNATFPLNVHPNSEPDGTSEIRPGFAKVARLVEGRGGRNEMFITIDTKLSPFYKDISVLKFVCCKYQEFRGMGSGGFGGGGRGGYGGRGGYGGGRSDSRDSRGGYGGYRSDSRDSRGSYGGRSDSRDSRGGRDYRDESRGRRDSYDSRDSRRGSSGDYDGTDYSAQDVTEIEKFVSNGGHKFVSTLDNALKGLFVMPTHLKNTASQVRITGVRENTANNTRFVKKDEKGEREISVYDYFYEEYNIKLKFPELPLVVSKRFKHECFYPMEVLRIIPGQRIKVQKMTATVQSAMTGRNASMPRQHVELVQNILHDSLKLQKNPFMEAFGIQLESTRPVQLKAKLLPPAQIRFKNQSYMPDMSRPQFRTQGKFVEPANIHRVALVSFDRALDMRLAEDFCDQLYNYCRDNGINVERPSKDWSIREMNSGDCVAIKEVMEGWMKKGVDILVGVAREKKPDVHDVLKYYEESVGMQTIQLCKQTVDKMMNAQGGRQTIDNVMRKFNLKCGGTNFHVEIPNNVRGKSVCSNTETLTKKLLEGVQFIGFEISHGAPRTLYDKSRGQMDGEPSIVGVSYSLTNSTQLGGFSYMQTQKEYKLQKLDEHFPMCVKMYGEHAKRLPSRIVIYRVGAGEGDFKRIKEEVEEIRKTFDKVQLGYNPQLVVIIAQRASHARVFPLRIEGHKAGEQNVPSGTCIDNVVTAFGYDEFILSSQSPLIGTVRPCKYTILVNDAGWSKNEIIHLTYFRAFGHQVSYQPPSVPDVLYAAENLAKRGRNNYKIHQRNVSCQAIERSIIAEHAEYINEDMQEELASRIVDYMSDAMNGMTIRKRNFWA

***Caenorhabditis briggsae*  C16C10.3**

MSDPLDKIMGNMGGDLKPASSTSSSSFTRIPKRDRTSPEKGEPSSKRSAPFTTPSSTTKIGRDSFAVMDTMEVQMNMFQLDITKMPPVIQRLHVDTLIITSKGKEINLNLGTVSTGGDVNSHNRRLAQHEIMRKFFEKKRSLFSSSSYHSLAYDTAACLYIPEGQYRGGEYEEVCLSQQDFAPEEWDIVSKISRRKDDSFIVRIKPAGAVYTQGDELFEPSNRYELTRIVEIVTSQKLNTTDFYQFGNATFPLRAHPNSEPDASSEIRSGFAKVARLTEGREGKHEMLMTVDTKLSPFYKPTSVIRCVLTKIQESRGGGGGGGRGGYGGGRGGYGGRNDSRDSRGSFGGRSDSRDPRGYGGGYDRRSDSRDSRGGRDSYDSRRGGGDSEDFSSHEIDEIARACMESDKFLYSLESALKGLFAEAIHLKGSACTIRITGIKQCTANNTKFTKKDDKGEREISVSDYFYEEYNIKLKYPNLPLIVSKKMKYECFFPMEVLRIIPGQRIKVNKMSATVQSAMTGRNATMPRQHVDLVQRILQDSLKLERNKYMDAFGIELKSTRPVQLKAKILPPAEIRFKNLTYMPEIKRPGFKNPGKFVEPAHIYRVAVVSFDNAIDMRQAEDFCDRLFDYCRDQGIKCDRESKDWSIRELSSSDNVGIKEAMENWMKKKVDIFVGIARDKKPDVHDIMKYYEESVGMQTIQLCKQTVDKMMNPQGGRQTIDNVMRKFNLKCGGTNFYVDIPSNIRGRSICSNTETLHKKLLEKVQFIGFEISHGAARTLYDRSRNQMDGEPSIVGVSYSLKQSTQLGGFSYMQTQREYKIQKLDDAFPKCVKAYAEDAKTLPTRIVIYRVGAGEGDFKRIKEEIQEIRGTFEKIEKGYNPALVFIVAQKGSHTRIFPSRIDGNKAFEQNVPSGTCIDNVVTSFGYDEFILSSQTPLIGTVRPCKYTIIANDPAWSKNELIHLTYFRAFGHQVSYQPPSVPDVLYAAENLAKRGKNNYRVHQRYVSMQAIERRIISENADFVNENMQEQLASAIIDEMSDAMNGMTIPKRNFWA

***Caenorhabditis japonica* C16C10.3**

MNTASALLNPFLKLKSAHSTCGDNYGSAKFFFAINRAERTVFTMKDENGEREISVADYFVEKYNYRLKYPRMPLVFSMRLKHANYYPVELLKIIPGQRIKMSKMTVTVQSAMTSRNSSMPRQHVELVQKILHESLRLERNPYMDAFGIELESTNLIQLKAKILPPAQIRFKDQAYLPRPRSVAFRTQAKFVNPARLKSIAIIIFDHAIDERQAGGFCEALHRYCRDQGISVMEDHRKWQIKEMRSCDNSEIKRKMERYRDEKVDILIGICQEKRPDVHDVLKYYEEACGMQTIQLCSQTVNKMMSQQGGRQTIDNVMRKFNLKCGGTNFHVDIPQECMGRSVCSNNETLRRKLFEHTQFIGFEISHGASRTLYNRAHGQMDGDPSVVGVSYSLTNSTELGGFTYLQTSREYKLQNLEKKFPECVNAYKEHAKRLPSKIVVYRVGMGEGDIKRLKEEIEEIRSTFDKIQPGYRPHLIVIVAQKNSHTRVFPARIEGHKAGEQNVPSGTCLDNGITSFGAEEFILVSQTPLIGTVRPCKYTILENDPKWTKNEISHLTYFRAFGHQVSYQPPSVPDVLYAAENLAKRGRNNWIVHKKMDNMQDVERKILKRYPELDTEEMRDELAAAIITDMSERMNGMTIMKRNFWA

***Caenorhabditis remanei* C16C10.3**

MSDPFDRIMGDMSSGQASRSTSSSRIPKRDHRHSPEKDAPSSKRSNPGFTTPSSTTRIGRDSFAVMDTLDVQMNMFVLNLSKMPQKIQRIHVDTLMLCSNGKEINLNLGVVAVGGEWVLMRVFHHLFINFSVNSHNRRLAQHLIMRKFHEKKRHLFSNQSYHVMAYDCAAALYVPAGVYTGSDEEEASFSKEDFSSEDWTLVSKVSRRKDDKFLVRLKPAGFVETQGVDALEASNRMELTRCVEIVTSQKLNNNEFYQFGNATFPLRDSPNSEPDGTSEIRSGFAKVARLVEGRKGTNEMLMTIDTKLSPFYKNTSVLKFVINTYAESRGVGGGGYGGGGRGGYGGGGRGGYGGGRNDSRDSRGSYGGRSDSRDSRGGYGRSESRDSYGGRDESRGRRDSYDSRRSGGDSSGPDYNAQEVAEVEKAVRDNKNLVKTFEQALKGLFVEAIHLSGSSKIIRVAGVSEASAESSYFTQKDDKGEISVAEYFYKEHNIKLKFPHMPMIIMKRFKHECFFPMEVLRILPGQRIKVHKMSATVQSAMTGRNASMPQQHVDIVQKILSHSLKLEKNLYMDAFGIELESTKPVQLKAKLLPPAQIKFKNAVYMPDMGRPAFRNPGSFIEPAHIRRVAIVSFDRAIDMRQAEDFCDRLYDYCRDNGIKVDRDSKDWSIREMNSGDNVAIKEAMEDWMKKGVSIFVGIARDKKPDVHDVLKYYEESVGMQTIQLCKQTVDKMMNPQGGRQTIENVMRKFNLKCGGTNFHVEVPNSIRGKCVCANTETMNKKLLEQVQFIGFEISHGAARTLYDRSRNQMDGEPSIVGVSYSLTNSTQLGGFSYMQTQREYKLQKLDEVFPNCVRAYKEHAKKLPSRIVIYRVGAGEGDFKRIKEEIEEIRSTFAKIDHGYSPQLVVLVAQRASHARVFPSRIQGHKAFEQNVPSGTCIDNVVTSFGYEEFILSSQTPLIVSLIYHLSSFIVFNFQGTVRPCKYTILANDPNWSKNELIHLTYFRAFGHQVSYQPPSVPDVLYAAENLAKRGKNNYKVHQRYVSLQAIERRIIADHPDFVNEEMREQLASAIVDEMSVAMNGMTIAKRNFWA

***Haemonchus contortus* C16C10.3**

FVGFDISHAGPQSFADRQMKVPQSEPTVVGVISTRPMRAVARRFHPPAINYGGSMKVQPNHLGDMQWKLGAREQFAEPAKLYGRWTFIIFDGCVRKSDAEYVWKSSFNFFRRPMDQNVPSGTVVDDALHPAYNEFLIVPQKALTLPSVCTSATNLSKRGRNNWKAEKWVFAQQGAFMVLGNLCLKLNLKLGGINHILKSRRRVCMSLFNTLVKKHAHLFPKSSKYSFIYDCGNSLFMKKPLPDERGDAVLKIDLLPEMIELQKAFVEVNHLYRHGMYSPSLTCLVVQTNSNYRIIPTSINKCFNFTYRHFLEVHQTGYMPSSMVM*LSMSHGLRRNGFNVNDCKWH*MSLLHL

***Pristionchus pacificus* C16C10.3**

MEVDSKPIARRHTDFDHVQLPEPQGAGSEPIGERINTFINAYVIDTTESVEFGFKHELVFTAITAKREFAVHTKGGKDDAIKLLRHRCLKKLFAIVFTENADHFTRGDENKSGSLAFDNGCALYSPYQIDPLDGTIPLTDLPEEFLELINGVTELRYCLTFVERIPLKDQNVAKRTAIQLIDVASWMKLDKTRHIQFDNKAYYTSTKCPDDRMDGGKVVKNGFAKSIRPIGAHLALQIDGKVSPFFKAQKLIDLCEELGGSRNIGRNRQMEQQIKNLCVQTTHLPHNRIFQIKGFAKYNANELKFEMKAESGEMRTTSVAQYFEEKYKMKLRCPDLPLIEERKGSKASFHPIEVLYVVEGQRVSNTKSTATMIQDLIKRAQKRPPELMKHIEEQAYKAFLDGSKHENLDAFKYRITNRQLTSAADTVFSPTVLTANGKASDDMKFPEKWKLGNMDRFVQPAKCTDDMFCIVFERCYSDRDAEDAIRNLFDAGRQRGMDIDSRNVKIVPMSSSFDELRSFMFSKVGRAGAVIGFTSSNTDCVHENLKLFEAETGIVTLHCTKRVIDQVLQGKPLACGNIMMKFNQKLGGTNFKIAPPQELAKYAPKLAEFSKTWFSKTRMFFGLFVSHAGPQSFADRSAGIPQSEPTVIGMSFTTTMPTKQDGWWFMQEPGENLILDMVEDIVKALKCFHKANGALPNDIVVYRNGKSEGEFKAISTEAAQFKKAFAHVADNYNPTLTIIVVCVGSNYRIVTEGQRGLDNVPPGTCLTAEGCNPFYKEFIMVSQRAIMGTARPIRYNVVTEIQGTVGKVLMVDELKLITNALAYTTGIVTAPISLPGPIDSAEKVANRGRNNYKATIMSDCDASTSSSGPPRELRHDGSTDFFKKLSHKMETKMLDRSSVLAPTP

**PPW-1**

***Ascaris suum* PPW-1**

MQYLSSLTIELSRLVHLPLCELLSKMQDERLDGKTGAEETQYIFEFLLNDYDFPSDAIKDVERKYEVVTQDVKMSTAMDVVRKKKWQTMENIVNKTNVKLGGLNHAIVATHPG

***Caenorhabditis brenneri* PPW-1**

MESNMQNLSLSGNAPPNKLGLLPLAAKKQRNTEKGTKVLVETNIRRLTIAPNQPIYKYAVEVKYFYSKADGSEVAVEMSKSVKKGTEHDNDKTRCQKVYNEAIKRYDALKTGGPFFYDRQASLYTLSKLKNENISFDVVEGISKRPNFKKAQFVLKKVDESFQSTSNDIKKTVHPCPANADKTLLEALNIVVSGPAYENKNVITIGSCVHYLIDTAGVDINFKNYQEGYLYSGVGASKAVKTLEGSDRKEPSLFMTTEMKTTLFHPDYYPLLEIMKSYKGFATNLKANSFAAQRIEKAFVGLDVVLDYGPHKGLQEDGVVMNIRKFGTTASETTFELGGKNTTVFNYFKTKYGITLKYPDLFTVEAKGKKGKIHIPVEVLLLCPSQTVTNDQMINNEQADMIKLSAAKPQVRKATTDCIVKKVGLASNNIYNFIKVEEPIKVEGMVLNKPKIVFAENKMAVLNDPRAKFPTDFNRAGKYYIAKELTNWEIVFVQGEDMPGLDKILVEEMKNNGMKAAMPATSTIVRGDLEPIFKKAKDAKRQLLFFVIKSRYNYHQQIKALEQQYDLLTQEVRAETAEKVPRQSQTRLNIVNKTNMKLGGLNYVIGSESFNKPGRLIVGFETSQRSGGNPDYPISVGFAANMLDHHQKFAGGYVFVKRDKDVFGPVIKDTLITILETTKKNRGMPNDILLYFNGVSEGQFALLNETFRQHVKDACISLSETYRPNFTIIASSKTHNERLYKSDKGQILNLEPGTVIDHTIVSPVYNEWYHASAAARQGTTKATKFTLIFTTQPAEPMWNLQQLTNDLCYDHQIVFHPVGLPVPLFIAGRYSQRGAMVLAANDGPKYKDGQVDLQETNKELGYGGKKLFETRFNA

***Caenorhabditis briggsae* PPW-1**

MYSRHQKNSNHQSTGSRDGQSQSLFQKQPRNRNGDSVKLFTNLREIIFKEGFKIFKYTVNVTYITMKDGGKTETFVEVSKSIRKDSQLEQDKIRSFKVVKEAFNLINQLREDRRPRFYDCQGCLYTFATFLKPSYSITLKDGISTSPVFLRASFQMTRSRESFEMTVEDIKNTVSKYPGNENKSLLYALKTIIEGMMNPVFVETSDFRDTAFLAPDGMLHLINGKTVGLGPMEFEEGILRSSAGIKTEVKALESTGTPKVFASHDLRNDLFHPEKAPLFDVLASFMGFRNDLEATSNLAKKISNAVSGLDFTLNYGDYPDLQEDEVIFKIDGFSKSANEIKQIVADKEMSLREVFKAKERMINGEQQDVDLYYGGKPHITKKYTDDMVISMFLESAAPGISFRKQLELRGIVLKKPKIVFRNGSAQTFLQPVSLKKWTVVFVRKEEVEDLVEMLLEQWNQHGMEIAYPNVDWMGQDNNLEVVFHKAQKREKQLLPFVTHSRYALRKDIEWMEHKYRIPSLAIDLKIARKMVEDKAVKLDLADQVNKKTQGVNYAIYSDVFRNGNDLVISISFSEPSPKYAKVRILILFSHMIFCFQYPFTVGFAANTSNHPLVFTSEFLYSNSDNMDSGAVLTEIFYKCLTGTRKNRGELPTRLIIYLDEVPEEEFWRIDHHYEPSCAEACSQMGAEYNPQTTMIVATQKHNERFYKIDDKCLIWNPEPGTIIDHTAVSPVFNEFYHIGPSTSYKSTVKPAKYTVVYASDPVDMEHLEQLTNDLCYWNQLKSELINVPAPLDMAQKEASKGIQFLIDNGGPIYMEDGEVDLAETNAKYGYGNMSEKL

***Caenorhabditis japonica* PPW-1**

MTTKENPEISEEVGKLSVKENEKRKLGLLPLAEKKPRNTSGKPVQIATNIRRLVITPNKPIYKYAVKVLYVFRTAENTEARVEMSKSARSGTEHENDKVRCQKVYKKAVERYDDLKKGGPFFYDRQACMYSLSKLKVDNISFNVTEDVCKRRNFIRAEFELCKVEDSFQSTSNDVKKTVNRCPGLADRTLIEALNVIVSGPAFENKNVITVGACVHYLIDPSQIEVPYKEYQEGGLYSGVGASKAVKTSEGTGKEPSLFMSTEMKSTLFHPDYVPLVELFKTYKGFSTDLKANSPAALRIAKAFVGIDVNLDYGPHKGLLEDSVVMKIRGFSTSATDTFFLREDKKISVKDYFKSQYGITLKYPNLFTIEAKGKVGKVFLPAEVLTICPSQTVTNDQMINNEQADMIKIAAAPPHLRKRTTDAVVKNVGLASNNIYGFIKVEEPLKVEGIVLEKPKILYAQNRAADLNNPKARFPTDWNIAGQYFIAKSLQNWEICYIQGDEIGGLDEQLYREMAKNGMKVSKPVITYIVRGDLKAVFEKAKKAGRELVFFVIKQRYNLHQEIKALEQKYDLLTQEIKYETAEKVFRQAQTRLNIVNKTNMKLGGLNYQIGSNSFKNPGRLIIGFETSQRGGGGGDMPIAVGFAANMLDHFQKFAGGYVYVKRDRDVAIPPLPIYGPIVQSTLQKILQTVKKNRGAPSDILIYFNGVTEGQYALINEEFSASVKTACQKMHESFKPNITIIASSKTHNERLYQLEKNGIANLEPGSVIDHTIVSPVYNEWYHASSVARQGTTKTTKFTLIYTTIPNEPMSNLETLTNDLCYDHQIVFHPVGLPVPLFIAGRYSQRGAAVLSFNGAEYKDGEIDLEATNAKLGYGNKKLFETRFNA

***Caenorhabditis remanei* PPW-1**

MENEIANLVISENPSKTSAKLGLVPLAAKKPRNADRGKKVTVETNIRKLSIAPNQPIFKYAVEVNFVYQKPDGTECTIEMSKSTKKGTEHDYDKIKCQKVYEEAVGRYEALRKGGPFFYDRQASLYTLTKLQLEVVEINKNSYISSLQNIAFDVTQGISKRPNFKKAQFILKKVDESFQSTSNDIKKTVNPCPANADRTLLEAMNMVVSGPAFENKNVITVGACVHYLIDLKNIDVQSKFYEEGALYSGVGASKSVKTLEGSDRNAPSIFMTTEMKTTLFHPDDWPLLDLLKSYRGFTTNLKANTPAAQRIEKALVGLDVQLNYGPHVGLGADGIVMKIRKFSTSAKETNFLVDGKNTNVAAYFKSKYGINLKYPDLFTIEAKGKHGKIHFPPELLVLCPNQTVTNDQMINNEQADMIKMSAAQPHIRKSTTDSVVKQVGLASNNIHGFIKVEEPVKVDAIVLNKPKIIFHGNKFANLDDPKSRFPTDFNRAGAYFIAKDLANWEMVFVQGEEVKGLADQLVSEMRTNGMKTNPPAVSFIVHGDLNTVFQKAKAAKRQLLFFVVKSRYNYHQQIKALEQRFDLLTQEIRLETAEKVFRQPQTRLNITNKTNMKLGGLNYQIGSESFNKPNRLIVGFETSQRSGGNPDYPISVGFAANMLDHHQKFAGGYVYVKRSNNVFGSIVKDTLVKVLETTKKNRGVPNDILLYFNGISEGQFAMLNEEFSQHVKEACKFMSPSYQPHFTIIASSKTHNERLYKSDKVISSIPVFGRIVNLEPGTVIDHTIVSPVYNEWFHASAVARQGTAKATKFTLIFTTQPNEPMWNLEQLTNDLCYDHQIVFHPVGLPVPLYIAGRYSQRGAMVLNENDGPIFANGEVDLVATNAQYGYGNKALFNTRFNA

***Haemonchus contortus* PPW-1**

QNGMQPSEFLMQICSDS*GFFQVVDDELFA*IKEACEEFGNKHGRDSWMPRFTVIVATKRHNARFFV EKRGKIQNPLPATVVDTDVVRNDITEFY

***Pristionchus pacificus* PPW-1**

MAAPTAEMAKMTLKTAVPAPPQLPKGKKGTAKKVVANSYIVTLKPNVPFYLHDFRVVAVFMRNGEEKLKEVCKQTRDDFTEQERKQAAMLTYLAMKKNGQLKGDFMYDRAALLVSLQEICKGGEHTTVLKKANAPELFALASIKEATEVRVTIKKAADTYQVSTNDLTSRNEATRKALLSVANLATSQTLFEDMSVEHD

**CSR-1**

***Ancylostoma caninum* CSR-1**

ELNGRDPWMPFFTVVVATKRHNARFFAEKGRFIENPQPATVVDTDVVRNDITEFYMQSHRPVQGTAKPTSYQLIVDENDMGSDEVQSLMLALCFHHQIVDAPVSIPEPVYQADEWAKRG

***Ascaris suum* CSR-1**

MGAYIQATITMAFTCGGPFAMRGDYWMQEPRVATVQCLKSHVVNALNFFKEESNIKAFPEHVVVFRGGVSEGEYAKKRETHENGEQSLSRNEQIDLPQVARER

***Caenorhabditis brenneri* CSR-1**

MQSESSFNRRGGHGRGGGGGRDERGAHRGGRGGGRIQESRRRDGDAEGYGGRGGYGGRGGSRGGRGADRGGSYRGRGGGSRADNHSRPVISDERCNGLLPKDKENTGDSSKINQNSNSRLVINMFGLELSERRVFRHVVQMKLLDNKSNKDHILTTMSSRGRGNRSSRQNDNFILLGKLLDEFAASKKDKKKSVYAYDGAQSLFSLEGISMVVKIQKEDAIQIPGISEFLKDSIKFLDGELEIRCDPDLEKPSFVQTEVQEWSDPRFYAYLDIVTSQSAIRSGRYLSQSKGLYVNTTHMRELRVKWAVAAEGVHKGCRIIGNEVPVPILELDPQSTQYYAPIQLNVMLDLAFPKEFPSNRSINPSLRLQRDVKLLLKFLKCNSYYEDINDWGTNTVTVNDVDYNAPKDSEYRKKYPNLKFPNLPAAVCGQGQNKKTMPMEYLRALPYQSIDRRVLEEFDLTPRANDPDKRWSILQSHFEQFGFNDEVMEQFGVRIRNDPFKNINEIDGERVTAPKIAYSVPVRVDDEKRDWKAQDKKFAKAATIDHLVFVLVAGYSRNFAVESKATEYIAHSFMERCKEKGMKIQKVEFKCWEGEKDSEQFLNKVFGDLVKHPRHNDSSFIPFVLLVSDDVPNIHECLKFEERMSDIPTQHILLKTVRKMHDNIEKKSQGGRRGYDLTLDNIVMKANIKCGGLNYTADIPRDLECWSDIPTFVMGMDVAHPDRMSAREGSPSTVGLSCTSAHHPYSFIGDFLFTDPRQEAIQDEILRKFTDQNVRNFAEIRGFPKKIIIFRDGVSCGEEKEALREVKTIEETIISAAKAMGYRDYSPKVLAIVVKKRHHTRFYAKGGNQGTTPTNPLPDTSMGGEIAEFGKRQIFIQAFRPIQGTAKVPSFLIIRDDEELSDEHITRMVCAVCSLHQIVNSPTSIPTPIFVAHELAKRGTGLYKAYRFKWGELNGDWDALTNQLSYSTLDRLSKVRVV

***Caenorhabditis briggsae* CSR-1**

MQSENRGRGGGGRGRGRGDRESGRGAGRGSYRDGGSERGPPSSSRDERESNNRGRGGYRGGSDRGTSSREGNESRGGYRGRGGPRNGQNSSYRGRGGDGHRGGRSNNDDNHARPVEAVGRCNGLLPDDKENKGNSSNMNQNNPRLAINIFGLELSDRTVFRHVVQMKLLDHKTKKDYILTTMSARGRGNRASKQKDNFILLRKLLDKWAGRKGGKKTDVLFAYDGAQSLFTLEGISELMVIKKEDALEIPEISEFLKDSIKFLNGDLEITCEPDLEKPSFNQTEVNEWSDPRFYAYLDIVTSQSAIRSGKYLSQSKGLYVNTGHMEELRVKWAVAAKGIHKGCRIVGTGHPLPILELDPQSTQYYAAIPLSQMIQYAFPRDFVPNRMINPNMRLQRAVKLLLKDLKCNPFYEDLADWATNTITVSDVDYNAPKDPEYRQKYPNLRFPNFPAAQCGNGPHRRLMPLEYLKVLPFQSVDRRVLEEFELTPRANAPNERWTTLQHHYEEFGFNDQVMQEFGVQICNDPFQNISEIDGERVLAPKITYADPVHVDDEKRDWKAQDKKFETPATIHHLVFVLVAGYSRNFDAEIKATEFVARAFMQRCNDKGMRIDNYELRSFEGERNSENFLTSVFKNLVTHNRYRDPSFIPFVLFVSDDVPNIHECLKFEERMSDIPTQHILLKNIRKIRDNIEKKSQGGRRAYDLTLDNIVMKANVKCGGLNYTADIPRDIGSWREVSTFVMGMDVAHPDKMATREGSPSTVGLSCNSAESPYSFVGDFLYTDPRREAIQDEILRKFTDQNVRNFAERRGFPKRIIIFRDGVSFGEETEALREVKIIEETCIAAAKSMGHRDYAPKVLAIVVKKRHHTRFYAKGGHHGNMPTNPLPDTSVGGEIAEYGKRQIFIQAFRPVQGTAKVPSFLIIRDDEEVTDDHITKMVCAVCSLHQLVNSPTSIPTPVYVAHEMAKRGTGLFKAYRFKNGELRDWATLSDQLSYSTLDRLSKVRVV

***Caenorhabditis japonica* CSR-1**

MNQNQTPRLTINIFGLELENRTIFRHVIQIKLLDPKTKKDYILTTMSARGRGNRASKQGDNFILLGKLIDKWVEKTEGKRPIFAYDGAQTLFALEGISLLLTIPNEEAMEIANISHFLKDSIQFLNGDLEISCEPDLDKPSFRQTEINEWSDPRFYGYLDIVTSQSAIRSGRYLSQSKGLYVNTQHMEKLRVGWAVAAKGVHKGCRVVGTQGPLPILELDPQSTQYYAPILLNEMLLYAFPREFQSNRPLTNPNQRLQRGVKLLLKDLKCNPYYEDKGSFATNTIIISDIDYNAHRDQEYRDRYPKIQFPHLPAAVCGMGPRRRLMPLEYLRVLPFQSIDRRVLEEFELTPRANAPEERWKTLQSHFDEFGFNDEVMQAFGVEICNDPFNNVSEIDGARIDKPSIAYADPVHVDDEKRDWKAQDKKFVAPAKIDYLLFVLVAGYTRDFKGDTMATEYVARAFMQRCREKGISLGNFDFYSHQGERDSENFLTSVFKRLVTHPKYHDSNFTPFVVFVSDDVPNIHECLKFEERMSDIPTQHILLKNVRKIRDNIVRKSAGGRRAYDLTLDNIVMKANVKCGGLNYTADIPQDIACWRDVSTFVMGMDVAHPDKAASREGNPSTVGLSCNSADNPYTFIGDFHYTDPRQETIQDEILRRFTDHSVRNFAEFRGFPKKIIIFRDGVSSGEENAAFKEVGIIENTIVSAANSMGLCDYSPKVLAIVVKKRHHTRFYAKGGYRGTQPMNPMPDTSVGGDIAEYGKRQIFIQAFRPVQGTAKVPSFLIIRDDERVSDDHISKLVCAVCSLHQVGFLVGLSLLIIGIQLVNSPTSIPTPVYVAHELAKRGTGLFKAYRFKRGDIRDDWHTLTNQLSYSTLDRLSKVRVV

***Caenorhabditis remanei* CSR-1**

MQSENSNRGGRGGGGRGRGARDREDRDPGGSGRGGRGSYRGGRGGSERGGSQEHRFSNRGGRGGRGSHRGGSENGPLSGYRSDRESNGDQRGGRGSHRGDYKGSERGSHHRDDNASRGGYRGRGGHRGDGDGAYTRGGGSDRGRRDHADNHRRPVETNDREQRYESTPHPRLALNIFGLELSERIVFRHVVQMKLLDHKTNKDYILTTMSARGRGNRASKQNDNFILLGKLLDRWITTKGTKKHPVYAYDGAQSLFTLEGISQNMVIQKEDALQIPGISEFLKDSIKFLNGDLEISCEPDLEKPSFVQTEINEWSDPRFYAYLDIVTSQSATRRFTIFQCKKLLNYYSGKYLSQSKGLYVNTEHMEELRVKWAVAAKGIHKGCRIVGKEHPLPILELDPQSTQYYAAIPLSKMIQYAFPRDFGSNRIVNPNLRLQRAVKLLLKAIGRLTPSLFRISTTTLQKIRSLNRNIQTSTLPAAQCGTGPHKRLMPLEYLKVLPFQSIDRRVLEEFELTPRANAPNERWTTLQHHYDAFGFNDNVMQEFGVLICNDPFQNISEIDGERVLAPKIAYADPVHVDDEKRDWKAQDKRFETPATIDHLMFVLLAGYTRSFDQDIRATEFVANAFMQRCRDKGMIIKNYELKHFEGERDSETFLTSVFKYLVTLREYPNPAFIPFVLFVSDDVPNIHECLKFEERMSDIPTQHVLLKNIRKIRDNIEKKSHGGRRAYDLTLDNIVMKANVKCGGLNYTADPPRELGAWKEVPTFVMGMDVAHPDKMASREGNPSTVGLSCNAADNPYSFIGDFLYTDPRREAIQDELLRKFTDQHVRNFAERRGFPKKIIIFRDGVSFGEETEALREVEIIEQTIIAAAKSQGLRDYAPKVLAIVVKKRHHTRFYAKGGQNGNTPTNPLPDTSVGGDIAEYGKRQIFIQAFRPVQGTAKVPSFLILRDDEEVSDESIAKMTCAVCSLHQLVNSPTSIPTPVYVAHELAKRGTGLFKAYRFKNGELHDWATLSDQLSYSTLDRLSKVRVV

**PPW-2**

***Ancylostoma caninum* PPW-2**

AEHITRFLCHGHQQSTLPTHIPAVLYAAENLAKRGRAAWKSKINENSDGASSSASHRISLREGETAEDFYSRISQELMNSLPNHYFA

***Caenorhabditis briggsae* PPW-2**

MPATPLPPAPMPPVTAPPGPMPPPPLPPAPGPVLPITSAHKTANDACIKRLSQIGIMESPRIYPSNQPPGTYGKEILVQSNVFGIEVENEMELFQYSVCIRADVAVKKEVVFTKKGKDDFVVLDRHEKCCTVLYHAYEAHDDFFQSAKNCLIYNGQSMLYSSMDLFSNRLDADIKSRTFQIDGVEIGHKDLLKLPCIKLEISATKNPVVKFNRQDVGKRSADQNMLAVNTAYRNILELALNQSCVQDYNRCAVFEHGKIFFIRPTEEGFISRDCVDVGDGKRMMPGIKKTVQFVEGPFGREECNPAVVIDGMKVAFHKEQPIIEKLQEVVTSDFSKGLKELDRERCAGVIKGLDCYSNYMGRLRHLKIEGIHHESASKARFQVKDGNSSTVADYFQSRWNITLKYPEVNLICCKEKGNVNFYPMELMFISPNQRVKISQLTSAQSQRTTKESAVPPDVRQRLIMTGKTAAKISGDSAVLNKLGVQICEAPLLVRARQFPPVKIALNPTSSVIPQKEGKWRIGQYSRPAAAPKVWAMYAVGTPSSRFSQSQLTKFAREFVTVAQQKGIQLNPPSDSQCVLATDIESQLAAAHKASCEFVFIITDDTITNLHQKYKMIESLQSIVVQDMKMSKALSVIDSGKRLTLENVINKTNVKLGGSNYIFTDTKKYLDRVLVIGIGISQPPPGTKFIVEGKGFLNPQIIGFAYNGKQKQEFAGDFVLCPAGQDVSGFSASKIVISFQTLSPIEDIMKESILGYKKWHDGNDPETVVVYRSGISEGNHGNVLAYEIPLARAGINEVSKSVKLVYLAVSKDHTYRFFRNNLDAIGKSSSSGTQVSASASRTSVLIPKKKSFQSSQSQLKPCDINIPSGISIDQMVTNPAIKQFFLNSHTTLQGSAKTPLYSILADDTRASLESLEELTYNLCHLHQIVGLPTSIPTPLYVAAEYAKRGRNLWNEANLKNPLVRSGSEREQLRAATHSINYKHTGDFSDRRVNA

***Caenorhabditis japonica* PPW-2**

MPGPYPLPPVTAPPVGYPPIAVPPAPHPPVAQPPVVVPPVGLPPPPVPPVGPGPIPVLPVESTHKTSNDACIKRLRQLLVDPAPRIYPTAVQPGTAGAPFLVQTNVFGVEVRKRTQIFQYVVHIKADLTNKKEVVFTKKGKEE

***Caenorhabditis remanei* PPW-2**

MPPMPPYPLPPVTAPPGAFPPPPMPPGPTPALPITSEHKTANDACIKRLQDLNVLPAPKLYPLPKDPGTAGHPLDILTNVFGIEVHKSVELFQYNISIKADLTPTKEVVFTKKAKEDFVVTDRHEKCCAVLFHAIERNQDFFHAPENSLIYDGQNMLYSTFNLFGDLPMGSTKFFNNETLNFQTKVIQVNGAEMGHKDLDRLPLIKLEVYTTKNPSVRFSQDDLGRRTSDTNIESINRAYHNILELALNQFCIRDFSRCVVFEHGKVFFFKPLEEGYERRDCIEVGDGKMMYPGIKKTVQFIEGPYGRGSNNPSIVIDSMKVAFHKDQTVMEKINDINLKPCGDGLSDFERDKCQAVIKGLDCYTSYTGRVRHLKIEGIHHDSALKARFQLKTGGTSTVHEYFQQRWQIRLQHPQANLISCIERGKQNFYPMEVIRVSPKQRVKITQQTGKQSQITTKAGAVPPADRQRLIMTGKNAAKINQENKILSDLGLQVYDDPLLVPARQLPRVKIAEHKTGPELPERGNKWRFNHFARPATPPEKWAIYAVGLQNSKFDRGNLAAITDRFLHACKFKGLNMPEQIDQDLVNGNNIEERLAMAAKGGCKYVLVITDDNIVNLHQKYKVIESLRGMIVQDLRMSVALDICSNSRNETIENIVQKTNVKLGGSNYIFSDMKNLLSGVLCIGIGISNPPPNTKFAYEGRGLLNPMVLGFAYNGMAEQEFYGDFVLSPAGQDTIAPIEDIINQCILGYKKWHDGNPPKAIIVYRSGVSEGNHGNIISYEIPLARTAISKSTKLIYIAVSKDHTFRFFKNEDVTSSASASKSQSQTSVGSRSSTSSRGPKVSEQNIAPGLVVDSVVTNPACKQFFLNSHITLQGSAKTPLYTVLADDSYASMDRLEELTYSLCHLHQIVGLPTSLPTPLYVANEYAKRGRNLWNEANVKDPAIRDADSERTRLQQLTDSINYKSAGDLVDRRVNA

***Haemonchus contortus* PPW-2**

RYPTLFTVSERHNPSTYYPVELLVVAPSQRVTLQQQTPDQVASMIKVTSVLKLLEVEYQIVSQEVKGSKVDSVMFKNQNQTLDNVIAKVNFHTHIPM*CFAERKMCLDQHRFETESGKATVEQYFKKHYNIQLKYKASATLPAVRIQQTKVMKDALGITSGNAKLSSAGISVEDLLTNCFQMMGATLGELIVEILKKFKAATSRAPRHIVLYFSGISEGFLRVSKWLNDEARLFVGFEISNPPALSKMEIERGATYKVGFLSNTTECVSFRNFFNMKLGGVNYVSVQDFGASVS*IIILISKEINFAF

***Oesophagostomum dentatum* PPW-2**

KDLENKMKTAAQHSCKFCLIVTADSITSVHKQIKLWERELEMVTQDVKLSNALKVVNERRVVTLENILLKSNLKMGGLNYELELPRDETAKNWVRPGRLFLGIGVSHPPPTTKFDP

**SAGO-1**

***Ancylostoma caninum* SAGO-1**

FRILLHSLKVEIERGATYRMPSVLGWGANCAKNPQQYLGDYVYIETHQSDMMGAKLSELIVTILKRFRAATDVAPRHIVLYFSGISEGQWSMVADTYMRAIHTGIKSLSASYKPSLTALTVSKDHNERIYKANITGNRATEQNIPPGTVVDTKIVSPVINEFYLKDHSAFQGTAKTPQKGLV

***Ascaris suum* SAGO-1**

MSKTTWLANRNHYLLPAKCEKWHVVALVGPSERFFSNDKLRAYVRAFMNQCRNRGMQMADPMVVDYVRGAREQEVDVRMQKAKQMGATFVHFVTSDMLKFHGHIKLVEMQLQIVTQDLTTRTASQAPQKWQTLDNIVNKTNLKLGGINFGLILENEIFLRFSAQKWLMNEGRLVVGIDVAHPPLAAVRGIDRTKVPSVVGYSSNCKKFPLEFIGGYRYATANMEELTDNSIRDVIVDSIRKFQVNRGKLPDHLFILRDGISEGQYKYVVVSEVEGVKKACGLVGGIGYRPNITYIVATKLHNMRLFKKVHL

***Caenorhabditis brenneri* SAGO-1**

MSRRNATSFVDNTLTSSGISGSGSMSPPITSRPPSGQASPLSSNGSLSPPHVDDQGSVSYNSDSPRDLSPLLLSELACLNMREVVARPGLGTIGRQIPVKSNFFAVDLKNPKMVVIQYHVEVHHPGCRKLDKDEMRIIFWKAVSDHPNIFHNKYALAYDGAHQLYTVARLEFPDEQGSVRLDCEATLPKDNRDRTRCAISIQNVGPVLLEMQRTRTNNLDERVLTPIQILDIICRQSLTCPLLKNSANFYTWKSSCYRIPTAAGQALDLEGGKEMWTGFFSSAHIASNYRPLLNIDVAHTAFYKTRITVLQFMCDVLNERTSKPNRNPPRGPGGPGGPGGPGGYRGGRGGARGGGYGNFGNRGGPPNANTSRDDFGGNGLTFTMDTLSRDTQLSSFESRIFGDSIRGMKIRATHRPNAIRVYKVNSLQLPADKLMFQGIDEEGRQVVCSVADYFSEKYGPLKYPKLPCLHVGPPTRNIFLPMEHCLIDSPQKYNKKMTEKQTSAIIKAAAVDATQREERIKQLASQASFGSDPFLREFGVAVSSQMIETTARVIQPPPIMFGGNNRSVNPVVFPKDGSWSMDHQTLYMPATCRSYSMIALVDPRDQNSLQTFCQSLTMKATAMGMNFPRWPDLVKYGRTKEDVCTLFTEIADEYRVTSTVCDCIIVVLQAKNSDIYMTVKEQSDIVHGIMSQCVLMKNVSRPTPATCANIVLKLNMKMGGINSRIVADQITNKYLVDQPTMVVGIDVTHPTQAEMRMNMPSVAAIVANVDLLPQSYGANVKVQKKCRESVVYLLDAIRERIITFYRHTKQKPARIIVYRDGVSEGQFSEVLREEIQSIRTACLAIADDFRPPITYIVVQKRHHARIFCKFPNDMVGKAKNVPPGTTVDTGIVSPEGFDFYLCSHYGVQGTSRPARYHVLLDECKFTADEIQNITYGMCHTYGRCTRSVSIPTPVYYADLVATRARCHVKRKLGLADNTDCDTNSLSSSLASLLNVRTGSGKGKKSHASSVDDESISLPDASSDQILQDCVSVAGDFKSRMYFI

***Caenorhabditis briggsae* SAGO-1**

MPNVAVVEQLGALQLNENQAPMGDVPLAEKLPIGQPDGTTVKISTNMRAMILKKNTPVFKYDIKIIAYFKRDGREIAREISSSHEKGPKKENDKSACSSIYRLACDQCPELSKGFFFYDRQALLYSLSDFKKEVIETTIAGKKIEGFPNFVKAEFKITNVAESFQTSSNEIGKAVNIRPARADVTLLESLNMMASGHALENPNVFTKNNCIHYLSHPDRNIAIGEVRGQGMQASSVGATKAVRVLQGKNPTPTAYLITECKYTSGNLIDQYETFSVKTTLFHPNHAPLLNVFREIRGFATNLTATSGWAREHIQNYKGLCCYLDYGSSEGLENDRKMVKIHSFGETARNQKFEKSDGKVSSVFDYFKGRYEKILKFPDLFTVVVMGARGGRLIPVELLTFCDKQIVKTQQMEAKVQADLIKMSASKPQDRKKITNQVADSIGLGNGDGHFFTLSPPEVVEGRVLPKPVILGGGIKPNEKKSCFWNTKPEGPSTDFSIQHFSDGKSLSTWDVVFHESEPLESAVHHLIGTMTQMGMKVNAPNFVFIKNNDLRTIFDNAKKRNVELLMFITKSNREYHKEIKVLEHEFDIRTQDIRFETALKFERQQNTRRNLVNKINVKLGGINYEVESPTFTKDRIIIGLETSQNSTMGDGLICVGFSANMMAKETQFCGGYMFTQRSNDIYGSVLKDIVRDVIKQTCTHPCRVQNKPQELFFFLSGITEGQYSLINERYSNLIREGWFAAVSGGKPISFVPAITIVAVSKIHNTRRYLEGTAFTYALCYNHQIIYSPISHPVPLYMAGDMSERGSNILGFHRANYKNEELDLARINAELSYSNRKLFGTRFNA

***Caenorhabditis japonica* SAGO-1**

MAAQLAEQIGAIQVNETSQPAPIYGMKPLAAKKPKEKNVEGTPVDVITNIKKLTISKNLSIFKYSVKVIHYYKSIQYGEKAIEISKSTLKGKEHEQNKEYCHVAFLKAIRQCDQLKGGNLYYDRQASLFTFTRFSDENEINITLTGSDICSRDAFIRCEFSLKKVDESFQTTSNDIPRTCNICPALIDRTPLEALNLIISCSAIDNPNVYTNDDCTHYLYNTDKVKTVKGVGGKTSVVGVKTSVKTLEGAGNAPCLYLVLELKNSFFHPNNTSLLNVLSSYPDFKPDIRSNSTFICKNRHAFVGMKVTRNYGKNKSLGNEGAIVNIRGVGLSAAECKIVVNNEQTTVEKYFKDRYNIVLKYPKLFTIEARGKFGNSEYIPVELLNVCNFQIVTTEQMSKKEQESLIALSAAKPHLRRATTEAVAKEVGLLSSSINEFLKVADQSERVKGVVLPKPTILFGQGRKVDWGALARTGNKKPDTDFMDVGQFFQPAQCLNWEVVFNEGGELEGAISQLVSAMTISGMKVQPPKVTFIRNEDLKTVFENAKAKNCELLVFITDKSCNYHLEIKSLEQKFQILTQDIGYATAEKLFFSKDTKKNIVNKLNMKLGGINYVIETPYFKDTKLVIGFESSQFGGLNSPTTVGYSANMSNHFQKFTGGYHYVKRSSDVYGPILKDIVFRILTTLKANKQRARPQEMFIYFSGVTEGQFAQINEVYMPAVKDACRMIGNNFQPHITLIAATKKHIERFFGTDPVSGAVTVGICNLKAGTAIYETVVSSVLSEFYLASAVARQGTIKTTKFTIVSTTNQKEPLYSLMKLTNDLCFLHEIIYHPVGLPPPLYLAGELSKRGAKLYSFRVSEFDKESRENLEFINQELGYSGRKLAGLRFNA

***Caenorhabditis remanei* SAGO-1**

MSNISVVDRSMAELNLSNSTAKELPIGRKPLSQKLAKGDRKGPFVQLETNMSKIELKPNVTIFKYAVEVLMFFKKENGEEFSIELSKSRKRGFDHEHDKKTCAAVYEKACKKSKELNSGGNFVYDRQALLYSTTKLKTDPLSIIVDDKEVCSRLNFIRAELKISKVADSFQTTTNDVSKTVHNCPALADTTILEVLNLMASGEALKDSKVLTIGNCVHYLYDDAGIQTHPMFYDQGLKSSAVGASKSIKTLEGIEKQPSLYMATELKTTLFHPDDTRLIDLLKSYPGFDSNKTANSFWATNVQHSLQDLFCFLDYGKNKNLNDDRITIQIARFGDSAVNQKFEYENRETGISKPVSVFEYYKMKYGITLSYPNLFTIVAKGRGGKNSYFPVECLQLCNGQPVRTSQMIGTEQADLIKYSAALPAQRKEKTDKVVKALKLGTDEIGLMRVNKPETVTGRVLEKPKIKFGPGGRMVVNWEDPRQRGAATDFNITKFIDPKSLTNWDVVFDEGEPNPVAIENLMETMKMMGMQVAPPRQLFIQKSRLRPIFESAAAKKVQLLLFITKQRNNYHQEIKALEQEFDILTQDMKFETAVKLPRQQNTKKNIVNKINVKLGGLNYEIESPELQKRNSLIIGLETSQKGGLGDAPISIGFSANMMDHPQKFSGGFVFVKRSSDVYGPVLQKLIHQIIKQAQSRRTGELNQILIYISGITEGQYGIINEKYSMFIMEACRAIHPNAFRPEIIIVAVSKTHSTRLYKNHSGQLSNVDPGTVVDEVIVSPVLTEWYLTSAVARQGTAKPIKYSLIFAVLKNNPLTATQLQKMTYDLAFGHQIVYSPVSLPVPLYLAGECSTRGAALMATRKATFTNGEFDMAATNNKLGYIGKNLFNTRFNA

**T22B3.1**

***Ascaris suum* T22B3.1**

MTVAAYFGERYGELKYPKLPCLHVGPITRNIYFPLEVCMLDTPQKYNKKLTEKQTSAIIRAAAVDATSREQRISALCEQAAFQKDPFLKEFGLHINPKMCETTARVLNPPRILFGEKNRYS

***Caenorhabditis brenneri* T22B3.1**

MSRRNATSFVDNTLTSSGISGSGSMSPPITSRPPSGQASPLSSNGSLSPPHVDDQGSVSYNSDSPRDLSPLLLSELACLNMREVVARPGLGTIGRQIPVKSNFFAVDLKNPKMVVIQYHVEVHHPGCRKLDKDEMRIIFWKAVSDHPNIFHNKYALAYDGAHQLYTVARLEFPDEQGSVRLDCEATLPKDNRDRTRCAISIQNVGPVLLEMQRTRTNNLDERVLTPIQILDIICRQSLTCPLLKNSANFYTWKSSCYRIPTAAGQALDLEGGKEMWTGFFSSAHIASNYRPLLNIDVAHTAFYKTRITVLQFMCDVLNERTSKPNRNPPRGPGGPGGPGGPGGYRGGRGGARGGGYGNFGNRGGPPNANTSRDDFGGNGLTFTMDTLSRDTQLSSFESRIFGDSIRGMKIRATHRPNAIRVYKVNSLQLPADKLMFQGIDEEGRQVVCSVADYFSEKYGPLKYPKLPCLHVGPPTRNIFLPMEHCLIDSPQKYNKKMTEKQTSAIIKAAAVDATQREERIKQLASQASFGSDPFLREFGVAVSSQMIETTARVIQPPPIMFGGNNRSVNPVVFPKDGSWSMDHQTLYMPATCRSYSMIALVDPRDQNSLQTFCQSLTMKATAMGMNFPRWPDLVKYGRTKEDVCTLFTEIADEYRVTSTVCDCIIVVLQAKNSDIYMTVKEQSDIVHGIMSQCVLMKNVSRPTPATCANIVLKLNMKMGGINSRIVADQITNKYLVDQPTMVVGIDVTHPTQAEMRMNMPSVAAIVANVDLLPQSYGANVKVQKKCRESVVYLLDAIRERIITFYRHTKQKPARIIVYRDGVSEGQFSEVLREEIQSIRTACLAIADDFRPPITYIVVQKRHHARIFCKFPNDMVGKAKNVPPGTTVDTGIVSPEGFDFYLCSHYGVQGTSRPARYHVLLDECKFTADEIQNITYGMCHTYGRCTRSVSIPTPVYYADLVATRARCHVKRKLGLADNTDCDTNSLSSSLASLLNVRTGSGKGKKSHASSVDDESISLPDASSDQILQDCVSVAGDFKSRMYFI

***Caenorhabditis japonica* T22B3.1**

MSRRNATSFVDNTTLTSSGISGSGSMSPPVTSRPASGQASPLSSNGSLSPPQYVDDGGSVSYNSDSPRDLSPLLLSELACLNMREVVARPGLGTIGRQIPVKSNFFAVDLKNPKMVVIQYHVEVHHPGCRKLDKDEMRIIFWKAVSDHPDIFLNKYALAYDGAHQLYTVARLEFPEDKGSVRLDCEATLPKDNRDRTRCAISIQNVGPVLLEMQRTRTNNLDERVLTPIQILDIICRQSLTCPLLKNSANFYTWKSSCYRIPTAAGQALDLEGGKEMWTGFFSSAHIASNYRPLLNIDVAHTAFYKARISVLQFMCDVLNERTAKPNRGMQRGGPGGPGGPGGYRGGRGRGGGYQNFGNRGGPMGGQGGPMMGQGGRDDFGSGPTFSMDTLGRDTQLSSFESRIFGDAIRGMKIRATHRPNAVRVYKVNSLQLPADKLIDFIDFSPNFYRFFTTFFTDFWFFSAYLIIFLENFPKTNDFFIIFPTKTPFFEISENSDFIDFSLNFLAFFLFSPANLIVFLVISTKNNDFSIFFPTKTPFFEICENSDFIDFSPIFLAFFRETPPTFLCRSVCVSSNITVRVHCLIDSPQKYNKKMTEKQTSAIIKAAAVDATQREDRIKQLAAQASFGSDPFLKEFGVAVSSQMIETTARVIQPPPIMFGGNNRSINPVVFPKDGSWSMDHQTLYMPATCRSYSMIALVDPRDQSNLQAFCQALTMKATAMGMSFPRWPDLVKYGRSKEDVCTLFTEIADEYRVTSTVCDCIIVVLQAKNSDIYMTVKEQSDIVHGIMSQCVLMKNVSRPTPATCANIVLKLNMKMGGINSRIVADQITNKYLVDQPTMVVGIDVTHPTQAEMRMNMPSVAAIVANVDLLPQSYGANVKVQKKCRESVVYLLDAIRERIITFYKHTKQKPARIIVYRDGVSEGQFSEVLREEIQSIRTACLAIAEDFRPPITYIVVQKRHHARIFCKFPNDMVGKAKNVPPGTTVDTGIVSPEGFDFYLCSHYGVQGTSRPARYHVLLDECKFTADEIQNITYGMCHTYGRCTRSVSIPTPVYYADLVATRARCHVKRKLGLADNNDCDTNSLSSSLASLLNVRTASGKGKKSQASSVDDAESFQPADGSSDQMLQDCVSVAGDFKSRMYFI

***Caenorhabditis remanei* T22B3.1**

MEVREHLDFFSTGTIKETEMSRRNATSVVDNTLTSSGISGSGSMSPPVTSRAASGQASPRSNGSLSPPAFVDDQGSVSYNSDSPRDLSPLLLSELACLNMREVVARPGLGTIGRQIPVKSNFFAVDLKNPKMVVIQYHVEVHHPGCRKLDKDEMRIIFWKAVSDHPNIFHNKYALAYDGAHQLYTVARLEFPEATIRLDCEATLPKDNRDRTRCAISIQNVGPVLLEMQRTRTNNLDERVLTPIQILDIICRQSLTCPLLKNSANFYTWKSSCYRIPTAAGQALDLEGGKEMWTGFFSSAHIASNYRPLLNIDVAHTAFYKTRITVLQFMCDVLNERTSKPNRNNNPRGGPGGPGGPGGYRGGRGGGRGGGYGNFGNRGGPPNGGGRDDFGGNGLTFTMDTLSRETQLSQFESRIFGDSIRGMKIRATHRPNAIRVYKVNSLQLPADKLMFQGIDEEGREVVCSVADYFSEKYGPLKYPKLPCLHVGPPTRNIFLPMEHCLIDSPQKYNKKMTEKQTSAIIKAAAVDATQREERIKQLAAQASFSTDPFLREFGVAVSSQMIETTARVIQPPPIMFGGNNRSVNPVVFPKDGSWSMDHQTLYMPATCRSYSMIALVDPRDQTNLQTFCQSLTMKATAMGMNFPRWPDLVKYGRSKEDVCTLFTEIADEYRVTSTVCDCIIVVLQAKNSDIYMTVKEQSDIVHGIMSQCVLMKNVSRPTPATCANIVLKLNMKMGGINSRIVADQITNKYLVDQPTMVVGIDVTHPTQAEMRMNMPSVAAIVANVDLLPQSYGANVKVQKKCRESVVYLLDAIRERIITFYRHTKQKPARIIVYRDGVSEGQFSEVLREEIQSIRTACLAIAEDFRPPITYIVVQKRHHARIFCKFPNDMVGKAKNVPPGTTVDTGIVSPEGFDFYLCSHYGVQGTSRPARYHVLLDECKFTADEIQSITYGMCHTYGRCTRSVSIPTPVYYADLVATRARCHVKRKLGLADNADCDTNSMSSSLASLLNVRTGSGKGKKSHISSVEDETFSLPDASSDQILQDCVSVAGDFKSRMYFI

***Haemonchus contortus* T22B3.1**

CSREFQCLGPVPIEMRRTRTSNLDERVLTPIQVLDIVCRQSLTCPFIGNAANFYSWESSCYRIPINCALALDLEGGKEMWTRFFSSAHVASGWKPLMNIDVAHTAFYKAKISMVQFMCDVLKDKTTQMGMVFPKWPDLVKYGRGRDDVVVLFNEIANECKQTSISCDLVMVVLPGKNSDIYMTVKESSDMIHGIMSQCVLMKNVQRPSPATCPNIIL*VSIKLGGINSRIVADNITHKYIIDQPTLVVGAVRVYRVNSLQMPADQLTSRGVTEDGRETIKSATQHFVEEYVELRFSKLPCLHVGPPTRNIFFPLEVCEMDTPQKYNKKTEFLKSGLSV*PRMFETMARVIQPPQIMFGGNSKMVDPIVHPKDGARSMDNQALYLPATCGSYSMIALVNPRDQNLPQGFCQALVIHPTQAEERKNIPSVAAIVANIDLLLQSYGANVKVQQKCRESVVYLLDAIRERLVSFCGNTNQKPTRLKKLSEKQKSSIIRAAAVDASQREERTDQLCQQAGFDSDPFLEEWSFGLMRLEADVPLAKDSRERTPCAVSFQSLYKEFSLSNHEMKILAEAVKGIKIRISHRESPQQRHLARQALSTCQQGLSESTTAFADRLRNLIRAATTGQDPGSLVNSHFSATGSELHLCLSTGVSLEERLSRHHPPQQYGSNNRCFN

***Pristionchus pacificus* T22B3.1**

MSDLNSKFESMGISSSGYETGSNRRGTSNDGSKGSETSGGPPQRKADLAYNHLELAVRPGQCKAARAKINVTTNYFRLGAPTTMKYVSFRLDFFNEGRNRGTWEPVKKEKMSDCFKNLFSDKRNLFPRRDAAVVYNGTDMMYCPPDHRISHTEVQFQNNKGAKFRGKPARPSFTRCIITPLGLSEISTVQVDDEANRCALMQFLDCILTQEHRFDLTGRAARFIQHGHSLFKIPHSAEEKRLDVIPIGKGEEVWLGLHAAVKCEGEELMLNADTSGSIFKSRNLNLIEFYAQIVHGYTQSLVDVDFDRLTIDPKSLGDLENILKGTKLLLKHPNGTEKAKKFDGFTRNSADREMFNYNGRQISVAEYFLYECNTRLRCPKFPCLKFFNKVTKQNNSIPFEFFYTLDEPARFKGKLTDQQLDVFVKAVCQNPKDKKSRIQRFCTRGGEYLGETNIMKEWGVSLDRKMIEVKECPVLPLPYMEMQASAPQVQVNGETGEWILTENVNGKEEPIKPIELNEDRVLLFIAFVGHNAMGIQDRDRCYLLRDTLRNYGMNVDNDIDSMGTPPLTQRKGRSYDTNVPFFNQYFDKLKQKVARKTSESGGVKYTPLIIWVFPRRDSATYAAIKYHCDVAHGVASQCTVRKTYEKLMSNPNTNTSAQNLLLKILAKTGSVQFRLSSHAHKNVERLQNPEDPVLIIGIDVSHPGKEERFDAIKDHEQKTGQNAQQANKFRGQHEKLPTARPAAKTLFSELNFYDCPRSVVSVVGTTDIKGARYGVSSRVQRIGQEETVNMIEPFKERIHEFFNNTGKMPAHIIVYRDGASDTQIKKIAYEELESLERAIESFGENDEKKPTITYIMAAKRHHTRFFRSNLDEIPHGTPHLNCPPGTLVESSIVSRNKWDFFLQSHYGTLGTALPTRYTVVRDDWKTIVFAHCFGNARCPRPLSIPVPLHYAHLAAKRSKVLYDYYKDNACDLYRRSKEDLVREDLEKQAITPHNNIISMKGMHYA

**T22H9.3**

***Ancylostoma caninum* T22H9.3**

WWYQQPRLHRVQYITIHFERAFLNYHKRNKRLPTEVFVYRSGTSEGEFSEVAEEANEIRIVAEKMRDLNSGRPYRPKITVIVAQTNSNYRIMPASMPPPTGGRPRAQDFNVPSGTCADSVIVHPRYREFILTSQQANIGTSRPTRYTIVTEDKPQMSVDDAEHITHFLCHGHQQ

***Caenorhabditis japonica* T22H9.3**

MDPFDRAMSLMGSNDGGTKRERKVSTKEEPAMKRRMETTTAPPRIFSTPTSAAPAGRESFAAIEHIDVQMNMFPISIVRMPPRLQRLCFETTLVLPDGKSFNLNTGLVAVSGDVNSHNRRLAQHTIFRKFLDKNRHLFGNKDHHYVVYDCAATIYVPDGYYSGGEEVKFELRRDDFREDEWERVSKLCRKRGCYFSIKMTPTGFVYTSGENLLETSNRMELTRCIEIVTSQYLNSPDFYQFGNATFPLKQRPVSEPDSVTEVRPGFAKVARLVDGRMGRNEMLITIDTKFSPFYKNITVLKFIVSKYEDLKGSRGGGGRIGGGRDDHRGGYGGHGGRGDYRDSGRGRGGGYGRDNYRHANDSFGGGDSMRSEPSSREIEEVQKGISEGGSLRSAIEASMKGLYAEATHMPKDSNRIVVITGIADSNAEK

***Caenorhabditis remanei* T22H9.3**

MWSQQSQSVNDRQSSGGTFKRGLISSEMGGPSAKKIAPVLEDSFKVTKTFKVTTNMLPLNVWSMKEVQRIKTETFIKVLSVHSLICSVLTNNVRLRQLAVMTLIPYLRPKLFPKQRDFVFDATTLYVPEGQYIGETVEEFKICKKQFENLPGFWKRSISRFLRNDDDGFLVRLTKDGYIPQGDKALEEEAHRSEFIRFLGILTSQKSNKEVYFHKGNLSYPVDSKQEQLDSTAYTRAGFAKSIFITRENAAVTMSINTQFCSFYRTLPILTLVSNMHKEFKNGVATNSIDEAEKCLRENSEFLRIVRKELIGLYVKATHLKEVIPYCSKPLRTYFSEFITLEGHSITVAEYFYKIYNITLQHPRMLLIIGNLFGKERVFPMEVLKVAQYQLKHSMSIKDKKKYNEAIELFSAPESYMRDVASVISDPLELNNSSLMKAFGIEYRFNAPVISQAKLLSCPDIRIGAEVKRLQLGSSEIPQTGSFEQPANIHQAAVISFDNILSKKKVRDLAVRLEEICLEKGMKTSSKFCAHEMSVEDYMGMDRSRMGWKNQKIGIVFGIVENEKSDVYDALKYYRITKVQTILLTKETVDKILNDNTETINKVTRKINMKCGGINFLINIPRTIDGKESALHKKLRTSVKYIGIKTYQSIDMKNHRMTTIGVSFNVSPSSRLSGHCYQEMHHSRKIQAIDSILLESLESYKKRSKQYPATIVLYRSICNNEDFSMVKKEVEEIRESFVGRNDAYSPSLVVLAVQEDALVRLFVPAADFKGPNTNVPPGTCIDTEIITYGYDEFILNSHTPTKGVSVPARYTILANDPSWTKNEIAHITWFMTFNNQVAYKPHAVVDVLHFAGKLAKRGTNILRFEQNITPSDSDAA

***Meloidogyne incognita* T22H9.3**

MASTLEQDVTNSGSAPTAAHIGVLLNGYQMDIRKTVPQVFQFELTFVGERTVNVQGQQNVKKQQFERGPRNDESKETRRRLIWGLFQQLLDQNRDVFGDNKKTFTYDCATLLYSISDLRMKQGEKREFVLNVERSALEDRAKAYIRNTERIVAYLLRTETVNLKNAVQSYLQGDRSVVQFLELLSSQRMFEKGQHYSFGNRLYEQESTVQLQGVPQILKSGMQKNVRFVGETLQNAMPILQIDPKKSAFFPGVDLLNFVCDFLGVNHPDVIMQNMGRYFDQMNKQIKGLVLQTTHLSENQVTFANSGLTNRPANEITFQREGQTISVVQHFQQTYQMNLRFGRLPCVIQKAGPKESFYPMELVKIVQGQRVPIDKQTPKLTEQMIRQCQVLPAAMQEQITIQRDQGMLQNMNSYFTAHNVRIDPQLCKTEASMLFPPAIQYNPDRVEPSQNGALDWKLIGGGAQNRRFTNPVEWPKLWAVVILQNCVQMDQCQRFCEQLVQTAQRRGIGNAMLPQRVDHYTESSIDYVSEKFQFYATHRCMFVLFFADGKERAHAGDFHHVMKYCEQKYDILTQHVGPKMMSKGVGGGGGGAMIFENILMKTNLKMGGSNFNIVTPDVFKRAIRNNQDVFSEIWMGGRRMFFGIGMSHAPPQTLFERQTGKAPAIPTVVGMAYTTSREMLKLRGTYWFQQARTTIMKEEQMRPLVDAVKNALYIYETENGDQFPEHLIVFRGGASEGEFKKVSRWEGGAIELACKEIAHERKGKFNIPAITIVVCQRQSNYRIVPERINSRGRAPEQNCQPGTVVDKGVMNAALTEFLLIPHKALQGTAQPLRCTVVHEFRPPQGPSARMPQMKLEEIEYITYALAYSHGICAMSTAVPGVLYSADELAKRGRNNWKMHTSDGSNAQVFELPPPGQGQEEAIQALDNQRTNYFAEVTDQLNPKIPTKFWA

***Oesophagostomum dentatum* T22H9.3**

AYLDAFGVRTDENFITSNAKVLSAPEIKYKTGSLQPDRSGPMLKWRLGSCQFVRPAEVRSVSLVVFDQVMGSEQAMQFYHSLARAGRERGMTIADDSAKVKHLPSEVDEEIESHFRSCAGKVSMILCITRQKKDPVHDTIKMLEAKHKVVTQHVSMDTAFACMKGGGAKTLENVLLKFNAKNKGLNHTVSTPRQALGRFADQRDMNARLFHAKMFRWFRTLTCCCPIVV

***Pristionchus pacificus* T22H9.3**

MAERTGGSGQPMPTEDEQTAAIERMKLPDRIGEPGSNGAAFGIPTTVTLNVFRLHLEKVPGKIFKFDLQFIACGKNGKDFEINNALHKSPEYLRSKKRHALVAFLRAAHESEKEYFRQQQIGDPDVNYSWQRPLFLHVLAWRSTHDRRVCYATYMWRHCCAFDCGNAYFTAVELPNHSGAIPEELWKASDYTQCYIPNCEGPIKWELKKAEEFRIDGEFAKSPQAQAFFTILATQNLSRSDEIDVKKDAAYSLEGETTSEHKELRKGILAASRMVGSDASIQLHSKIGMFFKPQPLLEYLRESSGKHSTMELVAFLRNPRASERLRREIFNLPLQMRHIKSQRIFECKGLAATSAIETTFPYNDRVVSIAEYFLERYQYRLQHPTLPLILERNKRGQSFHPIECLDITAERVSNQKMTPKGQEEMISKSCRAPAVLARDLETSRNIAQLDTTNKYLNAFEVKIKDGFSELPAKLLAKPHVIGMAGIKPEITDIGKIGYSKGSSFKFAGPVKLEKPIVLFIVDNAIDIASP

**ALG-2**

***Ascaris suum* ALG-2**

EVKRVGDTVLGIATQCVQAKNVIKTTPQTLSNLCLKMNVKLGGVNSILLPAVRPRIFNEPGLFASGRE

***Haemonchus contortus* ALG-2**

*KNQSVLSPHLKAWKMWILQLNAIFVIEVLRHCAFNGLTCLLTERRALSDAQRVKFTKEIRGLKIEITHCGQMRRKYRVCNVTRRPARGLFFNSSHCIPKPRRLLSKMNSEKKTSLHFPTWPEPYRYGFSNFVCHLFVGQEQKHTYLPPEVCHVVPGQRCIKKLTDTQTSTMIKVKRVGDTVLGIATQCVQAKNVIKTTPQTLSNLCLETGQTIECTVAKYFYDKYRIQLKYPHLPCLQVR*KHRLIFLVLQYELRAMREACMMLESGYQPGMTFIAVQKRHHTRLFMCHTYVRCTRSVSIPAPAYYAHLVAFRARYHLVDREHDSGEGSQPSGTSEDTTLSNMYAATVRVQQHRQEIISDLTYMVRELLVQFYRNTRFKPRRIVVYRDGVSEGQFVL*ATARSAPEREREIASLVRKAEFSADPFAHEFGIAINSAMTEVKGRVLSAPKLQYGGRNKATALPNQGVWDMVAVTKICQSLILVPVFKDQVGKAFNIPPGTTVDVGITHPTEFDFYLCSHAGIQGTSRPSHYHVLWDDNQLTADELQQLTYQVC

***Meloidogyne hapla* ALG-2**

MSWRGQSQQGRSGGSSGGGGGYGGGGRGGGSGYGGGGGRGGGGGGYDRSSGGGGGRGGGGYGQSGGSRGGYDSGRSGGGGRGGGGGGYDSGRSGGSGRGGQVATRPMRNLQVAKLTEAENPSNFKGTLGRKTDVVVNYFKLKFNTRMHIFKYRVDFFHQQEDKRKEISTTNKMVLRQWFWKFVQANEAYFGGRNNLVYDDSHLCFAKNQLPVQSGTVGEVKLETDEGRRDGNKATFIMVIKPTGTITIKQQETHEDDAFRVLNLIITQQARCPIGALDKTIHCEISRLYIKPPNSYHEPLDLGGFEMLRGLYAYAKRGLAPPKGNVGHGFVNFDVAHSAFYKVGCDILQFYAFAKTGRFLNVSEIERLGDYGMNQSQRNELRTLLSGLKMKKKRIAENFIESESSFCDVVDKSPDAYKFECEELGKTVSVTEYYAAKYNYRIKFRSMPLIKIMPKERNLVLPIEVLRVSDKLQRLRRKLPDALQAMTNQFTTTPPRQRFSDIDQMAKVECKFYANPVMNTFEVAINTEFLEIGARVLNPPRCEFSKSFRDIMGIKAVEKPNLFGKPAVNIVFDLVIVDKCISFERYRKAWSALVQSCSNRGIPVHPEPAGVTDFDTRHKDQLANLIAHRSQEAKSWGQGVMPIIIVAMPDNRWIYQSFKGICELRFGIRSQMISQKTWNKLSNEAERGGPMSSAVARNIFLKINAKCGGVNCKIDANATEKWGTFVKKESPTLFIGIDVTHPAPGDTLSPSISALVGNIDLNATRFTATVRAQHHRTEWIEDMGEMFNERLIHFQKGSKLFHGSELRPAKIIVFRDGVSESQFQGVMDFEVSSIRKTIDQYFQGVQKPKLTVVVVQKRHNTRFFDKNDNDEKNKGNLQPGTVIDGHITSYAKEFYLCSQKGMLGTSRPSHYFVLMDENNFTADELETCSNNLCYLFARAPMPVSIPAPVYYAHLACFRARHHYAAADDDLPVGKNGQPLTKQKLASGDKILQQPSEAVIVEQGLKPSMYFC

***Meloidogyne incognita* ALG-2**

MQRSDGRSGGGQRGYGGGGGSRGGGGGGGGQRGGGGGGRRDEGSSGGYRGGGGGHRGGGGGGYQSRGGGGGGGYQPRGGQDRGGGSSRGLQGFSQGYREEPARSSSSTSSQPEQQSLPAHMMAPPVQQQQKELMPSQKRVDELSCPPPMPPATGFVQKCQGICSIYTLKVEPGRRAFRYDVDISRLPMPKRNGTMDDGKSLVRGADDGQRALNRNLCFELITLAFTTNNRFGMPVGHEIVYDCKAVMFTSMPIQPFDIQGGHQFILENHQVNDYVRAYCGDGQNVRFLVHIKRNSSVPELDLNDFTQYMNGTSVFNEDHSLRTFFEIAISQFALNNELFVSIGAGKLFEYFDPSQNPKRVGNGMYLRKGLSKGVKVIKNDNPFTNGKNGGGNNNKGPRAAVVLDAKVALFFEPQALHLTVLDMLPRSNGRQTRLSDHPPKVWQEVERLLEDLRVEVVYRRTRTFQLGKFTEKPLRDLYIDVDLPNGAGTNFMSLAEYFLWKYNIKLEYLDLPCIKSNSYLPPGKEA

***Trichinella spiralis* ALG-2**

QVKSCGDITLGLVTQCVLPKTISDVAIKKSYSTMLNIAMKINMKIGGINTKLLEDEVYDIEFMNALDNYLYKNNALVIGVDVVHPSAVETHLPSIASVVGNVDAKVTKFHASVKLQPAKQELITGFIEQFSERLLEYLDVNGTAPKNIIVYRDG

**ERGO-1**

***Caenorhabditis brenneri* ERGO-1**

MTNSNEITTSMGNVALDVVGNEPLAEKNKKKTPGTAVQIVTNMRPVTITKNTPMFKYDVKVMFVYSKADGKEFVKERSKSIFKGPEHERDKGLCSLAYKKAVRQCPELQKGGPFYYDRQASLYSLSLLKKDPLTLNLTGNDLSQKSNFLRVEFTVTKVADSFQSTSNAIKKSVNIRPNLADKTILEALNLMVSGKALEDPNVLTMGNCVHYLYNDDHIKMDRVRVLDGEKNSAVGTCKSVKTLEGRDKDPSLYLTTELKATLFHPDGYTVLDVLRTYPKFNATRQANDAWSIPVRDSLLGLYCYVTYGSDANLGVERRMVKIRGFGLSARQQTFKRDGQPTTVLNYYREKYNINLRFPDLLTVVARGREGQSENYPVECLELCPAQPVRTEQMIGNEQSDLIKLAATAPHNRNRITEQVVQSVGLGNDREGYVKVGAPEVVTGYVLPKPTISYGGKTVNWNEPGKRGPATDFNITKFLRPARLVNWEVVFDKNTQLDSAIAPLVNTMKEMGMQVENPQRSFIVNGNLKPIFTEAVKNKRQMLLFITPDQNNYHQEMKALELEHDMLTQDIKFTTAERYVRQPNTRKNIANKINIKLGGLNYDVESKYFDKNRLVIGFETSQKGGGGDAPIAIGFSANMSDHHMKFTGGYFFVKRSSDVYGPIIKNVVKLCIDQTKKNRAAPTSIVVYFSGVTEGQYGLINEKYVGAIKKVCESFSGNYRPHITVIAATKLHNTRLYKQDQRGVSNMEPGTIIDETIVNPLLGEWYNSSAVARQGTNKAAKYTVIFNTDKTKPLEMWEGLTNDLCYDHQIVYHPVSYPAPLYVAGMYSHRGAEVLAQRSAVYKEGEFDFEATNKQLGVFDKKLFATRFNA

***Caenorhabditis briggsae* ERGO-1**

MSNYNDDYNRRGGGGDRGGYNRNNYSNRDDYRDNSRGGNDYREGHNDRNNYRGGGGQDNYNRNDRGYDRGYDRGHDRGDRGHQDYGRGDRGHQDYGRGDRGHQDYGRGDRGHQEYGRGDRGHQDYGRGDRGHHGREDYGRGDSGRGGYGRGGYGKVATVKDLQMDRMSLNGGGRGGGNNMYGSQREGRGGYRAPQRCTAGMSNLGELAGGADHPNMPVAKKDLAHNQQKFYERPDTSAYENPRCGQKIELLTNHALVHMPSTPIVLYEYNIDVFKGRKKLEKREEAGPMFREILQTVPKHIFPPATDFIYNDVNLIWSTMKLPSHERKVEGRNRNSFYYKYTRCVELKFELATNCQDSQLISTLVDAIGTARVTWPKQTGNKFSVFKRSIFLLQDRLDEGAFADAPVFVKLRNGVDARIGVSMAIRLNLRVGITACFDVSHTLFTRPGYPLVRLFWELIQGETLEDDQILDGQWDADMQLARPTKRNIELMSNVLKKMTLIFSPEDGITIDEKGKYHETDLRRLKTRGREFKFYELGPDSSFQFLDEKAGKKITVAEYFLAAHNYRIRYPNLPCLQKKPSKMNPHRLVLFPMELVSLLVDPIRFTGAVTEKLKGDMVKYTTLSAVQRRLVLQNVIAQKAIGDCPQIVDNEDRYMKNHEIKIEKDMLTVKACLLPPPEVVYGNSTFLDTEHRGQWEAVINDPIRTVLEDAVYRRNRNPNAPKLKKRLLGSILKIGSPMNSTVGFDIDDSCYHNLMRAIHEAGQPVCWQNPELGQAAIQGSMEYLQKRDQPGTIMDWFNSLKENLDDYKQSEDEVIVPLVFVIFEVRFTTLNLQRQDFQNDYNLFKWLADNHVGIFTQGMLYKTFNGIGSTPATCKYTSQIVEKVLGKVGTTHRRLERNGEHKSWKKIVVEPTLLLGVDVSHPSTRDLMGEDQVKRLSVATVVGNIDLDCTEYRASSKIQDVGEERIVRFENEILERISEFVQFTGKRPAHIVIYRDGLSEGDFQRTLYEEKNSVNMACRSIAADFNPTLTYIVVTKRHHTRFFLKNEKEGLAEQGYNVRPGTLVEDTVTTKNYYDFFLTTQVGQMGLARPTHYYVLHNNWKVPGTFWPTVTHALTYLFCRATTTVNLPAPVLYAHLAAKKAKETMDGALYANALNGKFLDTSSFADLAQLERCIKNHQELDGMTFV

***Caenorhabditis japonica* ERGO-1**

MSRGGDNRYYDNGNRHDSYRQNDRHREQYNSNEDRYHNHSNRYENSSRERDGSGFNGRSRNNYSNNHRYDDDELARNMRQMNVSGGVRGGGRGGQNIYSTYSSGGTGSRGFTGTLNDSTAPPGSARNLDGGIDKKQLPYNDQKFAERPEFMIQEKKLGEPVMLYTNHTLVHLPEKTMRVLQYNVDIFNNGKLVTKREEASVLFWEIYKKRRNMFPAIHAFIFNDVNILWVIESDKFRFSEGEIATAENPKRPKLYLKYSTSFEFGKDVDKMQESSLLSTLIDAIATQRSRKPTPSNCYTVFKRLTFLVQDKAVEKGLDGIALYHPFKFGTDARVGFSIAVRMNLRAGPTACFDINHTLFNRPGYPLVRLLVELCLNEVITDEEYEERYDQMIKDSQIDEANRATMHKILDKMTLHYQFETAMSVESARKMQDNVPAGRGAGSRFDKSFKYYNLGLPADRAFFEYQKDEYSQKREMSVAEYYKLEYGYALRYPHLPVVIKKPSAKQRNGMLVQYPMEFVSYVVNPTRYSGFTPESLRAEMVRETAYTPVQRLKLLQHVMEQKGIAGAQAPVDNDDPNMRAFGLSMEKTMLEVKGTVLPVPKLLYGNSAFLDSDHSGSWEAVRNEPIRTVLPESLYERKCIRGQQPRKKRLIGSIFLIESPNHNSGASLNYDT

***Caenorhabditis remanei* ERGO-1**

MSFNRYRGNGGGGYRDDGRNDQENGRGGHHQGGSGGRADLSHDMRRMDINGGRGGARGGNMYSHNGPARPRFERSASGVANPGDLVGSAHYPRIPVAKKDLEHNKQEFFKRPEGSVYERKVGNKTELWTNHALVHLPTQPYLIHEYNVEIYQNRKKLEKREEAAPMFRIICASKNGRSALPRSADYIFNDVNLLWSIEKLPNSQETVGDRRNYFVYKYTQSFEFGEGIAQRDSQLLSTLIDAIATARVRNPRLCQNKFTVFKRSMFMIQDEKYREDFDDAPLFLELKNGLDARIGVSIGIKLNLRAGITACYDLSHIMFTRPSYPLIRLLVEMIAGETINDEDFENHWDNSLKNAKVTHGNRDKMKSILHKMKLCYTLESALEVDATGEVTNNGALGNARQANRDFKFYDVTEKSAQELMFFNEELGREISVAEFFLNRRNIRLRYPNLPCIQKKPSRMNGNRFIAFPMEFVTLIAEPKRYAGLTTLEQKSEMVRWTTFTAKQRLVVLQHIMAQKNITDVPAVVDNNDRYMKRHGITIDKEMLSVKASVLPPPTVVYGGNDKFSDVHHEGEWKAVDHEPIRKVLEDAVYKRTTDKSAPKLKKRLLGSILKIGSPYNNKVDIEIDDTCYHNLMRAIESAGQPVCWENEEMGQAAIQGSTEFLQGVHLPAVIYNFLHDLKTNIDDKYKKSDDEVIIPLVFVIFEQRFTNIVNSRNMFRNDYNLLKYLADTQLGVFTQGMLYSTFNTIGATPATCKFTRLIVEKILGKVGTTHRKLESGGTHKSWTKVTNPKEPTLFLGVDVSHPSTRDLKDPESDVKKMSVATVVGNIDIECTEYRASSKIQSAGEERIVRFQDEIKTRIADFTMHNSIRPAHIVVYRDGLSEGDFQRTLYEERLAIENACISFDPAYQPSITYIVVTKRHHTRFFLKDESEGIEEQGFNVRPGTLVEDTVTTTNYYDFYLTTQVGQMGLARPTHYYVLWNTWHGCLPTFWPTVTHALTYMFCRATSTVALPAPVLYAHLASKRAKETMDGAIEAHRLMGRVFNMDVYSDVAELTQQINNHQDLDGMVFV

***Trichinella spiralis* ERGO-1**

MHWVIGVGCGSSISCRKRILPINSFRWVVGNVDTKVTKFHASVKLQPAKQELITGFIEQFSERLLEYLDVNGTAPKNIIVYRDGVSEGQFMQVLEEELPALRRACKSFATNCRR

**PRG-1**

***Ancylostoma caninum* PRG-1**

YTGQRAEWAKYVKNNGNFRGVCLQNWVVIAPNSNDGERLSRDFISEVEYICAAMQVQYGHPMLQFCRESSANGYCNAVRDAIARAGNQSIHMMVLILADDSKTRYDMLKSWLCTETNIPSQFIQLSTLRGRPQDRGRNRNFGSIVLKIVLQMNCKMGGALWKVAIPLKRAMIVGYDLYHDSTLRGKTIGACVSTLDQDYTQFYSQTRPHENPTELGTNLGFFIRKALYQYYTNNNRTLPEKIFLYRDGVGDGQIPYVKDQEVVLVQQACAEAVQRAEGVGKDFKIKLAFIIVTKKVNMRIFKGNPNSTLTNPDPGTVVDSVVTRPERYDFYLVPQYVNQGTVTPVCYNVIYDDTELSPDKHHKLAFKLCHLYYNWQGTVRVPAPCQYAHKLAFLVAQSIHQEANQDLRAKLFFL

***Caenorhabditis brenneri* PRG-1**

MASGSGRGRGRGSSSNSGGGGKTQEYFGTIQPDLFVRQPGENKVGSSGRVQRCFANFIPIEMEKADYSIYQFHVEFDPTVDSKHMRERMLLHKNVTDEIGEYHVFDGMILYLTNEWNQNQEIEVPHPTTGDLIRVIFKQTNRFLLDNAQTINIFNTIVRRCFDEMGLTQLGRHYFNSKDSRNVREYNMSILPGYETAIRMYENQLMLCVENRFKMVRRDSMFQLLRKEMQACQNNRMRVQEKMNEMYGGSTIITLYNNKLHRFTRLDWSISPMSEFQKDGESITLKRYFKEQYNKDIQNDDQPIIISEGKPKQPGEPPQINYIVPELCFPTGLTDEMRKDFKMMKEIASHTRMSPQQRLTETRKLIEQFHNNEKVASCLNYWGIRLADDLANVNARVLKSEPLHAEGSKKYEGRNAEWARGVKESGIYRGSNMTNWIVVGPNTGNSGMLIQKFIGEAARLGNTLRVQIGDPMCVPIGGVTPNDYLEGVKRAIQQVNGEEVHMLVVMLVDDNKTRYDSLKKYLCVECPIPNQCVNLRTLAGKASDGGENKNFGSIVLKIFLQMVCKTGGALWKVNIPLQDTMIVGYDLYHDSTLKGKTVGACVSTTSGDFTQFYSQTRPHENPTQLGNNLTHFVRKALKKYYDGNNNTLPGKLILYRDGAGDGQIPYIKNTEVKLVRDACDMVTERAAKMSNKEHKPIKLAFIIVTKRVNMRILKQGANANAAINPDPGTVVDTTVTRPERMDFYLVPQFVNQGTVTPVSYNIIYDDTELGPDKHQQLAFKLCHLYYNWQGTVRVPAPCQYAHKLAFLTAQSLHGDSDEQLRDKLFFL

***Caenorhabditis briggsae* PRG-1**

MASGSGRGRGRGSGSNNSGGRTQEYFGTIQPDLFVRQPGEPKIGHSGRPQKCFANFIPIEMEKADYSIYQFHIEFDPTVDHKHTREKMLLHPNVVDEIGNYHVFDGMILYLTQEWQQNQEIEVPHPNTGELIRVIFKQTNRFLLDNAQTINIFNTIVRRCFDEMGLTQLGRHYFNSKDARNVREYNMSILPGFETAIRMYENQLMLCVENRFKMVRRDSMYDLLQREMQASNNNSQRVLEKMNEMYGGTTIITLYNNKLHRFTRLDSSITPLSEFQKDGQSITLKDYFKQQYNKDIRIDNQPIIICEGKPKQPGEPPQVNYIVPELCYPTGLTDEMRKDFRMMKEIASHTRLSPQQRLDETRKLVKEFHNNVRVRDCLVYWGIRLADDLANVSARVLKPEPLHVEGTKKYEGRNAEWARGVKDSGIYRGSDMTNWIVVGPNTGSSGILCQRFIGEAGRLGNTLRVQIGDPLCVPLNGVSPNDYLEGVKKAIKQVAGAQVHMLVVMLADDNKTRYDSLKKYLCVECPIPNQCINLRTLAGKSSDGGENKNFGSIVLKIVLQMVCKTGGALWRVNIPMQDTMIVGYDLYHDSTLKGKTVGACVSTTSSEFTKFYSQTRPHENPTQLGNNLTHFVRKALKKYYDGNNNTLPSKLVLYRDGAGDGQIPYIKNTEVKLVREACDQVTLRAAQMNNKEHKPIKLAFIIVTKRVNMRILKQGASGNQAVNPDPGTVVDTTVTRPERMDFYLVPQFVNQGTVTPVSYNIIYDDTGLGPDKHQQLAFKLCHLYYNWQGTVRVPAPCQYAHKLAFLTAQSLHGDSDEQLRDKLFFL

***Caenorhabditis japonica* PRG-1**

MSGGVRGRGSDANNSGERPSGMLGTIQPDLFVRKPGVSKVGSSGRAQRCFANFIPIEMDTPDYAIFQYHIEFQPNVDHKHSRERMLLHENVVSEVGEFHVFDGMIMYITKEWEANQIIEVPHPTTGVPIAVVFKFTNRFLLDNVQTINIFNTIIRRCFDEMNLTQLGRHYYNSRGGRSVREYNMSILPGFETAIRMYEDKLMLCVENRFKMVRKDSMFQLIANEMKVCQHNRLRVQEKMNEMYGGSTIITLYNNKLHRFTRLDWNISPMTEFEKDGRPITLKDYFKTQYDKDIFIDDQPIIISEGKPKQPGEPPQVNYLVPELCFPTGLTDEMRKDFKMMKEIASHTRLSPQQRLEETRQLLRQFHENARVSACLNYWGIHMGDDLANVSARVLRPEPLIGEKAKKYEGRNAEWARGVKDGGIYRGSNMRNWIVVGPNTGNCGLLIRKFIEEAKRLGDVLKVEIGDPLPSPIDGISPNDYLEGVKSAIRKAGNETVHMLVVMLADDNKTRYDSLKKYLCVECPIPNQCVNLRTLAGKANDGGENKNFGSIVLKIFLQMICKTGGALWKVNIPLNDTMIVGYDLYHDSTLRGKTVGACVSTTTTDFTKFYSQTRPHENPTQLGGNLTHFIRKALKQYYDENGNTLPTRLILYRDGAGDGQIPYIRTTEVKLVRNACDMVTERASKLSNKEHQRIKLAFIIVTKRVNMRILKQGATANSASNPDPGTVVDSTVTRPERMDFYLVPQFVNQGTVTPVSYNIIFDDTELGADKHQQLAFKLCHLYYNWQGTVRVPAPCQYAHKLAFLTAQSLHGESDEKLRDKLFFL

***Pristionchus pacificus* PRG-1**

MHDKINEVPQGSRPSSTETNNSGGFGRGSGAGDFSSKGGSGSGSNSGTHNSSTGKQQKDLGTVHRDLFTRPQGLDKKGKLGRAVACNANFVEIISDIDRPIHQLHVGFEPEIDAKQMRIDLVREQCVDIVGDHCIFDGMILYLPHNLSAENSKRVVKHKITGTDVTVTFRPSVTFGRNDPQAINIYNLLIREAFKAIGFVQVQRNFFDPKRSNLVKEWDVEVWPGYETAVRMYEEQMMLCIENRFKVLRRATVFQQLVSEFKRANGNMDLVKTNCDHTYTGSTILTLYNNRMHRLSRFEWDMNPMSQFHHAATDTKMSFVEYFKTQYGVEIKNMDQPLIVSEGKPKQPGEDPQVTYLVPELCNCTGLADSMRKDFKTMRVLSDYTRLSPDRRRESTLKFIQSAKANDHVAKMFNFWKVDFGDSLVDLPCRQLEPETLYGKGPQFSYKGHNAEWAKDVKSAGNFRTVNLDDWIIVCPDMPKAFDEAFKFIKECNNLGRTMQMNVNDPFKWKVDNPQATAYHEATKTAMENYTKETGKKAKMVVVIVNDDNKTRYDTVKKLLCHDMPVPSQVVKLETLAGKAGVNKNYSSIVLKILLQINCKLGGAIWKVTIPLEKTMIVGYDLYHDSTMKGKTVGACVSSYDKDFTKWYSQTLPHSNPVELGDNLRKFIRKALQKYHETNGGELPGRIFVYRDGCGDGQIPYVRDTEVRQVQEAAAEVGKVLDQEYAPKLAFTIITKKVNMRVFKKVGPVDLKNPDPGTVVDSVVTRPERYDFYMVPQFVNQGTVTPVCYNIIHDTTGMPPDRHHQLAFKLCHLYYNWQGTVRVPAPCQYAHKLAFLVAQSLHDESSEELRDNEIQAPILSPAHRGVVPASACSSEN

**F55A12**

***Ancylostoma caninum* F55A12**

EYQIVSREFKGSKVDAVVSRIQNQTLDNVVAKINEKLGGVNYNIMLGARPTDDVNKWISDKDRMFVGFEISNPPALSKVEIERGATYRMPSVLGWGANCAKNPQQYLGDYVYIEPRQSDMMGAKLSELIVTILKRFRAATDVAPRHIVLYFSPKGEGQWSMEADTPLRGRHTGIKSLSAS

***Ascaris suum* F55A12**

GISEGQFAMVLQYEVPLIRAAAKEAGCTDVKLTLIVANRFHNVRLIPSNVNPRDRASSQNIRPGTVVDTQLVHPAFSEFYLNSHRALQGTAKTPRYTIIVDDSRFEMDELEGMTFCLCFCHQIVAITTSLPTPLYVASQYAERGRRLLIQRNGGQETSSSSSGEAGHLDIXAANKELPYSGSRLAELR

***Haemonchus contortus* F55A12**

LQGTAKTPKYSLLADDSNIPLDVIESMTHGLCYLHEIVTSTVSVPVPLIVADRCAKRGHNIFGERATDQNIPPGTVVDTKIVSPVINEFYLNAHSAFQ

***Oesophagostomum dentatum* F55A12**

GSQLSELIVQILKRFRGATEVAPRHIVLYFSGISEGQWSMVADTYVRAIQTGIKSLSPNYKPNLTALTVSKDHNERLYRANITGNRASEQNVPPGTVVDTKIVSPVINEFYLNAHSAFQGTAKTPKYALVYDDSHIPIDVVEGMTHGLCYLHEIVTATVSVPVPLIVADRCAKRGHNVYIANSNQRNAVGSIDEANARLVNQGELQKVRYNA

***Pristionchus pacificus* F55A12**

MSKNIRHSLSLLTLEFVCYGNGNAYLLNPTDHGFPATFDVGCNDKYGGVGLTKGARIVEGPTGPAIAITVDTKKTAFHNDWQFVNEKMTQMGVQNDPVEATALLRGLKAVVFYSGLPQGEQMKRTITIGSVSRQQVRDVTFKDDKGATKRLTDYLRQQYKTTVNGNQFVVLDKYEGNAYSPDCLRIAPNQRVKSENIQPVNIEKLIRESASLPAKRLAETGTLAKCLAAGKANAEQARRGITVETAAPMLVDARQLAPVALGTGNGNVSANGPWRQGKIAQAPHADFKKWTMLHINNGGDPENLGETTARALVTYARNIGYNIQMALNVIHVRTESNQEAQMDQIFAHEKANGSTFIFCIAHARIKNHEYQYNHSDLLKAMELKHDVVTQQITSQVAQKVAHQSNTGTNVCLKFIEKLGGQNHYIARTTTDLPAWVTAKATMICGLQMQHPSALSGKEREANTMPSRPVAIGWSVNAVRGASDNGKDKIDQQIEKESTHFSSDWSFAMPKDYKDGEQYRASYKEAVLTMLRTYKEKRGCVPMRIVLFRGGVPEGDYDKIRGEEVPIWQAAFKQLNAAYSPSFIVISVNDAHSDRFYQQNIPGNEKAPKQNLPSGTVIDSGVTNPNLPMAYVQSHVPLQLITIDADHPVAATSAIDRRGSTTNRTIRASKSIRAFAGTAKVPSIVVQHCHLEPSHKLVVTMDDVIRMTYALAFSHGVCNTPTAVPTPLYIARESMKRANELFNFWNKRSGGNWTIDDIKQKFTMNPTVHVNCHGLRINA

**T23D8.7**

***Caenorhabditis brenneri* T23D8.7**

MEDQWLLSAIYDDGLVDRIRERRAASSRQTSIHVPSVENEILSTSSESRVYDDLYLHIVEEPKESFQLARKPRPSGFGRYIPLLANQFRITCTGARVYRYKIRIDPFIPSKKLNRKIIFSLKEQVLELRELNVVYDGVDTIYATDSIDIEKIGQLFVNVKGVLNTKESPNRFSVNLTYVDNFLLDTRIPPGNLDPHEKLRMMHAIDTIFRQTSSKTFHTVLQSFFSITPHLNQRTSRGFGWGTVNLGLGREVCYGFYQNVVETFDMMTMNLDVATTTFYRPIALVEFLAEVLEVPLATVTDGRALSDAQKKKFNREVAGLKIETRHCLSPRRFRVARCTWKPMESLKLNIVEDSEEAVSISMVSYFKTRYNIDLKYLHLPCIEVGRSRECILPLELCFIVSGQRCIKKLNEQQIANLIRATSRNATERKNAVLSLHDRIKLNDDPHASKFGLCVEDQLMRIDGRVLPAPRLLYCYPNSKQQNCVTTPNNGTWDMRGKNFYLGVEIRKWAVVCFAEPAIVPSHNIQSFIGNLCKVAREIGMPFVDDYCFCRYAQSDQAYSLLDYLLKEFVDLQLVICIVPGKSTVYGDLKRKGDLLGLTTQCVRTHNVSKNSPHTLSNLCMKINSKLGGTNVVISSPPPSVTSEPVLFVGCHLSRTSVSTPSDAASSVSHNETSIACIVGSVDGHPTRFAPVFRIQPRQMNTIVEMRDMMKEAILNFSRSTGYKPHKIVIYRAGIGEGTVDEILQTELRAVREACSMIDYNFQPGFTFIGLDVTHHTRLFASNEIDQIGNSRNVPAGTLVETGITVNNLFEFYLVSHAGIQGTSRPTKYVVMWDDNRMPPDEIHEMTYQLCHTQSRCTRSVSIPSPVYYAKLVAQRAKILMADEKFDVELFREKALTDGMLFA

***Caenorhabditis briggsae* T23D8.7**

MAPPKPSAKGAKKAAKTVTKPKDGKKRRAHRKESYSVYIYRVLKQVHPDTGVSSKAMSIMNSFVNDVFERIAAEASRLAHYNKRSTISSREIQTAVRLILPGELAKHAVSEGTKAVTKYTSSKEQFQLTRRPVPSSAGRHISVFANHFQISCNGSIVFQYHILINPDVLSRNLNRIIVNTLLEQAPELLESNLVYDGLSTVYTTTPIDVERTNQMLITVKGIKFTKDSPNQFSIFFTCVDRFSLDTKISYNKHDTYEKLRMIHAIDTILRQTSSARFHTVLQSFFSITPHTKIGPSHGLGWGTVNLGLGREVCYGFYQNVVETFDKLTMNIDVATTTFYRPIALAEFLAEVLEVPLATVTDGRSLSEVQIKKFNREITGLKVETRHCHTPRRFRIARCTWKPMKSLMLNIDNDKEASTSISMLDYFKTRYNIDLKYPHLPCVEAGRTRECILPLELCYIVSGQRCIKKLNEQQIANLIRATSRNATERKNAVLNLHERIEVGKDHYASEFGLRVQNQMMKLDGRILPAPRLIYCYPNSKRQDCVTTPNNGTWDMRGKNFYSGVEIKNWAVVCFAEPAIVPSNNLQMFIGSLEKVAREIGMPFVNEYCFFRYVPAEQATNSLEFLQEQFPDLQLVICIVSGKSTVYGDLKRKGDLLGLTTQCIRTNNVARVSPHTLSNLCMKINSKLGGVNVVLSAPPPSTATTPTLFIGCHLSRNSVASSSESSSSLNYSDTSIACLVGSMDGHPTRFAPIFRLQPRNANTIIDMSEMMKEAILNFRKTTGFKPHKIVIYRSGVEGDSIDEILQAELRAIRDACTMIESGFQPGITFIALDVAHHTRLFAANERDQIGHSRNVPAGTLVETGITVNNHFEFYLVSHAGIQGTSRPTKYVVMWDDNNIPSYEIHEMTYQDKSLSEGMLFT

***Caenorhabditis japonica* T23D8.7**

MEYAYRGDNKAVEQCSTIFMKVIQPEIVGSDILTIIFAAVAAVFAVLDIIFAVFIFSQQGKKKAVKIKSNMENQWQFSSLLDDLVDKKETDQSNSLRCTSISLKTIEDELLSLSPVSLLCDDPIFRHFEEPKKKFNLVAKPMASTSGRELSLFANNFQITCSGATVHQYHVQISPDIASKKLNRRVLRALEEEIPHLAYSNLVFDGWHTVYATKPIDLALVNQKVISMKGVVNTKESPNQFTVHFVHVDTFPLDCRIPPQQQNSDQKLRMIHAIDTIFRQTSSSRFHVVLQSFFSITPQRVINPEHGLGWGTVNLGVGREVCYGFYQNVVETFDMLTMNLDVATTTFYRPIALVEFLAEVLDVPLATVLDGRALSSVQKKKFSKEIAGLKVVETRHRNSPRRFRVVRCTWKPINALTLVHSNGIEDATSVSLLDYYKMRYNIELKYQHLPCLEVGRSRECLLPLELCYVVSGQRCIKKLNEQQIANLIKATSRNAMERKDAVLNLRTHVGIDEDPYANQFGLRVDQQMMKIEGRVLSPPRLLYRSPLSMRQDCVTTPNNGTWDMRGKNFYTGVEIQKWAVVCFAPSALVVPSSIRTFVSSLQKVAGEIGMPFMEDHCFCRYAQPDQALSLLQHLQHEQPLLQLIICIVPGKSAIYGDLKRGGDLLGLTTQCVRTHNVTKVSPHTLSNLCMKINSKLGGINVVISTPPRVMTEEPILFVGCHVARNSIVCSSADSSSSLTHIDTSIACLVGSMDGHPTRFAPFFRNQQRHQNTIVDMQEMMREVIVNFKMATGFKPHKIVIYRAGISDAVVDEILQAELRAIRDACASIEHDFQPGVTFIGLDVTHHTRLFAANGNDRVGNSQNVPAGTLVETGITVNNLFEFYLVSHAGIQGTSRPTKYVVMWDDNKMASDEIHQMTYQLCHTQSRCTRSVSIPSPVYYAKLVAQRAKILMTEEDFDFATFRQQEQTNKVKLGPRCLRTYGFTKLRPHYVILEW

***Caenorhabditis remanei* T23D8.7**

MEDQWLLSAIYDDDLVERIRESRGSCSRHTSINLPTFENEILSSSSGSRTFDEYYLPAIEEPREKFQLVRKPAPSFAGRYLSLLANHFQITCKGSIVYQYYIGINPSIPSKKLNRKILSLLEEQVPELLELNLAYDGMHTIYSSKYIDTRRINQISIDLKGTVKESPNKFTIFFTYVDNFRLDTRIPPENQTAIEKQRMKHAIDTIFRQTSVGRFHVVQQSFFSITPHLQVGPAHGLGWGTVNLGLGREVCYGFYQNVVETFDMLTMNIDVATTTFYRPIALVEFLAEVLEVPLATVIDGRSLSEAQKKKFNREVAGLKVETRHCASPRRFRVARCTWKPMENITLHISNGTDASLSISMVDYFKSRYNIDLQYRHLPCVELGRSRECILPLELCYIVGGQRCIKKLNEQQIANLIRATSRNATERKSAIMNIHERVDVRNDPCGSENGLRVENQMMKLDGRVLPSPRLLYCYPNSKLQNCVTTPNNGTWDMRGKNFYTGVKIKKWAVVCYADSAIVSPNNLESFIGNLQRVAKEIGMPFVEEYCFYSYIPPDDAATSLEILHRTYPDLQIAICVVPGKSTVYGDLKRKGDLLGFTTQCVRTHNVTRVSPHTLSNLCMKINSKLGGVNVALSAPPPAMTSDPTLFIGCHLARNAVPLVSDSSSSDSNMDTSIACLVGSLDGHPTRFSPMFRVQSRNSSTIIDLTDMMCEAIVNFRQSTGFKPHKIVIYRSGIGEETIEEILQTELRAIREACKLIEPNFQPGITFIGLDVTHHTRFFAANDRDKIGSSQNVPAGTLVETGITVNNAFEFYLVSHAGIQGTSRPTKYVVMWDDNQFPPYEVHEMTYQLCHTQSRCTRSVSIPSPVYYAKLVAQRAKILLADEKFDSQLFHEKSRLDGMMFV

**NRDE-3**

***Caenorhabditis brenneri* NRDE-3**

MSNPLDSLLPPDNGEKRPSSSSSKDPGGGRGGARDVWGNPRDPKKGELKENSSEYHRGGSSSTSTSRYRTGPGFSTPNQRAGPVQGFKREADASNREYDQYGAPDSKRRHEGASTSQHSPRDGPFYAVSKTGVQFNSWGLDMSKMDECIKKVLFEITLVAGERRHKLSDGIPMMKGDVNTQLKRLSSCVIFKKWCELNPDVFRGKSDPSGVLYEPDAVTEIKSGFYKVSRVVAAKSGPPEAILTVDTASSPFFKATSVLKFVVSKLQGGGGRGGFGGRGGGRGGFKGGRGGGGYGRDSYGGGGRNSYGGGGRDNYRGGRDRYDDRDHRDDRARRDDRYGDRRDDRRGNDDNRNDGGLMVMDFDERAVKDMERDFKRGRGPICDNLKTISAALKGLECYPLHLKGKQANCQLGTFVKPAKIKNVMVINFDPKFDEGCVKDFMSRLCKQCDQQGFRMEKRHDKWDVKTSHPDAFDDLRRYMERAKEKGMTIVIGIVEEKKPLMHDYMKYYEEKIGMQTIQITTDTARKFLPETRGAQTVNNVLRKLNPKCGGTNFYVQIAEKHRKGIACTDSVELYKKLYSRTQFIGFELSHTGARSRFDIQKGIYDGDPTSVGVAYSLRHASQLGGFTYFQDTRLHKLTHLDKKFRICLEGYLKADQRLPKHLVIYRIGSGEGDYEQILKEVEEMREACLKFEAEYKPRFVVILVQRRSRIRVFPEHIKGNNSKEQNVQSGTCVDTVGNAHGFDEFVLCCQTPLIGTVRPTKYTILVNESGWTKNEAMNVTYQLSFGHQVSYQPPAVPNVLYAAENLAKRGHNNYKTHSRLKDMSEYAGRISREFSGDVTEDMVAGILAQRYIELVSDEINDKTISNRNFWA

***Caenorhabditis briggsae* NRDE-3**

MDRKPFGSSRPPFSTPESRMKREYGEMKKEETEDYNPTEAKRRYGESSSGGSGGSFSPIDSEKVQLNSCELDISKMDPYINKIVFKVVMVMTNEKRIDLADGLVAVSGDVNRQSRRQALCLLFNKWLEKNPRYFQSNFYPFMAAGTVYEVTVKSTGKLATRGPRALEEDNRSELTRCIECVSNQILHTNNFLLYSTGIYPVNGNVLSEPTGTTEIKGGFSKVSRVTIDGSEVKAHITVDIARSCFFKASSMLKFISAKYMELKYGVSGGSGGRGGRGGGRGGFGRGGFGRGRGDRGGGYHGGGGDRRDSSYRHSDDRGFGRDRYDNRDRHSDRDRHRDDRHRYDEDRDRRRDYRDDRDNGRSSEDRENNRRSDYDPEELRKFEEDYKSGRLCLSALNAVIKGLEVRPIHLDNSKANRTVILDSLCKGSTETLSLEEEKMPGICLFKMPVVVSKKRQQYEFYPLELLEIIPGQRLKNNKMTGDIVSDSEALSCAIYLFLQQQFMTGANSSLPLDHIAQTRIILEDYLKLGGHQKNKHFDAFKIRFGDTRPIVVRSNILAPPHILFKPEKPHVIADGADVRIIAGRNETFVKPAKLRSIMIINYCGELREIDDFKRCLQGKFREHGFRYKDQDVQWIDERCDPSDLAKTKDLMKHTLRNKVSLVIGIALEKMPHIHDVLKYYEEKIGQQTLQLCAETVRKMGQGGGNKTTVDNVIRKLNAKCGGTNFYVEVPNAVKNRQVCINPEEMRKKLYKYTQFIGFELSHTGARSKFDKQKQTVEIDPTIVGVSYTLKHATQLGGFSYFQDSRVHKLTRVKEKFGECLRGYKHATEALPRTIVMYRIGSGEGDYDQVRKEVEEMREAADSFEPGYKPRFLMVLAQRNSHVRIFPEHINKGNARVQNVRSGTCVDSIGSAHGMMEFILCCQSAMIGTVRPTRYTVIVNDTDWTKNELMNVTYHLAFGHQVSYGPPSVPNVLYAAKNLAKRGQNNFKIHKDMINIRDKTKQLYAEYADVLGKKEKEEAVLDKFINDLSNQVNDCTISGRNFWA

***Caenorhabditis japonica* NRDE-3**

MKGFIAFCAPKRNSTLIKLLQIFTQGKAALTDTNRPELTRCIETVTNQILNNGNFFLYNTGTFEVQGNVLLEPDETSEVKTGFTKEPKVVEDENGNPSVSLTIDTVSSAFYKPMPMTSFVVAKYAELAGGERSYHGDRGRDRRTNESGHHRRDRGNESGSMSETKVKEFEKMFAKKNLITSYLEKVNKAMKGLECESSHLKNAKANRSVFVDKLYHCNAHEVVFDWKTESGTKQISVYEYYREQYSYTLKYPELPLVVSKRLKTQNFFPLELLTICPGQRIKNSAISAALQGHMTRTICALPFQHVQQTQRFLKDVLKIESSRVNPYLQAFKLSFPTNNPIRAKGRILAPPIIQFYKQNFIPDPRKSVRFSGKGKFIEPARIRSVAILNVDKGFRSVTKFAEQMERHCREQGITFEKPARDWKVVETHSEDTAGVKTFLSECLHKKVTIVVAIVNEKKPDIHDVLKYYEEKMGQQTIQICTDTAIKIMSEHGGKQTLENVLRKFNPKAGGTNFYLNIAPTVCGTSITPDATRLHEKLFARTQFIGFELSHTGARTQYDKQAELFDGEPTVVGVSYSLRQSTQLGGFSYFQPTREHKLSRLEEKFQICVDAYEKSAQSLPETIVVYRVGSGEGDFKRIFEEVNDMRKSAERTKQGYHPKFICILTQRHCHVRLFPERIQGRKAQEQNVPSGTVLETNVYRNGFDEFIMCCQTPMIVSFRGDFFL

***Caenorhabditis remanei* NRDE-3**

MPNEIAEQKIVLSESAFSMEDWEIVSRIFRRKDTTFEVTVKSTGQLYTRGPKALSVENRSELTRCLECVTNQIVHTNDYLLYNTGTYSLKGGALSEPDGVTEIKSGFSKVTKVIMGDDNTKPQAVMTVDTAQSAFFKSTSMLKFFHAKIEEANGRGGGGRGGRGGYRGHGGYGGDRGGGYDGRLQTSRVYRGHGGYGGDRGGGYDGRRDSYGGHRESYGGNRDGHRGDRYRYRDDRRDDRRDDRRDDRRNDRRDDRRDDRRDDRGESRRDRQNDDRTRSRHEIDYDTEAIKKLEREFEEGRITKTMLENISRALKGLECYPTHLKDVKANRGFTVHSIVDQSAINVKFDREVNGAFNQITVRDYYLDKYGLKLKFPNLPLIVSKRMKEMNFYPMEYLFIVPGQRIKQQKMTPDIQQHMTGQNSSLPAQHIRQNEKILRDHLKMEGGNCYLDAFKIKLESKEPIKMSAQLLAPPYIKFKPSQPIRLEGKGVRFPTNDRTTFVKPAELQSLLIISFDRNFRGVDMFEECLNRQFQSQGIRKDRDYKCIKHQQDANDADGIYRLMKNAKKDKVTLVIAICAEKKPEVHDTLKYFEEKLGQVTLQLSTETCEKMSRGGGNRQTVDNVLRKLNNKCGGTNFFVEVPEQYQGKSVCRNAAEMSRKLFQDTQFIGFELSHTGARTKYEQQNSIVEGDPTSVGVCYTLKQSAELGGFSYYQHTRLHRLSHVGEKFVKCIIEYKNAAGKLPKTIVIYRIGSGEGDYEKIRQEVDDMKKAAQKYEEGYIPKFLVVLSQRNSHVRIFPNQIREGGKAIEQNVQSGTCVDSIGSAHGLMEFILCCQTPMIGTVRPTRYTVIVNDTDWTKNELMNVTYFLSFGHQVSYSPPAVPNVLYGAANLAKRGHNNFKTFTRLGKTKDIMKKVMDSHRDIINPAEMAELVTDDYIETISEKINKLTIKSRNFWA

**SAGO-2**

***Caenorhabditis brenneri* SAGO-2**

MSSSTLENDLNAMSIKPSEQSNGKSNGVEKPSDISAPPSKQSNGDANNGEKTSDVPTQEQSNGATNGHVKMSYTKPLAKKQPPVHIAGARMVSVATNIRKLTLPPNQLIYQHRATINIVMRKMDGKEVVVEMSSSKWKHVEHEKDKRICRAVYLKAVAQLDVLKDGGQCFYDGQATLWTLKDLGIGEEPLSVTLTEGVSRRKNFVRAQFLLDRPNGPPVSTDDIAKTVHENPSLADRRLLDALGIITAEAPSTNVDVFTIKGTHHYLLDSRQAGVHKYSPREGREESSVGATKSIKTLEGRDGAPGAYMTTEMKATFFHESIPVIEKLRSFAGFHPRLRREDRLAERILANMKGMIIQMNYGQYKNLGIDGILFKITGFGAPPKYQTFNFNGQEICVDEYFNMKHGLDIHYKDLMTIEAKGSGGTLCYFPPEYVLISPNQTVTREQMINGEEQIISPLAGGAPHFRRETTENVSETVGLISENMAAGLSVTAPLVVDGYILKSPEISRDSQFVEPMTLSNWAIVFIAGEEIPPFANIITEEMRRCGMRVNNPEFLFISPHDLSDAFIDSKKRKRELLLFITKMNRDAHKYIKAAEQRWDVLTQHIHFETAMRIIDKGHKTTLLGCIQKINMKLGGLNYQTDSTSLDEKTIVLGFATSQKPSGYDDTKLVAVGFASNCLDDTHKFLGGYKYVENGKDVYGEHLADILETALKAKKRQSDVKPQRVVIYFHGINEAMFPTVRNSYVEICEKVFYQRGKTYKPELTIIASTKLHNERLYMEDRGEIRNLEPGTVVSSVIVSPVYNEFYHTGNRPFRGTTKPTKYTVVYAPEPVNMEQIQTLTNDLCYDTQIIRAPISVPTPIHIAMDSAERGSAIMFVNQGPYLKRESGKVDYWRTNEEYTYIRKNLGNTRFNA

***Caenorhabditis briggsae* SAGO-2**

MEKDMANLALSEGTKPSAPAKLGMVPLAAKKEKNASKGRKVMVATNVRKLTIAANQPIYKYAVQVNYVFVKEDGTECTIEMSKSTRKGTEHDNDKARCQKVYKEAVTRYEALRTGGPFFYDRQASLYSLTKLKTENISFDVDKGICKRPNFKKAQFILKKVDDSFQSTSNDIKKTVNPCPGKADRTLLEALNIIVSGPAFDNKNVITVGACVHYLIDTSGIDVANKDYQEGGLYSAVGASKSVKTLEGANRLAPGLFMTPEMKTTLFHPDNVSLVDVLKSYRGFAANLAYNTPVAQRIEKAFVGLDVSLNYGPYAGLGEDGLVSKIRKFSTSSVQTTFKWNDCEVNLVTYYKEKYGITLKFPHLFTVEAKGKVGRINFPVELLVLCPNQTVTNDQMINNEQADMIKNTSCRIKMSAAQPHIRKSTTETVVKNVGLASNNIYGFIKVEEPLQVEGIVLDKPKIIFAGNKLANLDDPKSRFPTDFNIAGNYFIAKELSNWELVFVENEEVKGLAEQFVTEMRKNGMKATNPVISYIVRNDLEAVFKRAKVAKRQLLFFVVKSRYNYHQNIKCLEQKYDLLTQEVRAETAEKVFRQPQTRLNIVNKTNMKLGGLNYQIGSETFRRPDYLIVGFETSQRSGGNPDYPISVGYAANMLDHHQKFAGGYVYVKRSNDVFGPIIKDTLIKILQTTKKNRATPKEILLYFNGVSEGQFAMLNEEYQQQVKNACKAISDTYRPHFTIIASSKTHNERLYKSDKGRIVNLEPGTVVDHHIVSPVYSEWYHASAVARQGTAKATKFTVIFTTNPKESLYMYEQLTNDLSYDHQIVFHPVGLPVPLYIAGRYSQRGAMVLASNMGPIYTNNVMDIAATNKEYGYENKGLFETRFNA

***Caenorhabditis remanei* SAGO-2**

MPPTGDEHDDSGPVSERSPRHHIGFQPLAKKESPHSGRGGARMINVGTNCRRIGLFSLIEFYIKLLRILVLSPKFTVYQYAVTINYVFSRPGGGEVTIPISKSNRMDKEHEKDKNRCRSVYTKAMSSYVNLRKGGTLFYDGQGILWSLSELENENISLIITEGISKRPNFIRAEFKLNKILGSGELSSDDVWKSVHQCPGEADKSIHQAISTMIAEGPNSRSSVLEINNTTHYLMKGNNLPIHLEKFPEGEISSAVGVTKSIKTLEGFDGNPSLFMTNDIKVSLFHPNLCWPLIKVFSTFNGFHCRLSAENSVARRIFEHHKNCYVVLDYGEFKHLGVDGSVMKIKGFAASARNQEFQQDSGLMISVYDYFRSRHNIEIQYPDLFTVAAVSLNADRRVSYFPPEVLRLAPDQKVSKERMTKEEESRLIRMNALKPEQRMDIVDRIVEQVGLTNEVNPDVFRIEQPMIVPAIVLPAPHLNYSSNNSRHFVEPKKLTNWEIVFLNDETCWDVGDILMNEMFECGMQVEPPSFSHIRNKDVHSIFTNAMRTGKQLLFFVLSKQTAYHEFIKACEQRYDILTQEINLEKARTLARQARTRRNIVNKTNMKLGGLNYNIGSNFLNEENILVLGFSLSHTAYGESEVVSVGYAGNILDRAHKFCGGFYYTKRTKDIFGDVIYDVLKDSLKTARKNRAMKAEKVVIYFNGIAESQLATVNEVYTKKCLECFASLKASYNPELIVMAATKMHSTRLFDSFQGRVCNLMPGTIVDTAIVSPVYNEFYHVGANAIQGTTKPTKYTVIHSSKRVDMEYLEELTNSLCFDHQIIESSISLPVPLFIASDCSERGTADLKYCDESLFENGEFQKDKINDMFTYKYKKLKETRFNA

***Oesophagostomum dentatum* SAGO-2**

EGASRGFRENKGQESVLIIYIDRAENRSHEYLKLLERRFLIPTQQLTAELAEKIQQQRQSCGNFVSKTNLKLGGINYEVVPESFARNRWIAGGKTMIVGYDVAHPGKPTRDEIMNKMPPQKPSVVGFALMELSIPKHSLVTTISKHLAAKGLKTLS

**T23B3.2**

***Caenorhabditis brenneri* T23B3.2**

MAEEKMTVNMPADAAQEEGRVFVVESNRRDEMIKFVLLIVLIIVFPPAAVAVHANECNMHVLISLILVFFFMIPSYIHAIWYCFFRQPTQLTIS

***Caenorhabditis japonica* T23B3.2**

MSEEKMTANVPADAEEGRVFVVESNRRDELIKMVLIIVLIIVFPPAAVAVHANECNMHVFVSLILVFFFMIPSYIHAIWYCFFRQPTQLTIS

***Caenorhabditis remanei* T23B3.2**

MAEEKMTVNVPADAPEEGRVFVIESNRRDEMIKLALLIILIIIFPPAAVAVHANECNMHVFISLILVFFFMIPSYIHAIWYCFFRKPTQMTIA

**Y49F6A.1**

***Caenorhabditis brenneri* Y49F6A.1**

MSGFRIPKRPRPAEGGAAPSSTPSSSSSSGPPISFSRPTDTPTGLTFQPIGSSPITTNVFKVDTRRMPNSFTRLSMETHLCGGKIEFKLSEGIVGVSGGINATDRRHALISIFRKIIQRHPDIFGTNLHMYTFDQATTIYAVEGNFKGQNKKYEEKFERKDFSDEEWMPISKIIRRENTWFKVVLSSNGVVYSHGEKAMSEKNRQELTRLIECVTSEVLNTPDFLQYGSQTFPLTQAPVFKPDPTSEIRAGFDKGIRLLEGNGKEPEIAMTIDTKLSPFYASTSLLKFFIAKYSEFKGVGGSGQIRHQRHSGERGGRPGDRRDQQRDRSRSRSPGRGGPRSLHKEDYDPKEVLEVQTAFSNGKDENLFRRIEDAMKTLCVSPTHLDKAHNRNIIVLKLGKSAAETYFDMHPGTDQQQRINVAEYFLLKYNYRLKFPNLPLVATGRVRNVSHFPMELLWIVPGQRIKSAKMSATVQSSMTGQNATLPREHIQRIQEILRNHLRFDRNPHMSTFKIDVAREPIQMNASLISAPQIRFENKCTASMNPGAVSFRQKNNVKFARPAKIGKIAVVAFDTVNTDLEKFCERLYGICQSNGIDCGGSWQHWIRLRANSNDTTRIKKEMAHWQSNGVTLFLGITPEKKPDVHDLMKYFEASLGLQTLQIHVNTADCFVREKGGAQTVENVMRKLNLKCGGVNFTLEIPDRAEGKSVCSNVTYAKQKMLDNVQFIGFEMTHGSARTLFDRNQGTFDGEPTIVGCSYSLKVSTDLGGFMYLQEMNEYRLKNLGTKFSDCFNAYQKASGSLPKTIVVYRTGVGEGDYKRVQEEIQDMKKAMEKISGDKPKLIVIVVQKTSHIRIFPKEIKGTKAVEQNVRSGTMVDGQITTAGRNEFILVSQTALIGTVRPIKYTIMTNEANWSKNELIHATYFLAFGHQVSYGPPAIPHVLYGAENLAKRGRNNFLTHKRLGELNQQVQQVLTEYEGIQDEEHQKELDSVLIDNITEAVNAMAVDNKNFWA

***Caenorhabditis briggsae* Y49F6A.1**

MSSFRIPKKPRQEDSAAGGSGGSSGSRPPGGPPGGSKPPPGGTYPPVRFTTPGSSSSNGKSFQPLSTVPVQTNIFSVSISKTSRKFVRLSMETILCGGKTEVKLSDGIQAVSGGLNISEKRLALKQIFRKVFERHPEIFGTNFLHYTFDCATTIYCVDGAYKGGEKKLEETLQQSDFTEEEWKTISRILRRQKTFFKIILSANGVVYADGPNAMAEKNRQELVRVVETVTSESLNTNTYLQYGSQTFPMREQPMMKPDATSEIRFGFDKAVRLAEGTDGVPEIVMSIDTKQSPFVAATSVMKFMCSKFSEAKGRGVGAPGASGAQRGRGGYGGDRRGGHGGHRQDPRDQRRRSRSRSPIERSSVDYNDAEVQEMQDAFQRGNEEKIFEKIQDSLKGFFLEPSHLPKDKNRNITVMKLTPGNANTTKFELNAGEGKTEQVSVADYFLNHHHYKIKFPLLPLVVSGRLRNLSFTPIELLTIVPGQRIKIQKMSATVVSFKFFRHRLFSNFQQSSMTGQNATLPREHTRRIMEILKYHLKIEGNRHLAAFGVNVTMEPIQMPAMILAPAQMTFGGASVVFPPPGSVQFRPRNGARFWRPARVNNVAVIGFDGVRIDLDHFCNRLHSTCQRNGLDMRNRPQEWIKLQMNSSDTARLKGEMLQLLREQVSIVIGITSEKKPDVHDVMKYFEAGIGLQTLQIHDRTAECFMREQGGAQTVDNVMRKLNLKLGGINFNVDPVNMYEHKVICNNIGGMKKKLFEKVQFVGFEMTHGSSRTLYDKAQGQFDGEPTIVGCGYSLSSPTELGGYNFLQERNEYKLKNLDSHFKTCLDQYKKATGSLPETVVIFRTGAGEGDFKRVQEEVEEMQGVCRRIGGSKPPKLVVVVVQKTSHTRIFPKEIMGHKAYEQNVKSGTMIDSRITNSGREEFILVSQSALIGTVRPVKYSVIANAPEWSKNEISNLAYVLAFGHQVSYQPPAVPHVLYAAENLAKRGRNNYLQHKKLGELSKSIKKVLDEHRDLLDAEESQLDSVLVADITASMNKMAVKNKNFWA

***Caenorhabditis remanei* Y49F6A.1**

MSFRIPKKVRTEDAAGAPSGAGGASGSGGSSNPPNRNNPPIRFTTPSSSGSSYAKTFTPIGKCEVQTNVFKVDITKLPFRLTRLSMETTLCGGKVDVNLTEGHQAVSGGVNDSERRLALHTVFLKLFQRHPDIFGTDILQYTYDCANTIYGFETAYKGGDAKLEDKIERKDFSDKEWEMISRILRRQSMFFKVILSANGFVYSDGPNALSNENRQELTRVVENCSSEVLNTTDYLQYGSQTFPMNEPPVFKPDATTEIRCGFDKAVRLAEGLAGTPEIVVTIDTKLSPFFSATSTLKFFCSKYAEFKGVSTGSQPGTSRGNRPGDRGGDRRGFGGRDPRADQRRRSRSRSPIRRDEKPVQDDWNYNEVKEIQSAYERGTEKNLLGRIEEALKGLLVVPIHLPKDKNRNIIVSRIAGSNAMNSIFELNKGKDDARNISVADYFKEKYNYQIKFPHLPLVVSGRLKNETFMPLELLNIVPGQRIKIQKMSANVQASMTGQNATLPRDHVLRIREILKGHLKIENNPHLKKFGIHVAREPIQMEAMMLSPAQMIFANRAIVVPPPGTVQFRQNKGQKYYKPAKINNVAVVNFDGAVTNLEIFSARLHATCLKNGLEMRKSHQEWLKYQWNSNDASYIKAEMVKMRDAQITIIVAITPEKKPDVHDVLKYFEASAGLQTIQIHINTADCFVRDAGGAQTVDNVMRKLNLKCGGINYLVEIPQSYDHKVVCSNVSFVQQKLFAKTQFVGFEMTHGPSRTLYDRVQGTFDGEPTVVGCSYSLKAVTDLGGFHFLQERNEYKLKNLDSKITVCLEQYKKSAGALPEVLLVYRTGAGEGDFKRVEEEVQDMRKALEKVGSKSKLVVVVVQKTSHTRIFPKEIKGNKPVEQNVKSGTCIDGQITSAGRQEFVLVCQSALIGTVRPVKYTIVANDPAWSKNEMAHLTYFLAFGHQVSYQPPAIPHVLYAADNLAKRGRNNFLQHKKLGLLTKSIKKVLAEHKDLAVEGHEAELDSLLVESITDAMNKMAVKNRSFWA

***Pristionchus pacificus* Y49F6A.1**

MDTKIKEEFDVDDLTKEVEKMTLPRRDESENVEGYGIKTRVFMNVFKLSTKDAPKTIYKYELTFTMTKANGDEFQLHNALQKVDEQQQMNNPDDVYGENAKSPQALQFFQVLAMQTLNRKEDIDVKPDAAYLTGGDFTKDHKQLRKGIVTSSRVMGDNICIQLDSKISLFFKAEPMLEYLRKFSGRMSESDLEKYLNDRVQSRKLRKELVNLPLRVRHLPSRKTFEFKGFLTDNAVNTTFKLKDDSIISVADYIEKQYKYKLKFPRLPLVIEKNRGGNSYHAIECLDIAEGTRVSNQKMSKEAQENMISKSCRAPVQLGKDLEESRRIAAFEGTRENKYLTAFNVKTTGEFACLNAKLLSKPKVTGMGNDPQINERGQISYTKGGFRFADPAKPTRPITLLSLGNAMRDEDCRLMKDTLKQFGSDFGMSIEFLNIRDPGIFGGQIEPLREFMASYKDKTSMFFFITKEKMDESHYMVKKLEQELGVVTQLVCAKTAGDFLRKMRTTQFNVMAKMNQKLGGVVANPEVPPDLKNTNPDAYARAVKDWFPNRMFVGLTLSHAGSQSFADRVVGEEVREPTCVGMAFTMRQPGKRSAMSWFQEKRKATIDDLTRHFIRALDIYAESNGGKMPTSILIFRKGMSEGELKKAAFEMHQVKEAIIAVSKRPGQEDYRPTVQCLVCMTNTSDRLFYQNGSENVPAGTCIDKEGCNPDRVEFLIVPHTAIKGTAKAVRCTLVTNDKGKVGRRLEYSEIQSVYHAMCYLNGVAASPTRLPVPLADSEKAAERQMNNFKESMRNGDETMSMCSGRSGSGLIHDGSKDYYKNLSDQFDHKFKDNQYYA

**ZK1248.7**

***Ancylostoma caninum* ZK1248.7**

RGTLVTHSKGTSGQLLSMDELEQITNILCYGHQVVCGSTGVPSVCYSATNLSKRGRNNWKTENFRDIGSESTASGASGERGRLVHDGSPNFFKDLSSHLSSKVNTHYWA

***Ascaris suum* ZK1248.7**

MTNTQPGTVVDHTVTRADVTEVFMQSHRGTGKLPAYTMPVNEANMTMEELQSMMMALCYEHQIVNAAISIPEPIFQADEWAKRGRNNFRAFX

***Haemonchus contortus* ZK1248.7**

GSARTPRYTVLVDDLNLSMDELEGMTYVLTYDHQIVNLPTSLPTPLYVANRYAERGRNTYANQFSCRQEHAVFENGKVFMSHPWNFGFHQQDCPSVGGGKRLFPGTQKSVRFIEGPGGRNYNNPALIIDGRPTEQNIPPGVVVDEGLTHPSFKEFYLNSHITLQLEPEAPIMAKNAPPGVLGSRYVVQTNAFGVQLEKPMAFWRYDVVISAEIGSAKRPVFFTKKGRDEYVVRRTQVVHDRRMVTMENILLKANLKLGGLNYEIDMNGQMPSEEFK*VLPVSWPTNGETSFLTVNTMSDIIRNVLPKFAQNHGNKFPRDLIIYRSGLSEGSFSTVILTHEIPLLRGALAALGAKNVKIVFIVAQKEHNVRLMLERVRYIIMNRNYKCKLVFDAVVRLYSDFFDDPDQLWYDGQSILFSGKDLFKNRAKARAWCAVPPGERQDNIARGARALKLLGSDDNPFVSNAGLYIYKDPIKVTMSLTEKELLTKTANSDLSQNDRSITQFMELVFNQDELKARLKEAAKHNCKYCLIVSADSITTAHSLFYAANFKANSYDFVGDYLFQSSRREEVGSLAVNINPDMLTSCLITF*TVNTMSDIIRNVLPKHSTQITVLAKKAAFHEELPLIEKAKHILNDNLLERVTEIGLERLNAGMKGRLFLGIGVSHPPPSQNFDERSVPSVVGSRFAEMFFKEASNRGIRLPHPSE

***Oesophagostomum dentatum* ZK1248.7**

FFSMDELEQITNILCYGHQVVCGATGVPSVCYAATNLSKRGRNNWKTENFRDIGSHSTASGGSGERGNLVHDGTPTYFKDLSAHLSSKVNTHYWA

**PRG-2**

***Haemonchus contortus* PRG-2**

QVTLVQKACEEVV*****LKENAKQPIKLAFIIVTKKVNMRIFKGAPDSKLS*NPDPGTVVDTVVTRPERYDFYLVPQFVNQGTVTPVCYNVIFDNTGLKPDHHQKASFMKNAMIVGYDLYHDSTLHGKTIGACVSTTDQDYTQFYSQTRPHENPTELGNNLGHFIKLAFKLCHLYYNWQGTVRVPAPCQYAHKLAFMVAQSIHREVNEELRSKLFFLCELFTVPSQCVQMGTLRGRQSDGGRNKNFGSIVLKIVLQMNCKMGGALWKVNIPVRDVFVVAEALRKYYVANNHSLPEKVFLYRDGVGDGQIHYVKDQEVRIQPIHMIVLILPDDNKARYDSLKSWLCSE

***Oesophagostomum dentatum* PRG-2**

SRELEPEPLYGNRRDGYTGDRAEWARYVKSNGTFRGEALTNWIVVTPYTDDGRYFAEQFIQEIGNTYDVLRIEHRLPMIEYCKNLTGEGYLEAIQTAISRVGKQPVHMMVVLIPDDTKSRYDMTKSFLCTKTNIPSQFVKLSTLRGSNRPGQRCRSKNFLSIVLKIAYQMNCKMGGALWKVKIPMKRGMIVGYDLYHDSTLQGKTMGACVSTMDQEYTKFYSQTQPHDSPTQLGTNLNIFILRAIQKYFKANDNTLPDKIFLYRDGVGDGQIRIVKEEEVRLVQQACADAVERARQVTDIPKDFKIKLAFIIVTKKVNMRIFKGSHDTPLTNPDPGTIVDSVVTRPERYDFYLVPQYVNQGTVTPVCYNIIYDDTGLPPDRHHMLAYKLCHLYYNWQGTVRVPAPCQYAHKLAFLTAQTIHRESNDTLRQKLFFL

**C06A1.4**

***Haemonchus contortus* C06A1.4**

KLLEVEYQIVSQEVKGSKVDSVMFKNQNQTLDNVIAKVNFHTHIPM*CFAERKMCLDQHRFE-TESGK ATVEQYFKKHYNIQLKYRYP—TLFTVSERHNPSTYYPVELLVVAPSQRVTLSATLPAVRIQQTKVMKD ALGITSGNAKLSSAGISVFLRVSKWLNDEARLFVGFEISNPPALSKMEIERGATYK---VGFLSNTTE CVSFRNFFLLTNCFQMMGATLGELIVEILKKFKAATSRA-PRHIVLYFSGISEG

**C14B1.7**

***Haemonchus contortus* C14B1.7**

CMNCTKFRNGRLFNGKMFVGFELSHAAAQSFYDRQVAEKVKEPTVVGVSITIITMKTVV*GTSRPTRYTIVVEDKPQMSLTDAEHITHFLCHGHQVQQSTLPTHVPAVLYAAENLAKRGRAAWKLQDHFRRAFHDYHKKNKRLPVEVMVYRSGTSEGEFAEVFDQIHIRIMPASMPPANGGRMRASDFNVPSGTCADTGITHPRLREFIMTSQQVGKEASDIRAVAERMSDLNGGRPYHPKITIIVAQTNSNYRIISIVALSARSKVTPVSNCGRIIIRDKVNCGNC

***NON-ARGONAUTE RISC PROTEINS***

**TSN-1**

***Ancylostoma caninum* TSN-1**

AGYAKCVDWSIGLCTGGAERLRAAEKQAKDKKLRLWRSYQPSAASALTGDKKSFTGKVVEIVMSDAMVVRKADGSEVKIHLASVRLPRDSDEKPSVGRQFRPLYDVPFMFQAREFLRKRLIGKNVSVTVDYIQPKSEQFPEKTCCTVKVGELNIAEALILKGLSKVVRHRSDDENRSSEYDALLAAEANAEKSKKGLFADKTADKKDTLRIQELQGDLARSKQFLPYLQRSTRAEGVVEFVASGSRLRVYIPKETVVITFLLGGINCPKSGRPGPGGITGPSEPFADEATAFTRRMVLQHEVEIEVEGLDKMGNFIGYLFATPEGGGKPQNLSELLLEQGLASLHFTAEKSGHYHQLAAAEQRAKAANRNIWQNYKEEEIVPDEVVAQQNDVAERKVQYKKVAVTDVSKGALTFAAQLVEDGAKLEKMTADFREYLRQHPPMTGAFNAKRGDVCAAPFSVDGLWYRAKVESVRSGQAEVLFIDYGNRETVSASSLAQLPPGFAHFPAGAKEYGIALVAIPNDKEYGLQCEDALQQLLFSVPTVEINVEYKIGSIEYCQVMMECAGKKVDVGKALIEDGFALVEKRKEKRLQSMLSEYESAEQKARRERKNIWEFGDFTGNDL

***Ascaris suum* TSN-1**

VTYVLKVRHITWVIENPRALVDLHKQKPISAVIEQVRDGSTVRAFLLPDFHYITLILSGVKQGIYSLFHPSSKVLTVNAKHYSLSFITWSLLLLFEAPATRAGPDGRAEDFAEEAKYFVECRILQRDVEVILEGVSNQNLVGSIVHPKGNIAEALLREGFAKCIDWSIALATSGPEPLRAAERIAKEKRVRLWRSYQPSNQLSADKRTFTAKVVEIVMGDALVVQKENGEEMKIWLSSVRPPX

***Brugia malayi* TSN-1**

MSEQQTQPQQASSLKRGLVKQVLCGDAIVLQGPPMNGPPKEVTVYLSNVIAPRLAKRPTDTESGKEDEPFAWESREFLRKKLIGQNVVFRCDYTATSGRDHGRIYLGGTNLENSENVTETCVSEGWVEVRLGRVTDEYSTKLLELQEVAKAAKKGKWALEEGNAQQHVRLKVRQVKWIIENPRALVDTLKQQKIKAIVEQVRDGSTIRAFLLPDFYYITLMLSGIKAPAIRAGADGRAEDYAEEARYFVECRLLQRDVEIILEGTSNQNFVGSVIHPKGNIAELLLKEGFAKCVDWSIALATSGPEVLRAAEKIAKEKRLRFWRAYQPPNQLDIDKKSXTAKVIEIVMGDALVVQKENGDEMKIWLSSVRPPREDNRDTENKXGRQFRPLYDIPYLFEAREFLRKRLVGKKVQVTIDYVQGKTEQFPEKICCTVMSGGLNVAEALVSKGLAKVIRYRSDDDNRSSQYDALLAAEAKAEKSKKGLFADKELGDKGPVLRIQELQGDAQRSKQFLPYLQRSGRSEGIVEFIASGSRVRLYVPKETCLITFLFSGIDCPRGARIGPGGKLIGENEPFAEEAAKFTRSKIMQREVEVEVEGMDKSGSFIGYMFVQTEQGLCNMSIALVENGLASVHFTAEKGAYYSQLCVAEEKAKKAKLGIWVKWVDEEAIVQAEIASADEKMERTINYRKVVVTDVQRGNFKFAAQSVDDGPKLEKMMKELREELRKKPPVVGAYVPRRGDLCVARFSADKLWYRARVEGIKGKSIDILYIDFGNREVVDVTSMAALPAGYATQPAGAREYQMAFLQMPNDVDHANNSNIAFEQILFSVPFMFINIEYRNGGIENVTAIIETSDGTRTDVAKTLIAEGHALTEQKREKRFASLIAEYQETEKIARREHRNIWEYGDFTGNEL

***Caenorhabditis brenneri* TSN-1**

MTDASSPTVPPPTTSTTPAVRRGFVKSVLSGDAVILQGKPENGPPPEWTVYLSNVSAPRLGRRPTETSPATPDEAFAWQAREYLRNKLVGQFVTFVRDFTASSGRDHGRIYIGGTSPADAENVTEGAVSEGLLEVRQGKIADEYTTKLLELQEQAKAASRGKWGSGAGTIREVRWVIDNPRELVDKYAQKPVDAIIEMVRDGSTVRAFLLPDFEYITLQLSGVRAPATRNPNASDSRAEPFSEEAKFFVESRLLQRDVQVILESTSNQNVVGSIIHPKGNIAESLLREGYAKCVDWSIGVVTGGAQKLRDAERQAKEKRVRLWKSYQPSSSGYSGDRKAFTAKVTEIVLSDAVVVQKDDGSELKLHLSSIRLPRETGDDKAGGVGRQFRPLYDVPFMFQAREFLRKRCLGKKVQVQIDYVQPKAENYPEKTCATIKIGDLNLAEGLVSRGFSKVVRHRADDENRSCEYDVLLASEAAAEKSKKGLFADKTAEKKDTLRIQEITGDLAKAKQFLPYLQRGGKAEGVVEFLSGGSRLRIYIPKETVLITFLLGGINCPKGARVGPGGVTLGAAEPFADEAAAFTRKLVLQHEVQLEVESTDKNGNFVGYLFVSPDGNASRATNLSEALLENGLATLHFTAERSGHYNALLAAENRAKKAKKNIWANYTEEQQQEEVEVQQADTSERKQNYRQVAVTDVAPGALRFSAQNIEDGPKIEKMTAEMRQAISEHPPLAGSFSAKRGDLCVAKFSQDGQWYRAKVESIRAGQAEIVYIDYGNRETVDAAKLAQIPAGFASFPAGVKEYHLALVKLPNEDYVQLTLDALTQDLFGHSSVFINTEYKVGTADYVTVYYDSGNKKVDIGKSLIAEGLALADQRREQRLQTISAEYKSAEDTARKSRKNIWEYGDFTGNDI

***Caenorhabditis briggsae* TSN-1**

MSDAAATASPIAPPPASSTTPAVRRGLVKSVLSGDAVILQGQPQNGPPPEWTVYLSNVSAPRLGRRPTDSAPATPDEPYAWEAREFLRGKLVGQFVTFVRDFTATSGRDHGRVYLGGTSPADAENVAEEAVAAGLLEVRQGKITDDYTTKLLELQEQAKASGKGKWSSTPGTIREIRWVIDNPRELVDKYAQKPIDAVIEMVRDGSTVRAFLLPNYEYITLQLSGVRAPSTKNPTSHDSRAEPFSEEAKFFVESRLLQRDVQIILESTSNQNFVGSIIHPKGNIAESLLREGYAKCVDWSIGLCTGGAQKLRDAERQAKEKRVRLWKSYTPAASGYSGDRKAFSAKVVEVVLNDAVVVQKEDGTEMKLHLSSVRLPKETAEDKQPSVGRQFRPLYDVPFMFQAREFLRKRIHGKKVQVQIDYVQPKTDNFPEKTCATIKHGDLNIAEGLISRGLSKVVRHRADDENRSCEYDLLLAAEANAEKGKKGLFADKTAEKKDTLRIQEIAGDVAKSKQFLPYLQRGGRAEGVVEFLSGGSRLRIYIPKETVLITFLLGGINCPKGSRVGPGGVTIGAAEPFADEAAAFTRKLVLQHEVQLEVESTDKNGNFVGYLFVSPDGNTSRGINLSEALVEAGLATLHFTAERSGHYNALLAAENRAKKAKKNIWANYTEEQQQEEVEVQQADTSERKQNYRQVAVTDLAPGALRFSAQNIEDGAKIEKMTSEMRQAIADHPPLAGSYAAKRGDLCVAKFSEDGLWYRAKVESVRQGQAEIVYIDYGNRETVEAAKLAQIPGGFASFPAGVKEYNLALVKLPNEDYVQLTLDAFAQYLYGHSSVFVNSEYKVGTAEYVTVYFDMGNKKVDIGKSLVEEGLALADSRREPRLQTLCNEYKSAEDKARKSRKNIWEYGDFTGNDI

***Caenorhabditis japonica* TSN-1**

MTDAASPITAPAPPPPAASSSTTSQVRRGLVKSVLSGDAIVLQGQPQNGPPPEFTVYLSNVTAPRLGRRPTDSSAATPDEPYAWESREFLRKKIVGQFVTFVRDFTATSGRDHGHVYLGGTSPADAENVAETSVAAGFLEVRQGKVTEEYTSKLLELQEQAKSAGKGKWSTSAGTIREVRWVIENPREIVDKYNQKPVDAVVEMVRDGSTVRAFLLPDFEYITLQLSGVRAPSTRNPTSSESRAEPFSEEAKFFTESRLLQRDVQIVLESTSNQNFVGSVLHPKGNIAESLLREGYAKCVDWSIGLCTGGAEKLRAAERQAKEKRLRLWKGYQPAAAGYTGDRKAFTAKVTEVVLSDAVVVQKDDGTELKLHLSSIRLPRESGDDKTPAVGRQFRPLYDIPFMFQAREFLRKRILGKKVQIQIDYVQPKSENFPEKTCATIKVGDLNIAEGLVSRGLSKVVRHRADDENRSSEYDTLLAAEANAEKGKKGLFADKSAEKKDTLRIQEIAGDIAKSKQFLPYLQRGGRAEGVVEFISGGSRLRIYIPKETVLITFLLGGINCPKGARVGPGGVTLGASEPFADEAAAFTRKLILQHEVQLEVESTDKNGNFVGYLFVSPDGNTARGINLSEALVENGLASLHFTAERSGHYNALLAAENKAKKAKKNIWANFVEEQQQEEVEVQQADTSERKQNLRQVAVTDLAPGSLRFYAQNIEDGAKIEKMTSEMRQTLSENPPIAGSFTARRGDLCIAKFSQDGQWYRAKVESIRAGQAEVFYIDYGNRETIEAAKLAQIPGGFASVPAGAKEYNLALVKLPNEDYVQLTFDAFAQYLFGHASIFVNTEYRVGTADYVTAYYDNGTKKVDIGRALVAEGLALSDSRREPRLQTLVSDYKSAEETAKKSRKNIWEFGDFTGNEI

***Caenorhabditis remanei* TSN-1**

MTDTGSPTVPPPASSTTSPVRRGLVKSVLSGDAVILQGQPQNGPPPEWTVYLSNVSAPRLGRRPTDSSSATPDEPYAWESREHLRKKIVGQFVTFVRDFTASSGRDHGRLYLGGTSPADAENVTKEMVSEGLLEVRQGKITDEYTTELLELQEQAKSAGRGKWSSNAGTIRDIRWAIDNPRELVDKYAQKPVDAVIEMVRDGSTVRAFLLPNFEYITLQLSGVRAPSTKNPTAPDSRAEPFSEEAKFFVESRLLQRDVQIILESTSNQNFVGSIVHPKGNIAESLLREGYAKCVDWSIGLATGGAQKLRDAEKQAKEKRLRLWKSYQPTSSAYSGDRKAFTAKVTEVILSDAVVVQKEDGSELKLHLSSIRLPRETGDDKQPSVGRQFRPLYDVPFMFQAREFLRKRILGKKVQVQIDYVQPKSDTFPEKTCATIKIGDLNIAEGLVSRGLSKVVRHRADDENRACEYDTLLAAEANAEKGKKGLFADKTAEKKDTLRIQEITGDLAKAKQFLPYFQKGGRAEGVVEFLSGGSRLRIYIPKETVLITFLLGGINCPKGARVGPGGVTMGAAEPFADEAAAFTRKLVLQHEVQLEVESTDKNGNFVGYLFVSPDGNTSRAINLSEALVEAGLASLHFTAERSGHYNALLAAENRAKKAKKNIWANYTEEQQQEEVEVQQADTSERKQNFRQVAVTDIAPGALRFSAQNIEDGAKIEKMTTEMRQAIAEHPPLAGSYTPKRGDLCVAKFSQDGQWYRAKVESVRAGQAEILYIDYGNRESVEAAKLAQIPAGFGSQPAGVKEYNLALVKLPNEDYLELTLQAFAHYLFGQSSVFVNSEYKVGTSDYVTVYFDSGNKKIDIGKALIEEGLALADERREPRLQTIVKDYKSTEAAAKKGRKNIWEYGDFTGNDI

***Meloidogyne hapla* TSN-1**

MVEGPVKPVLENSMKRGFVKQVLSGDSIVLQFSVAPGSPPNETTVYLCNVVAPRLAKRPNENSAATPDEPYAWEAREFIRKKLVGQTVTFVREFTATSGREHGHIYIGGTGIEDGESITESGVSAGLLEVRPGKQIDESAKKLLELQEQAKEAKRGRWSGDEPTKHVRTIQWTFDDPRSLITKYCGKPVDAVIEQVRDGSTVRAFLLPSFDYVTICLSGVKTPGVRVGTEGKPEEFGEEAKFFVEIRLLQRDVKVILESTSNQNFIGSVLHPRGNIAEFLLRDGLAKCVDWTMGLVTGGGEKLREAEIFAKQRRLRIWKNFNAQTTTAPSKNGFSSKVVEIGLCDSLSVLKDNGEEVKIYFSSLRPPRREGADGTVPIQTAVRTQPRPLYDIPFMFEAREYLRKRLIDKRVNVMIDYIQPKQNQFPEKTCCTVLFNGQNVAVMLIERGYAKVVRHRSDDDNRSQQYDQLIAAETKAESEKKGLWADKSDQPTTLRVQELQGDAQRSKQFLPYLQRSQRIDGLVEFVASASRLRVYVAKESCLITFLLSGINAPRTARIGPNGQQIGSDEPFAVQAMNFTRYKCLQHDVLFFFKLFASFLFQVQIEVETMDKAGGFIGYLFVQVERGVWKNLSELLVENGLASVHFTAERSSYYTLLMNAERTAKAAKLGIWKHHVEEEQQLVDDNQKQGNDTTERKLDLKKILPTEVYSGFRFAAQTFDDGLSIEKLMNALQLELKTPSIVKFVPKRNQLCAAKYTDGNLWHRARVEGVKGDNVDVLYIDFGNRETLPTSRIAPLPPQFQSQAPFAKEYQLALVSAPPDPSYADDSLLTFKRLCFSKPYLYLNVEYRINGLEAVTVFADDSKGEKRDLAKKLIADGYALVEKRREARFQQLLDDYVEQETKARKAHMNIWRYGDFTGSEL

***Meloidogyne incognita* TSN-1**

MVEGPVKPVLENNMKRGFVKQVLSGDSVVLQFSVAPGSPPNETTVYLCNVVAPRLAKRPTENTAATPDEPYAWEAREFIRKKLVGQTVTFVREFTATSGREHGHIYIGGTGLESAENVTESGVSAGLLEVRPGKQIDESTKKLLELQEQAKEAKKGRWSGDEPTKHVRSIQWTFDDPRSLVTKYGGKPVDAVRDGSTVRAFLLPSFDYVTIGFSGVKTPGVRVGTEGKPEEFGEEAKFFVEIRLLQRDVKVVLESTSNQNFIGSVLHPRGNIAEFLLKDGLAKCLDWTMGLVTGGGEKLREAENFAKQRRLRIWREGADGTLPIQTAVRTQPRPLYDVPFMFEAREYLRKRLIDKRVTVMIDYVQPKQNQFPEKTCCTILFNGQNVAIMMIERGYAKVIRHRNDDDNRSQQYDQLIAAETKAEGEKKGLWADKTDQSNTLRVQELQGDAQRSKQFLPYLQRSQRIDGLVEFVASASRLRVYVPKESCLITFLLSGINAPRTARIGPNGQQIGSDEPFAVQAMNFTRYKCLQHDVQIEVETMDKAGGFIGYLFVQVERGVWKNLSELLVENGLASVHFTAERSSYYTLLTNAERIAKAAKLGIWKHHVEEEQQLIDDNQKQGNDTTERKLELKKILLTEVYTGFRFAAQTFDDGLSIEKLMNALQLELKTPSIIKFIPKRNQLCAAKYTDGNLWHRARVEGVKGDNVDVLYIDFGNRETLSMSRIAPLPPQFQSQAPFAKEYQLALVSAPPDPSYAEDSMLAFKRLCFSKPYLYLNVEYRIGGLEAVTVFADDNKGEKRDLAKKLITDGYALVEKRREARFQQLVDDYVEQETKARKAHLNIWRYGDFTGSEL

***Oesophagostomum dentatum* TSN-1**

TSNQNLVGSVIHPKGNIAESLLREGYAKCVDWSIGLCTGGAERLRAAEKQAKEKKLRLWRSYQPSAASALTGDKKSFTAKVVEIVMSDAMVVRKADGTEMKIHLASVRLPRDSDEKPSVGRQFRPLYDIPFMFQAREFLRKRLIGKNVNVTVDYIQPKSEQFPEKTCCTVKIGELNIAEALILKGLSKVVRHRSDDENRSSEYDALLAAEANAEKSKKGLFADKTADKKDTLRIQELQGDLARSKQFLPYLQRSTRAEGVVEFIASGSRLRIYIPKETVVITFLLGGINCPKSGRPGPGGVSGPSEPFADEATAFTRRMVLQHEIEIEVEGLDKMGNFIGYLFLTPEGGGKPQNLSEMLLEQGLASLHFTAEKSVYYHQLAAAEQRAKAGKRNIWQNYKEEEVVPDEVVAQQNDVSERKVQYKKVAITDVSKGTLNFAAQLVDDGAKLEKMTADFREYLRQHPPMTGAFNPKRGDLCAAPFSVDGLWYRAKVESVRSGQAEVLFIDYGNRETVATSSLAQLPPGFASFPAGAKEYGLAMVAIPNDKEYGLQCEDALQQMLFSVPTAEINVEYKIGTTEYCQVVIECAGKKVDVGKALIEDGFALAEKRKEKRLQSMLSEYEHAEQKARRERKNIWEFGDFTGNDL

***Pristionchus pacificus* TSN-1**

MASEGVSMRRGLVKQVLSGDSVVIQGAPRPNGPPEETTVYLSNVSAPRMGKRPTETVTATPDEPFAWQAREWLRKKLVGQMVTFIKEFTATSSRDHGKIYLGGTSVETAENVNETAVAEGWLEVRQGKVTDEFTSKLLDLQEKAKSAKIGRWSDNTDGAVRDIKWTFNDPRSLVDFYKQKPVDAVIEQVRDGSTVRAFLLPRFEYITLQLSGVRSPSTRAGNDGVEAFANEAKFFAEARVLQQDVQIVLESVSNNNFVGSILHPKGNIAEALVREGLAKCVDWSIGLCTGGAEGLRAAENQAKMNRKRMWATYKPSAGNLAEKKKFDAKVIEIVLNDAMIVVRDNGEESKVYLSSVRLPRDAGDRPTAAGRQFRPLYDIPYMYECREYLRKKLIGKKVSVTVDYHQPKSDQFPEKTCCTIEAGGANVAIGLLERGLSKVVRHRNDDENRSSQYDALLAAEAKAEKEQKGLFAVASKEEGNHTQRVQELQGDLARSKQFLPYLQRGARAEGIVEFLTSGSRMRIYVPKETCLLTFLLGGINCPKGARAGANGQTAAAAEPFSEEAAKFTRKLCMHREVELEVEGLDKQGAFIGYLWVRPEDGGRHQNLSELLLEQGLATLHFTAEKSAHYNHMSAAETRAKNAKRNIWATWTDTDAEAKEEEAANQKTERTVNYKKVAVSDVGKNGNSFRISAQSIEDGPKLERLMEELRVSVANSAAPSNVTVKRGEMVAAKFSADKQWYRAKVESVRAGQADVLYVDFGNRETISTSDIAALPGTLSSVPASARDYYLALVSTPNDDDYTGFALTALQTLLSSNSFAEINVEYSLGGAQYAQVLVEINGEKVDVGKALIEDGFAMADKRREQRLQKLVSAYDEAEKKARKERANIWEFGDFTGNEI

***Trichinella spiralis* TSN-1**

MYPNNVSSQTPQPAPVSSPPLMRGIGKMALGGDSIVIRGQPKGGPPPERLINLSNVISPKLARRQADSTATDSQDEPYAWEAREALRKLVVGHELLFTVDYKVPTSGREYGSVFVTIDGKRQNVAETLVSQGWLEVRQSGVKSNDDAVKRLLELQNTAKANSKGKWQADDATKHVRQIIWSTANPRSLVESFNRSRIKAVIEHVRDGCTVRAFLLPSFHYVTIMISGIRTPTFKLGEGGMIQDPEPFAEEAKFFTECRLLQNDVEVILEGASNQNFLGTVLHKHGNIAEALLKEGFAKCVDWSMPLVTSGPEKLREAERQAKERRLRLWKNYEPSHAKAAGENSFQAKVVEITLGDSMIIKKQDGMYQKIFLSSVRPPRLEDAGLVRETQSGRQFRPLYDIPFMFAAREVLRKKLIGKKVNVTIDYVQPSVNQLPERTCCTVVFGGQNMAELLVSKGLATVVRNRQGDENRSPFYDNLLTAEAAAEKARLGIHSLKHSVDANHMETANIYNTSEKQIVRLQELQGNVAKSKQFLPFLIRSGRTDGLVEFVVTGSRLRIFVPKESIMITLLLGGVSCPRPGRMTKGGGAAEAEDEPFSQEALQFTKDFCLQREVEFEVESVDKAGNFIGWCFFHGKNLSELLVENGLAAVHFTADRSKYGPALRAAEMRAKEAKLKIWTLAYYDDEAEELNDADDLEKGPSATPSAGIVPERVPQYKAVLVTEICENLKFYIQYFDQGSQLEQMMKEMRTALNADPGRQGAFVPKKGDVCAALFSADQQWYRARVEAVRKDEIDVFYIDFGNREARKQNELASLPAGFASRPPGARECAFALLKLPDDADYCTAAVKHFYKEVNGEQCLMNMEYRLGGVEYVTLLRADQSDIGKSLIRNGYCLVEQRRDRKMQLQLADYLQAQESAKSERVCK

**AIN-1**

***Ancylostoma caninum* AIN-1**

MAATSDAPNAPPPQHGAHGQPPRMFVQRPPQVWAPNGGMMGGPPVGKQAMPWDQQRMTGMPRGGMGRGGPAGGRYPPGGMDMSVPPPIDLPLSGMSGMRQMAPPNAAGTWKPEIGVAPNGIRGPTAFGQMAAPLIMSGKPQAAPNQFPIGGVDDLMWHDPNGDLKKWQRDTGVSLWGDPEKNNERPVRLWMIGEGEEEDLEVALMKCPVPQKKNEDGSARLPFPIPSKRPIVVTGWGELPENDPNNPIKHDEAQPGSGKWGDLPSHSQHASDSPWFLPSQQQPQPFAPEAPAWGQPNTAAAPMPPGPSQPHTQAVADQLKYAVDKGYLDMSVLQQTNLPPHVLQHMNQMLALIPALECCEAELKMLVDSVRPDGEVDGNSPQRWMNDVQKVEYNRLIIEVTTVKIEVAELSKKIQRGLADAGMSSGSATVEGLGSDAYHYSFLE

***Ascaris suum* AIN-1**

MLVNHGTKYGGVAQWAPGSSMAAPGPVKQPFTHVYSSRSSPPMSVGTFNAVPAHNTNSFMGEDSMWQDPNGDVRKWQRDTGTAIWGDPDKQPRYNVESVFTTNERAFAAEGCN

***Brugia malayi* AIN-1**

MWAPVDSTQAETASLNSWSIEQQQNRWGGVSDYSNNAAFDEHMRSIVPPAFRNSNAKVVWQEPKSLEANINEALGIWQATPLNQFSLEQEPLSGTSHFPPSFASCGTTSGWSQPEIEQRNWDMSTQSQTVRAAVPFCGGLTQNKLWPNEEQVWGGAQRPPVFPTAPPPPAYAASWQQNAPRGNVFPLPTRFSGPPFESNAVMGNWVQRPMNEPVQPPVPPSAIPPPQRSRLQNVWGGTPAVNNTVNGMYLPPPTVEYTMPNGLTQWNPSAGSNGATVTAVPVVPKPQFAQNFVNRSPPPNTFNSYSQVSKSDSFMGENAVWQDPEGEVRKWQRDTGTAAWGDPEKQQKEIKRWIIPVGKEYDNSDNESGVRVIVPLGWGDLPSKSSNNNAAPISANSLPAWPARSAQAIAVTASSGWGNRSASGTAKSTTIPTLWEILSEHQPPSLESGWIPPDGMGNATMPASTQQIVEQLRQAVAKGLIDVSLLSKPLPNEALSEMRTLLSRIPVLEQAENDLARLMNAVKINNDPEGGNGRMDTPTDLMTSEQRSEYSRLVIEIAATKTEISTIRERIRGNSDAIGVRPATNLAESQRMHPPQPQPAATTTTSPRFGDSILDVAVRVEQQKLFSM

***Caenorhabditis brenneri* AIN-1**

MESLERSLNNISIDDAPWRPHDQQQVWGANGIQQPQQQQPRVWAPSVQPFPGRSPWQQDSTDNSEQNVNDALGLSGTAPAQSQWGGMPNSEYDKQIWADPLSDVQYPPPPPHAPFGAINGLGGPSADWGSQPGHQVWSNGGMGKESDDFWKQQPQQQQPYPMPLIQGGGWNPRGMNHGPPGQGQRGGPMMQQQDYQGGWGGPNNMGGNKQMNRPWGQDNNRGMSGMQQRNGRPNHNQGRYSGNMGGGVDVSVPPPMDMHMGGGMGGMRNMGAPNGGHGSWKNNNGGGGHGGQSGRGNYNNRGGMGHGNQGGGNMWNTGGNGGNGGNMQQYMGGDQSYNNSSFAIGGASDDLTLSVWHDPNGELKKWQRDTGVSYWGDPEKQSKCYYCRPINLWLVPEGEDEDLETALNRCPVPQKKGEDNQRFPFPIPPKRPIVVTGWGELPENDPNNPNKTESSIFEENNRWNDLPTEQNPWYLPNSVNYSNNSTTGSWVQGGTIPINEPTANQQQMTENVKKAVDKGYLDQSVTMLANLPPMVLQYVNALIQKVPGLESVEEELKQLVEGSRPDENTEVDPQNPQKFMNDSQKLEHNRLIIAVTTAKIEVQEFSKKINRALVEAGIVPSQDQQPRPQATSEDYHYSFLE

***Caenorhabditis briggsae* AIN-1**

MLYCSPTCQTTLINHCISFLRLLNSIQNHSPSFIDNHFFYFQLTSNSDRQSAINATMESLERSLNNISIDDSSWRPHDQQQPQQSVWSNGGVPHQPGRGPGGGAVWAPPVPQFQNRPPWQQEPNDMEQNVNETLGLGGMPPSQTPQWGGMPNSEYDNKQIWADPASDVQYPPPPAHAPFGALNGIGGPNGDWSMGSQPNHQPWSNGGMGKESDEFWKNQPPHQQPQYPMPLLQGAWNPRGMNHGPPGQGSRGGPMMQQQDYQSGWGGPNNMGGNKQMNRQWGHDNNRGMQMGQRNGRPNHNQGRYNANIDVSVPPPMDMHGGMGGNMRNMGGPPNGNHGWKNTGGMGGGGGGGGGGRYGGNNYNNRGGGNMGHQGGNQGGNNMWNNGGGGNGGNQGSMQQYGMGDQSGSQFSVGGAADDLTLSVWHDPNGELKKWQRDTGVSYWGDPEKQNDKTINLWLVAEGADEDLETALNRCPVPQKKEHGGEDTNQRFPFPIPAKRPIVVTGWGELPENDPNNPNKTESNIFEETNRWNDVATEQNPWYLPNHNANNFSADNTTGSWVQGGTIPINEPGNHINIPEMLKNAVAKGYLDQCVPMLANLPQTVLKYVNMLLVKIPALESVEDELKQIVESSRPDDSTEIDPQNQQKYMNDSQKLEHNRLIIEVTTARIEVQDYSKKVNRALIEAGIVHPQEQRAPAAAATSEEYHYSFLE

***Caenorhabditis japonica* AIN-1**

MESLERSLNNITIDDGPWRPHDQQPVWGNNQQQQQQQQQQQQRVWAPPVQFPNRSPWQQDTNDIEQNVNEALGLSGNTSSDNQWGPPIPSSDYEKQIWADPLSDVQYPPPPHAPFGTLNGIGGPPSEWSLGSQPSHPVWSNGGMGKDTDDFWKQQQQPQQQQQQHYPMPLLQGAWAPRGGMNHGPPGQGQRGPMMGGQQDYQGGWGGPNNMGNKPMNRPWGDNNRGMSGMAQRNGRQNQGGQGRYSSQMGVDVSVPPPMDMHMGGMGMRNMQNGGHQWKNNQHNNGGGGGGGGGNRNYNNRSAMSHAGSQSQGSMWNGTQSNMSYMSEQSYNNSSFTIGGASDDLTLSVWHDPNGELKKWQRDTGVSYWGDPEKQSDRTINLWIVAEGADEDLETALSRCPVPQKKVGETDENQRFPFPVPPKRPIVVTGWGELPEDDPNNPSKSESTIFDDNLRWSNLTTETNPWYLPNHAASTFNSDNTGSWVQGGTIPLSEPGSNQQNTVAAMLKSAVEKGYLDQSVTMLANLPPMVVQYVNLLLVKIPALDSVENELKQILESSRPDESVEIDPQNPQKYMNDSQKLEHNRLIIEVTTAKIEVQEYSKKVNRALLEAGILPSQEPPRQQQQTSEDYHYSFLE

***Caenorhabditis remanei* AIN-1**

MESLERSLNNVSIDDAPWRPHEQQPLWSNNGIQQQQQPRAWAPPVQPQFANRSPWQQDSNEIEQNVNEALGLSGMAANPPQWGGMPNSEYDKQIWADPTSDVQYPPPPPHAPFGALNGLGGPSADWSMGSQPHNPVWSNGGMGKESDEFWKQQQPQQQQHYPMPLLQGAWNPRGMNHGPPGQGPRGGPMMQQQDYQWGGPNNMGGNKQMNRPWGQDNNRGMSGMPPRNGRPNHNQGRYSGNMQGGVDVSVPPPMDMHMGGGMGGMRNMGAPNGGHGSWKNNNSGGGNGGHGGQRNYNNRGGMGHNGGGNQGGNNMWNNAGGNGGGNGGNMQQYMGDQSYNNASSFPIGGASDDLTLSVWHDPNGELKKWQRDTGVSYWGDPEKQTERTINLWIVAEGADEDLESALNRCPVPQKKGEDNQRFPFPVPAKRPIVVTGWGELPENDPNNPTKSESTIFDENNRWNDVPTEQNPWYLPNHNATTFSSDNTTGSWVQGGTIPLNEPGSNQVAVAEMLKNAVDKGYLDQSVPMLANLPPTVLQYVNMLLVKIPALESVDNELKQIVDASKPDDTTEIDPQNPSKYMNDSQKLEYNRLIIEVTTAKIEVQDYSKKVNRALVEAGIVQPQEQRAPAAVPTSEEYHYSFLE

***Haemonchus contortus* AIN-1**

VDDL-MWHDPNG-DLKKWQ--RDTGVSLWGDPE-KNSMLINFYLLVEDERTVRLWAIGEAEEEDLEVS LMKCPVPQKKKRPIVVTGWGELPIPIVQGYLDMSVLQQMNLPPHVLQHMNKMLALIPALECCEAELKMLEYNRLIIEVTTVKIEVAELSKKIQRGLADAGM

***Meloidogyne hapla* AIN-1**

MNPSYFGATTLGGNPSALPPAPIDLTTSWKPIGHNSNDFTPIYNDSSTGGGGAGIGSKLITIWGHPMDYNDGGHADPAGSSTYAAAAANLQGYPNNGFCWNDSQQPPSAFHQIPPQYDQMPFQQPEAKFAAGIHEQSFHPQQQPHPPPQSNRYNRNSKWDQQATLRDDSSAFGNNLYATTPPYGMVHTFGGVGDNRLIHSNGQRGSTGPSQRWGDSMGSGGGSTIPGQIGKFGGCEVITVLPHTNAGPPPHHPSHQQHRGPPQMRGGNGTLFTAGPPPLHIAPPPSSRPLPSHQSHLIGGHMGGPPPAPTPLLPSLNQHSTHSIGTFGGGGSGVPLHIQGNIPPNGPNSNMQTGQIDDAYWHWQDPNSQLRKWKHDTGIAIWGDPIKQANTPIKRWTNFTSDEVVAEVVPGAVSGWGDLPPIGSSVNNNTNSNTNNGNADNNNHTIDGNVNNNVPQHFGNQGLAPNNQQQTNRSNSLSAATRGDWSTGNVILPQQHNQQQQSFNQNGLVSGGNGQFFAGMPPTSSETPVSTVGQSSIYQPPPSLLSSMMIDPLSGHTNSDTDQQSTGGNGSGESTSRKATVSPQDGNNSFGGGVRQPQQNTQRQANKKVSLADELSSLIVSNNHGLGGGSLW

***Meloidogyne incognita* AIN-1**

MNPSYFGATTLGGNPSALPPASMDMTSWKPIGHNSTDFTPIYNDSTAGGGPGIGSKLITIWGHPMDYNDTGHADPSASSTYAAAAANLPNYPNNGFCWNDSQQPASAFHQIPPQYDQMPFQQQETKFTAGSQWGKTEIDQTIPWNLENNIFMTSHSILSNNLIHLLNRIDIIVIANGISRHLFETMVLLLVIIFMRQHLLMEWCILLVVLVTIIPSNGQRGNAGPSQRWGDTIGSGGGSTIPNQIGKFGGCEVISVLPHTNAGPPPHHPPQHRGPPQIRGSNGAIFTAGPPPLHIAPPSNRPLPNHPSHLIGGHMGGPPPAPTPLLPSLNQQPTHSIGTFGNGNNGVPLHIQGNIPPNGPNNNIQTGQIDDAYWHWQDPNSQLRKWKHDTGIAIWGDPIKQANMPIKRWTNYTSDEVVTEVMPGVESTFNQGCRQNHPMGWGDLPPIGTNVNNINSSNNNNGTTDNSNHASENNANNNVLQNFGNHGQAPNNQQQTNRSNSLSAATRGDWSTGNVILPQQHNQQQQSFNQNGMISGGNGQFFSGVPPTSSETPVSTVGQSSIFQPPPPSLLSSMMIDPLSGHASSDIDQQSTGGNGSGESTSRKATVSPPDGSNSFGGGVRQPPQQTTHRQANKKVSLADELSSLVVSNNHGLGGGIW

***Oesophagostomum dentatum* AIN-1**

MMGGPPVGKQAMPWDQQRMTGMPRSGMGRGGPAGGRYPPGGMDMSVPPPIDLPLSGMSGMRPMAPPNAAGTWKPEIGVAPNGIRGPTAFGQMAAPLIMSGKPQAAPNQFPIGGVDDLMWHDPNGDLKKWQRDTGVSLWGDPEKNNERPVRLWMIGEGEEEDLEVALMKCPVPQKKNEDGSARLPFPIPAKRPIVVTGWGELPENDPNNPIKHDEAQPGSGKWGDLPSHSQHTSDSPWFLPSQQQPQPFAPEAPAWGQPNTAAAPMPPGPSQPHTQIVADQLKYAVDKGYLDMSVLQQTNLPPLVLQHMNQML

**VIG-1**

***Ancylostoma caninum* VIG-1**

GGPRGGGRGRGAPFRGRGGNANSFVDSPKDTQNNFDELPHDSPALEFQGERRGRGGGRGGFXGERRGRGFRGGRQFDRQSGSDRTGVRGVDKKDGHGRGNWGDDKDELVGETEPLAISEEPEAPREKTAEELAQEALEAELAKQKTLKEYIEAQKKEAPKFNVRKAGEGEEENFGKLVKLQKEVVVDKEEEEVSIIRREPREKALHIDIQFAEPNRGYRGDRPPRGRGGPRGGGRGGRGGNRAQAPPQFDSSLDAFPALGSK

***Ascaris suum* VIG-1**

CGNSGFGGGYRGGYRGGRGGYRGGDGERLSFRGGRGGRGRQFERISGSDRAGVKPIEKKEGYGRGNWGTDQDELTGETEQLNAAQEGAFCFIFASQSGCS

***Brugia malayi* VIG-1**

MVEYGVNVTNKFGFLSXDEVDDPEVIFRKAEALSKKEEKTASQKKADKNAAAKKKKEITSGAMITAVAKEIEVKKQPQLSVAGMKKNDFNKENKENRPDGERVRGRGRGRGSMSRGRGFVQQFDNTRPTFDQILFLYLYTIWFNHNSVQNDERDVMAERFGEGRGRGRGRGNRGRPTFRGGRGGVRNFDGDQDHNMIDLQPELNGNGNIDQFGCERQNFTDFGMYRGSYRGGRGGFRGSDRDGERERPVFRGGRGGRGGRQFERMSGSDRTGIKALDRKDGFGKGNWGTDQDEMNGQNEQMNDGTEVVMKDEDSAPREKTQEELRLEAEAEARAKQLTLDEFKAQIASKRSEPHFNIRQAGEGTNDKDFGKLVPLNKPIMEENSEEEIVVVRREPRTKRLDIEINFTDEQRGGRGGRIRDGFRGGRGSRAGRESRNPKQVQTFEVSADAFPALGSQ

***Caenorhabditis brenneri* VIG-1**

MTEYGCQVTNKFGLPSDDDDEYDDPRELIQKVSQIAAKKKEEKSVKPVQPAKPAAAPANVVTKTDSAGRGRGGRGRGRGGAERRPRDGENRVSNENGDRSGENRRGGPRRGGERGAARPSGRGGRGGFTRENRETTEPEVADEHIANDGVDTRAPRRRGGFTLGGGPTGGRGGGRGGRGRQFDRQSGSDRTGVRAFEKKDGHGKGNWGDQKDELAGETENIAPETENIEPEVPREKTAEELAFEAEQAVLAKQKTLKEFRAAQNADAPKFNTRKAGEGAADNFGKLVPMKKEVIPDREEDEVVVIHKASRKQLLDISITFRNDRPEREQRHDRDRGDRERSDRPQRGGPRGGGRGGRGGPRTGGQGGRHQATPFNASEDAFPALGAK

***Caenorhabditis briggsae* VIG-1**

MTEYGCQVTNKFGLPSDDDSEYDDPRELMQKVSQLALKKKEEKAVKPAQPTKPVAAAPASAAAKTDGASAGRGRGGRGRGRGGAAGRPRDGGDRVSNENNGERSGDSRRGGPRRGGERGAARPAGRGGRGGFTRENREGEEPRDQHFADDGQDTRAPRRRGGFTLGGPSGGRGGSRGGRGRQFDRQSGSDRTGVRAFEKKDGHGKGNWGDQKDELAGETENIAPEGETAPAEPEVPREKTAEELAFEAEQEVLAKQKTLKEFRAAQNAEAPKFNTRKAGEGAADNFGKLIPMKKEVIPDREEDEVVVIHKAPRKQVLDISITFRNDRPERERNDRDHRRNDDRSERPQRGGQRGGRGGRGGPRSGGQGGRHQATPFNASEDAFPALGAK

***Caenorhabditis japonica* VIG-1**

MTEYGCAVTNKFGLPSDDDDEYDDPRELMQKVSLIAAKKKEEKVVKPAQPAKEAAPASAVTKTDGAGRGRGGRGRGRGGAGRPRDGDRVSNDQANRSGEDRRGGPRRGGERGAGRPAGRGRGGFNRENRDANNSGEEVKENTYEDGQDTRAPRRRGGFTLGGPTGGRGGRGGRGRQFDRQSGSDRAGVRAFEKKDGHGKGNWGDQKDELTGETENIAPEAENAEPEAPREKTAEELAWEAEQAVFAKQKTLKEFKAQQNADAPKFNTRKAGEGVNDTFGKLIPMKKEVIPDREEDEVVVIHKAPRKQVVDISITFRNDRPERERNDRADRPPRGGQRGGPSGGRGGRGGPRAGGNRGNANTPFSTSEDAFPALGAK

***Caenorhabditis remanei* VIG-1**

MFKLTFLAGYPYYGLLNSLELKNLTEASTLGILSSYTQINNKMTEYGCQVTNKFGLPSDDDDEYDDPRELIQKVSLIAAKKKEEKLVKPAQPTKPVAAPAAAVTKTDGAGRGARGGRGRGRGGAGRPPREGGDRVSNENGDRSGESRRGGPRRGGERGGDRGEGGRGAARPSGRGGRGGFAPRENRDGAEEPKEQHVADDGADTRAPRRRGGFTLGGGSSGGRGGARGGRGRQFDRQSGSDRTGVRAFEKKDGHGKGNWGDQKDELAGETENIAPEAENVEPEVPREKTAEELAYEAEQAALAKQKTLKEFRAAQANNLIQSITFKNFQADAPKFNTRKAGEGAADNFGKLVPMKKEIIPDREEDEVVVIHKASRKQVLDISITFRNDRPEREERRNDRDRSDRPPRGGPRGGGRGGRGGPRSGGQGGRHQATPFNASDDAFPALGAK

***Oesophagostomum dentatum* VIG-1**

KTCKTGSGSSKRGGEARWQGKQQASWWRKRGRGGRGRGRGGGPRPPRQEGDLADRFGENRGERGGRGGPRGGRGRGAPFRGRGGNANTFIDIPKDTQANPDDIPPVSPAAEFQGERRGRGGRGGFNGERRGRGGFRGGRQFDRQSGSDRTGVRGTDKKDGHGRGNWGDDKDELAGETEPIITSEEPEAPREKTAEELAQEALEAELAKQKTLKEYIEAQKKEAPKFNVRKAGEGEEENFGKLVKLQKEVVVEKEEEEVSIIRREPREKALHIDIQFAEPNRGYRGDRPPRGRGGPRGGGRGGRGGNRAQPPPHFDSSLDAFPALGSK

***Pristionchus pacificus* VIG-1**

MTEYGVNVNNKFLFAGDSDEELEDPSVLLQMAQKKDDKTPAQKKAEKKAEKLKKEKAAAAALAAKEAEAKKAAAAKPAPGKENSRPEGRGRGRGGLRGAGGERRPPRENTEGGEQRPFSGEGRGRGGPRGAGRGRGAFRGGRGGNTSGGEEHHEVSVEDTPKEGETQLTGERPAFAGRGGRGGRGGFRDGDRPPRGEYATRGDRPAGRGGRGRFGDRQSGSDRTGVKSVDAKGGHGKSNWGSNKDEIEAVAENAEVEKSEEETPAVEVVPREKTAEEIAFEEEEARIAAQKTLAEFRAAQKKDDKKFNLRQAGEGGDDKSFGKLVPLQKERLADETTTEEEVVEIVKKEPRNKQIIVDFKFNEPNRGEFGGRGRGGDRGGRGRGGAGGRGARTGRGGAGSGSAGHKEFRVAEDAFPALGKA

**AIN-2**

***Brugia malayi* AIN-2**

MAGRFNNRYSKIIHAREAKTLTFLAAQQMWPGGSGSSIRNSNAPAAMSSSSGGNGNSSTGSGSGTATGSQPSFSGVRVKDQHGGVGNQQRWLDHHQTQQWNTQSAQWGPHXASGPSPAHPWPLSAAAQWPGTPSPNTGTGASTYAEHAKKNMVGRXGQGQVQGRYDQQQQQWNQVKVDQQTPWDTSSLAGFGNIHATTDAKISATEWGSAAAANTRWAPPVTQWPVHAAATSSTTGPDWATAAAVVAAAAVAHHRHDAATPWTNTTLAAHPAAAALPPQVGAAWGQNPTLQQPSASALPNLNEQYDPNPQTPGPWVAPQPQEMNNDMMWHDPNPKQKKVQRDTGTAIWGDPSQQPVVSLKILCVLRFLILKLLPVAFLRRKCLVQEIKRWKDAEVDDYSHIQSAIPSGGDWNIIAISSNGSTVCTGVNGAIMGGXGIGHATSSCCSATGTNVSTPTTTGNGPWPDGSHPEPSSSGHQERWQQQPSAWGDKVSGPPSVGGIGMHGLLDNAVIRPDIGSGGIAGTAPYTSLTQQIADRIRIAVNKGLIDVSMLNRPLPQSALVILNALLQKFPKLEQAQQEYQQVMRAMSSPAQKVGVESERLAVEINGLQNEIVQLRNSINEQLLKVRSNALINGTLAPVQQQDGQSRLQQWKQANATNAQDCNSEKMCAPSSDPNVSGLITGTQLLTIDGKIDWKVGSLDWSPPPSGTGDKAADITDKDDPSSSNEKQHQGASSSTNQSCNGTPTPQQSQTQAQMASVDDGPQEFVPGKKWEWRDPNKVAEDPNATPGTCKPNPLLTTGSAAQPFYLYGSSANNSSPQGSAITNSSSAVTTTANYLNDLQTINRSPSAGYLGWNSGTANPAAFAASDMWPSVTTGTAAVRQSRIASGLTGIGPFGHTAVRGGPVGSSGLSVGPYQRSNLSPFGLQQPQQHQWVFVQLHGVNEKQIQMLCQKVGQVVHAFCPHGAPFLCVKFMEPAEEIIRRMKTEAPFLQVKTVTESEMDRLLKPRAALSYGGPLGATGTTTEQWMFSAGTVATLLPNCASNNXDALPQQPPFHAADTASDLSRPF

***Caenorhabditis brenneri* AIN-2**

MYFQPNEADPGMNGDGWQGPGGRGGSHHMNGGGGRRGGGGGGGYNQRMPPNQHHGGMNRMMPPAFRPQQQQYMPPYPPMGGMPGDVMDLNMRMDGMSLASTPTQAMPGQYGDMGPPLPQWNQPAPFAAPPPQDDLYDPTGGYGVHHQQMPPYGQQQQQRGGQQNQYGNRMQQGGGNRGYQQRQQNYNMPPMMAPPNQSPYFGGGNQNHQQQPPYAPSPFSMSGSQTPHDHHMSGGNHGPNGNFGGTSSSMDDYSMWTDENDEEAKRKKTLRDKGLLAWGDAEVSNSKPIRRWLVPEGHEEDFETAMERCPSHLKKKALNEEALRRRLGSDNPQVVAQAQQQAEEEANATMKIGRRPIIPCGWGDLPSEIAGEKHHEYDQGSSKVWDDGLSGGRHQQHHQQQDDSSLWNNPSAGGQSSSETRNPFFAQHMQQQQHHQQQQHQHYSQGVYDNGVLDGGSVWGAVMDGSSMGPPPSMGGGPSSSSMPMGMLMMNAGVGGGVGSSSADEDGSQKKLAENLKLAVEKGHLDISLLSLPHIPPNVLDLLTEILTVIPRLDSYEDELKILGDSRPSNSEESNKDGQDATSWMNKDQKNEHEKAVIGVVTAKIEVTQLSKKINEALVEAGLIPAPPLSQMSNNPNHHHGPPGPPGPDGGAGPNSSRGGDSFNNSGGPPDHYYDYSFLG

***Caenorhabditis briggsae* AIN-2**

MHHRRGNRGGYRGNHHHHHHQHNATPPKQLYVDTGPDGSGLVPIGYAPQAIMVYQEFIPNEAAEQSGMNGDGWQGPPGGRGGSHHMNGGGGNRRGGYNNQRMPQQHHGNMNRMMPPAFRPQQQPQYMPPYGPPMGGIPGDVLDLSMRMDGMSMASTPTMAPYGDMGPPPPGVQQWNQPTPFAGPPPPQQDDLYGDPMGGGGGYQPPQHQMQQQPPYGQHRSGGGGNNYGNNRMNQNNRGYQPRNQGYIPPMMVQQQNQSFYGNQQGAHFNPSPFSMASGGGGSQTPHEHHMGGGGGHNGSYGGSQAGGQMDDYSMWTDENDEEAKRKKLLRDKGLLAWGDADVSNSKPIRRWIVPEGHEEDFETAMERCPNHLKKKALNEESIRRRMTSDNPAVVAQAQLQAEEEANATMKIGKRPIIPTGWGDLPSDIPAGPEKSVDYDQGSSKGGWDDGGFRHPAPVQQQQQYQQQQDDSSLWSNPLSGVFGGPSSSETRNPFFAHHMQQSAQQQQQQHQYGDNGVLLDTGSVWSPVMDGSTMGPPSMIGGNGGNSGGGVGGGGPSAASSSLPMGMLSMMMMGGGGNGDIDQLKLAEKLKLAVDKGHLDTSLLSVPHIPPNVLDLLTEILEVIPRLDALEEELDKVGRNRPTNSDGEGTSSGGGAPPPSDQATSWMTKDQKTEHDKRVISVVTTKIEVTQLSRKINEKLVECGMLPPSILQQHQPNNNSGNGGPSGPSDGPSGSGGGGGGEFNGNGTAPPDHYYDYSFLG

***Caenorhabditis remanei* AIN-2**

MNGDGWQGPGGRGGAHHMNGGGGGGNRRGGGGGYNQRMPPSQHHGNMNRMMPPAFRPQQQQYMPPYGPPMGGMPGDVMDLNMRMDGMSMSSTPTMPGQFGDMGPPVPPPPQQWNQPAPFAAPPPQQDDLYDPTGGYGVHHHQMPPYAQQQQQRGNGGQNQYGNRINQGGNRGYQQRQQGGYMQPMMVPQNQQQFYGGNQNHQQQPPFAPSPFSMTGSQTSHDHHMGGGGNHGPNGNFGGSGGGGSSQMDDYSMWTDENDEEAKRKKTLRDKGLLAWGDAETSNSKPIRRWIVPEGHEEDFDTAMERCPSHLKKKALNEEALRRRLGSDNPQIAAQAQQQAEEEASATLKIGRRPIIPCGWGDLPSELGGDKNEFDQGSSKGWDDGGLVSGGSGARHHQQHQQQNEDNSSLWNNPISGGPSSNETRNPFFAHHMQQHQQQQQQQQQHQQHYSQGVYGDNGVLDGGSVWSAVMDGSPMGPPPPMSGGPSSSSAPMPIGMLSMMTAGGGPMSADVDGSQSKLAENLKLAVEKGHLDISLLSLPHIPPNVLDLLTEILTVIPRLDNYEDELKKLGDNRPSNSEEGTSQGEQETTWMNKDQKNEHEKAVIGVVTAKIEVTQLSKKITEALVEAGLIPAPPPQLISNPNHHHHGGPPPGSDGAGPSTSGGGDYNGGGGPPDHYYDYSFLG
