# Supplementary material for: RNAi Effector Diversity in Nematodes
Source: PLoS Negl Trop Dis. 2011 Jun 7;5(6):e1176. doi: 10.1371/journal.pntd.0001176 (PMC3110158; doi:10.1371/journal.pntd.0001176)
Supplement: Dataset S4 — Nematode RNA interference (RNAi) inhibitor proteins; domains and sequence data. (*, putative stop codon) (DOC) [file pntd.0001176.s004.doc]

**Dataset S4.**

**Domain analysis**

ERI-1 (Enhanced RNAi 1) encodes an amino-terminal SAP/SAF box domain in addition to a carboxy-terminal 3’-5’ exonuclease domain. ERI-5 contains an amino-terminal tudor domain. ERI-6/7 contains a superfamily I helicase. LIN-15b encodes a carboxy-terminal zinc-dependent DNA-binding THAP domain. Both XRN-1 (XRN ribonuclease related 1) and XRN-2 are typified by an amino-terminal 5’-3’ exonuclease domain. In all cases, the aforementioned domain topology was required of putative orthologs, or minimally, one matched domain in the case of short sequences. ERI-3 is a novel protein with no known domains and as such, putative orthologs were identified on the basis of overall sequence similarity.

**Shared protein domains**

None of the above proteins shared known domains with each other.

**ERI-1**

***Ancylostoma caninum* ERI-1**

VMRRLHSLTAAEMRTLLKQVHQSGQGTKKQLRTRLRRYYRKEFSMYRMLHDGDSIPRFGNKTARHFDYLVAIDFECTCVEVIYDYPHEIIEFPAVLIDVRQMRIVDTFRTFVRPEKNPILDPFCIQLTGITQEMVDSAPVFKDAYRLFRDWMTQHSLGDTGHRYAFVTDGPHDLWKFFQFQCILSNFGQFPMTAGILSILSVFSNKE

***Ascaris suum* ERI-1**

MESRRSVSNKRGRHNENTGRRDPRPATRSHVSTQQYFDYFLVLDFEATCEQNAKIQPVQEIIEFPVVQFCTKTLQEVARFHEYVHPTERPVLTSFCTNLTGIVQEMVDNQMILPDVLAKFRRWLSQQCLIDAETGDRVRNSWTFVTCGDWDLGTILPQEASFRGLHLPPYFGSWINLKKAYRNAKGYFPNSLMVMLNDLQIPHTGRLHSGIDDVINICAIVRKLCEDGYLLXXTSYLTNNVVSGQRAPLF

***Brugia malayi* ERI-1**

MDKSMFSAESVSHNEELEKFYELIIDASQKVQEMNLRDGGSYEDNNATEDNNAPYMLPVVLYKEPLRKNPKHVLHVMQRLHFMTTTEMQKELKKAHIDSRGKRNQLYSRLKKCFRKDFTIIKNADPLRSKTNIFYDYFVMNNYDCSQVIDFECTCEADLYDYNHEIIEFPAVLVDVRKKEIVDVFHSYVRPLANPQLSEFCSAFTGITQEMIDKALPFIDVLDSFRTWMQLHRLGQNDMRYAFVTDGPWDIAKFFQMQCIQSKLNTVPHDFRFYINIRRSFANKYCKKYSMQKINLGGMLTFLNMKFEGREHSGLDDSKNIARIVIKMLEDRSELRVNEKLVRIKESEKAKLLPNMKMNKEDRDRQSWRNNLPYIVQQISRDSFISGEYLDCNTCDESD

***Caenorhabditis brenneri* ERI-1**

MSAEGSTENEKFLESLRSLLKVSHDHEKSTTECNFSFVFSQFIIKRLVESAIDNSRTDDSENYVEIPHEQQIEPATRVEPLKSMVEPDYVRKVIRQIDTMSSEQLKEALTKIKVSTGGNRKTLRKRVAQYYRKENALLSRKLEPVGNKTLRYFDYLIATDFECTCVEVIYDYPHEIIELPAVLIDVRDKRIISEFRTYVRPVRNPVLSDFCVDFTKIAQETVDEAPYFREALEKLYQWMRKFGLGEKKTRFAFVTDGPHDMWKFMQFQCLLSNIRMPHMFRNFINIKKTYKENFNGLVKGNGKSGIENMLDGLGLSFIGNKHSGLDDAKNIAQIVIHLMSHYELRINQKCSWTEPAAQTIKENDEELGDTADLKSIDVSRRDFQLWLRRLPLKLSSVTRREFLSEEYLDCESCDELTDEKVKERLRHTYKCDIYQIFEKNKA

***Caenorhabditis briggsae* ERI-1**

MTAEQNPEDEKYLESLKNLLKVSEEHETNQQTNEEVDEDDVETEESDIQPTIPPEHQIEPAERIEPLQSMVDPDYVRKVIRTMDTMSTDQLRQALQRIKVSTGGNKKTLRKRVAQYYRKENALLNRKLEPNSDKTLRYFDYLIAIDFECTCVEIIYDYPHEIIELPAVLIDVREMKIISEFRSYVRPVKNPKLSDFCIQFTKIAQETVDEAPYFREALEKLMQWMRKFGLGEKNTRFAFVTDGPHDMWKFMQFQCILSNIRMPHMFRNFINIKKTFKEKFNGLMKGNGKSGIENMLERLDLTFIGNKHSGLDDARNIAQIAIQMMKLKIELRINQKCSWVEPKNQIIKDDDDLDDNVDTIDVSRRDFQLWLRRLPLKLSSVTRREFLSEEYLDCESCDDMTDDKHDRQSFAEKMTIRQQIEALDEEEREKMKNEPPIVRNGPPKPVPPPPATTTQENDDSENDEEFLRDYQRQVELLDMEESLDAISNSNGIELEEVWECAGSEMAAESVSLDNLAYSSSESLSLEQFQYPTSQNSTHPPTPETDVMMNSDSIESMELMAPPPKNSLASTNRNNSRNY

***Caenorhabditis japonica* ERI-1**

MENEPNPEEEKFLQSLQNLTTISEEHERLRRNKNQQDGADNEDSGDEYPIIPSEQQIPPSERVEPLKSMVPPDYVRKVIKEMDMMSTEMLRQALLRIKVPTGGNKKTLRKRVAQYYRKENMLLNRKLETNSDKTSRYFDYLIAIDFECTCVEVIYDYPHEIIELPAVLIDVREMKIVSEFRTYVRPVLNPDLSDFCIQFTRITQETVENAPYFREALLRMYQWMRKYGLGEKNARFAFVTDGPHDMWKFMQFQCVLSSIRMPHMFRSFINIKKTFKEKFNGLIKGNGKSGIENMLERLGMSFRGNKHSGLDDARNIAAIAIELMKLKIELRINQKCSYKEIRAGHKEHEDEIADVTDLKLVDVSRRDFQLWMRRLPLKLMSVTRREFLNEEYLDCESCDELIDDKEDPECHSKKMAFRKMLEESQKEEFAEYCKEHGNFKIGEIVPKLKNIHNQNDDDDDSEDEDYGPEFAQFVNL

***Caenorhabditis remanei* ERI-1**

MTLSPEDEKYLDSLKNLLKISEEHEKLRNLQKMETPDEEVTEDNLRTEGSEDPVEIPLEQMIEPAERVEPLQSMVEPDYVRKVIRQMDTMTTEQLKQALMRIKVSTGGNKKTLRKRVAQYYRKENALLNRKSEPNSDKTYRYFDYLIAADFECTCVEVIYDYPHEIIELPAVLIDVREMKIVSEFRSYVRPVKNPKLSEFCIQFTSRWPPLLVPSLIDFLPEIAQETVDEAPYFREALDRLIQWMRHFGLGEKNTRFAFVTDGPHDMWKFMQFQCLLSNIRMPHMFRNFINIKKTFKEKFNGLVKGNGKSGIENMLERLELSFIGNKHSGLDDARNIAQIAIQMMKLKIELRINQKCSWHEPSYHTLKEDEEIGDHVDLASIDVSRRDFQMWLRRLPLKLSSVTRREFLNEEYLDCESCDELTDDKNDAQSFEDKMANRAEIETIDEDEFNKFAEGAPANEPTPTPPPVRDNEDSESDEEEYRREFEMMDIVDSISSEPPSEAVELNEIWQRRGSESDGQEGVVSLGDYAYSTSRATSSLVSSTRVVSFDDILETSSVEDMELMAPPPKNSLASTNRSSRNDNY

***Haemonchus contortus* ERI-1**

LQAIDFECTCVEVIYDYPHEIIEFPAVLIDVRQMKIVSQSGIQNMLSHYNLTFEGQKHCGLDDSTNIARLCIKLMQDKIELRVFPKGISQNMVDSAPVFKDAYRLFRDWMAHHNLGDVDHRYAFVTDGGTKKQLRARLRRYYRKEFSMYRMLREGDNMPRFGNKTARYFDFLVIPHDCRHFINIKRIFEQRVMKLVKGNGVSPHDLWKFFQFQCILVSVVDKFRTFVRPEKNPILDPFCIQLT

***Meloidogyne hapla* ERI-1**

MINERVEYLFEFFESMSDIELQREMEIIDEDSSELKRSKIIATLRDHYRDALIELRKKYSTRKFYNYFIVIDFECSCEENNYDFEHEIIEFPAVMISVESCNIQEDVDKAPTFPIALQLFRGWMAKHGLDGRRNMGKARRFCYITDGPWDIGKFFQMECFRSNQSIPHDFRCFMNIRRSFVNFYTMQSQPPRYHCDGNSVHVVEGTTTKEKLFPNGISLNIMLKHLNIQFTGREHCGMDDTLNIAFIVIKLLEEGAELRQKLVFNEKTKAPSCVASTSSSIENDDKKEKNPKNILKMPNEEDKSNGNEKIFVWKKWWDEMPYKLVRITRYEFLGDRHLECESCDEGDN

***Meloidogyne incognita* ERI-1**

MINERVEYLFEFLKSMSDIELQREMKRIDKDSSKLKRGKMIATLKDHYRDTLIELRRKYSTKKFYNYFIVIDFECSCEENNYDFEHEIIEFPAVMISVQSCKIIDKFHTFVKPSINPRLTEFCKDLTGITQENVDKAPTFPIALQLFRGWMAKHGLDGKRNMGNARRFCYVTDGPWDIGKFFQMECLRSNQSIPHDFRCFLNIRRSFVNFYTMQSQPHRYHCDGNSVHVVEETTTKEKLFPNGISLNIMLKHLNIQFTGREHCGMDDALNIAFILIKLLEENAELRVNEKLVRGKDVKNWQKLIFNEKTKAPSCIASVSSIENDGDRREEDSKKDPKTKEEKDENNGDVQVFVWKKWWDEMPYKLVRITRYEFLGDRHLECESCDEEDN

***Oesophagostomum dentatum* ERI-1**

CDVVESSPKELAEYYEKLLNISDELEKKRKTEGSTAYNPDGSAVIRTSMPPENVLKHVEYEEPPKGIEAETLIDVMRRLHSLTTAEMRTLLKQVHQSGQGTKKQLRARLRRYYRKEFSMYRMLHDGDCVPRFGNKTARHFDYLVAIDFECTCVEVIYDYPHEIIEFPAVLIDVRQMRIVDTFRTYVRPEKNPVLDPFCIQLTGINQEMVDSAPVFKDAYRLFRDWMTQHNLGDSGYRYAFVTDGPHDLWKFFQFQCILSNFGAVPHDCRYFINIKRIFEQRVMKLVKGNGQSGIQNMLAHYNLTFEGQKHCGLDDSINIARLCIKLMQDKIELRINQRMTQRQDKNEDRRLEELAKSDKADASDYHIWHRKLPLKLRQVTRDEFLSEEYLDCDSCDELDE

***Pristionchus pacificus* ERI-1**

METHEVGETPLAKMNPREEHLLSDYYDKLMKISDDVERETAVGILANDDDVRRLHKPPENHLDTVLAGDSDKGIDPKKVERLFKRINEMTAAEMRIELKEIHKSPHGSKKELRFRLRHFYRKEFSLIQEKKDYTRARSNNRKRFKYLVAIDIEATCEAETNDINYPHETIELPAVLVECNNFTIIDRFRTYVRPEINPKISEFCSQLTHISQSDVDAAPLFPEAWARLVEWMSRWGMMGDDKDASFAIVTDGWYNMNIPHEFRHFINVKKIFELRVERLSKGDGKTSIAKMCEKLNLEMEGTAHQGIMDAVNVAKIACALVLSKKLTLCVRSQFN

***Trichinella spiralis* ERI-1**

MSNDTDEKLIESDSSDLSLYSSINSLLPRTIHMELEKDNLCSNEGSDDLEERFYMHLRILDRIRKLKAFAENNPLPFTDYYCVLHFECTCATEKSGKTYSNEIIEFSSLFIHASTAKVKQLLYFNLDIPKELRSWIDLKKAFSDILRVNLLKLSEMLDVIGLHFECTKHCGMDDAKNISRIVIWLSELKIFLQKNAKLSKNGKFSMKLSDNYNNFKTPPDDNGSSFSLKTEELPYLFTVKRSKYYARLRELSSTSDEDDEDD

**XRN-2**

***Ancylostoma caninum* XRN-2**

LLPFVDEKRLLEVLKPMEDKLDAFEKERNSRGPDRLFVGSSHPFYKFAEQLYEGDKEGSIALPIDTSLTFGMAGTVGADAKAIMKGENYKSPFLVNEFADIAENKAIMMIYKDPQFEKGYVFAAKRLSGAIDVPRTLKDINLDRKEPYRPQIGFSREIPRHGLSDAGRRMLGHNIGHGPRPLFEAYQIDSHNRPHQSSSWIPAGSYAEDRMPNYSPTANQFRERRQTGGEWYGNSRRYRNFNEYERISQLPLPLRHPFRYEPYPELISSRARSRGRDRPLQHSVYRW

***Ascaris suum* XRN-2**

MDFIRRQRASPSHDPNTVHCLCGADADLIMLGLATHEPNFNIIREEFIPNQQRPCDLCGQYGHEVKDCQGLAQDEAAECDANPMQKETNFIFVRLPVLREYLEKELQMNNSSVPFDLERAIDDWFLRSVMCYVFLVGNDFLPHLPSLEIRENAIDRLIRLYKDMVAKLLLKALKDLLMDV

***Brugia malayi* XRN-2**

MGVPAFFRWLTRKYPSVVVDAVEEKPRDLNGIRVPVNTVDPNPNFQEFDNLYLDMNGIIHPCTHPEDRPCPKTEEEMFMLIFEYIDRLFAIVRPRKLLYMAVDGVAPRAKMNQQRSRRFRASKEAIEKVEQIAETRLRLESEGYPLPPEKKTEHFDSNCITPGTPFMARLAVALRYYVHQRLNSDPGWRKIAVILSDANVPGEGEHKIMDFIRRQRASPSHDPNTVHCLCGADADLIMLGLATHEPNFNIIREEFVPNQQRPCDLCGQYGHGLNDCKGLASDDNSECQSTPLQKETNFIFIRLPVLREYLERELHIPNCSIPFDLERAIDDWVFMCFFVGNDFLPHLPSLEIRENAIDRLVKLYKDMVCEMKGHLTDSGIVHIERAQIILDKLGEVEDEIFKSRQEREQQFKRRNKEKKWREQALATPSYIPSRGSLLSPQTNGPMYLSGSSTRTMARTARLEALDFTETVHTRAMNNATFHVPAINISEKDDHKQELKNNSSSEDEVFDEVRLWEDGWKDRYYKSKFQVDGKDIVFRRKVALAYVEGLCWVLHYYYQGCASWDWYYPFHYAPFASDFDAVSQFKPDFSAYTEPFKPLQQLMSVFPAASRMHLPEAWQELMISPVSPIIDFYPDNFVIDLNGKKCAWQGVALLPFVDEKRLLEVLNPMEDKLDAFEKERNSRGPDRLFVGPSHPFYNFAETLYENSRKDSTDLAIDTSLTFGMAGTVGVDAKAVMKGQNYKSPLSVDEFTDIAKNKAIMMIYKDPQFEKGYVFAAKRLPGAVDVPRTLKDTNLDRREPYRPQIGFLREIPRHGLTDAGRRMVEHSIYHSPRPLFEPYQTGPSNRLYQSCWAPAEPYVDNRVPVCPSSANQFREGRQVSGEWCGNPRRRRNFYEHRRIPQSPMPVRHSLRYEPYPELVPLRVRGRGREWPPQHPIYRW

***Caenorhabditis brenneri* XRN-2**

MGVPAFFRWLTKKYPATIVNANEDRQRLDDGRRAPIDCTQPNPNFQEFDNLYLDMNGIIHPCTHPEDRPPPKNEDEMFALIFEYIDRIFSIVRPRRLLYMAIDGVAPRAKMNQQRSRRFRASKEMAEKAASIEEQRRRLMAEGIAVPPKKEEEAHFDSNCITPGTPFMARLADALRYYIHDRITNDAAWANIEFIPRIPRNYHQSYPEFSKFIEINFQIILSDANVPGEGEHKIMDYIRKQRGNPAHDPNTVHCLCGADADLIMLGIATHEANFNIIREEFVPNQPRACDLCGQYGHELKECRGAENDTDLGDDYCKPEQREKNFIFLRIPVLREYLEKEMAMPNLPFQFDLERALDDWVFLCFFVGNDFLPHLPSLEIREGAIDRLIKLYKEMVYEMKGYLTKDGIPELDRVEMIMKGLGKVEDEIFKRRQQDEERFKESQKHKKARMQQFGGRGRGRGRGRGQPAYVPNHGILAPMSAPMHHSGESTRQMASDARQMAMQYNNDANAVAAAHLKALLNIKGEESPAEVASRKRKAEQPLILPEEEDEEPKDDIRLFESGWKDRYYRAKFDVGSDDVEFRHRVAWAYVEGLCWVLRYYYQGCSSWDWYFPYHYAPFASDFETVGDFKPDFSRPTKPFNPLEQLMSVFPAASKQHLPVEWQKLMTEDESPIIDLYPADFRIDLNGKKYAWQGVALLPFVDEQRLLETLKTVYPTLTDEEKMRNTRGPNRIFIGRNHKSFAFFNTVAESKSTELVDLDPSLLNGVSGKIAADSTATAPGLPFVSPVNHADCQDLPTNCGICVLYEDPEYPPGYIFPAVRLEGAKEAEKTLKPEDWNERRDGRFHPQVGFNRNAPRGGLDQSSHRHVNHHVRGAMQDRYGGGGNGGGYRGGGGGGYQGGYGRGGGGGYRGGYNDQRQDYGRNYGGREGGGPQRYHDQGYHGGGGGYHHHQQQPYNPDQRRGGYQGGGRGQGPPGYQRPPYRGGRGGGGGGASWR

***Caenorhabditis briggsae* XRN-2**

MGVPAFFRWLTKKYPATVVNANEDRQRGVDGRRVPVDCTQPNPNFQEFDNLYLDMNGIIHPCTHPEDRPAPKNEDEMFALIFEYIDRIFSIVRPRRLLYMAIDGVAPRAKMNQQRSRRFRASKEMAEKAASIEEQRRRLIAEGIAVPQKKKDEEEAHFDSNCITPGTPFMARLADALRYYIHDRVTNDPAWANIEIILSDANVPGEGEHKIMDYIRKQRGNPAHDPNTVHCLCGADADLIMLGIATHEANFNIIREEFVPNQPRACELCGQYGHELKECRGAENDTDLGDEYCKPEQREKNFIFLRIPVLREYLEKEMAMPNLPFQFNLERALDDWVFLCFFVGNDFLPHLPSLEIREGAIDRLIKLYKEMVYEMKGYLTKDGIPELDRVEMIMRGLGKVEDEIFKRRQQDEERFKENQKNKKARMQQYGRGRGGRGRGRGQPAYVPSHGILAPMSAPMHHSGESTRQMASDARQAAMQFNATNDANAQAAANLKALLNVKGEQSPAEVAAQESRKRKAEQPIIITDEDEEPKDDIRLYESGWKERYYRAKFDVGSDDVDFRHRVAWAYVEGLCWVLRYYYQGCSSWDWYFPYHYAPFASDFETVGEFKPDFTRPTKPFNPLEQLMSVFPAASKQHLPVEWQKLMTEDESPIIDLYPADFRIDLNGKKYAWQGVALLPFVDEQRLLETLKSVYPTLTDEEKYRNTRGPNRIFIGRNHKSFAFFQQVAESKSNDLVDLDPSLLNGVSGKISYDSTATAPGLPFPSPVSHEECQDLPTNCGICVLYEDPEYPANYVFPAVRLDGAKEAEKTLRPEDWNERRDGRFNPTIGFNRNAPRGGLDLSGQRHINHHVRGAMYDRQGGNDNYRGGYRGGYQGGYDDRRGGRGGGGYRGGYNDSRPDFGRNYAGREGGGPQHYHEHQPGGGHGRPHDQQPYQDNRRGGGYHRGGRGNGPTGYQRPPYRGGRGRGGGGYQGNSSWR

***Caenorhabditis japonica* XRN-2**

MAIDGVAPRAKMNQQRSRRFRASKEAAEKAASIEEQRRRLMAEGIAVPPKKEVEEHFDSNCITPGTPFMARLADALRYYIHDRVTNDASWANIEIILSDANVPGEGEHKIMDYIRKQRGNPAHDPNTVHCLCGADADLIMLGIATHEANFNIIREEFVPNQPRACDLCGQYGHELKECRGAENDQDLGDDYCKPEQREKNFIFLRIPVLREYLEKELSMPNLPFKFDLERALDDWVFLCFFVGNDFLPHLPSLEIREGAIDRLIKATGLFLKLYKDMVYQMRGYLTKDGIPELERVEMIMKGLGKVEDEIFKRRQQDEERFRENQKAKKARQQQFGFGRGGRGRGRGRGAPAYIPNQGILAPMSAPRHHSGQSTRQMASDARQTAMQFNDDANAQAAANLKMLLNVKGEESPAEKDSRKAGFMKLKNDRKPVLRKITFGN

***Caenorhabditis remanei* XRN-2**

MGVPAFFRWLTKKYPATVVNANEDRQRDADGKRVPVDCTQPNPNFQEFDNLYLDMNGIIHPCTHPEDRPPPKNEDEMFALIFEYIDRIYSIVRPRRLLYMAIDGVAPRAKMNQQRSRRFRASKEMAEKAASIEEQRNRLMAEGIAVPPPKKEEAHFDSNCITPGTPFMARLADALRYYIHDRVTNDASWANIEIILSDANVPGEGEHKIMDYIRKQRGNPAHDPNTVHCLCGADADLIMLGIATHEANFNIIREEFVPNQPRACELCGQYGHELKECRGAENDTDLGDDYCKPEQREKNFIFLRIPVLREYLEKEMAMPNLPFKFDLERALDDWVFLCFFVGNDFLPHLPSLEIREGAIDRLIKLYKEMVYEMRGYLTKDGIPELDRVEMIMKGLGKVEDEIFKRRQQDEERFKENQKNKKARMDQYGRGRGRGRGRGRGQPAYVPTFGILAPMSAPMHHSGESTRQMASDARQTAMQFNTTNDANAQAAANLKALLNVKGEESPADVAARESRKRKAEEPLVVPEEDEEPKDDIRLFESGWKDRYYRAKFDVGSDDVDFRHRVAWAYVEGLCWVLRYYYQGCSSWDWYFPYHYAPFASDFETVGEFKPDFTKPTKPFNPLEQLMSVFPAASKQHLPVEWQKLMIEDDSPIIDLYPADFRIDLNGKKYAWQGVALLPFVDEQRLLATLKSVYPTLTDEEKQRNTRGPNRIFIGRNHKSFAFFQQVAESKSTDMVDLDPSLLNGVSGKIASDSTATAPGLPFVSPVNHEDCQDLPTNCGICVLYEDPEYPENYVFPAVRLDGVKEPEKTLKPEDWNERREGRFNPTIGFNRNAPRGGLDPSGQRHINHHVRGAMSDRQGGDNYRGGYRGYQGGYDDRRGGRGGGGGGYRGGYNDQRPDYGRNYGGREGNVEEMFKNDDNFSGGGPQRHHDNYQGGHGGGGGGGGYNQQPYNQDQRRGGYQGGRGGGPPGYQRPPYRGARGGYQGNSSWR

***Haemonchus contortus* XRN-2 (Likely exon-intron boundary issue with predicted protein)**

SGQKYVWSGGTLLGSQTCHPQPIHCLCK*VNPYLLSGLDTVDSRVDLHEIKETKGPHNKI

DVEEKARNTTGPNRIFIGKKHPAFDFFKELYSNVNGVRLFEAGWKDRYYRQMNQQRSRRF

RASKEAWEKAESIEEYRPQIGFTRDVPQRASLDSSGHRFVQHNVQQDEERFKAIQKAKKA

RMSGGRPQAPSYVPREGSLIAPLSRATPMSGETTRALAATARQRLAEVLRYYIHDRITTD

PAWAKIQVVIYLTKDGNVYMDRVERIMLGLGEVEDEIFKRRQVC*ISRIFQDSRRQVSEV

NSVDIDPAFTYGMSGRITADRTALAPDLPFPSPVDDKSCPDLPVSFQACLQVNGAMMVIY

EDPHYPEGFIFPAKRLENAKEIPRALRDYSDRRVGFNRDSIQTIQVQHTLTEEEKARNTT

GPNRIFIGKKHPAFDFFKELEEFIPNQQRPCDLCGQYGHELKECRGALIFEYIDRIFSIV

RPRKLLYMAIDGVAPRAKVSLTRRSLASETYLDLLTDESATQQTLSSFERGMGEGRAKFD

VGADDVDFRRKVAWAYVEGLCWVLKYYYQENEEKGPEHADPISKEKNFIFLRIPVLREYL

ERDMAMENLPFKYDFERVIDDWVFQEFDNLYLDMNGIIHPCTHPEDRPAPANEDEMFVSI

FKVFLCFFVGNDFLPHLPSLEIREGAIDRLIKLYKDMVYTMKGMGVPAFFRWLTRKYPSI

IVNANEERQRNADGTKIPIDATKPNPNFQVSLYFGMFDSLPVLLNGRQHLPLQWQKLMTD

EDSPIIDLYPTDFRIDLNGKKFAWQGVALLPFVDEVRLLRTLKTVGCVSWDWYFPYHYAP

FASDFDTVSQFKPDFSKPTKPFKPLEQLMSVFPAASKVILSDANAPGEGEHKIMDFIRKQ

RSNPAHNPDTVHCLCGADADLIMLGLATHEANFNIIR

***Meloidigyne hapla* XRN-2**

MGVPAFFRWLSRKYVSIIVDAVEERRKEVEGIKIPVDCTQPNLNYQEFDILYLDMNGIIHPCTHPEDRPAPKTEEEMFILIFEYVDRLFSIVRPRRLLYMAIDGVAPRAKMNQQRSRRFRAAKEAAEKREQIASIRKRLENEGVPLPPPRKEEEHFDSNCITPGTPFMARLSTALRYYIHKRITYDPAWMKIQVILSDANAPGEGEHKIMDYIRRQRASPSHDPNTVHCLCGADADLIMLGLATHEANFNIIREEFVPNQPKACELCGQYGHELEHCQGLARVEAGPDQVDPLKKEKNFIFIRLPVLREYLERELFMPNLPFAYDLERAIDDWVMMCFFVGNDFLPHLPSLEIRENAIDRLVKLYKNMVYQTGGWLTCDGVVNIDRVKMIMHELGKVEDQIFRERQLRELKFKESQKQRKRRAKEAAEMLMPYNASLIAPQPISSQNLNKSGEEIRKMAADDRKQMQLAAEQAQKLKSLLTPVAVTTIGSKRKMEQTLNDSPSGSPPIKDGRVVEDKSTLDGPKPEIVFTTPSGGKAPLAKGAVGALMNDDSDEEETTDEIRLYEEGWKERYYRAKLHVEESDTELRKNVVCAYIEGLSWVLLYYYQGCASWTWYFPYHYAPFASDFYLAGDYKPDFSKSTRPFKPLEQLMGVFPAASRAHIPKGWHWLMTDEKSPIIDFYPDDFEIDLNGKKYAWQGVALLPFVDEKRLLDALNLVYDILGDEERARNDTGPDRLFAGKQHGLFELMHSVHQLTKENNGNSIVTSSVERSRISTGNSQSVESERSQSNEEDSNERSQSTEESNDRSQSNEGDSNERSQSNEEETNEEQSHSTGEEQSRITSNETNSGENNEIVVAKDEKQWVIIDASKAYGMAGEIHPDESAIDYGVDYVSMFRGSIEFGDIKKNSCVMVQFRDPQFPPRFQFKACRLDGIKELPRALKPQNYDDRRQGPYRPQIGFSRDIPRASLSSSGHRTLDHYVTQQNRRSGPASLFAYQAQQPQQVQASPQQQLPWHSGAAQQQYQQLMPSDFQQPLPLNFMQMAMPPPPLPPQHWVNPMQQQQQQPYVGQWGQPPSLLQNQNYGGNNNSDTWRRRSDDGGGGSSRSNQQHLPYHNNQYGRSSVRNYNNDDNRNNSNSSQREYRSNQRRQ

***Meloidogyne incognita* XRN-2**

MGVPKFYRWTSERYPCLSEVINESQIPEFDNLYLDMNGIIHNCSHPNDDDVTFRITEDEIFVNIFAYIENLYNLIRPQKVFFMAVDGVAPRAKMNQQRARRFMSARTAHTQLAKALENGEIMPSEARFDSNCITPGTYFMTRLHQKLDDWIKTKSATDSRWQGRKIILSGHNVPGEGEHKIMDFIRTERAGSSYDPNTRHCMYGLDADLIMLGIVSHEPHFSLLREEVTFNTRPRKNDNKATKKPKRTDSDVKKFHLLHLSLLREYLAWEFADVKDSLPFEYEMERIVDDWILLGLLVGNDFLPHLPNIHIHDDALPLLYSTYKKVLPTLDGYINEAGYLNLSRFEAFLAELALNDKTSFMERLEDEQFMESKRIRVVDPEQQELAEPELVAFESSDVEDEAGAEESKDEAGGAGAEDDEDDAAFVSDHEEDGDDLEPGSGSDELLLSNLDAQLLEDEFNDELATLALSGMNDADFANDVEACWTKTINNQFKRHKKTYYSDKLRYKNISKQQLREQAEGYVRAIQWNLHYYYHGCVSWSWFYPHHYAPFISDVRGFVGMRMEFELSEPFHPFEQLLAVLPEASADCLPRPLRELMSSDPAKSPICDFYPANFETDLNGKRNEWEAVVLIPFIEEKRLLEAIEAKRSRLTSEENARNSHGSHIQCISSPEPPNSQGQWRTVRSEIPQDSFRIPRSQVKWGLLPNVKMDVYFPGFPTMKHLSHLGELRFANCNIFGMASRKESMVLKVENERAHTDIIELGSELCDEEVCIDWPILKVAKIDSIWGGDDKIVRKCDDEIIVKDMSEEEKRQWQAHANQLTEHLMTRFAIEIAAQKDSKGKHIRPAVAWVRKFTGLVYEAQGGGKEPSVLKACKQWSSPQQLMPVLLPLVVKDVLLENSRVLADLPMKQAYPKQSIVWITDPKFALFGMPGMVNGFSNEKSADCRIEVIGMSTQNKVDKMEALRKKMEQKSLRWMGGYDCARQCQVDTRLFARITGTMFLWNEPRERVEKGQQISSSDSKINCGLALKYSRRDLCVADYTDRTEHTNQRGVTNKVWFYTNLATRLVSEYRRKFPDVWKYLETIGLTQQDDVYYTEDIWSNEKTREKRFGELSEFLGGLPSLEAEQLKCGTVYADRQLITEIEMILAEPDEKKPVMNKYMMAPGALFRYELYNGKVHADPAADFQILDRVALMSSDTKVPKTTQGTVVGIHDDKIDVFFDKPFDGGQKVRGSNEAAAIRVPQSALLNVTFGIVRKNVQHKKQVEKALTGAYVPMPAQKNSAKNEPAPSTSSSNSNSTNNKKNSSKKEKNKQKPTKTDEESPEALTDSLNKLLKIKPPSAEATSGKQVSLMELLGGGGAAGAKKPAQNAKTLAEIESDSSQSASKSSILQQLSAAQKAAAPKPKKEASQKKQKPQAAPPQKVEILRKPTPPPPPPAEEQKPAPAPFGAPTPPPQFPFGPQPLQFAPHPMMMMGYPMGMPMGMPIGGPHHYQQQQNQQEKRANHPTLTDFKPSAVHRRQNCHQRPSNQPPKVTMITKRTSSPTPPEKEEDAAPRALPSSPPPPSKSAAEEKTTTKKPRKKKQSRLGSNFSNPTASS

***Oesophagostomum dentatum* XRN-2**

GLVSWDWYFPYHYAPFASDFDTVSQFKPDFSRKTQPFKPLQQLMSVFPAASKQHLPIHWQKLMTDEDSPIIDLYPVDFRIDLNGKKYAWQGVALLPFVDEDRLLRALEAVESTLTDEERARNTTGPNRIFVGRNHPAFDFFQELYTNTNGGAKFVDIDPAFTYGVTGRISADDTAVAPDQPFPSPVQDKTCPDLAVNGAVMVIYEDPQYPEGFIFPQSA

***Pristionchus pacificus* XRN-2**

MGVPAFFRWLSKKYPSIIVNANEERQRDVNGEKIPIDCSQPNPNFQEFDNLYLDMNGIIHPCTHPEDRPAPRNEDEMFVLIFEYIDRIFSIVRPRRVLYMAIDGVAPRAKMNQQRSRRFRASKEMAEKAMDIENVRERLAAEGLPLPPKKAPEEHFDSNCITPGTPFMARLADALRYFVHQRLTTDPAWAKIQVILSDANVPGEGEHKIMDYVRRQRASPSHDPDTVHCLCGADADLIMLGLATHEANFNIIREEFVPHQQRPCELCGQYGHELKGCTGLDATEKGADEADPVNKEKNFIFLRIPVLREYLERELAMPNLPFKYDFERVIDDWVFLCFFVGNDFLPHLPSLEIREGAIDRLIKLYKDMVWKLNGYLTKDGDVNMKGVQQIMVGLGSVEDEIFKRRQQNEERWKEREAAKKQRTGDWQGRRPQRPSYTPGDNSLIAPGGGGRNLTGEQTRAMARDDRVQESVAKAHREAEQEEEVDPEPVDEVRLYEDGWKERYYQSKFCIEGDDIDFRREVAWAYVRGLCWVLKYYYQGCVSWDWYFPYHYAPFASDFDTVAQYVPDFDLDTKPFKPLEQLMSVFPAASKQHLPTEWQTLMTSSDSSIIDFYPVDFKIDLNGKKFAWQGVALLPFVDEKRLLRTLKKFLQKPVQIEPAFAYGMAGAVRADSTAILPGTVFKSPVRSEECPDLQETRGIMENGGIMVIYKDPSFPDGFIFPAQKLEGAKELERTLKPGDWDDRRNGQYQPMIGFTRNTGGRASLDSSGHRAIQHEVGMGGGRNAPGGGQWERREGAGSDWNGGGGGGRGGYGGGGYGGGRGGGGGGGRGGYGGNYGGGGGGGYQQQMRHSDGGWAAQNA

***Trichinella spiralis* XRN-2**

MGVPAFFRWLSRKYPSIVMNCIEDTPRDVDGTTVPVDNTQPNPHGIEFDTFYLDMNGIIHPCCHPEDKPAPKSEEEMMVAIFEYIDRLMCIVRPRRLLYMAIDGVAPRAKMNQQRTRRFRASKEAAEKEEQIRQIREDLRAQGIPLPAESTDKQHFDSNCITPGTPFMARLAICLRYYIHERLNTDPAWQNLLVILSDASVPGEGEHKIMDYIRHQRACASHDPNTHHVLCGADADLIMLGLATHEPNFTIIREEFVPNLPRPCEICNNYGHTMQDCQGLSILENENEEAHRPVLKKTQFIFIRLSVLREYLQRELEMPNIKFKYDFERCVDDWVFMCFFVGNDFLPHLPSLEIREGAIDRLVKLYKDCVYRTGGYLTENGFVNLKRVQLIMSELGKVEDEIFRQRQEREAMDNIFLLDWIQFSNKAKMRRMQAENFDAPAFIPQNAFAPTPIGESPLPLSNAKRTAMEMRQAAMAVTSSTKREFNGAANAEDANDEGPLDEVRLWECGWKDRYYLVKFQCSPKDLEFRHHVANCYVEGLCWVLRYYYQGCCSWKWYFPFHYSPFASDFLNIGDLKIDFSEKTMPIKPLEQLMSVFPAASSKHLPKSWAALMHDPVRDKSTIIDMYPSDFKVDLNGKRYAWQGVVLLPFVDAERLNEALEVVYPDLTEEERFRNKQGNDLLFISSKHEAFDFIQSIYEGDMSAEWLNMDPSLCNGISLMVKPYKFHVPVGKTVHSPLPQCNDVENNHVLSVFCLNPQFPDDYIFSTARLSGACCLFRDPQPVLKPKDWDDDRDGRYRPVTGFVQSAVTAQLNRASKRILE

**XRN-1**

***Brugia malayi* XRN-1**

MHLIKFDFNFQEKLPFDYNLESVIDDWILMGFLIGNDFIPHLPHVHIHEDALPLLYATYNEVLPQLDGYINEAGVLNLKRFQIFLKNFAKNDKKNFLEQMDDEAYLSSKRTSNAWKKDAFTSNNDEQSFNEDLEVFDLSNDETNKVTDIGITKGAFDSDSNASDECEEITDQRTLTSTKAVSRKFSNTYEYVRAKKKKSISMLFN

***Ascaris suum* XRN-1**

MEINFELSAPFHPFEQLMAVLPAASADCLPVPLQELMFDESSPILEFYPRDFETDLNGKKNDWEAVVLIPFINEKRLLDAIASKEARLTDEVSATNL

***Caenorhabditis brenneri* XRN-1**

MGVPKFYRWTSERYPCLSEVINESQIPEFDNLYLDMNGIIHNCSHPNDDDVTFRITEEEIFVNIFAYIENLYNLIRPQKVFFMAVDGVAPRAKMNQQRARRFMSARTAQTQLDKAIEKGEVIPTEKRFDSNCITPGTYFMTRLHDQLDEWIKKKSESDSHWQGRKIILSGHNVPGEGEHKIMDYIRTERAKSDYDPNTRHCMYGLDADLIMLGIVSHEPHFSLLREEVTFNTRPRKNEQKNKKPKRTDSDVKTFHLLHLSLLREYLAWEFADVKESLPFAYDMERVVDDWIMMGLLVGNDFLPHLPSIHIHDDALPLLYSTYKKVLPTLDGYINESGYLNLARFEKFLAQLALNDKNSFMDKLEDEQFMASKRLPRTSQPGDSSEEASGEEELVPFNSSDIEDNDEDDEKGTDESDGNGDDDEAAFVSDHEEEDEVEGKSSGEELSPSNLDALLLEEEFNDELATLALSGMDDADFANNVEACWTKTLDNQFKRHKKSYYADKLRYKNISKSELRAQAEGYVRAIQWNLHYYYHGCVSWSWFYPHHYAPFISDVQGFADMKIEFELSKPFHPFEQLLAVLPEASADCLPRPLQELMSSDPAKSVIYDFYPSKFETDLNGKRNDWEAVVLIPFIAENRLLEAIESKRSRLTNEENMRNTHGCHIQVVSNKSADNKWTVTRTELPQEIFRIPKEQVKWGLLPNVKMDVYFPGFPTMKHLNHTGELRFANCNIFGMSSRKESMVLKVENDSNIEKDIIEWGSELCDEEVCIDWPILKLAKVDCIWGGDDKIVRKIDGDEIIVKDMSEEEKKQWIAHVNQQTERQMTRFAIECAGQKDAKGKHIRPPIVWVRKFTGLVYESHQENGKAPILKAVKQWSSPQSTFPVLLPLVVKDVLLENSRNLADLPVRLAYPLRAIVWMNDPSTALYGMPGMIDGYQNENSSNCRIQVVGMSQDNRIAQMEELRKKAEQKSLRWMGGYDWARQCHVDPRLFARITGTMFMWNEPRERVEKGQQLSSESKINCGLSLKFSKRDFCIADYTDRVEQTNAKGTMKVWLYSNLTCVLVNEYKNAFPEVFKYLESAGSTQMDDVYYAEEIWKDERERSKRFGELKDFLSNLPSQEAEQLKCGTEYADRRMIEAIEKALKEKEVVPRIMHRYFPTPSGVFRYELYNGKVNVDPEADFLILDRVAMMSSDTKKVEKALEGAYTPMPQKAWQPKEQQKEQQKKVQQPQNQKKNQTQKVSAQPSTSSTSNSNSNLTQNSKNSKKNQKSQKKGKPEKVTVAKRNSDDTPEEELTSSLNKLLNIKSSAQETTTSAAAPSTSEPEPTKPLSLVELLGGGAKKAEAPPPPIKKRELPKKPEGSLSLEEIEAAHKKVEKKKEEKKPMSLLEQLAAAQNGKKNQKKDSKSSSNDSSKNSKGSNRNQKNQKQQPQPKYEILRKPSPTPNQQSEQQKRASPVEPPQQQPSQNRVSPVQSQQNRKPSPLNSQRYSPVQNQQQKSGSPLQPGYRGGSPVNRGPSPPQHPLPPHLPQLPYGMKPPHLPFDFKVPPPPPQFRVPPMGMFPVMMPMGGMPIHPMGMPPQPHYQQHNQHMGFDGKRSNPSLTDFKPSAISRRSNRSTNQPPKVTMITKKSPRPATPPAATSSSEAVVVKKETVEKPGDTPENEESKKTKKPRKKKQSRLAINFDKPSSSS

***Caenorhabditis briggsae* XRN-1**

MGVPKFYRWTSERYPCLSEVINESQIPEFDNLYLDMNGIIHNCSHPNDDDVTFRITEEEIFVNIFAYIENLYNLIKPQKVFFMAVDGVAPRAKMNQQRARRFMSARTAQTQMDQAIAKGEVLPSEKRFDSNCITPGTYFMTRLHEKLDAWVREKSVSDTRWQGRKIILSGHNVPGEGEHKIMDFIRSERAGTGYDPNTRHCMYGLDADLIMLGIVSHEPHFSLLREEVTFNTRPRKNDQKKKPKRTDSEVKTFHLLHLSLLREYLAWEFADVKESLPFEYDMERIVDDWIMMGLLVGNDFLPHLPSIHIHDDALPLLYSTYKKSGYLNLARFEKFLAQLSLNDKNCFMDKLEDDQFMESKKLPRNGSGEEEEELVAFESSDIEDEEADGEKEDSSRNNDDHDAAFVSDHEEDKNNGTGKTSAEESPDYDAQFLEEEFNDELATLALSGMDDADFANNVEACWTKAIDNQFKRHKKYYYSDKLRYKNISKAELRAQAEGYVRAIQWNLHYYYHGCVSWSWFYPHHYAPFISDVQGFADMKIEFELSKPFQPFEQLLAVLPEASRDCLPRPLQELMSSDPAQSAIHDFYPSKFETDLNGKKNDWEAVVLIPFIEEKRLLEAIESKQNRLSQEEKMRNTHGCHIQVISTRQGGGEWKMDRQELSQEIFRIPKEQVKWGLLPNVKMDVYFPGFPTMKHLSHSGKLSFANCNIFGMASRKESMVLKVENEKSDKDIIEWGSELCDEEVCIDWPILKLAKVDSIWGGDDKIVRKVEGDDDIIVKDMTEEEKKQWQSHVNQLTERMMTRYAIEIAGQKDGKGKHIRPALAWVRKFTGLVYETNEGSDSEGPTLKAVKQWSGPTSLYPVLLPLVVKDVLLENSRFLADLPVSLAYPLQSVVWILDPKASLFGMPGMVNGYRDQDKSQCCLEVIGMTDDNRVDIMENLRKDSDRFTLRWMGGYDCARQCQIDVRLFARITGTMMLWNEPRESVETGQQISSDSKINCGLSLKYSRRDICLADFTDRIETVNARGVTSKVWFYTPLTCALVTEYKKRFPELIKYFESVGMSQTDDVYYTEDIWKNEKQRDAKFAELKEFLSNLPSHDAEQLKCGTEYADRQIISEIERRIHQLPPKKKRAMTRYFVPPSMVFRFELYDGKVQMDPEADFLILDRVASMSPDTKLPRITQGTVVGIHDDKIDVLFDDSFEGGNKVRGSTLASACRISRNALVNITFGIMRKKTQKKKPEIQALEGGYSLLKPKNAKPQDKNKEQQSTSSNPKDQNQKNSFKKEKNQKKEKKFDNKKQNENTPETLTNSLNELLKIKPRNAVPVEVESQSTSEAPSTTTGKQVSLMELLGGSGAKKAATTNPKIPKKPHGSKNLEEIEATSSGAEKESSGSLLLQQLTAAQNGSKNQKNPKHQKKNEDGQQKKNENSKNAKNSKNSKGNKNQMQQKVEILRKPSPTQGGSARASKTPSPPVVNEKKSSQQKNPPPPPSAQQKPHQEPTDQKRKTPSPPLVNQPQRPFPSAQLPGHFQPHPMGMFPPGMMHPMGMHMGMMHPHHPQQHQQHQGYDGKRQNPTLTDFKPSVISRRPNYRNQPPKVTMITKRKSPTPPDAPKVPVVETAPESSEEKTKKPRKKKQSRLAINFSNPSPST

***Caenorhabditis japonica* XRN-1**

MKTQVCIHRFFGNQNKNHKSHSHNFQSSSLNDNELLEQEFNDDLATLALSAMTDDDFANNVDACWTRTLNNQFKRTKRSYYAEKLRYKNISKAQLRGQAEGYVRAIQWNLHYYYHGCISWSWFYPYHYAPYISDVQGFTDMKIEFELSAPFQPFEQLLAVLPEASKSCLPRPLQELMSADATKSEIYDFYPENFETDLNGKRNDWEAVVLIPFIDERRLLSAIESKANRLSKEENDRNTHGSHIQCISSKNGQGAWVSARTLLDNKLFIIPRESVKWGLLPNVKMDVYFPGFPTMKHLTHTGELRFANCNIFGMASRKESMVLKVDDSKQKDVIELGSELCDQEVCIDWPILKLAKVESIWGEDKIVRYSPNDDNIIVKDMSEEEKRQYVSHCQQLAEQLMTRYAIEIAVNKDEKGKPIRPPIAWVRTFLGNVYETVNEPNGPVLKAIKQWSSDKQVKAVLLPLVVKDVLLENSRSLADLPISQAYPKQSVVWLTDNKFALYGFPGMVCGYQKENTADCRVEVVGMAQPNNVQKMDALKKRIPQKSLRWQPGYDSARQCSVDSRLFARITGTVFLWNEPRDVVEKGQQISSDSKINCGLALKFSRRDLCVADYTDRTEQMNHKGYTNLIWFYSNLTCRLVTEYKKKFPEVFKYLENAQSTVMDDVYYAEDIWPGKQRDERFAALKEFLESLPSRDAEQFKCGTLYADRQLVAEIESTLTEENTKNDSEMTAQELAEKRKKQPAMMKYIVSPSVLFRYELYDGKVSVDPGADFELLDRVACMSTNIKVARTVQGTVVGIHGDKIDVLFDKPFEGGSHVRGSKKASAYRLPRSALFNVTYGLVRKGKESETQRQTALLEKTYVPMAPKKTSEKENKTEKPSTSSASASTSSNSQQKLSKKQKKQEKVQIATKKSPTDSESVEALTSSLNKLLKIKKNPENSEVSQGPSTSTQGHVVKGKPVSLMEMLGGGAAKKPIKLEGLEKPVQKKNQKSSILDQLRDAQKPNEQNKKTKEAKSEKKKKVQVLQKRVSQEEAPPTSKTPPTPIKEPTLPTPSNQNAPPPFVPFGMPPMHPMFPGPVGAMGLPLVPIGMPVSPQQMHQIHLMQMMDEQHRQQQQQMQKHNLFKYFYSESKSAVYISG

***Caenorhabditis remanei* XRN-1**

MGVPKFYRWTSERYPCLSEVINESQIPEFDNLYLDMNGIIHNCSHPNDDDVTFRITEEEIFINIFAYIENLYNLIRPQKVFFMAVDGVAPRAKMNQQRARRFMSARTANSQMEKAIANGEILPTEKRFDSNCITPGTYFMTRLHNKLDEWIKKKSASDSHWQGRKIILSGHNVPGEGEHKIMDFIRTERAKSGYDPNTRHCMYGLDADLIMLGIVSHEPHFSLLREEVTFNSRPRKNEQKNKKPKRTDSDVKTFHLLHLSLLREYLAWEFADVKESLPFAYDMERIVDDWIMMGLLVGNDFLPHLPSIHIHDDALPLLYSTYKKVLPTLDGYINESGYLNLARFEKFLEQLSLNDKNSFMDKLEDEQFMESKKLRPSQNDIFQEASVEEELVAFESSDIESDAEDKDDEKNETDENGDDDAAFVSDHEEENESEDKLSGKELSSENSDDVLLEEEFNDELATLALSGMDDADFANNVEACWTKTLDNQFKRHKKSYYSNKLRYKNISKTELRAQAEGYVRAIQWNLHYYYHGCVSWSWFYPHHYAPFISDVRGFANMKIEFELSQPFHPFEQLLAVLPEASADCLPKPLQELMSSDPTKSMIHDFYPANFETDLNGKRNDWEAVVLIPFIEEKRLLEAIESKRNRLSREENMRNTHGCHIECISTRQDDGQWSVIRSELPQEIYRIPKEQVKWGLLPNVKMDVYFPGFPTMKHLSHSGQLKFANCNIFGMASRKESMVLKVENEKSEKDIIEWGSELCDEEVCIDWPILKLAKVDSIWGGEDKIVRKLPGDDEIIVKDMNEEEKRQWQAYVNQLTERLMSRYAIEIAGQKDSKGKHIRPPIAWVRKFTGLVYETKEGEDGSAPTLKAVKQWSSPQTLIPVLLPLVVKDVLLENARLLADLPVPIAYPQQSVVWITDTRVALYGMPGMVGGYRNEKTSNCQVDVVGMVSDNRIAVMEELRKQVDQKSLRWMAGYDCARQCQVDVRLFARITGTMFLWNEPRARVEKGQQLSSDSKINCGLCLKFSKRDMCVADYTDRTEHTNHKGMTTRVWSYTNLTCRLITEYKKKFPELWRHLESASSTQMDDVYYAEDIWAEKVRDHRFGELKEFLDNLPSRDAEQLKCGTVYVDRQMITEVEKVISADPVKKPVMGRFYVSPSILFRYELYDGKVQADSEVEFLILDRVSIMSSDTKVPKVVQGTVVGIHDDKIDVLFDREYEGGSKVRGSRLTSAFRVPRTALFNITFGLIRKNDQNKKKVDQEFDGAYTPLPQKNSKHQQKNNDWQPSTSTPSTSSSYSNSKNQKNNSKKDNQKQSKKEKKCENNVMKRNSVETPEALTNSLNKLLKIKSGNPQAAAASSSSATSSEVPTTSGKQVSLMELLGGSTSKKNTQSGPQMLKKPDGSKNLEDIEMSNKNQKQEKKPANSILQQLSDAQSNSKKQKHQKNQKEKKSDDWQKKNDVSKASKSSKNSKDHNNQKPKVEILRKTSPTQQEQQHAIHQTHPEQQQHAPQFKTSSPQLAHPQPSGPFVMSPPNFNFSVPPPAHFNQPPIAMFPGMVQMTPMGLQMAPIGMVGQHNQHNMGYDNRRHNPTLTDFKPSAISRRPNHRSTNQPPKVTMITKRKTVTPPPLPAEVDKSVENKANTPPASSEEKSKKPRKKKQSRLGVNFCNPSPST

***Haemonchus contortus* XRN-1**

RANPSQTKFHLLHLSLLREYLSWEFDPLKLDDDQEESELCEALMAAELREMDDKLFENDVEVRCWFTFEREKYIYFSARTAAEQEQAHVRKGGKLPTEKRFDSNCITPGFELTGTVFMAELHQALSSWLNVKIDKDPLWHNIRVYLSGHDVCWTKTVSNSFRRHKRSYYREKLQLMLGICSHEPHFSLLREEVKFSRPPSKKLSLRLIIGYINESGILHLKRFEVFLKAFAANDRKHFLQIMEDESYLRSKRQKLFNIIRPQKVFFLAVDGVAPRAKMNQQRARRFMPGEGEHKIMEFIRHERIADGYDPNTRHCMYGLDADLVGYFCSMLSYSSHQIPEFDNLYLDMNGIIHNCSHPNDDDVNFRISQEQIFCDIFAYIDVRFSISLPFPYDVDRIVDDWVLMGFLVGNDFIPHLPHVHIHDDALPLLYQTYIQILPTLDGML

***Oesophagostomum dentatum* XRN-1**

GLVSWDWYFPYHYAPFASDFDTVSQFKPDFSRKTQPFKPLQQLMSVFPAASKQHLPIHWQKLMTDEDSPIIDLYPVDFRIDLNGKKYAWQGVALLPFVDEDRLLRALEAVESTLTDEERARNTTGPNRIFVGRNHPAFDFFQELYTNTNGGAKFVDIDPAFTYGVTGRISADDTAVAPDQPFPSPVQDKTCPDLAVNGAVMVIYEDPQYPEGFIFPQSA

***Pristionchus pacificus* XRN-1**

MGVPKFYRWLSERYPCLSEVITDAQIPEFDNLYLDMSGIIHNCSHPNDDDIHFRISEEQIINDIFKYIENLFNIIKPQKVFFMAVDGVAPRAKMNQQRARRFMSAKNADALIEKAKRAGERIPTVKRFDSNCITPGTQFMVKLQRRLDEWVQMKVNTDSRWRRMRVYLSGHDCPGEGEHKIMDFIRAERAKEGYDPNTRHCMYGLDADLIMLGMCSHEPHFSLLREEVKFTRPSKPGGRKKKAKKTESNTICFHLLHLSILREYLSWEFIKVKDSIKFDYDMERIIDDWVLMGFLIGNDFIPRLPNMHIHDDALPLLYKTYMDVLPTLDGYINEDGHLNLHRFQEFLTAFSRNDRNSFLQVMKDEEYLASKMGATGIDDDIESTVKNTIGYCGLRRTVLVVVICY

***Trichinella spiralis* XRN-1**

MGVPAFFRWLSRKYPSIVMNCIEDTPRDVDGTTVPVDNTQPNPHGIEFDTFYLDMNGIIHPCCHPEDKPAPKSEEEMMVAIFEYIDRLMCIVRPRRLLYMAIDGVAPRAKMNQQRTRRFRASKEAAEKEEQIRQIREDLRAQGIPLPAESTDKQHFDSNCITPGTPFMARLAICLRYYIHERLNTDPAWQNLLVILSDASVPGEGEHKIMDYIRHQRACASHDPNTHHVLCGADADLIMLGLATHEPNFTIIREEFVPNLPRPCEICNNYGHTMQDCQGLSILENENEEAHRPVLKKTQFIFIRLSVLREYLQRELEMPNIKFKYDFERCVDDWVFMCFFVGNDFLPHLPSLEIREGAIDRLVKLYKDCVYRTGGYLTENGFVNLKRVQLIMSELGKVEDEIFRQRQEREAMDNIFLLDWIQFSNKAKMRRMQAENFDAPAFIPQNAFAPTPIGESPLPLSNAKRTAMEMRQAAMAVTSSTKREFNGAANAEDANDEGPLDEVRLWECGWKDRYYLVKFQCSPKDLEFRHHVANCYVEGLCWVLRYYYQGCCSWKWYFPFHYSPFASDFLNIGDLKIDFSEKTMPIKPLEQLMSVFPAASSKHLPKSWAALMHDPVRDKSTIIDMYPSDFKVDLNGKRYAWQGVVLLPFVDAERLNEALEVVYPDLTEEERFRNKQGNDLLFISSKHEAFDFIQSIYEGDMSAEWLNMDPSLCNGISLMVKPYKFHVPVGKTVHSPLPQCNDVENNHVLSVFCLNPQFPDDYIFSTARLSGACCLFRDPQPVLKPKDWDDDRDGRYRPVTGFVQSAVTAQLNRASKRILE

**ADR-2**

***Ancylostoma caninum* ADR-2**

GERPATSYKKVAKMDCFVKGIKVLCDQFSVEIAKSSQDLEFKQDTTFFELLRKHTYSKFYKLSSVRPETILSEKVIASIFLVTRTNQSSRAEIIALATGNKGLRGDYLSLHGCSVNDCHAEVITRRGLLRFLYAQTMLYTKNPAKSIFVKNAKTGKLALRKGLSFHMFVNTAPCGDARVYTLNDTTLVNANEAETNSLLRFKVENGMGTVLGRYPESLVTQTVDGIAGGERLRTMSCSDKMMXGTSWEFKGDYFL

***Ascaris suum* ADR-2**

MCARQRVVYGRVYRERLACGGVMSTRPSQDPPKRYLGPIIFGNCCAARDIRYYKQYAIAAKIESLEALGLRKLSIVKRFGLIFLYQQLEVYLRDEQKSIFRKVMGRLALRPSLTFHLFINTAPCGDGRIFSFSSEWSL

***Caenorhabditis brenneri* ADR-2**

MDLGTSSTPTAANSTIDSMMVEENVPSFVRETVERSGKNPMSLFSELYIQLTTHQPDFEYYIRNQPDGSTKFVCVTILDDEKIEGQQKNRKKDAKLSCSLKGLEKILSHVSESMVPEVTFQEPTTFFELLREHTYAKFYELCKNNASIYGFEKVIASVFMKFNEKLILISLATGNKGLSGDKIRSDGSALIDCHAEILAKRGLMRFLYSEVLKHSSTPIKSIFLESGGKLILKLGITFHLFINTAPCGTARVDKKMRNGNPEEIKSSSRLRFKIDKGMGTILGGDREFEDPQTFDGIMVGERMRTMSCSDKLLRTNVLGVQGALLSHFMNPIYYSSIAVAEKSNFERMNRAVNDRAAGFPAQLPFRVNKSIIGECQVEDTEQSTSSTARSTIGSMNWNLADGTTEVVKTSDGLVHEKDMTGADVEKPSRLSKKKLAELMKTVCTLAKIEIETPASYEELKAGSVKYAATKKEFYAWMRRQDLGIWQQKPREFQMFTIN

***Caenorhabditis briggsae* ADR-2**

MAADISDIKIESSTNALQIPEGVPEAVKEAVERSGKNPMSLFTELYQQLVGNTPVFDYYVRNLPENRINFVCVVELDGQRVEGLAMSKKKDAKMSCSWKGLEIVLKLVENTKAPHVKFMEQTTFFELLREHTYAKFYELCTMETEIYGYEKVIASIFLQINDKLQLISLATGNKGLRGDQITSDGSALIDCHAEILARRGLLRFLYSEVLKFSGSATSSIFETGKIGKLALRQGISFHLFINTAPCGTARVDRKVRTGTAEEEQALSRLRFKIDKGMGTVLGGAEEFADLQTFDGIMSGERMRTMSCSDKLLRANVLGVQGALLSHFIEPVYYASIAVAEQNNFVRMNKAVYTRASSFKPPAPFRVNQVKIGECQQEDVEQSTSVSARSAVGSMNWNLADGTVEIVRTFDGKVHEKDMTGADVEKPSRLCKKSLVELMVKTCFLTKTPLDESMTYEEMKAGCLDYSAAKKSFIMWLRRQDLGIWQQKPREFEMFHVN

***Caenorhabditis japonica* ADR-2**

MSEEMQVEEAKPNQIGLLMAEENVPDYVKETVERSGKNPMSLFSELYVQLTSEVPTFEFYVRNQNDGTAKFYCSTELDGERIEGSTKSKKKEAKLSCALKGLAVVLKHVSESVIPEVKFEENTTFFELLREHTYAKFYELCKTNAAIYGFEKVIASVFLKFNDQLQIISLSTGNKGLRGDKVVSDGTALIDCHAEILARRGLLRFLYSEILKYSQDANTSIFMKVKGTQKLVLRPGISFHLFINTAPCGAGRIDNKAKDESVKTVNALSRLRFKIDKGMGTILGEADEFEDPQTFDGIIMGERMRTMSCSDKLLRANVLGVQGALLSHFVEPIYYSSIAVAEKNNVERLQRAVFGRAASYQPKKPYRVQSVVIGECQVEDVEQSTSATARSAMSSVNWNLADGTVEVLKTSDGKVHDKDMAGVDVERPSRLCKQQLGELMVHVCAITQTKTDQPVRYDELKAGCKSYETEKKAFIAWLRQQDLGIWQKKPREFQMFNVNC

***Caenorhabditis japonica* ADR-2**

MAEEMETESINSNGDQPQAPPTPRIKEVPEGTPDFVKETVERSGKNPMSIFSELYVQLTGNQPLFEYYVRNQPGGGIKFVCITMLDGQKIEGEQMNKKKDAKLSTAWKGLEILTRHASDSMTPEVQFQMETTFFELLREHTYAKFYDLCKSNASLYGYEKVIASIFIQNNENLQVISLSTGNKGLRGDKIVNDGSALIDCHAEILARRGLLRFLYSEVIKFSTSPELSIFERGTEKLVLKKGILFHLFINTAPCGTARVDRRMKSGTAEEIQATSQLRFKIDKGMGTVLGGADEFADPQTFDGIMMGERMRTMSCSDKLLRANVLGVQGALLSHFIEPVGGYYSTIYYTSIAVAEQNNFDRLNKAVFARAANYQPPAPFKVQQVIIGECQIEDIDQSSSVAARSSVGSMNWNLADGTTEVIRTNDGLVHEKKENGVEVETPSRLSKKMLAELMMATCKLTQTAVDSPITYDELKAGSLEYLNAKQSFIKWLRQQDLGIWQRKPREFQMFRVN

***Haemonchus contortus* ADR-2**

ICSDLGRPVPKGYTYENMKVHCQAYEQGRISLETWVNWNIADDSIEVLTYRSNIYHRFLYAQVLLYTQDASKSIFVKTNELGISPLRDQKLGMWQSKPEEVSMFVVIIGSEKVIAGIFLVTRNHHGSSADLVSLGTGNKGLRGGDMLSLHGLSVNDCHAEVVARRGLLRRVVFSNNIGFLEFKQETTFSELLRKHTYAKFYGLELNLPFRNRENTTLVNITIEFMVNGHLIYLSSISVAEKADKSRLERALFRRVEGFVPVAPFRVNKPYIGHCQVLALRKGLSFHLFVNTAPCGDARAYVLNEDGEQINEIESHSNLRFKIESGGMGTILGRTPEVLGPQTIDGIAGGERIRTMSCSDKIMRWNVLGIQGELLSLLIDPVCFRTLVVGNEEGHSLEHMHVQFQEERYGVFAGTSNHLPQIFTHVLRNGRHSHDFSLPVKLRVGVFTKKFRERSFLFEFE

***Meloidogyne hapla* ADR-2**

MSSTEKMPTEPTQEAEIAGDSQTTAYEYYKRLVDDLNITGAFYRQQDVCQAAKDYITSTNKDPVSLLNTYCTISGEVAFKFFEYELAGRPDKNKFLIVSEFDGQPIFGFPSPSKKEAKKSCAVCILEKLFLEQRLNCLGSNGRKVKHPNRHPRPFPFTPVPKQEEDLGKIGAEFYQQPLSVDPQMAMFAEDIDFDGPVGPKLLEMKQNFEEQNPLPADFKDKEKFEDVRNRAFKLFCENAFWVELKLCDATIEKLNAAGMWHENWNSVAYVNQSIDRLNSWRVKEMNELKQQQEEKMIGGQEKLERMEDKEVKKVEPPEYFIFTLPYLPPRYACVALFNEKLFHGQISVSKSKAKSSCAVVIAQNLIELGWTDFNKKIVKHPKTVKSRPTQKKRKKETSEEDLVSIETNSSSHSIDFQPYQFLLTLHPNEDLDPRTHFLLATHGNKNATALLTEICAKYRLGALSYNETNEMVVGAALSPRFTTDAQISPYPIPGVPLEVLGGPLFQARGIPEKNKKNAKASASRAALDILVRKGTIQINEQNFAEFKASIKNAIFCEESPPVIREGAPMNENVNGAENDNTNS

***Oesophagostomum dentatum* ADR-2**

NPISLFLEMYMQIFKESPKVFVQNIPGDTKGSFLFVTSVRIGNNEVTGLPHRTKKVAKMDCFVKGIKVLCEQFSVEISKSSRDIEFKQETTFFELLRKHTYSKFYGLSSSQPESILSEKVIASIFLVTRTNQSSRADLISLATGNKGLRGDFLSMHGCSVNDCHAEVIARRGLLRFLYAQVLLFTQAPTKSMFVKNAKTGKLALRKGLSFHMFVNTAPCGDARVYTLNDTTLVNANEAETNSLLRFKVENGMGTILGRYPETLVPQTIDGIAGGERLRTMSCSDKIMRWNVLGVQGGLLSLFIDPIYLSSIAVADKADRKRIERAFFGRLEGFEPPAPFHLNKPFIASCQDEPYTRDTTAGSPISVNWNIADDAVEILRTSSGRIDASVPKEISRLSKKQISELFKKTCDVVGHSVPSGFTYENMKTHCKQYEQAKLALNNWLQEHKLGMWQSKPEEVSMFTV

***Pristionchus pacificus* ADR-2**

MFRTISLQLGPTEAFLAAECDEIRADLNGKELRKPAISLLFEILAGTFKCFSTITTGGVAGAFSASLVLGPSTISSCEHKNKKDAKNECARRAIRALLTMSVDSVREVKKEAADDTVARTYWDAVVKRKAGTVVKTEEDTVARTAWDAAVTFEELDTVARKTTVFKIEEDTVAREGTVSIEGEQRKGGCFGYQCDVQRPRADGAMAQTRLSNLMQEQAYALFHKLAETCPAAANTHRVLAAMFLRDQTTARLHCVSLATGNKCIKATSLSFDGCAVNDCHAEILTRRGLVRWLYTQVQLSLTSPGKSALVREQGEEGGKLRLRKRFSLHLFISTAPCGDGRVYQFGSTKNKDYRNVGRLRHKIEDGEGTVLGEAADERLSIDSFAMGQRLRTMSCSDKVLKWNVMGLQGALLSHFLHPGKFIVIKLLICLSYGIYGIPQIYLSSLALAHFTRESCIARACYGRVARFKPSEPEYTVNSSLVLHRSTFVLPTTVSRARPKSSNVSANWNATDGEVELIDTRTGRALQAKDTGLGAVTSRLSKISMLTRFAALTPGAGSIKYKDVKRSATDYSKTQFELIRYLEEEAKLGQWQSKPNDFSQEDSVVSKLPIQSY

**ADR-1**

***Ancylostoma caninum* ADR-1**

KNTHQGKIYTHEGFGKRSTRDVVVREALLDLFNVPQSEIRRIVRRHLMGKLTDMPIIQTLYQVAHLCGCEVAFKVDIATEQKAVGHAAPTFVAVCTLTDHSMDDKKVEFTSPPSRSKHEAKEYAAHELLRSHFDIDPPAVAAERPPSVEQPVPPCQKLHVLLAKQNRSVTPNIKYEDLGLADDTVSQMGTIFKSKLIINDKEEFIGSGKSKKAAKNDAAMLALKKIFMFDYNNPESEPVIETPPRGRRGKNGSSQLCFDVAEYAKREYYSMCNFYAVKHSTEVAAFFLVNELDEKRLVAIGSSRPGTVDGQTVGTARGTAIIHFDPIILARRSLMRYLINEVGKVGDPKCIFVRGEDGMLRLNEGLRLVLYATYAPNCSYSCKEASVKKLSVLGPSHLIPAPDDVQSLADIQQTGRLNVHCAADKIFKWSRLGVQGALLSTLMHPILPHSVFFGSQAPVSDASLLFALFGRMEPPKNPCKVESIKNNIILSSSPAHVWTRDMEHVEMLNIDSGRTNKGSPSRLCKRLYTRRSLNWPRRI

***Ascaris suum* ADR-1**

CHEARAQARVRLKHYQNLYRPLGYKVVLYAAFPPLIGRPSGSGKQKLMCHVGNIRARDVPDNLQTMDEIISTGQINVMSITDKMLKWNYLGLQGALLSYVLEPIYMTHLCIGSPTHDGHLMQAVLLRFGDARKNELVVKSVQKGIRPQGEMYHDWVDGVGTIERLDPSTGRTVTGSPSRLCKSELFESWSRVLAAMX

***Brugia malayi* ADR-1**

MDSHSYYGAGGCPSWLRIGSTSSDAISTITSQQQQQQQQQQQQQQRFYYPMMSLNNITKNKSNRVSGGVGTGYHNYQNHYPQQQHYSGTDPNTTGYYGSGFFTKHQLQHSTVSSRGKRERSYGSFPRKNRFDCGVDTTYAPPNKKSRTYSATGKTAAMILNELYPEFKDECSYRTLVVNKLPRFECSFVVQAKKFCAEGSNKKAAKQLACELALKELRPDIILDASVSISETKLADFDFCAQQANGSEEKERISNEKFIPQFTFSEIPTTDIDGKSSKKYKCMLVLPHQGKVYSHEGYGKSPTKNAVIREALVDVFDVPQDELKMVERRTMNLKPGKPMQVLIQALNFYDRTMQVDVDTVDGKPVQGNSRFICRITLDNNKTIVGPPQDNKQKAKDAACEKVLIEELDLTMPSEDVKIKKASSVLSPPYALHQLMLKQNRKKNPDIVYDEPQDISQAPNKPPMFKCTLTINGTHKFEGIGQSKKTAKSAAAEQALTKLFKFDLYAENALEMISMKKSRNENDVLFCTEICTFVRQEYEGVCHQQACPITTHISAFVLIAPNGEKQLVXIGAGRNAVIDGQILKDAHGNVLIHMQSTVLARRAFVLFLHGQIKNHDAENSVVERSPTSNKFRLKTGYQIVLYASFPPNLRSSGETQKLSCYMGGARLEDPPEIPQTFGDIGTSGHVYVMSLADKMLKWNYLGLQGALLSHLLEPLFITHLCIGVPSNDKAIAHAVLLRFSDVRRGELIVKSSQNGVVPHGEMYHNWVSGIGTIERLDPFTGRTVTGSPSRLCKSELYESWARVVAAIKADAYKPIWSCSEAKQSEVVYQQALQTFHLQLHENGLGMWQRKEPQVDGFQLAFFDE

***Caenorhabditis brenneri ADR-1***

MDPNLNYNFAYGDNYSGADPSNDPTNYQAWASSWTQPTSAATQGSSNFQSYPQYQSQHLQQQQQQAQQQAQNTYAALNPISTFMQQQQKPFQQQQQKKFGGQPQGGSFPKPGVNSRNFGAFGAINNTSGGAGGSGGGGIGPEWVQPMSQQSMMSGGGVGGGGAINPALRTFPGKNGGGGGGFQKPQWRQNKQGKMSNGPKKFDTTGKTPAMILHEVFKQVNEEYTEVQDTVPKRFRCTLTVEGRSFQMESPNKKAAKQKCAEVVVRELRPELHVTPFEEGINAKATPVKKSNGESNKRNAAEMMNQPPAQAKKTPVSAKKAKLPPLEAANSLLDLLMKLVTESEGKYKPVFEATELPREDDPEGDQEVKLEEQEELKQEVNEEPVEEEVKQEPMDQDVKPDITQQQKLSKKEKAKIKKPEVLHKCVLRFEEQGKEYTKIGPNRGNNKDFVVREALRDIFQVSQEDITAIARRHAASRLGSDMTILQCLNTIAAVLNCKVTLEAEPAEDTPIGVGRMHFMARCTVIDHNENDRQVETTSPSLNGKGMAKEYAASEMLKTYFKIDPEQCIKPGEGTNTQGPCATLHAMINKQSKTKEQIVYEFKENVPAVAGNPATVFYCDCIVHGERHTGSGRSKKIAKNEAAVLALKKIFKIDYDPNTVYPLALSSRAMTESKISPMCKNISEFCKREFHGMVTEANMHSGNQMAAFILVNEKDEKRLLSLCTSGQYIMEPDALALSNGTVLNHLEAIVLARRALIRVFIAELAVLNENPDACIFERRPDGKAALRPNFRLMLYANWPANCSLSVDDAQKKSLGVVRPTSTAPVPDDVLTAEQMREQKTFRVHCVADKIFKWNNLGIQGALLSNIIEPIFINAIYFGSSCPVTDESLKYALVNRLGDNESDRDVIVECIPAQLRPQQTYSQVWFRGSSGIEILHVQSGRANNMAPSKVSKISIFEEYHKLPDADKSIVNYAKAKENASTYQYEKNVLYGKLEAAGLGKWQVKPAEFVDSFTLPEYD

***Caenorhabditis briggsae* ADR-1**

MDPNLNFNYPYSDNYSGSGAATGDTNDSSQQFGWASQWTQPASATSQGAGGYPQYVSQHQQQAQQQAQQAYAMMNPLSPFMQSQQKPQQQQLQLQQQFQQQKKFGQHGPVGTPKAPGSQLGGAFNPRAFGAFGAVGGGGDRSTSSGGILGQEWTGEAANQFGMQSHLRTFPSKSGGGPGGHSKPNWRQQKQQRQSAGPPAPKKTETAGKTPAMILHEVFKSITEDYSEVEGAVPKRYICTLTVNGRQFQMESANKKAAKQKCAEAVVRELRPDLHVTPFEEGVTAKATPPAPPKSAAGNGQPNKRNAEDQTNQPAAKKTAAPGAVKKIKLTPVESAQSLLDFMQKTIAESKEKYTPVFEAIELPRAEEMVEVKAEDMKQEPTEVTVDENAEAPAAGAKRSKKEKVRKPEVQHQVTLKFLEQGKEFTKTGPNRGMLKDMLIREALREVFNVPQEAITIVARRHALNRLGTDMNLVQCLQTIAGVLNCKVSLDTEPAEDRPIGDGKMYFMGRAKIIDLNEDGKEFETKSTSVPSKALAREHAAMEMLKQYFKMDTDSIQTNEAHGNSSGQQGPCAVLHAMMNKATKQQTKIGYEFKDNVPPVAGAGSPGPVFYCDCVIDEERFTGSGRSKKLAKNAAAVVALKRKFSVDYNPDGMCPLAHSSRPERQCSPLCREISEFCKREYYDMTRHYGVNQSNQIACFVLINDRNEKRILSIGSSIQYVCEPDSLNGADGNHIVHMDPIVLARRGLIRSIYHELLYMPPEGSVVFEKQPDGRYALNKTMKLVLYSNFSPACRFSCDDVPKKSLSYVTPLTCKPVPEDVLTLDEIREKREIRIHSTADKLFKWNHLGVQGALLSHVLHPLFISHIFFGTQAPVPDDSLKFALVNRIGPVDAGADHQETELESVQGNMEVFTTRYSHVWSRGLDLIEQLDVSTGRTINGMPSSVCKAQLFANYCQLPHVTQKDVNYGKEKEASSNYQYEKKVLYEKLEAAGLGKWQTKPMELVDSFTLPASFN

***Caenorhabditis japonica* ADR-1**

MSKNGGFQHTQQAKKFPGQAGVPKPVNRTPFGAFGTENSQAAGTPNWGQAGQNRQFNRGGGQQKNFFSQFGAKPSWKPKGKPQEPKQRKFDTTGKTPAMVLHELFKNIGEDYEEVEGVPKRYRCTLTVNDRKFQMLSANKKAAKQKCAELVVRDLRPDLHVAPFEEGITAKAVPVVPKVENASVTAAAGNGQSNKRSAAPDAAKKPANAKKPKLTPVESALSLLDLMQKIISELPEKHNPVFEAVENPKPEEPKEVKTEVESSPAAEKKPNEKKGWGRKYSQYTVTLKFAEQNKQYSKVGSNRGILKDLCIREALRDLFKVPLEDIITVARRHASNRLGSDMNILQCLHTICSILNCTVTLETDFAEDKPIGDGKAYFQGKCTIIDLSNNDTEFTTTSPALHSKPLAKEYAAQEMLKSYFKIDPANCPKGDSLASQGPAAVLHAMLNKQTKQRTKVVYEFKDNVPPVAGQPTTVFYCDCVVDEKDRYTGSGRSKKLAKNEAAMVALKKIFSIDFDPNATYPLALSARAMAESKVSPLCKMISEFCKREYHQMAQYYHIMASNQIACFVLINENEEKRLLSIGSSIQYVVEPDTLNGANGTAIIHMDPIVLARRALLRNFISELEVLETNSEASIFEKKADGKAALKSNLKLVLYSNYSPVCSFSVDDAEKKSLAYVTPTSLAAVPGDVLSYESIKGTKTLRIHCTADKVLKWNAIGVQGALLSNIIQPVFISSIFFGSEAPVSDESLKFALRGRLVHGDEGRELAVESMPVQMRLHSGPSHLWIRGFDALETLDYNTGRTSKGSPSRLCKAELFEAYRKLATVNQSVIDYSKAKEQSSEYQYEKKVFYEKLEAAGLGKWQTKPAALADQFTLAAFDTV

***Caenorhabditis remanei* ADR-1**

MDPHLNYNFNYGDSYGSGADPTNDPTNHYGWASQWSQPASAASQATTGQYPQYVSPQQQQAQQQAQQTYAALNPLSTFMQQQQKPQFQQQKKYGQSGNGNAPKPVGAHRNTPHFGAFNSGSVGGGRGGPAAGSGSFLSQEWVQPMSSQNMMGVNPVLRTFPSKGGSFNQNNKPNWRQNKQQQKNSLGPKKFDTTGKTPAMVLHEVFKSVSEEFTEVENAVPKRYRCTLTVDGRQFQMESANKKAAKQKCAEIVVRELRPDLHVTPFEEGVTAKAVPVKPQNAGGAAGPVASGGNGTPNKRNADEMMNQPAQKKGSGNSAAAKKPKLSPVESALSLLDFLQKMIGESKETYTPVFEYSFEPPRSQEEEEPKAPEVKHEPMETEVVKAEPAEEVTENQPAVEAEQSDANQPATGKKPVAEKKVRKPDVQQKVTLKFVEQGKEYTKTGVNRAVVKDMVIREALQDLFGVSHKDITTVARRHATNRLGSDMNILQCLYTICSVLNCTVTVECEPAEDRPIGDGRMYFMGKCSIVDHSEGGQTFQTKSDSVQSKPLAKEHAASEMLRNYFEIDPNTCCKSENVNAQGPCAVLHAMLNKQTKQRTKIAYEFKENVPQVAGSSAQDFYCDCVIDENDRYTGSGRSKKIAKNAAAMMALKKKFNIDFDPNACYPLALSNRAIAESKVSPFCKTIAEFFKREYHKMCNEYSITPSTQTACFIIYNHLEAKRLLAIGSSPKFVVEPDTLNGANGTSILHLDPIVLARRALCRNFLYELAAMTEGSESAIFERREDGRFAMRSNMKLALYCNYSPNCFHSTDDAAVKSLAILTPMSLAASPPELLSLDEIRATKTLRIQCTADKILKWNTLGVQGALLSNIMEPVFITDIFFGSSYSTNDESLKFALYNRLGPQENDRDIAVESIPVDIRCQQSVAHVWARGFMDIEALDMNTGRTQKGSPSKVCKAAIFEAYRQLPIPDKSIVDYSKAKEHASSYQYEKKVLYQKLEAAGWGKWQTKPTELADQFTLAADEF

***Haemonchus contortus* ADR-1**

TLTFAEQGKVYTHEGFGKMSTRDVAVRELQTLYQVAHLCGCEVQFKVDISTDKAIGHVPPTFVAVCTLIDHSAFTLRSMSFLIKVSEYAKREYYSMCNFYAVKPSTEMAAVVLQALLDLFQVPQADIRRIVRRHLMGKLTDMPIIQVLFRSKLIINDKEEFIGSGKSKKAAKNDAAMMALKKIFMVPKHLIAVPDEAQSLADIQQTGRLNVHCAADKIYKWSKIGINEVSKIGDPKCIFVQTENGMLKLNDGLRLVLYATYAPNCSFCCPEASVKKLSYLKTAEVALRTLRPDLEITPFEDGVTSVPVKDGVPVTAQTAQRPVKAESEDAPPAKKIKVCRSLSDMGICRFSVNLLFLQLNALESALSLLDCMRKLCAERVSEGPYNPVFDFFLVNELDEKRLIAIGSSRPGVVHGETVATARGTAIIHFDPIVLARRSLIR

***Oesophagostomum dentatum* ADR-1**

MADGLLPGVSVRVQQMEETFQRSFGQNFFKKAAKKETDPTGKTPAMLLHEVFKEISENYEEVASHPKAYRCILTVAGQQFAMVAPAKKTAKQKTAEMALRTLRPDLPITPFEDGVTVQPVTAEGTVPVQPVARPAKPEGEDAPPAKKIKLNALESALSLLDCMRKLCAERVSEGPFNPVFDITDITESASTTGDKRKFRATLTFAEQGKVYTHEGFGKMSTRDVVVREALLDLFNVPQAEIRRIVRRHLMGKLTDMPIIQTLYQVAHLCGCEVEFKVDIATEQKAVGHAAPTFVAVCTLTDHSMDDKKVEFTSPPSRSKHEAKEYAAHELLRSHFDIDPPAVAAERPPAVEQPVPPCQKLHVLLAKQNRSVTPNIKYEDLGLADDTVNQMGTIFKSKLIINDKEEFIGSGKSKKAAKNDAAMMALKKIFMFDYNNPESEPVIEVVPRARRSGKNGNSQLCFDVAEYAKREYYSMCNFYAVKPSTEVAAFFLVNELDEKRLVAICSSRPGLVDGQTVGTARGTAILHFDPIVLARRSLLRYLINEVGKVGDPKCIFVRSEDGMLRLNEGLRLVLYATYAPNCSYSCKEASLKKLSVLGPGHLIPAPDEVQSLADIQQTNRLNIHCAADKIFKWSKLGIQGALLSTIMHPILPHSVFFGSQAPVSDASLLFALFGRMEPPQIPCKVESIKSHIILSSSPAHVWTRDMEHVEMLNIDSGRTNKGSPSRLCKAAVFEAFLKFAPEGMKCGTYMEAKQKAIAYNEAKRVLYEQMEAAGLGKWQTKPSKLVDFSLDSFDI

**LIN-15b**

***Caenorhabditis brenneri* LIN-15b**

MQTLQSARLTSKPLPLPLFFTNSVNEKLKDFPSRPQVKAPGILRQRTVVPVVTKVPTVDTNPKTCEAIAKFFASQGIAVEAVDQHAFRELIKFLDPDALLPDEDELGSFLKKYATTHKAGVNFSKTVGPLSVTIDVSGGDDDKFLVFSIHYFEDLRERKNIIYLRKLLLSELDPASFINSIRRAVHNHNYQNVKFSNIVCPSDEIYTLLQTTPVAKRVYMCCYQYMTKFVCDVLDIDEFAGGLEELREFVRFVKETSDHYAKYRRYQLGNNGDLEVPGVDDGEWSETVLFLTKCLVLHDTFKDFVKRHKFNYNYIEDDTFCYLVYLQRLLQHCVQLCRSMSAPNSSISQVVYVYSSLKSFLTENSMGYRFQPYIRQLVDEAFKDVNDPMNERRYNIATYLDPRYAYRDTIYSRDRWKTIEGWVTDDFVNYDPSLAECYSFDLAEYTTGELQELIRQELIHYRTVSFVERPEENECPFAWWGDRQLHFAILSVMSREYLACPAVSVDAASFFTFNGKFEHITSLYNTEMLQACLTVGGNFQEFRGRGTSENKITQEMVDTLKSTITRNLKGANFDLFTHHLAATEEERNRIITSDFPPLPIVPREHHKEYLPPQEANQPVVETITIIQPPPYPSKKIPPHRMLGKPIQLNGKELKAAIPPVRNLQQGKTLPPLPSTLALKKVVPITDHTPTGLNVKPPVKNVPSTSKAASVEEEKPPKDLLTLDKEVKEESQKTDLERVKEEIEDDHIAPLGPPEIEEQKPPQIHAMFPAKSPYLLPVKVCPADSQFVRKHPTTIQHIQPHNFVQKVVVGKQNFVPKFPVKVIQPPPLPPPISIRAPIPKAPIVRTVVMPVKRVVEPLKDPKQDEEEKKPAEKDLLLESDVKLEPFDDFTPQSNYNNSYVPQTSKIYADVLNERSAAQAFERHRMNMEYHKRKPCNRRCAVCGNLEIHDLLKNVTIDNEKLLIMIGCVYRGEFSLRAAQEFMARETKTYICRVHFGETLDEIDQMLDLKDGDSVLNACSNSIQNVMMTAKQLRPHVTALSLRNMMNEFVDRNCHLRRTRNEHFNENFDFYEQPVLEEERNDVDDEAIIPKVYRQPRKQVLEADSHDGTVKVIEQEDFKLPTAKQTGNEEVQNPGVCCYCSKRGERNTMLRVPRGEDRLARWIEKLGEEFEKRIMAEEESLICRSHFPDNAFSSRGRLLKGMTPVAAPEKVVCTYSIQGNDFIKVREKKSGTDKDARVNLDSNPNGTKPFWINQYAGESSEEEEPPLPVAVPRIPKSQKKSGRPRGRPRGTTKLSIQAKLAAASRFSQRLEKLEGAVSDDDDFFYDSEFDDFEGPSDSSSEAKCNVPRLSTSPVDALLDEDDTDYALPAHFQPSDGTTRKRGKTRKDIKEEEVEDPTSRRNSTTSTQSASSRQGSVEKDESTKPKKTATRRSVPRKSADEKDEDYSVESDKPKPKKARYNVRIGGKFAKRVIEEPAIAE

***Caenorhabditis briggsae* LIN-15b**

PPPYKASGILRHRPPPAARAEEQQQLETDPKTVELLARFLVSQGLPLECSKEPAFLDLARHLNSRMVIPPENVMNKFIDRAAQIAKPLVNFQKTVGPLTVTLDIAGDDDQKYLVFSIHYFEDLYERKNIVYLRKMYLRFIEAETLLTSIRRAVNNYTYGTVKFSNIVCPNESILEMISNSGVVKRYYVCFQNYMTQFVHILLKIPELAQGLNQIRCFVKYIRQDADKYSRFRRMQLSRSAEMDVPKLDKESWESTSVFLTRCLVLHDTFVEYCQLMNLDPYISNKTFNHLIYFQRLLEQCIKYSRELSQSNNSMSQVLPAIISLKGYITTNNLGYPFQRQIDETAEKIFGPITYGDSRGLYEIASLLDPRYAYGDCFPMEQWKMLEKRVIQEFVGADQRLEKCFYHDITLMSNTERMKVLGKEFTLYRHCAFVERPLESESPFFWWGRRQTDMEFLSVIAREYIACPATSIDSTAFFNEGGKFNYLCNTYSYSQLDSCLNVAGIHQHYRGRGPTSEPISPEMVETLNSTANRLQQTTLYALTEQEAAEITAGFYPPMPTYANQEEKVFEKLPPHRRTVNRIPNSVPNPIPIRKVPLQRMVPGGRPSILRSTLPPSRTLGLPLTAKRPVVPRESPPGNVVTEEKPRDLLDILQEPSQEKEIKLEEPDDFWDSAPSGPSTSAASAPSTSTQPSTYQTSLKGIPQKIATHNFVQKFAQKQNFIIKKKPLPPPISIRANMPRIQQEYKPELFNVGDRERILARERFQMAFQPQETVYSQTMNAASVADAYERKNMKSSLNPKCSRHRINVEMVKSRPCNRRCTVCGHLEVHEKLKNVTIDNEKLLIMLGCIYRQEYTLREAISFMNRESKTYICLMHFEETVDEIYTMLRLSCPDDINTCSDDLIQNALITVTALRPFIQIKQLRKILFDFTLKNNHTRATMTVSRLDECIRRRSPAAPKTDNLDDQEITPKVNRAPRKQVLEEDQHDKTVKLIEQEEFKLPTVQQSDNEECANPTVCCYCSKRGSRLKMHRVPRTEDRLQRWIEKLGSVFEKRLKAEEDNFVCKSHFPEDAFSSRGRLMKGMIPFSEAEKKEVTYKIQGDSFLKLNEQKS

***Caenorhabditis japonica* LIN-15b**

MSLASSTDTAGPGTSAPITRSQEQPVSIRPMSLRPAGIMRHRPRPVPQQEEVIQSAALTDSRTDALITNFIVGSGLPYEIVNDPIFTELLSYMNPRCVLPTPKLIEACAEKMGNPGKPIVNYQKTQGPLSVTLHVTDTNHKKYLAFSIHYFNETRHRRNVIYLRELLLSKLDENNLLTTLRRAVSAYNYANVKFTNLVVSNDELYALLHRSGVVKRTFVCFYKYVSQFVEKILAFDDFASGLEQLRTFITVVKKDPDAYSKFRRMQLTKNAELDLPALDNSVWESTMVFLTRCLILHETFTEFSERCNIEIYISHAVFNQLVILQRVLQKCVACSRELSTKTSSISQVIPALLGLRSFIASAWGQNSLWKDVREGFTEVFSPLTSGSQAQRYDIATLLDPRYAYNESIYAQQVWTSIEKKLTRDFTVFDRVPNSERNFNVDLSSVGKDERIRILTTEFKLYRQLIIGVRPDDNDDPFSWWSRRSSDLEYLSVFAREFLACPAVSIDADYYFANGGKFSHLCYTYNHQLVENCLNAAGIHQEFRGRGASVDFIQPRQIDQLNTIANRRQKELNIKRFDGVDRSSSKDEYDRLMQSEYPPPPTVSEILKRPKIMDRDLSPSLVVKQELSPDNEVKPMTESNPVFKLTPSELGRGASITERYLAQNRPIKKMFRVVPPENPRQKPQIVQITNPSQLKPTPAEPTKEEKPKDLLNDLELKDIKEEPLDEDDVQAPVSAPVPQPSVQTPKPYSIKVRTPTNTSQISDSSAYLRGGVFTSTPQTRYSNNAAPKPSIASGVTFPKDRPLPQTVAPANFVQKYAQTQKFVQKFVVRKPQIQTLPGSVHELKHNGQNWKKNIFHQSEEKPETLAFYASKPGLPARNSLLRNTFDCFDDFDDSDEEVSHSRDSTFNEVLNRMHSERLENLGDFRETHNIG

***Caenorhabditis remanei* LIN-15b**

MQTVQQARLTTKPPSLPPPTAIPQVCRPPPAKAKGILRHRTVAPVSEPKVDLDVDPKTTEYLSKFFASTGVPFDTIHSSSFRELVRHLNPNCALPEEDLMFKYVEKQNSLTKPLVNFQKTVGPLGVTIDVAGNADEKYLVFSIHYFDDLYERKNIVYLRKLLLSEVDSEGLLISIRRAVNNQTYSNVKFSSVVCPNYQIYNLISNSGVIKRYHICFYNYISMFIADLMEINEFWDGLTSLRKFVRFVKSDPELYGRFRRMQLSKKADLDLPIIDEGPWENTFVFLTRCLVLHDTLTEYFERFQQTSYINNSAFNHLIYLQRLMQQCLKYCRELSSSNNTISQIIPAVEGLRQYIQTHDMGYRFQKTIENSLNNCLGYLNQIHVRSRYEMATLMDPRYAYRDIFPPLKWKQIEIRVQEEFVNMDASAEKSFYQDISQMTSIERRNIIMNEFVHYRQVSFVERPEEWDSPFYWWGSRQLHMEHLAVLAREIVATPATSIDASHFFSSGGKFQHLCKKYSSGRLEDCLSVAGIHQEFRGRGATVETMTESMLESLNSTAKRLRCTHLSDISGLPYPPLPTMAMNGYEMEEKPQHLLGAPLQMGMIPQHRQVKPITGRPIHSSGIDLNNVPKAIRIIQAPLQGKVKAPVPPGTVIYPREEQKPVELLEKEVKEEPVIEKVVKEEPLEEVSPPENIMKFHQNPSPPPPMTAPMSQQQTGNVIQGVQRTIINRAPPPQYIASHDFVEKFAEQEKFVIKNAQKYPPPVGIRSNIPTPSLNPPEEVKFEDEVFTGRDGDDYNEIYLQAVEIARATKAELRRQKRCNRHCAVCGHLQCQEDLKNVTIDSEKLLIMLGCLYRQEYTLEKAQEFMAKETKTYVCRVHFAETLDEIYSMLRLSRPEDIFNCTLFQIQNVLSTITALRPHISLKQFMLILYNFADRYRHLVETKYDMIGVNNSRYTQSEEGNDVDDEEIIPKEYRQPRKQVLEADQHDGTVKVIEQENFKLPTAKPSEHGDWDNVCCLCSKSGARNGMLRVPRGEDRLARWIEKLGSEFEQRLKSDGENLICRQHFPEAAFSRGRLLKGMIPDAVSEKVEVTYRIQGNNFLKLNEQKSGTDKNARIDLENTEDTRIREMLAHDHDYTPEPSTSSARSTYKRRAESSSSSEDVDDYGEPATRMPRRQAKVASSYEGDYVYDKKYARIASRHFNKR

**ERI-5**

***Caenorhabditis brenneri* ERI-5**

MTETSPYTPDNSDFPDPNSIRRIALKESAIVELLRVESPSSLFVRPIDHIRNQLVYKEPYSLTPITSIDVGVYALAPIEERVFGRCIIVRNIELLEACRVFFIDEAVTANVSWKCLFRIEESQMFHPWQAMHITLGRMISLTNEWSLEQCRNFSEILSDFPKFQITPSQVDLEDDSDRPSILVNLYGLQDNQDVNQKVAIEDICSVSMQDVLVTIFPSCLTDDPKLADLDKEQDDLEVILLEEFRRDLPYDWMHETPPDWVDGDENWDIEKCKVNEWNSSFLKPYLLENGCFWGFIKPNATVSPWKMYITPIIVEEEKNMSNEEWITEQFSQKLEMQRQFDDFYSIPQNQRPLEIDEIKFALTSGRAYAIAAVQHRKKTGRQWLRVEILDVLPNNNLEVRFIDQGIHGFQVLKSIHSIHSSHTKHPPFVIEMGQFFNDSVSDTDMEWGNEHWRYIVPYDIPVVFGPKLDFVETGKLLFAEVRNIDEEENLLDDIPPQPVFTDQSHDSRSWNEFSNEKKGENWELREMDEEDIDSYISA

***Caenorhabditis briggsae* ERI-5**

MTSVPCPYSKIQPEFINTKSIQRMPMKDSALVELLRVESPSSLFVRPISHIRDQLVYTEPYFLVPHTNFEAGNYALAPIEERVFGRCLIVRNIPLLEACRVFFIDDAVTANVSWKCLFVLKDEERFHPWQAMHISLGRLGSLTNEWSFDQRRQFLDILSEFPKFEITPCQVDIEDDQEEIDRPSLLVNLYGIEEDKTMDEKVSIEEICGVLMPNVMVTCFPTLLTEDPRLAELDKEQDNLEVILLEEFRRQLPYDWRHEDKPEYKEGDEDWDIVTCKLVEWDKKWLKKYLLEDGCFWGFITTNVTVSPWDMHITPILKENEGSADEQWIFDQFDTIKRMQLVFDDFYVCPKNQRPLDEEEIRYALTQGRAYAIAAVEHRKKTGPQWLRVEILDILPNNNLDIRFVDQGFRGFENIKNIYRIHRLHTEMPPFQIPMGLYFDDVSISETEMGWSSHFWRDIVPFDVPIVVGPKLEFIETGKLQFAEIREIGEQENLLDEIPPMSLYSGDGFSHYDDEEENSDLGSSGSSEVNYEEDEMELKSDRRGIPEEEDEYDSEDPDYYDDYD

***Caenorhabditis japonica* ERI-5**

MAETCPYDANVQLKLKQQNWKSIQRMPLKDTAIVEMVRIESPSSIFVRPKNHIRDRLIYREPYKLSPLTHLEPGQFALAPLEERVFGRCVILRTDKTNDECRVFFIDDGITSSVNFECLFQIDESQFFYPWQVMQITLGRMESLSPDGSWTPSQCSDFVNILSRFDYFQINACHVDLNCAPDRPSILVNLYGIADIDNLSSNISIEELCQSEMADVLVSVFPTNLVDDALLDDKDSEDFCVEEFRKKIPHDWVHEITQTGDNNDNDEDWDIEKCCVPEWSMETLEEYCRYDDCFIALVEPRSSITSSEIHVLPIKNELSNGRYLVFWVTVALISTVSKTALSKGKVYAMASIPHRKQTESSWYRCEIIEVMTTHNVSVRFLDNGQKSFEKPKNLYRMHRNHTYQLPLAIEIFEIKGDGEDEKRIKIPYDVPLLVGPEMKFIAPGKLQVSQLRFFGEIGGGRGNKNLLEAI

***Caenorhabditis remanei* ERI-5**

MPNLPDPYEKCHPEFLNPHAIKRIALKDSAMVDVLRVESPSSLFVRPIDHIRDQLVYREPYPLTPVAKYDAGNYALAPIEDRVFGRCIIVRNIELLEACRVFFIDEAITANVSWKCLFEIDEEQRFHPWQVMHVTLGRMVSLTNEWTLEQRQQFLDVISKFPKFQITACQVDIEDPQKETERPSLLVNLYALDEEQSVDEKVSIEEICSVSMDDVMVSIFPINLTEDPKLAELDKEQDNLEIILLEEFRRSLPHDWIHEESQDYVEEDTDWDIKTCHIAEWNESHLQKYLKEDGCFWGYVTTNVNFSPWEMHVTPIINQGYGANNEEWIFDQIQALKEKQLEFDDFYSMVKNQRPLEEEEIRVAFNYGRPYAMASVQHRKRDGAQWLRCEILEVMSNDVVAIRYVDQGFRGFEKLRNLHRLHVQHTVQPPFMIEIGKFFKDNSLAETEKEWSDYFWRDIVPTDVPIVVGPKLQFLETGKLLYEEIRNIGETQNLLDQIPPLEENTENTDDLRTELDCEEENYESEESDDSLVYF

**ERI-6**

***Caenorhabditis brenneri* ERI-6**

MSEETSIYSGFILKVVKLDVPDLPSIIRVASFKPRSDFRRILPRKAEKSYKFDFGQIIHFSPRSGELEENSLVIIHEDIESLYEHRKEFHTMIIHILKDVSVSAEDLPKAGTYKHPDLGIMKDEESRIKTGMMSQIFIKVLLTKDSFEIEVQEAKPQKHETPLEKVIPELEKFEQIHEIKNCGNEINRKIENKLWRNIGLLKLTDFNKTNIQGYRPVF

***Caenorhabditis briggsae* ERI-6**

MSKQLVVNGRTILVEEQDVDEVCAMFKENIIIDSEEGWSNRFEVTQVAVFFCGFALHHSGLPWKRFDYLSDCWVKAERMHGDTYGRGNFSPSEYSSVISDVQTFVNEFSKIDRKKLWSEFTSLHVVNGCAQTFGFNVLKQWADDQETMGSLPKKTSKLEKDTKKQTAEIEKKVVEISNALKDLDVMKKVYTKFVDGKTVESRSSRDNLCVTDICLFRDKEACQLVNKTTVDCNGCAGRAHAVCCGIWTEDEYLITLDQSVVAQCWSCEGLGVSEILLFCEQQTTDLKNVHNQLTESLKFVTQYLYVIACIDFRDVIETADYRANFWKGNGSLRKESEHRWRRNGADISAIFGKETEVSEKNRSTDGEEMELILNLCWEHLQVFAGDMNMTPKCHILLDFMPYARRFGTLGRMSEQSIESFHALFNRLQDRFKTVRNDVTRYTHCFRVLLFFNHVFMNS

***Caenorhabditis japonica* ERI-6**

MDVIRYLRKRAVIKQNMTSFEGFKFFQSLNLSRGHFSLTKSLFREFGISDPTPSRSQIESIEGLYGDDDYFEVITVTSTDFNGNSREVTVSRLKNVQSYVQLRIQELAYRGKLLFDEESGPRIWLTIFGDKGGDEFKLAVSIANVETPNSAHHLVPLGIFNDDENAENLTKYLGPIIDELNAMSEVEIELANGSSKIPIVQFLGGDMKFQYEVLGHEGGGSLRSCSYCYKQGKSLIEEYERGVGCVKRSEASYEQDAHSESKKNRNNVKPNSTFLFKRVTLDRVAPASLHIMMGVVQKYGICYCLNSTTQTDNDSGLSHSLHRSKN

***Caenorhabditis remanei* ERI-6**

MTETSPKPLIRNDKVVERIRRELTTLNGRFKPLIEHNEILKKDIDMMKGEKETVAMRVVEMEERNEILESIIKREMDNNDSLRDLLEYEKGKSEKLSERVATAHHLKNIAIREAEEKKIENELIKVEIDKVKATLEKAQADSKELKNNEKILKEQKRSVILKMKKEERKTNRYKTRLSRVIPPYDGLASRKGQYNRIEKAFSYLQYLAGTNSKHFYKAIVLKMERKGVLKLKLTDKEGFRLYHCTRMTRHRLKLMKREFKANGVIDPLPNIQAIVSIEGDVGSKEIFTVKQEKSVKEDREVVVVHLTDVVKSLTHRVQELIDNQKLTCDFSRALWVTILGDKGDAEFKLCLSIANVENSNSCYHLLPVGIFDDDESSVAIQHHMGVVVEQLNRLKSLKINIGGTQYDLPVEFFVGGDMKMQYDVLGHQGGASTYSCMFCKKKAKQLIKNYKRGEDVELRTEETYVEDSLKGTPKKPVHSIKAGSEPLFKSVKISNFIPGSLHIISGLAQRMGFDYLLTWSAQMDCKKKIHRKDIKKKRISSDKIQMLETELKTLDLHIQSMSRIVEVLHNIKNRRIDGSDELEANACQAEKCLFRDKAMELAPIYDSHTLHCSICDCRLHALCSGCWSIEHFFQSFDSLVPFECFACRGLSGDRIDARATAMLDELKMEKLNMEKELIDEKSAFDTNLSAIKGNEPTRKLLESIWKRHGAYMSTWQQNFVGNHIHKLLQEEAVDEYMSIFKDHEGMPSMSRFLKALGELQRICLPKMLSDEEIDRMENIIDTIWVNLQDFAAEDKVIPKLHVLLEHVMPFVRLHRTWAKTSEQPIESIHAYYNSTKVQFRTIRNKKLKATMCFKSLLYKNFISDHS

**ERI-7**

***Caenorhabditis brenneri* ERI-7**

MSEENTVYSGFILKAIKLDDPDLPIIIRVACFKKRSDFRRILPLKVVKDYDFSYGQIIHFSPRDGNLEENSFVTIHEDTKPFSEHQKDFRTMIVHVLKDVPVSAEDLPESGIYKHPDLGIMKDKESTIKPGMMDEIFINVLIIKDSFEVYVRGAEHEKTGTPPEESIAELKKFERIHEVIRCEHEYNKAAENATWRKIGFLQLIDYNKESFQGYKPIFKLLDQQNLEVFNKQKKNSEVAMIQVVEIDGKYQDASEVQLATLIENQENIGERCVMAYLKAAYRHKFEQIFEIGALFRFESALSEKTGYFWEKQKHKNNELFEHTLYRSTEKEIEEMKIMDLIDSRMKSEGEVLKDRGSQQNSLNETQYKALKMALNEQRKVVCIQGPPGTGKTFVLAHILATLIFGEKQAIVLTPTKEALKNIMIMTEKVIKEKNLECHDKTLMDQVKFEKAVNNSPAAEDAKEKIAQWDENFKNNMLKFADFYDMKTNLINRTFAEVGTEIMKQTRIVFATIQSSFVDTTMKYKIFNPCMCVIDEAAQVMETQTWPAVIRMKRIILAGDPKQLPALVLSAKAKAAKLEDSIMDRIIHNKEKFSWIMLDEQYRCHPNIIAWSNKSFYDGALKNKTSEDNTIRNNFKIEAPPQFRNLFDAAVLVDTSSETDPDRRETLHELFTVGPSPNEDGGSSRSYKNEGEARLVLLHYKHLRELGIEAKNIAIITPYRAQTELIKQGMANIIEDDGDLSCSETKIGTVDGVQGQEYDCVIFSMVRSNPRNAMGFVGDLRRLNVAMTRAKRHLMFIGNGCLLANHLSPQIQNLFHEFHKNDRVFHPQNVGYLPA

***Caenorhabditis briggsae* ERI-7**

MTANQNLYSGFFLKCLPDNENGEHLIRVACFKPGTTFRKVLELTTNVSCERKFGEIVHFWSLDGEMTDKSHIKFKDDQPPLFQHETAAVNEIVHLIQNHQVNIQDIRPLNVLWHSFLGEINCDRFPPSPARYMFLLIQVNVKKDTFEIELMGGREEDLKQKKSLDITLEQQIANYPEVLKIENDHTCQSENSAWKEIGFFELKKFSDKELTGGIRVIFRLCDSKGLISLKRRRQAYPAKIIKQRINGNEFEDDPNYSIESGSLFEREKKNKIIEEEVVCKLKSESRASFERNFKAGDVFRFESEQNPSNVEFWQKQLQKMMPLYNLTIDDAPESLQVQDILDNHQVEAEKLRKLTDKGFPPMNLNPSQLLAIRMAMNEDRPLVCIQGPPGTGKSHTLSYLLFRIMRSKKQAVVLTPTREALKNLKQMTEKLLKEREQEFQVHEHALMDIKTYHKLIDASDEAKSAIEQIRSIREDAQMGEIAHEDYSESSRRLEKAVCSEVGREILKNVRVVFSTIESSFVTEVMKVQSFQPAMCLVDEAAQVMECQTWPAVLKMKKLVLAGDPKQLPALVKTKLGRDLKLNQSVMERLMLKKENYSWVMLNTQYRSHEEITHWSNSCFYDCHLKSSTKDEKKLVDELNPKPSFTGLYEPMVHIDTSGVKTDPERALTYEQRVTLVTDGEKEYSYSNIGEATYAMQHYKNLLDMGVKPENIALISPYRGQIELLGRMIDEYCKTSNNMDCKNTKIGTVDSVQGQEYDVVIFTSVRNNPKKNFGFVSDVRRLNVVVTRAKRHFVLIGSGYMFKHNHISEYHPQLESTIPSSSSNRRVPGLDLEPRNNFGFNFENFMKNSEDTDMIAWCEEWIRNGKDPLRAQTDDWKLKKLRDELRSQLYRSLNDENS

***Caenorhabditis japonica* ERI-7**

MNLFMGFFLGIIEKKEDDDRAEQILKIACFKPTTEFRRVVDLLVDGEMSVNYGDLVMVDIKTESEPTDGVPAQLRSLAAELKYVHHENKVTDRLVYTVRDVQVGQGDLLEIGMYRNIDFGDMIDTNREIEPGSYATITFEIVQRGEDSFELVILQALTQTAQMAKLSKQLQQRKFDEMPEVMEAEIQHNIELETKKWESVGLLRIAAFNNSNLFSGLRPIMEIMDMSKVLTFQKLLNASRVALIKQKASAKEGEWEDDEVYDPQIGTAFETVNEVNRCVIQLKPHYRGKFETSFQIDDVFRVEADIDRDDVAFWQQKRHEENEVFKHTIEEVSRDKLLEVLEEHACDRDELAQMTDTTEFPRRLNPTQNAAIQMALNEKRKLVCIQGPPGTGKTYTLANLIYRILRQQRSDKIRKAIVLTPTREACRNIENATRALLASKPLRKNPHDHALMNPLLFNEILENCQESRDGLQLVNAWREEAASGRISWEEFKKKRAEHIRAIYQEKLKEVMGNVIIVFSTISSSFVDVPTRFPSFQNALCIIGT

***Caenorhabditis remanei* ERI-7**

MDFDRNYCSGFFLGLLPDKPNGNNLIKVACFATGTNFRRVLDLETESECKFAYGEIIHFITQTDRLGPKSVIMLQKTDLQMYHHDKSIIHEVLHTIKNLYVDAINLPASGNFNHPDFGEMIDTTSIMSVGSYVFITFMLVLHERDDSFDLQILGGQTMEECEEIFLRNEETKQFLNGIHDVIAIEHDKNKESETAAWEAIGLFVLMGYNDKEVTSGLRPVMKLMDKSKLPLFLKRKETAPIVMIKQKEMDEVWVDDEDENRGQPQYGSLFPRIRVDATKKKRPILGKPQKAKENVDDECIIGLKRECRHQFETIFKVGDVFRFESSPALNNYSFWVNKMHRKNEIYQYTVEEQKSEGLEIQKVIESHYSTPPEDFKLKEDTDFKPEDVIKQEFNLKKILLSVWWSVHGLLYGELLPEGKNITADYYSSQLQKVKSKLKTSPLHGHGVHYLHDNAKPHTAKTIKSLLATLHWTVLTHPPYSPDIAHSDYHLFSDMHRSFEGQDFKTKSEIEKWLKKYFDSKQPEFWRKGIESLPTKWQRVVDKGGHYDKCTPRGDLNQSQYAAVRMALNPNRFLVCIQGPPGTGKSHVLSIFLWKLLKEGKQAVVLTPTREALKNLKTMTLKAIRQKIYLHPHALMDISLIKEILNTSSAAIEAVKTINTIMPNLRIVFATMESTFTCEVMRSKQFNPEVCVIDEAAQVMETVTWPAVNQMKRIVMAGDPKQLPALVKTEEAKARHLERSVMERIIEKKEQYSWIMLEEQYRSHKDITGWSNGCFYDGKLMNSTDVNNTLHTSLDPKPPKTYTKLFNPLVFVDTCLETDYLKRVQYYEKTMASITETEHSHTYCNYGEAELVMKHYERLLAFSIPPKNIAIITPYKGQVSEACHILFLDWWTIKRINVAVSNVLDKLKNGATLGQHSEKDNEIAGPPLASCRR

**ERI-3**

***Caenorhabditis briggsae* ERI-3**

MTPNPQVLVNALPLRLKPRRSRSKTALELAEEAFEAACYSSSSGVVISHKNGTGEVLVAESFEDSGLHFIFSKSTNIQYPTNFDDIGVGSVVQVYWTRSFERVVKTAHIVIQIEKMEVYKCATLLRDRIFVTFNSHVVPGVALGISERNTTVAFHPNCAPKLSTETLEAHAEGRTEFEMKAKHRENTNRMVEVFLVLVPFRVEIAGNVDKIPFVVVRKIANTRGREGVAVITKIIKNHFMEANFLKSSERVYFNSQACHSNILEKVTIGSLINVCASPAFPTSHYRWYGYDVTLCKNYLANADTQQSFNMRRNKILQNYDENDPDGGHPMKNAKEAFANITFAALPEEEVPQNLMNPREANEIVDSYFVDRMEIDDKENRYMRRVEREESYLRQQRELDENEDMQTTLPVYSESLADEIHGVLEPFNLNKPRTVTQQPPPPNVPYFMLPPPATVPRTHRRGNELPPPLSVEEVRARFGSLMDADGFALNQKVQPEFVIPDTNWKPTERRWIGIHDDVQWVLMATFLPPPPENCTEKKLLGGWWYRRSVPREHPVGTVERMETRRNVIKDCTVSMLIE

***Caenorhabditis remanei* ERI-3**

MNPPEPAIINGQPLRIITRGSSSKNELEIKEEAFDAACYSSSSGIVISYKDGSGEVLTPESFDDPGLHFIFSRSTVFQYPSVYDKIGIGSVVQVFWTRSFERVIRGSHIVVQVEKMEVYKCATMKRERVFVTFNSQMTPGVATGITENYTTVAFHPNCSSKMAFEALKAHGEGRTEFVMKQTHRENTHRMLDVYLACVPFRVEISGNFDKIPYIVIRKKGPARGKEGVAVITTILKNHFMEAYFLQSSDRVYFDRKACHSNILEKVSIGSLIHIQATPAFPSSFYKWYGYDVTLCHNYLADIHTQRSFQMDTANKILNDTKDDEEENDQPMKSAKIAFQTKPVVVVRQQDEVKQKIGQNRSEPSKTFRDDVYHALGCHDDLKWVLMATFIEPTAENSARKEGLLGGKDARSIPLPYGSESITSPYTHLIYLIPILRKPLNLHKFNSKHAQYKMRHLLLDQCYSTLIPREAKIILDAYLVDQYPEYDDSGSENQYVMTTRLESDDMNTTRPVYSESVEDDVYHALTPFGLNRKPRRFRATPPPKKSKKGNELPPKLSPEEVRQRFDCLMDSDGYALNQKVKDAFVMPDTKWKPTERRWIGVTMI
