# Supplementary material for: RNAi Effector Diversity in Nematodes
Source: PLoS Negl Trop Dis. 2011 Jun 7;5(6):e1176. doi: 10.1371/journal.pntd.0001176 (PMC3110158; doi:10.1371/journal.pntd.0001176)
Supplement: Dataset S5 — Nematode chromatin modifiers, histone methylation factors, and other nuclear effectors; domains and sequence data. (*, putative stop codon) (DOC) [file pntd.0001176.s005.doc]

**Dataset S5.**

**Domain analysis**

EKL-1 (Enhancer of KSR-1 Lethality 1) is characterised by dual tudor domains. EKL-4 contains a DNA-binding domain and DMAP-1-like DNA methylation domain. EKL-6 contains a carboxy-terminal armadillo-like helicase domain. MES-2 (Maternal Effect Sterile 2) is characterised by a carboxy-terminal lysine methyltransferase SET domain. MES-6 contains WD repeat (adaptor) domains and functions within a polycomb-like chromatin repressive complex in combination with MES-2 and MES-3. MUT-2 (MUTator 2) contains amino-terminal PAP-like domains and a carboxy-terminal PAP-like associated domain, similarly CID-1 encodes a carboxy-terminal PAP-like domain. MUT-7 interacts with RDE-2 and contains a putative 3’-5’ exonuclease domain and a carboxy-terminal uncharacterised domain. MUT-16 is typified by a glutamine/asparagine (Q/W)-rich domain of unknown function. GFL-1 (human GAS41-like) contains a YEATS domain which is thought to modulate aspects of transcription. RHA-1 (RNA HelicAse 1) contains dual amino-terminal dsRNA-binding domains, a DEXD-box helicase domain, an HA2-like domain and an uncharacterised carboxy-terminal domain. ZFP-1 (Zinc Finger Protein 1) is characterised by a single amino-terminal zinc finger domain. In all cases, the aforementioned domain topology was required of putative orthologs, or minimally, one matched domain in the case of short sequences. MES-3, RDE-2 and EKL-5 contain no identifiable domain characteristics, and as before, orthologous proteins were designated on the strength of overall sequence similarity.

**Shared protein domains**

None of the above proteins shared known domains with each other. RHA-1 shares a DEXD-box helicase domain with other RNAi pathway proteins, however, no putative RHA-1 protein was identified on the bases of DEXD-box helicase domain similarity alone.

**MUT-7**

***Ancylostoma caninum* MUT-7**

LFXRHCYAAHPKXLGFDLTNDLRALFGAASTANIQAVADNLCNVVCLKRLVENLLSVDRHFLGNQSHIEGGSDCDPDEDETVTHFKLSDLSKRLLDITLDKSEQRCNWSIRPLRQKQKSYAAMDAYIVIELYGELNKRAVARGLDFEKFVQQSVVDGRKRDKVRLRKERVKMDDMTWAEICDKLHDVQSGTRPATQLQCIVDSMLLGLGKHLRRCGVNVLIPGDRSELKMKARGNSRIIVTSGKAYDELRRQFADRVLAIPNASALGPIEQLKCVFAKNKVTFAGLDVFSRCMECNGTCFVQAPGPVIQALFENNVTCKNGFHDEPFNVVGWTERLSSLDCRDFSGIGCRLLPTNEGHMVVQCHGGIVYITANIVKHDHLEEGVDTMVRKVPEQVASRPGRVFYICGQCGKIYWEEQ

***Ascaris suum* MUT-7**

VNADDYSGYGCSLTINEDDKSWATARCYNGILDVRNCLVFASNRDAPSVVQIEKVPLPVLEKEGRFFYVCGQCGKVYWDGCHIVNYNTFAEPLFANETTEETVVSEDVNGKGKGGEVRWKRGWRVARCSGGQYFSKEHDKRGGVVGGKRDRLSVFLIGLDRSLCTTDLSNL

***Brugia malayi* MUT-7**

MVSERASITNVELFPGHPIKVIANLKDLEELYPVIEEADLIGIDTEWKPLFICTNERLKTFLEIARKVSVGLSLLQVALFQICVQHCSYLVDVITLENVLTEEQWTRFFKALFCDSTAIKLGFDFLNDLKVLRASYPYLQPLEEMKNVVCILKLVKSLLASNPAFLDFSDSINLPLSSETENLLDIVSDETVHFRLTDLCRKVLGQALDKTEQIGNWAMRPLRREQMKYAAMDGYCLLNLYNKLKIRAERDYNMDWTKHWKECDIAQIKSKENKIEKRTKKKGTKFDEKEFEQMIERVNSDLSNAQVKRKPKDLKVIVDSMILGLGKHLRR

***Caenorhabditis brenneri* MUT-7**

MEEDPPQRVKLTKAQKKEKYRKDIPEPIKSRRECLKSVMNGREEDREQNVRRLNFNYFDEDYRSQPPVNMYARAVDTLKAMPDKGKTGGQCLAEWYLKDFDSWLKEKAGREEQLRNEFLTPNVQGEALKACTVNQKALLCRVFDISQERLIEDMTELLSAAVQKQDYSNAAEYALQYNLAHKHSFEELALPLLLSGKEQLAYKLLEGQVEMQKELVKFLDSMSGASITTVEKILEPYKMKNIMTVQTERFSGKTLDKLIQTVIVRNVNDYNFDPNLAKYAPKHSVHGISKNLKYNINERYNNGRSDDVYFQHMVDGFKDCPSVREETLYWLWDSNDYQKQVDAISLAMHFGFDHSSSTHLPGKMKDFFRAPDSRMKEAEELLKTRKTMQTPGSNEVLYVNEEEKQYPIIIVKSESDLNNLCSQLNALSKSPDQAYVGFDSEWKPTNVTSNKQLFFADKVWLVDVVELGNANVSDDWWQKFAVKLFIDNKFRIIGFDMRNDLDAMLTIPALKNFLKIEKINNCFDLKRLAENICDVDMEILELPKKTFKLADLTLHFLNVTLDKTEQCSNWQCRPLRKNQIIYAALDAVVVVDTFRKIMEITLERDSSIDMANIVNNSNVLAPKKEKSSKTVRKLKTIPWKEIYETLRHHRDTRKPLQKPHEIQVIVDTMLLGFGKHLRRCGIDVYLPRDVSDFRAKLKLISRLGGDFKRHIITVPSKSYDALKQDYEKFVIALPDLNSKPPMDQLTAFFDIFNIDIRPEDEYLRCIECNSRLQIKFPGPVLHFLHQYSVIFLQNVYRPDMSEFKRQTAIHNNLPEGVEVRIHKVPDDEFQRPNICFYVCGDCGAVANDGHLPPNSASNESVL

***Caenorhabditis briggsae* MUT-7**

MDEPRKKLTKAEKKAKYRTDYPEPIKTRREDLKAIMNGRPEDREMKARSRMRQFFEEDYNSNVNIYGMAVDMMMAMPDRTKTGGENLAHWYLEDFGKWLTETGREEYLRGKYLTSSVQLEALKSCLAEQKSLVSQIFDISPEKLLEDVTQLLRDSIVKREYSKAAKLAVQHNLSNTLDFKELALPLILSGKNREAYQLMASAVEMQKDYVQFLDEMIGLSQTGVEEILEPYKSSRVMTIGLENFCGKTVEKQIQQVLSKVSQEFNFDKDLPKYAPKHSVRASLKALQYSIQQRYNGIDDNGDDNYFQTMVDTMQQDPNVQEKILFYLWDSNTHEKQVDAICIAIHLKINYPSSQHIPGKMKDFFREPDSRLKKLNVFCRRDCECKFPLKGGLRPKIFISTVCAPEEGEQMYVFEDDKCPIYMIKTESEMQSICEEIESLSREPEKSVYVGFDSEWKPSNLITANSSKIAIIQLFFKDKVLLVDCVELEKEKVPDLLWERFAKGLFETPKLKLIGFDMRNDLEAIIELPALKGRLNLEQIKNAYDLKRLAENICDIDMDILELPKKTFKLADLTQYLLGQVLDKTEQCSNWQCRPLRKKQILYAALDAVVVVNTFKKILEKTQERNEDVDVPSVVKNSNVLAPKKERDQKVMRKLKTIPWTEIYETLRNHRDTSMPLQRPHEIQVIVDTMLLGFGKHLRRVGIDVYLPRDVSDFKAKLRVINRIGGEYQRHIITVPSKSYNALRVDYESNLMAIPELNHKAPLDQLIDFFDRFNVDLRPEDEFLRCIECNSRLQIKFPGPVLHFLHQYAVIHVQNVYRADMSEFPLEEWWNRMLRINPDDYDGIKVEMSRPNLKSKWMVATVPTGCLHITRQTAIHNNLPDGVEVRIHKVPDDEFQRPNLCFYVCGDCGTVGYDGRAPTESNGYESMH

***Caenorhabditis japonica* MUT-7**

MAELFLPFSLRLLFHALFYAAVVVENHECCIPLEGKQLYVFEEERKYPIYMVKTEESLSQLSSQLRYLEQDPDPVYLGFDSEWKPGHMTEMNSMKIAIMQLFFQNTVWLVDCVELEKLSVPVKDETWQKFARRLLGSTKIKVIGFDMRNDLDAFLTIPALKYTLKIDDIKNTWCAKKLTENVCDIDMEILGLTKKTFKLADLTHTLLGRELDKTEQCSNWKFRPLRQKQIVYAAMDAVVVVETFQKVLEKAEEANTFLDVHKLMSESNVMAPKKEKNKRDCRKLNTIPWAELYQELYDKRDVTKPLQQPVDVQVIVDTMLLGFGKNLRRLGVDVYIPRDVSDFREKLKLMARLGGDLQRHIITVPSKSFEALKVEYQRYMTAIEDLNTMSAEKQLISFLSRFNIELRPEVHFHRCIECNSMRLIKFPSPILHFLHQYCIVFVQNVFAADRDQFPLEYWWGEMLKIDPRQYDGIEVKMTRPHPTSNWIVATVPTGCLHITRNTAIHNNLPEGVEVRIHKVPDDEFKRPNLCFFVCGDCGTVHYDGRTQYASNGPITE

***Caenorhabditis remanei* MUT-7**

MSYDPPIGLLTGREKKLAKKAKYGQGYPEPIKSRREELKKLMMNEPIEIREIKVRAKNMEFFDEDYNKRENMYSMVIEMMKAMPDKTKSNGESLAKWYLEDFDLWLQKSGREKMLRDEFLKDPIIRTNALKACHVEQKTLLSRIFDISQETLMKDVTELLQTNIAKGEFIKAARLAVKYQLSDTLDFGTLARPLITSGQNKEAYELMQGCKRMQIDFVKFLDEFVGMSRTAIEQDLLQYEDKHGTVSSSKFGSVDHDKLISSVLSKVSLEYNFERDLAKYAPNHAQNASFKNLKHKISQRYPTDGAKQEMSDENYFQDMVATLQNHPDVQEQILFYLWSSNVEMKQIDAISIAIHLEINDKRSKQIPGKMRDFFGSEAMTEEQQKMLKEARILLEKRTMVRTPQENEQLYVYEDNRCPIYMITTESEMKNLCTEIQLLSEDPKPVYVGFDSEWKPSNLTSINSSKIAIIQLYFKDKVYLVDCVQLEEKRLPDERWQEFARQLFGSKNLKIIGFDMRNDLDAIIALPALRETLAIDSIQNCFDLKRLAENICEIDMEILDLKRKTFKLADLTQSLLGQTLDKTEQCSNWQSRPLRKNQLLYAALDAVVVVLTFEKILEITLEKNSEIDIIEIRKHSNVLAPKKEKCQKAHRKLKNIPWLEICEVLSRHCDKSRPFKRPHEMKVIVDTMLLGFGKHLRFVFLNIQDEFISRRIGVDVYLPRDVADFKDKLKLINRLGGDYRRLIITVPSKSFTALREEYARDLFAMPELNNKPPMDQLIDFCDKFNVEVRPEDEYLCCIECNSRLQIKFPGPVLHFLHQYNVIHVQNVYRADMSQFPLEDWWNRMLQLNPDNYDGIVVKMSRPTPKSKWIVATVPTGCLHITRQTVIHNNLPDGVEVKIQKVPDDEFQRPNLCFYVCGDCGTVAYDGRASHQNSKTSQSNDKHLI

***Haemonchus contortus* MUT-7**

QCSNWTIRPLRASQKRYAAMDAYIVVEIFSKLKTSAEEKGPSSRVLQLYIPVHANNFKNMIDRHMFKEAMDVVEEFGLQSDYGLHAFVIPCLLQDKLSLVIKYIENDRKMQVIIILELHCISRTQYSQSLFKESLVAYLDSFVGMSEYDVIDRLREYKDSQIMTMQYERFTGSGSRPATELQCMVDSMLLGLGKNLRVGINIVLDLDDDSDSVPLVHFKLSDLSEKLLGTKLDKSEQTSTFLQLRPLFPSRVLCIPNAGNLNPIEQLKYVFNRHKVSLSKQDVFSRCDFQAMIQKSCVSAKKKERTKSKKERMKIDDMPWSEICEVTFFDDFSIALLQIATKDAVYLVDFISLKMRNNSKENELESFLRGLLCSPSIKIECNASCFVKVPGPVIQALFENVVLVPEQVVSRPGNVFFLCGSCGKVYWDFGDRPKAIVGFSPINKFDSMYSTLREGKLKSALIVTVTTWKQLSALTKKLDGETHIGIDSEWKP

***Meloidogyne hapla* MUT-7**

MFSDENYAQSSINGNIKESERKNSTGLKPLSFRECLLKVNEIYEKSNCALDDKDKFSVIQQTIRRYFDDEDPEDVFEGFVNLILHLDKITTILAKQYDLSLVKTALNAYNDFLIRKPELTQKPQTIEDTELLTNALEIVSSLKFKHSNLILKTFHLNDVSMTPAASNMITELLIIEDYQKAIQWMALLNLRDHYPFEQLVCRLLCLENPESLELFVGEAEYRRKKLLKKLDELVGLHLRRRNGSDEFENRLIADHKRLAAVKYAKIFKLPDSDIANVMIQRAYDALIYNHYNWKDGKHTWTHFEELALPPISQNEFIREKYFNFLLDKGLGDKALYFAQLLKLPESQYPRRLAYYMENMQPNEIDSIRDNITKNLELRKRNQEIEQDFDCSLDDGTPIILVDSLESLDNVIEILRSGGERLLGIDAEWRPHYLAAQEKISLIQIATSKTVFIIDVLRLEVELELTEAEWINFFDALFCTPNVRKIGKSLKNLGLSDVANYFLGIQVEKSERTGNWSQRPLRLEQKKYAALDAYCLVELYKKIAPLILTLREDQQNSLQACALIQFTNEEKKQENYILDDGQVVKIKEGENDFFKNIQMASAEINEEAANSSNGPIKSINDVRFVVDSMLFGLGKVLRKCGFDARFIGDRKKIVEFCQMENNEDFIVLSTGKGHKQLETLLNPEKVLFVPASTHKGATKPSQLVNHIMHELRILIRPEDLWSRCVECNKRAFVPVPVQIIQLLFYMNAVRLGATWLEISEEDLQDCFQKLRNESKQQIRITPEMHNGKQPRQDRRLLDDKSYYIYEDDNFIICETRKCAIDIINKLILADESGGPVSIHVGLKYKKSTFEAENLEFFACAECGKVQFDNPGSED

***Oesophagostomum dentatum* MUT-7**

IVIVDDRNKLNDLLSKLKDEDFIGIDSEWKAQYLGPTESVALLQIAIVDAVYLVDFCAMEKKLSEDDWSNLLRTLLCGKSRKLGFDLANDLRALFNGAPPAVRAATEELCNVVCLKRLVENFLEIDSSILENPNFSSASSENDADEAAPVLHFKLSDLSERLLNVKLDKSEQCSNWSIRPLRAKQKRYAAMDAYIVVELYGKLREMAESREIDLRKNWLNYQXXQQRRKRRSKQRRTASKXMFXRGMKFVRNXATFSRGTRPATELLCIVDTMLLGLGKHLRRCGVNVLIPADRSELKSIAMGNERIIVTSGKAYDELRRLFADRVLTIPNASNLSAIEQLKYVFSRCKVTFEGLDVFSRCMECNGTCFVKVPGPVIQALFENNVICRNEFHDEPFDSEGWTQRLBBBBBBDYSGIGCIVLPPEDPEDDYMAVQCHGGIVHITANIVQHDLLCEGVDIQVKKVPEQVSSRPGYVFYICGHCGKIYWEER

***Pristionchus pacificus* MUT-7**

MDTTSAASDTGNPIVNDETPMTSNALEEEKKERTLTKAEKKEKYGKGSFPEPLATWRDLIKDLLAPVDKTARSAEDEKETIEIAESTLSQIFGLAENPYKAMFDIYVVFSEKEKYKKVHRSLFKTFEEWIQKCATPEKMEKWLTSEFKVETMDKIVNKGMGQLENLLRIFQLKGSELEDRITIKIKNKTDEVEKFIKGNYRITRDLIRYLDSMVEETDQYKFVTSEYGMEDAVAPRFEEQKIKGKLRYLLFDREATDGGFTTTIFDHIKTHIKKSSPYRKDVIIALFDRIDKRDKTEDKSTYYEEAKLWMLYYDLNPADFYTTIRSYFSSQPNWREDAARMLVEYDTGVETCSENELEDGTPIEWIDTWEGMVEMLNEIEELGEESMIGIDSEFRSTTNYKQEIALLQVSSQEKVYLVDFELLDRKLSKSQWEQFTLRLFGGEHLKIGFDMLSDLKAYSATLKLDLEDLRCTMTRVVCLKRLSNDLLDLDSSIIDLTQSTSYRTRINEGKSTQEVESANRQRAIKLSDLVEVVLNSKLDKSLQKSNWSNRPLRSDQIIYAATDANILIKIFIRFKEMATEKGYNYEEMVESIENDLAERKETPTEKKKKAKMTEEEYAQLVESINAAAISANGHEDKPNRLGVDVIFADSKEHLLRLGRENKSRIILSFGKNIPEYKAVFGDRVFVVGMGMSALDQVKKVLTEFKIKLSPSNVFTRCMLCNGDRFVMAPAVVLQTLHDCAKRVGESFDDELFQPGPFKEKIENANPDDYGGFECKLEEYDPKDSRFIVKCTNGVVDIYNNLVMADSVDSPVEVQTLKVQPEVVESGRPFYYICGSCGKIYWDGTHGQRYKEFADGVVGTDSSKSNESVRNE

***Trichinella spiralis* MUT-7**

MTSAQETVDELQRLKISEIPKFLLDTFQSWTDPLVQLIDLLENVSPYFTPTRSKRLHLIIIEKFQHWLEENCKVTSVSNDLLIRAFDIATACDMEQFYMMARIFQMESNNSIFVDSVRKLAHSGRYVDAVVCINTLRLHSIFELDEILVPCMFHINGNTTDVLQYLRSAPNLIKKFFQTFDRLLNSYFSMKQYAEQVHIIGSNFTRKDRKSISKSVIFLAKHLDVDISKYKSLRFFAYCGALKFLCASLYTNGDMNYDGWKELVEVVLQNYPESINFLIELLCDTYCDQAEAAKWAEQYEVQLRNLFKCRNRRFLTSQRTFVNKAAVISPQTKPKIGEFLQLPSFIEICFVDNYDAALTVLSEISKEDTIGIDSEWRPTMFINDVVSYDMRMSDIFQFNHNVKHLFKGSRCYSNSYSSTGHSLKTDLYYIFGNKDFSVTANVKSVEDISKQIYGMCGSHFNDAYDVFAKKYNLLSIKQLSDNFDIDTLVSCSEGDGNIKLPKLSFTRSLNDHCFVYLGYWLSKSERMSNWQRRPLREMQMQYAALDAYSVISICDRLEDFMNIAGEFMSNVENEHK

**CID-1**

***Ancylostoma caninum* CID-1**

MRSSEHWSDACPMMTIPKIETISHTKQEYEWLELDHVILGTYEKNRMKEHRLEKLGEMVARIRSFLEKDLKRTVRLDIFGSLVNGLGAGGSDVDICFRFDSDEQPVNVDGVEIVREISTSLQKMKGLDKVYAITGAKVPIVKFLWPKFGFEGDISYYNVLALYNTQLLKTYCAWDQRVAPVGVWVKRWAKSCDIGDASRGSLSSYAIIILLIHYLQNCEPPVLPRLQEDFRNGDVKPIMVENHDVYYHNTVFEKWSENRSSVSQLFVGFLDYYARFDFETQVVQIRRKKPLLKMEKDWSRSVCIEDPFDLNHNLGSGVTKKMYVFIVRNIHNSRKKFMLSDIRREFMETNEISHDKKIPPNLIDDYAIVLLKKCEMGLAPSDRQCRICHRVGHFAESCPKNQHGLRTPRREPWPKKALAGTNGDTPRAGQSSGRTYSTRSFYRKTERTTRQNGRSTQ

***Ascaris suum* CID-1**

YGNVFDYILVAGRCETHDAEDGKECLSVVPDTGVFEMDSTDVSRCYYSRRVMFELSRKADSNFISQKVAHTLEGLGISHGDPNANNKRIETAVAADTQITDAKDRGRFLSVFVGVIEGKKNSARRRESRRAKKSPTSKHADESIEFTLELPELNMEMLEEELTSLHKEDFVFEWDVKALTNDVRKPSMVTFASVRMKLLQIHSGRLPSIKEDLFARWNGTLEKVDKRIADVEKRGFILCDEGSKPQMRCVCCGRSGHLIDNCPDLEVPPAIPLKPLTNMQKEFLGDIIISVYENLRMTVQYGNAMRELCRNVERRLRADYRSDCRLALFGSAGNGFGLIGSDADICLRFASDTLDEKLIYKGKRFQGVDTNEVIMRVAAVLSSMPGIANVIPIPNAKVPIVKFHCQHRYNRLEADVSLYNVLALENTRLLHAYSELDERAKALGVVVKEWAKCCEIGDASRGSLSSYSFIVMLIHFLQRTTPPVLPFLQEAGDRSVELGEGVIFFKRERTS

***Brugia malayi* CID-1**

MGSAIAQGSSNSKRTAADQKEKQKTTINSDSLIVSQKSRMASQKISKNISNRKRTRKKRTSVISATSSALDASLSRFENMSIQRENVFGHTEMQSATQDNGHLNWKVNDMSSEMNSEVRNFWEHPDLGSSHEKRSKRNKLKNSSFRLVTLAASTSVNEHANAVARCNEPDVISLSGKEGSENLEDDAEFTLVEKAGNSLILRNKEQMKLDVVCKEKKQFTTKVPLRSGPLLIRKDSFDGFNLIPGTHKFIQNGEIEVKIEKPKGKDFIQLILFECCGPLEDPKQVTRGGYKICASAVLHENTSVEVIRAALIDLAEQLERISRDSFYGGEKLSYSVSKYSLSYLEKQYIYPLKKKSSSFPRALYYCQLCKYHICSISQAVTHFLAQEHIDNKEKAESLKKLACLPGPVREQMFKVDQIIRELYIFTGLTDVRCEVGSQIANLLSDFLQNKAHRNYSVMVYGSYLTRLATVHSNLNLSLIFPLEYSLGHALSEVMEVLNDTALEKSFTVHSITPDFQRPDPSIHCTVNSVAVVITANCVRQQRATQLVNLYCTICSQFRILATIFRSWANLCSLTNVQLGGIPKFAFDIILIYYLQRKGLLPFVFESSKYLVLNISQVMGSLQILSEEEMLLLDSESLDFDHQVDKINTGFGTGSEKWNFGEIWIDLFRYYAIEHPIEELIQIRLRKRYLSEDCTRWSKKRLAVEDPFAPYHIMQPQQRINGYFSSCFLFTYFHFALPRTIVCSLIPYSVITPGTVAVQTKKRRKHRSRRTTLLNGSCSDEAAANNNQQINKGDLDDVDNLYQDATLEAIRLETDVSPQGISEVTETRLEDVNQIWTNKDVNDENSLLYGEHKDLEMSFSDPVTSCLDLHTSHSDLQRDDAGETCCDRNVTSTFQQHGIHFKELPSRMKAFKLSAAAVQNCGNLYIKGEYEGRELAGNRLANASILNIKFHDYDEMAIRKLWLTLNNNDYDYPWALHFFTGGLEPVARCTCCNADGHLRENCPELVIPEPKNFPPMTNAQKKIINLIIFDIFNTMRIRPYYVHHMSALCRELEGFLRRFYRTDCHLSLFGSAGNGFGLLGSDADICLQFGPGVRPEDIDSVEVINKIAEVIRKMPNVVYVCEIPHAKVPIVKFRCRNHYNLEADVSLYNVLALENTRLLRTYSKLDRRIHQLGIMTKIWAKNCEIGNASKGSLSSYSYIIMLIHYLQRTDPPVAPFLQEVAPPGRCREPIIIDNCDVYFCNFEDLEWTVHNRLTVGELWIGFLDYFATKFDFTREVVQIRQTPPLMKLDKGWQSRPIAIEDPFDLSHNLSSGVHSKTMAYIQKSFIQSREKFNTLSVVPNSKLNHNSLVLYASLLLSSCRVGNGPPLDRNCCHRCRQVGHFVMDCPLGIKDKKRNK

***Caenorhabditis brennneri* CID-1**

MPDTSTSSNGSGGTNKGAKQHQEKQDSRRRRGRGNNNKKENAQRSNGGTPKNQRSKSPQSSTASSSTVTEDRSKAATSSTTTKNTSSSSFPKVNLLKRGSDTSLLPPRSTPDQSFQVNCIMPSLMDVEVKPTTTMKSQNYKEVFTPEKKSTINLRQSEANIKQRQEYYFSNNPGTAVSADKFQEAFQYGMNTFPLEKNVNIQLDRKYILLITPTTSRCNGLFIKVKSQHGDDQLSTGALLRSDANDEAVRAACKETIDRYVEAKCHNERFPFSKLSEPYLTACYIRRLEQRQDAFPEAIYYCEKCDYHINTVGHAKAHLESSTHFDDIKRQEQRETLLKKIPKPSQAQLAAINKVLNEALEDYQKIRRKGQEHADNIITYLNTSVFPGLGCENVKLQPFGSVTYDVVLPDSDFNIAYTMDLPEGTPIFSLLEKVRRKIADDGHPADHSMEMGTPSTILFTYQDIRVRLCWMSCFNNRSQLCLSDLMKTYVGLRKEVVEFLQIIRLWASCAEVDSKNRPRIGLPRYGFDIMAIHFLQEKKYLPILHEMFDEDSAESSDLNSDDLSLNRDDCSERSKIPPEEAGQRRIRLGLKYEKDLEKIKKKFDLEKKWNNAKLFIEFLRYYVQQHRDGVIQMTQSPPMSRDINRWNKKVLHVVDPFRGDNVLSIPKVSTWQPYYFNCLLTTFLSFAIPRTKNGPVVEIGLIHNKSNNSKKKMRDTPKRDPTITTTSSTPKPISKITEEEFQKAADELAAEEEEARFMENLKQRLVIDGIKYTARKPEDCNIDDHSQGIYSNRSLRRFRRVLDCQLNERIAIPLEEHDLLTKKGKGWMKKWKKRRDSVEEVTEKLDETLKISDSHPIIHARVMCVMASQVLPTDSTSSDTTIKAEPSPASSVKDCGSEATESLSEVVPKTTPDVPITPEQGETQGAPVESDSENPEDQNPKDPGNPETEELEIEADHRTDSEAPPTPAPKTPKTTFAKPLLPQGSVEKQTCSEEFFIKENLAANEIQQKSRKISPSEYHYEFNNDAFCGGYEMEMKCTHCDGSHCVENCPMMEIPPIKKYEARTAEELKDIDNIIDKYYEENILTQHRLDLMESRKKELEEYLKKNYQKDIHLTIFGSVMTGLSVNCSDIDICLRFGDGDVPPKDRTPKEVILKVEEVLRKCGMVKRVQAIVTAKVPIVKFQLRLKTGEMVDADISYYNILAIYNTALLREYTLWTPDSRFAKLALFIKKWAKSCDIGDASRGSLSSYAHIILLISYLQNCDPPVLPRLQEDFRSDNDEKRLVDNWNTSYAQVEDELVQNWPKNKETCAQLLIGYFDYYSRYDFRNFVVQCRREMILSKMEKDWPRPICVEDPFDLNHNLSSGVTKKMFVFIMKVFISSRAAFMSEKPAAARDINFTTSYQHQLLRKCNQGSAPSDRQCHNCHRIGHFVESCPQRAQLKDSRRRYGSSNSTTSSYRSDAGGNNKKGPQDEGSLDVLKFHKRTYYNRSYK

***Caenorhabditis briggsae* CID-1**

MPDKNRNSSGGGENSSKSSGKKQRPRNRNKQSNNGVKSSSDNTDGNSTTSSSRNPSKSSPDISNARKVEILKRDPKAPPLISLMDIQMSKAPFQKNQNPASNGTTIETIKAQTRAYYFSENPGSAVSADTVTHQYTMQNFPLERNVNTMIDGKFSLMITPTTSRCNGLYIRVKSQNSEDAQYEKCGQSATGALVKLDSSEKTIRKACRETMENFYEVSTACRSKRHPFSKSSEAYLTSCFIKRLEERLETFPDAIYYCEKCDYHISTISHAKAHLESSTHFDDIKRQDQREQLLKHILDPSANHLKSINTVLEAVLNEYKEVQQISQDQTAHILYYLNTSVFPTLGCNNVKLRPFGSATYDVVLPDSDFNVAYTMDQHDGVSIFSVLEQIRKKIADDGYPADHSMEMGTPSTIIFTFEGVRVRLCWMSCFVFRSQLHFSDLMKTYVDLRKEVVQFLQLIRIWATRAGVDSKNKQRIGLPRYGFDIMAIHFLQRQQYLPILHELYDGEIEVEDPQTTKDEASSEVSKIPPEEAGQRRIRLMSRYEKDIKNIREKFDLTKKWNSADLFIKFFRYYVKQNREIVIQITQSEAMSRDANRWNKKILHVVDPFRGDNVLSIPKVSTWQPFYFNCLLTTFLAFAIPRTENGPLVEVGLLHNKTTTSKKKVKEPQTPKRNHVEVPPQTPDVFKTPKVIAQITEEEYQAAADELAEEQDEEEIMKQKLSRWKSMESSPPARPVTSMHAMENHVFMANDIRRNKGYQRSMETIDEYRKGEISLICTYSSDGAQFKRISSCMAVATRKTNGKVTFDNSFPSQLRIDEEMIEAAERCIDGYTCWQVPAIYSYIPPTLINIDPFHVRGAGVCKDIVQEFQKPTTWRDFKIMANKLEMLQFSVDSIIPYTKEKIKSQSYKKLIKATGRELDKLSIITSALVGITGSCKSMDFNALVLGWYYCLEFLGPVLAPQRRVRLLLDGCYKLHVVLESQSITLKWHTFYHHLLDHERIYGHLHTTEIFEREYKTIMTFCNIIEAVFRSSPSNHPEVLNFLEFFGIVILELKDFLIFKFLFSQNTSFQNS

***Caenorhabditis japonica* CID-1**

MSDEVVQGKNRNKGTAKKQPQKSKGQKEKTTQPPEKPSTTSPVQPTPQRPNHSKILQKPKLQILKRVDNSSAKPTAFPLMQVNTNPPPLMQMNKNPALRIKPSKTGPVDLPEESMNVTTRQAEANRKQTTAYYFSEKPGTAVSAHEFQESVQYRISNFPLERNMDILIDKKINLLIMPTSGRCRALYIRVKSQSTDSTVATGALLKLDANDKEIRKACKETIDNFYSVLEMSSSDRYPYCVASEEFLVSCHIKRVEERREAFPEAIYYCEKCDYHINTMGHARSHLESSTHFDEVERQQQREKLLDAIPEPTQAHLKAVNKVLKTALEEYKKLRIISNPTNSVGSKQKQLLSIYLSPQRKSLQIKEDREKLFGRISSYLSASILSVVGSSGITLMPFGSATYNAVLSDSDFNVAFTTELPEGTPIFTFLEKIRSLISQDGHKADHSMDMGTSSTIIFTYDSVRVRLCWFSNFNNRSQLYLTQLIHTYTSLKPEVTQFLQLIRIWASKAQVDSKNRSRIGLPRYGFDLMAIHYLQQSAYLPVLHEMYAEGGKESEENFRKKRDDDDEEVNKTKCSQNPDDTSNQSKEQPEEARQRRMRLMSRYLDDPEKIYAAFPSLCEPWNLAKIWIGFFQYYVRLHREVVVQITQRSPMARDLNRWNKKVLHVVDPFRSDNVLSIPKVSTWQPHYFNCLLVTYLSFAVPRTVKGPMMEWALTQNKTSSTKKKKESVTTPNCVRPQARIVTSETIDEDSIAEYWTVDDCELNQYQDDLRRRLTFDGIKLNDLKCEDVEIDEHVSHIYSCRTMRRVQRLHANDDPNWEMMGQLSENGLLKRRFEKKLFRKNRKQVDNENGVSSQVQVCTVMMSHTTLESIDAEVIGQNGVNDELSSSSYTDHNLMVAMDAECGDFRKEPKNLDEFNSDPKESNKQNTPIDLDNSVDLVETSSKLSKASEEEVHESKASTGSTFIARKTALNRKETKICTEEFFIKEKNVPSNLQSRVAEMTEADYVFNFTPESFSDGYEMEMKCTHCDGSHCVESCPMMEIPPIEKFASRTPNELKDIDEIIDKYYKEHVLDERRLELLKDKVGELEKYLQKCYREDVNLTIFGSVMTGLGVDCSDIDICLRFGEGDEPPKGITPKDVIQKVEKQLRKCNLTKSVLAIISAKVPIVKFQMRLANNDLVDVDLSYYNILALYNTQLLKEYTFWTPDNRFSKLALFIKSWAKSCDIGDASRGSLSSYAHIILLISFLQQCDPPVLPRLQEDFRNGNGESRIVEKWDTFFVQAEQKFITNWAKNKETCAQLLIGYMDYYSRFDFRNFVVQCRREMILCKMEKDWPKPLCVEDPFDLNHNLSSGITKRMFVFIMKVFISSRAAFMSHKPDCPRDSNFLGAYRDQLFRACSQGTAPNDRQCHLCHRIGHFYESCPQRKETRMRLGSKSSNSSYRSLGNNGASEEVERLVYHKRTFYRRFNR

***Caenorhabditis remanei* CID-1**

MTNKSEKPGGNDSGGGSGSKRRRGKKGKNTPSDSQSTASSSKTTSPKIQNQKQKQPEPTSSSTTPKIQILKRDPSKNVTMPLVACVMPLMEVNLSENPLPQQPPTTPKYHQNPQNHQNSQNHQNPQNPPSTKTQNQRLYQTPEKTEKSGGGMNTRRSEANKQQTQAYYFSQNPGVAVSGDKFKQPSQYKMHNFPLERLVNIFIDGKYSLMITPTNSRCNGLFIRVESQHQDYKPTGALLSLDSDDETIRNACLETIERFDKVTQKSATDRYPYSKCSEPYLKSCLIKRLDDRLDAFPDAIYYCEKCDYHISTMSHARSHLESSSHFDDIKRQEQREHLVRHIPKPSKNHLRSIRKLLDDVLDDYKKVQEIGNEKASHILYYLQTTVFPAAIGGNRNVQLRPFGSATYDTVMPDSDYNVAYTMDLPENEPIFSMLEKVRKRIVDDGHPADHSMEMGTPSTILFTFEGVRVRLCWMSCFNYRSQLYFTDLMKTYVSLREEVVHFLQLIRLWACKAGVDSKNKPRIGLPRYGFDIMAIHFLQNQGLLPILHEMYEEDKTMEFDEQSLNSAAGDDASERSRIPPEEAGPRRMRLMSKYEMDVEKIRKKFNLTKQWDQADLFIKFFKYYVEKNRDIVVQITQTAPISRDANRWNKKVLHVVDPFRGDNVLSIPKVSTWQPYYFNCLLTTYLSFAIPRTKDGPLVEISLIHSKSAATKKKVKEPQTPKRSGEPPISTTPMQNIPEEDYNRAADLLAEEAENEKYINDLKAKLMFDGIKYKDLKPTNFEIDDHWQSVYSRKALTRFRRVWPSRLNERIAIPLEDHDLLNNKFGKKWMKRWRKGELGRSESLEMPEVSKEEEEEEDKDVAEITEKMEEMLGIVESEESQRSHSEETPVTVIEKDLVSQQSEIKDESMVMSEIEENEAEKEKDASIPEDPQTSEAIVMCIRSVEEPEAPEASDDVKDSEVASKAPEAPESIVPTKPTSESSAILTTPSKSTIQKTTATFVKTKVIPVAEKQICCEEFFIKENVDFKEIAVKAKRLHQHEFQFEFSSDLFCGGYEMEMKCTHCDGSHCVEKCPMMEIPPIIKYEARTPEELKDIDDIIDKYYQENILNDARLKLMEERKTELERFMKEKYQNDVNLTIFGSVMTGLSVNCSDIDICLRFGSGDTPPKDRTPKEVILRTERVLRNCGLVRKVQSITTAKVPIVKFQLLLINDEYIDAE

***Haemonchus contortus* CID-1**

LLSVYTAIRPQFASLCRVVRKWAEVCGENAIEPDITEAQVKKKRRTRRRKLHSVENDVKVVMEKLISDLSEVSLVTGSIKLTVSGVRVRISWRCENGLKFGKLLSVYTAIRPQFASLCRVVRKWASGIYSVDRRQGGLTSYGFDLMVLYFLLQKNLLPCLHEVENFPDAIYWCSLCDFHMSNIQHVRSHFDTHQHFPEKKIGVLEKPWDLAELYVEFLCFYASRIHQNEIVQVYTAKQVSKDRSRWNKKLLQIISDPFRTDNVVTFTKAYQVYFFNCFLKSFLYFAIPQTINGPLLDVT

***Meloidogyne hapla* CID-1**

MPPCIPPGSWIRIILTNAVSHKLLKSNTILIPKKVLFCQRRVMTRCFDGQVSFAVTATRRNDICNENKFVYHSRLCDLLIDKEELIDPLIKIFGKQCEINNNNLKNEKENDDIPTKIRLWCFPFIENKEDSQLEFKLKHIDERYYTAIDTTENEYINDEKQIIINSSNDDSDCNNLNEQQQFSINFCKWFSECIERFYIKNKMNNDRKIKMDKFINLLNKKLQNNLDLPNEAKLILFGSAISGFGSNDCDLDICLINCGIDSSLNPSKTFTIRRLILSQIATLLRKDDEFMQITAVLDARTPIVKFEHLPTGFHGDLSVDNTLAIHNSELLKLYTEFDERVAPLGFAIKCWAKLYGINDASTGSISSYAYIVMLIYYLQRCSPPILPFLQQEEFIINNNSLLVNKIVEGWKVYYTKDINLIKENFKSKFTNKQTLAELFLNFLHFYGFKFSFISNIAQIRANGLYDKIQANISIKRKIYVEDPFDLNHNLTSGVSSETLNHFIKCCQQPIIGLRQLDKEIFSLNETKDDENNKLEKEKLKMAQKRYPFNLLTRSQITNSVNNMDSAGSQQSDMESSGGGRAKHHQHKGKKQAIYKTIIHSKNRHVLVENNRGNSFERNDRILQGGFFKRKNNKCSALRVVEALIGIVSVANDGGRPHSYEVDISKMERLIFDMETFPMETTRIIITTTQQKQQPSKMPMTKAA

***Meloidogyne incognita* CID-1**

MSQSSNNNNTSTNNKENGNNSRNFRPSKQRKQKENPQKILEGNKNGEYAQGFYKIVEKKYIPQDLLNKSFAGCSSSNSKIPSKNIKVITPTRQQKGNKAFNKYLQKDETKIEFSKKKNSNNFQQQNGGKINNQNKWSNQNGSHFNKNIQNNYSLIRQSPTSLLRPLKEELKIDGKSSQFNSSPVIPPDVNCSPRRRRHRPRNSIPAEINGNTLSGNEGEVFTRSLVGVILKNSLEENKLKNGAVNNWAEFLVYQDSKASFNSIIRLQADEMLFCISPGSWLLVILKNPINYKTLNFDTILVPKKVVFCRHRFMTSCFDGQVSFFVTATLRRDIISGDKNKFVYHSRLCNLFIDKEELIDPLIKMFGNNSEKEENDIPIKIRLWCFPFIQNNNCQLKFKLKQIDERYYAKINSTENEEESNDENSDFNIGEEKQLLFSTNFCNWFSECIERFYIKNKMNNDRKIKMDKFVNLLNKKLKNNLDLPNEAKLILFGSPISGFGSNDCDLDICLINCGIDSSLNPSKSFPIKRVILSQIANILRKDEEFMQITAVLDARTPIVKFEHLPTKFNGDLSVDNTLALHNSELLKLYQEFDERVAPLGFAIKCWAKLYGINDASKGSISSYAYIIMLIYYLQRCSPPILPFLQQEEDNNNSLKIVEGWKVYYTKDINLIKQKFQSKFTSNKQTLAELFLNFLHFYGYKFCIHSNIVQIRVNGIYDKIQAKLPMRRKIYVEDPFDLNHNLTAGVYSENLNYFIKCCQKPIIGLRQLDKEIFSLNEVNDQISSDNNNNNKLQNEKLKMDFSTLMLIYRPRRVTEPKKKKTKPKKELPSKEILEN

***Oesophagostomum dentatum* CID-1**

CAWDRRVAPLGIWIKQWAKSCDIGDASKGSLSSYAMIILLIHYLQNCEPPVLPRLQEDFR

GGKVESVMVENHDVYYHQKLPSGDHWSSNNSSVSQLFIGFLDYYARFDFDTLVVQIRRKK

PLLKMEKDWNRSLCIEDPFDLNHNLGSGVTKKMYVFIVRNIHNSRRRFMLSDIRREFLEK

KKLQKLQDKEMSPTLIDQYAATLLKECEMGLAPTDRQCRICHRVGHFAESCPKNQHVMRT

PRREPWSKKAPNGSAESTPRAGQSGGRRYQSARSYFQKGGRPQKLNGLPQQ

**EKL-1**

***Ancylostoma caninum* EKL-1**

KENMNEWRDWTPHIARVPPLEVEWLDSNGYRTLSQDYLMNIEGSQTQSPYEFYARPIRRERVKVPPSEISSGEEEQLQSETEAMLNAHDELRKKATVLDTFYSLEDNRSPLESNQVANWLKDNIRVFAICACSEERGAYTGEWQRVEVLSCDIFANVLFLDSGGTELVVPYSLYKIHPIHCTYPPMCMQLCMYGVGPPEADGRLEWGEGPKNEWRKLLREDLPMAISVLKRLNAANDNTVLQSNEPAWRRPGVLFVRYLKVHGDNVTTLEKFCSPSRFPNSGMFQNDMPCRWDLE

***Brugia malayi* EKL-1**

MDGRTSPRLEEPINRNDKRNAVPKMMQNGDVRGDGRFKIHRIPLCRTARCIVSHVTSPSCIWVKPVNHITEKLQIRDLNTLTPAPVAHENRYVMAPLEEGVYARARICEVDQRTKFVKVLFIDEGISAWMNSTCLAKMDEILSFHPWQAIPVALFKVKPYQDILNDNVQLKWSKEDTAALRGILKKFELVRVEAILNSVPSNDYRDFVKVNMYGMESESDEMGTSITHLFARERFDQVDFERNLFDGITQKIFENFHKEVLTVETLETWRLNFPSENETEAIVEQPILNEATVXSAQIPVADVSWLNEEGYCHKGEYLVNIEGRNTVSPYEFYARPLKMTRLKEASQRNTGIEEYEDNNAADGEEDAMIAANDELANFAEELNSFYGHPKNRKLINSSNVVIALEKGKRVYGIVEVDDEFAQFTGCWQRIEILGIKDGYYSESFFCRLRFLDSGGTDVRLLSSILEIDPMYKFCLIQCTSD

***Caenorhabditis brenneri* EKL-1**

MISVGLRLTDQVFPGPEEPIAKLPTASENPEMILDGIDKNTGKAVIKRIVLSRTANVELLRAESPSRIWVRLPNHITDTTLTFREPFELKPKTKIVQGDYAVAPIAERTFRRCRILETANEYELIKIFFIDDATTAFVKKECLAEMDEHYMFYPWQAIQISLFGVYPAIGDGETESMWSVDTCFNLAEILLGFSILRVEVVLSTVVFNDYARPIPVKLFGIEGDQLEEKEHKSRSVEVKQILEKKVPTQIVSMDLHDAAFHEIYEVAETVEIPEDALDIHQCIPADWKHKDEVEMAEKEEETEELFNKDSLTPEKEHEDWDPRQNKIEMPTIEELAENFRFPDPPGSEPRIMLHVEGKCTKNPFEWYARPIVKTGKNEREENLPWTSQKDTEEFKEVDLVDWILYGNDQLTSFAEQLDNYYSNAKNRKPLRREEIQIMRKENREVFAVCAVNEEKANYTGEWQRVLIVNCDEFAEVRFLDSGGRDMVLTSSLYRIHKQHCRFPPMCLRFSIHGVSTKVNSNKADKAWTTKETSRFRMCLREDVPIFINVSDLAPMVLPQRKDPRPYFANYVLMVKNVSYIDESATLMDRFLDTSEEGCAIKDPNEPIPWTR

***Caenorhabditis briggsae* EKL-1**

MISVGIRLTDQVFPGPEEPIAVFPGGTENPKQILDGIDKEGVAHIKRIILNRTANVEFLRAESPSRIWVRLQNHITDTTLTFREPFPLKSKLEIRKDDYALAPIDERVYRRCRIIETENENGLIQIFFIDDATTAWVKSECLAIMDEHYMFYPWQAIQISMFGVYPSVVDHSGKLEQLWSIQTCNKLSQILRAFPILRVQVALSTVVFNDYAKPIPVKLFGIPPGVEEREYHYRAMSISQILEGGLPMPAEPEEEAKDDVDSDEEEVLETGPSVRKEEVDNKPPEIVCMDLYDAAFHEIFDVAERDQIPTEPLDIHCEFPKDWKESDDAEEEEKKEKEEELYSKDSLTVKLEHADWDPRLNKIEMMTVKELGEKFRFSEDDKEPRIMLAVEGRCTKSPYEWYARPIVKTGKNKREENVAWGTDSDLREVDETDWMLYGNDQLTSVAEQLDTFYSNTKNRKPLRTVEIQAMMQEKRDVFAVCAVNEEKANYTGEWQRVLIVNCDQFAEVRFLDSGGRDMVLTSSLYRIHEQHCRFPPMCLRFAIHGVSTRLNSGREDGKWHSNEIARFRMCLREDTPIFINIADIVPLPAPMDKERRVHMARHVLMAKDVSYMDETRSLLDRFLNDEDSVNAVRNPELPCQWPPF

***Caenorhabditis japonica* EKL-1**

MISVGIRLPDNVFPSPEEPIAKLRTSENPEMYKNGIDENGVARINRIVLSRTSIVSLLRVESPSRIWVRLQNHITDSQLTFTEPFELVKKANIVVGDYALAPCDERAFRRCRITEIGRDHEGNEVVKIFFIDDAVAAWVEPGCLAEMKEHYMFFPWQAIQVSMFGIYPAMQDLTDTEQKWSEETCDELAKILNEFQLLQIEVILSSIVNNDYAKPTPVHLNGIKNDDNSESQQRSTVSISDILLKTLPNSVIIVPNHDGAGHELFGTNEIELPTRAHEIHKRFPADWKMKSVCVEQANEEAERLHGSLELEHRDWDPRQNSIPMLTIQDLKDKFVFPEKINRSDHIMMAVEGRCTKSPFEWYARPIIKTMKNILDEVVPWNDDSENLRETDKVDWMIYGNDQLLSVAEQLDTYYSNPKNRKPLKSEEIQWSQQHQSPGVVPVRRGNRVRKPVNRYDPSN

***Caenorhabditis remanei* EKL-1**

MLSVGLRLTEEVFPGPEEPIADLDTEKKDASGSIVKKDPKMHLDGIDVKTGVAKIKRIVLNRTANVEFLRAESPSRIWVRLENHITDNTLTFREPFELKPQKTFKVNDYAIAPIDERVYRRCRIVEPENERKLIKIFFIDDATVAWVKKECLAEMDEHWMFYPWQAIQISMFGVYPAVSDDIEVCLWTKTRLKKSNSCFFRKIVKIMTKFNFLEGFIMKRKDTKVMPKFSFFQTKPVWTPKICFKLAQILANYHKLKLDVVLSTVVFNDYARPIPCKLYGIEPDIVETNRTVCIAQMLESAVLLEDGTLSAKEEEDETGDQNDMESSQEEIPTDPSPPEIVCLDMYDAAFHEIFEVAEKDLIPENPLEIHRNLPGDWKQTNDLEEEERMDKENELFSKDSMSPQMEPDEWNPRLNQIPMLSIDELGEKYRFPSEDGKQRIMLAVEGRCTKSPYEWYARPIVKTGKNARDENVPWSSESGLAEVDATDWMLYGNDELTGFAEKLDTYYSMQKNRKPLMRDEIKIIMNEKRDVFAVCAVNEEKGSFTGEWQRVLIVDCEEFAEVRFLDSGGRDMVLTSSLYRIHSQHCIFPPMCLRFAMYGVVTSSGRVDKKWAAKETSRFRMCLREDTPIFINIEDIAHLPSSNAERRLPHVAKFVLMVKNVSYMDESRTLLDRFLSKDEEGHARQADGEPAIWPPAKTQFY

***Haemonchus contortus* EKL-1**

PERPSSPIFETGGKHPQMCRNGLQEDDIVKVFFIDEGFQAWVSKDEREQLESXTSAMLSAHEELKKKATILDAFYSLEENRSPLEKNQEYCMAPVRERTYGRCRILEVQCLACMPKDFQYHPWQAIMISLCGQMWTAAESASFRGILNRFPLVKTRTVKSSIVHNDYRRPVQVTIFAFHTPIEVFSPLRLSFSSIEWLRDKIRVFGICACSEERGAYTGEWQRVIKVLSCDTFANVLYLDSGGTELVLPHSLYKILTGAKRYIITLRHCNYPPMCMQLCMYGVGPSSADTSTDWPEGAKTVWRKLLREDLPM

***Meloidogyne hapla* EKL-1**

MAEQPPRIKTMGFCNRPGSPVLKSVHNRLQEMHTENGKIHRIIPKSQAICLIAHIDSPNCIWFKMVNNITEQMQLHKSAYLEPLKNSKELKSYIYVMAPIEEGIYSRARILHIQPVKYKEKRFSFVFAHFIDEGYGAWMLEDCLAKMDSQFQWHPWQAFPVSLFKFDLPKNLRSFERLNYWPEEVNDELIRIMGEYEQFKVVPVQGKLTNDYCEYTRAEIYGLDSDKDNEKEKGKAESIGHRLAIEFQPLELIDRNMFHVNQQIIAKRDGIKPLSISDAILLLQQMPKWRRTFPTDFSIKNEEINYENEEQERQEQIPPHWQLIEGQIPLVENFTLNLLKTHYSTNEGKQVVCIEWNSLKSPYEFYAFPLKQTPNETKNKEELIEKNPIRRLYNLQTEISLELNKFYGDPINRRKVDPQEVFANLEMGPYFGIYETNDEIRDRNFRRIQIIAMRKKRGQEWTTEFCRIRYLDIGGTEIVPICCILQIHT

***Meloidogyne incognita* EKL-1**

MAEQPARIRTMGFCNRPGSPVLKSVHNRLQEMHTENGKIHRIIPKSQAICLLAHIDSPNCIWFKMVNNITEQMQLHKSAYLEPLNNLNEVKSYIYVMAPIKEGVYSRARILHVQPVKYKETRFSFVFAHFIDEGYGAWMLEDCLAKMDPQLQWHPWQAFPVSLFKFDLPKNLESFERLNYWPEEINDELIKIMGEYEQFKIVPVQGKMTNDYCEYTRAEIYGLNSDRDNERGQGTAESIGHRLAIEFQPLELLDRNMFHVNQQIISKRDGIKPLTVGDAILLLQQMPKWRRTFPNDFSTIREEMIPENEEEREGQLPPHWQLIAGQLPLVEDFTFNLLRTHYSTNEGKQVVCIEWDSLKSPYEFYAFPLKQTLPEEIKNKNKEVIIEKNPIRRLYNLQSELAFELNKFYGDPINRRKVDPEEVFARLAVGPYFGIYETNDEIRDRNFRRIQILAMRNRRGQEWTTEFCRIRYLDVGGTEIVPICCILRIHTYHCNKPPLCMQFSLQTIQPTKTNNWHLDEIRFFKSQIRVDIPAICKLRLLNPHNAVTDYNSLPQSWPGVYGAYELNMGNVKLEAKMVESGNAVYTEAVNRNGN

***Oesophagostomum dentatum* EKL-1**

LPGGDDLKRDEYCLAPIDDKTYARCRVLCIEGALVKVFFIDEGISAWLSKDCLATMPTELAYHPWQAIKVSLCGVTMRKSSYYGAKSSLMWSEEECISFRQLLSAFPLLKTRTVKSSIIHNDNRRPVQVELYGIPEGYSDTSKEVGVSIAALFSAKQRGHLKTQRIANAADFVSAPNFEEKLTPPKMADVPSFRRQFPADRQQLPAKDPTALSNEKMREWKEWTPAKARVPPLELDWLDKNGYRNMLINTDEEYLINVEGAQTQSPYEFYARPIRRKRVRVAPNEFDNGEEEQLQSETEAMLNAHNELRKRATDLDTFYSIEDNRSPLESNECANWLKANLRVFAICACSEERAAYTGEWQRVEVLRVTILPMFSSWILVVPSLLYRIVSIKFTLHTVLILQCVCSSACTALDHPQLMEVLNGVRVRKLSGEGFFVRIYQCRFQCLSA

***Pristionchus pacificus* EKL-1**

MNSSLEKRWRRDSSPIFSTKRIMGKNNTEKQIRIEEKELLPNGFFTKDGVRVCKINRVPMITTAEVTVVAAHSPSLIFVRITNHIRDKLVLREPSKVAPLDEHELVEFYYVMCPIEERAYGRARILKIIKDPKDSTKKLVQLILIDDGTIVWADSTSLVSMDPDRSTGVKDFAYHPWQVQAISLAGIRPKKTPQNLDQKWSENVTKRLQRLVNGYQTFKVKAVTLSMTNNDYGVASVIDIGSMLAGGLPREVEAIRLYDGKKQERFEMRMTEDEKKQSKMEPFIEDYRKQCIMGWKDDVKENEERADSWANNLNSVEIKDWTLEELESKKYTYQSRIYISLEGAHTISPWEFYGRPIKIVVDKEEVEKVEKEGGEENVDPSGGEMQIKEAVMLGQADAMLKGNNVLKNHATELDLYYSKDGNRKQITKEEIERVHSDNGRVYGICQASESRAEYTGQWQRVEVLQTNEISAIVRFLDSGGTDMVMYGALFHINPIHTIEPALCVQLCVHGMKCAGKESWTGGGSSNEKPDYLIPHEDFLREQRSKREAFMRKGVMFVNEMKVS

**GFL-1**

***Ancylostoma caninum* GFL-1**

FIRKVQFKLHDSYAVNTRVCDKPPYEVTETGWGEFEVQMRIYFVDVNEKPVTVFHYLRLFQPVFTLPNGTTQVIAEHYDEIVFQEPTIPMYKALTAGDGKKHDRKKFHADLLQICRRTVQNVNMAKEEIQQEIDDLRESLRAAHKXTLRYKAGN

***Ascaris suum* GFL-1**

MGETGGHGVERCKSKRVIKAIVYGNTASYLGKKLENDHTHEWTVFVRPYHNEDPAKFIRKVQFKLHDSYANPTRVVEKPPYEVTETGWGEFEVQIRIYFVDVNEKPMLCFCITAKW

***Brugia malayi* GFL-1**

MERVKDKIFIRPIVYGNTAHYLGKKREEDGHTHEWTVFVKPYYNEDPSKYIRKVQFKLHDSYANATRMVEKPPYEVTETGWGEFEIQIRIYFVDVNEKP

***Caenorhabditis brenneri* GFL-1**

MADIVERMKKKTVVKPIVYGNTATRLEQKRESDQHTHKWTVFLRPYMLEDPTKWIRKVQFKLHESYANQTRIIEEPPYEVTETGWGEFEVQIRIYFVDSNEKPIVAFHYLRLFQPLIELPSGEQIVCTEFYDEIIFQEPTVQMYRALQASEGKRPDKQSFLNDIEQVKNRTRELGEHAQKEIAAEIEDLRETLKDAHKLIVKYSAELVEQE

***Caenorhabditis briggsae* GFL-1**

MAEVIERMKKKTLVKPIVYGNTATPLLQKRDSDQHTHQWTVFLKPYLAEDPTKWIRKVQFKLHESYANQTRIIETPPYEVTETGWGEFEIQIRIYFVDNNEKPISVFHYLRLFQPVAELPSGKSVVCTEFYDEIIFQEPTVQMYRALQAGDGKRPDKQAFLNDIEHIKNRTRELGEVAQKEIAAEIEDLRESLKEAHKLIVKYANECNEQE

***Caenorhabditis japonica* GFL-1**

MSEVIERMKKKNVVKPIVYGNTATPFAQKRDADNHTHQWTVFFRPYLAEDPTKWIRKVQFKLHESYTNPYRVCEKPPYEVTETGWGEFEIQIRIYFIDPNEKPITAFHYLRLFQPTLPLPSGKHLVCMEFYDEVIFQEPTVQMYRALQQSDGKRPDKTNFLNDIEQVKRRTLELAEHAQREMCAEIEDLRESLKEAHKLMVKYSAELTEQE

***Caenorhabditis remanei* GFL-1**

MSEIVERMKKKSVVKPIVYGNTAVPLVHKRDNDQHTHQWTVFLKPYLAEDPTKWIRKVQFRLHESYANQTRIIETPPYEVTETGWGEFEIQIRIYFVDNNEKPISTFHYLRLFQPTIELPSGNQIVCTEFYDEIIFQEPTVPMYKALQAGEGKRPDKQAFFNDIEQIKNRTRELGEVAQKEIAAEIEDLRESLKDAHKLILKYNAEVNREQD

***Haemonchus contortus* GFL-1**

EVVERMKGKRIIKPIVYGNTSTPFGYKRESDQHTHQWTVFLKMFYDEDPTDFIRKVQFKLHDSYAVNTRGEFFMVDSTPYFPFHSSITFSVCEKPPYEVTETGWGEFEVQMRIYFVDVNEKPVSASNMRLGASRKFSEHLHKSTVFL

***Meloidogyne incognita* GFL-1**

MASTSTNSGGVVYTERVRNKRVIKPLIYGNYAVIFPQKNSKGHTHKWTIFIRPYDRDEDLSIYIRKVQFRLHESYPNNVRIVEKAPFELSETGWGEFDIQIKLYFTDVNEKPVTMFHYLRLHQPLIELKNGQKMVLKELYDEIIFNEPTEPMYRALMKHPNHKKDSRYTGDTFSAQSYGYFRTPKDFEIERQELRNKLEAENRQIIAEIEDMKRTLKDGCELLERYRKMLNESEEKGGDAVKKEQTEKENS

***Oesophagostomum dentatum* GFL-1**

KRVIKPIVYGNTATPFGYKRESDQHTHQWTVFLRMFNDDDPSDFIRKVQFKLHDSYAVNTRVCDKPPYEVTETGWGEFEVQMRIYFADVNEKPVTVFHYLRLFQPVFTLPNGTTQVIAEHYDEIVFQEPTIPMYKALTAGDGKKHDRKKFHADLMQICRRTVQNVNTAKEEIQQEIDDLRESLRAAHKLTLRYKAGNDPDSNGSTPEVSICQ

***Trichinella spiralis* GFL-1**

MSRSPAKVKKSTPTAGTARVSGVKIVKPIVYGCASWSLPKSHLVNDRTHGWKLYVKPYFEENLQLFIRKISFTLHSSFAEPTRSKTTILLCSEPPYEVNETGWGEFKAVIKIYFKNSCERPVTIYQTVKLFSEKGVDYTIKKPLIDERYDEIVFRNPCANMYSGLMDAEESPKKIGHHAHDYEQKRKEILENIQKARQDVQKEVSLLEGIVRKYEDALEKAKI

**MES-2**

***Ancylostoma caninum* MES-2**

MTPPSAGSGRVAPFSNPTLMNILVSLLAGEKTSICIITAQMKMICEDIGAQARTCSEIYRLASQLAQANPSLTPEQKKVSIKDKHRSFRSFTWADGKGQVENKERMIPCSHNGHCDGNPLCVCSSGSGICSKFCGCPQDCRMRFPGCRCAPGNCRTKQCQCYFARWECDPDLCKSCKCDDLSTDGEKCRNVPLQRGHQKLLKVGISGIAGWGCFIQETADKGDLIAEYTGEVISKWESERRGLIYDKFCTSYIFGMNNDQFIDATRVGNLIRFANHSNNNANCSS

***Ascaris suum* MES-2**

MIDQVAGWGCFTEEDIAKNDFISEYCGEVISHDESERRGKIYDKKKCSYLFGLNEEYLVDATRKGNVIRFANHSKDPNCKGRVFMVNGDHRIGIFARRNIAAGEELFFDYSYNSTQQVSELLAY

***Brugia malayi* MES-2**

MDTPNNSCNIGKRGIKNDGQVSRGAKRRCFKKDQLKEDVENEDSGEEKSFRGWKEDEDAPLPKYLRDDVTRIYEDVMSRYRNMIDAEGLKLYHKMLQEEPRKKYIKCPSKCIEAVTSTPRMQYWTLTECNILCEDERTLSHIPFLGDHEDDDIGFGEELLKTFQEGIHGTKVGCGSFINDHILYQVLKKLFDKYSDVGIADQMIYRAVYEQFPNKASVQQLPFEDLKRRFGPSDLFIEDSNQSEFLDTKALHSFQLLLCSRCLTYDCLIHGVNATETEVRRRRGVTSVEPCGLQCFRHLTKEMEEAKRRCASPPDAKTINAILNIKTEDINWTAQQESMFIALRRTYKNDFCKLSEVLNLVVGNAPSKSCRELYAYSFRTAPISPRADVSPNSPPKKKKNMKDQHRTFRAVKWAKTEGKVGNTHVYEPCSHIGPCSAENNCSCVSVDNLCTKFCRCGEQCKYRFPGCRCAPGLCRTKQCQCFYANWECDPDVCKSCKCDILDDPNVATCKNVAMQRGLQKKLVIAPSQVAGWGCFAEEDIEKNDFISEYCGEVISHDESERRGKIYDKLKCSYLFGLNDEMVVDATRKGNVIRFANHSKDPNCMAKVFMVNGDHRIGIFARRPIVAGEELFFDYSYNSYQQVKFVSKERPKP

***Caenorhabditis brenneri* MES-2**

MDADRKKKKAEKRTITVSNNEVDEERNSDNIKIRKNSKNPQSALDGPLGARRVANIQSIFTEKVDTVESINQDVKRIQDTLMLHYEDIKKEHDDVAKEETENNHGNWLAAPKKRNYHSENKRNNFGKRVHSNVNELAQKCPYSRKEGVIYLTPIHDDKGNKLPKLNKVQCVRTEVGDILPYMNYWASIEQTLVSDDQLRLSHLPPIEEGVSEKEFYSKLAKHFSDGIHGSSVDSSCINDWILYRLFRKVLPCFNDTPDAFYYAVYCLWPNKLSQRQLSFAFPNWCDLYAENGFNLFELEHWKKQNCLKIAENVQNLSCYACFNYSCIEHGFKAQFPTRLPEGTYAAVNLEVCSESNTTNSQCSNTCWKSVDKRKLKWRTLSHQEESGGNKVEVYLDKLDMLRMSHEEGGSIVSAFVFNHNLPFCDFAQLAINSYHGKNAEFKKCSGIYELILEKCQKLSEQRYNLGINSNQLSKQDRVNKFRKDQRIQAKAKIAACSEAERITTKAKKNKQTVDLSLVTPIVPCRHSGPCSSTTEYCACRENGICTYLCECDINCPQRFPGCNCSPGQCQSKACQCYFANWECNPITCHNCKCDNIDEEGLICKNFPMTRNVMKRLTVAPSKIAGNGLFILDSAEKDEFITEYVGERISEDEVERRGIIYDSTHCSYIFNLSSGGAIDSHSLGNISRFANHDKKHPTVYAKTIVVAGELRIGFFAKRQLSPGDELLFDYSYSMEHQNAIRQVNFVHFLTRQFDEKLLWALQKIQ

***Caenorhabditis briggsae* MES-2**

MEAQEGRKSGPRKSKFTMDTPSERPKKKARRRLEAEKHRESSDVYVEESQRAKKMDFQKITGIQRIYGDVEYKNGSENAEVDAAIAKIKEIYVPIRQEFNKMVEEDRKEKLRPWQAKADEAYDPRLRNLGIRMRNLQVAEWEETPDGWIPGFGETIVRDFVGGAKPYSSSSDDRDNRPNVKRTVNVLKLPTGDIYPPMVHWIPIETSIPMADQLRLTHMPYFPNASDDNDMYEKLIDHFPDGISGFAFASGPVRAGGGPADKLSLKRGIQTVLAPNDRSRQGSSRLYHSSRIPVRKMCLNDKKSAFCQIGRKLSFNPRLRHVIRPESDSNGIGWALSRCIICQLVVYQQLFGIQSDAKSSRIIHWTTGYPLLCTLKIWPNKFSQREFSTVFPKLCELFSENESDWEALEPWKSDEFMNGIADNLDNLQCYSCLTFACGNHGFRCQLPPEHPNGDVSLVHLPLPTDQAPPGEKCSDKCWRTLKKEKIHKILTPSAEEISEGQAKIWLCKESIGKMSIENGAMIAAMFLLDQSDTFCDFVKSYVREGSEPRFKTCRDIYELIASLAENFSDRQILIGQAPKMLPHQARDNAFRRLFANKEKKLSESETQSNQDSASINGTDSESTNGMEDRKPTRPMKTGKKSLFDTTLPFKGCRHNGPCGPDVLECSCRENMTCSAHCHCDKNCKQRFPGCACRPGQCNQNKCQCFLAGWECDPLTCFNCKCDDITNPKSCKNIPMTKMLQKRMMCCPSGIAGNGLFLLESVEKDEFITEYVGERISDAEAERRGAIYDKIQCSYIFSEFLDKNFYLSSGGAIDSNKLGNLSRFANCASEKDATLYARTKVIGGEHRIGFYAKHAMEPNTELTFDYGYSFDNKEKMRRERRVSPVELPNEPSSDDVASTSTSSPMELSEPLKPITSKFGHYDPDNDNHLYF

***Caenorhabditis japonica* MES-2**

MGVNTTSPKPRRPNERERLQETDSFVVHPEDPTTLRAKRHTERERLLSENADSDSDSSPSPAKTSKKREPPAKKKKQEQDEESGKTKKKIVETGIIKMVAIFGSEKAKTKALSKKPEMEETLAKYKEFYSEIKTEFDKAVDAELAEEFTHWFQEEDQYIPKDSKHTMEKCPVNLVHENSSIFWDKPETDDDGAMNRKNKHKKHQVSHTRIECGDLLPPMNYYVPIEQSINTKDQLRLTHMPYFCDGQADENLFDKLVDVFPDGIHGFSDNYGYVNDWLVYKLLRKALEAESTPTCPDIIYYNIYRVWPNKLTQRELSVEFPKLCERFAEEKLDPRVLEPWKPHAEQEVALNIRNLDCYSCLSYMCPIHGFKAELPPDFPSGDFYQVTVPLLKPNDTNFCSSKCWKHVKLSKVADVLKPTEEEVRNFKVTMYMDKARLIKMPAEDGALLASIYGAESKIIRGETPRLIVSFSHFGKKDVH

***Caenorhabditis remanei* MES-2**

MSSDKGHCKIQKIWSDSEKPARELHRQYVLLEKFVQDAYKEEYREYENKVKPEIKAFLDEWTTGPTPINHYRTNLIERVGPIPELEINEFESPKDVKEGKISWIKPDCDWDGVVHKNELVPTGRIDIGRKYPRMLHWVHITQSIVAKDQLRLTHMPFFENGMDDSQEYEKFAKLFTDGIHGFTKNWKYINDYLLYKVMRRVLEYGYTANVDVFYYTFYRLWPNKFSQRQLSEVFPKLCARYAEEGFDWQSLEHWKPKPEGMYGLNPYMEKPAENVQNPTCFACLEYLCPVHGLRLQIAPEIPGGDIAEVFLPLPGTNQSTVACGGDCWKTIDPEEIMAALTPDEEEIEEHRVKIYLDKGKLMEMSIPEGSMVVTLYIYDKSGQSFCQFVHENLHGKTDDNDKIRTCRDAYRLIMGLAEYVTDRRIQMGQTQPMRPYKERYNAFRNIQMRNHQQATKAREAALQERANSEGKTLQQVRKEEEREFMRKHGRAMNEKIMISSTRPFVPCRHEGTCKDDPDCSCQENGVCSHLCKCSMDCPQRFPGCICAPGTCRNQHCACFRANWECNPNTCKNCNCETIDGTADEVICGNFPLTRMVQKRLYVAPSRISGFGLFLMENVEKDDLVVEYVGEKISDGEAERRGAIYDIFKCSYILGLESGGAIDAFKVGNLSRFANNNSVNPTLYARAKIVNGEHRIGFYAIDALKAFTEVTFNYGYHKEHAATVSSTLGKKGRRSAATSSKPSTSSKPSTSSEMDYSDFDL

***Haemonchus contortus* MES-2**

ERFAPGRTPSKSTQELRSRSDQNFHSVNVLCCERCYQYDCVLHGGIHGADRGCDKYINDYIMFNMLEILKQDWEDHIYYAIFKLFPNKLSHRQLVTAYDLIAEYTGEVISKWESERRGLIYDKFCTSYIFSGNCRTKQCQCYFARWECDPDLCKSCKCCSHSGHCDGNSQCVCSSASGICSKFCGCPPDCRMRFPGCRCAPGK

***Meloidogyne incognita* MES-2**

MSAEKRKRKRSSFEDDLSSNIMDEYWKVRKQYERKIAVQGLSFESSRKISPKTVNFNFIPLQIPEYNQVKDNQSTILRRAETGSVVNKCPVIPLEHVESTPPMQYWTHTEANVIAEDEYTLSHIPFLGDSVDDTSFCLDLMKTFPEGIHGTKDGCGEYINDFILYYTVKAVKARLKDTGKIFVIKFNSFFTDLKSIFKYVYQCFPNKASKHELLTMYPDLQQRFQPETITRQEFTNEEGELVANFSSNRLLNSYEVLLCHKCFAYDCIMHNATIADGYGGGNRTRKRNAFSERNKSKKNTEVPRPCSLQCYMTVERVKSPLSNQKGNENSKNGWTPHEESIFAILRKTGEDDFCKITKMLNVCTNSSNKKTCIEIYDYAARAGPLSPRLELATSPIKNSKYRKNRDSHRTFRAMKWANTNGKVENGGMFKPCQHKGDCREEHGCYCIRVNNLCTKYCGCSDECENRFPGCRCAPGNCRTKQCQCYYASWECDPDVCTSCNCDKLSSSGEPICKNVSIQRGLQKRLIVAPSQVAGWGCFANEDIEKNDFVSEYCGEVISQAESERRGKIYDRIKCSYLFGLNDEQVVDATRVGNVIRFANHSKNPNCRARVVVVNGDHKIGIFAQQSIKCGEELFFDYAYNKIQQVEFVPKELKSIVKHRERQAGRSVGRHSAIREDSI

***Oesophagostomum dentatum* MES-2**

NCRTKQCQCYFARWECDPDLCKSCKCDDLSMEGEKCRNVPLQRGHQNCLRBBSGIAGWGCFIQETADKGDLIAEYTGEVISKWESERRGLIYDKFCTSYIFGMNNDQFIDATRVGNLIRFANHSNTNANCSSEIKIVNGEHRIGVYASRHILFGEELLFDYNYGQTWNKFVPIEKSIRAQANRSTPDSDCSRTGASQNKPEKLRRSVLKKTVKGEPRLSEGGKIDSAKHDDGSHKNGKQKTKMHKKV

***Trichinella spiralis* MES-2**

MDILYSRKRLVGRTRVEKSAEGTPTLEETKMMMDIYDEIMKDYKQPVSKVKVNDSYLSNYEALVQDLWRDSQRMPYPNEDRVSVVDVDTVSCFNEKSENPSNSYNLFVMKIDEKTNSHTKNWMMIPKNFLGRPCYDLRTLRDDQQENNDHRFLPDFAFYKLVLKASEVFYNQNITDQWSGELILPNDRLFTIIHHLFPHKLSANNLKELFIHIFCMLNEKKGNNKLQHLNDRESIFVKAFCPICFQFCCTTHGESLVDPMYVHNYNPKLNKKSKVKDPCGKFCYLKSLDKNTVRVLRHNSSKSKASKVKLEWTAEKEEMLRALESLFGSNSCIIAECLNTPYCSEVYARIVKSESVASTSWKIEEEKWKMPEIPQKMVKSEAYSTRSCVNNKVLLYSSGSSDTDSDWDSDGEGMEAYKPCYCRGRCRDNANCSCNEREYCEKYCRCSDNCSKKYLGCNCKGVCHRKVCLCMKNNRECDPTLCKNCGGNQLLICKNDFMQNGIRRRLYVCESNVHGLGLFTTEDIAAGDFICEYRGEILTKAEAQRRGKIYDSRGMSFLFMLNTDFDLDATRFGSVARFINHSKIPNCVPQVKMVLGSHRIAFYATRNIEANEELFFNYGVLPECVKLLLERLS

**EKL-4**

***Ascaris suum* EKL-4**

MSYYADSSLTREHSSLRFPEFRSAGAHLRSQEMKLPTNVGQKKLKNIETVIEKLKLDLVPVGAEEIVVGYNEFRSSIVLLQELKHALQTAEYELESLRTRYQAATGKTFEIEPRMRVSSMGDSSMFGEDKEGTNAGLPSSTRTITDMIELSSSLPQAVSVSL

***Brugia malayi* EKL-4**

MAFRMNTADAQDILGLSSSAGKDDGLIPAGGLTDVDRKHKKSHRSDAYFKRPEGMHRELYNLLDRERNFAALMPTTTKNTGYCHQKARIGMKRVRPWEWTPFENAARTDGLKLNHWKRADKVDDVYPFARFNKVINVPTFTDAEYDKCLNSAKWSKRDTRHLFDLCRRFDLRWVIIVDRWEGSTRRTMEEMKERFYNAINELHALKNETADALYYDAEHEKRRKEQLIKQWNRTEQQIEEEEMLIAELKKIEVRKRERERKAQDLQKLITAGERTPASPSTSTVSVVPSSNMKKSHKSRLLKTSSIPNPSISASFIQDHSNLRFPEFRSAGAHLRSQEMKLPTNIGQKKLKNIETVIEKLKLDLVPFGVADIVKGYNEFRARIVLLQELKHSLHSAEFELESLRTRYNALSGKTFDIEPRMRVRTASESSVTDAPQLGTEGAPTTSRTITDMIELTTSLPQTMRKRKAPSSSTSPSPVDNRRLRKS

***Caenorhabditis brenneri* EKL-4**

MIGDVQQILQCSEPSTSNVKKTPKAGQIQKKPEGMKRELFNLIAGKDLTSVMPTDVKKTYKQKFQTGFRAVRKYKWMPFINEGREDGLQLHHWVRSDRIDPETPYPFAKFNKSIDVVTYTDDEYNACMRHPKWSREETDYLFEMCRRFDIRWLIVYDRYDCKKFGVNRTMEDLKERFYNTSYDLNMMRDPCSSQANFDAEHERRRKEQLNKQWNRTPEQLKEEEDLTAELRRIELRKKEREKKAHDLQKLINMTEQPASPSAGGVGGAATAKRKNVFRTKAGSISVAMPMFNPNDMSTTALRFSEFKSSGAHFRCQEMKLPTNIGQKKLKNIEVVLEKCKMEMNPVASESIMKTYNDFRSQIIMVQDLKSAMQTAEFELESLRTRMQEQGKDFDIEPRFRISQLNEGGLDEDAVGGPGQAATARRITSYVDASNKDITQIASRKRKTLAVTPTQNAAPSTSMAMGGDPKRPRKI

***Caenorhabditis briggsae* EKL-4**

MIGDAHQILLGGAADASKETTSKKTPKQTSFRKPEGMKRELFNLIAGKDITSVMPTDVKKTYKQKFQTGFRAVRKFKWIPFTNEGRTDNLMLNHWVRSDKIEAQTPYAFAKFNRVIEIPTYTDEEYENHLKIAKWTREETDYLFDVCRQFDLRWFIVADRYDCKKFGVNRSAEDLKERFYQIQYELQLLRDPSSTPTGYDADHERRRKEQLNKQWNRTKEQLQEEEDLIAEMRRIDQRKKEREKKAHDLQKLINMSEQPASPSTAGFSGAAAGKRNKQFRTKAGSISMAPGPLFNPLDISVTALRFSEFKSSGVHMRGQEMKLPTNIGQKKLKNIEVVLEKCKMEMNPVASESIMKVYNDFRSQIMLAQELKSAMQTAEFELESLRTKLQEQGKDFDIDSRFRISQLPEGGLDDDMVGGPGMASTARRITSWIDCSSSKEISTANARKRKATATTPTLTSTPSSSSMGGDPKRRKNN

***Caenorhabditis japonica* EKL-4**

MNMDVHHILQGSSKTDKDNGAKTINKPPKKVYEKAVKKPEGMKRELFNLISGKDITSVIPTDVRKSYKQKFQSAFRSVRKYKWMPFTNEARKDGLMLHHWVRADKIDINTPYPFSRFNKVIDIPTYTDEEYENHLKSPKWTRDETDYLLEICRQFDIRWPIIHDRFDSTKYGVSRSIEDMKDRFYSILQQLASLKDPNANPIAYDSEHERRRKEQLCKQWNRSKEQLQEEEDLTAELRRIELRKKEREKKAHDLQKLINMVEQPSSPSTSGGNGAATAKRKNAFRTKTGSVSTTTSTFFNPLDISVTALRFSEFKSSGAHLRCQEMKLPTNIGQKKLKNIEVILEKCKMEMNPVASEPIMKTYNDFRSQIMLLQELKSALQTAEYELESLRTRVQDTGKDFDIDSRFRVSNLPEGGLDEDQVGGEGNATTSRRITSYIDANMKDLTTVASRKRKVPAVVTPTTSTDVKRVRKI

***Caenorhabditis remanei* EKL-4**

MIGDVQQILQGSEVPKDVVKKTPKPSAVRKPEGMKRELFNLIKGKDLTAVMPTDVKKTYKQKFQAIFIIIFTGFRSVRKYKWMSFTNEARTDGLMLHHWVRADKVEAMNPYPFSKFNKVIDIPTYTDEEYENHLKIAKWSRGETDYLFDTCRRFDIRWPIVFDRYDCKMFGVNRSVEDLKERFYSINYELNLLRDPSSSPTAYDAEHERRRKEQLNKQWNRTAEQLQEEEDLTAELRRIELRKKEREKKAHDLQKLINMTEQPASPSTAGFPGAATAKRKNQFRVKGGSISMAVGPLFNPLDISVTALRFSEFKSSGAHFRGQEMKLPTNIGQKKLKNIEVILEKCKMEMNPVASESIMKTYNDFRSQVMLVQELKSAMQTAEYELESVRTKMQEHGKDFEIDPRFRISQLPEGGLDEDLVGGPGQAATSRRITSYIDTSGTKDAATIQSAAARKRKATATTPTLTSTPSSSSLADPKRPRKV

***Haemonchus contortus* EKL-4**

KVISIPDFTDEEYEKYFKVDKWTLEETRHLFDVCRRFDIRFISNFKTNIMITFRWPIVHDRYDREKYGLRSMEDLKERFYAIVNEIALLKFQLGGRPVRKYKWVPFTNEARNDSLQLYHWQRVDKLESPEPYPFAKFNKVFKVSISLNFLSCLQLFWFKVISIPDFTDEEYEKYFKVDKWTLEETRHLFDVCRRFDIRFISNFKTNIMITFRWPIVHDRYDREKYGLRSMEDLKERFKYSSIRFHLANFVLVDVAVSSIRFPEFKSAGAHLRSQEMKLPTNIGQKKLKNIEVVLNKCKLDLNPMGLDTVVAAYNEFRSLVIALQEMKTMLQSTEFELESARGRLIEEGKQVGVLIQDPTCEPICFDADHERRRKEQAIKLYNRTKSQVFDIEPRMRISTLPEGGLDPEDVTGEDGAPASIRRIASYIDTSSSSFKRPEKMHRELYNLIGSSDVKEASTVVPTEIRRGFAQLNAQVGFQVKEEEYLAAELKKIEARKKERERRAHDLQKLIN

***Meloidogyne hapla* EKL-4**

MLTGDAQDILGATSMSRRQQSSTLPNFNLTNVDKKTIKKPDQVKRPAGMHRELFNLFVHQGKDNNKEKIESLAPTNTKHGYSVVKADLGKRIVRKWSWRPFLNEAREDGLQLFHWEREDLIGQPYQFARFNKQLDLVTFTDVEYDEHLDDERWTKEETIHLLDLCHRFDLRWPIVEDRFDRERFKNRKSIEDLKERYYGIVNELNASRNTQSEPLAYDAEHERRRKEQLIKLWNRTEEQIKEEEELKEAVRKIEAKRKEREKKAHDLQRLINATAERISVSPDSSTCGSPSVASGSSASRAAHRRSLKRLRTQASLTSGQLMPSEPHIRWLEYRQPGPHLRTQEMKLPSNTAQRKLNNIGMVVTSLQIVSIGLEYPPATEEIVKLYNDFRSNIVLLQELKTAVYNTEQELDQLAHRLKSEKNIELPIESRLRVSEAFYEELSRNPASLLNEINSIEGDGKTLGKGGKGGKQQKQIPKLAPVTSRRITKMIETNPISMTQTRKKRTAITAEGTSTRLSNQ

***Meloidogyne incognita* EKL-4**

MTGDAQDILGTTSMSRRQQSSTLPNLNLTNVDKKTIKKSDQIKRPAGMHRELFNLFVHQGKDNNKEKIESLAPTNTKHGYSVVKADFGKRIVRKWMWKPFLNDAREDGLQLSHWEREDLIGQPYQFARFNKLHSLIWNMMNIWMMNAGIKKRQFIYSIYATDLTFDRFDRERFKDRKSVEDMKERYYGIVNELNVARNTQSEPLAYDAEHERRRKEQLVKLWNRTEEQARKIKEEEELKEAVRKIEAKRKEREKKAHDLQRLINATAERISVSPDSSTCGSPSVVGGTSTSSRAAHRRSLKRLRTQASLASDQLMPSEPHIRWPEYRQPGPHLRTQEMKL PSNTAQRKLNNIGMVVTSLQIVSIGLEYPPATEEIVKLYNDFRSNIVLLQELKTAVYNTEQELDQLSNRLRTEKNIELSIEPRLRVSEAFYEEFTRNPASLNEINSIEGEGKSSGKTGGKGGKQQKQLPKLAPVTSRRITRMIETNPISMTQTRKKRTTITAEGTLTRSSIQQ

***Pristionchus pacificus* EKL-4**

MSFTKVQRQMAADIPAHGHLRFTEYKSAGSHLKSQEMKLPTNVGQKKMKNIETILDKCKLDVFLSQTPNVCKLEMIPMGATEIVAAYNDFRNNIVYLQELKQALQTAEYDLEGLRTRLVQQGQPAFELEPRMRISTLPDGGYDPSSLQGEGAPTSLRRITSWLDINQPAGVVNVRPRKRKLSVIPPSPMELKRSRKLTDQ

**MES-6**

***Ancylostoma caninum* MES-6**

LRSIKDPSPDMDIFTLTWCYDITDRAHRIAFGGYSGLIRLXDPCSGXLLMNMYGHGDHVNEMRTDPNNSMIFASVSKDTTIRLWNIRVHGPIAILGGYEGHKDQILSLDWSLDSKYIVSCSMDHSIRLWYLGTDKLQERIRDSMTLKGQANFRKELADDCQSGRTIQIHYPIAINTDLHNDYVDCVRFLGHYVVSKGSDMSVVVFRFGSFGEEFYKIRPKLQVDTSALQLVRMDLPDSDIWFIKFDIDPLNRWIVSGNKMGQLCFWDLTEGLPGVNMNVTVKIAECCIRQICFGANGRIMVAVADDYSVTRLERILEGEDIPPYCKSAGLSSVNGPSAKRAKRRSRKQGRKRNSDSGSSENSEDRAS

***Ascaris suum* MES-6**

MGNQRFVGKDEAFFTVCWACDTHSNAHVVVAGGVRGIIRVIDFDSATLVANLIGHGDAINDIRVCPKDSAIIASASKDFTARIWHIRNSACLAILGGVEGHLDQVISVPLV

***Brugia malayi* MES-6**

MKLSASLRSPPRIPFKHLTTVYEGHKKTIYGVAFSPYLISNPHFATVGENRISIYAVMKDGNGVKLLRSFHDSAKTEWFFTVCWAYDTENDVHVVIAGGNRGIIRVXDVVTGDLVNSLIGHGDAINDVRVFPNDSMIIASASKDFTARIWNIHNSACLAILGGVEGHLDQVISVDFDAESEYLASASMDHTVKLWYVGKGSGVDRLVEQSKADLRLVDFPAEIHYPRCSTRDVHTNYVDCVRIFHRLIFSKSTENEIALWKFGDFDDLVAGQGNKVKTETCVIHFRQMELPETNMWYIKFEIDPLEKYLVCGNQKGEIHIWEINNGSLPSVKSNHVLHPKDVGCAIRQIAFSPCGQHMIAVADDASISRFARRT

***Caenorhabditis brenneri* MES-6**

MSSKRNIQLHDIGPGAKKLKMEDFNEEKPFIVTYHLLEKNRFNYFGAAFNQFVKWPQNPIAAVVAGDLVKVYEFPVNEAKMKLIKSEKYQFKFTENQAFWAVAWCCLGADQYKIVAGCESGRLFVIDFTTMEIEKDFNDCGGAITDIRTSPITPSMVAVSSDDKTVRIFDIRATAALIICGGARFHQDRVQSVDWTPDGKELVSSGIDHRVMCWDLATKRVQDHLEYCAGFLDQGLEIAPTNEYEGNGQLEQARRVFNPKGYTLFILTPSHAITNLHHDYVDCIRVFRKNHRNYLLSKACGKESAISFWRFGTYGDVKENVDDREPATSHVKIGAKSLKGGVEWFCKFGVDPLRKYIGVGGRGGHLQFHDLQNWEKEEPALSIKFKTAAIRQVVFSDQGRIVLVTGDNGFLCRLDRVQSGAQQTRNIWN

***Caenorhabditis briggsae* MES-6**

MPPKRQTPKPENGPKEPSAEQLKVDRSFAAMKLEKCSGKEDLEDRPFVFTAESQLEQGFPLYGCAFNPYVKPQHRQMVAVCGGIGAHVFLVPHDKNRLEHIWGVSFEQPADPTKKDRKEELLTVTWAYDTYDADQGRAAFRVVVAGVLGHIYVVDFKTRNLCNRLRSYGGDINDIRVSPADSNLIAGASSDQTIRIHHIRNQGALITIGGPFSHPGPVLSVDWNSEGTYLLSCGFDHQVMKWDLTAEPAKSWLEKTCKELEKGKKDIYFQSGLDQKSDNTLELYTPVAQISDLHHDYMDCIRVLPDSDCFASKSVSYDPHLNISKLGLPGNMRTHDRGAPLEPERNAFPLMWFAIGEGKRWFHKFSIDPKRRWIAGGGDEGSIMFFDLNDEQHTEDGKYITKPEPNPPKPYSDQPASSESLPHPSRVRYYKVSDVGIRNVDFSPCGRYVVAVTEESSIIRMDRVAEDVAVDKLTEYLGVMGITK

***Caenorhabditis japonica* MES-6**

MEAKKFKKLDLHGEQHDKKLDNPENPFIMTAKIRLPQDSVYNCDFNPYIGWEQTQVLATVGGTKVLVHEVPRHVNHIITRYGCTIASSTPNQEPEDLYAVTWALDTYESPQNAHRIVTGGLHGQLYVINSRNGQQQNRLQSCGGAINDIRTSPANSNLVAVASKDQTVRIFHIRNDSCLAVMGGLNCHRDQVLSLDWDRDGNFLVSCGMDHLSMRWDLAKDHVRKHLDACCEALAKGRMNVLSQSDPVLKKVEDKKEIIKENVKKSAFEIENYHPNLEDDSNIDSVLGEIGKASGCTLPIYRPASVCSDVHEDYVDCIRVMPGINYFLSKGCGKEKAVNMWRFGPPKGVVERVTSSMAPQKTTTQLLAFKIWNGDTWFTKFEMDPRRRWLAVGGTQGFVNFFDVTKLKSHEPVLRVKVFNGTVRNTCYCAQGRIMIAVGEQGAVTRLDRVPLSIDTAELAKCIPK

***Caenorhabditis remanei* MES-6**

MEHMRRFKELNLQNTEGSQGDFSSRAFTNTASIQQYQKGELLGAVFNPYAAPEAEQHFAVVGGEYVQCYRVVKDVNNLEHVWGIHFPNSPDEKKSHRKPDDPTSEELPVDDRKESLYCVAWAFDTFDHKNGGDPYKIICGGVLGFIYVVDFATRQLDNRLQSFGGDINEIRTCPTNSDLIACASSDQSIRVLHIRNSQCLICIGGLASHPSMVLSVDWHYTGEYLVTGGMDHQVMKWDLSTFIVKSHLKYTCDELAKGKRNIFSPQVSKPPQIKPVPPRKMCPDGTGKVKQVMASLDYAVDKVYHIYTPMAVCSDLHTNYVDCVRFLPGSDVIVSKDCGEQPTVNIFRFGAGVPRNEDAIPMKEPETCTTKIMSVTNDNGEVWFTKFAIDPRRRWLVCGCTRGIVNFIDLKYRDRPKINFSLTICQNTIRQVDFSPCGRFMVASGDDMRIVRLDRVPDTVDVSLLAKFNK

***Haemonchus contortus* MES-6**

IQIHYPIAINTDLHNDYVDCVRFLGHYVVSKMDLPDSDIWFIKFDIDPLNRTTKIAECCVRQICFGANGRLIVAVADDYSVTRLERILEGEDVR

***Meloidogyne hapla* MES-6**

MPPTSKAPYSYSSSFVESHKTTVYAIAFNTFTPQEETSSNCNINYFATAGKNKVSVYSCSSDQTGIKLLRQFDDQDAKNECFYAITWAYNLDTSLHVLVVGGHRGIIRVISPHNGRMFCSLLGHGDSINELRTSPTHPMIVASASKDFTARIWNIVHRQCLAILGGVQGHRDQVISLDFDATSHFLATASMDHAVKLWHIGPGTEVGDAIQASLAKTNEPAPQFPTELHFPICNSRDLHTNYVDCVRIMGDFIFSKSSEDCITLWKFGTFEEGICGKGSLKCPETFASHTVIMKMPNTEMWFIKMAVDPHRKFLACGSQQGEIRIWRLNSNRLPLAESDYFLIPSNKELKGCIRQIAFSPDGKIMMAVGDWGLVIRFDFNQDNSN

***Oesophagostomum dentatum* MES-6**

MMSNEHFMETDTRSTSHGCSRMLDISKPSSIPPDDFSHPFLASKTVYEQHRQPIYACAFNPYQPEGYVPLLATAAKNMITIYECPLDSNKIILVRNIKDPSPDMDIFTLTWCYDITDKAHRIAFGGYSGLIRLVDPSSGKLLMNMYGHGDHVNEMRTDPNNSMIFASVSKDTTIRLWNIRVHGPIAILGGYEGHKDQILSLDWSLDSKYIVSCSMDHSIRLWYLGTDKLQERIRESMTLKGQANFQRELADDCQSGRTIQIHYPIAINTDLHNDYVDCVRFLGHYVVSKGSDMSVVVFRFGSFGEEFYKIRPKLQVDTSAIQLVRMELPASDIWFIKFDIDPLNRWIVSGNKMGQLCFWDLTEGLPNVNMTVSLKIAECCIRQICFGANGRIMVAVADDYSVTRLERILEGEEIPPYCKNAGLSSVSGPSAKKAKKRGRKYGRKRNSESGSTENSDEHGS

**RHA-1**

***Ascaris suum* RHA-1**

MTLEAISARLLEEEQKRILLQSIREQRESLPVFHYRDVIIETIAGNPVTLIKGETGCGKSTQICQYLLEDFVLKNRGADFAAIVTQPRRISAITLAERVAEERGEVLGTSIGYGVRSQLFMVLECHCVLTEHCLAGDCMLLWFYKRHNLRFESVHPRPYGAVMFMTVGVLLRRLESGLRGVSHVIVDEIHERDINVSLRRFISYLQILCMWSNVVITILRLESECEEWPSSLQGTDFALIVLREMVRQYPEIRVVLMSATIDTDLFTNYFGTCPIIQLQGRTFPVQRELLSQFVLDCFNLFEGIAFRFYFKNTEVGKSPDFEILKLIGIDFFLEDIVQRTRFLPPAPSAKKKGRDADEEGEETTHDGEVILCSLSGLDDKLCWSGSGDYRKEELMLNVEAVSAYASNWEVMFDFYCKSNKNMNMIVGDEYGPNTKLAMSRLSEKEISFEIIEALLMDITNQGDEGSVLIFLPGWNIISMLLSFLTNHPVFSKRPTDSRL

***Brugia malayi* RHA-1**

MSDEIRGWLYGWLGKRKLGTPSYSTTPLANCGGKTRFRCELRIPGFTYLFPIIFASTGVIDIRINAAWSKTRQHHVGLGISVNKKDAATKAARDFAHFLIRQKLLDPTELPKLTASMLEATNLDSFGRDSVEDVILLNKAEENSYTTTNVSMNMQFIPSSRIKTEHQRYIEQKAEEIALSESVDLRTHIHGGWTADNSKMHLNEFVQKIKQPPLRYDIRSIGTDNSRTFVAEVSLFVPKVRCTFSARAEGSTKKTAEATCALSLMRQLFHNQLVGAYTGEKKKKTAGNLADIPVIVSDELSKEIAHYLALVGVDEVQPSPEASSSNPVSLLITQKLNHFEPSQPISDGLVSWSPALENWNPWKSLNIDEAPLAFMSLEAISADLLEREKKRVIPSSIKTQRELLPVYQYRDQLIDAIRNNSVTIVKGETGCGKSTQVCQYLLEHYINNCHGAEFAAFVTQPRKISAIALAERIADERGEQLGVSVGYAVRFDSLHPRPYGSLMLVTVGMLLKRLELGLRGISHIIVDEIHERDINTDFIMIVLRDMVNMYPNLRIILMSATVDTNLFTNYFGDCSVILLKGRNFPVQYYFLEDIVQMIRFLPSTDKLKRETKGGRDDEGDEVTEETQNLNLGVSEEYGLNTKLAMNQLSEKEISFELIEVVVELIEALLNDIVNKGEEGAVLIFLPGWNVIQLLLNFLKSHPVFSNESLFVILPLHSQLTGQEQRRVFERHSPGVRKIILSTNIAETSITIDDVVYVIDSCKVREKMYTSYNNMVHYATVWASRTSIVQRRGRAGRTREGFCFHLCSKSRYEALEEYRTAEMLRIPLHEIALMVKLIGLGSIGDFLAKAIEPPPIDSIIEAEVLLRGWLFQQQDMSALDSNSELTELGRILARLPIEPVLGKTLILATACGIGELLATISAASSFATPYIPRDRTTSKLSFQQRSFSGNRFSDHIALICVYNRWCEAYDQDTIAEKDFCERFSLNSTVLRMIRVAKRQLTDTLISCGFSESLFIPLAISNREPDSNLDLILSLLVYALYPNVCHYRDKRRVYTLEQATALMSKQSVNTPFHSSDIIKFPSPLFVFSEKLRTEIISCKQISNITPLQLLLFGSRKVEYHGNNIIRLDNMISLKMNVQAAARIVALRPCIEALIVRSCLNPETTNKVDENDNKLLKILKQLSSPFGWSPNEKVAGTQQQEYAYVEGISRSSYIKGFRSSANGMKLNTRENRGSANGGRGRTGPTGAFGNNGTRYYSYEGTLETVVRGFDYSPSKSWSQETYVGRPHSRAGYSNINNREGFRNNFGRRGRSNRGLHPSSFFQNKAFHPCMSDSVMRNCGRGTPSDYAEYSRKGSGTIGLAANWDIAPTKRARTGSGAAVPSYLANELVSQSMFGTAVQQCAGNGRERVDPLDAIDEKFKRWWRNVDGSNEYGNF

***Caenorhabditis brenneri* RHA-1**

MSRDIKEFLYAWLGRNKHGNPNYDTRGETRGAKQRFKCELRVPNFPYTAFGNSTNKKDAATNAALDFCQYLVREGAMQQNELPTFTSSSLEVASTWQDTNSSEPATMFVGAEDGSTYQQQQPPQPVKQIRYPWSNAYERDEVTHEQYVTQKADEIAASETVDHKSNFHGGWTMENSKKALNEYLQKMRLPQVSYVTKLKESNAVRTMETSAEIFVPQLNKKIIGKGSGSNKKVSEAGCAMNVVRQMFHLNIMQAYSGPTKKTKVSTLPDIDINLPEELSTRVINYVKSCGIDLPTINESLSSTEAPSSLLTDVKLAQFPNSEASTASNISWAPPLQNWNPWRASNIDEPPLAFMSMDEISHRIIEKEEAKQGPQIEKSKAQRNELPVSQYRDQIVQTVANNRVTLIKGETGCGKSTQVAQFLLESFIGNSKAAHFNAVVSQPRRISAISLAERVANERGEEVGETCGYNVRFDSATPRPYGSIMFCTVGVLLRMMENGLRGISHVIIDEIHERDVDTDFVLIVLRDMINQYKDLRVVLMSATIDTDLFTNFFGSMPDVGPTPVIVMHGRTFPVQAFFLEQILQNLRYMPEEVEQKKKKKGAPPPEEDDGDEEVDDKGRNMNILSDPSINESLRTAMSRISEKDIPYGVIEAVLTDIADRGVEGAVLIFLPGWAEIMTLCNRLLEHSEFGQASKYEVLPLHSQLTSQDQRKVFNHYPGKRKIIISTNIAETSITIDDVVYVIDSCKAKERMYTSNNNMVHFATVWASKTNVTQRRGRAGRVRAGYAFHLCSSTRYEALEEHGTAEMLRIPLHQIALTIKLLRLGSVGDFLGKALEPPPYDMVVESEAVLQAMGALDRNLELTSLGQMLARMPIEPVIAKVLILGTALGSGSVMCDVAAAMSFPTPFVPREKHHSRLNGIQRKFSGNKFSDHVALVAVYQGFREAVQMGASAAEREFCDRNSVSNPILKMTDGARRQLIDVLRNQCSFPEAILYDIGVSVNAPDRELNLMRSLLIMALYPNVAYYTGKRKVLTIEQSSALINKYSVLVPMNNKQEVELPSPLLVFTEKVRTRCISCKGMSVITAIQLLVFGSRKIECIGEGLVRVDDMITIRMDVKTAAALVSLRPCMEALLVRSCENPESLATMNQPDAELRQLLRDISSEDYMSEAGPIKDSLLTDHALVQKPSVPQNRPNNAYSDWASGSSNNSSFQASDSSFLSQGSPNNYAPARGGRMYSSRGNRRGNTYHPYARPFPPPNAGMGYQQFNNSGYGAGGDWNNSPSMRGGYGGGYGRPGGGEGYQGGRGGRGRGGNRGWNASQW

***Caenorhabditis briggsae* RHA-1**

MAMKTTTTPHDPAKELRDRTTEIMYLSAKLNHAKETCVSLDNERQKYREVTRQIKEKDIDPVWVFNGTCFLQTSQANSLKILEQDTKTVEDFRGTVEKVIKMSKDIKEFLYAWLGKNKHPNPVYDTKAETRGGRQQFKCELRVTSFPYVAFGNSSNKKDAATNAARDFCLFLVREGEMKESEIPTLTTKCLETSSTWQDTSASEPATMFCGGEDGNSYQEPQAQPKKPRYPWSNAYQREEGTHEEYVTQKAEEIAASETVDHKSSFHGGWTMENSKKALNEYTQKMRLPQINYVTKIKESNTVKTMETTAQLYVPQLNKGLTGKGNGSNKKVSEAACAMNIVRQMFHLNIMQAYSGPTKKNKVSTLPEIDVNLPEELSTRVIEYVKSSGLELPVVDESVSSSESPSTLLTDAKLAQFPNSEACTGSNISWAPPLQNWNPWRAANIDEPPLAFMSMEQISQRITEKEEAKQTGSLDSVNDQRKGLPVAQFKDQIIQTVANNRVTLIKGETGCGKSTQVAQFLLESFIETNKAAYFNAVVSQPRRISAISLAERVANERGEDVGETCGYNVRFDSATPRPYGSIMFCTVGVLLRMMENGLRGISHVIIDEIHERDVDTDFVLIVLRDMINTYKDLRVVLMSATIDTNLFTNFFGSAPDIGPTPVITMHGRTFPVQAFFLEDILHNLRYMPDELEQRKRKKGPAAPVDDDDGDEEVDDKGRNMNLLNDPSANENLKTAMSRISEKDIPYGVIEAILTDIASRGVDGAVLIFLPGWSEIMTLCNRLQEHEEFGQANKYEVLPLHSQLTSQEQRKVFNHYPGKRKIIISTNIAETSITIDDVVYVIDSCKAKERMYTSNNNMVHFATVWASRTNVIQRRGRAGRVRPGYAFHLCSQMRYNSLEEHGTAEMLRIPLHQIALTIKLLRLGSVGEFLGKALEPPPYDMVVESEAILQAMGALDRNLELTSLGKMLARMPIEPVIGKVLILGTALGLGSVMCDVAAAMSFPTPFVPREKHHSRLSGVQRRFAGTKFSDHVSLVSVFQSYREAAEMGAAAGEREFCERNSLSNPVLKMTEGARRQLIDVLRNQCSFPEDILYDHQVNVMAQDRELNLMRSLLVMALYPNVAYYTGKRNVLTIEQSKALINKYSNLVPMANRQELDFPSPLIVFTEKVRTRCISCKGISVITAIQLLVFGSRKVECIGEGLIRLDDMITIRMDVATAVALANLRPCIEALLVRSCENPETLAVMNGGDAELRQLLRDISSDDFMSQAGPLKDSLLTDTALIQKAPAQQNNQNNSYSDWGSSYSNRYQSYSASAAGGKAKHSFRGNRRGSPYYAQNRPHPPPHSGMGYQQFNNSGYGGAAGGDWPRSPSGAPNSYGSGGFRGRRGGGGGRGRGGGNRGWNASQW

***Caenorhabditis japonica* RHA-1**

MSQDVKQSLYAWLGKNKFGNPNYEVKQDTRGNRTRFKCELRVPGMSYMAFGNSSNKKDAATNAALDFCQYLVREGKMLQSELPVLTSSNLEASSWQDSGATDSGAASFFGNIGEGGGQNQNNPQQHNFSGEPAKPRFPWSNAYQRNEGTHAEYIAQKADEIAASETVDLKSEIHGGWTMDNSKKALNEYLQKMRQPPVVYMTKIRELNTVKTMETTAQIFVSQIRKTLTGKGTGSNKKVAESACAMNLVRQMFHLNIMQAYSGPTKKAAVSTLPELEIKLPDELAQRVSNYVKNLGMELVEVNESALTPDTPATIISDIKLASFPVSEVCSASNISWAPPLQNWNPWRASNIDEPPLAFMSMEEISAKIIEKEQVKQGERLDKIKAQRSELPVFQFRNEIIETVANNRVTMIKGETGCGKSTQVSQFLLEHFIEQGKGAHFNAIVSQPRRISAISLAERVSNERGEEVGETCGYNVRFDGASPRPYGSIMFCTVGVLLRMMENGLRGISHVIIDEIHERDVDKRGLRMFFQTDFVLIVLRDMINEYKDLRVVLMSATIDTNLFTNFFNTAPDIGSTPVITMHGRTFPVQAYFLEQIISNLRYMPDEPEAKKRKKGPSAPPAEDDDGDEEVDDKGRNMNLLAPGANETLRTAMSRISEKDIPYGVIEAILIDVAERGHDGAVLIFLPGWAEIMGLHNRLTEHQEFGQASKYEILPLHSQLTSQDQRKVFQHYPGKRKIIISTNIAETSITIDDVIYVIDSCKAKERMYTSNNNMVHFATVWASKTNVIQRRGRAGRVRPGYAFHLCSQQRFEALEEHSTAEMLRIPLHQIALTIKLLRLGSVGEFLGKALEPPPYDMVVESEAVLQAMGALDRNLELTSLGKMLARMPIEPVIAKVLILGTALGAGSVMCDVAAAMSFPTPFVPREKHHSRLSGIQRRFTGNKFSDHTALISVFQSFQESINMGSSAAEREFLERYSLSGPVLKMTEGARRQLIDVLRNQCCFPEDILYDIPVNTNGPDRELDLMRSLLVMALYPNIAYFTGKRKVLTIDQSSALINKYSVLVPMNNRQEMDLPSPLLVFTEKVRTRCISCKGLSVITALQLLVFGSRKIECIGPGLLRIDDTFTLRMDVPTAIQLVALRPCIEALLVRSCENPESLSVVNPDDAELKQLLRDISSENFMTTAGPLKDSLLTDQAMGVKSTGPPPRRDNYSDWGAESTSPGKSLAGGGGYRGRGGHHHPYRSQGPPNSGMRYQNNNFNNSGGRGGGGGGNWNGGGYNGNGYGGGGGGGGFHRGGGRGGRGRGARSWNASNW

***Caenorhabditis remanei* RHA-1**

MSRDIKEFLYAWLGRNKYGNPAYDTKGETRGARSRFKCELRVPSFNYVAFGNSSNKKDAATNAALDFCQFLVRDGKMQQSEIPSLTSSSLETPTWQDASSSEPGTIFCGGEDGMSMPSNDTQGSQYAQPKKPRYPWSNAYQRDEGTHEEYVTQKADEITASETVDHKSSFHGGWTMENSKKALNEYTQKMKLPQVVYTTKIKEANTVRTMETTAQLYVPQLNKTLIGKGSGSNKKVSESGCAMNVVRQMFHLNIMQAYTGPTKKNKVSTLPDISVSLPEVLSTRVVDYVKSCGLELPIIDEATSSAEAPTTLLTDIKLAQFPISENCTASSISWAPPLQNWNPWRASNIDEPPLAFMTMEQISQRINEKEEAKLGEPLDAINAQRRDLPVAQFRDDIVQTVANNRVTLIKGETGCGKSTQVAQFLLESFIDKKQAAHFNAVVSQPRRISAISLAERVANERGEDVGETCGYNVRFDNATPRPYGSIMFCTVGVLLRMMENGLRGISHVIIDEIHERDVDTDFVLIVLRDMISQFKDLRVVLMSATIDTNLFTNFFGSAPEIGPTPVITMHGRTFPVQGAFISLFNNTVSSFLAFYLEDIIQNLRYMPDEPEQRKKKKGAAPPEDDEGDEEVDDKGRNMNLLTDPSINESLKVAMSRISEKDIPYGVIEATLVDIANRGVDGAVLIFLPGWAEIMSLCNRLLEHQEFGQTSKYEVLPLHSQLTSQEQRKVFNHYPNKRKIIISTNIAETSITIDDVVYVIDSCKAKERMYTSNNNMVHFATVWASKTNVIQRRGRAGRVRAGYAFHLCSRMRFESLDEHGTAEMLRIPLHQIALTIKLLRLGSVGDFLGKALEPPPYDMVVESEAVLQAMGALDRNLELTSLGKMLARMPIEPVIAKVLILGTALGAGSVMCDVAAAMSFPTPFVPREKHHSRLSGVQRKFTGNKFSDHVALVSVFQSYREASQMGNSAAIEREFCERFSVSNPVLKMTEGARRQLVDVLRNQCSFPEDILFDVQVNVNGPDRELNLMRSLLVMALYPNVAYYTGKRKVLTIEQSSALINKYSVLVPMNNRQEMELPSPLLVFTEKVRTRCISCKGMSVITAIQLLVFGSRKIECIGEGLVRVDDMITIRMDVPTAAALVGLRPCIEALLVRSCENPESLGVMNSSDAELRQLLRDISSEDFMSQAGPIRDSLLTDNAIIQMPTAPQNRSNNSYSDWGPTSSNNSSFQADSSYQNIPGSQQSYSPAPGGKMFSSRGYTRGRRPYAQNRPYPPPRSGMGYHHFNNSGYGGAGGDWNSNSSRGAYGGGDSGYGAPGSNDGFRGGRGGRGRGGNRGWNASQW

***Haemonchus contortus* RHA-1**

FFLEDVITMLKYMPNSFRTYVAEATIFVPQLRKSQSAHDKYVTQKAEEIARNLNILSGDVSPDLKRAMANINEKEIPLGVVEVCSLPISSQGRGQGSSKKVAESGCSMGIVRQLFHLGILAEFKGERKKTVATTARDFNFSLPEIPITIPPELAERVKNYVTSSGVEPITVDPNENVTTEAPKSLLTNCKLDQFPDSEVTDFILIVLREMLREYADLRVVLMSATIDTKMFIDFFGGCPIIEMEGRTFPVKRKVLLVLHSESVDLKAEIHGGWTMENSKKALNEFLQKTRQPPIAYNTQLKEANNCR

***Meloidogyne hapla* RHA-1**

MHIGSNAYSHKDLSLFSTELTIKDLAIRLDRFVDTNILVEQSIYGRGQGSTKKIAETTCALSIVRQLFHIGAIPSSSDKLPSNKKLRADNLSDIKVHVDPGLVTRIEDFLESVGLHPVIDGLEETTPDTPKSLLVDKKLEEFPPSEDKYCSTSIMWAPPCQNWNPWKASNIDNQPIAFWSMERISDDLLMKGKSKQVPLQLVQARQRLPVFNHREQIIKAVTEHPVVLIKGSTGCGKSTQICQYLLESHILDKRGANFNCYVTQPRRISAITLAERVANERYEQLGESVGYSVRFEGIIPRQYGAIM

***Meloidogyne incognita* RHA-1**

MHIGSNAYYARLLSPIHVVVEPTALFNCVNHLKILPFWQGRSFVIIFIAVEQNECTFVAENELFLPRLNRTIYGRGQGSTKKIAETTCALSIVRQLFHIGAIPSSSDKLSPTKNRKADNLAEIKVHVDPGLVTRIEKYLGDVGVEPVVDGLEQTTPDAPKSLLVDKKLDEFPPSEDNYSNASIMWAPPCQNWNPWKASNIDNQPIAFWSMDRISDDLMMREKAKQVPQQLVQARQRLPVFNHREQIIRAVDEHPVVLIKGSTGCGKSTQICQYLLESHLLDKRGANFNCYVTQPRRISAITLAERVASERCEQLGESIGYSVRFEGITPRQYGAIMFVTVGVLMRKMEMGLRGISHVIVDEIHERDINTDFLLIVVRDMLRANPKLRVLLMSATIDTSLFTNYFGRCPIIEIEQRVHPVRGFFLEDVISMLKYMPQLPEPDKKKKKKRSGKPEPSTSNADVDEECDEMSMSVTDNLLVCNDDYPPETKQALGRITEREIPIELIEQLLSDIDQNGKPGSVLIFLPGWQMISLLWNRLMLHRIFSNSRRFVILPLHSQLSAREQHRVFEPVGIEQRKIILSTNIAETSVTIDDVVYVIDSCRVREKMYSSRNNMVHFANVWASRTNLIQRRGRAGRVQEGFCFHLVTRARFDALEDHRTAEMLRTPLHEITLTIKLLHLGSVGEFLEKAVQPPPIDAVVLLREMNALDKDLELTDLGRILARLPIEPKLGKMVLLGAAMGVGNLMLTSAAATSFNTPFVPKERMHTKLAHSHRSFSGNRPSDHIGLVNVNQQFSEQFDIDVSSAEDLCRRFSLSYPLLCMTREAKRQLYDVLVNHSGFPESVFTSYSIDVRGPDSNLDVFLSMLVAAYYPNVCYLRDRRRVYTLEQAVALLSNMSVCVPFNRGEQANFASPLFMFSEKLRTNCISCKQVSMISPLQLLLFGSRKVEAIGADKVRLDNMIPLNLSAKMAAKIVALRPCIEALVVRSCMHPEQLSDQPKQDKILTALIRELSSDRAWIPSGEEGLEPVEQSSSSKGSNAHQFNVNEQRSYATPLNQTLNLSSSAPVPLRSLKRPYGSPPSIVDVYGNQPSPLTQPNSLGQEAVAAATGLASSADFGGRRTPYTAHSPSGGDYSSMAGYGPGSYYGNPQNQQNQLPQGLQQFNGMNGRGMRGFGGRGRGHGGGGGGGRRHGGFRRRRGN

***Trichinella spiralis* RHA-1**

MSSIKCSVKSTLERCGVEATFAKNPKGTLLGFFQAMRWKMPSIVTERTGLSREAIWHCRISFLLPESDNLLTFNSCVDSKSKSEAESWAAYSACKFLSEKYDHFPSRKKSITPEKIKADELLESQKWSQLGRHPKSVVHEFYQARGFDKLPLENGKITVSLTAEVPLVFSTVISRNTRKHAVAVAYWEFLLQLKEAKYVDDNFNVIFHSKQEIMEFKRNQRLPTFIDISESLFRRIDCLLFSKDFMQSVQRLRIKQAVDKLINSNGCSVNLNEEEDEDGSVDECLELDMILKRNHSLYTSAQRLRNSFDPAVINLRSFRQQLPIFVIKDELLSTLEHHQVVIIAGDTGCGKTTQIPQFIFDDYVTKFRGAECNIIVTQPRRISAIAMANRLAAERQEAVGETVGFNVRLNSCIPRNKGSILFLTPGTFLRSAMFAEQIENISHVIIDEVHERDVLTDLMLVFLKRKLVILPKLKLILMSASINPYKFSSYFNNCPVLSAAGRIFDVAEYFLDDVYRFLGREMPSEEEIVDHDYETDANLVAELLCWIHKNRPVRSNSFNNFNYPNENGDVLCFLPSWNDICRVSGHLDARKCSEEQMVVLPCHSSLPISEQKRIFEPMSCGKRKIVLATNIAETSLTIDNIRYVVNTGTRKIGRLIASKNWLSHHQSWASKSSQIQRKGRAGRQMEGECYHLFSKEVYKNMPEYDVPTIQAAPLERLALMTKSLFKNSDPFQVLSEALDPPSKEGCKSNLRFTIEILVVAFFPVDAARHLLQDLKIFDKENRLTELGKNVALFGCHPFLGIALLYSIIFRCTEPILSIVACLESDENFFDLSFSNKDQIIKLLDYLIGRSFSLHCSAVNLSRFGCDLMRQHSSETDEWKSNIFSKNFISLRSVVFILELRKQFAEEMVRSNLVDEWKDLFSLDHPLNLSSNNFEIIKAVIMAGLFQNVAKGIHGILSKRGKIEPDAVTICESNLKPVRPRRYGNYILKESDNWWSDWFIYYKKIWSEKSRSFAVEGLSMINPLIAVLFSGFTPEILQSDNATVIALVPAKHFQLQVSSARNAELLIRLRSCIHDFFNLYINHHEYIMKLAENDPIKKWHNDLLTLITDLLNVEQNYNSKQFT

**EKL-6**

***Ancylostoma caninum* EKL-6**

MAVGLAGGVIMGACVDEQVSSALLETCDALMSFSNFASGLKRSDDAILVVAADARKLASLFGKDLPSAAETPPPIKNKEGPGNIVDSIRDRLADDSPVERGGALLDAGRLIRLRNPTILLHLEEWLFKAMKEAIFDCDSYVYLAAINAVAEAACYNSNYLRELISIFKNSESVTESKSEVDVEGEKSAADKDNSVPVDVVVRSRLCEVIGKVFKELGDMSPVWIDECAGVFLACFTEKDEILRASASQSLAELILACRGRNLEKYINEIFLVVEHVISFDDSALVRRAAVNLLRQIIRSCEAKIFEVVGARLRDLHRELLRLWRFDSDHVVRLHAELALEEIKAAIKCTVLEETSNNTRSIIF

***Brugia malayi* EKL-6**

MGQVTIHDYLYLIQMGCTPLSLPKVRRLPFDPLKHQTIHLERLMRSKGLMDLQLDEEYRLPHSENIPCDPDVNETTLFDEHSYSVSPPLAPPPPSPTTDVAETEIDYRIHYSYRMLYLLKKVAEILEEMSKKASNPSILLSINQLSIVETAFEFIIPTAVRPYVDDGVILHLGRSSLVEHWKPLVGDVEFRKKQLRKALEVFDLLMNSCEMIKNCVVRKFLADYICLNEQLIKLGEKYHKQKYGKFILTIDRPMLITSLFILLTTNKEQPKWLHKAVSKHITKLLLLENGLYHLITAITEGANCFGDFGFVQSLSRILVTVPYKMPKEKYYISLIENFLVVIENHPNAGDAAVCFAVVLDMLPKELVEMIFDTLLLPWENLKIDCFSKYSSRKTDFFDPMLDRSLRILSFYAKALPRGMHLPLFRRFKALFNLWTLLYASYSENENRMHGISVEYDNAGQGIFDILHRILDDPSFETVDEKVLIEEVEQKEVSEEENKQMNTSSITSTIYGSHIFWLKLLKAHYANLRIHLVKPGKAEDAFIRFEKGILKILQVEKIQGEEPKASRRLAIRTAMCCIERWAEVDSKNDSYESTCSSEETSDNEEIGFEGRKQKVNYFLNDLLPILSETGTLSDENLANLIDIPLSILDVATVKLNMRVNTIPVDIRGIPSEEDKELEATDRNNIHLALTILQCILVFKQGEFVKFMENLVRGANILQSFSNTVSKLNDIDKRLYLEFQQLAGEIIALLKANNIEPREDDAAPSFIDESPSIGDNPINGRGSESFSLEQIQSDLLDESESVRGHALIKLARGIRRKNRQLLDDITTYPAIMRIVLEQVADNDSYVYLAAINALAELAYWKQQFFDEMVEFFLDPAEKLKSILEFSMDNLREDEKETYLLIQRVKIGEAIAKANKNLGEMAPAYFDRMVNPMLSLMLHTKDDMLRASVLSSLSNLIVSCRGLNIHKHLDEMLLAAKLYIRDAEAEIVRRAAVDLMRAIVRTYDISLLQEAPSHLYDIADSLSYILDQDEDLVVKTHAMLCLQDIDAQMEEGFLEFEKAHTRKIRF

***Caenorhabditis brenneri* EKL-6**

MKKNCFRICIVYKNVLYFTAETWKNDPFEVLVKQCPKVVSDKYLTPHSVDSIPSTSDDVRTVNADARVHYSQLLGSLYLELASTLSTLRTSSPDEYQLISLQDVSVIKKSFEFFLLTGILPFLEPGVGLPASTRSTFIKSWKLYDGNKESCIERLDFAAKVIVAMLESNEAIAVQFLPKFIYDILAVRYQLLELKVDKYELQLEDIISKCPMDVLFGGLMFLTQDRKSVKTPMWLKISCGKQMTKILVDKDGLSYLLQYYRERAGDTWTDNLPLTKQVAWHLATVPKMFKHPLQYHEIISNQFFELIWSQKVLDKTTVTVFTNYVDELRTRFWLNADLTVFDKILNFWEILGKKLQDRTLTTSEKIETFSPNYVRNLQLLSQLQNTSDTKRLRALIICFIACIEQIPYIKDILKGALDGVGSLGYTIYYYVITPSLVVQLHNRSKTTSMIEEVGERNVDESPVDELWVYGFDNPDDGVARRLDTAFYVVDNVLSSAQTRTLMEMMNAALEDFLKVSEKERDDDFARFVQLDGSKHFSSSHAHLVVGCCYERLISIAGDHGFSQEECIQLIKISESILNNATAKFLRIVARKRAVDVFQLSAAEKKEFEHTRDTARMCLPIISTIFFITQGTPRMQDVHLKSMEAMANFTKAADLLPSEDPTFNSAVDEAKGLLRKLKIDVNQVSAPVVPQRNERRRYNQTDICNEWIDELHDDEPAVKGGALILIAKAFRAKSWHCQKLLDYAAFDTVKDMVSDTDSYVYLSAINCLCEMALFDRHVFDGFIEYYEEIASIPNKDERLIIRVGRTSEALGKLLIARGETSIAYFDRLATVFMSGINEKDELSRASSCGAFGNLLMATGGKGVAKWMDQLLQTITNVLRTDRSPLVRRSATDLIRHSLHSVGRDMFVVLRERLLDIHREVRQLWRTDRDETVRLHAQLCVEEIAAALRQNQEDVDREYQRKLRL

***Caenorhabditis briggsae* EKL-6**

MTVVCKLISSTKPSGEETCRETATTAAQPAAASPGRVVKGRILIKTSQRRSPRTRANLRSYLAMVLLISLTVDVYALATTNPDCPDLFSQLTGLHPLANITMVMLLHLICLFSGLSRTVPGCRLAWFFFMVSLVLMMLVPAFIGSYMASGLAPEMRWEKFEANNNITFVSELTAAQFRFQTAFYMAVEGQLFVFILFISETWKSDPFEILLKQCPKEVFDKFVDPTIEDPTPSTSDGAKTVNADARAHYSRILGGLYLELAGVLESQRATTDDEYQLISLQEIAIIKDSFQFFLLTGVIPFLEPGVCLPASARSTFIKSWKVYDGNKETCAVKVRSATVYFEKENNFQLDFAAKVIVALLKCNDAIASQFMKKFIDDVIAIQYQLNELKVNNYEAQLEEIISKCPVDLLFGSLMFLSRGGKSAEPPMWLKGACGRTLSKILVAEGGLTLMLQYYQERAGENWTDHLPMTKSVAWCLAAVPKIFSHPRKYHENISNQFFEIIWSQKEVDKNTLNLFVSYVNEVHVRFSLNADLTVFDKILNFWELLDKKLQNKEQKHTEKIEGFSTNDIRNLQLLSQMQNSYNVKRIRQLTTCFFACAEQIPYIKDILRAILDSVGSLGYTIYQYVITPSLAVRLVKKSVTSSKIQEVGETNGNETPSEELWVDGFSDSDESVGHRIDTAFYVIDNVLTAARVRTIMEMINVALEEFLKVSEKEREDDMARFVQLDGAKLFSSTHSHFIVGCCYERLIAIAGERGFAQDECIQLLRMTENILNNATLKFVRMANRKQSVDVFQMTETEVKEFDSTRSTVRLCIPLITAIFSLTRGASRMQEIATKALEAIANFTKASDLFPSSDVVFNNTIDETKRYLKTLKIDVNDIVPPVVPQRNERRRYNQIDLCNEWIEELHDDEPAVKGGALMQISKAFRQRTWHCQKLIEYGAYDTVKDMVIDDDSYVFLSAINCLCEMALYDRHFFEGLIEYYEELINATNKDEKLVIRIGRTAEAIGKLLISRGESSITFFDRLATAFMTGIEASDEILRASSCGAFGNLLVATRGRGVEKWLDQMLLKVTNILRIDRSPLVRRSATDLIRHSLKAAGKDILVILRESLLDLHREVRNLWKTDRDETVRLHAQLCCEEIDAALRTNQEESERGYHRKIRF

***Caenorhabditis japonica* EKL-6**

MITKDDYIQVINQLITQPKETWKNDPFEELVKNCPKDIVEKYVTTLPEDVTPSTSEDVRTVNFDARVQYSQILGGFLVLILILKMKNHENDEFQLSSYLKSQRDKSSDEYHLVSLQDVAIIRNSFQFFLLAGIIPFLEPGVGLGAAARSTFIKSWKVYDGNKEACREKLIFAAKVIQALLSCNDAITVQFLPKFIDDVICVRFQLLELGVDEYDSEIENILEKCPIDIYFGSLMFLTQDKKGKLTPKWFKAACGRQMTKILVAKDGLSHFLQYYRERAGETWTDNLPMTKQVAWHLATVPKMFKHPLQYHEIISKQFFETIWSQVLIFLLILVVLILNFQNVVDKNLLAVFAGYVEELRIRFSLNCDLTVFDQILRFWEILNKKIIEEKSPNSSRVIDFAPNHVRNLQLLSQILPSADSKRIRACFTCFFACVEEIPHIKDILKASFETVGNLGYSLYQYVMTPSQPVAIVKKRAETSSKIQEIGEKEEEPFDEMWLHGIGDADQSVAMRLEKVLFIVDNILSQSLNRTILEMMSFGLEEFLKTSEKEREEDTARFVQLDSAKMFSSFHAHLLVGCCFERLINIAEKTGFSPEECLQLLQISIQILNNASAKFNRLTVRRRSVDVFSLTEEEKKEIEATRATAKMCFPIVSTILLLTQGSRGLHDYHVKAVEAMANFIKAADTFPTDDHSFNSTVEEARNLLKDLKIDISQISAPPVPSRGDKRRYSQVDICQELMEELHDDEPAVKGGTLMQIAKVFRQRSVICNRLIEYGGFEVAKELVIDDDSYVYLAAINCLCEMALFDRRLFDQLIKYYKELSSFEDKNEQLVIRLGRIAEAIGKLFAVRGEITIEIFDKMATLFMNGIQDSHEIVRASSCGAFGNMLNATRGHGIEKWTDQLLHLLTNILKVDRSTLVRRSAIDLVRQALKACGTNVFVVSFCNFL

***Caenorhabditis remanei* EKL-6**

MKKNCFRICIVYKNVLYFTAETWKNDPFEVLVKQCPKVVSDKYLTPHSVDSIPSTSDDVRTVNADARVHYSQLLGSLYLELASTLSTLRTSSPDEYQLISLQDVSVIKKSFEFFLLTGILPFLEPGVGLPASTRSTFIKSWKLYDGNKESCIERLDFAAKVIVAMLESNEAIAVQFLPKFIYDILAVRYQLLELKVDKYELQLEDIISKCPMDVLFGGLMFLTQDRKSVKTPMWLKISCGKQMTKILVDKDGLSYLLQYYRERAGDTWTDNLPLTKQVAWHLATVPKMFKHPLQYHEIISNQFFELIWSQKVLDKTTVTVFTNYVDELRTRFWLNADLTVFDKILNFWEILGKKLQDRTLTTSEKIETFSPNYVRNLQLLSQLQNTSDTKRLRALIICFACIEQIPYIKDILKGALDGVGSLGYTIYYYVITPSLVVQLHNRSKTTSMIEEVGERNVDESPVDELWVYGFDNPDDGVARRLDTAFYVVDNVLSSAQTRTLMEMMNAALEDFLKVSEKERDDDFARFVQLDGSKHFSSSHAHLVVGCCYERLISIAGDHGFSQEECIQLIKISESILNNATAKFLRIVARKRAVDVFQLSAAEKKEFEHTRDTARMCLPIISTIFFITQGTPRMQDVHLKSMEAMANFTKAADLLPSEDPTFNSAVDEAKGLLRKLKIDVNQVSAPVVPQRNERRRYNQTDICNEWIDELHDDEPAVKGGALILIAKAFRAKSWHCQKLLDYAAFDTVKDMVSDTDSYVYLSAINCLCEMALFDRHVFDGFIEYYEEIASIPNKDERLIIRVGRTSEALGKLLIARGETSIAYFDRLATVFMSGINEKDELSRASSCGAFGNLLMATGGKGVAKWMDQLLQTITNVLRTDRSPLVRRSATDLIRHSLHSVGRDMFVVLRERLLDIHREVRQLWRTDRDETVRLHAQLCVEEIAAALRQNQEDVDREYQRKLRL

***Haemonchus contortus* EKL-6**

FRRSSSHLIAGVFFEQLQDGDINFSDTEIILSLLGLVQWFQIFYAVEKMLIADPSSLVRRAAVHLLRQVIKSCDTALIEVVNVISEIREGLIDESPVTRGGALLQAGRSIRARNVDILMELEKWLFKALKGVFIKFSENSLFLSNCSISDSIVDPDSYVYLAAINALAEAACYSSTYLRELITLFKTFRGSAESPCKEVGDLGPVWMDESAGVFLSCFSEKDEIIRTSALSSLAELILACRGKNIEKYLEEVLKIVGDRLRDLHRELVRLWRWDSDHVVRLHAELALEELRVIMR

***Pristionchus pacificus* EKL-6**

MGEISVLSVMSVLKKAMEIDGSFDIANVRPSKTNDVKRELIKRGLGLALTEMEKHPLLMDQYDRQILKTASSSSGESIWESDARIKFGNLIIFLFANLTKAIEREKARSIIVLHVDTWWKTKEWEGIQEDKPTQAKKLHSYLKTILDLSDVSFQVKGTIVSRELFTVVMAAELLHHLGHTALHARYRSFLSSIDAAVVLNVASAFIRPKANQTNTQWLNKSMIGLVNDMVGKGHLRDLLRAFDSRGQLFKGNMNTMSLPFLLDLVKALCSGTAFSMSKQEYFESLVNQFFVWLDLVFLFCGVSNALAEYRTHHKDDSNEIVDFQDIMNDLVRTVFEFLPKKELFILWGARQPELLPRFEVKPNTKFVNSKIKVIGEDNPIRCPVFVTAHKSSVEDHHLFLENTVTTMIDLVKVEEKADFIFTVLLEVFKAYIKDDAYFNRLSATHDESEFFYLGQLNVLALILNEKLDENTTTLAKLLNLTIITMTSTVNNIEKEVVKQKKEADLDIRGGMNEFEKEVREDMISITMSTATYGIKFTKFIPLLRMIEGDKMKELDTEANEVLRLTAVLKKLLREMKEVPAEYKEIVKAFENDGDLNSTIADMEGSIKTKEDNDASFFNVEELLSLLNGLVYEKGQGLLTLSQLLLKRNFRLIQIYDDTFHDIVLGLISDSDSYVYLSAINCAAEAALAMTDKIIPFVKLFSQINSVREKKNGQGEVVKRETIQEKVIVRLRIASVICKVFDNIGAAAPKYTRETIDCAMSCVDDMDEDIRASAYAIIEYLFRTMAARYTIDNTFQPILDSINHTLTCEKSCQVRRNAIKILTNMLSGAGRDMLTTLSEQLLPINRMLTSVLQSEKDEVVILFAQLAKEEIGESLKEKMIMETTQRIRQIKM

**ZFP-1**

***Ascaris suum* ZFP-1**

MARREKRQFLREKWKGGNKEDVEMLGGCCVCADENGWEANPLVYCDGPNCEVAVHQGIFMRNFAIIDAFF

***Brugia malayi* ZFP-1**

MPHSDRGYVDLSCYGIVEVPEGEWYCAKCADFIAHSQYNGNNGDVGEVREIPRCKLCPFGHGALKRTDNDEWAHVICALYIPEVRFGDVHSMDPVILSDVPLERFQQQCYLCMERGEEKRAYLGACMPCNKPGCKKCFHVTCAQAEGLLCEEGGGSKNVKYCGYCAAHAKKARLFAGGNKEEEVSEGKKSFEGTCIESNCHLSLVGFGSI

***Caenorhabditis brenneri* ZFP-1**

MKEMVGGCCVCADDNGWTDNPLIYCDGDNCEVAVHQGCYGIQEVPEGNWYCAKCTKAATMPAGTKNEETFCCSLCPFSYGALKNTDQNGWAHVICALYIPEVRFGNVHSMEPVILSDIPVEKFQKICYLCQEDRPNDAKKGACMSCNKNNCKRSFHVTCAQRKGLLCEEGAISRNVKYCGYCDNHLKKAINDPAIKVIPACPPVQRLAAKEENAKKNKNNSTPSTVLLTPPPPPPPVPKVTPMLADPIRPTPVNNTLGLGGSSSSASSTPTPPSVAAVVNSIGSVKLDDRPPEKLFAPAFSPPLTTSSRSSVALEPTPPLPSTKITSGPLIPQSAGSSTTSSSSGIMANGSTSQETTVGNHCLQQLQIQSAAASSLSSNNANSSVNDLNGYPGPSQLSSFMHEIPARNTTSVASLLPPGAAEFHLNGSGDEEKTVKAVLTAPLTKAKRMRDAKGDLVDKSVKRPRANARPPAVLGSSSNSSGGTVGKSASMQRLISLVQPVVSEVVTDFQRDRVADRNAAAAERRAAVAQSQPSTSANGTGTNSAPNPPADTILPNITPPVNSTPNGVANTSATSLPGTSTCPEPATNTTIPTPAAPTVNGTATPASSSRLTLPTFMESLLERQWDQGSSLLMANANFDVAQLLSCLFQLKSENLRLEDNLTTLRKRRDHLYTLNSRLNEVNTMDVNESKKRGSLLHQELVLPKQEVPKIEPGTVPANHSSQLFEDVKPPKPSKKNAAHAPAANTLPLTTAAALTTTTTAALVNASTPIQNNRATPSTTGAPLAATPVMTAVTAANELAGLSPDRVSNGAHALLNIYRNMPQMDPSVAAQLSMMGQFQGAVNPTQALFSRILAAQPAMAGLASMMNGGLHAPSQPLPPLPPTSATPNGK

***Caenorhabditis briggsae* ZFP-1**

MTKEMVGGCCVCADDNGWVDNPLIYCDGENCEVAVHQGCYGIQEVPEGEWFCAKCTKAAGMLPGSINDETFCCQLCPFDHGALKRTDRKDGWAHVICALYIPEVRFGNVHSMEPVILSDVPLDKFQKVCYICKEADRPNDAKKGACMTCNIHKCKKSFHVTCAQRQGLLCEEGAVSRNVKYCGYCEPHLAIAVSLLLLHFYPPPGPYRRPLLLRESRETTIRWGEEKGVEKRDGWSHTYYNKNSGNYKKPFFVKKCQDDPAIKIIPACPPILQKLKAPEKHKKNAPTPTVLLTPPPPLPPVPKVTPMLADPLPIRPPPNQVNGISSTVEERTSGIFAPPTTFSPPLTTSSRSSVAPDPIVEKPKNSFSSGPLIPSTVQSTATTSSEKAAMANGSTLPSSSAETTVGSHCLQQLHIQSAAAAAGIAPQNDLNGYPGPRELSSFMHEIPARNTTSVASLLPPNAADFHLNGSGDEEKTVKAVLTAPLAKAKRMRDSKNDHLVDKTNKRPRANGGTVGKSASMQRLMNLVQPVVSEVVTDFQRDRVADRTAAERRAAVAQSQPSTSTNGVVTAPPVPNPVEPMLNLTIPPPTHQSNGVAPPNIPPSQIPGPSTSNEAPVNGVSPSQLPHRLNTTPSFMEQLLERQWDQGSTLFISNASFDVAQLMSCLFQLKSENNRLEENLSVLRKRRDHLFALNARLAEVNTLDLARKREASLPDLPLVPKTEIHKQEHVPTSVPLPANHSSVLFEDAKPPKSHKKATVHQTPIAHPVPLTTSAVLPSSTPSAMSSANIQSARATPSTAGVPLAANPIMTAVTAANELAALSPDRVANHTQAILSMSRFMQPGMDANVAAQLQMLSTLQQGMNQSNLFSRMLTNPTMAGLASMLNGGLQQNAVVAQQQQHPLPTIPSTSATPNGK

***Caenorhabditis japonica* ZFP-1**

TMFNMKVRFNRTCYLCNEERPVDAKKGACMSCNKSTCKRSFHVTCAQRKGLLCEEGAISRNVKYCGYCEGHLKKAINDPAIKVIPACPPVQRLPKEEKKKAKVLSSPPPPPPLPPPPKLHMLVDTLPRPNQVNSVLSIGSTINANSNSNANSTPLDERPPPVSSNSSIYAPPPTAFSPPLTISSRSSVALDQTPPLVPKSSISSGPLIPTAAPFAPTASSSSSSPYPSMANGSSLPSSEPTVGSHCLQQLHSATPVFSVLSTVPSQASNTVLNDLNGYPAASQLSSFMVSSKFSIKPTVEKANFQHEIPARNTTSVASLLPPGAAEYHLNGSGDEEKTVKAVLTAPLAKAKRMRDSKNDIMDKSNKRPRAHPRAPAVLGSSSTSSSAGGTVGKSASMQRLINLVAPVVSETVTDFQRDRVAAAERRAAAAQSQPSTSTNGSMNTPNPPSEVFANSNSASHTSNLLSAGLLSMPGTSNETALNGSASSSHSSRLQLPTFMEQLLERQWDHGSNLLMANAHFDVAQLFSCLFQLKSENFRLEENLSQLRKRRDHLYTLNSKLAEVNVLDVPKRQKEFVQQNQDAASSSSSSSACALAKEPKVESMPPTAIPLPANHNISAGGVHNNTMSEDVKPSLKAASANRKSAPLSMPTPVPYTNAPVSMTASSTPSLATIQNNR

***Caenorhabditis remanei* ZFP-1**

MKEMVGGCCVCADENGWTDNPLIYCDGENCEVAVHQGCYGIQEVPEGEWFCAKCTKAASMMRGSINEETFCCQLCPFDYGALKRTDRKDGWAHVICALYIPEVRFGNVHSMEPVILSDVPIEKFQKICYICNEERPNDAKKGACMSCNKSTCKRSFHVTCAQRKGLLCEEGAISRNVKYCGYCENHLKKAINDPAIKVIPACPPVQRFVKEQEKHKKTAAATSTTVLLTPPPPPPPVPKVNHMLADPLPIRPPTNQVNSVLGLGSAINAVSLDERPSSTSASEKNSSSGIFAPPPTAFSPPLTTSSRSSVAQNPTPPLPTKNSTSSSGPLISSTAQQSATTSSEKGNMANGSTLPSSETTVGNHCLQQLQIQSAAAAASSHSSSVGDLNGYPAASQLSSFMVSKFTTFLFKFLETFVFYFKFPLFQHEIPARNTTSVASLLPPGAAEYHLNGSGDEEKTVKAVLTAPLAKAKRMRDSKNDMLDKTNKRPRANGRPPAVLGSSSSSSGGTVGKSASMQRLMNLVQPVVSEVVTDFQRDRVADRTAAERRAAVAQSQPSTSTNGGIGMAPSAPNPTVETGHLNSTNPTNLVNGGLSNAPSISIPGTSTESAPVNGTATPHNARQNLPSFMEQLLERQWDQGSTLLMANAHFDVAQLLSCLFQLKSENIRLEENLTSLRKRRDHLFALNSRLAEVNTLDVTKRQRDGILQHPDLSTPKLEVHKQEPTSSTPLPANHSSVLFEDVKPTKTNSHHRKSAGHTPAATLPAPVPLTTAAALTTTTATTLGNSTIQNHRATPSAAGTPLPAQPLLTTNEMAALSPDRQNQANQQAAMLMNIYRTLNPMDASVAAQLQMINSFQNVNPTTALFSRILAAQPGMNLGNVMNGGIQQPQQSLPTLPPTSATPNGK

***Haemonchus contortus* ZFP-1**

MKEMLGGCCVCADENGWTDNPLIYCDGPGCEVAVHQGMKEMLGGCCVCADENGWTDNPLIYCDGPGCEVAVHQGRGFHVTCAQQRGLLCEEGGGSKNVKYCGYCEHHLRKAVRGFHVTCAQQRGLLCEEGGGSKNVKYCGYCEHHLRKAVGWAHVICALYIPEVRFGDVHSMDPVILSDVPMERGWAHVICALYIPEVRFGDVHSMDPVILSDVPMERGCYGIQEVPEGEWLCAKCHVAATTYSNGELSRNGSSNGVGHDMIKARCELCPFSYGALKRTEQKGTSSCIGCYGIQEVPEGEWLCAKCHVAATTYSNGELSRNGSSNGVGHDMIKARCELCPFSYGALKRTEQKGTSSCIRFEKLCYICANAGDSRAAQMGACMSCNKPGCKRFEKLCYICANAGDSRAAQMGACMSCNKPGCK

***Meloidogyne hapla* ZFP-1**

MLTVLNNSDYQQITINYTDQTVHLDPECSFDVTWSSGGGQFTNNSHKSTQQNQTDNIPKPHYKTLAKNELTIQQTASMPKSYVKYIDCEGENKANEIEYDLDREDLEWLKLINEERKEKGYNSIDDIVLEKAINLLEKESYFQYVKTGQLNASNLPLTDDDAPCCICNDAEDSNANQIIFCDMCNVAVHQECYGVPYIPEGQWLCRRCQLSPSVVVKCVLCPFTTGAFKQTADGRWVHVICAIWINEVHFANTVFLEPVEGVDFALKRRKKLTCIVCRNRMGACLQCSHRSCARSFHVTCAFYAHVRMELKEMRTKKNPDTIVNRFVYCHQHSHNGPNTEETAAAWKRDIQCKIAQARKRLHLEAKKQNLSAPIPVIPHNKIAQLADDLSIENFSDITAYWALKRQSRCGVPLIRRLQVYLGQQKPIEGRLTATDRLGISPTRGEHHIELQQGDTSSQPLMKMALLRREFERVRLLSELVKKREKLKKESLSCDEKLLSMITPVAILIKRTLEKLISKDHQDVFMHPVSEEDVPGYRKFIAHPMDFCQMQKKLEMGQYKRVADIRSDFLLMMNNCATFNRDNKYFYDYGQRIRQIGLKVIRAAEIEESKISQTLQMVETFISRFEFLSVDYINKLQSELKLKDEIIADDVSEHGARKNVEKQPKKSNITINKTEDNNNANNTSISSIPASNDKKKLQTSRKRINNVKSLNSSIDESKNKIKEQKQRRNSKKIKKDNSDESLIILEEKPSTSTENLNNNEDTEKQNLSTPRRLSTSRKCKLPTVSTPGPSEQSKITRKRVRTDINNKTSNKSANSSLSDTAKLFYRQISTPANSTTVFDRDKITKSSNNSTKPTLDDCEQNGTSTEKTSTVKNCHNWIGRNNKNKNFTYSYLTEASSAADEEDSFNEDDEDLLPPINLPNGIKQQQNNKSSKRLRKEIIQNNSTDEQFR

***Pristionchus pacificus* ZFP-1**

MYPVQYILLNAVRARRLDGHEKRRSKERKRFNIIEPTHRFARDSRPLQAAKIRSKTSSTLVHPLNGGEKKDRIHSLVSYSLSPSPSPVGSPFPLAAAAATQPAAAAAAAAAAAALLIAKQPLPFPSIASTLTSVSTSSSAAMKEMLGGCCVCSDENGWNGNPLVYCDGPDCEVAVHQGCYGILEVPEGEWLCAKCQIASANGTSKANGVDHRNGNSLASPITHPRCDLCPFGYGALKRTNKNSWAHVICALYIPEVKFGDVHSMDPVILDDVPQDRRAFGEGYDSIVHVICARTRALLAVPRSAPA

**MUT-2**

***Caenorhabditis brenneri* MUT-2**

MNNKEVENANSYKVHPNTERILHFRRTCAEIVHSNKLSFDCLSIDMVHEFKRVMQDQAELDRKIAFCEDLQSTIQRINPTWNFRIVPTGSSVTGLATRNSDLDVAIHIPQVARIVEEMCSGRLVTAEEKLVMWRECDDRFAPLCFVVKKWADSTGVKNPKDGGFNSYALVLLVIHFLQCGTSPPILPNLIKIYKGMNFIAQSEHDFPERLDLEAPFPKPLPTFSPNDASIAHLFFEFLNYYSGFKFDENYISIKDACVYSRSLIFVGNCSVCELFCFRKSSILPEAVRNQKQKQVYIEDPFDSHNPGRTVRSIRTLKKIFKMTIKKFNPNACSQDDNSSTNSDSNFKFPTLSDILNMSLSNQEEEDEEAQKEDENEEEEEEVQNVSVNGCNEFVEWR

***Caenorhabditis briggsae* MUT-2**

MLLRQDDQDQIAYHVTSNLTSYHGFNERFKKILEAFNDEFQLLSCNMFDYFDDSKQSTEEFDRKMDLCYQLKNIISKHNSTWLFNIVPTGSTVTGLATKNSDLDVAIHIPQAAKLLEEMHSDIYHIEEERNRLWRGMQLEILQIVRLLLENDEQIKSRIDWNKGVQLVQAQIQILKIETVDGIDCDVSVVMDPFLSSMHNSFMIRHFANIDARFAPLCAVVKQWAASSGVKNPKEGGFNSYALVILVIHFLQCGAYPPILPHLSKLYKDDNFIAQNDRQYPLRLDFGAPLPRALPTVSANHSSLAQLFLEFLHYYLKFDFHMYFISMRDAMIKNRQQSMHPTVKNEIQKEVHIEDPFDAHNPGRTVRSLHHIKKVLKDTINLFIPVYVPNEDIDTQKKNFHFPTLDDILFMATSNSHLEAFEEHAQDDETQNEGPSTSGVPPNVIS

***Caenorhabditis japonica* MUT-2**

MQRCNEHENVREFNQISYKVHSNVTPFIEFNENYREVVNQYEEQFQKLSLDMENFFYRRKQPEEEFQRKMDFCSQLKRTIQKYCPSWIFDIVPTGSSVSGLATSNSDLDVAIHIPQAIKVMHCFTDRIVFFRKIQLEILQIVRRIVVNDEEFKKRINFDKGVQLVQAQIQILKIETVDGIECDISVVLETFLSSMHNSLLIRHFHHIDSRFGPLCAIVKQWAASTEVKKPKEGGFNSYALVLLVIHFLQCGTLPPILPHIQEIYEGKNVIAQDEHRFPKKLDFGAPLPGKIPEIEINPAPVALLFFQFVHYYLHFDFQKYYISMRKAMVINRNRSHSSEVRIQDKKEVYIQDPFDSHNPGRTVNSLKNIKITLRDTVSKFLPNGNNFKFPTLDDILYMDSSKAKFDNEEDRDDAPEEEEKQNL

***Caenorhabditis remanei* MUT-2**

MTSNRDAYQIAYEVCSNLGSFHKFNERFSPVLDVFEDEFHVLSCNMFDYFDTSKQPEEEFNRKMNWCYQLKNIISKHNPTWLFNIVPTGSTVTGLATKNSDLDVAIHIPQAARLLDELYPQIALSEEERFCKWRGMQLEILQTVRLILEKDEQIKPLVNWEKGIHLVQAQIQILQIETADGIECDISVVMEPFLSSMHNSFMIRHYVHIDHRFATLCAVVKKWAASTGVKNPKDGGFNSYALVILVIHFLQCGAYPPILPNLSKLYKDDNFIATNDKKYPELLDFGAPLPRDLPKIQMNQASTAQLFLEFVHYYFEFDFQETYISMRDSIVKSRTRCPNETVKNEKQKDVYIEDPFDAHNPGRTVRSLTNIKRILRETLNMFIPPIEPTGNLQNQKRNFLFPRLDDIINMPSSSHFSPPPPVPEEEESSDVPSTSGIPPVSFRR

***Haemonchus contortus* MUT-2**

SYAMVLLVVHFLQCAVFPPVLPNLQELFPRRKIIDKRFAPLCMLVKDWASRCDVKNPKHGGFNRFVFIRDPIDDHNPGRTVRDVEYLQHIMRSLAELFIRFLDYYSRFDFGHHYICMRDAAVVPVLKIKWKKGLETDLSCSTEMFVSGIQNSYLIRGFA

**EKL-5**

***Caenorhabditis brenneri* EKL-5**

MLNAENLRGQEAVPAHEAARDRYRRAVEYRLNQNRMAVQFNSPSRCPFCQAPLRYDRFLAHVEVCPEAANDPQKKLLAIHEFNSGPHVFHRELQKLREACEVSFLEAKLDPGKLANMACRLCKSIDEHLCGGCLSDQQKSVFDLESNKIAARVLVHFEQYLNLKMQIAVKNLTQEHEQYFDSIGGLLNGELADMKAWSKIEETTRKHHIEKTKLINDLFAENQKQYESFQAEVKRTVERSARDMRAHLSSNQRNVHNLHRVIAIRRRVQAVEEVEQDQPNVMGEEEAAQLAAMLANIKIPEI

***Caenorhabditis briggsae* EKL-5**

MFSLPLGQSSEIVCDLRRPAEHSKAIEKRESIERDAAIPNRNNNAPDPPLIEDQDQEDQREVVDHSAMEPENVMRVVRNHDHRHEQLEAAREQHEAAARRLGQAIVDNGGEEASMRQREAARAMRQAIVVNDDEDGRIRRQQRQIPPRRRARDEAEARMRLREAARAMRQAIVANDDADDRIRRQQIPIPPRRRVPDRREMPIQQAIHRAQYAIEAQQEQQKKTECSFCNTLILNTEFVEHLTPCATEHGIVGTQKNLPLYNFLSNSWTLDQEFYKMRQGLEIEYLEACRDPSKISDMHCIKCPTWRDHNAGGCMSNLQKAAFLHHTDRILNLYMAHYEISLELKKDRGVEEINAKHIADFGSVEEYLNSNPVNLESEHSREGVRHETLEKQNVERDKFIQEEMDKNRAAYARMKEMVIRYALRKCSEMVVYLGQNKDRSDIGQVLAYRKCLRDEGYEAASRLERAEQGEVDDWFGGWRGERNEDVRNAGPDPEDYPWMENDEFVNANEELRRGQVLLDMWNNANVVEDDDPIIEEDDDAVMAEAPLNQDQPI

***Caenorhabditis japonica* EKL-5**

MKTQVCIHRYQTMVIHKFLSSKFVTKIEIHDCVIRHEIAYYKVWGIPEESPQQPLKALLDLDCVKCATFADHMAGKCLNDNMESVLLKELMTIADSIMAHFEAYLDLKKVIGLQKIEDKHNAHFNEIESCFNDDSTDDETESLRERMWYDFRKTTIDEQMNEKRNYLHETSIEHKEMMSRQKECLRSDMSIIASKLSSTILANRRPENLPRVRQLVREDYVSHGIDAPPEFAARFDDKFTMRLGSATHDELDFAAMEHRRLRDAMAWDGIVNRVERQVLARRRRHLIAQVAEDNARVDGAL

***Caenorhabditis remanei* EKL-5**

MANRREHNQMLDLERIELMDEFNEVMMNEIRNPARNDRGEHHQMLELERFEFMNAFHGRMMNADRDPARIRIQELLNRRANHHGRNGHGFVLRFENGPMDLFDLEFDNHELHICNICLAQIPNIEYLKHLDDCAEKHHSLEHVKNAAYYHFTSSDYYVHQEFAKLMENLEIRYLSAVVDESFVKWMPCMSCDTLMDHLSEKCMDNNMEEIFTFEGKTLVNRSLNNYKLYLEYNMQVRLDGIVSRHSKYLDSIEHILDMEPTDMESQLLKNRTWDNFVNVTLPAQHVEIFDFKEKTMLENEEMLEKEENSLDSLFLKNKNRMVAYIETFKKKENLREILQQRKSLRLDPIDMFIPSNLMIGPYQEGVVEEEYPYVANVQAADEEMSGL

**MES-3**

***Caenorhabditis brenneri* MES-3**

MSPDPTAPTTTNTNTTTDAGRKRRNEVEPPSAKRSPRSIKEEKENEMNQQPTTSSANSSTSNLLVTVKEEPMESEEINTTNTTRNCRKNGRPRRLEDVQKTLKTSSDPNELHEYYGRNKTVASSNVRFLVRNVVQKKTLFEGKAICRERVMPKIQAEADRLLERKARMYDHHHLATLDFTKVNSWGNSMDASNFDTSACSVQAFAVLLDSQNRVKLKKLGMAVISLTQKRLNEEDGNQFNTKKIHLPAHEFVGARKIYIAVIVKRMVVKEEKRGATRKAAAGASSANAKTTPQVLEQKMRIGALLIYDSCGRNLLNNGIERIILVDSTEKPEFEAIFTEMLSSPTEWMKATHNLEILAKQSLRQEDQHLPRLLFTSEATRFSQIYNSLLDRAAESQVKFEAVLEYRRLYPNHKEARKDDRWLSSRFPCYEFHLDEETGECRPVQNVDYYKSDTVETHENTCMFRPVTERSKGRRHRPIVRHVRHDPVEPMDVDDGIGMDIDYDVEMDHPHEAQRYDRYSPQPPVNIGPDGKRLSGLQFKFPKGHSSYAPCTFGNRAVDQVPHLDEQQPKTYRKEEFIRHNPRVYTEFQMREKRIDDLPAPEFRKRNQEMYVVQVPATQQQIDIYNKELEKVKITRKVNPDLKHSNDGHPIPPGCAIDDEVHIHFDSQIYGSRNPPKFEKQAKFISEYCVTYERKLQLGRMRAKQLHEKHFFSKPGSISFDLNHGFETFDPKEGDDKLTMLLEWIRQMSPEGGRLRKVIHEADFVCWRGLLTKISATIYQKEDGWRVKALRRKGVIFLMEEKTEQARQRELNQTDMEKRMSYWGHKFEQYVTRDDDKEEPDTTIPVTTKEEYGVVFRNDLFTDPRLHPSRRTIGILYSGEVDCLDRHGNMVELKTQKGELHGGFWKQSKSLKWWLQSSLVNVDNIVVGHRTQEGQVTALSTLKAREMPQRASWNFRACFDFTSTILTSIFNYLEKDGEACLVEYNNDLGLHKGIQMKRISAEECDFVPDSFLEKYH

***Caenorhabditis briggsae* MES-3**

MPATRKRQNDGNTPAPKRVRRSRKAKEEEKENIIRTSVNSTPDASIAGSSRVSPIASTNRRKTKPVNLCNLEKTLTNFNKEGKKYINLLNSLWFSFSFLDVINKMREENIDNRMFLVRNVVRWRKLEDKHLKFSRRRKVAIADVLPRLQKMVKANKHYDLCTVNFTGIRSLGTFSEHQTNATNAASVQTYTIIVDEKKQVQVLNNALCTISLEDKFLSAELDRNLKKITLTAEEFTNAQKIYFATVVTRMVLLGEKRFSRMATVANPKGIKARVQQKVEPEKLSLMKEVRIGCTQMYQKDKFSKLHDGVMRILLRDEDDPFILSRKIFKQAGSSIDWLVNVDNLHFLSEKNSTNQDAHTARLIFEVSAFGFHDILEEAEKSRVQSPRRSRANTLTSPKRCVVDKKVPFSGHTEKGVYYLMAVRKAARFPWFRVQKTENELVQLARNGKWFEESVITHIADSCFYRKIVHRSRLVAIQAIAAEREAKIAAARAAGLIVDDTRNSAVGKRKVYGLDFVQSEGHGPFLKNIYRTRDTTHIPPLIPKKDTMEEVDLSERNDPVFSLGNAPLCNSLSLRLQERIHIPKKYENHGSEMSVVNKPASQVRRDVHNTKLKAVVVKKSIVVPRKKNPAHVVGALDTDLDGRPFPTTCEIFFPYAKHETYNMLLKDYIVSIGWRGGSYSSEQKSRLLSGFIEQNYEMIFEHGLENVYLKHLQVVGMHSFNWKQIHYTAPRDKYLDLKKKWTEQHCPSTSRKN

***Caenorhabditis japonica* MES-3**

MNRSDAHHIARKRRKEDEKENRLNSASTTPSTTSREESCQQSSNSALLSLTEAVCRRFERKPRKSTPVKWLDRKPVVFTKNADHLFQYYTKLGNKCSRGGFLTKNVMEKRDLLGAEKMLQMNLRCRERNAPPLGTVFENLAKIQMSKSAEKIMFTVNFARIHSWANAVETGKEITATSMLQSYTIFIDDDDRVQVNSHLPRVIISTEVPQDSDSDSGEDGNVNSNQSKRIKESSNFNILPLKGADNEKNWYCQLRGTMFDTVQELCANFNARFPCFELTTKTDKQNNGYHSYPIDKFFEETISLENAFFYRPIAQRCFPQRTGSSTDLSLAALISVGANIGAARNVTPHSAERKFGLDFVQLKSTPSVLACVYKNRINPIIPFSENEVVPKIEVLEDVEMEDNEEQEGEEDGDPKKRGRKPKRLQMRREWYPVNNAKLYSDFILFDNVHPKLESIIEKNHEMQVVELPVSREETVSFNKKLEDIKHGRLDKKSRTPSQPRDSSTSSTVTRVVNGVDSDGRKIPTFAEIFFPHGKTVLYNMMQRFYYAEAGHRPGSFTAAQKARVLVSIMGYGRTHFNCVEKCASRHFVGRKLAVFKAGFITEHGRFVLENSLGHMFEKQVQLIATTTADWKTEHFKMAMEKYKEMRREFRQMRVISNDR

***Caenorhabditis remanei* MES-3**

MRNVMRGSLVVEKKAKCKRREMTHLEEIIKTLEEEMMKEKDLHDLCCININQVKCWNTQSDTENWATDHASVQTFVLIVDDNDRVTVIDTGLSSVSLATTSLKETISCDNWKNVKATSAEINNAKHIYYAVVARRMTDKQVAKRGASRKPITSDNTHISDPSTLNQMVSAGCIRMYEKGRPNRMDNGMSRVILVEKEDSPVLEVALSTDWMLNPRHLETLHEVNVTKQDSHLPRIVFYSNSPKMGETILATTPSTPVKKPESPRSLWFKGPNGLLPKDELNWLAKDEDEAHANRRVKSRFPCYSLYNGYFYGDTAYFTDTSLRYYDDFCLYRRSRDRGIVAGRRATGRVDTTARNVGVRRRQVFGLDFVTSGAGNYFESVIYQPRRDHEAIPPIFYEAPVRKAGKKSTKPVIVLSPRPNKESYPNNNAKLFTELHFQDTMTYDIPEELRVGTSDMAVVKIKPTENFIKVWNHKLENQPVIRKSKKNKKKVVVVPPRRPNPENRPDESDRDLDGRKIPTFSQMFFPSGKTEIYDMMLRFYLSMTGIRPGSFSADFKSRLLAGFISEYSDYVFQYNLGDMFEKQVQLIGLYTLDWKKHHYDLPRDKFFELKAEWEQKQQPKP

**MUT-16**

***Caenorhabditis brenneri* MUT-16**

MSHSDDDYPELDVSTDGNENALTAADGYGEVVGAPPSSDVESDESAYHTPSSEENVIRQYEYNECDLYDSGSEEFDVEGYENEQLADLHIHDMSSYVDVGNEQESNAAHNFQEFEDSNKEIFQVLLPSAMIASIEHMTIGFEPSVINDCLKDVGYCLEVVAMMLLGAEKFATIPRAPVDWCHALKDANHLLFENGKFYPRVPESLRAHCTSLIEGASRLSQFEEKKKKEQETYKSEEFELRVLYTFNMIAELLNAVRDEQRQLKIGYSHLMNAYQAMVEGLKYHDIFKRYKGTLSLNDPKKLWNSQWFNEYTGRSSLMKFIHMARFSEIAVTNTNPRTYYFRADDDVPQRDVKLFIKKDFDEVREKWRSGNQANRNFGRNYGAGGQRGEGRGPSQQKKKTIEQDPNYKSARFNGLMDGVDDGSLEPSTSTRYNRERSPPRPIRNRSLSPRENRSPIPARSPSPFDEPTSQAQEQAARPSSPESQSDPPQRVALSDPFGGRPVQRREETNFGGSSDNQLTRDNYGRNRAGEVRRIDVHLIGETSSEGEITSDGSYSNEDEDTKQAKRERRLAVKKKKDEREMRRRKQHEENRNKPIEQTRFSVSQFKKKTPQNAPRSPERRRQESPLPFEDEPTTSTEPLPPVELPVEEPTPVQTTTADSDDEPLPVRPQATNKAPAVVTDTMTNALSHQAQVPYRPSALDVQKKKMEQEEKAKKASMNAVKKYGADAVLAYHPALFSQNSGPGPSSSPLPPPQAYIEPEPPSGFGNSQRFVDYIKRFFV

***Caenorhabditis briggsae* MUT-16**

MSGLCENSFEQRTVLIGSHLGISATGLVMTSQEDDYPDDFDITTSEQGDADDIQNHCLGAPPISDFDSNEEQDSENELEDSYDSGSEPFDVDGYDYTTEPLPAMPDDLVGNGAQNYADMNDFGGDPRELLPIFFTSTLAAHPLRLKEGYDPEELDKCLRENMGFTLPVVATILLGSHITDGLPNDSKTEYAIALANKNWLTRNGDKFLPIFPESEKEFITSLIEGSELLASKEETKKKEAQVFPSMEKEIRAHQTFNMIVELLQIVRDELRQRKVPYQNIMFAFNEMAKGKKHAHVFEKYSKQLELDSTMEWNTKWFNQYTNRSTLKKFVQMSKFSEIVVSNMKDSPFYFRADDEGDRPVKMFTDDDIKAVKDKWMSGNERNKNFGSNYERSGQRGDRGGPSRYQQQPKKVIEQDPNYRSSTFNGGISGAANDDGSLEPSSSSRTYDNQPSSRTRCHRSPTPSPERRRENPVAERSTIRDESTQRRSESPPPVTREVFDPFGGGPPLNQKTEDNRGNNRGRGFGNGNFSSLAKPVARIDHIGDTSSETSSRFNTAYSPPRRRTPSPKPRRNVSAEPEIPSPQPQPTQNQTPSPFLGQESPPSIDNDRMDDYMPPPPAAPVVRQLPLAVSSSRHDAEHMTAQVPYRPAEGVLERNKAEEEKKKRRQEIDDVRIESENRQPPTQHPFLTSSRAKPTTSRNPEQGYSDPVTTNGFARRNVAQPTYAATMAAQAPPAPPAPAPQPSPVPPPVFPLHAPMQAPLQVPVSRTNSVIKIILKPSILEEIDKEGGHLNRGYPLPPQQYPPQNQYQPPLQQQIQQPNYQYPQQNQYQQGPPPPQYPMETPQYNPTPPPPPRAEYSSNYPPPQNQMNRPPQQSYQDQQYSRNQYSSNGNWSNPAYPQQPRSPPLPNGPPQDYNRWRDRDPPPRQPPPATYGRRDVGRPSLSTFSLLAEEDDRRAAPQRPDVGRMRDIIMSIAYNCRTKGCQLDKERLKYEVCQSRFQQHFPGGPEWFDFTSFIRNELRGTMEVRGNENGAVWYELLRN

***Caenorhabditis japonica* MUT-16**

MSDESYADLDLDSSYNEEEQPVQNHVIGPAPTSSEDDEMDTRSVDSYMSGESDYDLENATPYIEPSVEELAKLDENLEPELPEHLIAAENYSDDIYASNSNEYVETVIKVRVKKPHSRATFKQNLQYFLPAAICSTPLLLRNGFSPDEVDKCLTDEGQISLEATAYILLGARIVENLPNKSKTEFCHALVKAGWLTEKQGKFFPVLPESEKEFILELIDGAENTKYYEERKKKEAEQYPSEEAEIRAHITFNFICELLSAVQRELQQNKIKYQTLSTEFEKLVSGQKNANLFSKYKHLLDLESDAKWDSEWFKKYTNRSSLKKFVTMPKFAAIVADQTSEFRFRTDNVHLFTNEDLDEVRQRWRRPAARADRRWQSGSQNAKSRENVPDPDYRPAAVSGGISALDEDDDGSLRPTTSSNHRNRHHSPTATIRDSSRTRPTTSSHPARSRSPSITTKTTRTRRDSSENPPPEPLNMEPAANPFGGPLPPPARAEHRPAHQPPPPNFPRTADYAGYSSTEDDITSDGSYTDEKSEEKERRRDDRQKKLAKKQKKLMERQNAPPKPPPDN

***Caenorhabditis remanei* MUT-16**

MTEVNDEDYPEELDLSHTTEENEGIVGSVPSDEMHFIGAPPGSDSEVDEEYRQYQRDPYSDYSSSDNEFDCDDYLKNVPRELPELTNDLIVSGSDNTRLQELQDLGDPVEFLKVFLTASMMGQPLKVKDGFDPEDLDKCCSELMGITLTGVASVLLGAELQNIPNRSKTEMAFALANRGWLRAVNSKFYPVIPETENDFVLSLIEGAERLQHLDENKKREAEHYPSAEKEINALISFNMIVELMDAVREEYRIMLLPYQTLSNGYLDMVTGKKYPEIHKKYFNKLNLDEKKVWNSEWFNAFTGRSTLKKFVQMARFAEIVVVENPTSQFCFRADSENRPVRMFTANDIKDVEEKWSSGNEKNKNFGRNYGGQGQRGLPRGASQQPKRKIEQDPNYRSSTFAGGINDDDDDGSLMPSTSARENQAGPSSSSQPVYGRRSPSPTVRNQRARSPSQESRNSNTVPQSSYRPIDPFAGAMVHPISAGVNGNRNGQGGYETRRLQTFGASSSESDEVSEGCYSDDDPEDQKRKTIERNEKRLRKEAREMERRKKHKAEVHVPPVTRFQTNPFYKHKSARAPEPANPRASTAVEQEEREELVVPPRQSPFDAPSALAPAPAAPAPVSQPSNSDAQVNAHEDVLPPPPAAVAANTLPLFVSSSTHDQDRCSAQMPYKPAEGVLERNKQEEAERLRREQLSEMRMRSGFNQVPASHPWMSAASKPGPSQPQAYEEPAPTKGFGTTLPPTVQRSVPQPPPQQAPQMQQQQEPIYSTPFVETVSLSSLIVLNNSLGFQYQGRNRQGMAPQNYAQPAPQNYAQPAPQNYAQPAPSQQQYPQHQNLVQQNPPLFPDHSASQPQYQPMQQQYQQPLPQNPYPIGSQSSSTLPQQQPYHHQNQDTYRPTSRNHANPQHMDNQYSSMRDAMRPPVPRNDMNYDSIPSNSLHQIPPMNQPQYSSQQRQYNDVYPPVNPYNNRVPSTDFNDRRIRNEQWHQVQPGSYDPYGRSQNPGPGSSYNPRGEENCASFFGRLRKAAAASGGGESEEVKQIKIEIQRIVFDYASENRELTLSELKANLVRKMRHLQFFDVHEFIQTYLRNQVVIVNNGPYGPVVRPT

**RDE-2**

***Caenorhabditis brenneri* RDE-2**

MDVVEDESNAEKRITHGTYNMPDRRFLKDVSGKTYERFQSERARKGEQEETALCTVVQKFEGMCLLYTAKKDVLNVLLYEKKCDGVKEPLQLGQCAFFKILPRQNETQDELLPRAPYTHIAVMMKEVTPESQEKITFFQQSVRCYGGLLEMCVKIKLTQGGKVFFHYEDDDQIRSDEDRRFYYLKATNGVLVTIPCQRIITLLNKDLSADFDLVAWVTHRRAVGNVSLHIGKIGKAYREFKDGSMKELEPVCTHWQSGGRK

***Caenorhabditis briggsae* RDE-2**

MYGGYPNQNYPNQNNYPNQNYPPPQQNYQQNQQNYGQQPQQPQQQYNPNMHQYNPNVQQYYNNPPGPPRPFVFMNMMPPNYYYQAPPMMPPNQYYQQMPPQAQFQQVYQQRPTPARAPPQPIPPHYHAVIQHQQIPQSISYPVRERPESPNKTFIHGVIIGKSKDGKDNFVLYSAKCGLCHISHEVPDLFIGQWLGITVSIPNYNGDVLLDRSNSEGYEDRDDLPDPMYTCVRYLPTREDPSLGEVVVTCRFRVFPDHETRGWAVHSYDIMFSDIIDDYKLISTSNDWFNGRIFIVEAQASMAGWVALRVTEDQRKEFSCDAVSIPDGKRKLPSPLVDFNIQWLEDCDPALLKIMTGNGYVQPPEHALPPPPQDFFDELGGSVGLNQPSTSATMASESSERELRQIDACISRATLTQDTVPAPPQTTLMAEKEETTTQQKPSESKEVLSDDEIDDEDTDGALGTHGMVKKEFLKDVSGKTYQRFQQERARTDQKPESALCVVVQKIDRIAVMYTAKRDVQNVLLYEQECEGVHEPLHLGQIAYFEISPRRMETQDELLPRAPYSHIAVRMKQYAPEFQSKFDRFKRLVRCFGGLVEMKVTIPLTRKGALEIFHLPEEESTHEQDKTFYYLHATNGVRVSIPWDRLVPFLKTDMTADFDLVAWASHRKAVGTVNLHIGRSGEAFRKYKDGNVEELPPLSASRYLMNAKKL

***Caenorhabditis japonica* RDE-2**

MSGWNYYAAPQYPTPRGYSTQVQTPRTWLDLYALSENYQGEYVLDEQNSYGYDRTNIEPNRQYAAARFFPSKYSDDLFLGEVLITARFGVKFNDASRKLEYISYDVNIEHLIDPSGLIEASEPLYHGRSFIVEAQASLQGFLILAVTEDPHIKLDCGHIMTADGYHKSSSTPPASNSQHNFQSPDVPLASSSEPKLGEIDCASSSRPSSETAIRQQTPTTIDASKVEVPEYDSEDDYDEENTALTLGTANIPAKQFLNQLEKKTIERFKMAKGMLDNVQSALCVVVQIIPGGALMFTVKNGVRNVLVLESGCEGLEGPLRLGDVAFFEISMRRTETRDDLLPTAIYSHVAVRRKPTTPLTETKINTFKNSIRTFGGLIEMKVKIRLTERGSVQHFYDDDQTFTKTESERKIFFLTSTNGLLVTIPNERIIELLDENLMAKFDLVAWAVHRKAVGNVCLHIGKDGEAWQKFVDGKLRELPTLSNNYHMNR

***Caenorhabditis remanei* RDE-2**

MFRNTKNSENLKILILSQNLATSHYKLHTFLSRLVVQSPEFHGEFILDYTNCVGFEIRDTLPDPMYASAKFYPSRHDPNNGELIITARFSVHANHQTMKFDVHSYDIEFLHIIDDFQMIKGSDQFYDGRSFIVEAQASLGGWLVLRVHEDSNRQFSCDAVSINPGKRRLLYPIIDFNIQFLEDCCPALIPIMTGDGWPHPPQGAPPQQPVFHEEAGSYGQINSSSSTNSHLAPSQIELREITESMSATSISQQAPPTPMAPPTPVAPPPPPQPTVDVTGDSDEEIDDEDSEGTMGTSTIPAKEYMKDVAGKMYQRLIDERPLTGQQPQSALCVVVQQIDKCALLYTAKRDVQNVLLYEKKCEGLPDGRSPELGTIAFFEILPRLMETQDELLPRAPYSHIAVRMKPSSTPESLEKIARFQQKVRCFGGLIEMKVRIPLTQPNNVSIYHPKDEELVNGDDKTFYYLKATNGVIVSIPSERLEPHLDANFQAEFDLIAWVTYRKAIGKVQMHIGRNGEAIRKWQNGRIDELPPLSANNYLMNGTAEKLKKLNFSSKNTQNPSKFVKNRDF
